# Molecular and proteome analyses highlight the importance of the Cpx envelope stress system for acid stress and cell wall stability in *Escherichia coli*

**DOI:** 10.1002/mbo3.353

**Published:** 2016-04-02

**Authors:** Kristin Surmann, Emina Ćudić, Elke Hammer, Sabine Hunke

**Affiliations:** ^1^ Department of Functional GenomicsInterfaculty Institute of Genetics and Functional GenomicsUniversity Medicine GreifswaldFriedrich‐Ludwig‐Jahn‐Straße 15AGreifswald17475Germany; ^2^ FB 5 MicrobiologyDepartment of Biology/ChemistryUniversity OsnabrückBarbarastraße 11Osnabrück49076,Germany

**Keywords:** Cpx RAP system, *Escherichia coli*, proteome profile, SRM, two‐component system.

## Abstract

Two‐component systems (TCS) play a pivotal role for bacteria in stress regulation and adaptation. However, it is not well understood how these systems are modulated to meet bacterial demands. Especially, for those TCS using an accessory protein to integrate additional signals, no data concerning the role of the accessory proteins within the coordination of the response is available. The Cpx envelope stress two‐component system, composed of the sensor kinase CpxA and the response regulator CpxR, is orchestrated by the periplasmic protein CpxP which detects misfolded envelope proteins and inhibits the Cpx system in unstressed cells. Using selected reaction monitoring, we observed that the amount of CpxA and CpxR, as well as their stoichiometry, are only marginally affected, but that a 10‐fold excess of CpxP over CpxA is needed to switch off the Cpx system. Moreover, the relative quantification of the proteome identified not only acid stress response as a new indirect target of the Cpx system, but also suggests a general function of the Cpx system for cell wall stability.

## Introduction

Two‐component systems (TCS) represent one of the prevalent signaling mechanisms in bacteria. These systems allow bacteria to cope with rapid changes in cellular or environmental conditions. TCS typically consist of a sensor histidine kinase (SK) and a response regulator (RR) (Chang and Stewart 1998; Hoch 2000; Gao and Stock 2009). The sensing of a specific stimulus results in autophosphorylation of the SK at a conserved histidine residue located in the dimerization and histidine phosphorylation domain (DHp) of the SK. After autophosphorylation, the SK transfers the phosphoryl group to a conserved aspartate in the receiver domain (REC) of the respective RR. The phosphorylated RR mediates the cellular response by acting in most cases as a transcription factor of target genes. In order to terminate the response, the RR is dephosphorylated by either intrinsic activity or by the SK, in the case of a bifunctional SK (Stock et al. 2000; Gao and Stock 2009). Some TCSs are additionally modulated by an accessory protein (Buelow and Raivio 2010; Heermann and Jung 2010; Krell et al. 2010). These accessory proteins are found either in the cytoplasm, the inner membrane, the periplasmic space, or in the outer membrane, such as NlpE, and modulate the activity of the SK or the interaction between SK and RR. The effect of accessory proteins is modulated via the protein level, modifications, and substrate binding. Hence, accessory proteins allow for the integration of additional signals and can link a given TCS to another regulatory system.

One prominent model system is the Cpx‐TCS (*c*onjugative *p*ilus e*x*pression), which detects and responds to perturbations of the cell envelope and was first described by McEwen and Silverman in the early 1980s (McEwen and Silverman 1980; Hunke et al. 2012; Raivio et al. 2013). The Cpx‐TCS belongs to a network of so called envelope stress systems including the Bae, Psp, Rcs, and *σ* ^E^ pathways that slightly overlap with respect to detected stimuli and regulon composition (Bury‐Mone et al. 2009). The Cpx‐TCS itself consists of the membrane‐spanning SK CpxA, the RR CpxR, and the periplasmic accessory protein CpxP (Jones et al. 1997). CpxA possesses both kinase and phosphatase activity, and its autophosphorylation activity is inhibited by CpxP (Fleischer et al. 2007). The Cpx‐TCS is induced by various stimuli including misfolded proteins (Snyder and Silhavy 1995), alkaline pH (Danese and Silhavy 1998), salt (Raivio and Silhavy 1997), changes in lipid composition (Mileykovskaya and Dowhan 1997), or attachment to abiotic surfaces (Otto and Silhavy 2002). Importantly, the outer membrane lipoprotein NlpE (new lipoprotein E) is required to activate the Cpx‐TCS in response to surface attachment (Otto and Silhavy 2002). Moreover, overproduction of NlpE activates the Cpx pathway and can be used as a Cpx‐specific activation signal (Snyder et al. 1995; DiGiuseppe and Silhavy 2003). In contrast, CpxP inhibits the Cpx‐TCS in a negative feedback loop (Raivio et al. 1999). Thereby, the *cpxP* gene is the strongest induced CpxR target gene under envelope stress conditions and the strongest induced gene during the initial phase of biofilm formation (Danese and Silhavy 1998; Beloin et al. 2004) However, direct inhibition of the Cpx response by CpxP is only monitored after overproduction of CpxP (Raivio et al. 1999). Nevertheless, deletion of *cpxP* results in mild induction of the Cpx response, which can be fully induced by other stimuli (Raivio et al. 1999). In addition to its inhibitory function, CpxP is important for the quality control of misfolded envelope proteins that go “OFF” pathway during biogenesis (Danese and Silhavy 1998; Hung et al. 2001; Isaac et al. 2005). It is established that the inhibitory and the quality control functions of CpxP are linked. A CpxP homodimer interacts directly with CpxA and thereby blocks CpxA autophosphorylation (Fleischer et al. 2007; Zhou et al. 2011). Salt stress and misfolded pilus subunits disturb this interaction resulting in CpxA autophosphorylation and subsequently in an induced Cpx response (Isaac et al. 2005; Tschauner et al. 2014). However, it is so far unknown whether the absolute amounts of CpxA, CpxR, and CpxP or their stoichiometry within the cell are involved in switching the Cpx‐TCS from “OFF” to “ON.”

Here, we determined the absolute amounts and stoichiometry of CpxA, CpxR, and CpxP to better understand the functionality and dynamics of the Cpx‐TCS. We addressed this question by quantifying the absolute molecular amounts of CpxA, CpxR, and CpxP for cells grown under noninducing, Cpx‐activating, and Cpx‐inhibiting conditions applying the selected reaction monitoring (SRM) method which is also known as multiple reaction monitoring (MRM). This targeted MS approach has been applied in the recent years more frequently for absolute quantifications of proteins, for instance in microorganisms (Schmidt et al. 2010; Gallien et al. 2011). It was already successfully utilized to quantify partners of the KdpDE‐TCS of *Escherichia coli*, as well as the Kdp(F)ABC potassium transporter (Surmann et al. 2014). We additionally aimed to gain deeper insight into the biological function of the Cpx system focusing on its influence on the level of other proteins. Therefore, we compared protein levels between the *E. coli* wild‐type and a *cpxRA* deletion mutant grown under noninducing or Cpx‐activating conditions by a gel‐free proteomics shotgun approach. Our results show that the absolute protein levels of CpxA and CpxR are only barely changed by different growth conditions and that the stoichiometry between both proteins remains constant. Moreover, our data reveal that a 10‐fold excess of CpxP is needed to promote Cpx‐TCS inhibition. The relative quantification of the proteome highlights not only envelope stress response and peptidoglycan (PG) modifications, but also identifies acid stress response as a new, negatively regulated target of the Cpx‐TCS.

## Experimental Procedures

### Bacterial strains and plasmids

All *E. coli* strains and plasmid used in this study are described in Table 1. *Escherichia coli* strains used in this work were derived from MG1655 using P1vir transduction. Strains were grown in LB medium (Miller 1992). When necessary, antibiotics were included at the following concentrations: ampicillin (Ap), 150 *μ*g/mL; chloramphenicol (Cm), 20 *μ*g/mL; kanamycin (Km), 40 *μ*g/mL.

**Table 1:** *Escherichia coli* strains and plasmids used in this study

Strain/plasmid	Relevant genotype	Reference or source
MG1655	F‐lambda‐ *ilvG*‐ *rfb*‐50 *rph*‐1	Blattner et al. (1997)
EMC07E	F‐lambda‐ *ilvG*‐ *rfb*‐50 *rph*‐1 *cpxRA::kan*	This study
pT*nlpE*	*nlpE* cloned in pTrc99A, Amp^R^	Zhou et al. (2011)
pT*cpxP*	*cpxP* cloned in pTrc99A, Amp^R^	Zhou et al. (2011)
pTrc99A	pBR322‐derived plasmid, Amp^R^	Amann et al. (1988)

### Collecting bacteria for shotgun proteomics and SRM

*Escherichia coli* cells were grown aerobically at 37°C in LB medium to an OD_600_ of ~0.5. Subsequently, IPTG (isopropyl‐β‐d‐thiogalactopyranoside) was added to a final concentration of 1 mmol/L to induce the overexpression of either *nlpE* (pT*nlpE*) or *cpxP* (pT*cpxP*) in MG1655 (WT) and EMC07E (*cpxRA::kan*). The efficacy of *nlpE* overexpression was validated by mass spectrometry as described earlier. Relative quantification of NlpE revealed distinct increased levels of this protein after *nlpE* overexpression compared to noninduced samples (Fig. S2). After additional growth to an OD_600_ of ~1, cells were harvested by centrifugation at 5000*g* for 10 min and immediately frozen at −80°C. The number of cells per milliliter cell culture was assigned using light microscopy and a Thoma chamber.

### Cell disruption and determination of protein concentration

Cells from 10 mL culture grown to an OD_600_ of ~1 were resuspended in 150 *μ*L buffer containing 8 mol/L urea and 2 mol/L thiourea and disrupted by ultrasonication (50 W, 3 × 30 sec, on ice). Samples were centrifuged (20,000*g*, 4°C, 1 h), and protein concentration in the supernatant was measured using a Bradford assay (Biorad, Munich, Germany). Absolute amounts of protein per *E. coli* K12 cell were determined as described previously (Surmann et al. 2014). Briefly, the number of bacteria per mL cell culture was determined in a Thoma chamber using light microscopy. Subsequently, the total protein amount determined by Bradford assay was correlated to the bacterial counts. Applying this method revealed the cellular amount of protein of *E. coli* K12 to be 1.4 × 10^−^7 *μ*g (average from four conditions and five BRs).

### Absolute quantification of CpxA, CpxR, and CpxP by SRM using heavy labeled standard peptides

Targeted SRM analyses mass by charge ratios (m/z) from pairs (transitions) of peptides of interest along with the corresponding fragment m/z values. Since other masses in complex samples are ignored, this method is more sensitive than shotgun mass spectrometry methods. Therefore, it has gained high popularity concerning quantitative proteomics (Gallien et al. 2011). To determine absolute numbers of molecules, heavy labeled standard peptides can be employed that contain ^13^C‐ and ^15^N‐arginine (Arg) or lysine (Lys) and correspond to natural peptides of the target proteins (Schmidt et al. 2010). Accurate absolute quantification of molecules by SRM requires careful sample preparation and method development. Here, we employed a protocol proven for reproducibility and exactness of cell disruption and digestion efficiency (Surmann et al. 2014).

For the absolute quantification, heavy standard peptides (SpikeTides^TM^TQL) containing ^13^C and ^15^N arginine or lysine were obtained from JPT (JPT, Berlin, Germany). Heavy standard peptides contain a C‐terminal amino acid tag that enables quantification during the manufacturing process. This tag was removed by tryptic digestion during sample preparation. For each of the target proteins CpxA, CpxP, and CpxR, two or three proteotypic heavy standard peptides were ordered. As described previously (Surmann et al. 2014), 1 nmol of each lyophilized peptide was dissolved in 100 *μ*L buffer consisting of 80% (*v*/*v*) ammonium bicarbonate (ABC) solution (100 mmol/L) and 20% (*v*/*v*) acetonitrile (ACN). Dissolved peptides were stored in aliquots (10 pmol/*μ*L) of 10 *μ*L at −80°C until utilization. For all standard peptides purity was controlled by LTQ Orbitrap tandem mass spectrometry analysis and subsequent search of spectra against a complete Swiss‐Prot database and found to be pure. Other identifications were only trypsin and keratin (<1% of the total intensity). SRM analysis of the standard peptides confirmed the complete incorporation of heavy isotopes (99.98–100%). However, a peptide of CpxR, peptide GSELDR, was not detectable as a heavy peptide, because the C‐terminal quantification tag could not be removed by tryptic digestion. Since the 26‐kDa protein CpxR does not contain more tryptic peptides fulfilling the requirements of SRM, it was only quantified by one peptide. Nevertheless, peptide GSELDR was detected in the nonlabeled sample and supported the protein identification. The heavy peptides were then utilized to optimize the SRM acquisition method by defining the optimal gradient and collision energy (CE) targeting the highest peak intensity for each peptide. Afterward, the standard peptides were spiked in different concentrations into sample background. This enabled us to determine the linear range between concentration and peak area for each peptide. The detection limit for the peptides ranged between 0.01 and 0.05 fmol/*μ*g protein. The correlation coefficient between concentration and peak area amounted higher than 0.99 for each peptide over the whole detectable concentration range, proving the accurate quantification within the range of applied spike‐in concentrations (Table S1). Furthermore, the correlation between concentration and peak area aided to determine the most suitable peptide for the quantification of the protein amount as described earlier (Surmann et al. 2014). For each protein, one peptide was chosen that exhibited a standard curve with the best signal to noise ratio. The standard curve provided additionally the actual concentration of the sample peptide, which is important to allow for the final quantification of the actual sample protein, by so called one‐point quantification (Surmann et al. 2014). Finally, for each BR two technical replicates were prepared and spiked with two different dilutions of heavy peptides corresponding to 0.5 and 10 fmol/*μ*g protein. This procedure enabled the coverage of the complete dynamic range within our samples. The ratio between light and heavy peptide, whichever was closer to one, was always used for absolute quantification of the peptide in the sample. All values for each peptide and replicate are provided in Table S2, which highlights peptides and spike‐in ratios used for final quantification of the proteins. The heavy peptides were spiked into the samples after determination of protein concentration, but before digestion (see below). Doing so, technical variances were minimized by processing sample and standard with the same protocol.

### Sample preparation for mass spectrometry

For each sample, 4 *μ*g of protein was diluted in aqueous 20 mmol/L ABC to a final urea concentration below 2 mol/L. When samples were prepared for SRM analysis, heavy peptides were spiked in an appropriate concentration at this step. Subsequently, proteins were reduced with 2.5 mmol/L dithiothreitol (DTT) at 60°C for 1 h and afterward alkylated with 10 mmol/L iodoacetamide (IAA) at 37°C for 30 min in the dark. The final urea concentration was decreased to 1 mol/L with ABC to ensure the efficiency of trypsin digestion. The protease was added to the sample in a trypsin to protein ratio of 1:25 (w/w). After overnight incubation at 37°C, digestion was stopped with 1% (*v*/*v*) acetic acid and samples centrifuged (10 min, 16,000*g*). Peptides in the supernatant were desalted and purified using *μ*C18‐ZipTip columns (Merck Millipore, Darmstadt, Germany). Elution buffer was evaporated in a vacuum centrifuge and peptides were dissolved in 20 *μ*L 0.1% (*v*/*v*) aqueous acetic acid containing 2% (*v*/*v*) ACN. In a previous study, digestion efficiency of this protocol for *E. coli* K12 cell pellets was determined by 1D gel analysis with silver staining and estimated to be >99.9% (Surmann et al. 2014). Samples were short‐term stored at −20°C before shotgun MS or SRM analysis.

### Data acquisition by mass spectrometry

Shotgun LC‐MS/MS analysis was carried out for four independent BR per condition. Peptide separation was performed on a NanoAcquity BEH130 C18 column (10 cm length, 100 *μ*mol/L inner diameter, and 1.7 *μ*m particle size) using a nanoAcquity UPLC (Waters, Manchester, UK). The separated peptides were ionized using electrospray and analyzed with an LTQ Orbitrap Velos mass spectrometer (Thermo Electron, Bremen, Germany) operated in data‐dependent mode. Up to 20 most intense ions were sequentially isolated for collision‐induced dissociation (CID) in the linear ion trap. Further details are provided as Supporting Information.

Prior to SRM analysis, peptides were separated with a nano‐HPLC (EASY‐nLC, Proxeon Biosystems A/S, Odense, Denmark) with the help of an Acclaim PepMap 100 reverse phase column (3 *μ*m, 75 *μ*m i.d × 150 mm, LC Packings, Dionex, Idstein, Germany). SRM analysis was performed on a TSQ Vantage (Thermo Electron). By targeted analysis separated and ionized peptides (precursors) were analyzed in the first quadrupole. Subsequently, they were fragmented using CID and products were analyzed in another quadrupole. Appropriate CE was optimized on precursor level starting from factory defaults (depending on the m/z ratio of the precursor) by applying different eV in steps of + or −2 eV. Settings for final SRM analyses are provided as Supporting Information. For each peptide, the doubly charged precursor and the four most abundant product ions (transitions) were chosen for SRM acquisition (Table S1). SRM data were recorded for five independent BRs for each condition.

### Analysis of proteome data

Mass spectrometric data of *shotgun* analysis were analyzed with the Rosetta Elucidator software (Ceiba Solutions, Boston, MA). Proteins were identified by an automated database search against a Swiss‐Prot database rel. 06‐2014 limited to *E. coli* K12 entries using Sequest v. 2.7. Relative quantification of differences in protein levels depending on the culture conditions and overexpression experiments of *E. coli* was based on summed intensities of aligned single isotope features representing peptides (PeptideTeller probability >0.95). Only proteins that were identified by more than two peptides or one peptide when the sequence coverage measured was at least 10%, respectively, were considered for further quantification. Utilizing the Genedata Analyst software v8.2 (Genedata AG, Basel, Switzerland) intensity values were median normalized and statistically analyzed by two group *t* test and multiple testing corrections according to Benjamini–Hochberg. Ratios in comparison to the WT were calculated from the mean of all BRs per condition. Ratios to the WT with values <0.5 or >2, along with a multiple testing corrected *q*<0.05, were regarded as regulated in the corresponding condition.

Setup of transitions, method optimization, and quantification for SRM were accomplished using the open source program Skyline v2.5 (Maclean et al. 2010). The final transition list is provided as Table S1. Quantification was performed as published recently (Surmann et al. 2014). Pairs of heavy and light peptides were identified by equal peak elution pattern and retention time. Several mixtures of each heavy standard peptide (0, 0.1, 0.5, 1, 5, 10, 50, and 100 fmol/*μ*g protein) in the background of WT bacteria were acquired as duplicates by SRM after digestion. These data were used to determine the linear range (R^2^ > 0.99), in which absolute quantification was allowed, and to further estimate the concentration range of the natural sample. The peptide per protein with the highest intensity and thereby best signal to noise ratio was chosen for quantification. Finally, each of the samples was spiked with 0.5 fmol/*μ*g or 10 fmol/*μ*g. The aim was to receive an almost equal concentration of synthetic and natural peptides in order to lower the dynamic range in concentration and increase the accuracy of the measurement. The heavy to light ratio, which was closer to one (single point calibration) was used for absolute quantification. The heavy to light ratios of peak areas from natural sample peptides and corresponding heavy standard peptides eluting at the same RT were used to calculate the concentration of the proteins in the different samples. Average values and coefficient of variance were calculated over all replicates for each condition.

### Acid stress sensitivity assay

The strains MG1655 (WT) and EMC07E (*cpxRA::kan*) were used to evaluate acid resistance. Both strains were transformed with pT*nlpE* (Cpx activation) and pTrc99A (vector control), respectively, and treated identically. The cells were incubated as described for the sample preparation for shotgun proteomics and SRM. After reaching an OD_600_ of ~1 the cultures were acidified to pH 2 by adding 1 mol/L HCl. The cells were additionally incubated for 1 h at 37***°***C. Several dilution steps were dropped on agar plates containing 50 *μ*g/mL carbenicillin and incubated overnight at 37***°***C. The plasmid pTrc99A was used to compare all strains on one single agar plate. Additionally, nontransformed MG1655 and EMC07E were dropped on agar plates without antibiotics or agar plates containing 50 *μ*g/mL kanamycin to exclude plasmid‐based effects on the acid resistance of the respective strains.

### Relative quantification of free Lpp

The level of free Lpp was determined in accordance to the method described by Cowles et al. (2001). In brief, MG1655 (WT) and EMC07E (*cpxRA::kan*) transformed with either pTrc99A or pT*nlpE*, and were used to determine the relative amount of free Lpp. One mL of each overnight culture grown at 37°C was collected by centrifugation at 10,000*g* for 4 min. The pellets were resuspended in 50 *μ*L 1% SDS in PBS. After boiling for 10 min the samples were separated by 15% SDS‐PAGE. Free Lpp and OmpA were detected by immunoblotting using rabbit anti‐Lpp antibody in a working dilution 1:1000 (provided by Roland Lloubes, Institute of Microbiology of the Mediterranean Life Science, Marseille, France) and rabbit anti‐OmpA antibody in a working dilution 1:10,000. The second ECL anti‐rabbit IgG horseradish peroxidase antibody was used in a working dilution of 1:5000. The detection of protein bands was performed by using the Supersignal^®^ West Pico chemiluminescent substrate kit (Thermo Scientific) and the ChemiDoc^TM^ MP imaging system with Image Lab^TM^ software (BIO‐RAD, München Germany). Band densities were quantified using Image J software. The relative amount of free Lpp was calculated in % referring to OmpA‐band densities, which served as a reference for relative cell density.

### Quantitative reverse transcription‐PCR (qRT‐PCR)

The expression level of the acid stress genes *cydA*,* gadA*, and *hdeA* was analyzed by qRT‐PCR using five BRs for each condition. Every BR was tested with three technical replicates. The extraction of total RNA was performed using the RNeasy minikit (Qiagen, Hilden, Germany) according to manufacturer's instructions. After dilution of the RNA to a final concentration of 20 ng/*μ*L and digestion of residual DNA contaminations by DNase I, cDNA was synthesized via the RevertAid First Strand cDNA synthesis kit (Fermentas, Frankfurt, Germany). The qRT‐PCR experiments were performed according to the following protocol: The first cycle (95°C/2 min) was followed by 40 repeats of Cycle 2 including several heating steps (95°C/15 sec; X°C/30 sec; 72°C/30 sec). Temperature X displays the primer‐specific annealing temperature. The protocol was completed by three more cycles: 95°C/1 min, 55°C/1 min, and 55°C/10 sec. The last cycle included a temperature increment of 0.5°C after every 10 sec to enable melt curve data collection and real‐time analysis. The primer pairs used in this study are ECO_cydA qRT fw/ECO_cydA qRT rev annealing in *cydA* (CGG GAT GAA GGC GTA CTC TC and GTT GCG GAT GAC ACT CCA GA), ECO_gadA qRT fw/ECO_gadA qRT rev annealing in *gadA* (CAG ACC TGG GAC GAC GAA AA and CAA CGC CAT TTC ATC GCC AT), and ECO_hdeA qRT fw/ECO_hdeA qRT rev annealing in *hdeA* (TGG TCT GCT TCT TCT GCC AG and ATA GCT GGG GTT ACG GTT GC). Since an internal standard is needed we used the primer pair GapA‐qRT‐fw/GapA‐qRT‐rev (CTC CAC TCA CGG CCG TTT CG and CTT CGC ACC AGC GGT GAT GTG) amplifying the *E. coli* housekeeping gene *gapA* (glyceraldehyde‐3‐phosphate dehydrogenase A). The levels of expression of *cydA*,* gadA*, and *hdeA* were normalized to the expression level of *gapA* and the x‐fold change in the expression after Cpx activation compared to WT cells was calculated.

## Results

### Absolute quantification of the CpxRAP proteins

In order to investigate and interpret the functions of the Cpx envelope stress TCS, we were interested in the absolute protein amounts of the sensor kinase CpxA, the RR CpxR, and the periplasmic accessory protein CpxP in *E. coli* cells. Especially, we wanted to investigate the protein amounts under noninducing, Cpx‐activating, and Cpx‐inhibiting conditions. Therefore, cells were grown in standard Luria–Bertani (LB) medium either without any induction or with induced expression of either *nlpE* (Cpx activation, ON) or *cpxP* (Cpx inhibition, OFF).

The first step in the SRM workflow for absolute quantification was the identification of proteotypic peptides for CpxA, CpxR, and CpxP by shotgun proteomics and theoretical tryptic digestion. These peptides had to be unique, contain at least six amino acids, but no cysteine or methionine (due to sensitivity to chemical modifications), and no missing cleavage sites were allowed (Mallick et al. 2007). Two peptides for CpxA (AEDSPLGGLR, LLLVTTEGR) and CpxR (EHLSQEVLGK, GSELDR), and three peptides for CpxP (DVTQWQK, LLTPEQQAVLNEK, LVTAENFDENAVR) were chosen (Table S1), employed as heavy labeled standard peptides, and tested for linear regression and concentration (Table S2).

**Figure 1:**
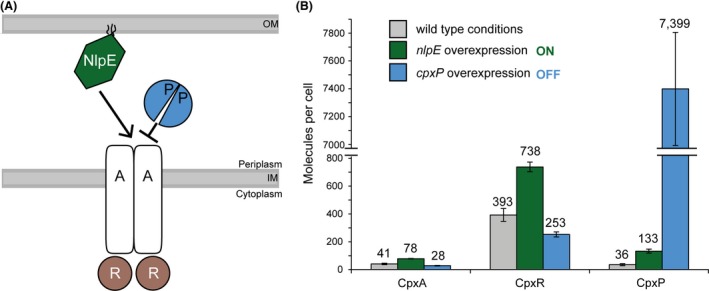
Organization and absolute quantification of the Cpx two‐component system. (A) Schematic representation of the localization and interactions between the CpxRA‐TCS and the inducing molecule NlpE, and the inhibitor CpxP. (B) Absolute amounts of the molecules CpxA, CpxR, and CpxP were determined by selected reaction monitoring (SRM) in the unstressed wild type (WT), after activation of the Cpx‐TCS by *nlpE* overexpression (ON) or inhibition of the Cpx‐TCS by *cpxP* overexpression (OFF). Absolute amounts are given in molecules per cell and represent the mean data and standard deviations of five biological replicates.

After identification of the optimal proteotypic peptides, transition pairs, and spike‐in concentrations, as well as accomplishing method optimization, absolute amounts of the proteins CpxA, CpxR, and CpxP could be determined for five biological replicates (BRs) per condition (Table S2). All three proteins could be quantified for all growth conditions in all samples (Fig. 1) even though the membrane‐associated protein CpxA was present in very low molecule numbers. Baseline synthesis of CpxA:CpxR:CpxP was found to be 41:393:36 molecules per cell. Under Cpx‐activating conditions, synthesis of CpxA and CpxR was about twofold increased (78:738 molecules per cell), whereas synthesis of CpxP was fourfold increased (133 molecules per cell). Overproduction of CpxP (7399 molecules per cell) resulted in a decreased copy number of CpxA and CpxR (28:253 molecules per cell). Interestingly, the CpxA:CpxR stoichiometry remained constant at 1:10 for all tested conditions demonstrating that this TCS is robust with regard to perturbation of its stoichiometry.

### Impact of the Cpx system on the *E. coli* proteome

In order to investigate the impact of the Cpx‐TCS on the global protein composition, the proteome profiles of an *E. coli* wild‐type (WT) and a *cpxRA* strain were acquired under standard LB/noninducing and Cpx‐activating (ON, overexpression of *nlpE*) growth conditions (four BRs each). Based on the normalized peak intensities, relative quantification of proteins in comparison to the abundance in the WT was performed. We defined that targets with a protein ratio between cells grown under Cpx‐activating conditions (WT_ON_) and in standard LB (WT) of WT_ON_/WT ≥ 2 are under positive control, and targets with a ratio of WT_ON_/WT ≤ 0.5 are under negative control of the Cpx‐TCS. In addition, the effect was abolished with respect to the ratio of a *cpxRA* strain grown under Cpx‐activating conditions (*cpxRA*_ON_/WT ratio) in order to confirm a Cpx‐dependent effect (Table S4A and B).

**Figure 2:**
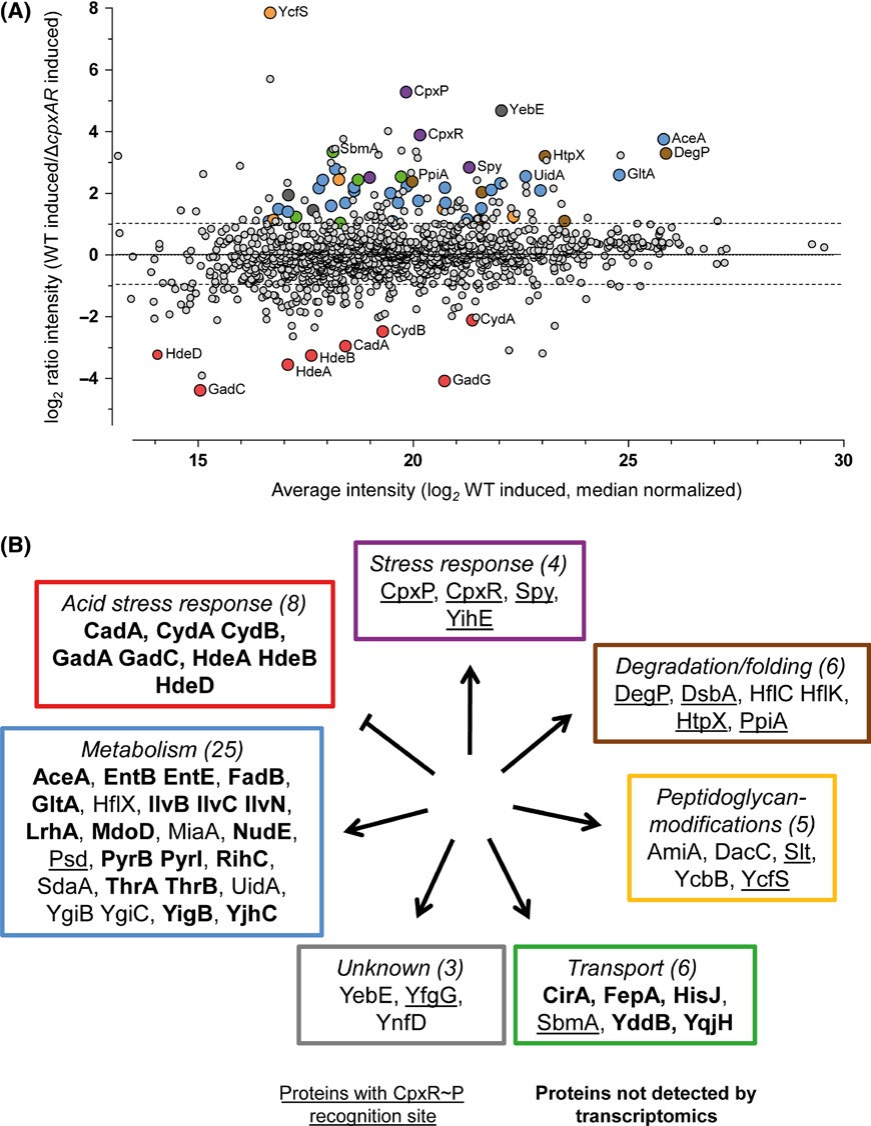
Comparative proteome analysis of the *Escherichia coli* response on envelope stress. (A) The proteome profiles of *E. coli* wild‐type (WT) and an isogenic *cpxRA* strain were acquired after activation of the Cpx‐TCS by *nlpE* overexpression. Ratio data of these two strains (four biological replicates) unraveled proteins whose levels were influenced by increased activity of CpxRA. Those proteins were highlighted with larger and colored spots. (B) Functional categorization of affected proteins. Proteins whose transcript levels are under direct control of CpxR~P are underlined. Within this study, newly identified proteins whose level is correlated with the activation of the Cpx‐TCS are written in bold letters. Colored frames around the categories correlate to the colored spots in (A).

Analysis of the 1259 proteins covered by shotgun mass spectrometry revealed that 369 proteins were altered in their abundance (ratio <0.5 or >2 and *q* < 0.05) in at least one of the conditions (Table S3A–C). Of these 369 proteins, some showed increased (98 WT_ON_/WT, 81 *cpxRA*/WT, 118 *cpxRA*_ON_/WT) and other decreased (103 WT_ON_ /WT, 105 *cpxRA*/WT, 129 *cpxRA*_ON_/WT) protein levels (Table S3A–C). Seventy‐seven proteins that were found in differential levels in the WT bacteria after induction of the Cpx‐TCS, but not in the *cpxRA* strain, were defined to be specifically controlled by the Cpx‐TCS (Fig. 2B). Functional categorization of these proteins revealed that activation of the Cpx‐TCS led to increased levels of proteins involved in stress response, degradation or folding of proteins, modification of PG, transport, and metabolism. In contrast, a decreased level of proteins involved in acid stress response was observed upon activation of the Cpx‐TCS (Fig. 2A and B). Some of the targets of the Cpx system were already known from previously conducted bioinformatics and transcriptome analyses (De Wulf et al. 1999; Shimohata et al. 2002; Gerken et al. 2009; Raivio et al. 2013) (Fig. 2B, Table S4A and B). Among the targets known to be under direct CpxR control, our proteome profile highlights (Fig. 2B, underlined) the l,d‐transpeptidase YcfS (WT_ON_/WT ratio = 28.7, but *q* value 0.06) and the proteases DegP (11.13) and HtpX (10.78) (Table S4A). Moreover, some so far unknown targets affected by the activation of the Cpx‐TCS were identified (Fig. 2B, bold). Among these were five proteins related to transport function (e.g., ferric iron‐catecholate outer membrane transporter CirA [6.02], iron‐enterobactin outer membrane transporter FepA [6.21]), and 18 proteins with metabolic functions (e.g., acetolactate synthase IlvB [8.71]). In addition, eight proteins involved in the acid stress response of *E. coli* showed decreased levels in cells upon activation of the Cpx system.

### The Cpx system reduces acid stress resistance

**Figure 3:**
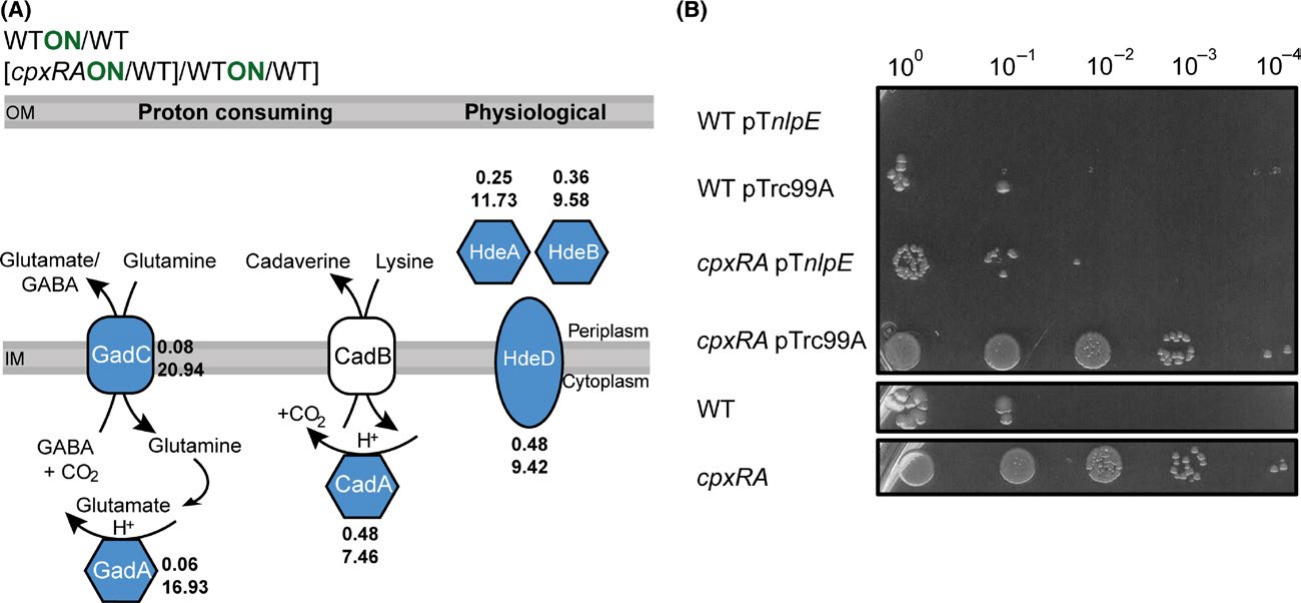
The Cpx system inhibits the acid stress response. (A) Proteome profile revealed that activation of the Cpx‐TCS affected different acid stress response systems of *Escherichia coli* by decreasing the level of the respective proteins (colored in blue). CadB was not identified by MS (white). The numbers represent the ratios of protein levels between WT_ON_/WT (top) and (*cpxRA*_ON_/WT)/(WT_ON_/WT) (bottom). The average of five biological replicates is shown. (B) The viability after rapid change of pH from pH 7 to pH 2 was investigated by evaluating the acid resistance of *E. coli* WT cells compared to a *cpxRA* strain. Additionally, Cpx‐activating conditions (WT pT*nlpE*) and WT cells transformed with the empty vector pTrc99A (WT conditions; control) were tested. Several dilutions of cells (10^0^–10^−4^) are depicted from one representative experiment of three independent determinations.

Eight proteins related to acid stress response were found with reduced abundance after activation of the Cpx‐TCS (Figs. 2A and B, 3A). Among them were CydA (WT_ON_/WT ratio = 0.35) and CydB (0.37), the amino amino acid decarboxylases GadA (0.06) and CadA (0.48), and GadC (0.08), the respective antiporter to GadA. The acid stress chaperones HdeA (0.25) and HdeB (0.36), and the acid resistance membrane protein HdeD (0.48) (Fig. 3A) were decreased as well. For validation of the proteome data, we also performed quantitative reverse transcription‐PCR (qRT‐PCR) for three involved genes *cydA*,* gadA*, and *hdeA*. The genes *cydA* and *hdeA* were also downregulated after Cpx activation. The third gene, *gadA* was not regulated on transcript level, but its production on protein level is Cpx dependent (Fig. S1). We investigated the survival of *E. coli* cells grown at pH 2 in dependency of the Cpx‐TCS because our proteome analysis identified predominantly acid resistance systems that are active under extreme acid stress conditions (pH 2–3). Since the protein amounts of these systems were reduced after activation of the Cpx‐TCS, we expected reduced viability upon activation of the Cpx‐TCS, but increased viability of a *cpxRA* strain during extreme acid stress. Consistent with this assumption, activation of the Cpx‐TCS (WT pT*nlpE*) resulted in total loss of viability for cells grown in extreme acid medium (pH 2) in comparison with the WT strain (WT pTrc99A) (Fig. 3B). Moreover, the *cpxRA* strain recovered viability in an extreme acidic medium. Viability of the *cpxRA* strain overexpressing *nlpE* (*cpxRA* pTnlpE) was comparable with the WT strain, indicating that NlpE does not only impact the Cpx‐TCS, but also other systems. Taken together, our data clearly demonstrate that the Cpx‐TCS is involved in regulating the protein level of several acid stress response involved proteins leading to a reduced viability under extreme acid stress.

### The Cpx system modulates protein levels of other envelope stress systems

**Figure 4:**
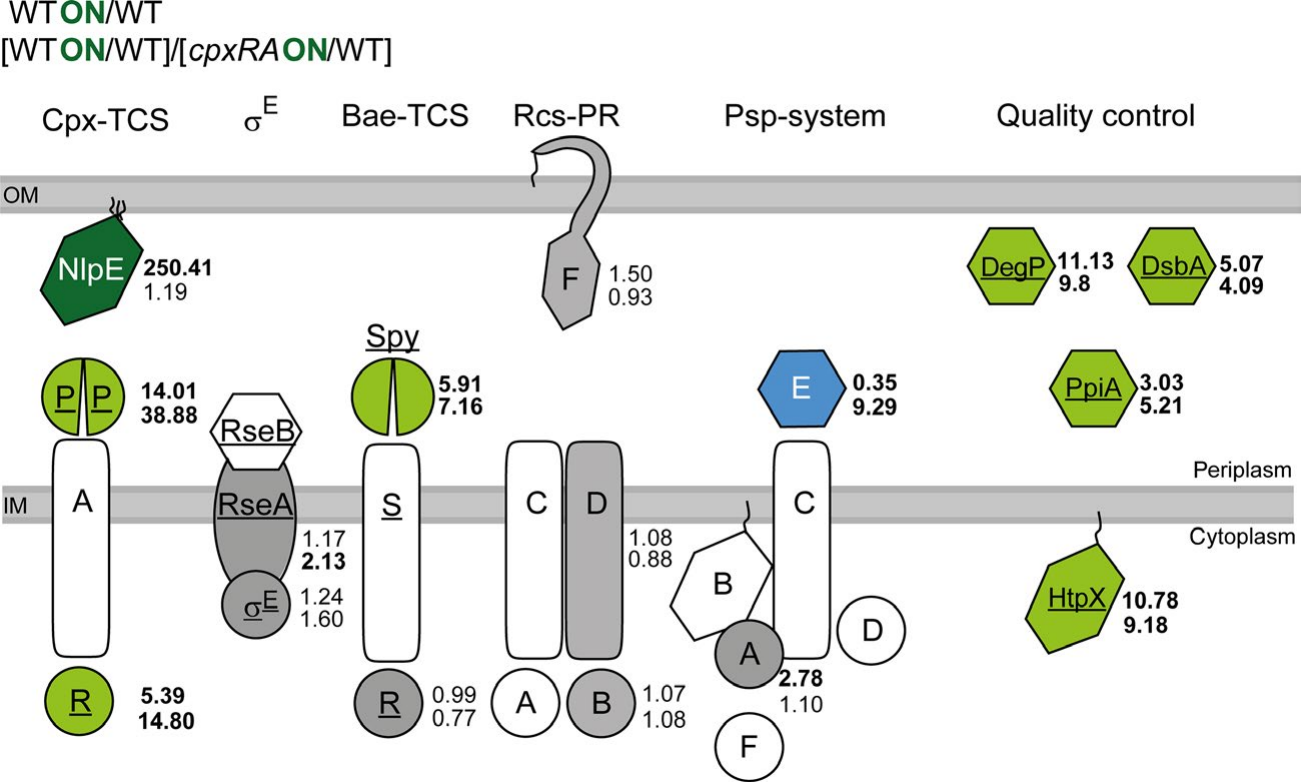
Impact of the Cpx system on envelope stress systems and classical Cpx targets. Proteome profile revealed that activation of the Cpx‐TCS affects several envelope stress systems of *Escherichia coli*. Green color represents proteins being enriched at least twofold, concerning either the ratio WT_ON_/WT or the ratio (WT_ON_/WT)/(*cpxRA*_ON_/WT). The latter mentioned ratio is shown to emphasize the higher amount of respective proteins in WT_ON_ compared to *cpxRA*_ON_. DegP, DsbA, PpiA, and HtpX belong to classical, known targets of the Cpx system, whose functions can be summarized as “quality control.” Blue coloring of PspE represents a decreased protein level after *cpxRA* induction, whereas gray‐colored proteins were detected, but either not enriched or not specifically enriched (PspA) after activation of the Cpx system. White‐colored proteins represent proteins that were not identified by MS. Dark green coloring of NlpE represents its overexpression. Underlined proteins are known to have a binding site for CpxR~P on DNA level. Numbers represent the averages of five biological replicates. TCS, two‐component system; PR, phosphorelay.

Further we investigated protein levels of well‐known Cpx targets that counteract envelope stress and determined to which degree other envelope stress systems are affected (Fig. 4). Levels of proteins involved in quality control of the envelope (DegP, DsbA, HtpX, PpiA) were significantly stronger affected (roughly two times) than expected from available transcriptome or promoter–reporter fusion data (Table S4A) (Raivio et al. 1999, 2013). The protein levels of only four other envelope stress system proteins (CpxP, CpxR, anti‐sigma factor RseA, envelope stress induced periplasmic protein Spy) were increased by the activation of the Cpx‐TCS, whereas the periplasmic accessory PspE protein was differently affected after expressing *nlpE* in dependency of the presence or absence of the Cpx system (Fig. 4). Other members of the four other envelope stress system (ECF RNA polymerase sigma‐E factor RpoE, transcriptional regulatory proteins BaeR and RcsB, phosphotransferase RcsD, outer membrane lipoprotein RcsF, phage shock protein A PspA) were detected, but their amount did not alter after activation of the Cpx system. In sum, the proteome profile shows that the effect of the Cpx system on the level of some envelope stress proteins and important quality control proteins is stronger than estimated from transcript levels.

### Activation of the Cpx system results in increased covalent outer membrane PG stabilizing cross‐links

**Figure 5:**
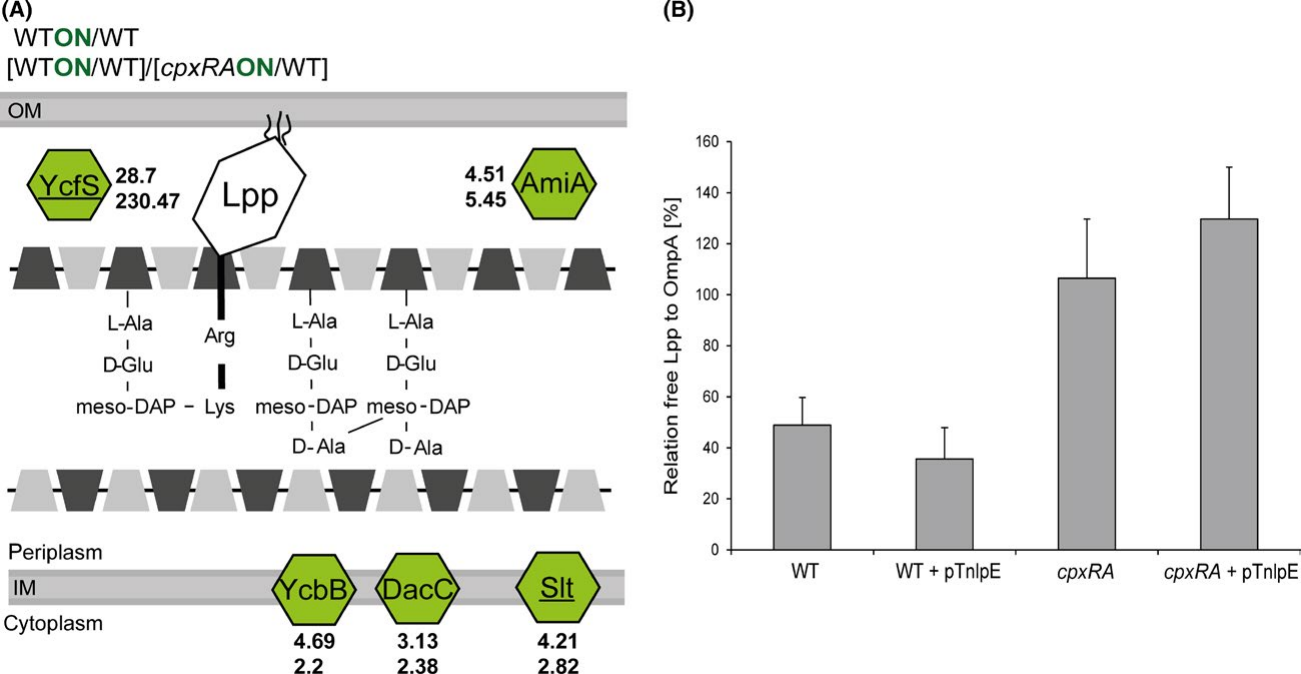
The Cpx system affects cross‐linking between Lpp and peptidoglycan (PG). (A) Activation of the Cpx system leads to increased amounts of PG‐modifying proteins. The PG of *Escherichia coli* can be modified by cross‐linking the Braun's lipoprotein Lpp to PG (YcfS), by intermolecular cross‐linking of PG (YcbB, DacC), by removing intermolecular crosslinks of PG (AmiA), or by cleavage of the MurNAc‐GlcNAc‐backbone (Slt). Color code corresponds to the code in Figure 4. Underlined proteins are known to have a binding site for CpxR~P on DNA level. Shown are the averages of five biological replicates. (B) The impact of the Cpx activation on PG‐cross‐linking was investigated by evaluating the relative free Lpp to OmpA amount in WT cells, after Cpx activation (WT + pT*nlpE*), and also in a *cpxRA* strain using identical conditions (*cpxRA*; cpxRA + pT*nlpE*). Free Lpp and OmpA were detected by immunoblotting and band densities were analyzed by ImageJ. OmpA served as a control for relative cell amounts. The relation of free Lpp to OmpA is depicted in percent (%) and the average and standard deviations of three independent determinations are shown.

We found that not only the levels of PG‐cross‐linking proteins YcbB (4.69) and DacC (3.13), but also those of the PG cleaving proteins AmiA (2.9) and Slt (4.21) were increased upon activation of the Cpx‐TCS (Fig. 5A), suggesting a general function of the Cpx system in PG turnover.

Strongest alterations among all proteins affected in their level upon activation of the Cpx system were found for YcfS (WT_ON_/WT ratio: 28.7, [WT_ON_/WT]/[c*pxRA*_ON_/WT] ratio: 230.47; Fig. 5A, Table S4A). Despite not passing the significance threshold of *q* < 0.05 (YcfS *q*‐value 0.06) the finding was considered to be relevant because YcfS is known to be directly under CpxR control (Yamamoto and Ishihama 2006) and influences the anchoring of Lpp to the PG (Braun and Rehn 1969). Lpp exists in two different forms: free in the periplasm and bound to the PG (Braun and Bosch 1972). Upon increased YcfS abundance, we expected that the level of anchored Lpp should increase and the level of free Lpp should decrease. To investigate this assumption, we analyzed the relative amount of free Lpp in the WT and *cpxRA* strain that were grown either in standard LB or under Cpx‐activating conditions (WT + pT*nlpE*) by immunoblotting. OmpA, which is known to not be affected by the Cpx system, was used as a reference (McEwen and Silverman 1982). Activation of the Cpx system led to slightly decreased levels of free Lpp (Fig. 5B). The connection between the Cpx system and the level of free Lpp was more significant for the *cpxRA* strain. Here, the level of free Lpp was clearly increased and the mechanism behind has to be subject of future studies. Together, our finding highlights cross‐linking between the outer membrane and the PG layer as a new target of the Cpx system.

## Discussion

In order to thoroughly elucidate the function of a cell, it is important to measure the amount of proteins which effect biological processes. In this report, we have determined the absolute amounts of the envelope stress Cpx‐TCS of *E. coli* MG1655 as well as the impact of the Cpx‐TCS on the protein composition under Cpx‐activating conditions.

### Quantification and stoichiometry of CpxA, CpxR, and CpxP in stressed and unstressed cells

Up to now, it was unknown whether the response of the Cpx‐TCS is predominantly modulated by the protein amounts or by the activity of the system. In this respect, it was not known what number of the accessory protein CpxP molecules is needed to inhibit the system.

Until recently, quantitative immunoblotting was the method of choice for analyzing the molecular composition of TCS, as shown for the EnvZ/OmpR‐TCS in *E. coli* (Cai and Inouye 2002) and the WalRKJ‐TCS in *Streptococcus pneumonia* (Wayne et al. 2010). However, due to the extremely low protein levels in unstressed cells, quantitative immunoblotting failed to determine the absolute amount of the accessory protein LiaF of the LiaRSF‐TCS of *Bacillus subtilis* (Schrecke et al. 2013). The relative protein level of LiaF was estimated by analyzing the amount of *liaF* transcript relative to that of *liaS* and *liaR*. Although we had the identical problem to quantify the absolute protein level of CpxP in unstressed cells, we were not allowed of deriving the protein level from the transcript level of *cpxP*. It is well established that *cpxP* expression is not only regulated at the level of gene transcription, but also by the stability of the transcript, the translocation efficiency, and protein stability (Chao and Vogel 2016; Isaac et al. 2005; Miot and Betton 2007). Very recently, SRM was used to quantify the absolute protein level of the KdpD/KdpE‐TCS and the KdpFABC K^+^‐transporter of *E. coli* (Surmann et al. 2014). We have now used SRM to quantify the absolute protein amounts of CpxA, CpxR, and CpxP in *E. coli* cells grown in standard LB, under Cpx‐activating (overexpression of *nlpE*) or Cpx‐inhibiting (overexpression of *cpxP*) growth conditions. To our knowledge, the data presented in this study provide for the first time information on absolute molecule amounts of a TCS and its accessory protein, while avoiding protein tags, or introducing any changes at the genetic or proteome level, or using varying methods.

Our data revealed low absolute levels of CpxA and CpxR in unstressed cells, which increased only twofold during Cpx‐activating or Cpx‐inhibiting conditions. A mild effect on protein level after activation was also observed for the EnvZ/OmpR osmotic stress TCS of *E. coli* (Cai and Inouye 2002). In contrast, the protein levels of the LiaFSR system of *B. subtilis* and the KdpDE‐TCS of *E. coli* increase at least 10‐fold upon stimulation (Schrecke et al. 2013; Surmann et al. 2014). Concurrently, the stoichiometry between sensor kinase and RR remained constant for these four systems. In case of the Cpx‐TCS, the stoichiometry of CpxA to CpxR was robust with ratios of 1:10 or 1:5 for CpxA_2_:CpxR, respectively, under all tested conditions. Hence, the level of the RR CpxR always exceeds the level of the sensor kinase CpxA, which ensures an efficient response. Obviously, a high excess of CpxP over CpxA is needed to provoke an inhibitory effect. Cumulatively, our observations indicate that the Cpx‐TCS response is in parts modulated as a consequence of mild changes in CpxR and CpxA levels, and in particular by its activity.

### Cpx activation has a mild effect on other envelope stress systems, but a strong effect on quality control and acid stress response proteins

The effect of Cpx activation on the whole cell proteome was assessed by a shotgun proteomics approach. To point out that the changes in protein levels were specific for the Cpx system, we not only compared the effect of *nlpE* overexpression on the WT strain (Cpx activation), but also on a *cpxRA* strain. The increased protein levels of several well‐established Cpx targets (DegP, DsbA, PpiA, HtpX) are consistent with transcriptome data collected under identical Cpx‐activating conditions (Raivio et al. 2013) and thereby emphasize the suitability of the presented method to investigate changes on the proteome level.

Remarkably, we observed that the protein levels of only five of 20 envelope stress system proteins were affected by the activation of the Cpx‐TCS. In contrast to this, previous transcriptome data imply a global effect of the Cpx system on other envelope stress systems (Bury‐Mone et al. 2009). Besides using different methods, these differences might result from posttranscriptional or posttranslational modifications. In this respect, we detected for some of the established Cpx target proteins (DegP, Spy, CpxP) significantly stronger alterations than expected from transcriptome data. It has been shown, that the level of CpxP is not only regulated at the level of transcription, but also by at least three different posttranslational modifications: (1) the acetyl coenzyme A‐dependent acetylation of the *α*‐subunit of RNA polymerase (Lima et al. 2012); (2) an inefficient translation initiation (Miot and Betton 2007); and (3) by protein turnover (Isaac et al. 2005; Tschauner et al. 2014). Hence, milder alterations than expected from transcriptome data might be caused by increased protein turnover during Cpx‐activating conditions. Such differences have to be validated, for example, by absolute quantification of proteins over a growth period after Cpx pathway activation, but this was not in scope of this study.

Similarly, we interpret the observation that acid stress proteins displayed significantly reduced levels after Cpx induction compared to the wild type as a result of posttranscriptional or posttranslational effects. To the best of our knowledge, the genes for the acid stress proteins CydA, CydB, GadA, GadC, CadA, HdeA, HdeB, and HdeD were not identified as Cpx targets neither by transcriptome analysis nor by genome‐wide profiling (Pogliano et al. 1997; De Wulf et al. 2002; Bury‐Mone et al. 2009; Raivio et al. 2013).

The acid stress network of *E. coli* can be divided into active and passive mechanisms (Slonczewski et al. 2009). Passive mechanisms depend on the composition of the cytoplasm and its buffering capacity, whereas active acid stress response mechanisms involve proton‐consuming acid resistance systems, as well as metabolic and physiological systems. Proton‐consuming acid resistance systems combine the consumption of intracellular protons by specific amino acid decarboxylases with the export of the respective substrate by a specific antiporter (Foster 2004). *Escherichia coli* possesses four proton‐consuming systems named GDAR (glutamic acid‐dependent acid resistance), ADAR (arginine‐dependent acid resistance), LDAR (lysine decarboxylase acid resistance), and ODAR (ornithine‐dependent acid resistance) (Sabo et al. 1974; Applebaum et al. 1975; Capitani et al. 2003; Andrell et al. 2009). While CydA and CydB were described to provoke metabolic responses to mild acid stress (pH 4–5), GadA, GadC, and CadA belong to the group of proton‐consuming acid resistance systems (GDAR) being active under extreme acidic conditions (pH 2–3). These systems use direct consumption of intracellular protons by a specific amino acid decarboxylase (GadA, CadA) in combination with the respective antiporter (GadC, CadB) in the inner membrane to protect the cells from acid stress (Kanjee et al. 2011).

Metabolic changes to acid stress include the upregulation of several components of the electron transport chain, such as the cytochrome *bo* oxidase (CBO) or NADH dehydrogenase II (NDH‐II), to obtain a higher rate of proton export (Hayes et al. 2006). In this work, we could identify CydA and CydB with reduced abundance after Cpx activation. CydA and CydB represent the high‐affinity cytochrome *d* oxidase in *E. coli*, and the *cydAB* operon is known to be induced under oxygen‐limiting conditions (Rice and Hempfling 1978). In addition to oxygen‐limiting conditions, *cydAB* is also known to be expressed under low pH conditions referring to pH 5.7 (Hayes et al. 2006). Physiological adaptations involve alterations of the membrane composition, lowering the proton influx by affecting outer membrane porins (delaVega and Delcour 1995; Samartzidou et al. 2003), and refolding of misfolded proteins by molecular chaperones. The chaperones identified in this study, HdeA and HdeB, contribute to physiological adaptations to acid stress by binding to acid‐dependent denatured substrate proteins, and releasing them for refolding after returning to neutral pH (Gajiwala and Burley 2000; Kern et al. 2007). Hence, our data not only show a decreased abundance of proton‐consuming proteins, but also highlights decreased levels of proteins that are involved in metabolic and physiological adaptations for the resistance against acid stress.

So far it is only known that all of the acid stress involved proteins identified in this study are inhibited by the global regulator H‐NS (*h*istone‐like *n*ucleoid *s*tructuring protein) (Rowbury 1997; Gajiwala and Burley 2000; Govantes et al. 2000; Giangrossi et al. 2005). Consequently, H‐NS‐deficient strains show increased survival rates at low pH (Atlung and Ingmer 1997; Hommais et al. 2001). In addition, we could successfully demonstrate that the decreased amounts of acid stress proteins correlate with an increased survival rate of a *cpxRA* strain after an extreme pH shock. Since it is well known that the Cpx system is induced by alkaline pH (Danese and Silhavy 1998), our data assign the Cpx system an extended function in pH response by inhibiting acid stress proteins after Cpx activation. However, it remains unclear whether the reduced amount of acid stress response proteins after Cpx activation is due to a direct effect of Cpx targets that act in quality control, or due to an indirect effect on a so far unknown regulatory factor that, for example, enhances the inhibitory effect of H‐NS.

### The Cpx‐TCS regulates processes that modulate cell wall stability

Our proteome data expand the knowledge on the impact of the Cpx‐TCS on PG‐cross‐linking. In addition to the increased levels of some PG‐cross‐linking proteins, our proteome analysis revealed the protein YcfS as the protein with the strongest difference in abundance after induction of the Cpx system with a WT_ON_/WT ratio of 28.7. YcfS functions in anchoring the Braun's lipoprotein Lpp to the PG thereby connecting the PG layer to the outer membrane resulting in increased cell wall stability (Braun and Rehn 1969; Braun and Wolff 1975). Hence, an increase of cross‐linking between Lpp and PG results in a decrease of free Lpp.

Here, we show that the induction of the Cpx system leads to a decrease of free Lpp in the periplasm, indicating an increase of cross‐linking between Lpp and PG, and thus, an increase of cell wall stability. These results perfectly match previous regulatory data demonstrating that the Cpx system monitors cell wall stability and that removal of several PG‐binding proteins induces the Cpx stress response in *E. coli* (Evans et al. 2013). Consistent with this, the Cpx‐TCS regulates the expression of LdtD and YgaU, two proteins involved in PG‐cross‐linking modifications (Bernal‐Cabas et al. 2015). In addition, characterization of the Cpx system in the rumen bacterium *Mannheimia succiniproducens* further unraveled the role of the Cpx system in the maintenance of cell wall integrity. Upon *cpxR* overexpression in *M. succiniproducens*, the majority of upregulated genes were identified to be involved in cell wall and membrane biogenesis (Yun et al. 2015). Thus, opposing to initial expectations, the cumulative data suggest a more important, generally regulating function of the Cpx‐TCS for processes that modulate cell wall stability.

To summarize, we were able to clearly demonstrate the broadly spread functions of the Cpx system affecting different stresses within the cell, and thereby exhibiting its role in the complex network of the cell. Our results allow three very important conclusions concerning the functionality of the Cpx‐TCS. First, the Cpx response is modulated by the activity of the Cpx‐TCS and not by the amount of the sensor kinase CpxA, or the RR CpxR. Second, in contrast to the RseA/Spy system, the Cpx‐TCS impacts other envelope stress systems only to a low extent, whereas the acid stress response is affected to a high extent. Third, the Cpx‐TCS has a general function for cell wall stability.

## Conflict of Interest

None declared.

## Supporting information


**Table S1.** Transition list of targeted proteins for SRM acquisition.
**Table S2.** Results from absolute quantification of CpxA, CpxP, and CpxR by SRM on peptide level under wild‐type (WT) conditions, induction of Cpx (*nlpE* overexpression), and inhibition of CpxRA (by *cpxP* overexpression).
**Table S3.** Proteome profile of *Escherichia coli* and an isogenic cpxRA mutant grown under wild‐type (WT) and Cpx‐inducing (ON, by *nlpE* overexpression) conditions.
**Table S4.** Comparison of transcriptome and proteome data of Cpx‐TCS target proteins.
**Figure S1.** Changes in expression and protein level of acid stress involved genes and proteins after Cpx activation.
**Figure S2.** Relative intensities of NlpE determined by proteome profiling.
