# Supplementary material for: Molecular and proteome analyses highlight the importance of the Cpx envelope stress system for acid stress and cell wall stability in Escherichia coli
Source: Microbiologyopen. 2016 Apr 2;5(4):582–96. doi: 10.1002/mbo3.353 (PMC4985592; doi:10.1002/mbo3.353)
Supplement: Supplementary file 1 — Table S1. Transition list of targeted proteins for SRM acquisition. Table S2. Results from absolute quantification of CpxA, CpxP, and CpxR by SRM on peptide level under wild‐type (WT) conditions, induction of Cpx (nlpE overexpression), and inhibition of CpxRA (by cpxP overexpression). Table S3. Proteome profile of Escherichia coli and an isogenic cpxRA mutant grown under wild‐type (WT) and Cpx‐inducing (ON, by nlpE overexpression) conditions. Table S4. Comparison of transcriptome and proteome data of Cpx‐TCS target proteins. Figure S1. Changes in expression and protein level of acid stress involved genes and proteins after Cpx activation. Figure S2. Relative intensities of NlpE determined by proteome profiling. [file MBO3-5-582-s001.pdf]

Supporting Information

Molecular and proteome analyses highlight the importance of the Cpx envelope stress system for acid stress and cell wall stability in *Escherichia coli*

Kristin Surmann[1]*, Emina Ćudić[2]*, Elke Hammer[1#], Sabine Hunke[2#]

*These authors contributed equally to this work

[#]corresponding author

[1]Department of Functional Genomics, Interfaculty Institute of Genetics and Functional Genomics, University Medicine Greifswald, Friedrich-Ludwig-Jahn-Straße 15A, 17475 Greifswald, Germany

[2]FB 5 Microbiology, Department of Biology/Chemistry, University Osnabrück, Barbarastraße 11, 49076 Osnabrück, Germany

Correspondence:

Prof. Dr. rer. nat. Sabine Hunke

E-mail: Sabine.Hunke@UOS.de

Tel.: +49 541 969-7141

Fax: +49 541 969-3942

Content

Details on data acquisition for shotgun proteomics ……………………………………….……...3
Presentation of protein identification results …………………………………………………………..4
Details on data acquisition for MRM ……………………………………………………………………4
Figure S1 ………………………………………………………………………………………………..5
Figure S2 ………………………………………………………………………………………………..6
Descriptions of supplemental tables S1-S4………………………….……………………….………..7

Details on data acquisition for shotgun proteomics

LC-Parameters	Settings
LC-column	Acclaim PepMap 100 reverse phase column (3 μm, 75 μm i.d x 150 mm, LC Packings, Dionex, Idstein, Germany)
LC-gradient	0 min-1%ACN-35-1-36-5-245-25-305-60-306-99-310-1-320-1
Solvent flow rate	300 nL/min
**MS-Parameters**	**Settings**
Mass range	m/z 300-2,000
Resolution	60.000 at m/z 400
Name of peaklist-generating software and release version (number or date)	ReadW in Sorcerer built 4.04 (SageN Research Inc., Milpitas, CA, USA) with default parameters
Name of the search engine and release version (number or date)	Sequest (v. 2.7) in Sorcerer built 4.04 (SageN)
Enzyme specificity considered	Fully tryptic
# of missed cleavages permitted	Missed cleavages=0
Fixed modification(s) (including residue specificity)	Carbamidomethylation at cysteine
Variable modification(s) (including residue specificity)	Oxidation on methionine
Mass tolerance for precursor ions	10 ppm
Mass tolerance for fragment ions	1 Da
Name of database searched and release version/date	Swiss-Prot database rel. 06_2014 limited to *E. coli* K12 entries
Threshold score/E-value for accepting *individual* MS/MS Spectra	Peptide Teller false positive rate 1%
Software/method used to evaluate site assignment	No PTM reported

Presentation of protein identification results

Information requested	Reported
Accession number	UniprotAccession (Table 3)
Number of *unique* (in terms of amino acid sequence) peptides identified, % sequence coverage identified from MS/MS data or a list of sequences identified	Table S3
Additional information, such as a protein's name, function, MW, pI, score, peptide sequences, etc.	Table S3
Single Peptide Protein IDs and PTMs	Not reported in this manuscript.

Details on data acquisition for MRM

LC-Parameters	Settings
LC gradient	0 min-5%ACN-3-5-26-35-29-45-31-100-33-100-36-0
Solvent flow rate	300 nL/min
**MS-Parameters**	**Settings**
Resolution	MS1 R=0.7 full width at half maximum (FWHM), MS2 R= 2.5 FWHM
Dwell time	20 ms per transition
Cycle time	2.4 s/cycle

MRM transitions are provided in Supporting Information Table 1. Results from absolute quantification are presented in Figure 1 and Table S2.

Figure S1

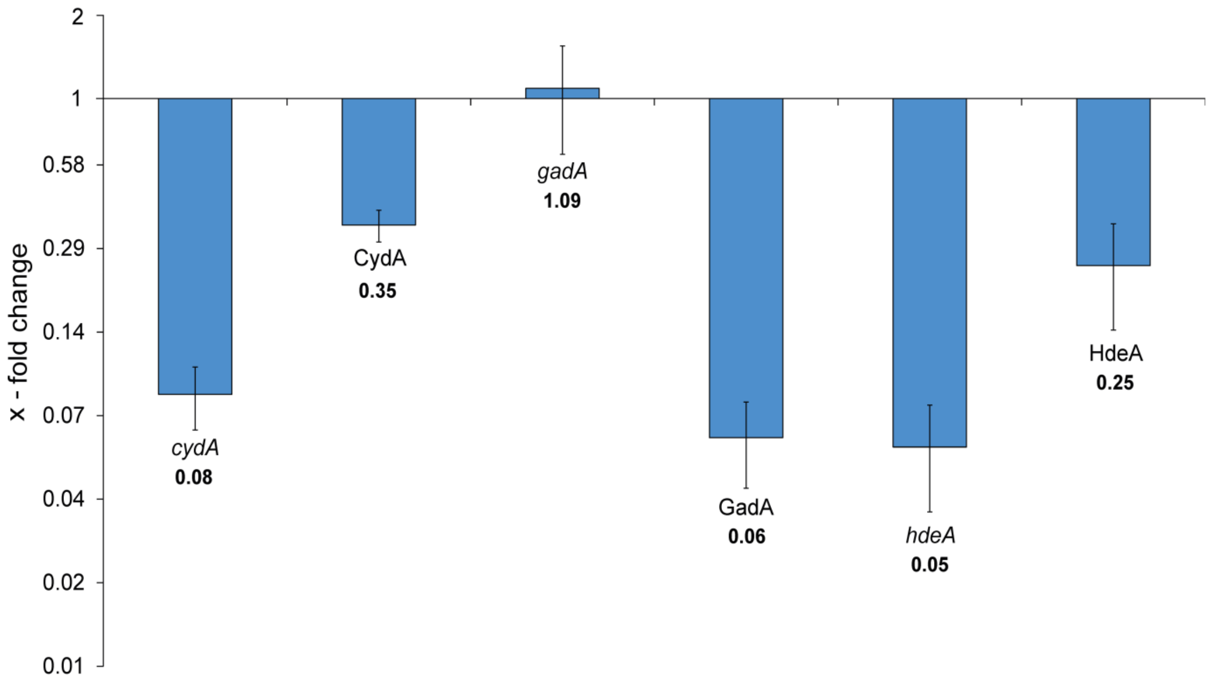

Figure S1: Changes in expression- and protein level of acid stress involved genes and proteins after Cpx-activation. Changes in expression levels for the genes *cydA*, *gadA* and *hdeA* were determined by q-RT-PCR. All depicted values represent changes between WT_{ON}/WT and represent the mean data and standard deviations of five biological replicates. Ratios of protein intensities WT_{ON}/WT are presented as well for CydA, GadA, and HdeA with mean values and standard deviations of four biological replicates. The y-axis is formatted in log_2 logarithmic scale.

Figure S2

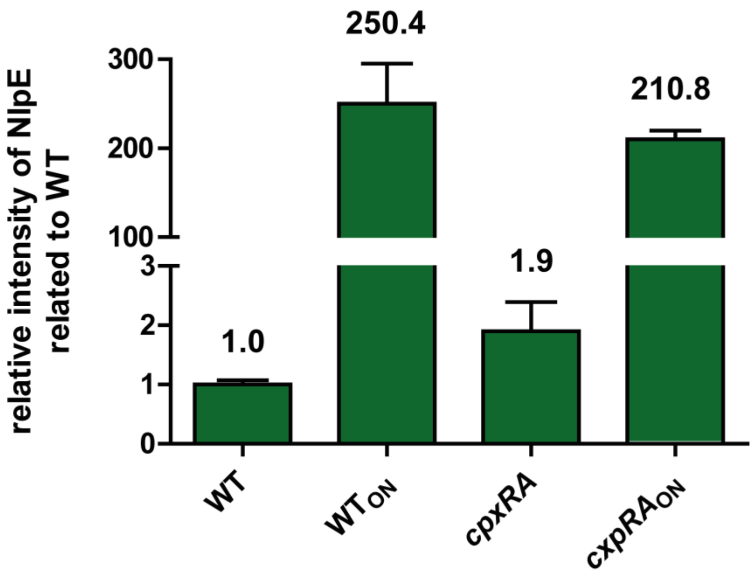

Figure S2: Relative intensities of NlpE determined by proteome profiling. Since the induction of the Cpx-TCS was induced by *nlpE* overexpression, we aimed to control NlpE production by relative proteome analysis. Average values and standard deviations of normalized intensities from four independent biological replicates are displayed.

Descriptions of supplemental tables.

Tables are provided as separate files.

Table S1: Transition list of targeted proteins for SRM acquisition. Proteins, proteotypic peptides, m/z ratios of heavy and light precursors and products are given with the optimized collision energy on protein levels. Peptides that were found also by shotgun MS analysis are labelled in the last column with “yes”, peptides which were derived from theoretical digestion with “no”. Further, the R^2 values of the standard curve (two technical replicates, representing the linear range for absolute quantification) for each heavy standard peptide are given. For each protein, the peptide with the highest R^2 value was labelled in bold letters and used for further quantification of protein level.

Table S2: Results from absolute quantification of CpxA, CpxP, and CpxR by SRM on peptide level under wild-type (WT) conditions, induction of Cpx (n*lpE* -overexpression), and inhibition of CpxRA (by *cpxP* -overexpression). 0.5 or 10 fmol μg^{-1} protein of each standard peptide were added to each sample. Peptides chosen for protein quantification due to an optimal R^2 are highlighted in bold letters. According to a spike-in peptide to natural peptide ratio closer to 1, the concentration of the sample peptide in fmol μg^{-1} protein used for final protein quantification is highlighted in green and the corresponding value in the column molecule per cell is highlighted in orange. Final protein concentrations were calculated as an average concentration (conc.) from five independent biological replicates together with their standard deviation (SD) and coefficient of variance (CV).

Table S3: Proteome profile of *E. coli* and an isogenic *cpxRA* mutant grown under wild-type (WT) and Cpx-inducing (ON, by *nlpE* -overexpression) conditions. Table S3 is subdivided in the following three tables: Table S3A describes the proteome profiling of WT_{ON} compared to WT, S3B the proteome profiling of *cpxRA* compared to WT, and S3C the proteome profiling of $cpxRA_{ON}$ compared to WT. For all four conditions (WT, WT_{ON}, *cpxRA*, $cpxRA_{ON}$) geometric means of median-normalized protein intensities (normalization performed with Genedata Analyst 8.2 (Genedata, Basel, Switzerland) from four independent biological replicates are displayed for all detected proteins, which were identified with at least two peptides, or when the sequence coverage exceeded 10%, only one peptide was identified, respectively. Further, ratios relative to the WT, p-values from a student’s t-test, and multiple testing-corrected q-values according to Benjamini-Hochberg (BH) were determined using Genedata Analyst.

Proteins whose intensities resulted in q-values <0.05 (highlighted green) and exceeded an absolute fold change of 2 were regarded as significantly different in the compared conditions. Ratios >2 were highlighted in red, those <0.5 were highlighted in blue. Also the coefficient of variance (CV) between the four replicates for each condition was determined.

Table S4: Comparison of transcriptome and proteome data of Cpx-TCS target proteins. All proteins being Cpx-dependently/-independently induced (**S4A**) or inhibited (**S4B**) are listed in comparison with the WT_{ON}/WT-ratios of transcriptome data from Raivio *et al.*, 2013. Furthermore, information on known CpxR~P binding motifs is provided. For each protein, the following ratios were calculated using the protein intensities measured in this study: **WT_{ON}/WT** (compares protein abundance between induced and non-induced WT); ***cpxRA*/WT** (compares protein abundance between non-induced *cpxRA*-strain and non-induced WT); **c*pxRA*$_{ON}$/WT** (compares protein abundance between induced *cpxRA*-strain and non-induced WT). To emphasize the higher amount (Cpx-specific induction) of proteins in induced WT-cells compared to induced *cpxRA*-cells we additionally calculated the **[WT_{ON}/WT / c*pxRA*$_{ON}$/WT]**-ratio (S4A). To highlight the higher amount (Cpx-specific inhibition) of proteins in induced *cpxRA*-cells compared to induced WT-cells we additionally calculated the **[c*pxRA*$_{ON}$/WT / WT_{ON}/WT]**-ratio (S4B). For each ratio minimum 2-fold difference is defined as significant. Calculating the difference between induced and non-induced *cpxRA*-cells, we checked whether *nlpE*-overexpression has an additional effect on the relative amounts of proteins. Minimum 2-fold difference (+) or (-) was defined as an additional NlpE-effect (S4A,B).
