# Supplementary material for: Molecular and proteome analyses highlight the importance of the Cpx envelope stress system for acid stress and cell wall stability in Escherichia coli
Source: Microbiologyopen. 2016 Apr 2;5(4):582–96. doi: 10.1002/mbo3.353 (PMC4985592; doi:10.1002/mbo3.353)
Supplement: Supplementary file 2 [file MBO3-5-582-s002.pdf]

Table S1: Transition list of targeted proteins for SRM acquisition.

Proteins, proteotypic peptides, m/z ratios of heavy and light precursors and products are given with the optimized collision energy on protein levels. Peptides that were found also by shotgun MS analysis are labelled in the last column with “yes”, peptides which were derived from theoretical digestion with “no”. Further, the R^2 values of the standard curve (two technical replicates, representing the linear range for absolute quantification) for each heavy standard peptide are given. For each protein, the peptide with the highest R^2 value was labelled in bold letters and used for further quantification of protein level.

protein	peptide	precursor m/z	product m/z	optimized collision energy	heavy standard	detected in shotgun MS	R^2 value for standard curve of heavy peptide	minimum detectable concentration [fmol/µg protein]
CpxA	**AEDSPLGGLR**	**507,764**	**402,246**	**19**	**no**	**no**	**0,9994**	0,5
		**507,764**	**612,383**	**19**	**no**	**no**		
		**507,764**	**699,415**	**19**	**no**	**no**		
		**507,764**	**814,442**	**19**	**no**	**no**		
		**512,768**	**412,254**	**19**	**yes**	**no**		
		**512,768**	**622,391**	**19**	**yes**	**no**		
		**512,768**	**709,423**	**19**	**yes**	**no**		
		**512,768**	**824,45**	**19**	**yes**	**no**		
	LLLVTTEGR	501,303	462,231	19	no	yes	0,9987	0,1
		501,303	563,278	19	no	yes		
		501,303	662,347	19	no	yes		
		501,303	775,431	19	no	yes		
		506,307	472,239	19	yes	yes		
		506,307	573,287	19	yes	yes		
		506,307	672,355	19	yes	yes		
		506,307	785,439	19	yes	yes		
	**DVTQWQK**	**452,73**	**461,251**	**18**	**no**	**no**	**0,9998**	0,5
		**452,73**	**589,309**	**18**	**no**	**no**		
		**452,73**	**690,357**	**18**	**no**	**no**		
		**452,73**	**789,425**	**18**	**no**	**no**		
		**456,737**	**469,265**	**18**	**yes**	**no**		
		**456,737**	**597,323**	**18**	**yes**	**no**		
		**456,737**	**698,371**	**18**	**yes**	**no**		
		**456,737**	**797,44**	**18**	**yes**	**no**		

CpxP	LLTPEQQAVLNEK	741,912	503,282	22	no	yes	0,9997	0,1
		741,912	673,388	22	no	yes		
		741,912	1155,6	22	no	yes		
		741,912	1256,648	22	no	yes		
		745,919	511,297	22	yes	yes		
		745,919	681,402	22	yes	yes		
		745,919	1163,615	22	yes	yes		
		745,919	1264,662	22	yes	yes		
	LVTAENFDENAVR	739,368	850,405	22	no	yes	0,9997	0,1
		739,368	964,448	22	no	yes		
		739,368	1093,491	22	no	yes		
		739,368	1265,576	22	no	yes		
		744,372	860,414	22	yes	yes		
		744,372	974,457	22	yes	yes		
		744,372	1103,499	22	yes	yes		
		744,372	1275,584	22	yes	yes		
CpxR	**EHLSQEVLGK**	**570,306**	**545,329**	**22**	**no**	**yes**	**0,9997**	0,5
		**570,306**	**673,388**	**22**	**no**	**yes**		
		**570,306**	**760,42**	**22**	**no**	**yes**		
		**570,306**	**873,504**	**22**	**no**	**yes**		
		**574,313**	**553,344**	**22**	**yes**	**yes**		
		**574,313**	**681,402**	**22**	**yes**	**yes**		
		**574,313**	**768,434**	**22**	**yes**	**yes**		
		**574,313**	**881,518**	**22**	**yes**	**yes**		
	GSELDR	676,326	403,23	26	no	no	not applicable	not applicable
		676,326	502,214	26	no	no		
		676,326	532,273	26	no	no		
		676,326	619,305	26	no	no		
		686,334	413,238	26	yes	no		
		686,334	502,214	26	yes	no		
		686,334	542,281	26	yes	no		
		686,334	629,313	26	yes	no		
