# Supplementary material for: Molecular and proteome analyses highlight the importance of the Cpx envelope stress system for acid stress and cell wall stability in Escherichia coli
Source: Microbiologyopen. 2016 Apr 2;5(4):582–96. doi: 10.1002/mbo3.353 (PMC4985592; doi:10.1002/mbo3.353)
Supplement: Supplementary file 3 [file MBO3-5-582-s003.pdf]

Table S2: Results from absolute quantification of CpxA, CpxP, and CpxR by SRM on peptide level under wild-type (WT) conditions, induction of Cpx (*nlpE* -overexpression), and inhibition of CpxRA (by *cpxP* -overexpression).

0.5 or 10 fmol µg^{-1} protein of each standard peptide were added to each sample. Peptides chosen for protein quantification due to an optimal R^2 are highlighted in bold letters. According to a spike-in peptide to natural peptide ratio closer to 1, the concentration of the sample peptide in fmol µg^{-1} protein used for final protein quantification is highlighted in green and the corresponding value in the column molecule per cell is highlighted in orange. Final protein concentrations were calculated as an average concentration (conc.) from five independent biological replicates together with their standard deviation (SD) and coefficient of variance (CV).

			protein conc. [µg/cell]			wild type														
						spike in 0.5 fmol/µg							spike in 10 fmol/µg							
protein	peptide	replicate	wild type	*cpxP* over-expression	*nlpE* over-expression	ratio 'light'/ 'heavy'	conc. sample protein [fmol/µg]	average conc. sample protein [fmol/µg]	SD conc. sample protein [fmol/µg]	CV conc. sample protein [%]	conc. sample protein [molecule/cell]	average conc. sample protein [molecule/cell]	ratio 'light'/ 'heavy'	conc. sample protein [fmol/µg]	average conc. sample protein [fmol/µg]	SD conc. sample protein [fmol/µg]	CV conc. sample protein [%]	conc. sample protein [molecule/cell]	average conc. sample protein [molecule/cell]	average conc. sample protein [fmol/µg]
CpxA	LLLVTTEGR	BR1	1,5E-07	1,6E-07	1,3E-07	0,88	0,44	0,55	0,08	14,74	41	48	0,04	0,44	0,55	0,10	18,60	40,63	45,52	0,53
		BR2	1,5E-07	1,3E-07	1,1E-07	1,18	0,59				54		0,06	0,63				58,28		
		BR3	1,4E-07	1,7E-07	1,2E-07	1,26	0,63				54		0,06	0,58				49,28		
		BR4	1,3E-07	1,8E-07	1,2E-07	1,11	0,56				43		0,06	0,65				49,76		
		BR5	1,1E-07	1,3E-07	1,3E-07	na	na				na		0,04	0,44				29,65		
	**AEDSPLGGLR**	**BR1**	**1,5E-07**	**1,6E-07**	**1,3E-07**	**0,69**	**0,34**	**0,49**	**0,15**	**29,62**	**32**	**42**	**0,04**	**0,43**	**0,49**	**0,07**	**14,41**	**39,07**	**40,46**	**0,49**
		**BR2**	**1,5E-07**	**1,3E-07**	**1,1E-07**	**0,98**	**0,49**				**45**		**0,05**	**0,46**				**41,89**		
		**BR3**	**1,4E-07**	**1,7E-07**	**1,2E-07**	**1,38**	**0,69**				**59**		**0,05**	**0,48**				**40,87**		
		**BR4**	**1,3E-07**	**1,8E-07**	**1,2E-07**	**0,89**	**0,44**				**34**		**0,06**	**0,61**				**47,14**		
		**BR5**	**1,1E-07**	**1,3E-07**	**1,3E-07**	**na**	**na**				**na**		**0,05**	**0,49**				**33,33**		
CpxP	LVTAENFDENAVR	BR1	1,5E-07	1,6E-07	1,3E-07	0,55	0,27	0,35	0,11	30,85	25	30	0,04	0,37	0,42	0,04	10,66	34,11	34,48	0,37
		BR2	1,5E-07	1,3E-07	1,1E-07	0,52	0,26				24		0,04	0,38				34,53		
		BR3	1,4E-07	1,7E-07	1,2E-07	0,98	0,49				42		0,04	0,43				36,79		
		BR4	1,3E-07	1,8E-07	1,2E-07	0,77	0,39				30		0,05	0,47				36,03		
		BR5	1,1E-07	1,3E-07	1,3E-07	na	na				na		0,05	0,46				30,95		
	LLTPEQQAVLNEK	BR1	1,5E-07	1,6E-07	1,3E-07	1,01	0,50	0,57	0,12	21,47	46	50	0,07	0,66	0,58	0,11	19,12	61,04	48,38	0,54
		BR2	1,5E-07	1,3E-07	1,1E-07	1,50	0,75				69		0,05	0,49				44,84		
		BR3	1,4E-07	1,7E-07	1,2E-07	1,01	0,51				43		0,06	0,63				53,70		
		BR4	1,3E-07	1,8E-07	1,2E-07	1,03	0,51				40		0,07	0,68				52,62		
		BR5	1,1E-07	1,3E-07	1,3E-07	na	na				na		0,04	0,44				29,72		
	**DVTQWQK**	**BR1**	**1,5E-07**	**1,6E-07**	**1,3E-07**	**0,66**	**0,33**	**0,42**	**0,16**	**37,20**	**30**	**36**	**0,03**	**0,32**	**0,37**	**0,18**	**48,58**	**29,60**	**30,23**	**0,42**
		**BR2**	**1,5E-07**	**1,3E-07**	**1,1E-07**	**1,03**	**0,52**				**48**		**0,03**	**0,30**				**27,90**		
		**BR3**	**1,4E-07**	**1,7E-07**	**1,2E-07**	**1,23**	**0,62**				**52**		**0,03**	**0,25**				**21,33**		
		**BR4**	**1,3E-07**	**1,8E-07**	**1,2E-07**	**0,87**	**0,44**				**34**		**0,07**	**0,69**				**53,00**		
		**BR5**	**1,1E-07**	**1,3E-07**	**1,3E-07**	**0,43**	**0,21**				**15**		**0,03**	**0,28**				**19,32**		
CpxR	**EHLSQEVLGK**	**BR1**	**1,5E-07**	**1,6E-07**	**1,3E-07**	**7,10**	**3,55**	**5,29**	**1,91**	**36,15**	**327**	**458**	**0,45**	**4,53**	**4,70**	**0,83**	**17,74**	**416,71**	**392,63**	**4,70**
		**BR2**	**1,5E-07**	**1,3E-07**	**1,1E-07**	**11,82**	**5,91**				**544**		**0,49**	**4,90**				**451,15**		
		**BR3**	**1,4E-07**	**1,7E-07**	**1,2E-07**	**15,42**	**7,71**				**655**		**0,52**	**5,24**				**444,95**		
		**BR4**	**1,3E-07**	**1,8E-07**	**1,2E-07**	**7,97**	**3,98**				**307**		**0,55**	**5,48**				**422,49**		
		**BR5**	**1,1E-07**	**1,3E-07**	**1,3E-07**	**na**	**na**				**na**		**0,34**	**3,35**				**227,84**		

6,02E+08 molecules/fmol

cpxP overexpression

final quantification			spike in 0.5 fmol/µg							spike in 10 fmol/µg							final quantification				
**SD conc. sample protein [fmol/µg]**	**CV conc. sample protein [%]**	**average conc. sample protein [molecule/cell]**	ratio 'light'/ 'heavy'	conc. sample protein [fmol/µg]	average conc. sample protein [fmol/µg]	SD conc. sample protein [fmol/µg]	CV conc. sample protein [%]	conc. sample protein [molecule/cell]	average conc. sample protein [molecule/cell]	ratio 'light'/ 'heavy'	conc. sample protein [fmol/µg]	average conc. sample protein [fmol/µg]	SD conc. sample protein [fmol/µg]	CV conc. sample protein [%]	conc. sample protein [molecule/cell]	average conc. sample protein [molecule/cell]	**average conc. sample protein [fmol/µg]**	**SD conc. sample protein [fmol/µg]**	**CV conc. sample protein [%]**	**average conc. sample protein [molecule/cell]**	ratio 'light'/ 'heavy'
			0,56	0,28				27		0,04	0,37				35						2,74
			0,90	0,45				35		0,04	0,44				34						3,09
0,09	16,65	44	0,66	0,33	0,36	0,07	20,10	34	34	0,03	0,33	0,40	0,06	14,23	34	36	0,38	0,08	20,51	34	3,72
			0,75	0,38				40		0,04	0,38				40						2,98
			na	na				na		0,05	0,46				35						3,73
			**0,51**	**0,26**				**24**		**0,03**	**0,29**				**28**						**1,91**
			**0,85**	**0,43**				**33**		**0,04**	**0,36**				**28**						**2,20**
**0,13**	**25,67**	**41**	**0,49**	**0,24**	**0,30**	**0,09**	**29,00**	**25**	**28**	**0,03**	**0,29**	**0,32**	**0,05**	**15,70**	**30**	**29**	**0,32**	**0,09**	**27,39**	**28**	**2,17**
			**0,52**	**0,26**				**28**		**0,03**	**0,28**				**30**						**2,26**
			**na**	**na**				**na**		**0,04**	**0,40**				**30**						**2,00**
			140,14	70,07				6651		8,12	81,20				7707						3,57
			153,16	76,58				5917		8,37	83,74				6470						3,47
0,10	28,06	30	140,67	70,33	73,01	3,31	4,53	7312	6984	8,32	83,22	87,18	17,72	20,32	8652	7875	87,18	17,72	20,32	7875	4,47
			150,12	75,06				8055		7,04	70,39				7554						3,66
			na	na				na		11,73	117,35				8992						11,28
			138,82	69,41				6588		8,11	81,10				7697						4,07
			171,95	85,97				6643		9,04	90,37				6983						4,48
0,12	22,31	46	126,97	63,49	69,28	12,03	17,36	6600	6521	8,96	89,63	91,52	16,65	18,19	9318	8284	91,52	16,65	18,19	8284	6,23
			116,50	58,25				6251		7,70	76,99				8262						4,12
			na	na				na		11,95	119,53				9160						8,34
			**96,17**	**48,08**				**4564**		**7,64**	**76,40**				**7252**						**3,60**
			**162,09**	**81,04**				**6262**		**7,99**	**79,90**				**6173**						**4,77**
**0,16**	**37,20**	**36**	**141,30**	**70,65**	**49,17**	**31,24**	**63,53**	**7345**	**4623**	**7,66**	**76,65**	**81,85**	**15,31**	**18,71**	**7968**	**7399**	**81,85**	**15,31**	**18,71**	**7399**	**3,06**
			**92,14**	**46,07**				**4944**		**6,82**	**68,18**				**7317**						**2,62**
			**0,04**	**0,02**				**1**		**10,81**	**108,13**				**8286**						**na**
			**4,38**	**2,19**				**208**		**0,22**	**2,22**				**211**						**24,65**
			**5,49**	**2,75**				**212**		**0,28**	**2,83**				**219**						**25,19**
**0,83**	**17,74**	**393**	**3,95**	**1,97**	**2,42**	**0,40**	**16,56**	**205**	**231**	**0,28**	**2,79**	**2,80**	**0,49**	**17,34**	**290**	**254**	**2,79**	**0,49**	**17,72**	**253**	**17,48**
			**5,54**	**2,77**				**297**		**0,26**	**2,60**				**278**						**21,72**
			**na**	**na**				**na**		**0,36**	**3,55**				**272**						**17,78**

nlpE overexpression

spike in 0.5 fmol/µg						spike in 10 fmol/µg							final quantification			
conc. sample protein [fmol/µg]	average conc. sample protein [fmol/µg]	SD conc. sample protein [fmol/µg]	CV conc. sample protein [%]	conc. sample protein [molecule/cell]	average conc. sample protein [molecule/cell]	ratio 'light'/ 'heavy'	conc. sample protein [fmol/µg]	average conc. sample protein [fmol/µg]	SD conc. sample protein [fmol/µg]	CV conc. sample protein [%]	conc. sample protein [molecule/cell]	average conc. sample protein [molecule/cell]	average conc. sample protein [fmol/µg]	SD conc. sample protein [fmol/µg]	CV conc. sample protein [%]	average conc. sample protein [molecule/cell]
1,37				108		0,12	1,15				90					
1,54				103		0,12	1,22				82					
1,86	1,63	0,22	13,83	133	120	0,12	1,20	1,20	0,06	4,80	86	88	1,63	0,22	13,83	120
1,49				111		0,11	1,13				84					
1,86				146		0,13	1,28				100					
**0,96**				**75**		**0,10**	**0,96**				**75**					
**1,10**				**74**		**0,08**	**0,84**				**56**					
**1,08**	**1,05**	**0,07**	**7,01**	**77**	**78**	**0,09**	**0,93**	**0,90**	**0,05**	**5,83**	**67**	**67**	**1,05**	**0,07**	**7,01**	**78**
**1,13**				**84**		**0,09**	**0,93**				**69**					
**1,00**				**78**		**0,09**	**0,86**				**67**					
1,78				140		0,15	1,46				115					
1,74				116		0,15	1,53				102					
2,24	2,65	1,69	63,77	160	199	0,19	1,89	1,70	0,22	13,14	135	126	1,91	0,20	10,59	141
1,83				136		0,17	1,65				123					
5,64				441		0,20	1,98				155					
2,04				160		0,20	2,03				160					
2,24				150		0,19	1,94				130					
3,11	2,72	0,92	33,81	222	202	0,24	2,37	2,11	0,21	10,14	169	156	2,20	0,15	7	162
2,06				153		0,19	1,91				142					
4,17				326		0,23	2,31				180					
**1,80**				**141**		**0,15**	**1,45**				**114**					
**2,39**				**160**		**0,15**	**1,48**				**99**					
**1,53**	**1,76**	**0,47**	**26,51**	**109**	**127**	**0,19**	**1,91**	**1,69**	**0,25**	**14,52**	**136**	**125**	**1,80**	**0,42**	**23,10**	**133**
**1,31**				**97**		**0,16**	**1,64**				**122**					
**na**				**na**		**0,20**	**1,99**				**155**					
**12,32**				**968**		**1,00**	**10,03**				**788**					
**12,59**				**843**		**0,95**	**9,45**				**633**					
**8,74**	**10,68**	**1,83**	**17,11**	**624**	**787**	**0,99**	**9,87**	**9,98**	**0,49**	**4,87**	**705**	**738**	**9,98**	**0,49**	**4,87**	**738**
**10,86**				**807**		**1,08**	**10,76**				**799**					
**8,89**				**695**		**0,98**	**9,77**				**763**					
