# Supplementary material for: Molecular and proteome analyses highlight the importance of the Cpx envelope stress system for acid stress and cell wall stability in Escherichia coli
Source: Microbiologyopen. 2016 Apr 2;5(4):582–96. doi: 10.1002/mbo3.353 (PMC4985592; doi:10.1002/mbo3.353)
Supplement: Supplementary file 4 [file MBO3-5-582-s004.pdf]

Table S3A: Proteome profile of *E. coli* and an isogenic *cpxRA* mutant grown under wild-type (WT) and Cpx-inducing (ON, by *nlpE* -overexpression) conditions.

Table S3 is subdivided in the following three tables: Table S3A describes the proteome profiling of WT_{ON} compared to WT, S3B the proteome profiling of *cpxRA* compared to WT, and S3C the proteome profiling of *cpxRA* $_{ON}$ compared to WT. For all four conditions (WT, WT_{ON}, *cpxRA* , *cpxRA* $_{ON}$) geometric means of median-normalized protein intensities (normalization performed with Genedata Analyst 8.2 (Genedata, Basel, Switzerland) from four independent biological replicates are displayed for all detected proteins, which were identified with at least two peptides, or when the sequence coverage exceeded 10%, only one peptide was identified, respectively. Further, ratios relative to the WT, p-values from a student's t-test, and multiple testing-corrected q-values according to Benjamini-Hochberg (BH) were determined using Genedata Analyst. Proteins whose intensities resulted in q-values <0.05 (highlighted green) and exceeded an absolute fold change of 2 were regarded as significantly different in the compared conditions. Ratios >2 were highlighted in red, those <0.5 were highlighted in blue. Also the coefficient of variance (CV) between the four replicates for each condition was determined.

Proteins whose intensit	gene name	description	protein coverage [%]	MW [Da]	pI	number of peptides	category according to Figure 2	geometric mean median normalized intensity WT	geometric mean median normalized intensity WT ON	CV [%] normalized intensity WT	[CV%] median normalized intensity WT ON	ratio WT ON/WT	WT ON/WT p-value	WT ON/WT q-value (BH)
ACCA_ECODH	*accA*	Acetyl-coenzyme A carboxylase carboxyl transfe	40,752	35241,539	5,762	9		3006460	3124905	7,3	5,4	1,04	0,43	0,52
B1XHM4_ECODH	*accB*	Acetyl CoA carboxylase, BCCP subunit	23,718	16687,211	4,658	3		3198997	5266426	14,9	14,8	1,65	0,00	0,01
B1XHM5_ECODH	*accC*	Acetyl-CoA carboxylase, biotin carboxylase subu	37,194	49320,738	6,655	14		7467437	8085920	7,1	7,9	1,08	0,19	0,27
ACCD_ECODH	*accD*	Acetyl-coenzyme A carboxylase carboxyl transfe	20,066	33321,891	7,578	5		2347406	2288056	12,1	4,0	0,97	0,71	0,77
B1XC14_ECODH	*aceA*	Isocitrate lyase	62,903	47521,566	5,161	17	metabolism	29467616	58975884	8,4	6,5	2,00	0,00	0,00
B1XC13_ECODH	*aceB*	Malate synthase	37,899	60273,574	5,386	14		5541986	8912210	9,1	7,2	1,61	0,00	0,00
B1XC89_ECODH	*aceE*	Pyruvate dehydrogenase E1 component	50,169	99668,484	5,457	33		83823832	140943449	4,4	5,4	1,68	0,00	0,00
B1XC90_ECODH	*aceF*	Pyruvate dehydrogenase, dihydrolipoyltransacet	55,873	66096,07	5,09	22		43025454	67138634	6,2	4,7	1,56	0,00	0,00
B1X907_ECODH	*ackA*	Acetate kinase	53,25	43290,453	5,846	12		16194575	27569779	8,8	20,0	1,70	0,00	0,01
B1XBM7_ECODH	*acnA*	Aconitate hydratase 1	32,66	97647,195	5,593	18		3515428	3226102	2,5	8,7	0,92	0,10	0,17
B1XC93_ECODH	*acnB*	Aconitate hydratase 2	46,59	93498,109	5,239	28		21519467	25419686	5,9	2,5	1,18	0,00	0,01
ACP_ECODH	*acpP*	Acyl carrier protein	32,051	8639,52	3,976	2		14209819	11037333	17,6	12,4	0,78	0,05	0,09
B1XFQ0_ECODH	*acrA*	Multidrug efflux system	35,264	42196,707	7,69	9		2290160	2647852	7,8	8,6	1,16	0,05	0,09
B1XFP9_ECODH	*acrB*	Multidrug efflux system protein	24,023	113573,65	5,387	17		3259625	3861231	13,6	7,2	1,18	0,07	0,12
B1XCV5_ECODH	*acs*	Acetyl-coenzyme A synthetase	27,454	72093,586	5,501	12		1177069	2141536	3,4	9,9	1,82	0,00	0,00
ADD_ECODH	*add*	Adenosine deaminase	46,246	36397,461	5,363	10		2001165	4325871	4,3	25,3	2,16	0,00	0,00
ADEC_ECODH	*ade*	Adenine deaminase	7,993	63739,359	5,234	3		218890	141630	12,4	13,1	0,65	0,00	0,01
B1XAT1_ECODH	*adhE*	Fused acetaldehyde-CoA dehydrogenase and iro	45,342	96127,242	6,324	29		24277271	15620244	4,9	4,0	0,64	0,00	0,00
KAD_ECODH	*adk*	Adenylate kinase	39,252	23586,016	5,551	7		8848699	5582263	11,3	16,2	0,63	0,00	0,01
B1X9C3_ECODH	*agp*	Glucose-1-phosphatase/inositol phosphatase	23,729	45682,91	5,484	6		1662520	846769	9,6	4,5	0,51	0,00	0,00
B1X603_ECODH	*ahpC*	Alkyl hydroperoxide reductase, C22 subunit	52,941	20761,443	5,027	7		11698272	31344281	34,3	33,2	2,68	0,01	0,02
B1X604_ECODH	*ahpF*	Alkyl hydroperoxide reductase, F52a subunit, FA	30,518	56177,109	5,469	12		3126874	4368594	3,9	7,9	1,40	0,00	0,00
SYA_ECODH	*alaS*	Alanine--tRNA ligase	43,95	96032,398	5,533	29		11421869	15841560	6,4	9,0	1,39	0,00	0,00
B1XDC8_ECODH	*aldA*	Aldehyde dehydrogenase A, NAD-linked	48,434	52272,789	5,067	16		9433873	10004600	6,4	2,1	1,06	0,14	0,21
B1X8K4_ECODH	*aldB*	Aldehyde dehydrogenase B	15,039	56306,352	5,439	5		468445	371444	8,4	42,7	0,79	0,29	0,39
B1XGA4_ECODH	*allR*	DNA-binding transcriptional repressor	15,498	29269,666	5,773	3		154760	176120	23,7	25,3	1,14	0,47	0,56
B1XAA6_ECODH	*amiA*	N-acetylmuramoyl-l-alanine amidase I	11,419	31412,129	10,1	2	ɔtidoglycan-modificat	68826	310510	9,2	19,1	4,51	0,00	0,00
B1X6C7_ECODH	*amn*	AMP nucleosidase	9,711	53994,914	5,897	3		217383	302279	18,9	7,5	1,39	0,02	0,04
ANMK_ECODH	*anmK*	Anhydro-N-acetylmuramic acid kinase	8,401	39496,277	5,699	2		102438	91918	7,5	10,8	0,90	0,15	0,23
B1XFB7_ECODH	*ansB*	Periplasmic L-asparaginase II	32,759	36850,691	5,956	8		4701317	900112	7,5	8,8	0,19	0,00	0,00
APT_ECODH	*apt*	Adenine phosphoribosyltransferase	43,169	19858,885	5,258	5		2344898	2222689	4,9	18,6	0,95	0,58	0,65
B1XFL1_ECODH	*arcA*	DNA-binding response regulator in two-compone	30,672	27292,016	5,212	5		1831233	1242852	14,6	3,5	0,68	0,00	0,01
B1XHI5_ECODH	*arcB*	Hybrid sensory histidine kinase in two-componer	3,985	87982,906	4,986	2		89952	80730	9,4	22,6	0,90	0,41	0,50
ARGE_ECODH	*argE*	Acetylornithine deacetylase	15,927	42347,344	5,538	4		192832	335388	2,2	6,9	1,74	0,00	0,00
ASSY_ECODH	*argG*	Argininosuccinate synthase	15,884	49898,398	5,23	4		231952	229710	10,0	19,4	0,99	0,93	0,94
ARLY_ECODH	*argH*	Argininosuccinate lyase	8,753	50318,223	5,108	2		68060	133062	14,0	13,4	1,96	0,00	0,00
ARGR_ECODH	*argR*	Arginine repressor	13,462	16994,521	4,928	1		179581	193392	23,2	22,1	1,08	0,64	0,71
SYR_ECODH	*argS*	Arginine--tRNA ligase	34,662	64682,961	5,321	16		4001860	3637407	6,0	3,9	0,91	0,04	0,07
AROA_ECODH	*aroA*	3-phosphoshikimate 1-carboxyvinyltransferase	24,122	46095,781	5,366	8		439700	478119	6,3	14,9	1,09	0,32	0,42
AROB_ECODH	*aroB*	3-dehydroquinate synthase	25,967	38880,988	5,721	6		560425	681275	6,7	6,6	1,22	0,01	0,02
AROC_ECODH	*aroC*	Chorismate synthase	5,54	39137,445	5,81	2		62898	92969	17,7	21,5	1,48	0,03	0,06
AROD_ECODH	*aroD*	3-dehydroquinate dehydratase	14,683	27466,654	5,19	3		77831	83949	12,8	32,9	1,08	0,69	0,75
AROE_ECODH	*aroE*	Shikimate dehydrogenase	18,382	29413,639	5,349	3		97079	108441	18,6	37,2	1,12	0,58	0,65
B1XBS3_ECODH	*aroF*	Phospho-2-dehydro-3-deoxyheptonate aldolase	11,798	38803,961	5,423	2		95023	207767	12,2	11,7	2,19	0,00	0,00
B1X785_ECODH	*aroG*	Phospho-2-dehydro-3-deoxyheptonate aldolase	30	38009,523	6,138	7		1208585	1521766	4,0	4,2	1,26	0,00	0,00
B1XG11_ECODH	*aroH*	Phospho-2-dehydro-3-deoxyheptonate aldolase	9,77	38735,055	6,423	3		104772	93206	21,5	20,0	0,89	0,45	0,54
AROK_ECODH	*aroK*	Shikimate kinase 1	21,965	19538,047	5,263	3		1327601	1371764	18,5	9,0	1,03	0,75	0,81
B1X804_ECODH	*artI*	Arginine transporter subunit periplasmic-binding	32,922	26929,557	5,792	5		613296	757443	13,4	4,3	1,24	0,03	0,06
B1X778_ECODH	*asd*	Aspartate-semialdehyde dehydrogenase	35,15	40017,883	5,369	8		3218349	6837929	4,2	15,7	2,12	0,00	0,00
ASNA_ECODH	*asnA*	Aspartate--ammonia ligase	19,091	36650,555	5,453	5		421725	609651	12,1	15,5	1,45	0,01	0,03
B1X6K7_ECODH	*asnB*	Asparagine synthetase	18,953	62659,012	5,55	6		572270	1311716	10,1	3,2	2,29	0,00	0,00
B1X8N4_ECODH	*asnS*	Asparagine--tRNA ligase	43,991	52570,441	5,172	17		11837356	12925830	4,2	7,9	1,09	0,10	0,16
B1XD19_ECODH	*aspA*	Aspartate ammonia-lyase	37,657	52356,129	5,191	13		17083751	5513139	6,4	5,1	0,32	0,00	0,00
B1X8N2_ECODH	*aspC*	Aspartate aminotransferase, PLP-dependent	55,051	43573,359	5,544	17		6576795	8254368	4,6	4,3	1,26	0,00	0,00
SYD_ECODH	*aspS*	Aspartate--tRNA ligase	44,915	65913,445	5,472	19		10042086	8593021	9,3	6,4	0,86	0,03	0,07
ASTC_ECODH	*astC*	Succinylornithine transaminase	11,084	43665,375	5,913	2		188320	136854	10,8	14,2	0,73	0,01	0,03
ATPA_ECODH	*atpA*	ATP synthase subunit alpha	50,097	55222,078	5,796	17		27712376	32096419	4,4	5,9	1,16	0,01	0,02
ATPB_ECODH	*atpD*	ATP synthase subunit beta	73,913	50325,422	4,902	21		50264832	55312070	5,7	6,9	1,10	0,07	0,13
ATPF_ECODH	*atpF*	ATP synthase subunit b	46,795	17263,957	5,991	6		2658358	3257345	11,6	14,9	1,23	0,07	0,13
ATPG_ECODH	*atpG*	ATP synthase gamma chain	41,115	31577,42	8,839	9		4866229	5660729	4,6	7,1	1,16	0,01	0,03
ATPD_ECODH	*atpH*	ATP synthase subunit delta	52,542	19332,225	4,945	7		3502126	3773412	9,8	12,3	1,08	0,38	0,47

B1X8I8_ECODH	*avtA*	Valine-pyruvate aminotransferase	15,588	46711,227	5,65	6		231258	278510	12,4	7,4	1,20	0,05	0,09
B1XDC5_ECODH	*azoR*	FMN-dependent NADH-azoreductase	24,378	21657,717	5,063	3		400931	340757	10,7	8,3	0,85	0,06	0,10
B1X7H5_ECODH	*baeR*	DNA-binding response regulator in two-compone	47,083	27655,934	5,522	7		282842	280234	8,2	18,1	0,99	0,93	0,94
BAMA_ECODH	*bamA*	Outer membrane protein assembly factor BamA	42,469	90552,766	4,93	26		8280189	10002239	5,1	12,1	1,21	0,03	0,06
B1XAF2_ECODH	*bcp*	Thiol peroxidase, thioredoxin-dependent	64,744	17633,936	5,032	7		4321502	3622540	4,5	12,4	0,84	0,04	0,08
BETB_ECODH	*betB*	NAD/NADP-dependent betaine aldehyde dehydr	11,633	52911,133	5,19	2		197948	271126	18,6	26,4	1,37	0,10	0,17
B1X6I7_ECODH	*bfr*	Bacterioferritin	60,759	18495,027	4,69	7		2682373	2087858	4,9	10,2	0,78	0,00	0,01
B1XEI8_ECODH	*bglA*	6-phospho-beta-glucosidase A	34,447	55360,766	5,577	14		2614121	2586899	3,6	8,5	0,99	0,83	0,86
B1X7M1_ECODH	*bglX*	Beta-D-glucoside glucohydrolase, periplasmic	22,876	83460,047	5,854	12		779213	738870	8,8	7,2	0,95	0,38	0,47
B1XAM7_ECODH	*bipA*	GTP-binding protein	26,359	67355,453	5,16	12		6358882	7774137	7,6	5,0	1,22	0,00	0,01
B1XDQ4_ECODH	*blc*	Outer membrane lipoprotein (Lipocalin)	10,169	19851,607	8,884	2		25506	23714	9,9	30,8	0,93	0,71	0,77
B1XFM3_ECODH	*bolA*	Regulator of penicillin binding proteins and beta	15,238	11993,675	6,187	1		260716	179369	21,1	14,6	0,69	0,04	0,07
B1XBX7_ECODH	*btuB*	Vitamin B12 transporter BtuB	19,055	68407,148	5,226	8		935209	838874	6,3	12,1	0,90	0,18	0,26
B1XG17_ECODH	*btuE*	Glutathione peroxidase	47,541	20469,557	4,812	6		739373	436921	11,7	10,4	0,59	0,00	0,00
B1XD12_ECODH	*cadA*	Lysine decarboxylase 1	20,28	81260,125	5,914	10	acid stress response	723120	349358	21,6	9,5	0,48	0,00	0,00
B1XCA1_ECODH	*can*	Carbonic anhydrase	21,364	25096,768	6,164	4		1849930	2796569	12,7	9,8	1,51	0,00	0,01
B1XBF8_ECODH	*carB*	Carbamoyl-phosphate synthase large chain	14,632	117841,72	5,221	11		1163861	1715929	44,1	18,5	1,47	0,13	0,20
CBPA_ECODH	*cbpA*	Curved DNA-binding protein	11,765	34455,008	6,327	2		568691	336611	6,8	6,3	0,59	0,00	0,00
CDD_ECODH	*cdd*	Cytidine deaminase	55,782	31539,869	5,419	9		3010913	2543537	5,8	19,2	0,84	0,15	0,22
B1XFX1_ECODH	*cfa*	Cyclopropane fatty acyl phospholipid synthase (U	9,686	43909,004	5,694	3		164741	278307	19,3	44,0	1,69	0,12	0,19
B1X7P3_ECODH	*cirA*	Ferric iron-catecholate outer membrane transpo	13,876	73895,727	5,106	5	transport	69349	417816	23,5	27,5	6,02	0,00	0,00
B1X6W4_ECODH	*cld*	Regulator of length of O-antigen component of li	31,595	36454,746	5,427	6		1197243	676483	13,3	13,4	0,57	0,00	0,00
B1X823_ECODH	*clpA*	ATPase and specificity subunit of ClpA-ClpP ATI	22,296	84206,93	5,911	13		1740778	2327742	6,2	6,2	1,34	0,00	0,00
B1XBR5_ECODH	*clpB*	Protein disaggregation chaperone	47,841	95585,023	5,367	30		18857061	34794610	3,1	11,9	1,85	0,00	0,00
B1XFM5_ECODH	*clpP*	ATP-dependent Clp protease proteolytic subunit	25,121	23186,648	5,523	4		3108536	4249979	15,9	11,3	1,37	0,02	0,04
CLPS_ECODH	*clpS*	ATP-dependent Clp protease adapter protein Clp	32,075	12179,059	4,936	2		36012	62172	12,5	11,2	1,73	0,00	0,00
CLPX_ECODH	*clpX*	ATP-dependent Clp protease ATP-binding subur	47,642	46356,008	5,236	12		4844448	6834750	3,0	10,3	1,41	0,00	0,00
KCY_ECODH	*cmk*	Cytidylate kinase	26,432	24746,342	5,559	4		421511	343989	10,6	14,7	0,82	0,06	0,11
CMOA_ECODH	*cmoA*	tRNA (cmo5U34)-methyltransferase	37,652	27776,645	5,357	6		741466	645268	4,3	13,8	0,87	0,09	0,16
COAA_ECODH	*coaA*	Pantothenate kinase	12,025	36359,777	6,325	2		142844	131337	9,4	13,4	0,92	0,34	0,44
B1XA30_ECODH	*cobB*	NAD-dependent protein deacylase	17,204	31464,039	7,788	3		221146	203941	12,9	13,1	0,92	0,40	0,49
B1XAI1_ECODH	*corA*	Magnesium/nickel/cobalt transporter	39,873	36589,711	4,636	8		995570	1685099	6,6	25,0	1,69	0,01	0,02
B1X653_ECODH	*corC*	Predicted ion transport	6,507	33298,488	4,55	2		178215	220675	14,4	15,9	1,24	0,09	0,15
B1XG37_ECODH	*cpdA*	3',5'-cyclic adenosine monophosphate phosphoc	13,818	30937,996	5,543	3		145325	104761	17,8	23,4	0,72	0,08	0,13
B1XDW3_ECODH	*cpdB*	2':3'-cyclic-nucleotide 2'-phosphodiesterase	27,202	70832,195	5,45	9		1337862	777302	9,6	10,7	0,58	0,00	0,00
B1XB80_ECODH	*cpxP*	Periplasmic protein combats stress	27,711	18965,246	6,379	3	stress response	65948	924007	8,5	13,8	14,01	0,00	0,00
B1XB79_ECODH	*cpxR*	DNA-binding response regulator in two-compone	25,431	26312,113	5,393	5	stress response	383597	1158355	30,9	14,3	3,02	0,00	0,00
B1XFK7_ECODH	*creA*	Protein CreA	29,936	17107,891	9,037	3		193212	154752	9,2	22,1	0,80	0,12	0,19
CRL_ECODH	*crl*	Sigma factor-binding protein Crl	22,556	15654,994	6,316	3		567209	322310	9,1	11,9	0,57	0,00	0,00
B1X705_ECODH	*crp*	DNA-binding transcriptional dual regulator	42,381	23640,426	8,376	8		5370359	4549700	9,4	6,1	0,85	0,03	0,06
B1XA88_ECODH	*crr*	Glucose-specific enzyme IIA component of PTS	58,58	18251,068	4,734	6		16009474	16162782	8,1	5,4	1,01	0,86	0,89
B1X8H1_ECODH	*cspA*	Major cold shock protein	31,429	7403,278	5,578	2		1432668	1191557	12,5	12,7	0,83	0,08	0,14
B1XH90_ECODH	*cspC*	Stress protein, member of the CspA-family	63,768	7402,368	6,539	3		4674281	1988045	7,9	17,3	0,43	0,00	0,00
B1X821_ECODH	*cspD*	Cold shock protein-like protein	55,405	7968,975	5,808	2		1596597	935553	23,8	27,5	0,59	0,03	0,07
B1X620_ECODH	*cspE*	DNA-binding transcriptional repressor	53,623	7463,451	8,087	3		11926045	7291459	8,1	16,8	0,61	0,00	0,01
B1X5Z5_ECODH	*cstA*	Carbon starvation protein	6,134	75105,234	8,01	3		212933	293967	14,1	18,0	1,38	0,03	0,06
B1XC98_ECODH	*cueO*	Multicopper oxidase (Laccase)	12,791	56556,152	6,28	3		83284	131312	19,8	61,4	1,58	0,19	0,27
CUTA_ECODH	*cutA*	Divalent-cation tolerance protein CutA	22,321	12331,083	4,852	1		35065	40744	26,3	13,5	1,16	0,34	0,44
CYAY_ECODH	*cyaY*	Protein CyaY	41,509	12231,413	4,241	3		40103	59895	33,7	96,7	1,49	0,40	0,50
B1XDV8_ECODH	*cycA*	D-alanine/D-serine/glycine transporter	5,745	51659,656	8,918	2		75310	137185	9,4	4,8	1,82	0,00	0,00
B1X6R3_ECODH	*cydA*	Cytochrome d terminal oxidase, subunit I	17,816	58205,082	6,353	8	acid stress response	7613775	2695046	4,8	10,6	0,35	0,00	0,00
B1X6R4_ECODH	*cydB*	Cytochrome d terminal oxidase, subunit II	5,805	42453,117	6,901	2	acid stress response	1673172	612605	6,0	31,0	0,37	0,00	0,01
B1X826_ECODH	*cydC*	Fused cysteine transporter subunits of ABC sup	11,344	62920,168	8,754	4		399104	595767	8,6	13,0	1,49	0,00	0,01
B1X827_ECODH	*cydD*	Fused cysteine transporter subunits of ABC sup	12,925	65056,191	5,997	4		295663	565979	6,1	14,3	1,91	0,00	0,00
B1XFM0_ECODH	*cyoA*	Cytochrome o ubiquinol oxidase subunit II	33,333	34911,297	6,758	6		7281483	5027573	14,6	6,1	0,69	0,00	0,01
B1XFL9_ECODH	*cyoB*	Cytochrome o ubiquinol oxidase subunit I	7,994	74367,906	6,698	5		1797954	1743439	15,8	11,1	0,97	0,76	0,81
B1XBM4_ECODH	*cysB*	DNA-binding transcriptional dual regulator, O-ac	14,815	36150,469	6,873	4		182232	91471	10,6	9,6	0,50	0,00	0,00
B1X941_ECODH	*cysE*	Serine acetyltransferase	20,513	29316,639	6,05	3		265597	422363	19,5	10,9	1,59	0,01	0,02
B1XA85_ECODH	*cysK*	Cysteine synthase	45,82	34489,652	5,829	11		5522376	11307700	4,9	24,4	2,05	0,00	0,01
SYC_ECODH	*cysS*	Cysteine--tRNA ligase	35,792	52202,086	5,326	13		1917801	1725009	19,2	12,8	0,90	0,40	0,49
B1XBA0_ECODH	*cytR*	DNA-binding transcriptional dual regulator	11,437	37819,781	6,015	2		64038	94275	12,4	38,0	1,47	0,08	0,14
B1X627_ECODH	*dacA*	D-alanyl-D-alanine carboxypeptidase (Penicillin-l	28,288	44443,957	8,31	9		1799978	2035961	10,4	9,0	1,13	0,13	0,20
B1X7Y0_ECODH	*dacC*	D-alanyl-D-alanine carboxypeptidase (Penicillin-l	43,25	43608,906	7,738	12	ptidoglycan-modificat	1663586	5204403	20,2	16,6	3,13	0,00	0,00
DADA_ECODH	*dadA*	D-amino acid dehydrogenase small subunit	40,278	47607,293	6,174	12		2723001	2699323	5,7	11,7	0,99	0,89	0,92
B1X735_ECODH	*damX*	Uncharacterized protein	10,748	46161,91	5,687	3		694871	673881	13,9	16,1	0,97	0,79	0,83
B1XAF0_ECODH	*dapA*	4-hydroxy-tetrahydrodipicolinate synthase	34,247	31269,971	5,981	7		2699024	2441564	3,9	10,3	0,90	0,13	0,20
DAPB_ECODH	*dapB*	4-hydroxy-tetrahydrodipicolinate reductase	11,355	28756,613	5,446	3		356577	389647	9,4	16,6	1,09	0,40	0,49
DAPD_ECODH	*dapD*	2,3,4,5-tetrahydropyridine-2,6-dicarboxylate N-su	34,672	29892,098	5,563	8		11244404	15719269	7,3	7,3	1,40	0,00	0,00
DAPE_ECODH	*dapE*	Succinyl-diaminopimelate desuccinylase	21,867	41269,012	5,307	6		364110	400081	9,5	7,0	1,10	0,16	0,25
DAPF_ECODH	*dapF*	Diaminopimelate epimerase	8,029	30208,562	5,856	2		258757	173677	31,7	11,7	0,67	0,08	0,14
B1XEC5_ECODH	*dcp*	Dipeptidyl carboxypeptidase II	26,872	77515,75	5,488	12		1449804	1777731	6,4	3,5	1,23	0,00	0,01
B1X7T0_ECODH	*dcrB*	Periplasmic protein	23,784	19787,492	5,085	3		2705675	2903452	11,0	12,8	1,07	0,43	0,52
B1XD18_ECODH	*dcuA*	Anaerobic C4-dicarboxylate transporter	3,926	45750,707	7,629	2		413352	152595	3,2	15,7	0,37	0,00	0,00
B1XEX0_ECODH	*ddlA*	D-alanine--D-alanine ligase	35,714	39315,809	5,02	9		2049956	1283845	4,3	11,3	0,63	0,00	0,00

B1XC69_ECODH	*ddlB*	D-alanine--D-alanine ligase	17,974	32839,707	4,772	4		780643	815866	11,7	3,4	1,05	0,50	0,59
B1XGX4_ECODH	*deaD*	ATP-dependent RNA helicase DeaD	29,253	70546,156	8,746	14		4234079	4351171	7,2	6,4	1,03	0,59	0,66
DEF_ECODH	*def*	Peptide deformylase	15,976	19328,422	5,229	2		416736	390463	18,5	23,5	0,94	0,70	0,76
B1XD31_ECODH	*degP*	Serine endoprotease (Protease Do), membrane-	41,772	49354,23	8,648	13	degradation/folding	5493300	61160686	7,4	7,5	11,13	0,00	0,00
B1XHK7_ECODH	*degQ*	Serine endoprotease, periplasmic	18,022	47205,055	5,763	4		371799	433031	8,2	15,1	1,16	0,13	0,20
TYPH_ECODH	*deoA*	Thymidine phosphorylase	62,727	47207,121	5,214	19		3638898	4890665	6,8	7,5	1,34	0,00	0,00
DEOB_ECODH	*deoB*	Phosphopentomutase	60,934	44369,961	5,106	17		8957027	10218739	4,2	7,4	1,14	0,02	0,04
DEOC_ECODH	*deoC*	Deoxyribose-phosphate aldolase	61,004	27733,801	5,496	12		6588487	8318717	3,5	14,4	1,26	0,02	0,05
DEOD_ECODH	*deoD*	Purine nucleoside phosphorylase DeoD-type	64,435	25949,91	5,418	11		10616246	16935519	5,8	8,6	1,60	0,00	0,00
DER_ECODH	*der*	GTPase Der	21,837	55035,875	5,596	8		1452154	1765150	4,4	10,6	1,22	0,02	0,04
B1X973_ECODH	*dfp*	Fused 4'-phosphopantothenoylcysteine decarbo:	22,414	43438,141	7,056	5		321417	287712	20,0	12,5	0,90	0,41	0,51
B1XAP1_ECODH	*dhaK*	Dihydroxyacetone kinase, N-terminal domain	16,854	38215,207	4,815	3		1678691	1352507	7,0	13,7	0,81	0,03	0,06
B1XAP0_ECODH	*dhaL*	Dihydroxyacetone kinase, C-terminal domain	58,571	22631,746	5,309	7		1185980	869879	15,4	17,1	0,73	0,04	0,08
B1XAN9_ECODH	*dhaM*	Fused predicted dihydroxyacetone-specific PTS	31,78	51448,621	4,612	9		1628995	1749732	7,3	14,4	1,07	0,40	0,49
B1XC56_ECODH	*djlA*	DnaJ-like protein DjlA	8,118	30579,363	9,744	2		117821	113968	2,5	22,7	0,97	0,79	0,83
B1XFG4_ECODH	*dkgA*	2,5-diketo-D-gluconate reductase A	22,182	31109,637	5,999	5		739654	427654	15,5	11,6	0,58	0,00	0,00
B1XD71_ECODH	*dkgB*	2,5-diketo-D-gluconate reductase B	8,989	29436,791	5,492	2		84454	95282	30,9	8,2	1,13	0,53	0,61
B1XCC0_ECODH	*dksA*	RNA polymerase-binding transcription factor Dks	23,841	17527,752	5,063	3		4180235	3247460	13,9	7,8	0,78	0,02	0,05
B1X7M2_ECODH	*dld*	D-lactate dehydrogenase	24,343	64612,332	6,19	11		755846	773385	3,5	13,6	1,02	0,75	0,80
B1X834_ECODH	*dmsA*	Dimethyl sulfoxide reductase, anaerobic, subunit	9,459	90398,617	6,42	6		579529	122081	8,9	14,9	0,21	0,00	0,00
B1XCU0_ECODH	*dnaB*	Replicative DNA helicase	10,828	52390,082	4,946	3		310124	239507	5,9	26,8	0,77	0,09	0,16
B1XD53_ECODH	*dnaE*	DNA polymerase III alpha subunit	1,897	129904,57	5,165	2		33528	34709	11,3	11,1	1,04	0,66	0,73
B1XG71_ECODH	*dnaG*	DNA primase	6,885	65564,625	5,685	2		26015	60756	53,7	25,8	2,34	0,04	0,08
DNAJ_ECODH	*dnaJ*	Chaperone protein DnaJ	23,404	41100,332	7,977	7		1431518	2871663	12,8	15,6	2,01	0,00	0,00
B1XBD9_ECODH	*dnaK*	Chaperone protein DnaK	54,859	69114,961	4,831	23		51520712	111343654	3,7	16,4	2,16	0,00	0,00
B1X9T0_ECODH	*dnaN*	DNA polymerase III subunit beta	17,213	40586,602	5,25	4		749729	874283	6,2	7,5	1,17	0,02	0,05
DNAT_ECODH	*dnaT*	Primosomal protein 1	12,849	19455,121	5,145	1		39686	29046	18,1	9,7	0,73	0,02	0,05
B1X8G0_ECODH	*dppA*	Dipeptide transporter periplasmic-binding compo	14,766	60293,672	6,211	4		638159	827793	19,9	36,8	1,30	0,24	0,34
DPS_ECODH	*dps*	DNA protection during starvation protein	66,467	18695,307	5,723	9		16441117	2836746	7,6	6,9	0,17	0,00	0,00
B1XAL7_ECODH	*dsbA*	Thiol:disulfide interchange protein	24,519	23104,537	5,946	3	degradation/folding	611814	3100010	11,1	21,0	5,07	0,00	0,00
B1XEI0_ECODH	*dsbC*	Protein disulfide isomerase II	17,797	25621,656	6,296	4		140652	180781	17,6	20,0	1,29	0,10	0,17
B1X602_ECODH	*dsbG*	Periplasmic disulfide isomerase/thiol-disulphide (	12,5	27494,99	8,318	2		138660	106022	4,9	22,7	0,76	0,07	0,13
SDHD_ECODH	*dsdA*	D-serine dehydratase	42,986	47900,57	5,584	11		5072805	3135122	7,6	12,5	0,62	0,00	0,00
B1XCT7_ECODH	*dusA*	tRNA-dihydrouridine synthase	22,899	38467,898	6,116	4		336998	370179	12,0	7,7	1,10	0,25	0,35
DUT_ECODH	*dut*	Deoxyuridine 5'-triphosphate nucleotidohydrolas	32,45	16155,47	5,031	2		965588	1624001	18,1	36,7	1,68	0,06	0,11
B1XD42_ECODH	*dxr*	1-deoxy-D-xylulose 5-phosphate reductoisomera	5,779	43387,977	5,675	2		45176	103105	21,1	21,5	2,28	0,00	0,01
DXS_ECODH	*dxs*	1-deoxy-D-xylulose-5-phosphate synthase	16,452	67616,938	6,164	8		846079	906688	8,2	4,5	1,07	0,19	0,27
NQOR_ECODH	*CDH10B_107*	NAD(P)H dehydrogenase (quinone)	51,01	20845,555	5,594	7		4789765	3030128	7,0	4,1	0,63	0,00	0,00
CINAL_ECODH	*CDH10B_240*	CinA-like protein	20,5	44225,645	5,463	5		255474	142777	14,6	10,2	0,56	0,00	0,00
B1XDQ2_ECODH	*ecnB*	Entericidin B membrane lipoprotein	39,583	4809,528	7,934	1		1924480	895306	12,3	9,6	0,47	0,00	0,00
ECOT_ECODH	*eco*	Ecotin	43,827	18192,049	6,602	5		1144393	838155	22,1	13,5	0,73	0,05	0,10
B1XHB8_ECODH	*eda*	Multifunctional 2-keto-3-deoxygluconate 6-phosp	34,272	22284,027	5,569	5		3666416	4392457	12,4	13,5	1,20	0,10	0,16
EFP_ECODH	*efp*	Elongation factor P	17,021	20591,314	4,897	2		2652391	1720701	15,4	16,4	0,65	0,01	0,03
B1X8Y0_ECODH	*elaB*	Conserved protein	27,723	11305,689	5,35	2		727051	654134	60,3	17,9	0,90	0,72	0,77
B1XHI4_ECODH	*elbB*	Isoprenoid biosynthesis protein with amidotransf	23,041	22981,551	4,675	3		1498034	977386	6,9	3,1	0,65	0,00	0,00
B1XCL8_ECODH	*emrA*	Multidrug efflux system	8,205	42736,047	9,333	2		225074	61316	13,0	11,8	0,27	0,00	0,00
EMTA_ECODH	*emtA*	Endo-type membrane-bound lytic murein transgl	35,961	22226,547	9,161	4		313275	348998	6,0	8,7	1,11	0,09	0,15
ENO_ECODH	*eno*	Enolase	52,546	45654,945	5,324	14		57757965	55010428	4,5	7,6	0,95	0,30	0,40
B1X5Z2_ECODH	*entB*	Isochorismatase	8,772	32554,338	5,046	2	metabolism	121152	272268	13,2	27,1	2,25	0,00	0,01
B1X5Z1_ECODH	*entE*	Enterobactin synthetase component E	11,754	59112,152	5,419	3	metabolism	40207	116857	14,3	22,3	2,91	0,00	0,00
E4PD_ECODH	*epd*	D-erythrose-4-phosphate dehydrogenase	14,749	37299,391	6,264	5		230172	216458	4,0	15,9	0,94	0,49	0,58
ERA_ECODH	*era*	GTPase Era	19,934	33810,066	6,73	5		565770	519367	2,8	10,2	0,92	0,17	0,26
ERPA_ECODH	*erpA*	Iron-sulfur cluster insertion protein ErpA	18,421	12100,484	4,109	2		805230	1218572	17,1	17,7	1,51	0,02	0,04
B1XAB2_ECODH	*eutB*	Ethanolamine ammonia-lyase, large subunit, hea	8,168	49403,047	4,789	3		114446	121147	27,6	14,5	1,06	0,73	0,78
B1XFF9_ECODH	*exbB*	Membrane spanning protein in TonB-ExbB-ExbD	16,393	26287,387	7,888	3		699985	1113486	3,1	25,0	1,59	0,01	0,03
B1XFF8_ECODH	*exbD*	Membrane spanning protein in TonB-ExbB-ExbD	13,475	15526,983	4,698	2		50998	89667	23,0	6,2	1,76	0,00	0,01
B1XG98_ECODH	*exuR*	DNA-binding transcriptional repressor	13,953	29835,695	5,395	3		513301	447039	7,3	21,6	0,87	0,25	0,34
FABA_ECODH	*fabA*	3-hydroxydecanoyl-[acyl-carrier-protein] dehydra	19,186	18969,049	6,126	3		4282581	2982515	9,6	6,0	0,70	0,00	0,00
B1X934_ECODH	*fabB*	3-oxoacyl-[acyl-carrier-protein] synthase I	39,655	42613,32	5,347	10		6604220	7751363	9,1	9,8	1,17	0,06	0,11
B1XA02_ECODH	*fabD*	Malonyl CoA-acyl carrier protein transacylase	45,307	32417,203	4,947	7		10883343	6511805	4,9	9,3	0,60	0,00	0,00
B1XA05_ECODH	*fabF*	3-oxoacyl-[acyl-carrier-protein] synthase 2	33,414	43045,766	5,714	8		3699537	4496690	12,6	1,9	1,22	0,02	0,05
B1XA03_ECODH	*fabG*	3-oxoacyl-[acyl-carrier-protein] reductase	54,508	25560,295	6,763	9		7000366	5780451	11,8	23,7	0,83	0,19	0,27
B1XA01_ECODH	*fabH*	3-oxoacyl-[acyl-carrier-protein] synthase 3	38,801	33515,121	5,078	8		4216170	3754157	4,6	7,6	0,89	0,04	0,08
B1XBN9_ECODH	*fabI*	Enoyl-[acyl-carrier-protein] reductase [NADH]	50,382	27863,939	5,577	9		18240176	17677299	5,5	5,6	0,97	0,46	0,55
FABZ_ECODH	*fabZ*	3-hydroxyacyl-[acyl-carrier-protein] dehydratase	28,477	17032,953	6,84	4		2510322	1661011	7,1	12,2	0,66	0,00	0,00
FADB_ECODH	*fadB*	Fatty acid oxidation complex subunit alpha	7,682	79593,914	5,838	4	metabolism	116228	240191	10,3	18,8	2,07	0,00	0,00
B1XD83_ECODH	*fadE*	Acyl coenzyme A dehydrogenase	3,931	89224,445	8,221	2		60777	62748	12,8	24,8	1,03	0,81	0,85
B1X9L7_ECODH	*fadL*	Long-chain fatty acid outer membrane transporte	25,223	48771,914	5,086	7		3080747	2763469	7,3	11,5	0,90	0,18	0,26
FADR_ECODH	*fadR*	Fatty acid metabolism regulator protein	30,544	26968,609	6,507	5		315983	416198	16,4	22,1	1,32	0,09	0,15
B1XEK8_ECODH	*fbaA*	Fructose-bisphosphate aldolase, class II	32,869	39147,262	5,524	7		24756889	28792193	7,0	6,1	1,16	0,02	0,04
B1X7I8_ECODH	*fbaB*	Fructose-bisphosphate aldolase class I	43,714	38109,191	6,248	11		2042080	2272523	7,7	6,3	1,11	0,08	0,14
F16PA_ECODH	*fbp*	Fructose-1,6-bisphosphatase class 1	39,157	36833,934	5,665	9		2202928	1569253	4,8	4,7	0,71	0,00	0,00
FDHE_ECODH	*fdhE*	Protein FdhE	8,091	34746,711	5,033	2		269150	179833	9,6	15,8	0,67	0,01	0,02
B1XE65_ECODH	*fdnG*	Formate dehydrogenase-N, alpha subunit, nitrat	33,3	112963,34	6,543	22		5395074	1674952	7,1	25,0	0,31	0,00	0,00

B1XE66_ECODH	*fdnH*	Formate dehydrogenase-N, Fe-S (Beta) subunit,	14,286	32238,719	5,575	4		501819	163601	16,1	30,4	0,33	0,00	0,01
B1XB62_ECODH	*fdoG*	Formate dehydrogenase-O, large subunit	42,52	112536,41	6,883	25		8587833	8276579	7,5	5,1	0,96	0,45	0,54
B1XB61_ECODH	*fdoH*	Formate dehydrogenase-O, Fe-S subunit	20,667	33100,383	5,166	5		403116	706577	39,9	25,2	1,75	0,04	0,08
B1XB60_ECODH	*fdoI*	Formate dehydrogenase-O, cytochrome b556 su	9,005	24606,365	10,21	2		213173	252654	21,3	22,0	1,19	0,34	0,44
B1XB00_ECODH	*fdx*	[2Fe-2S] ferredoxin	55,856	12330,815	4,491	4		1298059	1449851	18,3	9,8	1,12	0,36	0,46
B1XER5_ECODH	*fecA*	KpLE2 phage-like element ferric citrate outer me	18,088	85321,609	5,59	10		1755417	1268625	12,0	17,7	0,72	0,02	0,05
B1XER4_ECODH	*fecB*	KpLE2 phage-like element iron-dicitrate transpor	9,333	33146,348	8,832	2		174724	99150	11,5	49,7	0,57	0,04	0,08
B1X755_ECODH	*feoA*	Ferrous iron transporter, protein A	21,333	8370,826	9,425	1		39783	17071	25,3	69,9	0,43	0,38	0,47
B1X756_ECODH	*feoB*	Fused ferrous iron transporter, protein B: GTP-b	5,304	84473,656	5,81	3		192191	163055	6,5	27,2	0,85	0,30	0,40
B1X5Y0_ECODH	*fepA*	Iron-enterobactin outer membrane transporter	13,137	82106,898	5,386	6	transport	135800	843544	12,6	22,9	6,21	0,00	0,00
B1XBT2_ECODH	*ffh*	Signal recognition particle protein	43,929	49787,305	9,517	14		3437472	4002681	3,9	3,2	1,16	0,00	0,00
B1XCC5_ECODH	*fhuA*	Ferrichrome outer membrane transporter	34,672	82114,953	5,465	15		1829618	3624980	2,4	6,5	1,98	0,00	0,00
FIS_ECODH	*fis*	DNA-binding protein Fis	32,653	11239,932	9,344	2		5978028	6304085	11,4	13,4	1,05	0,57	0,64
B1XDV7_ECODH	*fklB*	Peptidyl-prolyl cis-trans isomerase	43,204	22216,193	4,849	6		6020863	8213711	7,9	5,7	1,36	0,00	0,00
B1X6J6_ECODH	*fkpA*	Peptidyl-prolyl cis-trans isomerase	27,407	28881,926	8,386	6		3459592	3780149	17,7	15,4	1,09	0,49	0,57
B1XBF3_ECODH	*fkpB*	Peptidyl-prolyl cis-trans isomerase	47,651	16081,006	4,3	3		601267	711194	12,3	14,2	1,18	0,13	0,21
B1X6L7_ECODH	*fldA*	Flavodoxin	38,636	19736,941	4,213	4		783980	1316297	48,0	39,1	1,68	0,17	0,25
B1X678_ECODH	*fliY*	Cystine transporter subunit periplasmic-binding c	12,406	29039,189	6,216	3		32216	70074	57,4	16,8	2,18	0,02	0,05
B1X6T5_ECODH	*flu*	CP4-44 prophage antigen 43 (Ag43) phase-varia	35,515	106825,45	5,777	21		26222767	3032371	11,9	19,5	0,12	0,00	0,00
FMT_ECODH	*fmt*	Methionyl-tRNA formyltransferase	46,032	34168,465	5,559	9		1820144	1369636	11,5	14,9	0,75	0,02	0,05
B1XCG3_ECODH	*fnr*	DNA-binding transcriptional dual regulator, globa	17,6	27967,207	8,25	3		982941	1086778	21,5	13,5	1,11	0,45	0,54
B1XC49_ECODH	*folA*	Dihydrofolate reductase	33,333	17999,377	4,839	3		198435	186498	13,1	27,3	0,94	0,70	0,76
B1XG63_ECODH	*folB*	Bifunctional dihydroneopterin aldolase and dihyd	13,934	13619,531	4,678	2		64434	77265	19,2	15,4	1,20	0,17	0,25
B1X926_ECODH	*folC*	Bifunctional protein FolC	19,194	45405,699	5,502	6		398435	407041	6,8	11,8	1,02	0,76	0,81
FOLD_ECODH	*folD*	Bifunctional protein FolD	13,889	31043,748	5,742	3		271822	234461	19,0	10,7	0,86	0,22	0,32
GCH1_ECODH	*folE*	GTP cyclohydrolase 1	34,234	24830,621	6,796	6		728044	1128524	15,4	10,2	1,55	0,00	0,01
B1XGY7_ECODH	*folP*	7,8-dihydropteroate synthase	14,539	30615,111	5,677	3		195235	329906	11,4	14,2	1,69	0,00	0,00
B1X914_ECODH	*folX*	D-erythro-7,8-dihydroneopterin triphosphate 2'-e	31,667	14082,037	6,509	4		947050	916937	12,0	1,8	0,97	0,60	0,67
B1XB90_ECODH	*fpr*	Ferredoxin-NADP reductase	17,742	27750,938	6,174	2		55055	77059	14,8	5,0	1,40	0,00	0,01
B1XDQ8_ECODH	*frdA*	Fumarate reductase (Anaerobic) catalytic and N/	19,934	65971,602	5,863	8		3146135	631114	9,0	9,9	0,20	0,00	0,00
B1XEU9_ECODH	*frmA*	Alcohol dehydrogenase class III/glutathione-dep	12,737	39358,992	5,849	4		607613	1013466	18,5	13,3	1,67	0,01	0,02
B1XEV0_ECODH	*frmR*	Regulator protein that represses frmRAB operon	26,374	10317,724	5,843	2		122134	108684	15,5	29,7	0,89	0,48	0,57
RRF_ECODH	*frr*	Ribosome-recycling factor	36,216	20638,572	6,436	5		6519852	6707829	14,3	8,6	1,03	0,75	0,80
FRSA_ECODH	*frsA*	Esterase FrsA	17,874	47008,77	6,465	5		237580	308335	27,1	13,5	1,30	0,17	0,25
B1X865_ECODH	*fruA*	Fused fructose-specific PTS enzymes: IIBcompo	8,526	57519,113	8,911	4		533297	694239	17,1	9,7	1,30	0,03	0,07
B1X866_ECODH	*fruK*	Phosphofructokinase	22,115	33755,688	5,372	5		1080046	1119561	11,8	9,2	1,04	0,65	0,72
B1X666_ECODH	*ftn*	Ferritin iron storage protein (Cytoplasmic)	27,879	19423,816	4,768	3		2302928	756261	8,1	12,5	0,33	0,00	0,00
B1XC71_ECODH	*ftsA*	Cell division protein ftsA	31,905	45329,969	5,845	6		770387	1243529	23,6	13,3	1,61	0,01	0,03
FTSB_ECODH	*ftsB*	Cell division protein FtsB	13,592	11622,075	6,568	1		15271	31321	102,8	60,3	2,05	0,35	0,45
B1XGY8_ECODH	*ftsH*	ATP-dependent zinc metalloprotease FtsH	37,422	70708,086	5,907	17		7239062	12215006	7,5	4,1	1,69	0,00	0,00
B1XC61_ECODH	*ftsI*	Transpeptidase involved in septal peptidoglycan	14,116	63877,434	9,644	5		219213	248269	11,8	14,6	1,13	0,26	0,36
B1X830_ECODH	*ftsK*	DNA-binding membrane protein required for chro	5,192	146662,72	4,916	3		173804	181188	4,5	9,4	1,04	0,44	0,54
B1XB99_ECODH	*ftsN*	Essential cell division protein	12,853	35793,23	10,17	3		320448	354226	11,5	22,5	1,11	0,48	0,57
B1X7S0_ECODH	*ftsX*	Cell division protein FtsX	9,659	38543,652	9,078	2		85311	154516	23,1	35,3	1,81	0,04	0,08
B1X7S2_ECODH	*ftsY*	Signal recognition particle receptor FtsY	34,004	54513,348	4,471	10		1381534	1664310	13,4	4,8	1,20	0,04	0,09
B1XC72_ECODH	*ftsZ*	Cell division protein FtsZ	53,525	40323,914	4,649	14		8848277	9904663	5,8	7,4	1,12	0,06	0,11
FUCI_ECODH	*fucI*	L-fucose isomerase	5,584	64976,672	5,586	3		287943	82184	17,4	15,4	0,29	0,00	0,00
B1XDK8_ECODH	*fucO*	L-1,2-propanediol oxidoreductase	7,833	40644,562	5,095	2		149165	84219	12,4	21,1	0,56	0,00	0,01
FUCM_ECODH	*fucU*	L-fucose mutarotase	26,429	15473,123	5,57	4		247656	174680	8,3	17,5	0,71	0,01	0,03
B1XF77_ECODH	*fumA*	Fumarate hydratase (Fumarase A), aerobic Clas	20,438	60298,578	6,114	8		5808238	2630762	9,2	11,8	0,45	0,00	0,00
B1XD03_ECODH	*fumB*	Anaerobic class I fumarate hydratase (Fumarase	24,453	60105,324	5,88	8		3232194	1226796	5,0	11,4	0,38	0,00	0,00
EFG_ECODH	*fusA*	Elongation factor G	65,199	77581,305	5,236	30		131344424	149807686	11,2	6,0	1,14	0,08	0,14
B1XD20_ECODH	*fxsA*	Inner membrane protein	18,987	17691,945	8,062	2		99455	191648	6,5	23,0	1,93	0,00	0,01
B1XBW7_ECODH	*gabT*	4-aminobutyrate aminotransferase, PLP-depend	25,822	45774,629	5,778	6		807241	519532	10,9	13,3	0,64	0,00	0,01
B1X7X2_ECODH	*gadA*	Glutamate decarboxylase A, PLP-dependent	48,283	52685,156	5,221	15	acid stress response	27203143	1711380	14,5	17,5	0,06	0,00	0,00
B1XE83_ECODH	*gadC*	Predicted glutamate:gamma-aminobutyric acid a	5,284	55076,672	8,756	3	acid stress response	394920	32426	6,8	33,1	0,08	0,00	0,00
B1X789_ECODH	*galE*	UDP-glucose-4-epimerase	27,811	37325,207	5,887	6		1306312	624377	13,7	10,6	0,48	0,00	0,00
B1X6X8_ECODH	*galF*	Predicted subunit with GalU	50,842	32829,227	5,733	10		2804253	2009851	5,8	7,0	0,72	0,00	0,00
B1X787_ECODH	*galM*	Aldose 1-epimerase	55,78	38190,457	4,845	12		2798592	2253268	5,1	2,3	0,81	0,00	0,00
B1XAS8_ECODH	*galU*	Glucose-1-phosphate uridylyltransferase	40,066	32942,258	5,108	8		5212777	4291403	4,0	6,5	0,82	0,00	0,01
B1XGN9_ECODH	*gapA*	Glyceraldehyde-3-phosphate dehydrogenase A	69,184	35532,488	6,609	16		107287691	157325201	4,5	4,9	1,47	0,00	0,00
B1XGT8_ECODH	*garR*	Tartronate semialdehyde reductase	20,608	30659,734	5,585	3		244436	98396	16,7	21,7	0,40	0,00	0,00
B1X7I6_ECODH	*gatA*	Galactitol-specific enzyme IIA component of PTS	44,667	16907,457	5,148	5		7059180	3030733	6,3	11,5	0,43	0,00	0,00
B1X7I5_ECODH	*gatB*	Galactitol-specific enzyme IIB component of PTS	63,83	10222,037	5,84	3		11562664	5262964	25,8	19,1	0,46	0,00	0,01
B1X7I4_ECODH	*gatC*	Galactitol-specific enzyme IIC component of PTS	10,421	48364,996	9,319	5		3474304	1792518	8,0	15,4	0,52	0,00	0,00
B1X7I3_ECODH	*gatD*	Galactitol-1-phosphate dehydrogenase, Zn-depe	38,15	37390,137	5,939	10		5428339	3544746	4,8	4,2	0,65	0,00	0,00
GATY_ECODH	*gatY*	D-tagatose-1,6-bisphosphate aldolase subunit G	33,803	30811,932	5,874	6		9672090	9796176	6,8	11,9	1,01	0,86	0,89
GCSP_ECODH	*gcvP*	Glycine dehydrogenase (decarboxylating)	27,9	104376,35	5,623	16		6939598	6696837	6,1	10,2	0,97	0,56	0,64
B1XGM2_ECODH	*gdhA*	Glutamate dehydrogenase	15,213	48581,375	5,982	5		118377	276080	4,7	12,1	2,33	0,00	0,00
GHRA_ECODH	*ghrA*	Glyoxylate/hydroxypyruvate reductase A	23,397	35343,422	6,317	4		237582	558020	14,6	22,3	2,35	0,00	0,00
GHRB_ECODH	*ghrB*	Glyoxylate/hydroxypyruvate reductase B	26,235	35395,504	5,5	7		860293	1168370	9,3	9,5	1,36	0,00	0,01
B1XFD1_ECODH	*glcB*	Malate synthase G	14,938	80488,516	5,792	9		560147	518787	4,7	11,6	0,93	0,25	0,35
B1XFD2_ECODH	*glcG*	Conserved protein	11,194	13736,714	5,77	1		82445	55674	13,1	12,1	0,68	0,00	0,01
B1XBB1_ECODH	*gldA*	Glycerol dehydrogenase, NAD	24,796	38712,199	4,813	7		704695	290254	4,5	3,6	0,41	0,00	0,00

B1X6X2_ECODH	*glf*	UDP-galactopyranose mutase, FAD/NAD(P)-bin	39,237	42965,883	6,615	12		3475905	2627319	6,0	9,7	0,76	0,00	0,01
GLGA_ECODH	*glgA*	Glycogen synthase	12,998	52822,426	6,145	5		290004	231784	7,2	23,4	0,80	0,10	0,17
B1X777_ECODH	*glgB*	1,4-alpha-glucan branching enzyme GlgB	4,258	84336,898	5,912	2		69647	76589	17,3	22,8	1,10	0,56	0,64
GLGC_ECODH	*glgC*	Glucose-1-phosphate adenylyltransferase	19,722	48697,645	5,774	5		501598	524542	17,8	11,7	1,05	0,71	0,76
B1X773_ECODH	*glgP*	Phosphorylase	6,994	93172,555	5,572	4		145342	130865	57,6	6,0	0,90	0,69	0,75
GLK_ECODH	*glk*	Glucokinase	17,134	34723,086	6,061	5		1239235	937365	9,1	18,5	0,76	0,03	0,06
GLMM_ECODH	*glmM*	Phosphoglucosamine mutase	40,674	47543,574	5,711	13		3315975	3054515	3,9	6,6	0,92	0,08	0,14
GLMU_ECODH	*glmU*	Bifunctional protein GlmU	21,93	49190,086	6,087	8		708249	602515	8,0	14,1	0,85	0,10	0,16
B1XAM6_ECODH	*glnA*	Glutamine synthetase	47,761	51903,762	5,264	13		5450693	4612418	7,0	9,3	0,85	0,03	0,06
B1XB28_ECODH	*glnB*	Regulatory protein P-II for glutamine synthetase	33,929	12425,448	5,17	3		557122	470912	6,2	15,1	0,85	0,08	0,13
GLNE_ECODH	*glnE*	Glutamate-ammonia-ligase adenylyltransferase	3,383	108418,41	5,078	2		49040	58597	9,9	23,2	1,19	0,21	0,30
B1X7E1_ECODH	*glnH*	Glutamine transporter subunit periplasmic bindin	45,968	27190,117	8,439	8		939650	666847	7,7	17,5	0,71	0,01	0,03
SYQ_ECODH	*glnS*	Glutamine--tRNA ligase	33,755	63477,891	5,886	15		7526797	9372448	6,6	6,9	1,25	0,00	0,01
GLO2_ECODH	*gloB*	Hydroxyacylglutathione hydrolase	27,49	28434,379	5,434	4		276486	281576	11,0	13,8	1,02	0,85	0,88
B1X8D5_ECODH	*glpA*	sn-glycerol-3-phosphate dehydrogenase (Anaer	35,978	58958,254	6,196	15		5782436	1477129	7,2	12,9	0,26	0,00	0,00
GLPB_ECODH	*glpB*	Anaerobic glycerol-3-phosphate dehydrogenase	27,208	45357,238	5,749	7		2232671	421973	10,4	16,0	0,19	0,00	0,00
B1X8D7_ECODH	*glpC*	sn-glycerol-3-phosphate dehydrogenase (Anaer	26,768	44108,039	8,778	9		2226882	418821	6,2	11,1	0,19	0,00	0,00
B1X771_ECODH	*glpD*	sn-glycerol-3-phosphate dehydrogenase, aerobi	59,481	56750,543	6,964	21		24075162	28688793	5,6	10,1	1,19	0,02	0,05
GLPK_ECODH	*glpK*	Glycerol kinase	61,155	56230,766	5,365	23		77451726	55433871	3,6	6,6	0,72	0,00	0,00
B1X8D3_ECODH	*glpQ*	Periplasmic glycerophosphodiester phosphodies	52,514	40843,352	5,377	12		10322805	2098560	5,7	19,7	0,20	0,00	0,00
B1X768_ECODH	*glpR*	DNA-binding transcriptional repressor	8,73	28047,838	5,821	2		143237	165167	5,9	8,6	1,15	0,03	0,06
B1X8D4_ECODH	*glpT*	sn-glycerol-3-phosphate transporter	7,743	50310,387	8,684	3		2169930	622888	18,2	20,4	0,29	0,00	0,00
B1XB91_ECODH	*glpX*	Fructose-1,6-bisphosphatase	16,369	35852,309	5,325	5		436638	311581	6,9	5,3	0,71	0,00	0,00
B1X6Q1_ECODH	*gltA*	Citrate synthase	45,199	48014,992	6,213	14	metabolism	8134109	28835734	4,6	8,7	3,55	0,00	0,00
B1XHI7_ECODH	*gltB*	Glutamate synthase, large subunit	21,49	166709,88	6,265	23		745388	1925085	8,7	5,3	2,58	0,00	0,00
B1XHI8_ECODH	*gltD*	Glutamate synthase, 4Fe-4S protein, small subu	13,559	52015,273	5,537	4		240841	584825	12,0	6,3	2,43	0,00	0,00
B1X650_ECODH	*gltI*	Glutamate and aspartate transporter subunit, pe	10,265	33420,164	8,61	2		78548	119986	19,2	10,3	1,53	0,01	0,03
SYE_ECODH	*gltX*	Glutamate--tRNA ligase	32,484	53815,727	5,593	12		5652760	4612722	4,1	7,3	0,82	0,00	0,01
GLYA_ECODH	*glyA*	Serine hydroxymethyltransferase	39,089	45316,594	6,028	11		8110992	8456689	6,3	11,2	1,04	0,53	0,61
SYGB_ECODH	*glyS*	Glycine--tRNA ligase beta subunit	49,927	76812,945	5,291	25		9774754	10461210	5,6	5,6	1,07	0,15	0,22
GMHA_ECODH	*gmhA*	Phosphoheptose isomerase	36,458	20814,748	5,966	6		1810557	1729299	4,7	12,8	0,96	0,53	0,62
B1X6W6_ECODH	*gnd*	6-phosphogluconate dehydrogenase, decarboxy	62,821	51481,309	5,055	20		31560622	25200722	6,1	4,3	0,80	0,00	0,00
B1X8U6_ECODH	*gnsA*	Predicted regulator of phosphatidylethanolamine	40,351	6575,613	5,392	2		1589437	1437136	10,7	6,0	0,90	0,14	0,22
B1XF19_ECODH	*gnsB*	Uncharacterized protein	12,281	6547,669	8,963	1		86146	183080	54,0	32,7	2,13	0,04	0,08
B1X7V5_ECODH	*gor*	Glutathione oxidoreductase	18	48772,516	5,639	5		1098395	1275800	11,7	13,6	1,16	0,15	0,22
B1X732_ECODH	*gph*	Phosphoglycolate phosphatase	22,222	27389,166	4,578	3		419519	554683	12,0	16,4	1,32	0,03	0,07
GPMA_ECODH	*gpmA*	2,3-bisphosphoglycerate-dependent phosphogly	32,8	28556,404	5,851	7		14240771	13865382	7,5	5,9	0,97	0,59	0,66
B1X946_ECODH	*gpmI*	2,3-bisphosphoglycerate-independent phosphog	36,381	56193,887	5,141	11		7942175	5993130	5,9	14,2	0,75	0,01	0,02
GPPA_ECODH	*gppA*	Guanosine-5'-triphosphate,3'-diphosphate pyrop	6,275	54871,141	5,946	3		60890	92452	18,4	16,6	1,52	0,02	0,04
GPDA_ECODH	*gpsA*	Glycerol-3-phosphate dehydrogenase [NAD(P)+	41,298	36361,602	6,085	7		528453	727647	18,7	24,6	1,38	0,10	0,17
XGPT_ECODH	*gpt*	Xanthine phosphoribosyltransferase	42,763	16970,588	5,52	4		458157	584861	2,9	30,3	1,28	0,18	0,26
GRCA_ECODH	*grcA*	Autonomous glycyl radical cofactor	74,016	14284,189	5,087	9		16580981	4454353	4,4	13,0	0,27	0,00	0,00
B1XGZ1_ECODH	*greA*	Transcription elongation factor GreA	55,063	17640,957	4,712	7		2075949	1564773	9,0	12,2	0,75	0,01	0,03
CH60_ECODH	*groL*	60 kDa chaperonin	69,708	57328,848	4,845	24		71878789	161298918	7,9	13,2	2,24	0,00	0,00
CH10_ECODH	*groS*	10 kDa chaperonin	51,546	10386,952	5,147	4		20955487	41379423	22,3	19,4	1,97	0,00	0,01
GRPE_ECODH	*grpE*	Protein GrpE	29,949	21797,83	4,681	5		11356294	16811885	8,2	18,6	1,48	0,01	0,03
B1X9H7_ECODH	*grxB*	Glutaredoxin 2	47,442	24349,266	8,717	8		1187584	699678	6,7	23,2	0,59	0,00	0,01
B1X944_ECODH	*grxC*	Glutaredoxin 3	68,675	9137,494	6,707	4		1657836	1098483	16,1	6,4	0,66	0,00	0,01
B1XFW5_ECODH	*grxD*	Glutaredoxin	46,087	12878,761	4,75	4		5083433	5066834	5,0	16,1	1,00	0,97	0,97
GSH1_ECODH	*gshA*	Glutamate--cysteine ligase	16,409	58269,293	5,273	6		406350	659638	15,9	15,9	1,62	0,01	0,02
B1XFA7_ECODH	*gshB*	Glutathione synthetase	31,646	35560,898	5,109	7		1235538	1776882	7,2	5,5	1,44	0,00	0,00
B1XFR4_ECODH	*gsk*	Inosine/guanosine kinase	14,516	48448,781	5,502	4		200868	232368	15,5	11,2	1,16	0,19	0,28
B1XFE4_ECODH	*gss*	Fused glutathionylspermidine amidase glutathior	8,562	70531,961	5,13	4		256805	328635	12,9	24,6	1,28	0,13	0,20
B1XFU7_ECODH	*gst*	Glutathionine S-transferase	34,826	22868,369	5,849	5		611195	776199	9,3	16,8	1,27	0,04	0,08
GUAA_ECODH	*guaA*	GMP synthase [glutamine-hydrolyzing]	43,429	58679,223	5,239	16		9029129	12531436	2,1	10,6	1,39	0,00	0,00
B1XAY3_ECODH	*guaB*	Inosine-5'-monophosphate dehydrogenase	46,107	52022,453	6,016	12		7710779	13415310	6,5	5,3	1,74	0,00	0,00
GUAC_ECODH	*guaC*	GMP reductase	24,784	37383,668	6,103	4		819052	710567	33,6	23,9	0,87	0,55	0,63
B1XCN5_ECODH	*gutQ*	Arabinose 5-phosphate isomerase	20,249	34031,43	5,919	4		206208	196723	8,1	13,0	0,95	0,56	0,64
B1X8C5_ECODH	*gyrA*	DNA gyrase subunit A	27,314	96963,516	5,086	20		7962950	7517526	3,5	4,6	0,94	0,09	0,15
HCHA_ECODH	*hchA*	Molecular chaperone Hsp31 and glyoxalase 3	28,975	31190,463	5,628	6		3534679	1765568	8,2	11,7	0,50	0,00	0,00
B1X7W5_ECODH	*hdeA*	Acid stress chaperone HdeA	54,545	11857,629	5,056	2	acid stress response	534489	132068	33,2	32,3	0,25	0,00	0,01
B1X7W4_ECODH	*hdeB*	Acid stress chaperone HdeB	43,519	12042,821	5,727	3	acid stress response	554124	198786	14,6	21,8	0,36	0,00	0,00
B1X7W6_ECODH	*hdeD*	Acid-resistance membrane protein	6,316	20903,369	9,553	2	acid stress response	35149	16918	41,3	9,4	0,48	0,01	0,03
B1XF84_ECODH	*hdhA*	7-alpha-hydroxysteroid dehydrogenase, NAD-de	34,902	26778,59	5,216	6		905859	621062	5,5	8,9	0,69	0,00	0,00
B1XEW0_ECODH	*hemB*	Delta-aminolevulinic acid dehydratase	22,531	35624,793	5,25	5		1491537	1423042	22,0	16,3	0,95	0,75	0,80
HEM3_ECODH	*hemC*	Porphobilinogen deaminase	54,313	33851,836	5,312	10		625231	707426	13,1	10,6	1,13	0,19	0,28
DCUP_ECODH	*hemE*	Uroporphyrinogen decarboxylase	24,011	39248,117	5,882	6		1004480	1039184	10,7	30,9	1,03	0,84	0,88
B1XAL2_ECODH	*hemG*	Protoporphyrin oxidase, flavoprotein	24,862	21226,301	9,678	4		307344	264424	21,3	12,9	0,86	0,26	0,36
GSA_ECODH	*hemL*	Glutamate-1-semialdehyde 2,1-aminomutase	37,793	45366,098	4,733	11		6434240	6321621	8,8	7,0	0,98	0,76	0,81
B1XAM3_ECODH	*hemN*	Coproporphyrinogen-III oxidase	9,409	52729,043	5,7	3		144003	129708	4,0	23,1	0,90	0,40	0,49
B1XAH0_ECODH	*hemY*	Predicted protoheme IX synthesis protein	31,156	45245,023	8,525	8		2341316	1557966	15,5	33,1	0,67	0,09	0,15
B1XDS6_ECODH	*hflC*	Modulator of FtsH protease HflC	35,629	37649,883	6,299	8	degradation/folding	1405943	2984572	9,9	9,2	2,12	0,00	0,00
HFLD_ECODH	*hflD*	High frequency lysogenization protein HflD	15,493	22947,547	9,338	2		202583	186548	9,7	13,0	0,92	0,36	0,46
B1XDS5_ECODH	*hflK*	Modulator for HflB protease specific for phage la	39,618	45544,883	6,189	12	degradation/folding	4133618	11927057	11,5	8,1	2,89	0,00	0,00

B1XDS4_ECODH	*hflX*	GTPase HflX	38,028	48327,102	5,684	11	metabolism	750564	2489489	8,4	7,5	3,32	0,00	0,00
HFQ_ECODH	*hfq*	RNA-binding protein Hfq	21,569	11166,386	6,97	2		997738	1192996	17,6	14,4	1,20	0,17	0,25
B1XFP7_ECODH	*hha*	Haemolysin expression modulating protein	11,111	8678,061	8,787	1		14814	55704	68,8	42,3	3,76	0,01	0,03
B1XA13_ECODH	*hinT*	Purine nucleoside phosphoramidase	25,21	13241,277	5,731	2		842422	721495	22,4	9,7	0,86	0,30	0,40
B1X6V9_ECODH	*hisB*	Histidine biosynthesis bifunctional protein HisB	16,338	40277,965	5,763	5		281196	533308	21,5	3,1	1,90	0,00	0,01
HIS6_ECODH	*hisF*	Imidazole glycerol phosphate synthase subunit H	14,729	28454,453	5,034	3		103802	130023	9,9	10,2	1,25	0,02	0,05
HIS1_ECODH	*hisG*	ATP phosphoribosyltransferase	27,425	33366,715	5,471	4		246225	524718	20,3	17,1	2,13	0,00	0,01
B1X6W0_ECODH	*hisH*	Imidazole glycerol phosphate synthase subunit H	16,327	21652,857	5,335	2		74595	78155	16,2	9,1	1,05	0,64	0,71
B1X6W3_ECODH	*hisI*	Histidine biosynthesis bifunctional protein HisIE	17,241	22755,818	5,245	1		42160	173675	48,8	21,9	4,12	0,01	0,02
B1X920_ECODH	*hisJ*	Histidine/lysine/arginine/ornithine transporter sub	26,154	28483,377	5,465	4	transport	332016	787134	10,4	12,4	2,37	0,00	0,00
SYH_ECODH	*hisS*	Histidine--tRNA ligase	44,811	47029,441	5,647	15		5096946	5350946	6,4	5,6	1,05	0,30	0,40
HLDD_ECODH	*hldD*	ADP-L-glycero-D-manno-heptose-6-epimerase	47,742	34893,168	4,8	13		8060830	7294599	9,9	3,9	0,90	0,10	0,17
HLDE_ECODH	*hldE*	Bifunctional protein HldE	37,317	51050,617	5,294	12		2975409	2763507	9,0	10,2	0,93	0,32	0,42
B1XB27_ECODH	*hmp*	Flavohemoprotein	15,152	43867,656	5,484	6		378381	305658	5,0	4,1	0,81	0,00	0,00
B1XAS9_ECODH	*hns*	DNA-binding protein	54,015	15539,633	5,436	6		24184214	47222863	5,6	18,0	1,95	0,00	0,00
B1XHH8_ECODH	*hpf*	Ribosomal hibernation promoting factor	17,895	10750,249	6,5	1		39106	15907	7,7	20,4	0,41	0,00	0,00
B1XCA0_ECODH	*hpt*	Hypoxanthine phosphoribosyltransferase	44,944	20115,238	5,091	7		1190083	1143186	12,6	5,7	0,96	0,58	0,65
B1XDC6_ECODH	*hrpA*	ATP-dependent helicase	5,923	149027,92	7,887	6		394218	454125	8,5	15,9	1,15	0,16	0,23
HSCA_ECODH	*hscA*	Chaperone protein HscA	20,942	65652,438	4,983	8		1273386	1452258	6,5	6,2	1,14	0,03	0,06
HSCB_ECODH	*hscB*	Co-chaperone protein HscB	13,45	20137,709	5,048	2		394291	381321	11,3	17,6	0,97	0,76	0,81
B1XD94_ECODH	*hslJ*	Heat-inducible protein	27,143	15165,621	6,721	2		148594	291242	30,5	19,7	1,96	0,02	0,04
HSLO_ECODH	*hslO*	33 kDa chaperonin	29,11	32534,477	4,354	5		1146412	2709642	12,6	14,8	2,36	0,00	0,00
HSLU_ECODH	*hslU*	ATP-dependent protease ATPase subunit HslU	30,926	49593,805	5,239	10		5985088	12740042	3,8	13,8	2,13	0,00	0,00
B1XFR0_ECODH	*htpG*	Chaperone protein HtpG	58,333	71422,531	5,086	28		27381473	53678389	2,1	8,6	1,96	0,00	0,00
HTPX_ECODH	*htpX*	Protease HtpX	16,041	31923,312	6,605	3	degradation/folding	809019	8718814	8,4	7,1	10,78	0,00	0,00
B1XG60_ECODH	*htrG*	Predicted signal transduction protein (SH3 doma	14,078	23076,389	9,096	3		481823	528850	5,9	14,0	1,10	0,27	0,37
B1XC03_ECODH	*hupA*	HU, DNA-binding transcriptional regulator, alpha	57,778	9534,985	9,572	4		30576421	23411002	7,7	10,6	0,77	0,01	0,02
B1XFM8_ECODH	*hupB*	HU, DNA-binding transcriptional regulator, beta s	15,556	9225,579	9,694	1		985714	323236	33,1	18,2	0,33	0,00	0,01
B1XFF2_ECODH	*hybA*	Hydrogenase 2 4Fe-4S ferredoxin-type compone	9,756	36002,996	7,104	2		74901	15835	29,2	22,7	0,21	0,00	0,00
B1XFF0_ECODH	*hybC*	Hydrogenase 2, large subunit	18,519	62491,16	5,843	6		921428	213126	13,1	28,1	0,23	0,00	0,00
IBPA_ECODH	*ibpA*	Small heat shock protein IbpA	37,226	15773,711	5,574	5		923405	2477198	15,3	12,8	2,68	0,00	0,00
IBPB_ECODH	*ibpB*	Small heat shock protein IbpB	37,324	16093,197	5,189	4		364491	915186	19,2	18,4	2,51	0,00	0,00
B1XA46_ECODH	*icd*	Isocitrate dehydrogenase [NADP]	38,702	45784,762	5,163	12		18780688	28209921	10,7	8,0	1,50	0,00	0,00
ICIA_ECODH	*iciA*	Chromosome initiation inhibitor	8,081	33471,688	6,402	2		99231	147303	17,4	20,5	1,48	0,04	0,07
IHFA_ECODH	*ihfA*	Integration host factor subunit alpha	41,414	11353,932	9,342	4		2230837	1967101	6,7	12,6	0,88	0,14	0,21
IHFB_ECODH	*ihfB*	Integration host factor subunit beta	26,596	10651,144	9,344	2		786442	521122	13,6	21,4	0,66	0,02	0,04
SYI_ECODH	*ileS*	Isoleucine--tRNA ligase	44,456	104296,84	5,657	29		12400238	15474996	5,2	1,8	1,25	0,00	0,00
B1X9A4_ECODH	*ilvB*	Acetolactate synthase	16,192	60440,57	5,302	4	metabolism	107900	939859	22,6	8,9	8,71	0,00	0,00
ILVC_ECODH	*ilvC*	Ketol-acid reductoisomerase	10,998	54069,02	5,204	5	metabolism	96932	818756	6,7	6,1	8,45	0,00	0,00
B1X9Y7_ECODH	*ilvE*	Branched-chain amino-acid aminotransferase	14,887	34093,652	5,54	3		191533	502798	11,5	16,7	2,63	0,00	0,00
B1X9A3_ECODH	*ilvN*	Acetolactate synthase I, small subunit	10,417	11105,758	5,69	1	metabolism	14387	129534	42,5	36,4	9,00	0,00	0,00
B1XC55_ECODH	*imp*	LPS-assembly protein LptD	37,372	89671,148	4,936	20		4090624	2267381	5,2	5,7	0,55	0,00	0,00
B1X824_ECODH	*infA*	Translation initiation factor IF-1	27,778	8249,575	9,22	1		162569	137882	30,7	25,1	0,85	0,50	0,58
IF2_ECODH	*infB*	Translation initiation factor IF-2	31,573	97349,898	5,802	20		13352697	16185148	7,5	5,4	1,21	0,01	0,02
IRAP_ECODH	*iraP*	Anti-adapter protein IraP	31,395	9937,478	4,818	1		33265	14973	14,4	25,2	0,45	0,00	0,01
ISCR_ECODH	*iscR*	HTH-type transcriptional regulator IscR	29,012	17336,561	6,824	2		296800	556824	10,1	15,9	1,88	0,00	0,00
ISCS_ECODH	*iscS*	Cysteine desulfurase IscS	46,782	45089,5	5,943	15		9256794	13410910	9,2	6,8	1,45	0,00	0,00
B1XB04_ECODH	*iscU*	Scaffold protein	60,938	13848,59	4,815	5		2880071	3954802	4,7	9,5	1,37	0,00	0,00
B1XF09_ECODH	*ispA*	Geranyltranstransferase	24,08	32159,633	5,273	4		530637	582021	9,9	15,7	1,10	0,38	0,48
ISPF_ECODH	*ispF*	2-C-methyl-D-erythritol 2,4-cyclodiphosphate syr	19,497	16897,537	6,044	2		240887	243884	32,6	15,1	1,01	0,95	0,96
ISPG_ECODH	*ispG*	4-hydroxy-3-methylbut-2-en-1-yl diphosphate syı	42,742	40683,613	5,871	14		2177761	2852241	6,4	9,2	1,31	0,00	0,01
ISPH_ECODH	*ispH*	4-hydroxy-3-methylbut-2-enyl diphosphate reduc	13,608	34774,559	5,196	3		399029	434902	11,4	10,8	1,09	0,31	0,41
B1XD82_ECODH	*ivy*	Inhibitor of vertebrate C-lysozyme	35,032	16872,289	6,267	5		252085	146787	13,2	12,9	0,58	0,00	0,01
B1XGJ3_ECODH	*katE*	Catalase	17,928	84162,617	5,541	9		1264073	736435	13,5	12,6	0,58	0,00	0,00
KATG_ECODH	*katG*	Catalase-peroxidase	37,328	80023,812	5,139	20		8369889	10677340	5,7	8,4	1,28	0,00	0,01
B1X951_ECODH	*kbl*	Glycine C-acetyltransferase	33,668	43117,039	5,64	10		5273266	4879016	9,3	8,7	0,93	0,28	0,37
B1X8E4_ECODH	*kdgK*	Ketodeoxygluconokinase	16,505	33962,359	4,923	3		570285	685022	10,0	18,1	1,20	0,12	0,19
B1XH95_ECODH	*kdgR*	Predicted DNA-binding transcriptional regulator	38,403	30029,348	5,435	8		981038	850992	7,7	7,8	0,87	0,04	0,08
KDSA_ECODH	*kdsA*	2-dehydro-3-deoxyphosphooctonate aldolase	48,944	30832,693	6,32	11		6363419	5715166	11,0	6,8	0,90	0,15	0,23
KDSB_ECODH	*kdsB*	3-deoxy-manno-octulosonate cytidylyltransferase	35,081	27614,439	5,147	6		920407	1059401	9,7	10,3	1,15	0,10	0,16
B1XHH3_ECODH	*kdsC*	3-deoxy-D-manno-octulosonate 8-phosphate pho	29,787	19997,113	4,942	3		366882	388675	8,2	7,3	1,06	0,34	0,44
B1XHH2_ECODH	*kdsD*	Arabinose 5-phosphate isomerase	5,793	35196,086	6,219	2		100038	87402	13,3	12,8	0,87	0,19	0,28
B1X967_ECODH	*kdtA*	3-deoxy-D-manno-octulosonic-acid transferase (	6,118	47291,082	9,797	2		87220	69800	13,2	18,4	0,80	0,12	0,19
B1XFQ2_ECODH	*kefA*	Fused conserved protein mechanosensitive char	7,679	127215,28	8,045	6		235764	188493	14,2	13,5	0,80	0,07	0,12
B1XBJ2_ECODH	*lacI*	DNA-binding transcriptional repressor	45,278	38590,164	6,39	11		1719418	89417452	10,6	8,8	52,00	0,00	0,00
LAMB_ECODH	*lamB*	Maltoporin	37,892	49912,227	4,807	10		3407953	2085420	3,7	21,9	0,61	0,00	0,01
B1XD95_ECODH	*ldhA*	Fermentative D-lactate dehydrogenase, NAD-de	21,581	36534,793	5,285	5		893013	1521827	3,7	6,7	1,70	0,00	0,00
LEPA_ECODH	*lepA*	Elongation factor 4	33,556	66570,273	5,401	15		3585084	3622934	8,2	8,7	1,01	0,87	0,89
B1X637_ECODH	*leuS*	Leucine--tRNA ligase	34,767	97233,758	5,156	21		13253120	7490670	3,4	9,9	0,57	0,00	0,00
LEXA_ECODH	*lexA*	LexA repressor	11,386	22357,719	6,229	2		127443	93133	15,1	33,3	0,73	0,12	0,19
LGT_ECODH	*lgt*	Prolipoprotein diacylglyceryl transferase	20,962	33107,949	9,515	3		220032	426132	5,0	29,9	1,94	0,00	0,01
DNLJ_ECODH	*ligA*	DNA ligase	14,158	73606,07	5,385	9		777280	606322	9,2	6,4	0,78	0,00	0,01
B1X623_ECODH	*lipA*	Lipoyl synthase	19,315	36071,715	8,084	5		1921475	1610914	9,9	8,2	0,84	0,03	0,06
LLDD_ECODH	*lldD*	L-lactate dehydrogenase [cytochrome]	34,091	42728,191	6,332	9		3025801	2924055	14,2	5,8	0,97	0,67	0,74

LOLA_ECODH	*lolA*	Outer-membrane lipoprotein carrier protein	43,35	22497,055	6,278	6		547191	1084072	7,4	11,0	1,98	0,00	0,00
LOLB_ECODH	*lolB*	Outer-membrane lipoprotein LolB	46,377	23550,77	8,886	7		806114	857829	10,2	12,1	1,06	0,46	0,56
B1XA26_ECODH	*lolC*	Outer membrane-specific lipoprotein transporter	11,028	43263,762	6,043	3		157281	220966	14,3	11,8	1,40	0,01	0,03
B1XFM7_ECODH	*lon*	Lon protease	41,071	87438,125	6,012	22		6651655	12684317	9,8	3,7	1,91	0,00	0,00
B1XC91_ECODH	*lpd*	Dihydrolipoyl dehydrogenase	43,038	50688,492	5,789	15		41789445	56280065	11,6	10,0	1,35	0,01	0,02
LPLA_ECODH	*lplA*	Lipoate-protein ligase A	11,834	37925,883	5,708	2		182005	220791	16,8	24,7	1,21	0,24	0,33
B1XFY5_ECODH	*lpp*	Murein lipoprotein	33,333	8323,479	9,3	2		36947808	45818050	21,7	10,5	1,24	0,10	0,17
LPXA_ECODH	*lpxA*	Acyl-[acyl-carrier-protein]--UDP-N-acetylglucosa	17,557	28079,996	6,63	3		459346	422009	14,5	24,8	0,92	0,56	0,64
B1XD48_ECODH	*lpxD*	UDP-3-O-(3-hydroxymyristoyl)glucosamine N-ac	22,287	36038,484	6,073	4		808670	643289	9,4	22,4	0,80	0,14	0,22
B1X9G7_ECODH	*lpxL*	Lauryl-acyl carrier protein (ACP)-dependent acyl	11,438	35406,824	9,805	2		73419	153876	19,8	27,6	2,10	0,00	0,01
B1X901_ECODH	*lrhA*	DNA-binding transcriptional repressor of flagella	10,897	34593,828	5,238	2	metabolism	122327	295189	13,3	15,1	2,41	0,00	0,00
B1X829_ECODH	*lrp*	DNA-binding transcriptional dual regulator, leuci	26,22	18886,783	8,904	4		923182	1229711	4,7	9,7	1,33	0,00	0,01
B1X811_ECODH	*ltaE*	L-allo-threonine aldolase, PLP-dependent	16,216	36494,707	5,807	5		334103	417611	11,3	10,2	1,25	0,03	0,06
LUXS_ECODH	*luxS*	S-ribosylhomocysteine lyase	50,877	19416,189	5,181	6		3495988	5066611	9,6	8,6	1,45	0,00	0,00
B1XC23_ECODH	*lysC*	Aspartokinase	7,795	48531,73	5,025	2		42636	125182	17,9	7,1	2,94	0,00	0,00
B1XEH7_ECODH	*lysS*	Lysine--tRNA ligase	41,98	57603,422	5,106	14		5897358	7081666	8,1	10,6	1,20	0,03	0,06
B1XD10_ECODH	*lysU*	Lysine--tRNA ligase	47,327	57826,57	5,104	19		12151150	12422828	6,9	7,3	1,02	0,67	0,74
MAO1_ECODH	*maeA*	NAD-dependent malic enzyme	43,009	63197,328	5,188	16		3789475	3603864	5,4	8,7	0,95	0,37	0,47
B1XAD4_ECODH	*maeB*	Fused malic enzyme predicted oxidoreductase p	38,208	82417,383	5,338	20		7557969	7004895	5,0	7,1	0,93	0,13	0,21
B1XEY3_ECODH	*mak*	Manno(Fructo)kinase	11,258	32499,744	5,48	2		139695	137579	16,5	23,3	0,98	0,92	0,94
B1XC33_ECODH	*malE*	Maltose transporter subunit periplasmic-binding	37,626	43387,641	5,534	10		5070923	2097718	9,9	37,0	0,41	0,00	0,01
B1XC32_ECODH	*malF*	Maltose transporter subunit membrane compone	18,482	57013,402	8,477	5		397771	179538	13,7	17,9	0,45	0,00	0,00
B1XC34_ECODH	*malK*	Fused maltose transport subunit, ATP-binding co	15,094	40990,453	6,228	4		1041012	550210	6,1	19,6	0,53	0,00	0,00
B1XC36_ECODH	*malM*	Maltose regulon periplasmic protein	14,706	31943,5	7,72	3		1619708	611806	9,3	13,6	0,38	0,00	0,00
B1X763_ECODH	*malP*	Phosphorylase	33,124	90522,398	6,938	18		4169601	3745840	2,8	16,1	0,90	0,23	0,33
B1X762_ECODH	*malQ*	4-alpha-glucanotransferase (Amylomaltase)	10,519	78503,492	6,138	5		327543	263727	4,4	20,2	0,81	0,08	0,13
MALT_ECODH	*malT*	HTH-type transcriptional regulator MalT	5,66	103118,45	6,028	3		296051	262848	10,0	21,2	0,89	0,35	0,45
B1XF78_ECODH	*manA*	Mannose-6-phosphate isomerase	29,923	42849,949	5,29	8		2234063	1330155	6,5	6,2	0,60	0,00	0,00
B1XH84_ECODH	*manX*	Fused mannose-specific PTS enzymes: IIA com	51,084	35047,551	5,742	10		5952514	6555454	4,1	26,1	1,10	0,48	0,56
B1XH86_ECODH	*manZ*	Mannose-specific enzyme IID component of PTS	21,329	31302,873	9,171	5		1835094	2283536	14,6	19,6	1,24	0,11	0,18
B1XD37_ECODH	*map*	Methionine aminopeptidase	29,545	29330,799	5,641	4		1744471	2548116	22,9	17,9	1,46	0,05	0,10
B1XG33_ECODH	*mdaB*	NADPH quinone reductase	12,953	21890,906	5,845	2		44320	40478	10,3	28,7	0,91	0,55	0,63
MDH_ECODH	*mdh*	Malate dehydrogenase	66,987	32337,299	5,607	13		27639542	38466203	6,5	12,1	1,39	0,00	0,01
OPGD_ECODH	*mdoD*	Glucans biosynthesis protein D	15,064	62757,945	5,893	5	metabolism	160453	343866	21,5	22,2	2,14	0,00	0,01
OPGG_ECODH	*mdoG*	Glucans biosynthesis protein G	30,724	57912,484	6,7	13		3613184	3211112	7,8	5,7	0,89	0,05	0,10
B1X9G3_ECODH	*mdoH*	Glucans biosynthesis glucosyltransferase H	10,39	96953,43	8,871	6		554545	585355	10,3	6,1	1,06	0,41	0,51
MDTA_ECODH	*mdtA*	Multidrug resistance protein MdtA	6,506	44464,289	8,903	2		23266	42502	36,5	28,7	1,83	0,06	0,11
B1X7W8_ECODH	*mdtE*	Multidrug resistance efflux transporter	42,857	41190,57	5,73	9		393338	209852	10,2	23,3	0,53	0,00	0,01
B1X7W9_ECODH	*mdtF*	Multidrug transporter, RpoS-dependent	8,004	111517,17	5,326	5		70557	43244	27,9	24,9	0,61	0,05	0,10
MDTK_ECODH	*mdtK*	Multidrug resistance protein MdtK	6,565	49447,047	9,63	2		80689	48874	17,1	10,3	0,61	0,00	0,01
B1X8X6_ECODH	*menB*	Dihydroxynaphthoic acid synthetase	29,825	31633,08	5,994	7		1654212	1113856	5,6	14,1	0,67	0,00	0,01
MENC_ECODH	*menC*	o-succinylbenzoate synthase	10,625	35476,613	4,836	2		141462	98251	11,2	5,4	0,69	0,00	0,00
METE_ECODH	*metE*	5-methyltetrahydropteroyltriglutamate--homocyst	14,475	84673,539	5,609	8		105289	539471	39,2	9,2	5,12	0,00	0,00
SYM_ECODH	*metG*	Methionine--tRNA ligase	41,802	76254,734	5,565	21		7789794	7833281	5,5	2,9	1,01	0,87	0,89
B1XC18_ECODH	*metH*	Homocysteine-N5-methyltetrahydrofolate transm	16,87	135997,05	4,97	14		529604	1297308	7,5	5,4	2,45	0,00	0,00
METK_ECODH	*metK*	S-adenosylmethionine synthase	50,26	41951,625	5,096	13		5592313	3570893	16,1	4,2	0,64	0,00	0,01
B1XBA6_ECODH	*metL*	Fused aspartokinase II homoserine dehydrogena	7,901	88887,695	5,343	4		97858	81221	11,2	13,6	0,83	0,07	0,13
B1XD69_ECODH	*metN*	ATP-binding component of DL-methionine uptak	32,362	37788,371	5,947	6		336229	834201	1,7	12,7	2,48	0,00	0,00
B1XD67_ECODH	*metQ*	Lipoprotein	53,506	29431,65	5,127	8		1626926	3701241	18,0	12,2	2,27	0,00	0,00
B1XA24_ECODH	*mfd*	Transcription-repair-coupling factor	13,415	129982,73	5,787	13		2707625	3482192	3,3	15,4	1,29	0,01	0,04
B1X7N8_ECODH	*mglB*	Methyl-galactoside transporter subunit periplasm	34,337	35712,574	5,68	8		6766580	1310351	14,2	5,6	0,19	0,00	0,00
MGSA_ECODH	*mgsA*	Methylglyoxal synthase	19,737	16918,576	6,12	2		589560	550011	18,4	19,0	0,93	0,62	0,69
B1XEM3_ECODH	*mgtA*	Magnesium transporter	15,924	99466,484	5,635	11		888880	1244639	8,1	5,9	1,40	0,00	0,00
MIAA_ECODH	*miaA*	tRNA dimethylallyltransferase	18,354	35065,152	5,682	4	metabolism	183851	725199	12,9	3,2	3,94	0,00	0,00
MIAB_ECODH	*miaB*	tRNA-2-methylthio-N(6)-dimethylallyladenosine s	36,92	53662,957	5,197	14		4113812	4567626	5,5	2,4	1,11	0,01	0,03
B1XA62_ECODH	*minD*	Site-determining protein	44,444	29614,033	5,246	9		5580154	2019903	4,1	13,2	0,36	0,00	0,00
MINE_ECODH	*minE*	Cell division topological specificity factor	54,545	10234,906	5,147	3		934685	340594	7,6	9,2	0,36	0,00	0,00
B1XGP2_ECODH	*mipA*	Scaffolding protein for murein synthesizing mach	31,048	27830,986	5,501	5		3670085	1924913	12,5	11,1	0,52	0,00	0,00
B1XDM2_ECODH	*mltA*	Membrane-bound lytic murein transglycosylase	15,89	40410,609	9,041	3		482508	656628	9,7	10,4	1,36	0,01	0,02
B1XCM8_ECODH	*mltB*	Membrane-bound lytic murein transglycosylase I	21,33	40255,746	9,068	4		317407	378069	6,9	15,6	1,19	0,09	0,15
MNMC_ECODH	*mnmC*	tRNA 5-methylaminomethyl-2-thiouridine biosynt	6,737	74434,164	5,43	2		46024	41735	13,5	23,0	0,91	0,49	0,58
MNME_ECODH	*mnmE*	tRNA modification GTPase MnmE	9,692	49230,871	4,892	3		121346	122434	10,3	24,5	1,01	0,95	0,96
MNMG_ECODH	*mnmG*	tRNA uridine 5-carboxymethylaminomethyl modi	19,714	69521,258	6,195	8		774735	763998	6,7	5,9	0,99	0,77	0,81
B1X7B2_ECODH	*moaB*	Molybdopterin biosynthesis protein B	29,412	18665,094	5,727	4		1551415	703107	12,8	17,7	0,45	0,00	0,00
MOAC_ECODH	*moaC*	Cyclic pyranopterin monophosphate synthase ac	24,224	17467,236	6,593	3		453317	371524	11,6	5,2	0,82	0,02	0,04
B1X7B4_ECODH	*moaD*	Molybdopterin synthase, small subunit	37,037	8757,937	4,381	2		236372	123710	11,4	16,9	0,52	0,00	0,00
B1X7B5_ECODH	*moaE*	Molybdopterin synthase, large subunit	23,333	16981,055	5,256	2		479309	342669	9,1	12,3	0,71	0,01	0,02
B1X791_ECODH	*modE*	DNA-binding transcriptional dual regulator	27,099	28281,225	5,22	3		215666	222700	18,4	11,8	1,03	0,79	0,83
B1X7F7_ECODH	*moeA*	Molybdopterin biosynthesis protein	40,876	44067,273	5,026	8		1796226	2055412	4,7	6,7	1,14	0,02	0,04
B1XBD4_ECODH	*mog*	Predicted molybdochelatase	41,026	21222,354	4,971	6		740304	567453	10,6	5,9	0,77	0,00	0,01
B1XEL5_ECODH	*mpl*	UDP-N-acetylmuramate:L-alanyl-gamma-D-gluta	19,037	49874,117	5,531	6		785866	829038	8,7	12,1	1,05	0,51	0,59
B1XCF8_ECODH	*mppA*	Murein tripeptide (L-ala-gamma-D-glutamyl-mes	5,959	59900,207	8,445	2		224235	79328	8,0	30,9	0,35	0,00	0,01
B1X743_ECODH	*mrcA*	Fused penicillin-binding protein 1a: murein trans	9,882	93636,172	6,155	6		313149	451599	7,4	8,6	1,44	0,00	0,00
B1XCC4_ECODH	*mrcB*	Bifunctional glycosyl transferase and transpeptid	8,886	94292,555	9,1	5		313003	308715	10,7	10,7	0,99	0,86	0,89

B1XHM1_ECODH	*mreB*	Cell wall structural complex MreBCD, actin-like c	40,634	36952,402	5,192	11		6796024	8117608	11,1	7,2	1,19	0,04	0,08
B1X7K3_ECODH	*mrp*	Antiporter inner membrane protein	18,97	39938,078	5,854	4		683796	589140	7,1	5,2	0,86	0,02	0,04
B1X854_ECODH	*msbA*	Fused lipid transporter subunits of ABC superfar	15,979	64460,707	8,62	7		814459	751585	13,0	7,9	0,92	0,32	0,42
B1XEK7_ECODH	*mscS*	Mechanosensitive channel	22,028	30896,021	7,896	5		1138295	1857238	14,9	9,0	1,63	0,00	0,01
MSRB_ECODH	*msrB*	Peptide methionine sulfoxide reductase MsrB	37,226	15451,175	5,581	3		690558	598410	6,1	17,8	0,87	0,16	0,25
B1X9G5_ECODH	*msyB*	Uncharacterized protein	50	14259,198	3,587	3		156594	96550	27,2	23,3	0,62	0,03	0,06
B1X8L3_ECODH	*mtlA*	Fused mannitol-1-specific PTS enzymes: IIA comp	11,774	67972,258	6,047	4		238941	330571	19,1	23,0	1,38	0,08	0,14
MTLD_ECODH	*mtlD*	Mannitol-1-phosphate 5-dehydrogenase	25,131	41139,016	5,357	6		496198	657285	13,9	15,9	1,32	0,04	0,07
MTNN_ECODH	*mtnN*	5'-methylthioadenosine/S-adenosylhomocysteine	15,086	24353,967	5,092	2		416729	612177	31,3	14,4	1,47	0,06	0,11
MUG_ECODH	*mug*	G/U mismatch-specific DNA glycosylase	35,119	18673,393	9,169	4		132001	151000	6,0	24,7	1,14	0,34	0,44
MUKB_ECODH	*mukB*	Chromosome partition protein MukB	15,007	170230,17	5,237	18		2379563	4626268	7,4	40,3	1,94	0,02	0,05
B1X8M7_ECODH	*mukE*	Chromosome partition protein MukE	32	25984,404	4,974	5		611704	638578	8,1	19,8	1,04	0,71	0,77
MUKF_ECODH	*mukF*	Chromosome partition protein MukF	9,773	50578,91	4,752	3		184186	171132	19,5	8,1	0,93	0,53	0,61
B1XBX9_ECODH	*murB*	UDP-N-acetylenolpyruvoylglucosamine reductas	11,404	37851,031	5,779	3		193427	157186	17,9	26,4	0,81	0,22	0,32
MURC_ECODH	*murC*	UDP-N-acetylmuramate--L-alanine ligase	12,22	53625,961	5,529	3		225879	324683	18,1	9,9	1,44	0,01	0,03
B1XC65_ECODH	*murD*	UDP-N-acetylmuramoylalanine--D-glutamate liga	8,447	46973,621	5,153	3		153651	145077	7,6	12,1	0,94	0,46	0,56
B1XC62_ECODH	*murE*	UDP-N-acetylmuramoyl-L-alanyl-D-glutamate--2	16,162	53343,598	5,425	6		541420	476808	13,9	13,8	0,88	0,24	0,34
B1XC63_ECODH	*murF*	UDP-N-acetylmuramoyl-tripeptide--D-alanyl-D-al	4,867	47447,148	5,253	2		122241	116613	14,1	19,8	0,95	0,70	0,76
MUTS_ECODH	*mutS*	DNA mismatch repair protein MutS	15,592	95246,898	5,394	9		645472	630753	7,3	12,4	0,98	0,77	0,81
B1XC76_ECODH	*mutT*	Nucleoside triphosphate pyrophosphohydrolase,	10,078	14927,073	5,012	1		29640	44046	19,4	21,2	1,49	0,03	0,06
NADE_ECODH	*nadE*	NH(3)-dependent NAD(+) synthetase	44,727	30636,834	5,413	11		2225754	3333809	5,8	11,2	1,50	0,00	0,00
B1XFK0_ECODH	*nadR*	NadR transcriptional repressor / ribosylnicotinam	15,61	47345,832	5,423	4		244108	306165	17,0	14,4	1,25	0,08	0,14
B1X6L0_ECODH	*nagA*	N-acetylglucosamine-6-phosphate deacetylase	8,115	40949,098	5,645	2		308686	280051	13,4	21,0	0,91	0,48	0,56
NAGB_ECODH	*nagB*	Glucosamine-6-phosphate deaminase	8,647	29774,203	6,411	2		577102	331493	11,8	11,7	0,57	0,00	0,00
B1X6K8_ECODH	*nagD*	UMP phosphatase	35,6	27189,133	5,067	5		801032	1155815	5,6	4,0	1,44	0,00	0,00
B1X6L2_ECODH	*nagE*	Fused N-acetyl glucosamine specific PTS enzyn	17,13	68346,891	5,784	7		2522252	1549210	11,7	12,2	0,61	0,00	0,01
NAGZ_ECODH	*nagZ*	Beta-hexosaminidase	8,504	37594,73	5,863	2		65382	57695	32,6	18,6	0,88	0,53	0,61
NANA_ECODH	*nanA*	N-acetylneuraminate lyase	44,108	32593,469	5,61	9		13245153	6399536	9,6	17,4	0,48	0,00	0,00
NANE_ECODH	*nanE*	Putative N-acetylmannosamine-6-phosphate 2-e	22,707	24073,66	4,892	3		1000090	885116	13,8	12,7	0,89	0,24	0,33
NANK_ECODH	*nanK*	N-acetylmannosamine kinase	32,302	29643,865	5,626	7		780866	826008	5,8	8,9	1,06	0,34	0,44
NAPA_ECODH	*napA*	Periplasmic nitrate reductase	17,874	93042,023	8,234	9		1218982	219284	5,8	11,8	0,18	0,00	0,00
B1XAR8_ECODH	*narG*	Nitrate reductase 1, alpha subunit	38,893	140489,39	6,052	33		17465338	2366993	9,3	10,7	0,14	0,00	0,00
B1XAR9_ECODH	*narH*	Nitrate reductase 1, beta (Fe-S) subunit	38,672	58066,41	6,362	14		5043912	648972	8,3	36,6	0,13	0,00	0,00
B1XAS1_ECODH	*narI*	Nitrate reductase 1, gamma (Cytochrome b(NR)	10,222	25497,365	9,774	2		177890	8644	11,4	36,3	0,05	0,00	0,00
B1XAS0_ECODH	*narJ*	Molybdenum-cofactor-assembly chaperone subu	36,441	26448,865	4,757	5		598401	54876	10,4	37,1	0,09	0,00	0,00
B1XAR7_ECODH	*narK*	Nitrate/nitrite transporter	7,127	49693,434	9,679	2		1878792	273476	12,6	6,4	0,15	0,00	0,00
B1XAR5_ECODH	*narL*	DNA-binding response regulator in two-compone	12,037	23926,791	5,727	3		273107	255201	11,2	6,4	0,93	0,32	0,42
B1X889_ECODH	*narP*	DNA-binding response regulator in two-compone	18,14	23574,975	4,816	3		309355	304821	20,6	16,4	0,99	0,92	0,94
B1XA19_ECODH	*ndh*	Respiratory NADH dehydrogenase 2/cupric redu	42,166	47358,672	8,961	12		5203853	7332249	8,5	6,2	1,41	0,00	0,00
NDK_ECODH	*ndk*	Nucleoside diphosphate kinase	64,336	15463,444	5,54	7		5048926	4012040	12,0	2,9	0,79	0,01	0,02
B1XFW1_ECODH	*nemA*	N-ethylmaleimide reductase, FMN-linked	15,89	39516,414	5,803	4		151593	192933	24,0	37,6	1,27	0,29	0,39
B1X5X3_ECODH	*nfnB*	Dihydropteridine reductase, NAD(P)H-dependen	37,327	23905,191	5,802	6		2242933	2621013	7,8	10,4	1,17	0,06	0,11
B1X7Z2_ECODH	*nfsA*	Nitroreductase A, NADPH-dependent, FMN-depe	31,667	26800,686	6,444	6		1052466	1272674	23,6	23,9	1,21	0,30	0,41
NFUA_ECODH	*nfuA*	Fe/S biogenesis protein NfuA	26,702	20997,705	4,524	4		3199824	4607446	15,4	6,1	1,44	0,01	0,02
B1X7T4_ECODH	*nikA*	Nickel transporter subunit periplasmic-binding cc	13,931	58719,02	5,745	5		397946	74895	13,9	25,1	0,19	0,00	0,00
B1X713_ECODH	*nirB*	Nitrite reductase, large subunit, NAD(P)H-binding	12,869	93121,32	5,79	7		1524584	258592	4,2	10,8	0,17	0,00	0,00
B1X714_ECODH	*nirD*	Nitrite reductase, NAD(P)H-binding, small subun	13,889	12284,003	5,074	1		317652	35196	7,0	14,6	0,11	0,00	0,00
B1XAE9_ECODH	*nlpB*	Outer membrane protein assembly factor BamC	56,395	36842,43	5,337	12		2707307	3040549	12,1	5,4	1,12	0,12	0,20
B1XCR8_ECODH	*nlpD*	Predicted outer membrane lipoprotein	17,15	40149	9,518	3		409387	405417	13,5	9,8	0,99	0,91	0,93
B1XD62_ECODH	*nlpE*	Lipoprotein involved with copper homeostasis an	70,763	25844,287	5	11		3091263	774087916	7,1	18,0	250,41	0,00	0,00
B1X8C8_ECODH	*nrdA*	Ribonucleoside-diphosphate reductase	31,012	85775,281	5,791	18		6917555	5679692	3,0	4,8	0,82	0,00	0,00
B1X8C9_ECODH	*nrdB*	Ribonucleoside diphosphate reductase 1, beta s	21,277	43517,273	4,691	7		2120658	1660992	11,4	14,3	0,78	0,03	0,07
B1XEL9_ECODH	*nrdD*	Anaerobic ribonucleoside-triphosphate reductase	12,079	80022,562	6,376	6		693274	218042	10,5	12,2	0,31	0,00	0,00
NRDR_ECODH	*nrdR*	Transcriptional repressor NrdR	16,107	17228,895	7,664	2		224062	231223	25,5	17,4	1,03	0,85	0,88
NUDC_ECODH	*nudC*	NADH pyrophosphatase	23,346	29688,939	5,518	4		181713	202354	4,0	17,0	1,11	0,24	0,34
B1X744_ECODH	*nudE*	ADP-ribose diphosphatase	13,978	21153,166	4,847	1	metabolism	42646	101014	20,6	16,2	2,37	0,00	0,00
NUDK_ECODH	*nudK*	GDP-mannose pyrophosphatase NudK	14,136	21748,664	4,879	1		8677	35673	32,7	64,2	4,11	0,01	0,03
NUOA_ECODH	*nuoA*	NADH-quinone oxidoreductase subunit A	17,687	16457,328	9,874	2		844903	586636	14,5	19,4	0,69	0,02	0,05
NUOB_ECODH	*nuoB*	NADH-quinone oxidoreductase subunit B	30,455	25055,816	5,587	7		2379764	1400389	8,4	13,2	0,59	0,00	0,00
NUOCD_ECODH	*nuoC*	NADH-quinone oxidoreductase subunit C/D	44,833	68694,648	5,98	21		9056337	5464324	5,8	3,4	0,60	0,00	0,00
B1X8Z7_ECODH	*nuoE*	NADH:ubiquinone oxidoreductase, chain E	19,277	18590,051	5,402	2		768682	553654	10,0	23,2	0,72	0,06	0,11
B1X8Z6_ECODH	*nuoF*	NADH:ubiquinone oxidoreductase, chain F	32,36	49292,453	6,441	10		2813394	2287850	14,5	9,6	0,81	0,07	0,12
B1X8Z5_ECODH	*nuoG*	NADH:ubiquinone oxidoreductase, chain G	35,714	100543,56	5,845	22		8981863	5019168	5,8	5,9	0,56	0,00	0,00
NUOH_ECODH	*nuoH*	NADH-quinone oxidoreductase subunit H	9,538	36219,152	6,898	3		825798	582839	17,4	22,2	0,71	0,07	0,12
B1X8Z3_ECODH	*nuoI*	NADH-quinone oxidoreductase subunit I	15	20537,75	5,396	3		478394	271130	11,4	21,4	0,57	0,00	0,01
NUOK_ECODH	*nuoK*	NADH-quinone oxidoreductase subunit K	12	10845,019	8,381	1		283364	89711	19,3	22,6	0,32	0,00	0,00
B1X8Z0_ECODH	*nuoL*	NADH:ubiquinone oxidoreductase, membrane su	4,568	66438,078	8,283	2		654536	376977	22,9	9,5	0,58	0,01	0,02
B1X8Y9_ECODH	*nuoM*	NADH:ubiquinone oxidoreductase, membrane su	3,143	56524,543	8,62	2		306885	225869	20,3	9,7	0,74	0,03	0,06
B1X9R4_ECODH	*nupC*	Nucleoside (Except guanosine) transporter	10,5	43475,754	8,5	3		332252	323202	32,3	21,2	0,97	0,88	0,91
B1XGY1_ECODH	*nusA*	Transcription termination/antitermination L factor	44,242	54870,922	4,527	15		7332866	7724688	6,9	11,1	1,05	0,46	0,55
NUSB_ECODH	*nusB*	N utilization substance protein B homolog	37,41	15689,056	6,601	4		1757353	1633907	3,7	14,9	0,93	0,38	0,48
B1XBY4_ECODH	*nusG*	Transcription termination/antitermination protein	47,514	20531,514	6,339	6		3295984	3657350	4,6	18,4	1,11	0,28	0,38
OBG_ECODH	*obg*	GTPase Obg	25,641	43285,863	4,743	7		1721199	2261581	12,0	4,5	1,31	0,01	0,02
B1X8R1_ECODH	*ompA*	Outer membrane protein A (3aII*Gd)	61,272	37200,762	5,989	14		117862205	138940726	8,9	4,8	1,18	0,02	0,04

B1X8B1_ECODH	*ompC*	Outer membrane porin protein C	80,109	40368,117	4,581	19		41404866	73389393	16,3	24,3	1,77	0,01	0,02
B1X8N3_ECODH	*ompF*	Outer membrane porin 1a (IabF)	30,663	39333,309	4,76	8		3711984	1830790	14,1	6,2	0,49	0,00	0,00
B1X752_ECODH	*ompR*	DNA-binding response regulator in two-compone	46,862	27353,617	6,038	7		2087056	2577434	10,6	6,6	1,23	0,01	0,04
B1XGF9_ECODH	*ompT*	DLP12 prophage outer membrane protease VII (	66,562	35562,309	5,755	15		9553423	6780241	8,4	3,2	0,71	0,00	0,00
B1XBK5_ECODH	*ompW*	Outer membrane protein W	10,849	22927,83	6,029	2		1090365	604474	13,9	34,5	0,55	0,02	0,05
B1X7E4_ECODH	*ompX*	Outer membrane protein	46,784	18602,617	6,561	6		22553305	25455966	6,9	12,5	1,13	0,15	0,23
B1XAT3_ECODH	*oppA*	Oligopeptide transporter periplasmic subunit	50,092	60948,004	6,053	17		9501322	7663499	8,2	11,1	0,81	0,02	0,05
B1XAT4_ECODH	*oppB*	Oligopeptide transporter subunit membrane com	8,17	33443,199	9,783	2		95408	100731	17,8	27,7	1,06	0,77	0,82
B1XAT6_ECODH	*oppD*	Oligopeptide transporter subunit ATP-binding co	19,288	37188,43	5,777	4		286261	284697	5,4	10,0	0,99	0,93	0,94
B1XAT7_ECODH	*oppF*	ATP-binding subunit of oligopeptide ABC transpo	37,725	37181,547	7,647	11		1050577	1003690	14,3	10,8	0,96	0,63	0,70
B1XDR6_ECODH	*orn*	Oligoribonuclease	16,575	20815,623	4,963	3		331221	263772	3,8	4,5	0,80	0,00	0,00
B1XE73_ECODH	*osmC*	Osmotically inducible, stress-inducible membran	40,559	15088,142	5,57	4		2086352	592478	4,3	9,3	0,28	0,00	0,00
B1XFI5_ECODH	*osmY*	Periplasmic protein	17,91	21073,707	6,315	3		171276	339435	19,0	35,8	1,98	0,01	0,03
B1XBX2_ECODH	*oxyR*	DNA-binding transcriptional dual regulator	16,393	34275,93	5,959	3		373488	488663	18,3	9,5	1,31	0,04	0,07
B1X6S2_ECODH	*pal*	Peptidoglycan-associated outer membrane lipop	32,37	18824,186	6,292	4		1890773	4947984	25,8	25,0	2,62	0,00	0,01
PANB_ECODH	*panB*	3-methyl-2-oxobutanoate hydroxymethyltransfer	46,591	28237,439	5,148	7		1687580	3011235	1,4	23,0	1,78	0,00	0,01
PANC_ECODH	*panC*	Pantothenate synthetase	9,187	31597,672	5,915	2		445392	497088	21,8	14,8	1,12	0,44	0,54
B1XFH0_ECODH	*parC*	DNA topoisomerase 4 subunit A	19,149	83831,266	6,243	8		882540	1084188	3,5	6,7	1,23	0,00	0,01
B1XG35_ECODH	*parE*	DNA topoisomerase 4 subunit B	17,302	70243,914	5,436	8		787285	962122	23,5	8,3	1,22	0,14	0,21
PCKA_ECODH	*pckA*	Phosphoenolpyruvate carboxykinase [ATP]	40,37	59643,48	5,459	15		17360214	10170696	5,2	5,3	0,59	0,00	0,00
PIMT_ECODH	*pcm*	Protein-L-isoaspartate O-methyltransferase	33,173	23257,775	6,528	5		237444	196630	5,4	7,4	0,83	0,01	0,02
B1XCB8_ECODH	*pcnB*	Poly(A) polymerase I	22,151	53870,91	9,669	8		807265	876684	7,6	7,5	1,09	0,17	0,26
B1XC88_ECODH	*pdhR*	DNA-binding transcriptional dual regulator	22,441	29425,471	6,036	4		243095	351084	8,4	16,8	1,44	0,01	0,02
PDXA_ECODH	*pdxA*	4-hydroxythreonine-4-phosphate dehydrogenase	12,766	35113,777	5,869	2		106515	110176	17,8	15,5	1,03	0,79	0,83
PDXB_ECODH	*pdxB*	Erythronate-4-phosphate dehydrogenase	36,508	41367,648	6,227	10		1571922	1480459	7,8	4,2	0,94	0,22	0,31
PDXH_ECODH	*pdxH*	Pyridoxine/pyridoxamine 5'-phosphate oxidase	29,817	25545,152	9,182	5		554655	438486	12,0	8,5	0,79	0,02	0,05
B1XB38_ECODH	*pdxJ*	Pyridoxine 5'-phosphate synthase	15,226	26384,287	5,61	3		352999	274881	14,3	19,8	0,78	0,08	0,14
PDXK_ECODH	*pdxK*	Pyridoxine kinase	20,141	30847,4	5,141	4		262623	109004	25,5	16,7	0,42	0,00	0,01
B1XFU8_ECODH	*pdxY*	Pyridoxamine kinase	19,861	31322,197	6,041	4		324944	298680	18,8	10,4	0,92	0,47	0,56
AMPA_ECODH	*pepA*	Probable cytosol aminopeptidase	28,032	54879,809	6,815	11		1643993	2644089	13,9	6,8	1,61	0,00	0,00
PEPB_ECODH	*pepB*	Peptidase B	53,396	46180,168	5,602	17		4347923	5760424	1,6	3,2	1,32	0,00	0,00
B1XDY0_ECODH	*pepD*	Aminoacyl-histidine dipeptidase (Peptidase D)	26,392	52915,406	5,204	10		5169246	5531684	7,8	2,8	1,07	0,15	0,22
B1X8N6_ECODH	*pepN*	Aminopeptidase N	18,736	98918,938	5,142	14		5335769	5772997	13,8	10,3	1,08	0,39	0,49
B1XEJ4_ECODH	*pepP*	Proline aminopeptidase P II	25,17	49815,434	5,245	8		967898	874319	9,3	12,3	0,90	0,24	0,33
PEPQ_ECODH	*pepQ*	Xaa-Pro dipeptidase	32,731	50176,176	5,602	11		3527535	4267241	4,0	4,3	1,21	0,00	0,00
PEPT_ECODH	*pepT*	Peptidase T	19,608	44923,426	5,36	7		1263556	587054	4,3	8,2	0,46	0,00	0,00
K6PF_ECODH	*pfkA*	6-phosphofructokinase	21,25	34842,039	5,47	6		15577497	10142200	11,4	12,2	0,65	0,00	0,01
B1XGI4_ECODH	*pfkB*	Phosphofructokinase	32,362	32455,992	5,252	7		1113787	1073028	7,8	6,9	0,96	0,50	0,59
B1X842_ECODH	*pflA*	Pyruvate formate lyase activating enzyme 1	11,382	28204,291	6,002	3		153239	181076	37,3	16,6	1,18	0,45	0,54
B1X843_ECODH	*pflB*	Pyruvate formate lyase I	59,605	85357,203	5,69	29		70304609	35955438	9,0	5,1	0,51	0,00	0,00
G6PI_ECODH	*pgi*	Glucose-6-phosphate isomerase	37,705	61529,766	5,853	16		10557213	10524016	3,5	3,8	1,00	0,91	0,93
B1XEK9_ECODH	*pgk*	Phosphoglycerate kinase	59,948	41118,219	5,079	15		46055121	40486148	5,0	7,9	0,88	0,03	0,06
6PGL_ECODH	*pgl*	6-phosphogluconolactonase	34,743	36307,637	5,06	7		3134457	3362731	15,4	13,0	1,07	0,53	0,61
B1X6M1_ECODH	*pgm*	Phosphoglucomutase	40,293	58360,949	5,429	15		3469062	3200903	5,7	3,8	0,92	0,06	0,11
SYFA_ECODH	*pheS*	Phenylalanine--tRNA ligase alpha subunit	36,697	36831,805	5,792	10		4256088	4253015	4,0	5,1	1,00	0,98	0,98
B1XG20_ECODH	*pheT*	Phenylalanine--tRNA ligase beta subunit	44,403	87378,109	5,169	26		12908867	14289161	1,2	1,7	1,11	0,00	0,00
B1XCZ1_ECODH	*phnA*	Conserved protein	25,225	12345,049	4,971	2		224617	164542	27,7	28,0	0,73	0,17	0,25
B1XCZ0_ECODH	*phnB*	Conserved protein	10,884	16171,316	5,037	1		37455	18226	16,4	20,7	0,49	0,00	0,01
B1XA40_ECODH	*phoP*	DNA-binding response regulator in two-compone	43,049	25535,217	5,098	7		1210074	847865	8,5	13,7	0,70	0,00	0,01
B1X7U8_ECODH	*pitA*	Phosphate transporter, low-affinity	8,016	53389,027	9,533	3		403122	400140	27,1	13,5	0,99	0,96	0,97
B1XAI6_ECODH	*pldA*	Outer membrane phospholipase A	39,1	33163,133	5,154	9		618931	639623	14,8	3,0	1,03	0,68	0,74
PLSB_ECODH	*plsB*	Glycerol-3-phosphate acyltransferase	36,307	91381,445	8,512	20		2187624	2507903	8,0	5,1	1,15	0,03	0,06
B1XFG9_ECODH	*plsC*	1-acyl-sn-glycerol-3-phosphate acyltransferase	7,755	27453,158	9,655	2		140152	116211	15,8	15,2	0,83	0,13	0,20
B1XEL7_ECODH	*pmbA*	Predicted peptidase required for the maturation a	33,556	48369,637	5,401	10		2090594	2370964	7,7	6,2	1,13	0,04	0,09
B1X8X3_ECODH	*pmrD*	Polymyxin resistance protein B	15,909	9870,51	8,641	1		85605	81403	13,1	14,4	0,95	0,62	0,69
PNCB_ECODH	*pncB*	Nicotinate phosphoribosyltransferase	17,5	45897,285	6,208	6		273929	315077	13,2	13,8	1,15	0,19	0,28
PNP_ECODH	*pnp*	Polyribonucleotide nucleotidyltransferase	41,069	77100,977	5,114	20		13143388	15086389	8,3	6,1	1,15	0,04	0,07
B1XF68_ECODH	*pntA*	NAD(P) transhydrogenase subunit alpha	11,961	54623,441	5,645	5		1110482	876215	14,9	6,5	0,79	0,02	0,05
B1XF67_ECODH	*pntB*	NAD(P) transhydrogenase subunit beta	20,13	48723,027	5,722	6		1114896	880661	6,3	10,2	0,79	0,01	0,02
B1XAM0_ECODH	*polA*	DNA polymerase I	27,909	103118,13	5,395	18		2287821	1590857	5,4	8,9	0,70	0,00	0,00
B1XA36_ECODH	*potA*	Polyamine transporter subunit ATP-binding com	28,307	43028,215	5,19	7		1379368	1278711	4,3	9,3	0,93	0,19	0,28
B1XA35_ECODH	*potB*	Polyamine transporter subunit membrane compo	7,273	31061,982	9,437	2		100264	120233	27,4	30,2	1,20	0,37	0,47
B1XA33_ECODH	*potD*	Putrescine-binding periplasmic protein	22,414	38867,082	5,242	6		707094	552811	24,0	9,8	0,78	0,11	0,17
B1X812_ECODH	*poxB*	Pyruvate dehydrogenase (Pyruvate oxidase), thi	26,049	62011,387	5,861	10		839473	526253	7,3	11,2	0,63	0,00	0,00
B1XDX5_ECODH	*ppa*	Inorganic pyrophosphatase	16,477	19703,557	5,027	3		4414666	5504047	33,1	17,0	1,25	0,25	0,35
CAPP_ECODH	*ppc*	Phosphoenolpyruvate carboxylase	36,693	99062,609	5,517	25		3627899	3910334	7,5	6,4	1,08	0,18	0,26
B1X711_ECODH	*ppiA*	Peptidyl-prolyl cis-trans isomerase	27,368	20431,322	8,804	3	degradation/folding	336405	1020639	13,7	13,9	3,03	0,00	0,00
B1XGC3_ECODH	*ppiB*	Peptidyl-prolyl cis-trans isomerase	21,341	18153,471	5,514	3		2971103	3065233	9,9	22,1	1,03	0,81	0,85
B1X9Z1_ECODH	*ppiC*	Peptidyl-prolyl cis-trans isomerase C (Rotamase	21,505	10232,021	9,23	2		157522	72917	12,0	23,0	0,46	0,00	0,00
B1XFM9_ECODH	*ppiD*	Peptidyl-prolyl cis-trans isomerase (Rotamase D	34,831	68149,859	4,939	14		4421334	4597323	11,8	6,2	1,04	0,57	0,65
B1XAX6_ECODH	*ppk*	Polyphosphate kinase	20,203	80431,508	8,96	10		439193	407927	9,8	22,9	0,93	0,55	0,63
B1XG09_ECODH	*pps*	Phosphoenolpyruvate synthase	15,909	87435,078	4,934	11		1626461	892353	6,3	4,9	0,55	0,00	0,00
PSRP_ECODH	*ppsR*	Phosphoenolpyruvate synthase regulatory protei	12,635	31210,867	5,987	3		211555	96671	12,3	34,9	0,46	0,01	0,02
B1XAX7_ECODH	*ppx*	Exopolyphosphatase	10,916	58135,969	6,646	4		291790	305491	10,4	11,2	1,05	0,58	0,66

B1XH98_ECODH	*prc*	Carboxy-terminal protease for penicillin-binding p	17,742	76663,094	6,19	9		1139784	932290	7,5	6,1	0,82	0,01	0,02
RF1_ECODH	*prfA*	Peptide chain release factor 1	19,444	40517,301	5,146	5		355451	315501	16,1	7,0	0,89	0,20	0,29
RF2_ECODH	*prfB*	Peptide chain release factor 2	28,493	41250,727	4,643	7		4759150	5084142	9,1	5,4	1,07	0,25	0,34
RF3_ECODH	*prfC*	Peptide chain release factor 3	18,147	59574,082	5,655	7		1715892	2029428	13,9	9,2	1,18	0,10	0,16
B1X7V3_ECODH	*prlC*	Oligopeptidase A	39,265	77167,234	5,151	20		5002220	9729202	5,5	7,2	1,94	0,00	0,00
B1X9K3_ECODH	*prmB*	50S ribosomal protein L3 glutamine methyltransf	15,161	35001,73	4,623	4		523053	823822	11,0	14,4	1,58	0,00	0,01
PROA_ECODH	*proA*	Gamma-glutamyl phosphate reductase	32,374	44630,055	5,418	10		1247561	1518496	8,7	5,0	1,22	0,01	0,02
PROB_ECODH	*proB*	Glutamate 5-kinase	24,251	39056,523	6,073	6		850950	1039199	4,4	11,5	1,22	0,02	0,05
B1XEX5_ECODH	*proC*	Pyrroline-5-carboxylate reductase	21,933	28144,877	5,638	4		550892	276607	14,1	14,4	0,50	0,00	0,00
B1XCZ4_ECODH	*proP*	Proline/glycine betaine transporter	5,4	54845,816	6,763	2		92353	121140	25,5	31,4	1,31	0,23	0,32
PROQ_ECODH	*proQ*	ProP effector	47,414	25892,518	9,664	9		3290284	3381024	9,9	7,5	1,03	0,67	0,73
SYP_ECODH	*proS*	Proline--tRNA ligase	53,147	63692,602	5,122	21		15180981	17940947	4,2	1,6	1,18	0,00	0,00
B1XCL5_ECODH	*proX*	Glycine betaine transporter subunit periplasmic-b	26,97	36022,547	5,903	5		1672522	1212302	16,8	9,5	0,72	0,02	0,04
B1XAP8_ECODH	*prsA*	Ribose-phosphate pyrophosphokinase	42,857	34218,27	5,23	9		8700154	6479547	9,2	13,4	0,74	0,01	0,03
PSD_ECODH	*psd*	Phosphatidylserine decarboxylase proenzyme	32,609	35934,414	5,51	6	metabolism	1465011	3120320	13,8	11,5	2,13	0,00	0,00
B1XEX3_ECODH	*psiF*	Conserved protein	13,208	11686,53	9,784	1		1145	2772	120,7	72,0	2,42	0,44	0,53
B1XCD3_ECODH	*pspA*	Regulatory protein for phage-shock-protein oper	19,82	25492,939	5,385	3		189134	526186	24,2	37,3	2,78	0,01	0,02
B1XCD7_ECODH	*pspE*	Thiosulfate:cyanide sulfurtransferase (Rhodanes	18,269	11475,313	7,864	1		24266	8530	31,4	38,1	0,35	0,01	0,02
B1XBR2_ECODH	*pssA*	Phosphatidylserine synthase (CDP-diacylglycero	34,368	52801,793	9,06	11		1916500	1931246	6,3	9,8	1,01	0,90	0,92
B1X9V4_ECODH	*pstB*	Phosphate transporter subunit ATP-binding com	15,564	29027,289	6,125	2		196127	453335	54,6	33,5	2,31	0,06	0,11
B1X9V7_ECODH	*pstS*	Phosphate-binding protein PstS	6,069	37023,922	8,386	2		105254	126495	23,4	18,1	1,20	0,27	0,37
B1X908_ECODH	*pta*	Phosphate acetyltransferase	41,457	77172,086	5,284	20		15409859	25000542	3,7	2,8	1,62	0,00	0,00
B1XDM7_ECODH	*ptrA*	Protease III	9,148	107708,48	5,782	7		205723	320633	1,1	8,6	1,56	0,00	0,00
B1XA11_ECODH	*ptsG*	Fused glucose-specific PTS enzymes: IIBcompo	20,545	50676,551	8,765	7		2450790	3106075	5,8	30,5	1,27	0,15	0,23
B1XA86_ECODH	*ptsH*	Phosphohistidinoprotein-hexose phosphotransfe	15,294	9119,371	5,646	1		221870	148319	41,3	22,8	0,67	0,16	0,24
B1XA87_ECODH	*ptsI*	Phosphoenolpyruvate-protein phosphotransferas	46,957	63561,836	4,776	17		17930060	24529945	5,6	12,2	1,37	0,00	0,01
B1XHH9_ECODH	*ptsN*	Sugar-specific enzyme IIA component of PTS	26,38	17959,568	5,568	3		395882	241215	4,8	23,2	0,61	0,00	0,01
PURA_ECODH	*purA*	Adenylosuccinate synthetase	46,528	47344,949	5,316	14		17032532	13440205	8,9	8,3	0,79	0,01	0,02
B1XA41_ECODH	*purB*	Adenylosuccinate lyase	32,237	51542,805	5,675	11		3086773	2420086	8,2	9,5	0,78	0,01	0,02
PUR7_ECODH	*purC*	Phosphoribosylaminoimidazole-succinocarboxan	46,835	26994,998	5,072	8		2024168	2026806	10,5	8,4	1,00	0,99	0,99
B1XC08_ECODH	*purD*	Phosphoribosylamine--glycine ligase	18,182	45940,359	4,957	6		427584	413311	7,3	1,4	0,97	0,40	0,49
B1XGC1_ECODH	*purE*	N5-carboxyaminoimidazole ribonucleotide mutas	23,669	17780,322	6,038	2		73630	85711	26,3	28,9	1,16	0,48	0,57
B1X923_ECODH	*purF*	Amidophosphoribosyltransferase	14,653	56488,055	5,325	5		316815	383628	15,0	18,3	1,21	0,15	0,22
PUR9_ECODH	*purH*	Bifunctional purine biosynthesis protein PurH	34,405	57329,207	5,526	13		1226762	1330036	0,9	10,7	1,08	0,20	0,29
B1XB32_ECODH	*purL*	Phosphoribosylformylglycinamidine synthase	20,849	141402,7	5,229	15		1230600	1427357	2,5	5,8	1,16	0,00	0,01
B1XAS4_ECODH	*purU*	Formyltetrahydrofolate hydrolase	23,929	31934,717	6,496	4		1910106	1310065	90,4	9,9	0,69	0,48	0,56
B1X9D5_ECODH	*putA*	Multifunctional proline dehydrogenase, delta-1-p	24,848	143815,16	5,691	21		2927172	2157478	9,1	5,1	0,74	0,00	0,01
B1XHC2_ECODH	*pykA*	Pyruvate kinase	38,333	51357,145	6,239	13		5792844	3490817	4,1	7,9	0,60	0,00	0,00
B1XFY4_ECODH	*pykF*	Pyruvate kinase	50,851	50729,422	5,77	18		18087688	13068995	7,7	2,8	0,72	0,00	0,00
PYRB_ECODH	*pyrB*	Aspartate carbamoyltransferase	35,37	34427,359	6,116	7	metabolism	582300	1723300	14,3	22,3	2,96	0,00	0,00
PYRC_ECODH	*pyrC*	Dihydroorotase	41,379	38827,383	5,771	8		1954888	1740302	12,1	3,7	0,89	0,12	0,19
PYRD_ECODH	*pyrD*	Dihydroorotate dehydrogenase (quinone)	19,345	36774,52	7,661	5		419778	235723	11,3	9,7	0,56	0,00	0,00
PYRE_ECODH	*pyrE*	Orotate phosphoribosyltransferase	13,615	23566,998	5,333	3		55255	33768	99,5	26,7	0,61	0,31	0,41
PYRF_ECODH	*pyrF*	Orotidine 5'-phosphate decarboxylase	37,551	26350,244	5,813	6		1108103	967116	7,6	15,1	0,87	0,15	0,23
PYRG_ECODH	*pyrG*	CTP synthase	26,422	60374,148	5,633	14		7928710	7309550	4,3	6,1	0,92	0,07	0,12
B1XD40_ECODH	*pyrH*	Uridylate kinase	19,087	25970,258	6,851	2		65029	168252	28,5	42,3	2,59	0,02	0,04
PYRI_ECODH	*pyrI*	Aspartate carbamoyltransferase regulatory chair	35,294	17120,631	6,895	4	metabolism	359136	744392	12,1	13,3	2,07	0,00	0,00
B1XCT9_ECODH	*qor*	Quinone oxidoreductase, NADPH-dependent	20,183	35172,188	8,351	4		233401	237387	9,0	13,3	1,02	0,84	0,88
QUEA_ECODH	*queA*	S-adenosylmethionine:tRNA ribosyltransferase-is	15,73	39430,844	5,089	3		574489	692007	16,3	37,0	1,20	0,40	0,49
QUEC_ECODH	*queC*	7-cyano-7-deazaguanine synthase	15,152	25514,172	5,682	3		358886	146687	59,8	20,3	0,41	0,02	0,05
QUEF_ECODH	*queF*	NADPH-dependent 7-cyano-7-deazaguanine rec	17,021	32587,508	5,726	5		234366	276824	10,7	15,6	1,18	0,14	0,22
RBFA_ECODH	*rbfA*	Ribosome-binding factor A	37,594	15154,482	5,932	4		1077891	1234610	11,7	21,2	1,15	0,33	0,43
B1X9X6_ECODH	*rbsA*	Fused D-ribose transporter subunits of ABC sup	15,369	55041,609	5,837	6		178225	181585	5,1	8,5	1,02	0,71	0,77
B1X9X8_ECODH	*rbsB*	D-ribose transporter subunit periplasmic-binding	61,824	30950,48	6,849	11		5483777	2876269	15,4	8,4	0,52	0,00	0,00
B1X9X7_ECODH	*rbsC*	D-ribose transporter subunit membrane compon	13,707	33451,824	9,811	2		329142	299335	11,6	13,2	0,91	0,34	0,44
RBSD_ECODH	*rbsD*	D-ribose pyranase	23,022	15292,5	5,929	2		306806	224679	7,9	17,8	0,73	0,02	0,04
B1X9X9_ECODH	*rbsK*	Ribokinase	33,657	32290,518	4,987	6		544738	435515	10,2	12,6	0,80	0,04	0,07
B1X8B3_ECODH	*rcsB*	DNA-binding response regulator in two-compone	16,667	23670,617	6,85	2		587711	628721	29,4	7,1	1,07	0,70	0,76
B1X8B2_ECODH	*rcsD*	Phosphotransfer intermediate protein in two-com	6,966	100372,17	5,056	5		182244	196022	12,4	12,3	1,08	0,44	0,53
B1XD66_ECODH	*rcsF*	Predicted outer membrane protein, signal	54,478	14163,468	9,352	4		752063	1131568	26,2	10,0	1,50	0,02	0,05
RDGC_ECODH	*rdgC*	Recombination-associated protein RdgC	34,983	33993,324	5,273	8		760525	938673	5,8	4,9	1,23	0,00	0,01
B1XEH9_ECODH	*recJ*	SsDNA exonuclease, 5'--> 3'-specific	5,546	63388,832	5,388	2		146400	224730	19,5	14,4	1,54	0,01	0,03
RECR_ECODH	*recR*	Recombination protein RecR	24,876	21963	5,133	4		155811	170294	3,1	13,5	1,09	0,25	0,34
B1X9Z2_ECODH	*rep*	DNA helicase and single-stranded DNA-depende	5,349	77024,195	6,769	2		66499	45361	17,4	26,9	0,68	0,05	0,09
B1X954_ECODH	*rfaF*	ADP-heptose:LPS heptosyltransferase II	14,08	39042,133	7,148	3		180728	229176	9,7	11,3	1,27	0,02	0,05
B1X6X5_ECODH	*rfbA*	Glucose-1-phosphate thymidylyltransferase	38,225	32693,557	5,386	9		2755186	1390465	14,7	4,0	0,50	0,00	0,00
B1X6X7_ECODH	*rfbB*	dTDP-glucose 4,6-dehydratase	29,64	40558,332	5,475	8		2198932	1532433	10,5	10,8	0,70	0,00	0,01
B1X6X4_ECODH	*rfbC*	dTDP-4-deoxyrhamnose-3,5-epimerase	11,351	21270,111	5,476	2		327884	182520	8,0	33,9	0,56	0,02	0,04
B1X6X6_ECODH	*rfbD*	dTDP-4-dehydrorhamnose reductase subunit, N.	11,037	32694,137	5,543	3		475325	354274	26,1	21,3	0,75	0,14	0,22
B1XAG2_ECODH	*rffA*	dTDP-4-keto-6-deoxy-D-glucose aminotransfera	31,383	41901,031	5,759	7		665084	729463	7,1	17,7	1,10	0,37	0,47
B1XAF8_ECODH	*rffD*	UDP-N-acetyl-D-mannosamine dehydrogenase	10,238	45838,734	5,223	2		244700	394503	52,6	25,3	1,61	0,17	0,25
B1XAF7_ECODH	*rffE*	UDP-N-acetylglucosamine 2-epimerase	14,362	42244,594	5,92	3		253235	264938	9,4	6,7	1,05	0,47	0,56
B1XAF9_ECODH	*rffG*	dTDP-glucose 4,6-dehydratase	21,408	39753,848	5,628	5		593170	414213	10,0	11,3	0,70	0,00	0,01
B1XAG0_ECODH	*rffH*	Glucose-1-phosphate thymidylyltransferase	7,167	32734,406	5,317	2		102257	80954	9,3	11,2	0,79	0,02	0,04

RHLB_ECODH	*rhlB*	ATP-dependent RNA helicase RhlB	29,216	47125,918	7,29	9		1231780	1773642	10,6	1,4	1,44	0,00	0,00
B1X7C7_ECODH	*rhlE*	ATP-dependent RNA helicase RhlE	8,59	49989,316	10,06	3		864493	995427	23,9	12,9	1,15	0,32	0,42
B1X9Z7_ECODH	*rho*	Transcription termination factor Rho	55,131	47004,211	6,751	18		13544094	14465231	3,8	7,7	1,07	0,18	0,27
RIBA_ECODH	*ribA*	GTP cyclohydrolase-2	12,755	21835,979	5,596	3		157336	174974	14,1	17,8	1,11	0,38	0,47
RIBB_ECODH	*ribB*	3,4-dihydroxy-2-butanone 4-phosphate synthase	15,668	23353,467	4,898	3		1134658	641103	9,2	6,9	0,57	0,00	0,00
B1XFX2_ECODH	*ribC*	Riboflavin synthase, alpha subunit	34,742	23444,896	5,642	6		1073484	931991	11,1	14,8	0,87	0,17	0,25
B1XF02_ECODH	*ribD*	Riboflavin biosynthesis protein RibD	14,714	40338,312	7,219	4		493399	420815	14,8	8,5	0,85	0,11	0,18
B1XBF0_ECODH	*ribF*	Bifunctional riboflavin kinase and FAD synthetas	22,364	34734,266	9,336	4		304553	246632	9,6	11,5	0,81	0,03	0,06
RISB_ECODH	*ribH*	6,7-dimethyl-8-ribityllumazine synthase	48,077	16156,511	5,155	5		4356575	4527849	14,2	8,1	1,04	0,66	0,72
B1X646_ECODH	*rihA*	Pyrimidine-specific ribonucleoside hydrolase Rih	8,039	33822,922	4,836	2		418068	266470	15,3	5,5	0,64	0,00	0,01
RIHC_ECODH	*rihC*	Non-specific ribonucleoside hydrolase RihC	21,053	32560,77	5,23	3	metabolism	180215	382293	30,1	41,1	2,12	0,02	0,05
RIMM_ECODH	*rimM*	Ribosome maturation factor RimM	43,956	20605,434	4,608	5		1874458	2000326	8,9	10,0	1,07	0,37	0,47
RIMO_ECODH	*rimO*	Ribosomal protein S12 methylthiotransferase Rir	18,367	49581,793	5,305	7		961022	1246565	4,5	7,3	1,30	0,00	0,00
RIMP_ECODH	*rimP*	Ribosome maturation factor RimP	12	16650,975	4,597	2		206234	187818	19,1	22,6	0,91	0,57	0,65
B1XDT1_ECODH	*rlmB*	23S rRNA (guanosine-2'-O-)-methyltransferase I	11,111	26556,629	6,174	2		231485	188649	2,4	9,3	0,81	0,01	0,02
RLME_ECODH	*rlmE*	Ribosomal RNA large subunit methyltransferase	34,45	23335,076	9,435	4		458604	1275292	5,0	13,9	2,78	0,00	0,00
RLMF_ECODH	*rlmF*	Ribosomal RNA large subunit methyltransferase	24,026	34225,922	9,297	4		250260	188163	15,0	21,2	0,75	0,06	0,11
RLMG_ECODH	*rlmG*	Ribosomal RNA large subunit methyltransferase	12,963	42331,449	6,318	3		162296	168757	4,5	11,7	1,04	0,54	0,62
RLMH_ECODH	*rlmH1*	Ribosomal RNA large subunit methyltransferase	14,839	17341,111	8,709	2		95805	125760	24,4	2,9	1,31	0,07	0,12
RLMI_ECODH	*rlmI*	Ribosomal RNA large subunit methyltransferase	14,394	44356,922	7,602	4		618760	511799	7,4	4,5	0,83	0,01	0,02
RLMKL_ECODH	*rlmL*	Ribosomal RNA large subunit methyltransferase	16,382	78854,102	8,964	10		734700	753499	8,7	7,9	1,03	0,68	0,75
RLMM_ECODH	*rlmM*	Ribosomal RNA large subunit methyltransferase	4,918	41905,199	7,038	2		115309	146613	13,4	25,5	1,27	0,14	0,22
RLMN_ECODH	*rlmN*	Dual-specificity RNA methyltransferase RlmN	12,5	43085,531	6,562	4		743327	765547	12,2	4,9	1,03	0,67	0,74
B1X628_ECODH	*rlpA*	Minor lipoprotein	16,022	37527,996	5,515	2		506182	1010414	9,7	17,4	2,00	0,00	0,00
B1X636_ECODH	*rlpB*	LPS-assembly lipoprotein LptE	33,161	21356,531	8,73	5		608219	456975	16,6	17,6	0,75	0,05	0,10
B1XBL8_ECODH	*rluB*	Pseudouridine synthase	21,649	32711,385	10,03	6		894983	883737	6,3	6,5	0,99	0,79	0,83
B1X9J9_ECODH	*rluC*	Pseudouridine synthase	15,047	36026,574	9,85	5		339266	396429	30,2	14,5	1,17	0,39	0,48
B1X608_ECODH	*rna*	Ribonuclease I	11,567	29617,588	8,863	2		123934	214718	25,5	16,6	1,73	0,01	0,03
RNB_ECODH	*rnb*	Exoribonuclease 2	36,801	72490,781	5,402	17		3773601	2171028	4,1	7,1	0,58	0,00	0,00
RNC_ECODH	*rnc*	Ribonuclease 3	19,027	25550,043	6,404	5		654657	824793	10,1	15,4	1,26	0,05	0,10
B1X9J7_ECODH	*rne*	Ribonuclease E	28,087	118196,73	5,476	18		6379687	6126084	9,4	6,9	0,96	0,52	0,60
B1X607_ECODH	*rnk*	Regulator of nucleoside diphosphate kinase	48,529	14927,063	4,483	3		565329	368720	9,7	11,1	0,65	0,00	0,00
RNPA_ECODH	*rnpA*	Ribonuclease P protein component	22,689	13789,198	11,82	2		273953	343204	18,3	14,4	1,25	0,10	0,17
B1XDT0_ECODH	*rnr*	Ribonuclease R	18,696	92109,195	8,777	12		868030	1381907	21,6	16,3	1,59	0,01	0,03
B1XFW3_ECODH	*rnt*	Ribonuclease T	11,163	23522,695	5,187	2		134493	139283	14,7	21,8	1,04	0,80	0,84
RODZ_ECODH	*rodZ*	Cytoskeleton protein RodZ	19,585	36191,668	5,585	4		1359394	1911391	8,1	14,5	1,41	0,01	0,02
B1XD58_ECODH	*rof*	Modulator of Rho-dependent transcription termin	20,238	9479,644	4,628	1		153116	136885	7,7	18,0	0,89	0,29	0,39
B1X733_ECODH	*rpe*	Ribulose-phosphate 3-epimerase	19,111	24554,254	5,127	3		447075	482362	13,2	13,6	1,08	0,47	0,56
RPIA_ECODH	*rpiA*	Ribose-5-phosphate isomerase A	39,269	22860,395	5,204	5		2863507	3102454	16,2	25,2	1,08	0,61	0,68
RL1_ECODH	*rplA*	50S ribosomal protein L1	51,709	24729,635	9,644	9		64228539	74828804	7,6	6,2	1,17	0,02	0,05
RL2_ECODH	*rplB*	50S ribosomal protein L2	49,084	29860,443	10,93	11		43964925	54620684	14,9	12,3	1,24	0,06	0,11
RL3_ECODH	*rplC*	50S ribosomal protein L3	43,541	22243,516	9,905	7		37954226	41943543	15,7	12,0	1,11	0,36	0,46
RL4_ECODH	*rplD*	50S ribosomal protein L4	47,264	22086,529	9,724	8		37411476	42607857	9,3	6,7	1,14	0,06	0,12
RL5_ECODH	*rplE*	50S ribosomal protein L5	55,307	20301,572	9,491	8		39074845	38300344	4,0	16,9	0,98	0,82	0,85
RL6_ECODH	*rplF*	50S ribosomal protein L6	71,186	18903,783	9,71	9		44319846	51507497	12,0	6,5	1,16	0,08	0,14
RL9_ECODH	*rplI*	50S ribosomal protein L9	61,074	15769,064	6,165	9		50988485	56058965	11,0	3,6	1,10	0,14	0,22
RL10_ECODH	*rplJ*	50S ribosomal protein L10	50,909	17711,588	9,038	6		55896250	57621055	8,8	13,1	1,03	0,71	0,77
RL11_ECODH	*rplK*	50S ribosomal protein L11	59,155	14875,379	9,641	6		43922745	54689853	18,2	10,8	1,25	0,09	0,15
RL7_ECODH	*rplL*	50S ribosomal protein L7/L12	43,802	12295,196	4,603	4		25739150	22153182	8,4	26,3	0,86	0,36	0,46
RL13_ECODH	*rplM*	50S ribosomal protein L13	46,479	16018,543	9,905	5		28773198	35704017	9,7	10,8	1,24	0,02	0,05
RL14_ECODH	*rplN*	50S ribosomal protein L14	43,089	13541,023	10,43	4		24679780	29525396	4,6	11,0	1,20	0,02	0,05
RL15_ECODH	*rplO*	50S ribosomal protein L15	40,278	14980,424	11,19	5		39498758	40153923	12,1	11,4	1,02	0,85	0,88
RL16_ECODH	*rplP*	50S ribosomal protein L16	32,353	15281,202	11,23	3		34402625	34448145	11,4	14,4	1,00	0,99	0,99
RL17_ECODH	*rplQ*	50S ribosomal protein L17	25,984	14364,586	11,05	3		22908959	26972212	10,4	10,7	1,18	0,07	0,12
RL19_ECODH	*rplS*	50S ribosomal protein L19	47,826	13133,235	10,62	5		33850823	36015908	9,5	17,3	1,06	0,55	0,63
RL20_ECODH	*rplT*	50S ribosomal protein L20	22,881	13496,957	11,47	3		15058102	16278554	9,7	11,8	1,08	0,34	0,44
RL21_ECODH	*rplU*	50S ribosomal protein L21	51,456	11564,349	9,846	4		19469356	25226198	14,4	7,8	1,30	0,02	0,04
RL22_ECODH	*rplV*	50S ribosomal protein L22	56,364	12226,292	10,23	5		22803080	20018820	14,3	12,9	0,88	0,23	0,33
B1X6G9_ECODH	*rplW*	50S ribosomal protein L23	12	11199,123	9,935	1		7603	6036	33,3	56,1	0,79	0,62	0,69
RL24_ECODH	*rplX*	50S ribosomal protein L24	36,538	11316,215	10,21	3		9594030	8520785	11,3	12,2	0,89	0,21	0,30
RL25_ECODH	*rplY*	50S ribosomal protein L25	56,383	10693,44	9,599	5		13011180	11875245	6,5	18,0	0,91	0,38	0,48
RL27_ECODH	*rpmA*	50S ribosomal protein L27	29,412	9124,472	10,59	2		228775	286300	7,0	18,7	1,25	0,06	0,11
RL28_ECODH	*rpmB*	50S ribosomal protein L28	21,795	9006,487	11,42	2		4181870	3811344	9,7	29,4	0,91	0,54	0,62
RL29_ECODH	*rpmC*	50S ribosomal protein L29	46,032	7273,449	9,984	2		30284618	31495842	5,4	15,4	1,04	0,67	0,73
RL30_ECODH	*rpmD*	50S ribosomal protein L30	33,898	6541,79	10,96	2		6426706	7692862	20,1	18,7	1,20	0,21	0,30
RL31_ECODH	*rpmE*	50S ribosomal protein L31	51,429	7871,062	9,461	3		11712955	18105403	11,1	18,9	1,55	0,01	0,03
RL32_ECODH	*rpmF*	50S ribosomal protein L32	26,316	6446,383	11,03	1		2627072	2079041	22,6	24,3	0,79	0,22	0,31
RL33_ECODH	*rpmG*	50S ribosomal protein L33	38,182	6371,585	10,25	2		4239245	5138162	16,4	10,8	1,21	0,10	0,17
RL34_ECODH	*rpmH*	50S ribosomal protein L34	17,391	5380,389	13	1		1896784	2276023	18,1	26,6	1,20	0,32	0,42
B1X6E7_ECODH	*rpoA*	DNA-directed RNA polymerase subunit alpha	51,976	36511,723	4,982	13		20783490	29864031	4,6	6,9	1,44	0,00	0,00
RPOB_ECODH	*rpoB*	DNA-directed RNA polymerase subunit beta	48,137	150632,34	5,148	43		54385226	64804003	2,3	3,1	1,19	0,00	0,00
RPOC_ECODH	*rpoC*	DNA-directed RNA polymerase subunit beta'	43,852	155160,25	6,667	44		45947836	48157464	8,7	5,7	1,05	0,40	0,50
B1XG72_ECODH	*rpoD*	RNA polymerase sigma factor RpoD	19,739	70263,281	4,69	10		1732538	2276275	14,8	13,7	1,31	0,03	0,07
B1XBQ0_ECODH	*rpoE*	RNA polymerase sigma factor	17,277	21695,738	5,382	2		123236	153282	32,2	21,7	1,24	0,31	0,42

B1XHH7_ECODH	*rpoN*	RNA polymerase sigma-54 factor	11,74	53989,785	4,627	4		194446	250754	6,9	16,4	1,29	0,03	0,07
B1XCR7_ECODH	*rpoS*	RNA polymerase sigma factor RpoS	12,424	37971,859	4,888	3		161979	236113	10,1	36,3	1,46	0,11	0,17
RPOZ_ECODH	*rpoZ*	DNA-directed RNA polymerase subunit omega	59,341	10236,573	4,87	4		2205921	2453302	3,6	10,8	1,11	0,12	0,19
B1X851_ECODH	*rpsA*	30S ribosomal protein S1	42,908	61158,074	4,892	17		50209639	52682316	7,0	6,7	1,05	0,36	0,46
RS2_ECODH	*rpsB*	30S ribosomal protein S2	70,539	26743,641	6,613	12		40152851	45341036	4,2	8,4	1,13	0,05	0,09
RS4_ECODH	*rpsD*	30S ribosomal protein S4	46,117	23469,094	10,05	8		45939708	56766201	8,3	7,2	1,24	0,01	0,02
B1X6F5_ECODH	*rpsE*	30S ribosomal protein S5	62,275	17603,379	10,11	7		73024084	76798367	11,5	5,7	1,05	0,46	0,55
RS6_ECODH	*rpsF*	30S ribosomal protein S6	67,176	15187,034	5,258	7		4084834	10915967	58,1	39,3	2,67	0,03	0,06
RS7_ECODH	*rpsG*	30S ribosomal protein S7	50,279	20019,092	10,37	7		62180848	71930049	12,5	10,1	1,16	0,12	0,19
RS8_ECODH	*rpsH*	30S ribosomal protein S8	47,692	14126,548	9,441	5		1494267	1348691	5,4	11,6	0,90	0,17	0,25
RS9_ECODH	*rpsI*	30S ribosomal protein S9	46,154	14856,199	10,94	6		26693006	23767406	8,1	15,5	0,89	0,23	0,32
RS10_ECODH	*rpsJ*	30S ribosomal protein S10	48,544	11735,587	9,681	5		20073888	20947245	9,8	3,2	1,04	0,45	0,55
RS11_ECODH	*rpsK*	30S ribosomal protein S11	27,132	13844,926	11,33	3		19873662	26214290	18,0	4,0	1,32	0,02	0,04
B1X6F0_ECODH	*rpsM*	30S ribosomal protein S13	61,864	13099,385	10,78	7		20150831	26367385	7,4	8,3	1,31	0,00	0,01
RS14_ECODH	*rpsN*	30S ribosomal protein S14	19,802	11580,483	11,16	2		369332	461954	34,8	48,2	1,25	0,44	0,54
RS15_ECODH	*rpsO*	30S ribosomal protein S15	15,73	10268,765	10,4	2		1614962	4251617	71,8	36,8	2,63	0,04	0,07
RS16_ECODH	*rpsP*	30S ribosomal protein S16	45,122	9190,556	10,54	3		10426181	12250039	12,6	15,7	1,17	0,17	0,26
RS18_ECODH	*rpsR*	30S ribosomal protein S18	29,333	8986,432	10,6	2		13843561	11853479	12,6	32,6	0,86	0,43	0,53
RS19_ECODH	*rpsS*	30S ribosomal protein S19	55,435	10430,286	10,52	4		3205338	7055731	38,5	21,4	2,20	0,01	0,02
RS20_ECODH	*rpsT*	30S ribosomal protein S20	24,138	9684,388	11,18	2		21840670	23805731	9,8	9,0	1,09	0,23	0,33
RS21_ECODH	*rpsU*	30S ribosomal protein S21	39,437	8499,957	11,15	3		5737306	9550672	45,3	36,7	1,66	0,12	0,19
RRAA_ECODH	*rraA*	Regulator of ribonuclease activity A	39,752	17360,365	4,071	4		609472	899123	11,4	19,1	1,48	0,01	0,03
B1XBP9_ECODH	*rseA*	Anti-sigma factor	12,963	24321,375	5,111	1		94341	110627	46,0	25,9	1,17	0,57	0,64
B1XD45_ECODH	*rseP*	Inner membrane zinc RIP metalloprotease	9,111	49071,324	6,628	3		161723	129420	10,4	20,3	0,80	0,11	0,17
RSGA_ECODH	*rsgA*	Putative ribosome biogenesis GTPase RsgA	12,286	39193,305	5,594	3		426203	412622	7,3	6,6	0,97	0,53	0,61
RSMA_ECODH	*rsmA*	Ribosomal RNA small subunit methyltransferase	27,106	30420,139	7,822	5		448642	657482	21,8	21,9	1,47	0,05	0,09
RSMB_ECODH	*rsmB*	Ribosomal RNA small subunit methyltransferase	33,566	48347,512	7,165	8		776497	742955	24,7	6,1	0,96	0,75	0,80
RSMC_ECODH	*rsmC*	Ribosomal RNA small subunit methyltransferase	10,204	37624,73	5,999	3		289721	290712	6,3	20,7	1,00	0,97	0,98
RSMF_ECODH	*rsmF*	Ribosomal RNA small subunit methyltransferase	8,559	53227,547	5,441	2		93870	103696	8,6	16,7	1,10	0,33	0,43
RSMG_ECODH	*rsmG*	Ribosomal RNA small subunit methyltransferase	10,628	23431,119	6,062	1		35576	27284	13,8	9,7	0,77	0,02	0,05
RSMH_ECODH	*rsmH*	Ribosomal RNA small subunit methyltransferase	18,53	34877,816	6,067	4		542936	490700	6,9	9,0	0,90	0,12	0,19
B1XF73_ECODH	*rstA*	DNA-binding response regulator in two-compone	18,595	27048,248	5,418	4		153194	172250	12,0	17,1	1,12	0,32	0,42
B1X881_ECODH	*rsuA*	Pseudouridine synthase	12,554	25865,303	5,747	2		277809	304295	13,3	10,4	1,10	0,33	0,43
RSXC_ECODH	*rsxC*	Electron transport complex subunit RsxC	6,351	80171,547	8,884	3		54311	58539	24,8	17,0	1,08	0,62	0,69
B1XBP5_ECODH	*sapA*	Predicted antimicrobial peptide transporter subu	5,667	61564,898	6,847	2		125212	118558	7,9	15,2	0,95	0,54	0,62
B1X6U7_ECODH	*sbcB*	Exodeoxyribonuclease I	5,263	54500,812	5,446	2		97400	67120	14,6	27,6	0,69	0,06	0,11
B1XEW6_ECODH	*sbmA*	Predicted transporter	7,143	46459,391	8,458	2	transport	34265	284495	8,2	14,2	8,30	0,00	0,00
B1X6U5_ECODH	*sbmC*	DNA gyrase inhibitor	10,828	18081,463	4,612	1		53327	26571	33,2	51,1	0,50	0,04	0,08
B1XH81_ECODH	*sdaA*	L-serine deaminase I	19,604	48906,645	5,178	7	metabolism	1562565	3666072	9,4	8,0	2,35	0,00	0,00
B1XDK6_ECODH	*sdaB*	L-serine deaminase II	29,011	48752,926	5,509	9		4420011	3066304	9,6	24,4	0,69	0,02	0,05
B1XDK5_ECODH	*sdaC*	Predicted serine transporter	11,888	46906,301	9,209	4		1097326	838717	20,6	13,3	0,76	0,07	0,12
B1X6Q4_ECODH	*sdhA*	Succinate dehydrogenase, flavoprotein subunit	33,503	64421,844	5,849	13		10266339	11358839	5,1	9,9	1,11	0,12	0,19
B1X6Q5_ECODH	*sdhB*	Succinate dehydrogenase, FeS subunit	50,42	26769,863	6,314	10		2265945	2451039	23,1	12,0	1,08	0,56	0,63
SECA_ECODH	*secA*	Protein translocase subunit SecA	42,175	102022,99	5,431	26		7036238	19703750	2,6	13,4	2,80	0,00	0,00
SECB_ECODH	*secB*	Protein-export protein SecB	55,484	17277,361	4,265	5		8539453	8512705	4,5	4,2	1,00	0,92	0,94
B1XEZ6_ECODH	*secD*	Protein translocase subunit SecD	33,496	66632,148	8,621	15		2832914	4090436	10,6	8,8	1,44	0,00	0,01
B1XEZ7_ECODH	*secF*	Protein-export membrane protein SecF	12,074	35382,379	5,567	2		589297	681870	8,7	12,6	1,16	0,11	0,19
B1XGY5_ECODH	*secG*	Preprotein translocase membrane subunit	27,273	11365,157	6,091	2		2904428	3040066	9,5	20,6	1,05	0,70	0,76
B1X6F2_ECODH	*secY*	Protein translocase subunit SecY	16,253	48511,688	9,894	5		1980504	2180358	12,8	14,1	1,10	0,34	0,44
B1X8K6_ECODH	*selB*	Selenocysteinyl-tRNA-specific translation factor	17,427	68867,445	6,107	7		431678	417805	6,1	8,4	0,97	0,55	0,63
B1XGM5_ECODH	*selD*	Selenide, water dikinase	45,245	36687,254	5,299	9		3253475	3410443	9,7	8,3	1,05	0,49	0,58
B1X6M0_ECODH	*seqA*	Negative modulator of initiation of replication	30,387	20315,447	5,815	4		702313	752362	8,7	12,3	1,07	0,38	0,48
B1XEJ8_ECODH	*serA*	D-3-phosphoglycerate dehydrogenase	20	44175,777	5,921	6		289084	378566	13,8	10,9	1,31	0,02	0,05
B1XFJ8_ECODH	*serB*	3-phosphoserine phosphatase	10,559	35042,691	5,492	3		164011	199961	7,5	10,8	1,22	0,02	0,05
SERC_ECODH	*serC*	Phosphoserine aminotransferase	37,293	39783,309	5,369	11		2351280	2424553	6,6	12,1	1,03	0,66	0,73
SYS_ECODH	*serS*	Serine--tRNA ligase	34,651	48414,023	5,34	12		8339481	8521732	6,7	10,4	1,02	0,73	0,78
B1X9L3_ECODH	*sixA*	Phosphohistidine phosphatase	8,696	17207,773	4,454	2		81676	74413	14,8	21,6	0,91	0,49	0,58
B1XD47_ECODH	*skp*	Chaperone protein skp	22,36	17688,234	9,694	3		6459720	1936334	7,4	12,0	0,30	0,00	0,00
SLMA_ECODH	*slmA*	Nucleoid occlusion factor SlmA	16,162	22836,344	8,783	2		146629	72991	6,2	68,1	0,50	0,05	0,09
B1X7W1_ECODH	*slp*	Outer membrane lipoprotein	29,787	20963,914	6,822	3		6614950	2132914	9,1	20,9	0,32	0,00	0,00
B1XFK2_ECODH	*slt*	Lytic murein transglycosylase, soluble	23,256	73353,156	8,847	11	ptidoglycan-modificat	399524	1683564	8,2	7,6	4,21	0,00	0,00
B1XFV3_ECODH	*slyB*	Outer membrane lipoprotein	30,968	15601,666	9,356	3		6702449	7225113	18,5	13,7	1,08	0,52	0,60
B1X6J8_ECODH	*slyD*	Peptidyl-prolyl cis-trans isomerase	11,735	20852,832	4,856	2		2061134	1821533	8,3	19,1	0,88	0,26	0,35
SLYX_ECODH	*slyX*	Protein SlyX	38,889	8214,33	4,889	2		206106	174914	12,8	14,8	0,85	0,16	0,24
B1XBT7_ECODH	*smpA*	Outer membrane protein assembly factor BamE	17,699	12302,034	8,71	1		929886	1178732	12,7	9,4	1,27	0,02	0,05
B1XB75_ECODH	*sodA*	Superoxide dismutase	12,136	23097,098	6,444	2		1260496	1848429	6,1	24,8	1,47	0,02	0,05
B1XFW7_ECODH	*sodB*	Superoxide dismutase	44,56	21265,779	5,581	5		3321777	5517831	33,5	39,1	1,66	0,08	0,14
B1XFV7_ECODH	*sodC*	Superoxide dismutase [Cu-Zn]	10,405	17680,977	5,947	1		#ZAHL!	1503	#DIV/0!	36,2	#NV	#NV	#NV
B1XGU2_ECODH	*sohA*	Predicted regulator	13,514	12358,941	5,109	1		60651	95942	5,2	20,5	1,58	0,01	0,02
B1XBM1_ECODH	*sohB*	Predicted inner membrane peptidase	12,034	39366,441	9,241	3		97908	122991	12,3	23,2	1,26	0,14	0,22
MTOX_ECODH	*solA*	N-methyl-L-tryptophan oxidase	23,925	40902,004	5,142	6		465987	443011	12,1	7,8	0,95	0,52	0,60
B1XFA0_ECODH	*speA*	Biosynthetic arginine decarboxylase	31,307	73898,445	4,832	13		2869198	2332413	7,6	7,8	0,81	0,01	0,02
SPEB_ECODH	*speB*	Agmatinase	25,49	33557,043	5,142	6		1621784	1687922	7,5	11,9	1,04	0,59	0,66
SPED_ECODH	*speD*	S-adenosylmethionine decarboxylase proenzym	13,636	30384,643	5,19	2		210159	231305	11,9	13,3	1,10	0,33	0,43

SPEE_ECODH	*speE*	Spermidine synthase	23,611	32321,391	5,33	3		590995	710362	8,4	12,0	1,20	0,05	0,09
B1XF49_ECODH	*speG*	Spermidine N1-acetyltransferase	28,495	21887,014	6,201	4		626163	705915	10,4	17,9	1,13	0,29	0,39
B1X984_ECODH	*spoU*	tRNA (Guanosine-2'-O-)-methyltransferase	10,48	25343,074	6,706	2		115848	156279	10,3	6,1	1,35	0,00	0,01
B1XGM7_ECODH	*sppA*	Protease IV (Signal peptide peptidase)	19,256	67219,336	5,724	7		554022	684764	7,9	7,8	1,24	0,01	0,03
B1XGK4_ECODH	*spy*	Envelope stress induced periplasmic protein	38,509	18199,072	9,66	6	stress response	430101	2541373	23,8	19,7	5,91	0,00	0,00
B1XCN1_ECODH	*srlB*	Glucitol/sorbitol-specific enzyme IIA component	51,22	13304,049	4,957	3		2633225	934813	7,8	3,7	0,36	0,00	0,00
B1XCN2_ECODH	*srlD*	Sorbitol-6-phosphate dehydrogenase	58,301	27857,877	5,922	10		2952693	2972755	13,1	24,0	1,01	0,96	0,97
B1XCN0_ECODH	*srlE*	Glucitol/sorbitol-specific enzyme IIB component	17,555	33332,039	6,516	4		1214495	567948	3,0	12,5	0,47	0,00	0,00
B1XCN4_ECODH	*srlR*	DNA-bindng transcriptional repressor	8,949	28236,148	5,217	2		61065	53731	24,0	15,6	0,88	0,41	0,50
B1XBQ3_ECODH	*srmB*	ATP-dependent RNA helicase SrmB	27,928	49914,141	9,273	9		1597581	1704634	12,1	7,2	1,07	0,40	0,49
B1XCU6_ECODH	*ssb*	Single-stranded DNA-binding protein	16,854	18974,996	5,441	3		1492187	1913024	11,0	17,5	1,28	0,05	0,10
B1XDH7_ECODH	*sscR*	6-carboxy-5,6,7,8-tetrahydropterin synthase	15,702	13772,88	6,037	1		36786	48924	13,4	31,8	1,33	0,20	0,29
B1XAZ6_ECODH	*sseA*	3-mercaptopyruvate sulfurtransferase	23,132	30811,84	4,562	6		1486572	857689	16,4	9,7	0,58	0,00	0,00
B1XHK2_ECODH	*sspA*	Stringent starvation protein A	44,34	24304,908	5,221	7		4153052	3672145	9,4	11,1	0,88	0,14	0,22
B1XHK1_ECODH	*sspB*	ClpXP protease specificity-enhancing factor	21,212	18262,416	4,377	3		399718	396495	10,0	11,8	0,99	0,92	0,94
STHA_ECODH	*sthA*	Soluble pyridine nucleotide transhydrogenase	27,253	51560,395	6,084	6		2461945	1920999	8,8	3,2	0,78	0,00	0,01
B1XCK7_ECODH	*stpA*	DNA-binding protein	30,597	15347,512	7,95	3		5231610	2289017	8,6	22,9	0,44	0,00	0,00
B1X6Q6_ECODH	*sucA*	2-oxoglutarate decarboxylase, thiamin-requiring	33,441	105061,72	6,038	23		10894610	10968200	4,4	1,8	1,01	0,79	0,83
B1X6Q7_ECODH	*sucB*	Dihydrolipoyltranssuccinase	28,889	44011,395	5,576	9		13005169	12024151	10,3	14,2	0,92	0,39	0,49
SUCC_ECODH	*sucC*	Succinyl-CoA ligase [ADP-forming] subunit beta	40,722	41392,645	5,367	13		14918991	17295996	8,8	10,0	1,16	0,07	0,12
B1X6Q9_ECODH	*sucD*	Succinyl-CoA ligase [ADP-forming] subunit alpha	46,021	29777,477	6,318	9		5049959	7198467	9,6	4,0	1,43	0,00	0,00
B1XFZ2_ECODH	*sufA*	Fe-S cluster assembly protein	16,393	13300,113	4,848	1		105006	178650	11,9	21,6	1,70	0,00	0,01
B1XFZ1_ECODH	*sufB*	Component of SufBCD complex	8,687	54745,316	5,058	3		48123	112872	24,3	25,1	2,35	0,00	0,01
B1XFZ0_ECODH	*sufC*	Component of SufBCD complex, ATP-binding cc	11,29	27582,367	4,84	3		33116	97597	54,6	8,7	2,95	0,01	0,02
B1XFY9_ECODH	*sufD*	Component of SufBCD complex	5,674	46822,75	6,358	2		52298	94583	16,8	14,6	1,81	0,00	0,01
B1XFG8_ECODH	*sufI*	Cell division protein FtsP	15,319	51858,164	5,935	5		262052	218299	12,8	3,2	0,83	0,04	0,07
B1XB08_ECODH	*suhB*	Inositol monophosphatase	38,577	29172,127	6,448	7		3186870	3517958	9,6	4,1	1,10	0,10	0,16
B1XC54_ECODH	*surA*	Chaperone SurA	36,682	47283,715	6,478	11		3288813	4106037	6,8	7,0	1,25	0,00	0,01
B1XAD5_ECODH	*talA*	Transaldolase	33,544	35658,801	5,889	9		4495993	4672033	22,5	15,4	1,04	0,79	0,83
B1XBD3_ECODH	*talB*	Transaldolase	60,252	35219,246	5,106	15		35799809	35970717	3,6	8,0	1,00	0,92	0,94
TAM_ECODH	*tam*	Trans-aconitate 2-methyltransferase	10,317	29005,959	4,855	2		91907	61207	9,9	33,2	0,67	0,06	0,12
B1XDP0_ECODH	*tas*	Predicted oxidoreductase, NADP(H)-dependent	6,647	38499,539	6,266	2		204733	233080	12,1	28,0	1,14	0,39	0,49
B1XAK1_ECODH	*tatB*	Sec-independent protein translocase protein Tat	14,035	18420,869	5,133	2		125491	133428	17,5	15,7	1,06	0,63	0,70
B1X622_ECODH	*tatE*	Probable Sec-independent protein translocase p	16,418	7024,294	9,05	1		93079	72579	15,8	20,1	0,78	0,11	0,18
B1XGT2_ECODH	*tdcB*	Catabolic threonine dehydratase, PLP-depender	23,1	35232,414	5,749	5		1556274	72065	12,0	11,0	0,05	0,00	0,00
B1XGS9_ECODH	*tdcE*	Pyruvate formate-lyase 4/2-ketobutyrate formate	17,147	85935,547	5,483	8		6195414	2556586	6,8	14,0	0,41	0,00	0,00
TDH_ECODH	*tdh*	L-threonine 3-dehydrogenase	48,387	37239,043	5,943	10		2782344	2447536	9,8	10,0	0,88	0,12	0,20
B1XAT0_ECODH	*tdk*	Thymidine kinase	28,293	23456,576	5,98	3		433310	469104	8,4	6,1	1,08	0,18	0,26
B1XDE1_ECODH	*tehB*	Predicted S-adenosyl-L-methionine-dependent n	30,457	22530,734	6,836	6		263071	148541	9,2	9,0	0,56	0,00	0,00
B1XFP0_ECODH	*tesB*	Acyl-CoA thioesterase II	9,091	31966,326	6,173	2		135110	117432	13,8	37,1	0,87	0,47	0,56
TGT_ECODH	*tgt*	Queuine tRNA-ribosyltransferase	8,533	42593,66	5,975	3		125627	132501	23,2	21,5	1,05	0,75	0,81
THII_ECODH	*thiI*	tRNA sulfurtransferase	21,162	54973,098	6,162	10		1499820	1831307	10,2	7,4	1,22	0,02	0,04
B1XBC7_ECODH	*thrA*	Fused aspartokinase I and homoserine dehydrog	45,854	89120,242	5,465	25	metabolism	3427310	8119788	4,3	1,4	2,37	0,00	0,00
KHSE_ECODH	*thrB*	Homoserine kinase	13,548	33623,645	5,453	2	metabolism	142783	401590	16,2	8,0	2,81	0,00	0,00
B1XBC9_ECODH	*thrC*	Threonine synthase	36,215	47113,836	5,238	10		2051717	3715449	7,7	16,4	1,81	0,00	0,00
SYT_ECODH	*thrS*	Threonine--tRNA ligase	21,651	74014,297	5,802	13		3702019	4634579	16,7	9,2	1,25	0,05	0,09
B1XDN3_ECODH	*thyA*	Thymidylate synthase	21,97	30479,688	5,62	5		718407	702947	12,1	14,3	0,98	0,83	0,87
TIG_ECODH	*tig*	Trigger factor	57,176	48192,668	4,825	20		53856473	57444327	6,2	9,3	1,07	0,29	0,39
B1XF97_ECODH	*tktA*	Transketolase	26,998	72211,742	5,429	13		14282430	14727234	3,6	10,6	1,03	0,62	0,69
B1XAD6_ECODH	*tktB*	Transketolase	19,34	73042,719	5,863	9		1351591	1170146	12,7	8,1	0,87	0,10	0,17
B1XHL6_ECODH	*tldD*	Predicted peptidase	24,532	51364,094	4,93	7		746325	750427	2,8	7,9	1,01	0,90	0,92
KTHY_ECODH	*tmk*	Thymidylate kinase	24,883	23783,143	5,326	4		771246	670957	26,7	18,5	0,87	0,40	0,50
TNAA_ECODH	*tnaA*	Tryptophanase	65,605	52773,465	5,876	21		216942465	29532619	10,4	5,9	0,14	0,00	0,00
B1X6S0_ECODH	*tolA*	Membrane anchored protein in TolA-TolQ-TolR c	7,838	43156,625	9,097	2		56836	74125	18,5	21,0	1,30	0,12	0,19
TOLB_ECODH	*tolB*	Protein TolB	41,628	45955,539	6,979	12		3905275	4615669	11,8	6,7	1,18	0,04	0,08
B1XG40_ECODH	*tolC*	Transport channel	54,97	53740,723	5,461	16		10467846	10803397	12,1	8,1	1,03	0,68	0,75
B1X6R8_ECODH	*tolQ*	Membrane spanning protein in TolA-TolQ-TolR c	13,043	25597,717	6,53	2		199136	347314	12,6	13,6	1,74	0,00	0,00
B1XBM3_ECODH	*topA*	DNA topoisomerase 1	20,578	97349,758	8,678	13		2564909	2451910	12,4	7,6	0,96	0,57	0,65
TPIS_ECODH	*tpiA*	Triosephosphate isomerase	48,235	26971,812	5,637	6		5623175	3999787	7,3	7,5	0,71	0,00	0,00
B1XCF2_ECODH	*tpx*	Probable thiol peroxidase	44,643	17835,316	4,75	5		9125338	10943302	3,2	9,2	1,20	0,01	0,02
TREA_ECODH	*treA*	Periplasmic trehalase	5,31	63636,719	5,6	2		38822	27819	12,2	25,7	0,72	0,08	0,14
B1XEM1_ECODH	*treB*	Fused trehalose(Maltose)-specific PTS enzyme:	21,142	51080,742	9,139	7		6556994	4053082	7,0	24,6	0,62	0,02	0,04
B1XEM0_ECODH	*treC*	Trehalose-6-P hydrolase	21,96	63837,672	5,513	11		8307341	5010575	2,5	16,7	0,60	0,00	0,00
TRMA_ECODH	*trmA*	tRNA/tmRNA (uracil-C(5))-methyltransferase	16,667	41966,949	5,707	3		397850	346703	4,5	7,2	0,87	0,02	0,04
TRMD_ECODH	*trmD*	tRNA (guanine-N(1)-)-methyltransferase	10,588	28422,359	5,502	2		153857	141976	11,9	5,8	0,92	0,25	0,35
B1XFC0_ECODH	*trmI*	tRNA (guanine-N(7)-)-methyltransferase	14,644	27307,322	6,417	3		382752	319062	16,6	24,6	0,83	0,28	0,38
TRPA_ECODH	*trpA*	Tryptophan synthase alpha chain	22,761	28724,162	5,314	4		182293	330537	18,4	13,3	1,81	0,00	0,01
TRPB_ECODH	*trpB*	Tryptophan synthase beta chain	7,305	42983,008	5,709	2		189680	224779	11,8	15,7	1,19	0,14	0,21
B1X731_ECODH	*trpS*	Tryptophan--tRNA ligase	32,934	37437,82	6,267	8		1883796	2158390	17,5	14,1	1,15	0,26	0,36
TRUA_ECODH	*truA*	tRNA pseudouridine synthase A	10,37	30399,619	8,683	2		82231	84510	21,7	8,4	1,03	0,84	0,88
B1XGX8_ECODH	*truB*	tRNA pseudouridine synthase B	29,936	35087,078	5,705	6		483916	613452	15,4	13,9	1,27	0,06	0,11
TRUD_ECODH	*truD*	tRNA pseudouridine synthase D	27,794	39091,488	6,153	6		556589	599360	13,2	8,9	1,08	0,41	0,50
B1X9Z5_ECODH	*trxA*	Thioredoxin	46,789	11806,623	4,674	4		6981308	7101303	15,4	16,6	1,02	0,88	0,91
B1X828_ECODH	*trxB*	Thioredoxin reductase	62,305	34623,035	5,295	12		5068777	5265744	9,6	7,4	1,04	0,55	0,63

TSAC_ECODH	*tsaC*	Threonylcarbamoyl-AMP synthase	28,947	20767,738	4,941	3		244473	201572	10,2	19,0	0,82	0,11	0,18
TSAD_ECODH	*tsaD*	tRNA N6-adenosine threonylcarbamoyltransfera	6,825	36008,406	5,915	2		81214	66965	21,6	26,8	0,82	0,32	0,42
EFTS_ECODH	*tsf*	Elongation factor Ts	49,47	30422,979	5,215	11		32316195	40505419	8,0	7,6	1,25	0,01	0,02
B1XEZ9_ECODH	*tsx*	Nucleoside channel, receptor of phage T6 and c	42,177	33589,023	5,07	8		8925515	7824026	9,0	16,3	0,88	0,20	0,28
TTCA_ECODH	*ttcA*	tRNA 2-thiocytidine biosynthesis protein TtcA	28,939	35560,914	5,953	6		586470	526016	3,1	7,0	0,90	0,03	0,06
B1XBY2_ECODH	*tufB*	Elongation factor Tu				19		534128784	643875982	2,9	2,6	1,21	0,00	0,00
TUSA_ECODH	*tusA*	Sulfurtransferase TusA	23,457	9094,543	5,181	1		97180	77066	42,1	23,1	0,79	0,32	0,42
TUSB_ECODH	*tusB*	Protein TusB	12,632	10718,333	5,115	1		43024	35092	15,4	33,1	0,82	0,35	0,45
B1XBS2_ECODH	*tyrA*	T-protein	7,775	42042,523	5,682	2		28274	45066	5,6	3,3	1,59	0,00	0,00
B1XCU2_ECODH	*tyrB*	Tyrosine aminotransferase, tyrosine-repressible,	41,562	43537,809	5,318	12		1204543	1898735	14,6	7,9	1,58	0,00	0,01
B1XCF1_ECODH	*tyrR*	DNA-binding transcriptional dual regulator, tyrosi	12,865	57656,141	5,543	5		226553	153315	7,6	19,6	0,68	0,01	0,03
SYY_ECODH	*tyrS*	Tyrosine--tRNA ligase	26,651	47526,969	5,59	9		1880290	2140157	9,2	6,6	1,14	0,06	0,11
B1XAK5_ECODH	*ubiD*	3-octaprenyl-4-hydroxybenzoate carboxy-lyase	19,718	55603,75	5,31	7		739822	746301	13,2	8,4	1,01	0,91	0,93
UBIE_ECODH	*ubiE*	Ubiquinone/menaquinone biosynthesis C-methyl	33,068	28073,211	7,771	7		1568345	1390086	14,3	2,5	0,89	0,15	0,22
UBIG_ECODH	*ubiG*	Ubiquinone biosynthesis O-methyltransferase	42,5	26555,451	6,119	6		739010	944487	9,4	13,9	1,28	0,02	0,05
B1XA97_ECODH	*ucpA*	Predicted oxidoredutase, sulfate metabolism pro	20,152	27849,975	5,126	4		903457	503287	3,9	9,6	0,56	0,00	0,00
B1X702_ECODH	*udk*	Uridine kinase	12,676	24353,115	6,393	3		354055	318837	9,3	2,2	0,90	0,06	0,11
B1XAJ5_ECODH	*udp*	Uridine phosphorylase	69,565	27159,082	5,813	10		6045186	16002811	18,0	16,0	2,65	0,00	0,00
B1XF82_ECODH	*uidA*	Beta-D-glucuronidase	21,891	68447	5,244	9	metabolism	316805	6375517	10,4	10,8	20,12	0,00	0,00
UPP_ECODH	*upp*	Uracil phosphoribosyltransferase	48,558	22533,264	5,317	7		6777178	5662948	9,1	10,6	0,84	0,04	0,08
B1X930_ECODH	*usg*	Predicted semialdehyde dehydrogenase	20,475	36364,133	4,381	3		364235	391602	12,2	11,9	1,08	0,43	0,53
B1XFR7_ECODH	*ushA*	Bifunctional UDP-sugar hydrolase and 5'-nucleot	13,091	60823,973	5,475	5		556841	431605	5,2	16,1	0,78	0,02	0,05
B1XCG2_ECODH	*uspE*	Stress-induced protein	27,848	35706,879	5,157	5		968793	594131	4,1	13,0	0,61	0,00	0,00
B1XCK3_ECODH	*uspF*	Stress-induced protein, ATP-binding protein	18,056	16016,532	5,603	2		713848	383204	11,0	8,3	0,54	0,00	0,00
B1X605_ECODH	*uspG*	Universal stress protein UP12	49,296	15935,185	6,028	5		1541950	1418776	6,6	15,3	0,92	0,36	0,46
B1X8Q3_ECODH	*uup*	Fused predicted transporter subunits of ABC sup	9,921	72066,883	5,355	3		347461	286489	16,6	16,0	0,82	0,13	0,20
UVRB_ECODH	*uvrB*	UvrABC system protein B	3,269	76226,055	5,14	2		63712	42758	19,0	15,4	0,67	0,02	0,04
B1XAH9_ECODH	*uvrD*	DNA-dependent ATPase I and helicase II	17,222	81989,883	5,857	10		504218	678694	7,9	2,3	1,35	0,00	0,00
B1X672_ECODH	*uvrY*	DNA-binding response regulator in two-compone	22,477	23892,654	6,528	4		148162	158365	29,7	11,2	1,07	0,67	0,73
UXAC_ECODH	*uxaC*	Uronate isomerase	25,957	53987,18	5,438	8		886999	664229	5,7	6,7	0,75	0,00	0,00
B1X9L9_ECODH	*vacJ*	Predicted lipoprotein	17,928	28041,602	4,895	3		409319	336236	18,7	27,8	0,82	0,24	0,34
B1XEN7_ECODH	*valS*	Valine--tRNA ligase	34,595	108192,35	5,199	24		9604324	11241064	6,2	4,5	1,17	0,01	0,02
B1X6X0_ECODH	*wbbI*	Conserved protein	26,97	37757,215	5,847	7		2668883	2126479	9,2	17,7	0,80	0,06	0,11
B1X6W8_ECODH	*wbbK*	Lipopolysaccharide biosynthesis protein	23,118	43188,176	9,017	7		802727	440558	4,2	16,3	0,55	0,00	0,00
WECG_ECODH	*wecG*	UDP-N-acetyl-D-mannosaminuronic acid transfe	19,106	27928,182	9,562	3		159654	187986	14,1	20,3	1,18	0,23	0,32
B1XAF6_ECODH	*wzzE*	Enterobacterial Common Antigen (ECA) polysac	32,471	39489,102	6,247	6		550883	628079	13,4	13,1	1,14	0,21	0,30
EX7L_ECODH	*xseA*	Exodeoxyribonuclease 7 large subunit	12,281	51832,23	9,992	4		127161	251999	11,5	9,5	1,98	0,00	0,00
EX7S_ECODH	*xseB*	Exodeoxyribonuclease 7 small subunit	35	8951,932	4,429	2		591935	721907	12,1	17,5	1,22	0,09	0,16
B1XGL0_ECODH	*xthA*	Exonuclease III	30,597	30969,166	5,799	8		917699	835708	8,4	5,8	0,91	0,11	0,18
YAAA_ECODH	*yaaA*	UPF0246 protein YaaA	18,217	29585,828	6,859	4		408557	348829	18,1	17,5	0,85	0,26	0,36
B1XCA2_ECODH	*yadG*	Predicted transporter subunit: ATP-binding comp	16,883	34647,066	8,438	4		194820	324863	3,1	11,4	1,67	0,00	0,00
YAEH_ECODH	*yaeH*	UPF0325 protein YaeH	39,062	15096,169	6,614	5		964223	807939	5,9	10,2	0,84	0,03	0,06
YAEP_ECODH	*yaeP*	UPF0253 protein YaeP	56,061	7214,222	4,536	2		303109	296822	11,8	11,6	0,98	0,81	0,84
B1XD72_ECODH	*yafC*	Predicted DNA-binding transcriptional regulator	8,553	33775,82	6,93	2		32368	48960	54,0	15,6	1,51	0,16	0,23
YAIE_ECODH	*yaiE*	UPF0345 protein YaiE	23,404	10234,396	4,469	2		463908	598073	8,6	11,3	1,29	0,01	0,03
B1XEU7_ECODH	*yaiL*	Nucleoprotein/polynucleotide-associated enzyme	17,318	19922,76	7,834	2		145567	86412	16,5	24,9	0,59	0,01	0,03
B1XEZ5_ECODH	*yajC*	SecYEG protein translocase auxillary subunit	20	11887,143	9,572	2		1152952	2097387	19,4	22,4	1,82	0,01	0,02
B1XEZ8_ECODH	*yajD*	Conserved protein	16,522	13363,993	6,14	1		87268	47688	20,0	36,5	0,55	0,03	0,06
B1XFM2_ECODH	*yajG*	Predicted lipoprotein	40,104	20950,027	8,685	4		1141064	1882447	11,1	43,7	1,65	0,04	0,09
B1XF12_ECODH	*yajL*	Conserved protein	25	20777,092	5,245	3		344315	323943	11,0	12,4	0,94	0,49	0,57
B1XF07_ECODH	*yajO*	Predicted oxidoreductase, NAD(P)-binding	27,778	36420,168	5,185	6		284441	274548	7,8	26,8	0,97	0,80	0,84
YAJQ_ECODH	*yajQ*	UPF0234 protein YajQ	53,988	18343,861	5,957	7		6440318	5759086	3,9	13,2	0,89	0,16	0,24
YBAB_ECODH	*ybaB*	Nucleoid-associated protein YbaB	21,101	12014,82	5,012	2		824154	726170	17,9	8,7	0,88	0,25	0,35
B1XFR5_ECODH	*ybaL*	Predicted transporter with NAD(P)-binding Rossı	5,018	59423,531	4,996	2		119484	141528	11,7	10,8	1,18	0,08	0,14
B1XFS2_ECODH	*ybaS*	Glutaminase	16,452	32903,324	4,814	2		171014	111850	22,7	17,0	0,65	0,03	0,06
B1XFP1_ECODH	*ybaY*	Predicted outer membrane lipoprotein	32,632	19431,199	7,866	3		1254415	564891	12,3	14,7	0,45	0,00	0,00
B1XFS9_ECODH	*ybbN*	Predicted thioredoxin domain-containing protein	63,028	31791,064	4,499	9		2065000	4900142	15,4	14,6	2,37	0,00	0,00
B1XGC6_ECODH	*ybcJ*	Predicted RNA-binding protein	30	7389,537	7,838	2		136434	262651	55,6	31,2	1,93	0,06	0,11
B1X5X4_ECODH	*ybdF*	Conserved protein	13,115	14050,323	8,71	1		18574	16788	9,8	36,9	0,90	0,57	0,65
B1X5X2_ECODH	*ybdG*	Predicted mechanosensitive channel	6,988	46602,648	7,872	2		143168	173398	29,8	16,3	1,21	0,27	0,37
B1X632_ECODH	*ybeB*	Ribosomal silencing factor RsfS	15,238	11582,228	4,519	1		192910	230964	18,8	13,5	1,20	0,17	0,25
B1X626_ECODH	*ybeD*	UPF0250 protein YbeD	39,08	9827,346	5,497	2		1129884	1754657	8,7	22,1	1,55	0,01	0,03
B1X638_ECODH	*ybeL*	Conserved protein	16,875	18797,201	5,107	3		163310	107715	30,4	18,9	0,66	0,08	0,14
B1X655_ECODH	*ybeZ*	Uncharacterized protein	52,601	39038,699	5,711	12		2496865	5432387	6,6	12,1	2,18	0,00	0,00
B1X6L8_ECODH	*ybfE*	LexA regulated protein	16,495	11279,979	10,09	1		85785	65859	11,4	27,2	0,77	0,13	0,20
B1X6S3_ECODH	*ybgF*	Uncharacterized protein	31,559	28231,404	8,581	4		1237424	1324024	13,3	7,9	1,07	0,44	0,53
B1X6P1_ECODH	*ybgI*	Putative GTP cyclohydrolase 1 type 2	27,53	26892,48	5,067	4		1215452	2043412	3,9	8,5	1,68	0,00	0,00
B1X6P2_ECODH	*ybgJ*	Predicted enzyme subunit	12,844	23946,602	5,162	1		61077	45589	20,6	40,4	0,75	0,35	0,45
B1X6P3_ECODH	*ybgK*	Predicted enzyme subunit	17,742	34386,352	8,591	5		137380	180970	5,9	8,3	1,32	0,00	0,01
B1X796_ECODH	*ybhA*	Pyridoxal phosphatase / fructose 1,6-bisphospha	30,147	30201,309	5,506	4		327548	438374	12,9	34,0	1,34	0,17	0,25
B1X7A3_ECODH	*ybhB*	Predicted kinase inhibitor	20,886	17085,121	5,27	2		178622	114742	9,1	9,0	0,64	0,00	0,00
B1X7A2_ECODH	*ybhC*	Predicted pectinesterase	33,255	46082,199	5,662	8		2014217	2939004	4,8	9,3	1,46	0,00	0,00
B1X7D0_ECODH	*ybiB*	Predicted transferase/phosphorylase	16,875	35048,539	6,377	4		263535	95274	14,4	24,5	0,36	0,00	0,00
B1X7E9_ECODH	*ybiS*	Conserved protein	44,118	33325,277	5,992	7		1975076	1534051	14,1	25,9	0,78	0,12	0,19

B1X7F0_ECODH	*ybiT*	Fused predicted transporter subunits of ABC sup	21,698	59857,996	4,989	8		1746348	2045591	5,2	4,9	1,17	0,00	0,01
B1X7F2_ECODH	*ybiV*	Predicted hydrolase	23,616	30412,828	5,909	4		175960	219505	15,5	11,7	1,25	0,07	0,12
B1X806_ECODH	*ybjP*	Predicted lipoprotein	42,69	18991,309	6,052	5		1293169	1604976	8,0	8,0	1,24	0,01	0,02
B1X818_ECODH	*ybjX*	Conserved protein	24,242	38357,551	9,437	5		739998	1085838	7,6	15,5	1,47	0,00	0,01
B1X837_ECODH	*ycaC*	Predicted hydrolase	31,25	23100,314	5,201	4		615297	180352	15,3	20,0	0,29	0,00	0,00
B1X845_ECODH	*ycaO*	Conserved protein	14,676	65651,578	4,379	6		2285868	3294295	2,6	10,2	1,44	0,00	0,00
B1X8M9_ECODH	*ycbB*	Predicted carboxypeptidase	7,317	67812,492	8,634	3	ptidoglycan-modificat	23424	109916	16,1	4,9	4,69	0,00	0,00
B1X8N1_ECODH	*ycbL*	Predicted metal-binding enzyme	39,07	23784,051	4,946	5		940440	1213688	10,4	10,0	1,29	0,01	0,03
B1X8Q1_ECODH	*ycbX*	Predicted 2Fe-2S cluster-containing protein	10,298	40644,41	7,577	2		130136	156826	15,3	19,1	1,21	0,16	0,25
B1X8Q9_ECODH	*ycbZ*	Predicted peptidase	16,041	65818,656	4,689	5		337914	427807	11,7	10,4	1,27	0,03	0,06
B1X8R5_ECODH	*yccF*	Conserved inner membrane protein	14,865	16275,364	9,412	2		44816	52714	16,2	5,2	1,18	0,11	0,18
B1X9C4_ECODH	*yccJ*	Uncharacterized protein	25,333	8524,493	4,701	1		366409	247182	21,0	3,6	0,67	0,02	0,04
B1X8R9_ECODH	*yccU*	Predicted CoA-binding protein with NAD(P)-bind	18,248	14701,123	6,723	2		254998	119727	17,4	13,5	0,47	0,00	0,00
YCDX_ECODH	*ycdX*	Probable phosphatase YcdX	8,163	26890,512	5,534	2		89464	104788	24,6	8,2	1,17	0,29	0,38
B1X9E9_ECODH	*ycdY*	Conserved protein	16,304	20784,387	4,209	2		742367	784878	10,0	25,9	1,06	0,68	0,74
B1X9H6_ECODH	*yceB*	Predicted lipoprotein	25,806	20499,74	6,15	3		379816	398084	7,9	22,1	1,05	0,68	0,74
B1X9K0_ECODH	*yceF*	Maf-like protein YceF	24,227	21690,799	5,887	3		174398	139761	37,8	23,1	0,80	0,39	0,49
YCEH_ECODH	*yceH*	UPF0502 protein YceH	49,767	24177,33	5,02	7		876840	786479	7,3	4,8	0,90	0,05	0,09
B1XA38_ECODH	*ycfD*	Conserved protein	26,005	42578,918	4,694	6		527282	611009	2,3	6,6	1,16	0,01	0,02
B1XA15_ECODH	*ycfM*	Penicillin-binding protein activator LpoB	36,15	22515,645	6,412	5		695778	707739	20,2	8,5	1,02	0,88	0,90
YCFP_ECODH	*ycfP*	UPF0227 protein YcfP	31,667	21226,176	6,132	5		1079210	580000	6,7	4,5	0,54	0,00	0,00
B1XA23_ECODH	*ycfS*	Conserved protein	7,188	34619,887	8,887	2	ptidoglycan-modificat	3596	103221	86,4	16,9	28,70	0,03	0,06
B1XA65_ECODH	*ycgK*	Uncharacterized protein	22,556	14905,824	9,498	2		58717	116301	14,4	24,1	1,98	0,00	0,01
YCGL_ECODH	*ycgL*	Protein YcgL	42,593	12414,504	9,162	3		385043	422892	19,1	14,1	1,10	0,49	0,58
B1XA67_ECODH	*ycgM*	Predicted isomerase/hydrolase	10,959	23711,117	5,879	1		165946	137919	24,9	23,4	0,83	0,35	0,45
YCGN_ECODH	*ycgN*	UPF0260 protein YcgN	12,418	17910,371	4,899	1		64202	62095	9,6	21,3	0,97	0,80	0,84
B1XAP4_ECODH	*ychF*	Ribosome-binding ATPase YchF	61,708	39667,32	4,87	15		8302648	9384256	5,5	11,4	1,13	0,09	0,15
B1XAR3_ECODH	*ychN*	Conserved protein	24,786	12692,747	5,018	2		138007	135519	22,5	21,4	0,98	0,91	0,93
B1XBK6_ECODH	*yciE*	Conserved protein	11,31	18961,447	4,939	1		30444	10157	8,7	48,9	0,33	0,01	0,03
B1XBK7_ECODH	*yciF*	Conserved protein	18,072	18597,164	5,474	2		211326	68564	26,4	22,4	0,32	0,00	0,00
B1XBM0_ECODH	*yciK*	Predicted oxoacyl-(Acyl carrier protein) reductas	15,079	27932,912	7,666	3		149321	215038	6,0	20,7	1,44	0,01	0,03
B1XBM2_ECODH	*yciN*	Uncharacterized protein	31,325	9385,636	5,474	2		589908	343508	11,7	18,1	0,58	0,00	0,01
B1XBL6_ECODH	*yciO*	Conserved protein	24,272	23211,758	5,969	4		607359	640552	9,3	12,7	1,05	0,52	0,60
B1XBN5_ECODH	*yciT*	Predicted DNA-binding transcriptional regulator	15,261	27602,605	5,986	2		264278	194765	10,0	10,1	0,74	0,01	0,02
YCJF_ECODH	*ycjF*	UPF0283 membrane protein YcjF	8,499	39392,203	8,743	2		84785	236521	20,3	17,2	2,79	0,00	0,00
B1XCE9_ECODH	*ycjX*	Conserved protein with nucleoside triphosphate	10,108	52609,484	8,246	3		58917	154640	24,9	13,5	2,62	0,00	0,00
B1XD92_ECODH	*ydbK*	Pyruvate-flavodoxin oxidoreductase	5,111	128824,45	5,523	5		352694	244305	7,3	10,8	0,69	0,00	0,00
B1XDC7_ECODH	*ydcF*	Conserved protein	14,286	29705,711	5,501	3		251940	196069	4,5	10,1	0,78	0,00	0,01
B1XDE2_ECODH	*ydcL*	Predicted lipoprotein	11,261	24426,971	8,401	1		91790	71248	28,0	23,5	0,78	0,23	0,32
B1XE86_ECODH	*yddB*	Predicted porin protein	4,051	89282,883	6,006	2	transport	36372	157959	2,2	7,8	4,34	0,00	0,00
B1XE89_ECODH	*ydeN*	Conserved protein	41,071	62802,266	5,67	12		3070302	522657	2,3	10,2	0,17	0,00	0,00
B1XEC6_ECODH	*ydfG*	L-allo-threonine dehydrogenase, NAD(P)-binding	19,758	27248,932	5,652	4		2030177	2095091	14,0	14,2	1,03	0,76	0,81
B1XEC8_ECODH	*ydfZ*	Conserved protein	34,328	7276,347	7,941	2		223801	25843	24,4	55,7	0,12	0,00	0,01
B1XF79_ECODH	*ydgA*	Conserved protein	28,486	54688,996	5,068	10		3120119	1975490	8,0	7,6	0,63	0,00	0,00
B1XF69_ECODH	*ydgH*	Uncharacterized protein	37,261	33903,293	9,271	10		2119249	1044882	6,0	11,1	0,49	0,00	0,00
B1XF90_ECODH	*ydgT*	Predicted regulator	53,521	8416,578	6,249	3		199028	99008	12,6	20,1	0,50	0,00	0,01
B1XFV8_ECODH	*ydhF*	Predicted oxidoreductase	21,477	33675,648	5,765	4		971607	921759	7,0	23,4	0,95	0,69	0,75
B1XFX4_ECODH	*ydhQ*	Conserved protein	16,986	42876,008	4,423	6		770461	2181234	8,7	35,9	2,83	0,00	0,01
B1XFX5_ECODH	*ydhR*	Putative mono-oxygenase	32,673	11287,857	5,085	3		324672	434395	34,6	7,9	1,34	0,12	0,19
B1XFZ3_ECODH	*ydiH*	Uncharacterized protein	13,483	10453,968	6,398	1		13464	15566	36,1	25,8	1,16	0,54	0,62
B1XGM6_ECODH	*ydjA*	Predicted oxidoreductase	28,415	20059,006	6,314	4		789739	764402	9,1	3,9	0,97	0,54	0,62
B1XGJ0_ECODH	*ydjN*	Predicted transporter	5,832	48661,691	9,059	2		115129	133969	4,9	22,0	1,16	0,23	0,33
B1XGN7_ECODH	*yeaC*	Conserved protein	18,889	10338,001	5,232	1		77101	78178	12,7	34,7	1,01	0,94	0,95
B1XGP0_ECODH	*yeaD*	Conserved protein	35,374	32666,096	5,89	7		2312174	2291747	15,7	7,6	0,99	0,92	0,94
B1XGP3_ECODH	*yeaG*	Conserved protein with nucleoside triphosphate	11,491	74480,336	5,627	6		351129	320051	14,9	17,6	0,91	0,45	0,54
B1XGP7_ECODH	*yeaK*	Conserved protein	21,557	17850,643	6,509	3		224411	165683	14,1	17,3	0,74	0,04	0,08
B1XGQ2_ECODH	*yeaO*	Conserved protein	10,435	13386,214	6,29	1		30542	49426	35,3	9,3	1,62	0,06	0,11
B1XGR6_ECODH	*yeaY*	Predicted lipoprotein	22,28	20921,123	9,55	3		194671	238632	26,5	17,9	1,23	0,24	0,34
B1XH75_ECODH	*yeaZ*	Predicted peptidase	30,736	25180,812	5,016	4		230089	243963	7,1	14,7	1,06	0,48	0,56
B1XHD2_ECODH	*yebC*	Probable transcriptional regulatory protein YebC	12,195	26422,561	4,714	2		1338810	853803	15,1	1,3	0,64	0,00	0,00
B1XHB4_ECODH	*yebE*	Conserved protein	38,356	23686,846	5,343	5	unknown	217522	4301151	9,9	10,9	19,77	0,00	0,00
B1XHB5_ECODH	*yebF*	Protein YebF	27,049	13464,311	8,806	2		26061	22264	35,8	20,3	0,85	0,46	0,56
B1XHB6_ECODH	*yebG*	Conserved protein regulated by LexA	45,833	10717,01	4,352	2		215975	273973	7,5	10,4	1,27	0,01	0,03
B1XHC1_ECODH	*yebK*	Predicted DNA-binding transcriptional regulator	11,073	31975,572	6,181	2		91936	66620	5,6	14,6	0,72	0,01	0,02
B1XHA0_ECODH	*yebR*	Conserved protein	19,126	20277,213	4,676	3		353129	349734	21,0	10,1	0,99	0,93	0,94
B1XHA4_ECODH	*yebV*	Uncharacterized protein	24,359	8752,796	4,465	1		106328	62939	6,7	40,1	0,59	0,05	0,09
B1X669_ECODH	*yecA*	Conserved metal-binding protein	21,267	25039,277	4,523	3		393274	333918	11,2	7,8	0,85	0,06	0,11
B1X673_ECODH	*yecF*	Uncharacterized protein	21,622	8238,546	5,25	1		110143	46875	15,9	32,8	0,43	0,00	0,01
B1X664_ECODH	*yecJ*	Uncharacterized protein	48,193	9122,583	4,884	3		499018	448716	11,3	13,4	0,90	0,26	0,36
B1XHE3_ECODH	*yecM*	Predicted metal-binding enzyme	13,83	21205,014	5,325	2		46462	19767	9,0	29,5	0,43	0,00	0,01
B1X685_ECODH	*yedD*	Uncharacterized protein	31,387	14983,258	4,867	3		709504	643881	4,8	11,6	0,91	0,17	0,25
B1X6C8_ECODH	*yeeN*	Probable transcriptional regulatory protein YeeN	29,412	25867,105	4,713	5		1094623	1114290	9,6	12,6	1,02	0,84	0,87
B1X6T6_ECODH	*yeeR*	CP4-44 prophage predicted membrane protein	5,49	57236,668	5,773	2		48752	4288	23,6	82,6	0,09	0,01	0,02
B1X6U3_ECODH	*yeeX*	Conserved protein	33,588	15132,504	9,161	2		447143	409248	5,8	54,0	0,92	0,71	0,77

B1X6V2_ECODH	*yeeZ*	Predicted epimerase, with NAD(P)-binding Ross	22,993	29679,943	5,278	4		278428	422961	5,9	63,2	1,52	0,19	0,28
B1X7G5_ECODH	*yegD*	Predicted chaperone	11,333	49371,391	5,113	3		190912	124758	14,9	10,8	0,65	0,00	0,01
B1X7H6_ECODH	*yegP*	Uncharacterized protein	22,727	12024,296	9,435	2		134352	65601	20,1	14,5	0,49	0,00	0,00
B1X7H7_ECODH	*yegQ*	Predicted peptidase	8,609	51193,184	5,797	4		364504	460726	9,9	13,3	1,26	0,03	0,06
B1X7M0_ECODH	*yehZ*	Predicted transporter subunit: periplasmic-bindin	14,426	32609,303	5,816	2		44570	50682	28,6	20,3	1,14	0,53	0,62
SFGH2_ECODH	*yeiG*	S-formylglutathione hydrolase YeiG	10,432	31259,279	5,177	1		30547	48963	16,9	8,7	1,60	0,00	0,01
EFPL_ECODH	*yeiP*	Elongation factor P-like protein	10	21532,631	4,924	2		305831	329107	17,2	19,3	1,08	0,61	0,68
B1X7N5_ECODH	*yeiT*	Predicted oxidoreductase	6,553	44329,465	5,265	2		169205	27963	19,5	22,3	0,17	0,00	0,00
B1X872_ECODH	*yeiU*	Undecaprenyl pyrophosphate phosphatase	9,283	26759,127	9,88	2		332271	335114	2,0	17,8	1,01	0,93	0,94
NDPA_ECODH	*yejK*	Nucleoid-associated protein YejK	24,179	37822,516	4,876	6		1106731	1114065	7,2	12,0	1,01	0,93	0,94
YEJL_ECODH	*yejL*	UPF0352 protein YejL	36	8288,458	5,497	1		31537	70702	50,9	78,5	2,24	0,22	0,31
B1X902_ECODH	*yfbQ*	Predicted aminotransferase	17,284	45517,371	5,847	5		339815	683712	10,5	9,7	2,01	0,00	0,00
B1X904_ECODH	*yfbT*	Predicted hydrolase or phosphatase	16,667	23007,521	5,739	2		95159	81690	9,6	13,9	0,86	0,12	0,19
YFBU_ECODH	*yfbU*	UPF0304 protein YfbU	52,439	19536,203	6,065	9		1830132	2452237	8,5	11,7	1,34	0,01	0,02
B1X910_ECODH	*yfcD*	Predicted NUDIX hydrolase	42,778	20375,861	4,698	5		494240	216044	15,4	40,6	0,44	0,01	0,02
B1X911_ECODH	*yfcE*	Predicted phosphatase	28,804	20122,078	5,632	3		147535	211178	23,8	41,7	1,43	0,16	0,24
B1X936_ECODH	*yfcL*	Uncharacterized protein	55,435	10000,043	4,26	3		185904	254346	13,8	34,2	1,37	0,14	0,21
YFCN_ECODH	*yfcN*	UPF0115 protein YfcN	14,754	21013,318	8,405	1		121849	96147	16,8	14,6	0,79	0,08	0,13
B1X9L6_ECODH	*yfcZ*	Conserved protein	27,66	10317,643	4,25	2		3341617	1166760	6,6	18,0	0,35	0,00	0,00
B1X9M3_ECODH	*yfdH*	CPS-53 (KpLE1) prophage bactoprenol glucosyl	22,549	34635,434	7,784	5		688769	486768	3,8	8,6	0,71	0,00	0,00
B1X9M4_ECODH	*yfdI*	CPS-53 (KpLE1) prophage predicted inner mem	9,255	51483,586	8,792	3		136034	97777	18,5	25,0	0,72	0,08	0,14
B1X9Q1_ECODH	*yfdZ*	Prediected aminotransferase, PLP-dependent	14,563	46216,215	7,71	3		221355	319469	15,5	10,1	1,44	0,01	0,02
B1X9R8_ECODH	*yfeD*	Predicted DNA-binding transcriptional regulator	12,308	14910,144	7,768	1		44921	16618	5,8	40,4	0,37	0,00	0,01
B1XAA2_ECODH	*yfeX*	Conserved protein	8,696	33052,266	5,336	2		321952	236206	11,3	21,2	0,73	0,06	0,11
B1XAA3_ECODH	*yfeY*	Uncharacterized protein	20,942	20897,609	5,213	2		142943	234461	13,8	15,9	1,64	0,00	0,01
B1XAE2_ECODH	*yffB*	Conserved protein	31,356	13600,587	6,091	3		544839	393407	13,1	9,6	0,72	0,01	0,02
B1XAC1_ECODH	*yffS*	Uncharacterized protein	6,093	31041,461	5,453	2		73701	113177	13,2	8,3	1,54	0,00	0,01
B1XAW9_ECODH	*yfgC*	Predicted peptidase	6,982	53907,922	7,084	2		91511	81042	12,9	13,0	0,89	0,23	0,33
B1XAX0_ECODH	*yfgD*	Predicted oxidoreductase	30,252	13398,582	5,895	2		402329	279632	8,0	14,0	0,70	0,00	0,01
B1XAX9_ECODH	*yfgG*	Uncharacterized protein	23,81	7461,619	11,89	1	unknown	75691	207510	16,5	11,1	2,74	0,00	0,00
B1XAY7_ECODH	*yfgL*	Outer membrane protein assembly factor BamB	30,102	41887,211	4,718	7		2051627	3317646	25,0	17,3	1,62	0,02	0,04
B1XAY8_ECODH	*yfgM*	Conserved protein	46,602	22176,055	5,065	6		1921559	2114837	6,3	7,5	1,10	0,10	0,16
B1XAZ9_ECODH	*yfhJ*	Conserved protein	24,242	7731,568	3,982	1		192103	148276	12,1	10,5	0,77	0,02	0,05
B1XAZ5_ECODH	*yfhM*	Conserved protein	2,057	181585,45	5,255	2		55531	39102	18,5	15,4	0,70	0,03	0,06
B1XB07_ECODH	*yfhQ*	Predicted methyltransferase	26,423	27047,938	5,687	5		796558	846964	7,7	10,9	1,06	0,41	0,50
B1XBR9_ECODH	*yfiA*	Cold shock protein associated with 30S ribosom	53,097	12784,585	6,204	4		3639036	6389548	9,3	14,3	1,76	0,00	0,00
B1XBQ8_ECODH	*yfiF*	Predicted methyltransferase	35,072	37784,375	8,936	6		1458535	1478612	26,8	9,9	1,01	0,92	0,94
B1XBR8_ECODH	*yfiO*	Outer membrane protein assembly factor BamD	50,612	27829,396	6,158	10		3272970	5030784	6,4	7,3	1,54	0,00	0,00
B1XBT9_ECODH	*yfjG*	Conserved protein	10,759	17726,67	8,598	1		144174	32138	21,6	43,0	0,22	0,00	0,00
B1XCM7_ECODH	*ygaD*	Conserved protein	25,455	17581,719	5,061	3		300905	280928	9,9	4,8	0,93	0,26	0,35
B1XBX0_ECODH	*ygaU*	Uncharacterized protein	44,295	16063,117	5,708	6		4129999	3371522	14,0	24,2	0,82	0,24	0,33
B1XDI8_ECODH	*ygcF*	7-carboxy-7-deazaguanine synthase	26,457	25029,645	5,71	5		263130	242738	10,2	12,2	0,92	0,36	0,46
B1XDK4_ECODH	*ygdH*	Conserved protein	28,194	50971,891	6,046	8		845030	847917	6,1	5,8	1,00	0,94	0,95
B1XDL8_ECODH	*ygdI*	Uncharacterized protein	26,667	8174,202	5,599	1		1037095	692382	10,0	13,5	0,67	0,00	0,01
B1XDM0_ECODH	*ygdK*	Predicted Fe-S metabolism protein	13,605	15940,271	6,114	1		95264	54869	12,4	29,4	0,58	0,03	0,06
YGFZ_ECODH	*ygfZ*	tRNA-modifying protein YgfZ	27,607	36094,117	5,175	7		1668542	1923698	6,3	15,3	1,15	0,12	0,19
B1XEK5_ECODH	*yggE*	Conserved protein	31,707	26635,264	6,1	7		993479	1163544	7,1	10,2	1,17	0,05	0,09
B1XF98_ECODH	*yggG*	Predicted peptidase	26,19	26842,318	5,748	5		352972	576658	19,3	18,0	1,63	0,01	0,02
B1XFA6_ECODH	*yggJ*	Ribosomal RNA small subunit methyltransferase	19,753	26978,062	6,183	4		212690	279394	12,7	16,0	1,31	0,04	0,08
B1XFB9_ECODH	*yggL*	Uncharacterized protein	12,963	12880,56	4,907	1		70260	27092	25,6	35,2	0,39	0,01	0,03
B1XFB8_ECODH	*yggN*	Uncharacterized protein	8,368	26429,213	8,968	2		49363	135960	57,4	21,1	2,75	0,02	0,04
B1XFB1_ECODH	*yggS*	Predicted enzyme	17,949	25787,436	6,092	3		217451	233864	25,2	13,0	1,08	0,67	0,73
B1XFB4_ECODH	*yggV*	dITP/XTP pyrophosphatase	38,579	21038,844	5,214	4		540275	780792	6,6	20,5	1,45	0,01	0,04
FETP_ECODH	*yggX*	Probable Fe(2+)-trafficking protein	52,747	10952,529	5,914	4		1287392	1158047	9,7	4,1	0,90	0,10	0,16
B1XFF5_ECODH	*yghZ*	Aldo-keto reductase	30,925	38832,16	6,723	6		543947	399190	2,5	13,6	0,73	0,00	0,01
B1XG42_ECODH	*ygiB*	Conserved outer membrane protein	34,555	19816,033	8,251	4	metabolism	419436	1737513	20,0	9,1	4,14	0,00	0,00
B1XG43_ECODH	*ygiC*	Predicted enzyme	37,306	45026,016	4,688	12	metabolism	1201046	4263892	5,9	3,5	3,55	0,00	0,00
B1XG59_ECODH	*ygiF*	Predicted adenylate cyclase	23,095	48388,953	5,731	8		617604	578270	11,1	9,7	0,94	0,41	0,50
B1XG34_ECODH	*ygiN*	Quinol monooxygenase	23,077	11532,391	5,793	3		441010	536234	14,9	4,2	1,22	0,04	0,08
B1XG26_ECODH	*ygiT*	Predicted DNA-binding transcriptional regulator	11,45	14703,1	9,098	1		46100	29485	12,8	19,6	0,64	0,01	0,02
B1XG29_ECODH	*ygiW*	Conserved protein	13,846	14010,758	5,076	2		791540	217367	10,4	16,3	0,27	0,00	0,00
B1XG78_ECODH	*ygjH*	Conserved protein	10	12315,344	5,012	1		41479	24350	20,4	21,5	0,59	0,01	0,03
B1XGS2_ECODH	*yhaJ*	Predicted DNA-binding transcriptional regulator	6,711	33256,406	6,051	2		53888	186056	15,0	12,8	3,45	0,00	0,00
B1XHH6_ECODH	*yhbG*	Predicted transporter subunit: ATP-binding comp	32,78	26800,648	5,64	5		1181701	1177901	9,1	7,7	1,00	0,96	0,96
YHBJ_ECODH	*yhbJ*	UPF0042 nucleotide-binding protein YhbJ	25	32492,285	6,724	4		243183	415153	9,4	17,9	1,71	0,00	0,01
B1XHH5_ECODH	*yhbN*	Predicted transporter subunit: periplasmic-bindin	10,27	20126,885	8,962	2		237541	129611	14,2	33,2	0,55	0,01	0,03
B1XGZ0_ECODH	*yhbY*	Predicted RNA-binding protein	21,649	10783,711	9,415	2		489384	572745	51,7	14,1	1,17	0,61	0,68
B1XHK6_ECODH	*yhcB*	Conserved protein	52,273	14960,849	5,648	5		3517374	4449281	9,3	9,7	1,26	0,01	0,03
B1XHM3_ECODH	*yhdH*	Predicted oxidoreductase, Zn-dependent and NA	31,173	34723,766	5,631	7		1357557	1109284	4,6	4,1	0,82	0,00	0,00
B1X6J5_ECODH	*yheO*	Predicted DNA-binding transcriptional regulator	21,667	26820,564	5,395	3		200797	287941	17,7	28,7	1,43	0,09	0,15
B1X6K2_ECODH	*yheS*	Fused predicted transporter subunits of ABC sup	16,797	71843,43	5,5	9		521565	740662	17,3	12,0	1,42	0,01	0,04
B1X754_ECODH	*yhgF*	Predicted transcriptional accessory protein	23,933	85119,867	5,918	15		2283843	2953648	8,0	11,2	1,29	0,01	0,02
B1X7Q6_ECODH	*yhhA*	Conserved protein	34,932	16623,82	10,89	3		130234	251667	24,5	27,3	1,93	0,01	0,03
B1X7S3_ECODH	*yhhF*	Ribosomal RNA small subunit methyltransferase	12,626	21677,604	5,959	2		94998	107766	12,6	20,9	1,13	0,35	0,45

B1X7R7_ECODH	*yhhK*	Conserved protein	29,921	14505,534	6,585	2		97698	79365	5,8	24,3	0,81	0,15	0,22
B1X7S5_ECODH	*yhhM*	Conserved protein	36,134	13496,499	10,06	3		150822	234158	11,8	24,7	1,55	0,02	0,04
B1X7P8_ECODH	*yhhX*	Predicted oxidoreductase with NAD(P)-binding R	29,275	38765,008	6,066	7		671157	710225	9,4	6,2	1,06	0,36	0,46
B1X7V4_ECODH	*yhiR*	Ribosomal RNA large subunit methyltransferase	13,571	31941,707	8,589	2		180634	190299	12,5	15,2	1,05	0,63	0,70
B1X8E5_ECODH	*yhjJ*	Predicted zinc-dependent peptidase	18,474	55527,371	5,726	6		569682	618619	13,0	3,0	1,09	0,27	0,36
B1X8G7_ECODH	*yiaD*	Predicted outer membrane lipoprotein	52,968	22197,221	9,81	5		2315098	3389091	15,9	8,5	1,46	0,01	0,02
B1X8G9_ECODH	*yiaF*	Conserved protein	27,966	25663,102	6,079	5		1312388	1417238	11,3	13,7	1,08	0,41	0,50
B1X8K7_ECODH	*yibF*	Predicted glutathione S-transferase	7,426	22545,061	5,101	2		35358	40755	3,4	41,7	1,15	0,52	0,61
B1X8L7_ECODH	*yibL*	Conserved protein	18,333	13696,007	9,485	2		160437	205648	9,7	6,9	1,28	0,01	0,02
B1X945_ECODH	*yibN*	Predicted rhodanese-related sulfurtransferase	26,573	15596,255	9,372	2		1667743	1940717	8,4	18,3	1,16	0,18	0,26
B1X8L6_ECODH	*yibT*	Uncharacterized protein	42,029	7995,298	9,395	2		525235	388456	11,7	14,7	0,74	0,02	0,04
B1X977_ECODH	*yicC*	Conserved protein	17,422	33174,926	5,1	5		747717	865030	10,1	8,5	1,16	0,07	0,12
B1X988_ECODH	*yicH*	Conserved protein	11,599	62272,441	5,673	5		310727	398465	17,5	5,2	1,28	0,03	0,06
B1X9S7_ECODH	*yidA*	Predicted hydrolase	12,593	29721,139	5,108	2		318100	259811	12,6	9,2	0,82	0,04	0,08
B1X9S8_ECODH	*yidB*	Conserved protein	34,848	13786,576	4,368	2		148142	121121	2,4	10,5	0,82	0,01	0,03
YIDC_ECODH	*yidC*	Membrane protein insertase YidC	30,657	61525,922	7,7	8		4344267	4426013	10,1	6,2	1,02	0,76	0,81
B1X9U2_ECODH	*yieF*	Chromate reductase, Class I, flavoprotein	50,532	20375,562	5,007	5		527387	589206	6,7	17,2	1,12	0,28	0,37
B1X9Y3_ECODH	*yifE*	Conserved protein	36,607	13133,577	6,102	3		374353	269617	28,2	25,6	0,72	0,15	0,23
B1XAH5_ECODH	*yifL*	Predicted lipoprotein	46,269	7177,217	8,889	2		63781	143907	50,6	30,5	2,26	0,03	0,06
B1XAH8_ECODH	*yigB*	Predicted hydrolase	12,605	27121,842	5,985	2	metabolism	102810	226566	33,7	16,2	2,20	0,01	0,02
B1XAI5_ECODH	*yigI*	Conserved protein	12,258	17162,771	6,423	2		46434	53266	7,2	21,1	1,15	0,27	0,36
B1XAJ1_ECODH	*yigL*	Predicted hydrolase	28,947	29707,729	5,228	4		330567	289887	4,7	5,1	0,88	0,01	0,03
B1XAM1_ECODH	*yihA*	Probable GTP-binding protein EngB	12,121	22083,229	6,769	2		174956	174821	21,4	11,4	1,00	0,99	0,99
B1XAL5_ECODH	*yihD*	Conserved protein	49,438	10272,91	5,179	3		676075	704090	22,1	34,1	1,04	0,85	0,88
B1XAL6_ECODH	*yihE*	Predicted kinase	10,976	38120,316	4,993	3	stress response	160189	517858	51,5	6,7	3,23	0,00	0,01
B1XB77_ECODH	*yiiM*	Conserved protein	14,732	25342,725	5,709	2		143451	205803	18,8	15,2	1,43	0,03	0,05
B1XC02_ECODH	*yjaG*	Conserved protein	22,449	22612,648	4,446	4		415926	438764	8,3	12,7	1,05	0,50	0,58
B1XCT3_ECODH	*yjbJ*	Predicted stress response protein	17,391	8325,252	5,441	1		877523	148472	17,5	17,8	0,17	0,00	0,00
B1XCU5_ECODH	*yjbR*	Conserved protein	26,271	13519,475	6,056	3		160853	158764	9,9	15,1	0,99	0,89	0,91
B1XD15_ECODH	*yjdC*	Predicted transcriptional regulator	27,749	21931,078	4,947	4		166313	258729	17,0	12,4	1,56	0,01	0,02
B1XDR9_ECODH	*yjeE*	ATPase with strong ADP affinity	17,647	16853,078	4,488	2		236724	208807	15,8	26,5	0,88	0,47	0,56
B1XDP8_ECODH	*yjeI*	Conserved protein	30,769	11958,425	5,497	2		1203742	1546862	9,6	11,5	1,29	0,02	0,04
B1XDR3_ECODH	*yjeP*	Predicted mechanosensitive channel	2,439	123967,61	6,551	2		12597	47461	53,8	46,5	3,77	0,01	0,02
YJGA_ECODH	*yjgA*	UPF0307 protein YjgA	15,301	21359,279	5,3	2		173882	170319	6,3	29,8	0,98	0,89	0,91
B1XEM4_ECODH	*yjgF*	Ketoacid-binding protein	64,844	13611,593	5,361	4		5335110	5746979	23,7	14,6	1,08	0,62	0,69
B1XEN1_ECODH	*yjgK*	Conserved protein	22,667	16865,359	5,308	2		190708	255673	11,2	23,6	1,34	0,06	0,11
B1XEP1_ECODH	*yjgQ*	Conserved inner membrane protein	8,056	39618,605	9,48	2		96734	92598	18,9	17,6	0,96	0,77	0,81
B1XEP2_ECODH	*yjgR*	Predicted ATPase	6	54332,293	5,915	2		14306	25574	48,2	25,9	1,79	0,09	0,16
B1XEQ6_ECODH	*yjhC*	KpLE2 phage-like element predicted oxidoreduct	14,247	41384,488	6,102	4	metabolism	502335	1129099	8,2	14,5	2,25	0,00	0,00
B1XER9_ECODH	*yjhU*	KpLE2 phage-like element predicted DNA-bindin	5,488	36111,586	6,326	2		48932	54294	14,2	29,0	1,11	0,60	0,67
B1XFK1_ECODH	*yjjK*	Fused predicted transporter subunits of ABC sup	38,018	62442,883	5,431	17		8036433	7838854	5,3	3,8	0,98	0,47	0,56
B1XE48_ECODH	*ykgE*	Predicted oxidoreductase	10,46	26004,295	7,501	2		226379	46601	15,8	26,6	0,21	0,00	0,00
B1XE49_ECODH	*ykgF*	Predicted amino acid dehydrogenase with NAD(I	8,211	53051,898	8,749	3		274944	39883	20,3	17,1	0,15	0,00	0,00
B1XE50_ECODH	*ykgG*	Predicted transporter	22,511	25212,707	4,963	3		488072	99919	10,9	25,7	0,20	0,00	0,00
B1X7X9_ECODH	*yliJ*	Predicted glutathione S-transferase	30,288	23713,178	5,05	5		240328	279760	6,8	20,9	1,16	0,22	0,31
B1X8Q6_ECODH	*ymbA*	Conserved protein	28,877	20634,406	5,807	3		261215	235080	8,3	17,0	0,90	0,32	0,42
B1X9F9_ECODH	*ymdB*	O-acetyl-ADP-ribose deacetylase	12,429	18880,385	5,445	1		24623	20710	21,9	25,4	0,84	0,39	0,49
B1XBP6_ECODH	*ymjA*	Uncharacterized protein	30,864	9321,317	4,234	1		53187	39285	16,5	18,4	0,74	0,06	0,10
B1XCF9_ECODH	*ynaI*	Conserved inner membrane protein	19,242	38754,508	9,363	3		172184	133403	17,8	20,0	0,77	0,12	0,19
B1XDG0_ECODH	*yncB*	NADPH-dependent curcumin/dihydrocurcumin re	7,536	37609,746	5,526	2		201744	131469	8,2	17,5	0,65	0,01	0,02
B1XDG2_ECODH	*yncD*	Predicted iron outer membrane transporter	4,714	77260,672	5,321	2		42293	38280	48,3	27,5	0,91	0,74	0,80
B1XDG3_ECODH	*yncE*	Conserved protein	29,745	38612,957	9,22	7		696278	1202235	7,3	16,3	1,73	0,00	0,00
YNFB_ECODH	*ynfB*	UPF0482 protein YnfB	10,619	12908,668	9,15	1		161289	187751	73,9	61,3	1,16	0,79	0,83
B1XF51_ECODH	*ynfD*	Uncharacterized protein	21,782	10476,697	4,735	1	unknown	45566	137365	23,5	21,6	3,01	0,00	0,00
B1XFY6_ECODH	*ynhG*	Conserved protein	7,784	36082,348	9,422	2		50678	26356	22,0	25,0	0,52	0,01	0,02
B1XGI6_ECODH	*yniA*	Predicted phosphotransferase/kinase	24,476	32458,617	4,979	4		269427	241496	3,4	20,6	0,90	0,34	0,44
B1XGI8_ECODH	*yniC*	Predicted hydrolase	18,018	24330,24	4,797	3		304529	212879	12,6	7,3	0,70	0,00	0,01
B1XH77_ECODH	*yoaB*	Conserved protein	21,93	12493,228	4,964	2		800535	865443	11,9	15,3	1,08	0,45	0,54
B1XH78_ECODH	*yoaC*	Uncharacterized protein	11,765	13231,266	5,156	1		15203	7202	39,4	97,6	0,47	0,25	0,35
B1XGQ3_ECODH	*yoaF*	Conserved outer membrane protein	16,667	8942,377	7,661	1		39825	60655	25,3	18,9	1,52	0,05	0,09
B1XAA5_ECODH	*ypeA*	Acetyltransferase YpeA	11,348	16311,566	4,835	1		28598	41899	47,9	38,0	1,47	0,32	0,42
B1XAE7_ECODH	*ypfJ*	Conserved protein	14,634	31460,186	5,578	3		141642	111360	27,6	31,5	0,79	0,29	0,39
YQFB_ECODH	*yqfB*	UPF0267 protein YqfB	17,476	11905,412	4,682	1		93092	96407	28,8	25,1	1,04	0,86	0,89
YQGE_ECODH	*yqgE*	UPF0301 protein YqgE	43,85	20685,906	5,343	5		368095	553218	18,7	8,6	1,50	0,01	0,02
RUVX_ECODH	*yqgF*	Putative Holliday junction resolvase	27,536	15186,305	6,741	2		89144	80394	2,4	21,2	0,90	0,38	0,47
B1XFG3_ECODH	*yqhD*	Alcohol dehydrogenase, NAD(P)-dependent	35,917	42097,023	5,72	9		2026968	2291508	12,3	13,7	1,13	0,23	0,33
B1XG47_ECODH	*yqiC*	Conserved protein	16,667	11275,98	5,895	2		203730	363331	38,3	19,0	1,78	0,03	0,06
B1XGA2_ECODH	*yqjD*	Conserved protein	25,743	11051,492	9,046	2		1389828	1091146	16,8	14,7	0,79	0,08	0,14
B1XG74_ECODH	*yqjH*	Predicted siderophore interacting protein	17,717	28871,555	5,532	2	transport	125739	311940	31,9	29,2	2,48	0,01	0,02
B1XG75_ECODH	*yqjI*	Predicted transcriptional regulator	10,145	23401,289	6,266	2		81011	108544	23,3	15,1	1,34	0,08	0,13
B1XGV8_ECODH	*yraL*	Ribosomal RNA small subunit methyltransferase	15,385	31347,904	5,828	2		123003	142866	9,0	31,3	1,16	0,37	0,47
B1XGV9_ECODH	*yraM*	Penicillin-binding protein activator LpoA	28,171	72825,164	5,265	10		1087232	1142017	3,9	6,6	1,05	0,25	0,34
B1XGW2_ECODH	*yraP*	Uncharacterized protein	26,702	20027,949	9,038	3		794735	783649	11,8	12,8	0,99	0,88	0,90
B1XHG5_ECODH	*yrbA*	Predicted DNA-binding transcriptional regulator	29,762	9451,736	5,831	2		351802	367339	15,5	8,3	1,04	0,63	0,70

B1XHG7_ECODH	*yrbC*	Predicted ABC-type organic solvent transporter	37,915	23962,611	9,354	5	256809	593593	32,9	20,0	2,31	0,00	0,01
B1XHG8_ECODH	*yrbD*	Predicted ABC-type organic solvent transporter	44,809	19576,223	4,787	6	1268656	1801949	6,5	4,8	1,42	0,00	0,00
B1XHH0_ECODH	*yrbF*	Predicted toluene transporter subunit: ATP-bindi	31,599	29096,805	6,157	6	402623	578725	5,4	20,2	1,44	0,01	0,03
B1X6D2_ECODH	*yrdA*	Conserved protein	44,565	20245,023	5,259	6	807223	996656	7,7	8,8	1,23	0,01	0,03
B1XAJ4_ECODH	*ysgA*	Predicted hydrolase	16,605	29425,211	5,569	3	46688	61783	17,6	52,9	1,32	0,32	0,42
B1XDV6_ECODH	*ytfB*	Predicted cell envelope opacity-associated prote	13,679	23505,5	5,063	2	122088	140143	22,0	7,6	1,15	0,29	0,39
B1XDX1_ECODH	*ytfP*	Conserved protein	11,504	12866,424	6,391	1	120010	98001	10,5	9,3	0,82	0,03	0,06
ZAPA_ECODH	*zapA*	Cell division protein ZapA	33,945	12594,17	5,217	3	422337	452517	14,1	4,1	1,07	0,36	0,46
ZAPB_ECODH	*zapB*	Cell division protein ZapB	50,617	9634,807	4,69	3	2708556	4029128	9,8	24,4	1,49	0,03	0,06
ZAPD_ECODH	*zapD*	Cell division protein ZapD	17,004	28291,629	6,31	3	466448	442904	6,4	6,0	0,95	0,28	0,38
B1X7S7_ECODH	*zntA*	Zinc, cobalt and lead efflux system	14,344	76839,875	5,697	7	420791	319283	7,3	4,8	0,76	0,00	0,00
B1XHC5_ECODH	*znuA*	Zinc transporter subunit: periplasmic-binding cor	14,194	33777,375	5,608	2	170179	246189	14,4	24,3	1,45	0,03	0,07
B1XCT4_ECODH	*zur*	DNA-binding transcriptional repressor, Zn(II)-bin	11,111	19254,168	5,974	1	23993	52185	39,2	66,2	2,18	0,10	0,16
B1XHC0_ECODH	*zwf*	Glucose-6-phosphate 1-dehydrogenase	39,511	55704,441	5,558	16	3585782	4783716	5,9	8,5	1,33	0,00	0,01
