# Supplementary material for: Molecular and proteome analyses highlight the importance of the Cpx envelope stress system for acid stress and cell wall stability in Escherichia coli
Source: Microbiologyopen. 2016 Apr 2;5(4):582–96. doi: 10.1002/mbo3.353 (PMC4985592; doi:10.1002/mbo3.353)
Supplement: Supplementary file 5 [file MBO3-5-582-s005.pdf]

Table S3B: Proteome profile of *E. coli* and an isogenic *cpxRA* mutant grown under wild-type (WT) and Cpx-inducing (ON, by *nlpE*-overexpression) conditions.

Table S3 is subdivided in the following three tables: Table S3A describes the proteome profiling of WT_{ON} compared to WT, S3B the proteome profiling of *cpxRA* compared to WT, and S3C the proteome profiling of $cpxRA_{ON}$ compared to WT. For all four conditions (WT, WT_{ON}, *cpxRA*, $cpxRA_{ON}$) geometric means of median-normalized protein intensities (normalization performed with Genedata Analyst 8.2 (Genedata, Basel, Switzerland) from four independent biological replicates are displayed for all detected proteins, which were identified with at least two peptides, or when the sequence coverage exceeded 10%, only one peptide was identified, respectively. Further, ratios relative to the WT, p-values from a student's t-test, and multiple testing-corrected q-values according to Benjamini-Hochberg (BH) were determined using Genedata Analyst. Proteins whose intensities resulted in q-values <0.05 (highlighted green) and exceeded an absolute fold change of 2 were regarded as significantly different in the compared conditions. Ratios >2 were highlighted in red, those <0.5 were highlighted in blue. Also the coefficient of variance (CV) between the four replicates for each condition was determined.

Uniprot identifier	gene name	description	protein coverage [%]	MW [Da]	pI	number of peptides	category according to Figure 2	geometric mean median normalized intensity WT	geometric mean median normalized intensity Δ*cpxRA*	CV [%] normalized intensity WT	[CV%] median normalized intensity ΔcpxRA	ratio Δ*cpxRA*/WT	Δ*cpxRA*/WT p-value	Δ*cpxRA*/WT q-value (BH)
ACCA_ECODH	*accA*	Acetyl-coenzyme A carboxylase carboxyl transferase su	40,752	35241,539	5,762	9		3006460	2718218	7,3	3,6	0,90	0,05	0,08
B1XHM4_ECODH	*accB*	Acetyl CoA carboxylase, BCCP subunit	23,718	16687,211	4,658	3		3198997	3925790	14,9	10,8	1,23	0,06	0,10
B1XHM5_ECODH	*accC*	Acetyl-CoA carboxylase, biotin carboxylase subunit	37,194	49320,738	6,655	14		7467437	6053742	7,1	3,0	0,81	0,00	0,01
ACCD_ECODH	*accD*	Acetyl-coenzyme A carboxylase carboxyl transferase su	20,066	33321,891	7,578	5		2347406	1885281	12,1	4,4	0,80	0,02	0,03
B1XC14_ECODH	*aceA*	Isocitrate lyase	62,903	47521,566	5,161	17	metabolism	29467616	5265933	8,4	3,6	0,18	0,00	0,00
B1XC13_ECODH	*aceB*	Malate synthase	37,899	60273,574	5,386	14		5541986	1062681	9,1	7,6	0,19	0,00	0,00
B1XC89_ECODH	*aceE*	Pyruvate dehydrogenase E1 component	50,169	99668,484	5,457	33		83823832	60555982	4,4	3,9	0,72	0,00	0,00
B1XC90_ECODH	*aceF*	Pyruvate dehydrogenase, dihydrolipoyltransacetylase co	55,873	66096,07	5,09	22		43025454	30822965	6,2	3,0	0,72	0,00	0,00
B1X907_ECODH	*ackA*	Acetate kinase	53,25	43290,453	5,846	12		16194575	17071928	8,8	10,0	1,05	0,47	0,54
B1XBM7_ECODH	*acnA*	Aconitate hydratase 1	32,66	97647,195	5,593	18		3515428	2649835	2,5	1,6	0,75	0,00	0,00
B1XC93_ECODH	*acnB*	Aconitate hydratase 2	46,59	93498,109	5,239	28		21519467	14430453	5,9	2,3	0,67	0,00	0,00
ACP_ECODH	*acpP*	Acyl carrier protein	32,051	8639,52	3,976	2		14209819	18338015	17,6	17,1	1,29	0,08	0,12
B1XFQ0_ECODH	*acrA*	Multidrug efflux system	35,264	42196,707	7,69	9		2290160	2112338	7,8	5,2	0,92	0,14	0,20
B1XFP9_ECODH	*acrB*	Multidrug efflux system protein	24,023	113573,648	5,387	17		3259625	2988063	13,6	3,2	0,92	0,25	0,32
B1XCV5_ECODH	*acs*	Acetyl-coenzyme A synthetase	27,454	72093,586	5,501	12		1177069	677833	3,4	14,3	0,58	0,00	0,00
ADD_ECODH	*add*	Adenosine deaminase	46,246	36397,461	5,363	10		2001165	2887011	4,3	6,1	1,44	0,00	0,00
ADEC_ECODH	*ade*	Adenine deaminase	7,993	63739,359	5,234	3		218890	133517	12,4	14,8	0,61	0,00	0,01
B1XAT1_ECODH	*adhE*	Fused acetaldehyde-CoA dehydrogenase and iron-depe	45,342	96127,242	6,324	29		24277271	26458040	4,9	6,1	1,09	0,07	0,11
KAD_ECODH	*adk*	Adenylate kinase	39,252	23586,016	5,551	7		8848699	9452231	11,3	6,0	1,07	0,33	0,40
B1X9C3_ECODH	*agp*	Glucose-1-phosphatase/inositol phosphatase	23,729	45682,91	5,484	6		1662520	1025429	9,6	5,7	0,62	0,00	0,00
B1X603_ECODH	*ahpC*	Alkyl hydroperoxide reductase, C22 subunit	52,941	20761,443	5,027	7		11698272	21014024	34,3	8,5	1,80	0,02	0,03
B1X604_ECODH	*ahpF*	Alkyl hydroperoxide reductase, F52a subunit, FAD/NAD(	30,518	56177,109	5,469	12		3126874	2778725	3,9	4,1	0,89	0,01	0,01
SYA_ECODH	*alaS*	Alanine--tRNA ligase	43,95	96032,398	5,533	29		11421869	17380780	6,4	2,3	1,52	0,00	0,00
B1XDC8_ECODH	*aldA*	Aldehyde dehydrogenase A, NAD-linked	48,434	52272,789	5,067	16		9433873	4204160	6,4	3,6	0,45	0,00	0,00
B1X8K4_ECODH	*aldB*	Aldehyde dehydrogenase B	15,039	56306,352	5,439	5		468445	214569	8,4	16,9	0,46	0,00	0,00
B1XGA4_ECODH	*allR*	DNA-binding transcriptional repressor	15,498	29269,666	5,773	3		154760	157711	23,7	11,9	1,02	0,89	0,91
B1XAA6_ECODH	*amiA*	N-acetylmuramoyl-l-alanine amidase I	11,419	31412,129	10,098	2	peptidoglycan-modification	68826	56369	9,2	13,0	0,82	0,05	0,08
B1X6C7_ECODH	*amn*	AMP nucleosidase	9,711	53994,914	5,897	3		217383	364225	18,9	3,9	1,68	0,00	0,01
ANMK_ECODH	*anmK*	Anhydro-N-acetylmuramic acid kinase	8,401	39496,277	5,699	2		102438	88973	7,5	7,9	0,87	0,04	0,07
B1XFB7_ECODH	*ansB*	Periplasmic L-asparaginase II	32,759	36850,691	5,956	8		4701317	230187	7,5	8,8	0,05	0,00	0,00
APT_ECODH	*apt*	Adenine phosphoribosyltransferase	43,169	19858,885	5,258	5		2344898	1613086	4,9	3,8	0,69	0,00	0,00
B1XFL1_ECODH	*arcA*	DNA-binding response regulator in two-component regu	30,672	27292,016	5,212	5		1831233	1787861	14,6	4,1	0,98	0,76	0,81
B1XHI5_ECODH	*arcB*	Hybrid sensory histidine kinase in two-component regula	3,985	87982,906	4,986	2		89952	77263	9,4	9,7	0,86	0,07	0,11
ARGE_ECODH	*argE*	Acetylornithine deacetylase	15,927	42347,344	5,538	4		192832	343226	2,2	6,3	1,78	0,00	0,00
ASSY_ECODH	*argG*	Argininosuccinate synthase	15,884	49898,398	5,23	4		231952	205045	10,0	11,6	0,88	0,16	0,22
ARLY_ECODH	*argH*	Argininosuccinate lyase	8,753	50318,223	5,108	2		68060	86119	14,0	16,0	1,27	0,08	0,12
ARGR_ECODH	*argR*	Arginine repressor	13,462	16994,521	4,928	1		179581	189906	23,2	12,4	1,06	0,67	0,72
SYR_ECODH	*argS*	Arginine--tRNA ligase	34,662	64682,961	5,321	16		4001860	3500075	6,0	2,4	0,87	0,01	0,01
AROA_ECODH	*aroA*	3-phosphoshikimate 1-carboxyvinyltransferase	24,122	46095,781	5,366	8		439700	691917	6,3	5,2	1,57	0,00	0,00
AROB_ECODH	*aroB*	3-dehydroquinate synthase	25,967	38880,988	5,721	6		560425	538631	6,7	8,4	0,96	0,49	0,56
AROC_ECODH	*aroC*	Chorismate synthase	5,54	39137,445	5,81	2		62898	54179	17,7	15,2	0,86	0,24	0,31
AROD_ECODH	*aroD*	3-dehydroquinate dehydratase	14,683	27466,654	5,19	3		77831	145831	12,8	3,1	1,87	0,00	0,00
AROE_ECODH	*aroE*	Shikimate dehydrogenase	18,382	29413,639	5,349	3		97079	98015	18,6	11,1	1,01	0,93	0,94
B1XBS3_ECODH	*aroF*	Phospho-2-dehydro-3-deoxyheptonate aldolase	11,798	38803,961	5,423	2		95023	93741	12,2	9,5	0,99	0,86	0,89
B1X785_ECODH	*aroG*	Phospho-2-dehydro-3-deoxyheptonate aldolase	30	38009,523	6,138	7		1208585	1816705	4,0	4,4	1,50	0,00	0,00
B1XG11_ECODH	*aroH*	Phospho-2-dehydro-3-deoxyheptonate aldolase	9,77	38735,055	6,423	3		104772	140870	21,5	8,1	1,34	0,04	0,07
AROK_ECODH	*aroK*	Shikimate kinase 1	21,965	19538,047	5,263	3		1327601	1166308	18,5	4,0	0,88	0,21	0,27
B1X804_ECODH	*artI*	Arginine transporter subunit periplasmic-binding compor	32,922	26929,557	5,792	5		613296	1793156	13,4	5,8	2,92	0,00	0,00
B1X778_ECODH	*asd*	Aspartate-semialdehyde dehydrogenase	35,15	40017,883	5,369	8		3218349	4440960	4,2	2,5	1,38	0,00	0,00
ASNA_ECODH	*asnA*	Aspartate--ammonia ligase	19,091	36650,555	5,453	5		421725	386242	12,1	3,5	0,92	0,22	0,28
B1X6K7_ECODH	*asnB*	Asparagine synthetase	18,953	62659,012	5,55	6		572270	807010	10,1	5,3	1,41	0,00	0,00
B1X8N4_ECODH	*asnS*	Asparagine--tRNA ligase	43,991	52570,441	5,172	17		11837356	10333704	4,2	3,9	0,87	0,00	0,01
B1XD19_ECODH	*aspA*	Aspartate ammonia-lyase	37,657	52356,129	5,191	13		17083751	3016426	6,4	2,1	0,18	0,00	0,00
B1X8N2_ECODH	*aspC*	Aspartate aminotransferase, PLP-dependent	55,051	43573,359	5,544	17		6576795	9936793	4,6	1,7	1,51	0,00	0,00
SYD_ECODH	*aspS*	Aspartate--tRNA ligase	44,915	65913,445	5,472	19		10042086	6637299	9,3	4,7	0,66	0,00	0,00
ASTC_ECODH	*astC*	Succinylornithine transaminase	11,084	43665,375	5,913	2		188320	70786	10,8	14,8	0,38	0,00	0,00
ATPA_ECODH	*atpA*	ATP synthase subunit alpha	50,097	55222,078	5,796	17		27712376	24906803	4,4	5,5	0,90	0,02	0,04
ATPB_ECODH	*atpD*	ATP synthase subunit beta	73,913	50325,422	4,902	21		50264832	40459765	5,7	2,7	0,80	0,00	0,00
ATPF_ECODH	*atpF*	ATP synthase subunit b	46,795	17263,957	5,991	6		2658358	2604963	11,6	11,5	0,98	0,81	0,85
ATPG_ECODH	*atpG*	ATP synthase gamma chain	41,115	31577,42	8,839	9		4866229	4549004	4,6	5,0	0,93	0,10	0,14

ATPD_ECODH	*atpH*	ATP synthase subunit delta	52,542	19332,225	4,945	7		3502126	2354865	9,8	7,0	0,67	0,00	0,00
B1X8I8_ECODH	*avtA*	Valine-pyruvate aminotransferase	15,588	46711,227	5,65	6		231258	265916	12,4	0,6	1,15	0,08	0,12
B1XDC5_ECODH	*azoR*	FMN-dependent NADH-azoreductase	24,378	21657,717	5,063	3		400931	431418	10,7	13,0	1,08	0,43	0,51
B1X7H5_ECODH	*baeR*	DNA-binding response regulator in two-component regu	47,083	27655,934	5,522	7		282842	313153	8,2	5,9	1,11	0,10	0,14
BAMA_ECODH	*bamA*	Outer membrane protein assembly factor BamA	42,469	90552,766	4,93	26		8280189	9323209	5,1	4,5	1,13	0,01	0,03
B1XAF2_ECODH	*bcp*	Thiol peroxidase, thioredoxin-dependent	64,744	17633,936	5,032	7		4321502	7386732	4,5	5,0	1,71	0,00	0,00
BETB_ECODH	*betB*	NAD/NADP-dependent betaine aldehyde dehydrogenase	11,633	52911,133	5,19	2		197948	25292	18,6	42,3	0,13	0,00	0,00
B1X6I7_ECODH	*bfr*	Bacterioferritin	60,759	18495,027	4,69	7		2682373	3125412	4,9	2,5	1,17	0,00	0,00
B1XEI8_ECODH	*bglA*	6-phospho-beta-glucosidase A	34,447	55360,766	5,577	14		2614121	2688299	3,6	7,8	1,03	0,53	0,60
B1X7M1_ECODH	*bglX*	Beta-D-glucoside glucohydrolase, periplasmic	22,876	83460,047	5,854	12		779213	827487	8,8	7,4	1,06	0,33	0,41
B1XAM7_ECODH	*bipA*	GTP-binding protein	26,359	67355,453	5,16	12		6358882	6808795	7,6	3,9	1,07	0,16	0,22
B1XDQ4_ECODH	*blc*	Outer membrane lipoprotein (Lipocalin)	10,169	19851,607	8,884	2		25506	53580	9,9	9,7	2,10	0,00	0,00
B1XFM3_ECODH	*bolA*	Regulator of penicillin binding proteins and beta lactama	15,238	11993,675	6,187	1		260716	412640	21,1	15,4	1,58	0,02	0,04
B1XBX7_ECODH	*btuB*	Vitamin B12 transporter BtuB	19,055	68407,148	5,226	8		935209	893840	6,3	2,3	0,96	0,22	0,28
B1XG17_ECODH	*btuE*	Glutathione peroxidase	47,541	20469,557	4,812	6		739373	1172564	11,7	4,4	1,59	0,00	0,00
B1XD12_ECODH	*cadA*	Lysine decarboxylase 1	20,28	81260,125	5,914	10	acid stress response	723120	958866	21,6	6,3	1,33	0,05	0,08
B1XCA1_ECODH	*can*	Carbonic anhydrase	21,364	25096,768	6,164	4		1849930	2215326	12,7	5,1	1,20	0,04	0,07
B1XBF8_ECODH	*carB*	Carbamoyl-phosphate synthase large chain	14,632	117841,719	5,221	11		1163861	846105	44,1	12,0	0,73	0,18	0,24
CBPA_ECODH	*cbpA*	Curved DNA-binding protein	11,765	34455,008	6,327	2		568691	625874	6,8	11,6	1,10	0,21	0,27
CDD_ECODH	*cdd*	Cytidine deaminase	55,782	31539,869	5,419	9		3010913	1846927	5,8	6,4	0,61	0,00	0,00
B1XFX1_ECODH	*cfa*	Cyclopropane fatty acyl phospholipid synthase (Unsatura	9,686	43909,004	5,694	3		164741	411559	19,3	10,4	2,50	0,00	0,00
B1X7P3_ECODH	*cirA*	Ferric iron-catecholate outer membrane transporter	13,876	73895,727	5,106	5	transport	69349	84501	23,5	13,3	1,22	0,20	0,26
B1X6W4_ECODH	*cld*	Regulator of length of O-antigen component of lipopolys	31,595	36454,746	5,427	6		1197243	1064036	13,3	3,7	0,89	0,14	0,20
B1X823_ECODH	*clpA*	ATPase and specificity subunit of ClpA-ClpP ATP-deper	22,296	84206,93	5,911	13		1740778	1807363	6,2	4,2	1,04	0,35	0,43
B1XBR5_ECODH	*clpB*	Protein disaggregation chaperone	47,841	95585,023	5,367	30		18857061	19026948	3,1	1,4	1,01	0,62	0,68
B1XFM5_ECODH	*clpP*	ATP-dependent Clp protease proteolytic subunit	25,121	23186,648	5,523	4		3108536	3387965	15,9	5,0	1,09	0,33	0,41
CLPS_ECODH	*clpS*	ATP-dependent Clp protease adapter protein ClpS	32,075	12179,059	4,936	2		36012	36866	12,5	28,8	1,02	0,88	0,90
CLPX_ECODH	*clpX*	ATP-dependent Clp protease ATP-binding subunit ClpX	47,642	46356,008	5,236	12		4844448	4596268	3,0	2,6	0,95	0,04	0,07
KCY_ECODH	*cmk*	Cytidylate kinase	26,432	24746,342	5,559	4		421511	402639	10,6	4,7	0,96	0,45	0,53
CMOA_ECODH	*cmoA*	tRNA (cmo5U34)-methyltransferase	37,652	27776,645	5,357	6		741466	575450	4,3	8,3	0,78	0,00	0,00
COAA_ECODH	*coaA*	Pantothenate kinase	12,025	36359,777	6,325	2		142844	97407	9,4	5,8	0,68	0,00	0,00
B1XA30_ECODH	*cobB*	NAD-dependent protein deacylase	17,204	31464,039	7,788	3		221146	273250	12,9	5,1	1,24	0,02	0,04
B1XAI1_ECODH	*corA*	Magnesium/nickel/cobalt transporter	39,873	36589,711	4,636	8		995570	1327312	6,6	4,0	1,33	0,00	0,00
B1X653_ECODH	*corC*	Predicted ion transport	6,507	33298,488	4,55	2		178215	153134	14,4	9,7	0,86	0,13	0,18
B1XG37_ECODH	*cpdA*	3',5'-cyclic adenosine monophosphate phosphodiesteras	13,818	30937,996	5,543	3		145325	115385	17,8	10,0	0,79	0,06	0,09
B1XDW3_ECODH	*cpdB*	2':3'-cyclic-nucleotide 2'-phosphodiesterase	27,202	70832,195	5,45	9		1337862	979765	9,6	4,9	0,73	0,00	0,00
B1XB80_ECODH	*cpxP*	Periplasmic protein combats stress	27,711	18965,246	6,379	3	stress response	65948	12186	8,5	48,1	0,18	0,00	0,01
B1XB79_ECODH	*cpxR*	DNA-binding response regulator in two-component regu	25,431	26312,113	5,393	5	stress response	383597	75674	30,9	5,8	0,20	0,00	0,00
B1XFK7_ECODH	*creA*	Protein CreA	29,936	17107,891	9,037	3		193212	215116	9,2	15,8	1,11	0,27	0,34
CRL_ECODH	*crl*	Sigma factor-binding protein Crl	22,556	15654,994	6,316	3		567209	752032	9,1	4,9	1,33	0,00	0,00
B1X705_ECODH	*crp*	DNA-binding transcriptional dual regulator	42,381	23640,426	8,376	8		5370359	5167760	9,4	4,1	0,96	0,48	0,55
B1XA88_ECODH	*crr*	Glucose-specific enzyme IIA component of PTS	58,58	18251,068	4,734	6		16009474	19217791	8,1	3,4	1,20	0,01	0,02
B1X8H1_ECODH	*cspA*	Major cold shock protein	31,429	7403,278	5,578	2		1432668	1116904	12,5	10,1	0,78	0,02	0,04
B1XH90_ECODH	*cspC*	Stress protein, member of the CspA-family	63,768	7402,368	6,539	3		4674281	3582803	7,9	14,1	0,77	0,02	0,03
B1X821_ECODH	*cspD*	Cold shock protein-like protein	55,405	7968,975	5,808	2		1596597	1120864	23,8	7,4	0,70	0,03	0,06
B1X620_ECODH	*cspE*	DNA-binding transcriptional repressor	53,623	7463,451	8,087	3		11926045	12933512	8,1	8,7	1,08	0,23	0,29
B1X5Z5_ECODH	*cstA*	Carbon starvation protein	6,134	75105,234	8,01	3		212933	56081	14,1	15,1	0,26	0,00	0,00
B1XC98_ECODH	*cueO*	Multicopper oxidase (Laccase)	12,791	56556,152	6,28	3		83284	196499	19,8	28,9	2,36	0,00	0,01
CUTA_ECODH	*cutA*	Divalent-cation tolerance protein CutA	22,321	12331,083	4,852	1		35065	83955	26,3	9,1	2,39	0,00	0,00
CYAY_ECODH	*cyaY*	Protein CyaY	41,509	12231,413	4,241	3		40103	170819	33,7	24,9	4,26	0,00	0,00
B1XDV8_ECODH	*cycA*	D-alanine/D-serine/glycine transporter	5,745	51659,656	8,918	2		75310	70534	9,4	9,2	0,94	0,35	0,43
B1X6R3_ECODH	*cydA*	Cytochrome d terminal oxidase, subunit I	17,816	58205,082	6,353	8	acid stress response	7613775	10968969	4,8	4,0	1,44	0,00	0,00
B1X6R4_ECODH	*cydB*	Cytochrome d terminal oxidase, subunit II	5,805	42453,117	6,901	2	acid stress response	1673172	2771626	6,0	11,5	1,66	0,00	0,00
B1X826_ECODH	*cydC*	Fused cysteine transporter subunits of ABC superfamily	11,344	62920,168	8,754	4		399104	298355	8,6	8,4	0,75	0,00	0,01
B1X827_ECODH	*cydD*	Fused cysteine transporter subunits of ABC superfamily	12,925	65056,191	5,997	4		295663	266644	6,1	11,8	0,90	0,18	0,24
B1XFM0_ECODH	*cyoA*	Cytochrome o ubiquinol oxidase subunit II	33,333	34911,297	6,758	6		7281483	4226803	14,6	7,3	0,58	0,00	0,00
B1XFL9_ECODH	*cyoB*	Cytochrome o ubiquinol oxidase subunit I	7,994	74367,906	6,698	5		1797954	1445211	15,8	8,4	0,80	0,05	0,08
B1XBM4_ECODH	*cysB*	DNA-binding transcriptional dual regulator, O-acetyl-L-se	14,815	36150,469	6,873	4		182232	191371	10,6	10,8	1,05	0,54	0,61
B1X941_ECODH	*cysE*	Serine acetyltransferase	20,513	29316,639	6,05	3		265597	290427	19,5	12,6	1,09	0,50	0,57
B1XA85_ECODH	*cysK*	Cysteine synthase	45,82	34489,652	5,829	11		5522376	7019601	4,9	3,8	1,27	0,00	0,00
SYC_ECODH	*cysS*	Cysteine--tRNA ligase	35,792	52202,086	5,326	13		1917801	2739050	19,2	6,6	1,43	0,01	0,03
B1XBA0_ECODH	*cytR*	DNA-binding transcriptional dual regulator	11,437	37819,781	6,015	2		64038	48852	12,4	17,4	0,76	0,04	0,07
B1X627_ECODH	*dacA*	D-alanyl-D-alanine carboxypeptidase (Penicillin-binding p	28,288	44443,957	8,31	9		1799978	1444983	10,4	6,4	0,80	0,01	0,02
B1X7Y0_ECODH	*dacC*	D-alanyl-D-alanine carboxypeptidase (Penicillin-binding p	43,25	43608,906	7,738	12	peptidoglycan-modificatio	1663586	1462759	20,2	10,2	0,88	0,28	0,35
DADA_ECODH	*dadA*	D-amino acid dehydrogenase small subunit	40,278	47607,293	6,174	12		2723001	2063280	5,7	7,4	0,76	0,00	0,00
B1X735_ECODH	*damX*	Uncharacterized protein	10,748	46161,91	5,687	3		694871	828138	13,9	7,9	1,19	0,07	0,10
B1XAF0_ECODH	*dapA*	4-hydroxy-tetrahydrodipicolinate synthase	34,247	31269,971	5,981	7		2699024	3315300	3,9	3,4	1,23	0,00	0,00
DAPB_ECODH	*dapB*	4-hydroxy-tetrahydrodipicolinate reductase	11,355	28756,613	5,446	3		356577	388262	9,4	4,9	1,09	0,17	0,23
DAPD_ECODH	*dapD*	2,3,4,5-tetrahydropyridine-2,6-dicarboxylate N-succinyltr	34,672	29892,098	5,563	8		11244404	11970262	7,3	5,9	1,06	0,23	0,30
DAPE_ECODH	*dapE*	Succinyl-diaminopimelate desuccinylase	21,867	41269,012	5,307	6		364110	308012	9,5	5,5	0,85	0,02	0,04
DAPF_ECODH	*dapF*	Diaminopimelate epimerase	8,029	30208,562	5,856	2		258757	247224	31,7	11,2	0,96	0,82	0,85
B1XEC5_ECODH	*dcp*	Dipeptidyl carboxypeptidase II	26,872	77515,75	5,488	12		1449804	1876044	6,4	6,1	1,29	0,00	0,00
B1X7T0_ECODH	*dcrB*	Periplasmic protein	23,784	19787,492	5,085	3		2705675	3032611	11,0	4,6	1,12	0,10	0,15
B1XD18_ECODH	*dcuA*	Anaerobic C4-dicarboxylate transporter	3,926	45750,707	7,629	2		413352	175018	3,2	11,1	0,42	0,00	0,00

B1XEX0_ECODH	*ddlA*	D-alanine--D-alanine ligase	35,714	39315,809	5,02	9		2049956	1895133	4,3	4,8	0,92	0,05	0,08
B1XC69_ECODH	*ddlB*	D-alanine--D-alanine ligase	17,974	32839,707	4,772	4		780643	617654	11,7	7,1	0,79	0,02	0,03
B1XGX4_ECODH	*deaD*	ATP-dependent RNA helicase DeaD	29,253	70546,156	8,746	14		4234079	2597794	7,2	2,2	0,61	0,00	0,00
DEF_ECODH	*def*	Peptide deformylase	15,976	19328,422	5,229	2		416736	564171	18,5	12,8	1,35	0,05	0,08
B1XD31_ECODH	*degP*	Serine endoprotease (Protease Do), membrane-associa	41,772	49354,23	8,648	13	degradation/folding	5493300	4812221	7,4	6,0	0,88	0,03	0,06
B1XHK7_ECODH	*degQ*	Serine endoprotease, periplasmic	18,022	47205,055	5,763	4		371799	504501	8,2	2,8	1,36	0,00	0,00
TYPH_ECODH	*deoA*	Thymidine phosphorylase	62,727	47207,121	5,214	19		3638898	40145671	6,8	3,2	11,03	0,00	0,00
DEOB_ECODH	*deoB*	Phosphopentomutase	60,934	44369,961	5,106	17		8957027	47110767	4,2	4,7	5,26	0,00	0,00
DEOC_ECODH	*deoC*	Deoxyribose-phosphate aldolase	61,004	27733,801	5,496	12		6588487	81160625	3,5	4,5	12,32	0,00	0,00
DEOD_ECODH	*deoD*	Purine nucleoside phosphorylase DeoD-type	64,435	25949,91	5,418	11		10616246	45981807	5,8	6,2	4,33	0,00	0,00
DER_ECODH	*der*	GTPase Der	21,837	55035,875	5,596	8		1452154	1292284	4,4	7,8	0,89	0,04	0,07
B1X973_ECODH	*dfp*	Fused 4'-phosphopantothenoylcysteine decarboxylase p	22,414	43438,141	7,056	5		321417	302674	20,0	6,6	0,94	0,62	0,68
B1XAP1_ECODH	*dhaK*	Dihydroxyacetone kinase, N-terminal domain	16,854	38215,207	4,815	3		1678691	1026917	7,0	11,1	0,61	0,00	0,00
B1XAP0_ECODH	*dhaL*	Dihydroxyacetone kinase, C-terminal domain	58,571	22631,746	5,309	7		1185980	988995	15,4	12,4	0,83	0,12	0,17
B1XAN9_ECODH	*dhaM*	Fused predicted dihydroxyacetone-specific PTS enzyme	31,78	51448,621	4,612	9		1628995	1351696	7,3	4,9	0,83	0,01	0,01
B1XC56_ECODH	*djlA*	DnaJ-like protein DjlA	8,118	30579,363	9,744	2		117821	91550	2,5	7,9	0,78	0,00	0,00
B1XFG4_ECODH	*dkgA*	2,5-diketo-D-gluconate reductase A	22,182	31109,637	5,999	5		739654	1267055	15,5	4,9	1,71	0,00	0,00
B1XD71_ECODH	*dkgB*	2,5-diketo-D-gluconate reductase B	8,989	29436,791	5,492	2		84454	129065	30,9	9,3	1,53	0,06	0,09
B1XCC0_ECODH	*dksA*	RNA polymerase-binding transcription factor DksA	23,841	17527,752	5,063	3		4180235	3550211	13,9	11,9	0,85	0,13	0,18
B1X7M2_ECODH	*dld*	D-lactate dehydrogenase	24,343	64612,332	6,19	11		755846	894172	3,5	8,4	1,18	0,01	0,02
B1X834_ECODH	*dmsA*	Dimethyl sulfoxide reductase, anaerobic, subunit A	9,459	90398,617	6,42	6		579529	75203	8,9	6,3	0,13	0,00	0,00
B1XCU0_ECODH	*dnaB*	Replicative DNA helicase	10,828	52390,082	4,946	3		310124	230477	5,9	6,0	0,74	0,00	0,00
B1XD53_ECODH	*dnaE*	DNA polymerase III alpha subunit	1,897	129904,57	5,165	2		33528	19495	11,3	20,4	0,58	0,00	0,01
B1XG71_ECODH	*dnaG*	DNA primase	6,885	65564,625	5,685	2		26015	42714	53,7	5,9	1,64	0,16	0,21
DNAJ_ECODH	*dnaJ*	Chaperone protein DnaJ	23,404	41100,332	7,977	7		1431518	2989104	12,8	3,7	2,09	0,00	0,00
B1XBD9_ECODH	*dnaK*	Chaperone protein DnaK	54,859	69114,961	4,831	23		51520712	50765124	3,7	3,0	0,99	0,56	0,63
B1X9T0_ECODH	*dnaN*	DNA polymerase III subunit beta	17,213	40586,602	5,25	4		749729	707755	6,2	3,8	0,94	0,17	0,23
DNAT_ECODH	*dnaT*	Primosomal protein 1	12,849	19455,121	5,145	1		39686	31218	18,1	11,7	0,79	0,07	0,11
B1X8G0_ECODH	*dppA*	Dipeptide transporter periplasmic-binding component of	14,766	60293,672	6,211	4		638159	181909	19,9	21,4	0,29	0,00	0,00
DPS_ECODH	*dps*	DNA protection during starvation protein	66,467	18695,307	5,723	9		16441117	9478237	7,6	6,0	0,58	0,00	0,00
B1XAL7_ECODH	*dsbA*	Thiol:disulfide interchange protein	24,519	23104,537	5,946	3	degradation/folding	611814	968761	11,1	5,0	1,58	0,00	0,00
B1XEI0_ECODH	*dsbC*	Protein disulfide isomerase II	17,797	25621,656	6,296	4		140652	245408	17,6	19,7	1,74	0,01	0,02
B1X602_ECODH	*dsbG*	Periplasmic disulfide isomerase/thiol-disulphide oxidase	12,5	27494,99	8,318	2		138660	161757	4,9	11,2	1,17	0,05	0,08
SDHD_ECODH	*dsdA*	D-serine dehydratase	42,986	47900,57	5,584	11		5072805	2923252	7,6	3,4	0,58	0,00	0,00
B1XCT7_ECODH	*dusA*	tRNA-dihydrouridine synthase	22,899	38467,898	6,116	4		336998	366793	12,0	7,5	1,09	0,29	0,37
DUT_ECODH	*dut*	Deoxyuridine 5'-triphosphate nucleotidohydrolase	32,45	16155,47	5,031	2		965588	1894179	18,1	18,4	1,96	0,00	0,01
B1XD42_ECODH	*dxr*	1-deoxy-D-xylulose 5-phosphate reductoisomerase	5,779	43387,977	5,675	2		45176	43887	21,1	7,4	0,97	0,81	0,85
DXS_ECODH	*dxs*	1-deoxy-D-xylulose-5-phosphate synthase	16,452	67616,938	6,164	8		846079	824824	8,2	1,7	0,97	0,56	0,63
NQOR_ECODH	*DH10B_1(*	NAD(P)H dehydrogenase (quinone)	51,01	20845,555	5,594	7		4789765	5274220	7,0	6,8	1,10	0,09	0,14
CINAL_ECODH	*DH10B_24*	CinA-like protein	20,5	44225,645	5,463	5		255474	506200	14,6	5,6	1,98	0,00	0,00
B1XDQ2_ECODH	*ecnB*	Entericidin B membrane lipoprotein	39,583	4809,528	7,934	1		1924480	2081956	12,3	11,7	1,08	0,40	0,47
ECOT_ECODH	*eco*	Ecotin	43,827	18192,049	6,602	5		1144393	1399451	22,1	11,9	1,22	0,16	0,22
B1XHB8_ECODH	*eda*	Multifunctional 2-keto-3-deoxygluconate 6-phosphate alc	34,272	22284,027	5,569	5		3666416	4644918	12,4	6,8	1,27	0,02	0,04
EFP_ECODH	*efp*	Elongation factor P	17,021	20591,314	4,897	2		2652391	2439875	15,4	3,3	0,92	0,31	0,38
B1X8Y0_ECODH	*elaB*	Conserved protein	27,723	11305,689	5,35	2		727051	2184180	60,3	6,8	3,00	0,01	0,01
B1XHI4_ECODH	*elbB*	Isoprenoid biosynthesis protein with amidotransferase-lil	23,041	22981,551	4,675	3		1498034	1668260	6,9	2,5	1,11	0,02	0,05
B1XCL8_ECODH	*emrA*	Multidrug efflux system	8,205	42736,047	9,333	2		225074	173795	13,0	11,9	0,77	0,03	0,05
EMTA_ECODH	*emtA*	Endo-type membrane-bound lytic murein transglycosylas	35,961	22226,547	9,161	4		313275	307361	6,0	7,2	0,98	0,70	0,76
ENO_ECODH	*eno*	Enolase	52,546	45654,945	5,324	14		57757965	49229130	4,5	1,9	0,85	0,00	0,00
B1X5Z2_ECODH	*entB*	Isochorismatase	8,772	32554,338	5,046	2	metabolism	121152	88966	13,2	16,7	0,73	0,03	0,05
B1X5Z1_ECODH	*entE*	Enterobactin synthetase component E	11,754	59112,152	5,419	3	metabolism	40207	38953	14,3	11,9	0,97	0,75	0,80
E4PD_ECODH	*epd*	D-erythrose-4-phosphate dehydrogenase	14,749	37299,391	6,264	5		230172	389792	4,0	3,5	1,69	0,00	0,00
ERA_ECODH	*era*	GTPase Era	19,934	33810,066	6,73	5		565770	457474	2,8	1,4	0,81	0,00	0,00
ERPA_ECODH	*erpA*	Iron-sulfur cluster insertion protein ErpA	18,421	12100,484	4,109	2		805230	1243452	17,1	10,0	1,54	0,00	0,01
B1XAB2_ECODH	*eutB*	Ethanolamine ammonia-lyase, large subunit, heavy chai	8,168	49403,047	4,789	3		114446	116766	27,6	13,9	1,02	0,90	0,92
B1XFF9_ECODH	*exbB*	Membrane spanning protein in TonB-ExbB-ExbD comple	16,393	26287,387	7,888	3		699985	573920	3,1	6,6	0,82	0,00	0,01
B1XFF8_ECODH	*exbD*	Membrane spanning protein in TonB-ExbB-ExbD comple	13,475	15526,983	4,698	2		50998	58716	23,0	27,0	1,15	0,46	0,53
B1XG98_ECODH	*exuR*	DNA-binding transcriptional repressor	13,953	29835,695	5,395	3		513301	418188	7,3	7,2	0,81	0,01	0,02
FABA_ECODH	*fabA*	3-hydroxydecanoyl-[acyl-carrier-protein] dehydratase	19,186	18969,049	6,126	3		4282581	6222959	9,6	6,1	1,45	0,00	0,00
B1X934_ECODH	*fabB*	3-oxoacyl-[acyl-carrier-protein] synthase I	39,655	42613,32	5,347	10		6604220	18271872	9,1	3,9	2,77	0,00	0,00
B1XA02_ECODH	*fabD*	Malonyl CoA-acyl carrier protein transacylase	45,307	32417,203	4,947	7		10883343	9743046	4,9	6,4	0,90	0,03	0,06
B1XA05_ECODH	*fabF*	3-oxoacyl-[acyl-carrier-protein] synthase 2	33,414	43045,766	5,714	8		3699537	3476829	12,6	2,5	0,94	0,37	0,45
B1XA03_ECODH	*fabG*	3-oxoacyl-[acyl-carrier-protein] reductase	54,508	25560,295	6,763	9		7000366	6038935	11,8	7,7	0,86	0,09	0,13
B1XA01_ECODH	*fabH*	3-oxoacyl-[acyl-carrier-protein] synthase 3	38,801	33515,121	5,078	8		4216170	3569824	4,6	3,7	0,85	0,00	0,00
B1XBN9_ECODH	*fabI*	Enoyl-[acyl-carrier-protein] reductase [NADH]	50,382	27863,939	5,577	9		18240176	18742451	5,5	0,4	1,03	0,38	0,45
FABZ_ECODH	*fabZ*	3-hydroxyacyl-[acyl-carrier-protein] dehydratase FabZ	28,477	17032,953	6,84	4		2510322	2288162	7,1	11,6	0,91	0,21	0,28
FADB_ECODH	*fadB*	Fatty acid oxidation complex subunit alpha	7,682	79593,914	5,838	4	metabolism	116228	207072	10,3	13,2	1,78	0,00	0,00
B1XD83_ECODH	*fadE*	Acyl coenzyme A dehydrogenase	3,931	89224,445	8,221	2		60777	44354	12,8	34,9	0,73	0,17	0,23
B1X9L7_ECODH	*fadL*	Long-chain fatty acid outer membrane transporter	25,223	48771,914	5,086	7		3080747	2012930	7,3	5,1	0,65	0,00	0,00
FADR_ECODH	*fadR*	Fatty acid metabolism regulator protein	30,544	26968,609	6,507	5		315983	203488	16,4	25,0	0,64	0,03	0,05
B1XEK8_ECODH	*fbaA*	Fructose-bisphosphate aldolase, class II	32,869	39147,262	5,524	7		24756889	50772289	7,0	1,9	2,05	0,00	0,00
B1X7I8_ECODH	*fbaB*	Fructose-bisphosphate aldolase class I	43,714	38109,191	6,248	11		2042080	3986601	7,7	4,8	1,95	0,00	0,00
F16PA_ECODH	*fbp*	Fructose-1,6-bisphosphatase class 1	39,157	36833,934	5,665	9		2202928	1955466	4,8	2,3	0,89	0,00	0,01
FDHE_ECODH	*fdhE*	Protein FdhE	8,091	34746,711	5,033	2		269150	221994	9,6	15,5	0,82	0,08	0,12

B1XE65_ECODH	*fdnG*	Formate dehydrogenase-N, alpha subunit, nitrate-induci	33,3	112963,344	6,543	22		5395074	780351	7,1	8,6	0,14	0,00	0,00
B1XE66_ECODH	*fdnH*	Formate dehydrogenase-N, Fe-S (Beta) subunit, nitrate-	14,286	32238,719	5,575	4		501819	89922	16,1	13,7	0,18	0,00	0,00
B1XB62_ECODH	*fdoG*	Formate dehydrogenase-O, large subunit	42,52	112536,414	6,883	25		8587833	5746106	7,5	2,8	0,67	0,00	0,00
B1XB61_ECODH	*fdoH*	Formate dehydrogenase-O, Fe-S subunit	20,667	33100,383	5,166	5		403116	488955	39,9	9,2	1,21	0,34	0,41
B1XB60_ECODH	*fdoI*	Formate dehydrogenase-O, cytochrome b556 subunit	9,005	24606,365	10,21	2		213173	205927	21,3	8,3	0,97	0,78	0,83
B1XB00_ECODH	*fdx*	[2Fe-2S] ferredoxin	55,856	12330,815	4,491	4		1298059	1797362	18,3	9,2	1,38	0,03	0,05
B1XER5_ECODH	*fecA*	KpLE2 phage-like element ferric citrate outer membrane	18,088	85321,609	5,59	10		1755417	1150333	12,0	7,4	0,66	0,00	0,00
B1XER4_ECODH	*fecB*	KpLE2 phage-like element iron-dicitrate transporter subu	9,333	33146,348	8,832	2		174724	176868	11,5	11,3	1,01	0,89	0,91
B1X755_ECODH	*feoA*	Ferrous iron transporter, protein A	21,333	8370,826	9,425	1		39783	30429	25,3	20,5	0,76	0,13	0,18
B1X756_ECODH	*feoB*	Fused ferrous iron transporter, protein B: GTP-binding p	5,304	84473,656	5,81	3		192191	158401	6,5	11,8	0,82	0,03	0,05
B1X5Y0_ECODH	*fepA*	Iron-enterobactin outer membrane transporter	13,137	82106,898	5,386	6	transport	135800	146290	12,6	9,1	1,08	0,39	0,46
B1XBT2_ECODH	*ffh*	Signal recognition particle protein	43,929	49787,305	9,517	14		3437472	3112165	3,9	3,1	0,91	0,01	0,02
B1XCC5_ECODH	*fhuA*	Ferrichrome outer membrane transporter	34,672	82114,953	5,465	15		1829618	1948069	2,4	5,9	1,06	0,10	0,14
FIS_ECODH	*fis*	DNA-binding protein Fis	32,653	11239,932	9,344	2		5978028	3829797	11,4	7,4	0,64	0,00	0,00
B1XDV7_ECODH	*fklB*	Peptidyl-prolyl cis-trans isomerase	43,204	22216,193	4,849	6		6020863	7176746	7,9	2,7	1,19	0,01	0,01
B1X6J6_ECODH	*fkpA*	Peptidyl-prolyl cis-trans isomerase	27,407	28881,926	8,386	6		3459592	4236171	17,7	8,0	1,22	0,08	0,12
B1XBF3_ECODH	*fkpB*	Peptidyl-prolyl cis-trans isomerase	47,651	16081,006	4,3	3		601267	594033	12,3	6,6	0,99	0,87	0,89
B1X6L7_ECODH	*fldA*	Flavodoxin	38,636	19736,941	4,213	4		783980	1558697	48,0	9,9	1,99	0,03	0,05
B1X678_ECODH	*fliY*	Cystine transporter subunit periplasmic-binding compon	12,406	29039,189	6,216	3		32216	99068	57,4	18,0	3,08	0,00	0,01
B1X6T5_ECODH	*flu*	CP4-44 prophage antigen 43 (Ag43) phase-variable biof	35,515	106825,445	5,777	21		26222767	2480595	11,9	9,5	0,09	0,00	0,00
FMT_ECODH	*fmt*	Methionyl-tRNA formyltransferase	46,032	34168,465	5,559	9		1820144	1717504	11,5	4,5	0,94	0,37	0,44
B1XCG3_ECODH	*fnr*	DNA-binding transcriptional dual regulator, global regula	17,6	27967,207	8,25	3		982941	59813	21,5	14,5	0,06	0,00	0,00
B1XC49_ECODH	*folA*	Dihydrofolate reductase	33,333	17999,377	4,839	3		198435	191571	13,1	10,0	0,97	0,69	0,74
B1XG63_ECODH	*folB*	Bifunctional dihydroneopterin aldolase and dihydroneopt	13,934	13619,531	4,678	2		64434	61480	19,2	9,7	0,95	0,66	0,72
B1X926_ECODH	*folC*	Bifunctional protein FolC	19,194	45405,699	5,502	6		398435	374918	6,8	6,6	0,94	0,25	0,32
FOLD_ECODH	*folD*	Bifunctional protein FolD	13,889	31043,748	5,742	3		271822	352793	19,0	4,3	1,30	0,04	0,06
GCH1_ECODH	*folE*	GTP cyclohydrolase 1	34,234	24830,621	6,796	6		728044	963776	15,4	1,9	1,32	0,02	0,03
B1XGY7_ECODH	*folP*	7,8-dihydropteroate synthase	14,539	30615,111	5,677	3		195235	145909	11,4	16,2	0,75	0,03	0,05
B1X914_ECODH	*folX*	D-erythro-7,8-dihydroneopterin triphosphate 2'-epimeras	31,667	14082,037	6,509	4		947050	1243138	12,0	5,3	1,31	0,01	0,01
B1XB90_ECODH	*fpr*	Ferredoxin-NADP reductase	17,742	27750,938	6,174	2		55055	116367	14,8	3,6	2,11	0,00	0,00
B1XDQ8_ECODH	*frdA*	Fumarate reductase (Anaerobic) catalytic and NAD/flavo	19,934	65971,602	5,863	8		3146135	438438	9,0	15,4	0,14	0,00	0,00
B1XEU9_ECODH	*frmA*	Alcohol dehydrogenase class III/glutathione-dependent f	12,737	39358,992	5,849	4		607613	617791	18,5	14,2	1,02	0,90	0,92
B1XEV0_ECODH	*frmR*	Regulator protein that represses frmRAB operon	26,374	10317,724	5,843	2		122134	105867	15,5	11,8	0,87	0,21	0,27
RRF_ECODH	*frr*	Ribosome-recycling factor	36,216	20638,572	6,436	5		6519852	7928921	14,3	2,8	1,22	0,04	0,06
FRSA_ECODH	*frsA*	Esterase FrsA	17,874	47008,77	6,465	5		237580	348406	27,1	5,2	1,47	0,05	0,08
B1X865_ECODH	*fruA*	Fused fructose-specific PTS enzymes: IIBcomponent IIC	8,526	57519,113	8,911	4		533297	34299	17,1	17,2	0,06	0,00	0,00
B1X866_ECODH	*fruK*	Phosphofructokinase	22,115	33755,688	5,372	5		1080046	135564	11,8	25,2	0,13	0,00	0,00
B1X666_ECODH	*ftn*	Ferritin iron storage protein (Cytoplasmic)	27,879	19423,816	4,768	3		2302928	1522357	8,1	8,3	0,66	0,00	0,00
B1XC71_ECODH	*ftsA*	Cell division protein ftsA	31,905	45329,969	5,845	6		770387	1054479	23,6	15,8	1,37	0,07	0,11
FTSB_ECODH	*ftsB*	Cell division protein FtsB	13,592	11622,075	6,568	1		15271	68025	102,8	12,2	4,45	0,01	0,02
B1XGY8_ECODH	*ftsH*	ATP-dependent zinc metalloprotease FtsH	37,422	70708,086	5,907	17		7239062	7332297	7,5	2,6	1,01	0,76	0,81
B1XC61_ECODH	*ftsI*	Transpeptidase involved in septal peptidoglycan synthes	14,116	63877,434	9,644	5		219213	206740	11,8	10,2	0,94	0,50	0,57
B1X830_ECODH	*ftsK*	DNA-binding membrane protein required for chromosom	5,192	146662,719	4,916	3		173804	168246	4,5	8,8	0,97	0,53	0,60
B1XB99_ECODH	*ftsN*	Essential cell division protein	12,853	35793,23	10,17	3		320448	315581	11,5	6,5	0,98	0,82	0,86
B1X7S0_ECODH	*ftsX*	Cell division protein FtsX	9,659	38543,652	9,078	2		85311	129376	23,1	9,3	1,52	0,02	0,04
B1X7S2_ECODH	*ftsY*	Signal recognition particle receptor FtsY	34,004	54513,348	4,471	10		1381534	1440453	13,4	3,7	1,04	0,59	0,65
B1XC72_ECODH	*ftsZ*	Cell division protein FtsZ	53,525	40323,914	4,649	14		8848277	8378980	5,8	3,1	0,95	0,15	0,21
FUCI_ECODH	*fucI*	L-fucose isomerase	5,584	64976,672	5,586	3		287943	48090	17,4	9,0	0,17	0,00	0,00
B1XDK8_ECODH	*fucO*	L-1,2-propanediol oxidoreductase	7,833	40644,562	5,095	2		149165	45094	12,4	14,6	0,30	0,00	0,00
FUCM_ECODH	*fucU*	L-fucose mutarotase	26,429	15473,123	5,57	4		247656	292337	8,3	7,0	1,18	0,02	0,04
B1XF77_ECODH	*fumA*	Fumarate hydratase (Fumarase A), aerobic Class I	20,438	60298,578	6,114	8		5808238	2395631	9,2	5,4	0,41	0,00	0,00
B1XD03_ECODH	*fumB*	Anaerobic class I fumarate hydratase (Fumarase B)	24,453	60105,324	5,88	8		3232194	1061104	5,0	9,4	0,33	0,00	0,00
EFG_ECODH	*fusA*	Elongation factor G	65,199	77581,305	5,236	30		131344424	122891624	11,2	1,0	0,94	0,28	0,35
B1XD20_ECODH	*fxsA*	Inner membrane protein	18,987	17691,945	8,062	2		99455	96276	6,5	9,8	0,97	0,61	0,67
B1XBW7_ECODH	*gabT*	4-aminobutyrate aminotransferase, PLP-dependent	25,822	45774,629	5,778	6		807241	564408	10,9	7,9	0,70	0,00	0,01
B1X7X2_ECODH	*gadA*	Glutamate decarboxylase A, PLP-dependent	48,283	52685,156	5,221	15	acid stress response	27203143	35097084	14,5	6,1	1,29	0,02	0,04
B1XE83_ECODH	*gadC*	Predicted glutamate:gamma-aminobutyric acid antiporte	5,284	55076,672	8,756	3	acid stress response	394920	624498	6,8	6,3	1,58	0,00	0,00
B1X789_ECODH	*galE*	UDP-glucose-4-epimerase	27,811	37325,207	5,887	6		1306312	1022961	13,7	8,2	0,78	0,02	0,04
B1X6X8_ECODH	*galF*	Predicted subunit with GalU	50,842	32829,227	5,733	10		2804253	2762685	5,8	4,1	0,99	0,69	0,75
B1X787_ECODH	*galM*	Aldose 1-epimerase	55,78	38190,457	4,845	12		2798592	2634545	5,1	2,3	0,94	0,07	0,11
B1XAS8_ECODH	*galU*	Glucose-1-phosphate uridylyltransferase	40,066	32942,258	5,108	8		5212777	4442687	4,0	5,8	0,85	0,00	0,01
B1XGN9_ECODH	*gapA*	Glyceraldehyde-3-phosphate dehydrogenase A	69,184	35532,488	6,609	16		107287691	143535240	4,5	1,4	1,34	0,00	0,00
B1XGT8_ECODH	*garR*	Tartronate semialdehyde reductase	20,608	30659,734	5,585	3		244436	78447	16,7	8,7	0,32	0,00	0,00
B1X7I6_ECODH	*gatA*	Galactitol-specific enzyme IIA component of PTS	44,667	16907,457	5,148	5		7059180	3820026	6,3	8,7	0,54	0,00	0,00
B1X7I5_ECODH	*gatB*	Galactitol-specific enzyme IIB component of PTS	63,83	10222,037	5,84	3		11562664	8236021	25,8	14,8	0,71	0,09	0,13
B1X7I4_ECODH	*gatC*	Galactitol-specific enzyme IIC component of PTS	10,421	48364,996	9,319	5		3474304	2250467	8,0	6,2	0,65	0,00	0,00
B1X7I3_ECODH	*gatD*	Galactitol-1-phosphate dehydrogenase, Zn-dependent a	38,15	37390,137	5,939	10		5428339	4585968	4,8	3,5	0,84	0,00	0,00
GATY_ECODH	*gatY*	D-tagatose-1,6-bisphosphate aldolase subunit GatY	33,803	30811,932	5,874	6		9672090	5503282	6,8	6,7	0,57	0,00	0,00
GCSP_ECODH	*gcvP*	Glycine dehydrogenase (decarboxylating)	27,9	104376,352	5,623	16		6939598	10616853	6,1	3,2	1,53	0,00	0,00
B1XGM2_ECODH	*gdhA*	Glutamate dehydrogenase	15,213	48581,375	5,982	5		118377	160545	4,7	3,1	1,36	0,00	0,00
GHRA_ECODH	*ghrA*	Glyoxylate/hydroxypyruvate reductase A	23,397	35343,422	6,317	4		237582	412188	14,6	8,2	1,73	0,00	0,00
GHRB_ECODH	*ghrB*	Glyoxylate/hydroxypyruvate reductase B	26,235	35395,504	5,5	7		860293	1580992	9,3	8,6	1,84	0,00	0,00
B1XFD1_ECODH	*glcB*	Malate synthase G	14,938	80488,516	5,792	9		560147	510578	4,7	5,6	0,91	0,05	0,08
B1XFD2_ECODH	*glcG*	Conserved protein	11,194	13736,714	5,77	1		82445	73886	13,1	15,4	0,90	0,32	0,39

B1XBB1_ECODH	*gldA*	Glycerol dehydrogenase, NAD	24,796	38712,199	4,813	7		704695	145116	4,5	4,9	0,21	0,00	0,00
B1X6X2_ECODH	*glf*	UDP-galactopyranose mutase, FAD/NAD(P)-binding	39,237	42965,883	6,615	12		3475905	2685794	6,0	4,7	0,77	0,00	0,00
GLGA_ECODH	*glgA*	Glycogen synthase	12,998	52822,426	6,145	5		290004	392058	7,2	16,4	1,35	0,01	0,03
B1X777_ECODH	*glgB*	1,4-alpha-glucan branching enzyme GlgB	4,258	84336,898	5,912	2		69647	132076	17,3	10,2	1,90	0,00	0,00
GLGC_ECODH	*glgC*	Glucose-1-phosphate adenylyltransferase	19,722	48697,645	5,774	5		501598	702295	17,8	8,8	1,40	0,02	0,04
B1X773_ECODH	*glgP*	Phosphorylase	6,994	93172,555	5,572	4		145342	161814	57,6	8,4	1,11	0,68	0,74
GLK_ECODH	*glk*	Glucokinase	17,134	34723,086	6,061	5		1239235	1068741	9,1	5,4	0,86	0,03	0,06
GLMM_ECODH	*glmM*	Phosphoglucosamine mutase	40,674	47543,574	5,711	13		3315975	2690748	3,9	3,2	0,81	0,00	0,00
GLMU_ECODH	*glmU*	Bifunctional protein GlmU	21,93	49190,086	6,087	8		708249	989739	8,0	4,1	1,40	0,00	0,00
B1XAM6_ECODH	*glnA*	Glutamine synthetase	47,761	51903,762	5,264	13		5450693	4598732	7,0	6,1	0,84	0,01	0,02
B1XB28_ECODH	*glnB*	Regulatory protein P-II for glutamine synthetase	33,929	12425,448	5,17	3		557122	595700	6,2	14,6	1,07	0,41	0,49
GLNE_ECODH	*glnE*	Glutamate-ammonia-ligase adenylyltransferase	3,383	108418,414	5,078	2		49040	74607	9,9	11,4	1,52	0,00	0,00
B1X7E1_ECODH	*glnH*	Glutamine transporter subunit periplasmic binding comp	45,968	27190,117	8,439	8		939650	1804248	7,7	5,1	1,92	0,00	0,00
SYQ_ECODH	*glnS*	Glutamine--tRNA ligase	33,755	63477,891	5,886	15		7526797	7051914	6,6	6,8	0,94	0,22	0,29
GLO2_ECODH	*gloB*	Hydroxyacylglutathione hydrolase	27,49	28434,379	5,434	4		276486	281350	11,0	8,8	1,02	0,81	0,85
B1X8D5_ECODH	*glpA*	sn-glycerol-3-phosphate dehydrogenase (Anaerobic), lar	35,978	58958,254	6,196	15		5782436	1020403	7,2	6,1	0,18	0,00	0,00
GLPB_ECODH	*glpB*	Anaerobic glycerol-3-phosphate dehydrogenase subunit	27,208	45357,238	5,749	7		2232671	263872	10,4	7,4	0,12	0,00	0,00
B1X8D7_ECODH	*glpC*	sn-glycerol-3-phosphate dehydrogenase (Anaerobic), sm	26,768	44108,039	8,778	9		2226882	387030	6,2	5,2	0,17	0,00	0,00
B1X771_ECODH	*glpD*	sn-glycerol-3-phosphate dehydrogenase, aerobic, FAD/N	59,481	56750,543	6,964	21		24075162	24050022	5,6	3,0	1,00	0,98	0,98
GLPK_ECODH	*glpK*	Glycerol kinase	61,155	56230,766	5,365	23		77451726	45923369	3,6	4,2	0,59	0,00	0,00
B1X8D3_ECODH	*glpQ*	Periplasmic glycerophosphodiester phosphodiesterase	52,514	40843,352	5,377	12		10322805	3290126	5,7	5,3	0,32	0,00	0,00
B1X768_ECODH	*glpR*	DNA-binding transcriptional repressor	8,73	28047,838	5,821	2		143237	120215	5,9	6,9	0,84	0,01	0,02
B1X8D4_ECODH	*glpT*	sn-glycerol-3-phosphate transporter	7,743	50310,387	8,684	3		2169930	635615	18,2	6,9	0,29	0,00	0,00
B1XB91_ECODH	*glpX*	Fructose-1,6-bisphosphatase	16,369	35852,309	5,325	5		436638	259324	6,9	5,3	0,59	0,00	0,00
B1X6Q1_ECODH	*gltA*	Citrate synthase	45,199	48014,992	6,213	14	metabolism	8134109	4907923	4,6	6,2	0,60	0,00	0,00
B1XHI7_ECODH	*gltB*	Glutamate synthase, large subunit	21,49	166709,875	6,265	23		745388	1740190	8,7	2,7	2,33	0,00	0,00
B1XHI8_ECODH	*gltD*	Glutamate synthase, 4Fe-4S protein, small subunit	13,559	52015,273	5,537	4		240841	538183	12,0	6,9	2,23	0,00	0,00
B1X650_ECODH	*gltI*	Glutamate and aspartate transporter subunit, periplasmi	10,265	33420,164	8,61	2		78548	152471	19,2	8,1	1,94	0,00	0,00
SYE_ECODH	*gltX*	Glutamate--tRNA ligase	32,484	53815,727	5,593	12		5652760	4474394	4,1	2,1	0,79	0,00	0,00
GLYA_ECODH	*glyA*	Serine hydroxymethyltransferase	39,089	45316,594	6,028	11		8110992	12207292	6,3	3,7	1,51	0,00	0,00
SYGB_ECODH	*glyS*	Glycine--tRNA ligase beta subunit	49,927	76812,945	5,291	25		9774754	8865552	5,6	3,8	0,91	0,03	0,05
GMHA_ECODH	*gmhA*	Phosphoheptose isomerase	36,458	20814,748	5,966	6		1810557	1688716	4,7	7,1	0,93	0,16	0,21
B1X6W6_ECODH	*gnd*	6-phosphogluconate dehydrogenase, decarboxylating	62,821	51481,309	5,055	20		31560622	43011014	6,1	4,0	1,36	0,00	0,00
B1X8U6_ECODH	*gnsA*	Predicted regulator of phosphatidylethanolamine synthes	40,351	6575,613	5,392	2		1589437	2019771	10,7	6,6	1,27	0,01	0,02
B1XF19_ECODH	*gnsB*	Uncharacterized protein	12,281	6547,669	8,963	1		86146	312905	54,0	17,5	3,63	0,00	0,01
B1X7V5_ECODH	*gor*	Glutathione oxidoreductase	18	48772,516	5,639	5		1098395	1162796	11,7	10,0	1,06	0,49	0,56
B1X732_ECODH	*gph*	Phosphoglycolate phosphatase	22,222	27389,166	4,578	3		419519	427768	12,0	8,3	1,02	0,80	0,84
GPMA_ECODH	*gpmA*	2,3-bisphosphoglycerate-dependent phosphoglycerate m	32,8	28556,404	5,851	7		14240771	13325194	7,5	6,1	0,94	0,22	0,28
B1X946_ECODH	*gpmI*	2,3-bisphosphoglycerate-independent phosphoglycerate	36,381	56193,887	5,141	11		7942175	7220642	5,9	3,6	0,91	0,03	0,06
GPPA_ECODH	*gppA*	Guanosine-5'-triphosphate,3'-diphosphate pyrophosphat	6,275	54871,141	5,946	3		60890	198982	18,4	6,4	3,27	0,00	0,00
GPDA_ECODH	*gpsA*	Glycerol-3-phosphate dehydrogenase [NAD(P)+]	41,298	36361,602	6,085	7		528453	707448	18,7	9,0	1,34	0,03	0,06
XGPT_ECODH	*gpt*	Xanthine phosphoribosyltransferase	42,763	16970,588	5,52	4		458157	419369	2,9	18,8	0,92	0,37	0,44
GRCA_ECODH	*grcA*	Autonomous glycyl radical cofactor	74,016	14284,189	5,087	9		16580981	660294	4,4	14,2	0,04	0,00	0,00
B1XGZ1_ECODH	*greA*	Transcription elongation factor GreA	55,063	17640,957	4,712	7		2075949	1829829	9,0	3,2	0,88	0,04	0,06
CH60_ECODH	*groL*	60 kDa chaperonin	69,708	57328,848	4,845	24		71878789	80571008	7,9	2,0	1,12	0,03	0,06
CH10_ECODH	*groS*	10 kDa chaperonin	51,546	10386,952	5,147	4		20955487	18898955	22,3	6,4	0,90	0,41	0,49
GRPE_ECODH	*grpE*	Protein GrpE	29,949	21797,83	4,681	5		11356294	8158855	8,2	5,1	0,72	0,00	0,00
B1X9H7_ECODH	*grxB*	Glutaredoxin 2	47,442	24349,266	8,717	8		1187584	1583112	6,7	9,0	1,33	0,00	0,01
B1X944_ECODH	*grxC*	Glutaredoxin 3	68,675	9137,494	6,707	4		1657836	3760915	16,1	4,6	2,27	0,00	0,00
B1XFW5_ECODH	*grxD*	Glutaredoxin	46,087	12878,761	4,75	4		5083433	5431450	5,0	1,8	1,07	0,05	0,08
GSH1_ECODH	*gshA*	Glutamate--cysteine ligase	16,409	58269,293	5,273	6		406350	733150	15,9	4,8	1,80	0,00	0,00
B1XFA7_ECODH	*gshB*	Glutathione synthetase	31,646	35560,898	5,109	7		1235538	1463615	7,2	6,8	1,18	0,02	0,03
B1XFR4_ECODH	*gsk*	Inosine/guanosine kinase	14,516	48448,781	5,502	4		200868	161987	15,5	16,2	0,81	0,11	0,16
B1XFE4_ECODH	*gss*	Fused glutathionylspermidine amidase glutathionylsperm	8,562	70531,961	5,13	4		256805	489596	12,9	9,5	1,91	0,00	0,00
B1XFU7_ECODH	*gst*	Glutathionine S-transferase	34,826	22868,369	5,849	5		611195	921994	9,3	9,5	1,51	0,00	0,00
GUAA_ECODH	*guaA*	GMP synthase [glutamine-hydrolyzing]	43,429	58679,223	5,239	16		9029129	10941063	2,1	5,1	1,21	0,00	0,00
B1XAY3_ECODH	*guaB*	Inosine-5'-monophosphate dehydrogenase	46,107	52022,453	6,016	12		7710779	11693384	6,5	6,8	1,52	0,00	0,00
GUAC_ECODH	*guaC*	GMP reductase	24,784	37383,668	6,103	4		819052	1510191	33,6	3,4	1,84	0,02	0,03
B1XCN5_ECODH	*gutQ*	Arabinose 5-phosphate isomerase	20,249	34031,43	5,919	4		206208	172909	8,1	3,9	0,84	0,01	0,02
B1X8C5_ECODH	*gyrA*	DNA gyrase subunit A	27,314	96963,516	5,086	20		7962950	7938091	3,5	2,9	1,00	0,89	0,91
HCHA_ECODH	*hchA*	Molecular chaperone Hsp31 and glyoxalase 3	28,975	31190,463	5,628	6		3534679	4590454	8,2	2,9	1,30	0,00	0,00
B1X7W5_ECODH	*hdeA*	Acid stress chaperone HdeA	54,545	11857,629	5,056	2	acid stress response	534489	3515913	33,2	5,3	6,58	0,00	0,00
B1X7W4_ECODH	*hdeB*	Acid stress chaperone HdeB	43,519	12042,821	5,727	3	acid stress response	554124	5632431	14,6	11,1	10,16	0,00	0,00
B1X7W6_ECODH	*hdeD*	Acid-resistance membrane protein	6,316	20903,369	9,553	2	acid stress response	35149	120398	41,3	21,2	3,43	0,00	0,00
B1XF84_ECODH	*hdhA*	7-alpha-hydroxysteroid dehydrogenase, NAD-dependent	34,902	26778,59	5,216	6		905859	1120186	5,5	4,0	1,24	0,00	0,00
B1XEW0_ECODH	*hemB*	Delta-aminolevulinic acid dehydratase	22,531	35624,793	5,25	5		1491537	1813868	22,0	2,8	1,22	0,13	0,19
HEM3_ECODH	*hemC*	Porphobilinogen deaminase	54,313	33851,836	5,312	10		625231	1203994	13,1	11,6	1,93	0,00	0,00
DCUP_ECODH	*hemE*	Uroporphyrinogen decarboxylase	24,011	39248,117	5,882	6		1004480	965518	10,7	14,8	0,96	0,68	0,73
B1XAL2_ECODH	*hemG*	Protoporphyrin oxidase, flavoprotein	24,862	21226,301	9,678	4		307344	428112	21,3	2,1	1,39	0,02	0,03
GSA_ECODH	*hemL*	Glutamate-1-semialdehyde 2,1-aminomutase	37,793	45366,098	4,733	11		6434240	6380185	8,8	2,3	0,99	0,86	0,89
B1XAM3_ECODH	*hemN*	Coproporphyrinogen-III oxidase	9,409	52729,043	5,7	3		144003	173426	4,0	7,8	1,20	0,01	0,01
B1XAH0_ECODH	*hemY*	Predicted protoheme IX synthesis protein	31,156	45245,023	8,525	8		2341316	1593486	15,5	5,6	0,68	0,00	0,01
B1XDS6_ECODH	*hflC*	Modulator of FtsH protease HflC	35,629	37649,883	6,299	8	degradation/folding	1405943	1183043	9,9	1,8	0,84	0,01	0,03
HFLD_ECODH	*hflD*	High frequency lysogenization protein HflD	15,493	22947,547	9,338	2		202583	116546	9,7	18,2	0,58	0,00	0,01

B1XDS5_ECODH	*hflK*	Modulator for HflB protease specific for phage lambda c	39,618	45544,883	6,189	12	degradation/folding	4133618	4193581	11,5	2,5	1,01	0,81	0,85
B1XDS4_ECODH	*hflX*	GTPase HflX	38,028	48327,102	5,684	11	metabolism	750564	833653	8,4	14,2	1,11	0,27	0,34
HFQ_ECODH	*hfq*	RNA-binding protein Hfq	21,569	11166,386	6,97	2		997738	908145	17,6	15,1	0,91	0,45	0,52
B1XFP7_ECODH	*hha*	Haemolysin expression modulating protein	11,111	8678,061	8,787	1		14814	16548	68,8	29,8	1,12	0,76	0,81
B1XA13_ECODH	*hinT*	Purine nucleoside phosphoramidase	25,21	13241,277	5,731	2		842422	1316725	22,4	4,3	1,56	0,01	0,03
B1X6V9_ECODH	*hisB*	Histidine biosynthesis bifunctional protein HisB	16,338	40277,965	5,763	5		281196	369104	21,5	1,3	1,31	0,05	0,09
HIS6_ECODH	*hisF*	Imidazole glycerol phosphate synthase subunit HisF	14,729	28454,453	5,034	3		103802	104535	9,9	3,8	1,01	0,90	0,92
HIS1_ECODH	*hisG*	ATP phosphoribosyltransferase	27,425	33366,715	5,471	4		246225	233784	20,3	7,2	0,95	0,68	0,73
B1X6W0_ECODH	*hisH*	Imidazole glycerol phosphate synthase subunit HisH	16,327	21652,857	5,335	2		74595	59075	16,2	28,8	0,79	0,21	0,28
B1X6W3_ECODH	*hisI*	Histidine biosynthesis bifunctional protein HisIE	17,241	22755,818	5,245	1		42160	81002	48,8	27,5	1,92	0,11	0,16
B1X920_ECODH	*hisJ*	Histidine/lysine/arginine/ornithine transporter subunit per	26,154	28483,377	5,465	4	transport	332016	870115	10,4	3,2	2,62	0,00	0,00
SYH_ECODH	*hisS*	Histidine--tRNA ligase	44,811	47029,441	5,647	15		5096946	4859771	6,4	4,7	0,95	0,27	0,34
HLDD_ECODH	*hldD*	ADP-L-glycero-D-manno-heptose-6-epimerase	47,742	34893,168	4,8	13		8060830	7550243	9,9	2,5	0,94	0,24	0,30
HLDE_ECODH	*hldE*	Bifunctional protein HldE	37,317	51050,617	5,294	12		2975409	3003522	9,0	4,2	1,01	0,85	0,89
B1XB27_ECODH	*hmp*	Flavohemoprotein	15,152	43867,656	5,484	6		378381	338643	5,0	9,4	0,89	0,09	0,13
B1XAS9_ECODH	*hns*	DNA-binding protein	54,015	15539,633	5,436	6		24184214	20960165	5,6	7,7	0,87	0,02	0,04
B1XHH8_ECODH	*hpf*	Ribosomal hibernation promoting factor	17,895	10750,249	6,5	1		39106	42247	7,7	54,3	1,08	0,80	0,84
B1XCA0_ECODH	*hpt*	Hypoxanthine phosphoribosyltransferase	44,944	20115,238	5,091	7		1190083	863142	12,6	4,1	0,73	0,00	0,01
B1XDC6_ECODH	*hrpA*	ATP-dependent helicase	5,923	149027,922	7,887	6		394218	248056	8,5	9,3	0,63	0,00	0,00
HSCA_ECODH	*hscA*	Chaperone protein HscA	20,942	65652,438	4,983	8		1273386	1079489	6,5	6,0	0,85	0,01	0,02
HSCB_ECODH	*hscB*	Co-chaperone protein HscB	13,45	20137,709	5,048	2		394291	357025	11,3	8,2	0,91	0,21	0,28
B1XD94_ECODH	*hslJ*	Heat-inducible protein	27,143	15165,621	6,721	2		148594	142074	30,5	18,2	0,96	0,83	0,87
HSLO_ECODH	*hslO*	33 kDa chaperonin	29,11	32534,477	4,354	5		1146412	1342212	12,6	6,1	1,17	0,07	0,11
HSLU_ECODH	*hslU*	ATP-dependent protease ATPase subunit HslU	30,926	49593,805	5,239	10		5985088	6316840	3,8	2,7	1,06	0,06	0,09
B1XFR0_ECODH	*htpG*	Chaperone protein HtpG	58,333	71422,531	5,086	28		27381473	24534776	2,1	3,3	0,90	0,00	0,00
HTPX_ECODH	*htpX*	Protease HtpX	16,041	31923,312	6,605	3	degradation/folding	809019	768163	8,4	41,2	0,95	0,79	0,83
B1XG60_ECODH	*htrG*	Predicted signal transduction protein (SH3 domain)	14,078	23076,389	9,096	3		481823	556725	5,9	15,4	1,16	0,15	0,20
B1XC03_ECODH	*hupA*	HU, DNA-binding transcriptional regulator, alpha subunit	57,778	9534,985	9,572	4		30576421	24163166	7,7	5,7	0,79	0,00	0,01
B1XFM8_ECODH	*hupB*	HU, DNA-binding transcriptional regulator, beta subunit	15,556	9225,579	9,694	1		985714	338674	33,1	11,8	0,34	0,00	0,00
B1XFF2_ECODH	*hybA*	Hydrogenase 2 4Fe-4S ferredoxin-type component	9,756	36002,996	7,104	2		74901	8926	29,2	15,8	0,12	0,00	0,00
B1XFF0_ECODH	*hybC*	Hydrogenase 2, large subunit	18,519	62491,16	5,843	6		921428	171902	13,1	10,3	0,19	0,00	0,00
IBPA_ECODH	*ibpA*	Small heat shock protein IbpA	37,226	15773,711	5,574	5		923405	878218	15,3	7,4	0,95	0,56	0,62
IBPB_ECODH	*ibpB*	Small heat shock protein IbpB	37,324	16093,197	5,189	4		364491	311678	19,2	4,4	0,86	0,15	0,21
B1XA46_ECODH	*icd*	Isocitrate dehydrogenase [NADP]	38,702	45784,762	5,163	12		18780688	15969934	10,7	3,4	0,85	0,03	0,05
ICIA_ECODH	*iciA*	Chromosome initiation inhibitor	8,081	33471,688	6,402	2		99231	153671	17,4	13,4	1,55	0,01	0,02
IHFA_ECODH	*ihfA*	Integration host factor subunit alpha	41,414	11353,932	9,342	4		2230837	3103249	6,7	6,6	1,39	0,00	0,00
IHFB_ECODH	*ihfB*	Integration host factor subunit beta	26,596	10651,144	9,344	2		786442	662973	13,6	7,8	0,84	0,07	0,11
SYI_ECODH	*ileS*	Isoleucine--tRNA ligase	44,456	104296,836	5,657	29		12400238	11696245	5,2	2,6	0,94	0,09	0,13
B1X9A4_ECODH	*ilvB*	Acetolactate synthase	16,192	60440,57	5,302	4	metabolism	107900	80400	22,6	9,0	0,75	0,05	0,08
ILVC_ECODH	*ilvC*	Ketol-acid reductoisomerase	10,998	54069,02	5,204	5	metabolism	96932	127469	6,7	4,8	1,32	0,00	0,00
B1X9Y7_ECODH	*ilvE*	Branched-chain amino-acid aminotransferase	14,887	34093,652	5,54	3		191533	457330	11,5	8,1	2,39	0,00	0,00
B1X9A3_ECODH	*ilvN*	Acetolactate synthase I, small subunit	10,417	11105,758	5,69	1	metabolism	14387	17231	42,5	25,4	1,20	0,49	0,56
B1XC55_ECODH	*imp*	LPS-assembly protein LptD	37,372	89671,148	4,936	20		4090624	4552296	5,2	2,8	1,11	0,01	0,02
B1X824_ECODH	*infA*	Translation initiation factor IF-1	27,778	8249,575	9,22	1		162569	161109	30,7	7,0	0,99	0,96	0,97
IF2_ECODH	*infB*	Translation initiation factor IF-2	31,573	97349,898	5,802	20		13352697	10112327	7,5	5,9	0,76	0,00	0,00
IRAP_ECODH	*iraP*	Anti-adapter protein IraP	31,395	9937,478	4,818	1		33265	19269	14,4	12,3	0,58	0,00	0,00
ISCR_ECODH	*iscR*	HTH-type transcriptional regulator IscR	29,012	17336,561	6,824	2		296800	253012	10,1	12,5	0,85	0,10	0,14
ISCS_ECODH	*iscS*	Cysteine desulfurase IscS	46,782	45089,5	5,943	15		9256794	7502287	9,2	3,0	0,81	0,00	0,01
B1XB04_ECODH	*iscU*	Scaffold protein	60,938	13848,59	4,815	5		2880071	3675726	4,7	4,1	1,28	0,00	0,00
B1XF09_ECODH	*ispA*	Geranyltranstransferase	24,08	32159,633	5,273	4		530637	456959	9,9	13,2	0,86	0,13	0,18
ISPF_ECODH	*ispF*	2-C-methyl-D-erythritol 2,4-cyclodiphosphate synthase	19,497	16897,537	6,044	2		240887	340894	32,6	9,7	1,42	0,13	0,18
ISPG_ECODH	*ispG*	4-hydroxy-3-methylbut-2-en-1-yl diphosphate synthase	42,742	40683,613	5,871	14		2177761	1989687	6,4	4,3	0,91	0,06	0,10
ISPH_ECODH	*ispH*	4-hydroxy-3-methylbut-2-enyl diphosphate reductase	13,608	34774,559	5,196	3		399029	329491	11,4	20,8	0,83	0,14	0,19
B1XD82_ECODH	*ivy*	Inhibitor of vertebrate C-lysozyme	35,032	16872,289	6,267	5		252085	1074997	13,2	7,4	4,26	0,00	0,00
B1XGJ3_ECODH	*katE*	Catalase	17,928	84162,617	5,541	9		1264073	1431512	13,5	6,9	1,13	0,16	0,21
KATG_ECODH	*katG*	Catalase-peroxidase	37,328	80023,812	5,139	20		8369889	5906089	5,7	2,6	0,71	0,00	0,00
B1X951_ECODH	*kbl*	Glycine C-acetyltransferase	33,668	43117,039	5,64	10		5273266	5556595	9,3	4,3	1,05	0,35	0,42
B1X8E4_ECODH	*kdgK*	Ketodeoxygluconokinase	16,505	33962,359	4,923	3		570285	391094	10,0	10,4	0,69	0,00	0,01
B1XH95_ECODH	*kdgR*	Predicted DNA-binding transcriptional regulator	38,403	30029,348	5,435	8		981038	720887	7,7	7,0	0,73	0,00	0,00
KDSA_ECODH	*kdsA*	2-dehydro-3-deoxyphosphooctonate aldolase	48,944	30832,693	6,32	11		6363419	6900021	11,0	10,2	1,08	0,33	0,41
KDSB_ECODH	*kdsB*	3-deoxy-manno-octulosonate cytidylyltransferase	35,081	27614,439	5,147	6		920407	922797	9,7	5,0	1,00	0,96	0,97
B1XHH3_ECODH	*kdsC*	3-deoxy-D-manno-octulosonate 8-phosphate phosphata	29,787	19997,113	4,942	3		366882	338438	8,2	13,7	0,92	0,35	0,43
B1XHH2_ECODH	*kdsD*	Arabinose 5-phosphate isomerase	5,793	35196,086	6,219	2		100038	111516	13,3	19,9	1,11	0,44	0,51
B1X967_ECODH	*kdtA*	3-deoxy-D-manno-octulosonic-acid transferase (KDO tra	6,118	47291,082	9,797	2		87220	71161	13,2	6,3	0,82	0,04	0,06
B1XFQ2_ECODH	*kefA*	Fused conserved protein mechanosensitive channel pro	7,679	127215,281	8,045	6		235764	344169	14,2	5,9	1,46	0,00	0,01
B1XBJ2_ECODH	*lacI*	DNA-binding transcriptional repressor	45,278	38590,164	6,39	11		1719418	1920917	10,6	9,0	1,12	0,16	0,22
LAMB_ECODH	*lamB*	Maltoporin	37,892	49912,227	4,807	10		3407953	1314977	3,7	7,3	0,39	0,00	0,00
B1XD95_ECODH	*ldhA*	Fermentative D-lactate dehydrogenase, NAD-dependent	21,581	36534,793	5,285	5		893013	830349	3,7	5,3	0,93	0,06	0,10
LEPA_ECODH	*lepA*	Elongation factor 4	33,556	66570,273	5,401	15		3585084	3030323	8,2	1,3	0,85	0,01	0,02
B1X637_ECODH	*leuS*	Leucine--tRNA ligase	34,767	97233,758	5,156	21		13253120	12135719	3,4	3,3	0,92	0,01	0,02
LEXA_ECODH	*lexA*	LexA repressor	11,386	22357,719	6,229	2		127443	90185	15,1	14,0	0,71	0,01	0,03
LGT_ECODH	*lgt*	Prolipoprotein diacylglyceryl transferase	20,962	33107,949	9,515	3		220032	226022	5,0	21,4	1,03	0,82	0,86
DNLJ_ECODH	*ligA*	DNA ligase	14,158	73606,07	5,385	9		777280	816973	9,2	3,4	1,05	0,33	0,41
B1X623_ECODH	*lipA*	Lipoyl synthase	19,315	36071,715	8,084	5		1921475	1373353	9,9	2,7	0,71	0,00	0,00

LLDD_ECODH	*lldD*	L-lactate dehydrogenase [cytochrome]	34,091	42728,191	6,332	9		3025801	690971	14,2	5,2	0,23	0,00	0,00
LOLA_ECODH	*lolA*	Outer-membrane lipoprotein carrier protein	43,35	22497,055	6,278	6		547191	1247700	7,4	3,9	2,28	0,00	0,00
LOLB_ECODH	*lolB*	Outer-membrane lipoprotein LolB	46,377	23550,77	8,886	7		806114	829080	10,2	3,1	1,03	0,62	0,68
B1XA26_ECODH	*lolC*	Outer membrane-specific lipoprotein transporter subunit	11,028	43263,762	6,043	3		157281	150055	14,3	10,2	0,95	0,61	0,68
B1XFM7_ECODH	*lon*	Lon protease	41,071	87438,125	6,012	22		6651655	7358404	9,8	7,7	1,11	0,16	0,21
B1XC91_ECODH	*lpd*	Dihydrolipoyl dehydrogenase	43,038	50688,492	5,789	15		41789445	29708323	11,6	3,4	0,71	0,00	0,00
LPLA_ECODH	*lplA*	Lipoate-protein ligase A	11,834	37925,883	5,708	2		182005	199269	16,8	17,1	1,09	0,47	0,55
B1XFY5_ECODH	*lpp*	Murein lipoprotein	33,333	8323,479	9,3	2		36947808	36036265	21,7	7,1	0,98	0,82	0,86
LPXA_ECODH	*lpxA*	Acyl-[acyl-carrier-protein]--UDP-N-acetylglucosamine O-	17,557	28079,996	6,63	3		459346	368266	14,5	10,0	0,80	0,05	0,08
B1XD48_ECODH	*lpxD*	UDP-3-O-(3-hydroxymyristoyl)glucosamine N-acyltransfe	22,287	36038,484	6,073	4		808670	739417	9,4	3,7	0,91	0,13	0,18
B1X9G7_ECODH	*lpxL*	Lauryl-acyl carrier protein (ACP)-dependent acyltransfer	11,438	35406,824	9,805	2		73419	71542	19,8	14,3	0,97	0,84	0,87
B1X901_ECODH	*lrhA*	DNA-binding transcriptional repressor of flagellar, motilit	10,897	34593,828	5,238	2	metabolism	122327	92937	13,3	11,7	0,76	0,02	0,03
B1X829_ECODH	*lrp*	DNA-binding transcriptional dual regulator, leucine-bindi	26,22	18886,783	8,904	4		923182	1260152	4,7	5,0	1,37	0,00	0,00
B1X811_ECODH	*ltaE*	L-allo-threonine aldolase, PLP-dependent	16,216	36494,707	5,807	5		334103	426581	11,3	4,9	1,28	0,01	0,02
LUXS_ECODH	*luxS*	S-ribosylhomocysteine lyase	50,877	19416,189	5,181	6		3495988	6648389	9,6	10,8	1,90	0,00	0,00
B1XC23_ECODH	*lysC*	Aspartokinase	7,795	48531,73	5,025	2		42636	52618	17,9	12,1	1,23	0,12	0,17
B1XEH7_ECODH	*lysS*	Lysine--tRNA ligase	41,98	57603,422	5,106	14		5897358	5123585	8,1	3,1	0,87	0,02	0,03
B1XD10_ECODH	*lysU*	Lysine--tRNA ligase	47,327	57826,57	5,104	19		12151150	13214500	6,9	4,4	1,09	0,09	0,13
MAO1_ECODH	*maeA*	NAD-dependent malic enzyme	43,009	63197,328	5,188	16		3789475	3642075	5,4	2,1	0,96	0,21	0,28
B1XAD4_ECODH	*maeB*	Fused malic enzyme predicted oxidoreductase predictec	38,208	82417,383	5,338	20		7557969	3543198	5,0	2,0	0,47	0,00	0,00
B1XEY3_ECODH	*mak*	Manno(Fructo)kinase	11,258	32499,744	5,48	2		139695	127566	16,5	10,0	0,91	0,40	0,47
B1XC33_ECODH	*malE*	Maltose transporter subunit periplasmic-binding compon	37,626	43387,641	5,534	10		5070923	1864939	9,9	19,5	0,37	0,00	0,00
B1XC32_ECODH	*malF*	Maltose transporter subunit membrane component of AE	18,482	57013,402	8,477	5		397771	101076	13,7	8,3	0,25	0,00	0,00
B1XC34_ECODH	*malK*	Fused maltose transport subunit, ATP-binding compone	15,094	40990,453	6,228	4		1041012	363874	6,1	8,4	0,35	0,00	0,00
B1XC36_ECODH	*malM*	Maltose regulon periplasmic protein	14,706	31943,5	7,72	3		1619708	588885	9,3	7,4	0,36	0,00	0,00
B1X763_ECODH	*malP*	Phosphorylase	33,124	90522,398	6,938	18		4169601	2063894	2,8	2,4	0,49	0,00	0,00
B1X762_ECODH	*malQ*	4-alpha-glucanotransferase (Amylomaltase)	10,519	78503,492	6,138	5		327543	150918	4,4	5,8	0,46	0,00	0,00
MALT_ECODH	*malT*	HTH-type transcriptional regulator MalT	5,66	103118,453	6,028	3		296051	182657	10,0	2,7	0,62	0,00	0,00
B1XF78_ECODH	*manA*	Mannose-6-phosphate isomerase	29,923	42849,949	5,29	8		2234063	2108508	6,5	2,9	0,94	0,17	0,22
B1XH84_ECODH	*manX*	Fused mannose-specific PTS enzymes: IIA component I	51,084	35047,551	5,742	10		5952514	3441347	4,1	2,9	0,58	0,00	0,00
B1XH86_ECODH	*manZ*	Mannose-specific enzyme IID component of PTS	21,329	31302,873	9,171	5		1835094	1052411	14,6	6,3	0,57	0,00	0,00
B1XD37_ECODH	*map*	Methionine aminopeptidase	29,545	29330,799	5,641	4		1744471	1814043	22,9	15,8	1,04	0,81	0,85
B1XG33_ECODH	*mdaB*	NADPH quinone reductase	12,953	21890,906	5,845	2		44320	66542	10,3	12,2	1,50	0,00	0,01
MDH_ECODH	*mdh*	Malate dehydrogenase	66,987	32337,299	5,607	13		27639542	17644127	6,5	6,9	0,64	0,00	0,00
OPGD_ECODH	*mdoD*	Glucans biosynthesis protein D	15,064	62757,945	5,893	5	metabolism	160453	143748	21,5	13,3	0,90	0,42	0,49
OPGG_ECODH	*mdoG*	Glucans biosynthesis protein G	30,724	57912,484	6,7	13		3613184	3800150	7,8	4,6	1,05	0,31	0,39
B1X9G3_ECODH	*mdoH*	Glucans biosynthesis glucosyltransferase H	10,39	96953,43	8,871	6		554545	166086	10,3	7,8	0,30	0,00	0,00
MDTA_ECODH	*mdtA*	Multidrug resistance protein MdtA	6,506	44464,289	8,903	2		23266	42388	36,5	5,0	1,82	0,03	0,06
B1X7W8_ECODH	*mdtE*	Multidrug resistance efflux transporter	42,857	41190,57	5,73	9		393338	1173527	10,2	3,7	2,98	0,00	0,00
B1X7W9_ECODH	*mdtF*	Multidrug transporter, RpoS-dependent	8,004	111517,172	5,326	5		70557	234013	27,9	13,2	3,32	0,00	0,00
MDTK_ECODH	*mdtK*	Multidrug resistance protein MdtK	6,565	49447,047	9,63	2		80689	67821	17,1	9,4	0,84	0,12	0,17
B1X8X6_ECODH	*menB*	Dihydroxynaphthoic acid synthetase	29,825	31633,08	5,994	7		1654212	1312029	5,6	4,1	0,79	0,00	0,00
MENC_ECODH	*menC*	o-succinylbenzoate synthase	10,625	35476,613	4,836	2		141462	110359	11,2	8,2	0,78	0,01	0,02
METE_ECODH	*metE*	5-methyltetrahydropteroyltriglutamate--homocysteine me	14,475	84673,539	5,609	8		105289	600302	39,2	9,1	5,70	0,00	0,00
SYM_ECODH	*metG*	Methionine--tRNA ligase	41,802	76254,734	5,565	21		7789794	6702436	5,5	2,3	0,86	0,00	0,01
B1XC18_ECODH	*metH*	Homocysteine-N5-methyltetrahydrofolate transmethylase	16,87	135997,047	4,97	14		529604	727173	7,5	4,7	1,37	0,00	0,00
METK_ECODH	*metK*	S-adenosylmethionine synthase	50,26	41951,625	5,096	13		5592313	5596898	16,1	6,1	1,00	0,99	0,99
B1XBA6_ECODH	*metL*	Fused aspartokinase II homoserine dehydrogenase II	7,901	88887,695	5,343	4		97858	100051	11,2	6,1	1,02	0,74	0,79
B1XD69_ECODH	*metN*	ATP-binding component of DL-methionine uptake transp	32,362	37788,371	5,947	6		336229	399357	1,7	13,3	1,19	0,04	0,07
B1XD67_ECODH	*metQ*	Lipoprotein	53,506	29431,65	5,127	8		1626926	2110634	18,0	6,2	1,30	0,03	0,06
B1XA24_ECODH	*mfd*	Transcription-repair-coupling factor	13,415	129982,727	5,787	13		2707625	2639304	3,3	6,9	0,97	0,53	0,60
B1X7N8_ECODH	*mglB*	Methyl-galactoside transporter subunit periplasmic-bindi	34,337	35712,574	5,68	8		6766580	1702765	14,2	5,6	0,25	0,00	0,00
MGSA_ECODH	*mgsA*	Methylglyoxal synthase	19,737	16918,576	6,12	2		589560	360275	18,4	15,7	0,61	0,01	0,01
B1XEM3_ECODH	*mgtA*	Magnesium transporter	15,924	99466,484	5,635	11		888880	1148743	8,1	5,7	1,29	0,00	0,01
MIAA_ECODH	*miaA*	tRNA dimethylallyltransferase	18,354	35065,152	5,682	4	metabolism	183851	131493	12,9	10,6	0,72	0,01	0,02
MIAB_ECODH	*miaB*	tRNA-2-methylthio-N(6)-dimethylallyladenosine synthase	36,92	53662,957	5,197	14		4113812	3717585	5,5	4,3	0,90	0,03	0,05
B1XA62_ECODH	*minD*	Site-determining protein	44,444	29614,033	5,246	9		5580154	5614458	4,1	5,1	1,01	0,86	0,89
MINE_ECODH	*minE*	Cell division topological specificity factor	54,545	10234,906	5,147	3		934685	866389	7,6	11,3	0,93	0,31	0,39
B1XGP2_ECODH	*mipA*	Scaffolding protein for murein synthesizing machinery	31,048	27830,986	5,501	5		3670085	2351192	12,5	5,3	0,64	0,00	0,00
B1XDM2_ECODH	*mltA*	Membrane-bound lytic murein transglycosylase A	15,89	40410,609	9,041	3		482508	527901	9,7	2,1	1,09	0,13	0,19
B1XCM8_ECODH	*mltB*	Membrane-bound lytic murein transglycosylase B	21,33	40255,746	9,068	4		317407	297617	6,9	9,4	0,94	0,31	0,39
MNMC_ECODH	*mnmC*	tRNA 5-methylaminomethyl-2-thiouridine biosynthesis bi	6,737	74434,164	5,43	2		46024	72272	13,5	6,3	1,57	0,00	0,00
MNME_ECODH	*mnmE*	tRNA modification GTPase MnmE	9,692	49230,871	4,892	3		121346	142003	10,3	9,7	1,17	0,07	0,11
MNMG_ECODH	*mnmG*	tRNA uridine 5-carboxymethylaminomethyl modification	19,714	69521,258	6,195	8		774735	464668	6,7	8,8	0,60	0,00	0,00
B1X7B2_ECODH	*moaB*	Molybdopterin biosynthesis protein B	29,412	18665,094	5,727	4		1551415	906380	12,8	6,0	0,58	0,00	0,00
MOAC_ECODH	*moaC*	Cyclic pyranopterin monophosphate synthase accessory	24,224	17467,236	6,593	3		453317	390513	11,6	2,7	0,86	0,04	0,07
B1X7B4_ECODH	*moaD*	Molybdopterin synthase, small subunit	37,037	8757,937	4,381	2		236372	184934	11,4	13,8	0,78	0,03	0,06
B1X7B5_ECODH	*moaE*	Molybdopterin synthase, large subunit	23,333	16981,055	5,256	2		479309	354980	9,1	10,8	0,74	0,01	0,01
B1X791_ECODH	*modE*	DNA-binding transcriptional dual regulator	27,099	28281,225	5,22	3		215666	177967	18,4	16,5	0,83	0,18	0,24
B1X7F7_ECODH	*moeA*	Molybdopterin biosynthesis protein	40,876	44067,273	5,026	8		1796226	2037831	4,7	4,5	1,13	0,01	0,02
B1XBD4_ECODH	*mog*	Predicted molybdochelatase	41,026	21222,354	4,971	6		740304	528562	10,6	8,9	0,71	0,00	0,01
B1XEL5_ECODH	*mpl*	UDP-N-acetylmuramate:L-alanyl-gamma-D-glutamyl-me	19,037	49874,117	5,531	6		785866	818415	8,7	8,0	1,04	0,51	0,58
B1XCF8_ECODH	*mppA*	Murein tripeptide (L-ala-gamma-D-glutamyl-meso-DAP)	5,959	59900,207	8,445	2		224235	123246	8,0	6,9	0,55	0,00	0,00
B1X743_ECODH	*mrcA*	Fused penicillin-binding protein 1a: murein transglycosyl	9,882	93636,172	6,155	6		313149	279789	7,4	9,7	0,89	0,11	0,17

B1XCC4_ECODH	*mrcB*	Bifunctional glycosyl transferase and transpeptidase	8,886	94292,555	9,1	5		313003	216245	10,7	2,4	0,69	0,00	0,00
B1XHM1_ECODH	*mreB*	Cell wall structural complex MreBCD, actin-like compone	40,634	36952,402	5,192	11		6796024	8359545	11,1	3,8	1,23	0,01	0,03
B1X7K3_ECODH	*mrp*	Antiporter inner membrane protein	18,97	39938,078	5,854	4		683796	571653	7,1	12,4	0,84	0,05	0,09
B1X854_ECODH	*msbA*	Fused lipid transporter subunits of ABC superfamily: me	15,979	64460,707	8,62	7		814459	721539	13,0	4,8	0,89	0,12	0,18
B1XEK7_ECODH	*mscS*	Mechanosensitive channel	22,028	30896,021	7,896	5		1138295	2555435	14,9	3,6	2,24	0,00	0,00
MSRB_ECODH	*msrB*	Peptide methionine sulfoxide reductase MsrB	37,226	15451,175	5,581	3		690558	914331	6,1	8,9	1,32	0,00	0,01
B1X9G5_ECODH	*msyB*	Uncharacterized protein	50	14259,198	3,587	3		156594	383457	27,2	19,4	2,45	0,00	0,00
B1X8L3_ECODH	*mtlA*	Fused mannitol-specific PTS enzymes: IIA components	11,774	67972,258	6,047	4		238941	434647	19,1	6,5	1,82	0,00	0,00
MTLD_ECODH	*mtlD*	Mannitol-1-phosphate 5-dehydrogenase	25,131	41139,016	5,357	6		496198	711108	13,9	11,9	1,43	0,01	0,02
MTNN_ECODH	*mtnN*	5'-methylthioadenosine/S-adenosylhomocysteine nucleo	15,086	24353,967	5,092	2		416729	876095	31,3	14,9	2,10	0,00	0,01
MUG_ECODH	*mug*	G/U mismatch-specific DNA glycosylase	35,119	18673,393	9,169	4		132001	282446	6,0	5,2	2,14	0,00	0,00
MUKB_ECODH	*mukB*	Chromosome partition protein MukB	15,007	170230,172	5,237	18		2379563	2000704	7,4	4,2	0,84	0,01	0,01
B1X8M7_ECODH	*mukE*	Chromosome partition protein MukE	32	25984,404	4,974	5		611704	530095	8,1	12,8	0,87	0,11	0,16
MUKF_ECODH	*mukF*	Chromosome partition protein MukF	9,773	50578,91	4,752	3		184186	164784	19,5	6,4	0,89	0,34	0,41
B1XBX9_ECODH	*murB*	UDP-N-acetylenolpyruvoylglucosamine reductase	11,404	37851,031	5,779	3		193427	202685	17,9	10,3	1,05	0,65	0,71
MURC_ECODH	*murC*	UDP-N-acetylmuramate--L-alanine ligase	12,22	53625,961	5,529	3		225879	235497	18,1	2,5	1,04	0,67	0,73
B1XC65_ECODH	*murD*	UDP-N-acetylmuramoylalanine--D-glutamate ligase	8,447	46973,621	5,153	3		153651	175991	7,6	8,9	1,15	0,06	0,09
B1XC62_ECODH	*murE*	UDP-N-acetylmuramoyl-L-alanyl-D-glutamate--2,6-diami	16,162	53343,598	5,425	6		541420	510577	13,9	11,9	0,94	0,54	0,61
B1XC63_ECODH	*murF*	UDP-N-acetylmuramoyl-tripeptide--D-alanyl-D-alanine lig	4,867	47447,148	5,253	2		122241	123609	14,1	8,1	1,01	0,90	0,92
MUTS_ECODH	*mutS*	DNA mismatch repair protein MutS	15,592	95246,898	5,394	9		645472	553720	7,3	2,8	0,86	0,01	0,02
B1XC76_ECODH	*mutT*	Nucleoside triphosphate pyrophosphohydrolase, markec	10,078	14927,073	5,012	1		29640	28561	19,4	10,2	0,96	0,73	0,79
NADE_ECODH	*nadE*	NH(3)-dependent NAD(+) synthetase	44,727	30636,834	5,413	11		2225754	3146535	5,8	5,0	1,41	0,00	0,00
B1XFK0_ECODH	*nadR*	NadR transcriptional repressor / ribosylnicotinamide kina	15,61	47345,832	5,423	4		244108	237632	17,0	6,6	0,97	0,78	0,83
B1X6L0_ECODH	*nagA*	N-acetylglucosamine-6-phosphate deacetylase	8,115	40949,098	5,645	2		308686	208068	13,4	6,1	0,67	0,00	0,01
NAGB_ECODH	*nagB*	Glucosamine-6-phosphate deaminase	8,647	29774,203	6,411	2		577102	240281	11,8	10,0	0,42	0,00	0,00
B1X6K8_ECODH	*nagD*	UMP phosphatase	35,6	27189,133	5,067	5		801032	901735	5,6	6,7	1,13	0,03	0,06
B1X6L2_ECODH	*nagE*	Fused N-acetyl glucosamine specific PTS enzyme: IIC, I	17,13	68346,891	5,784	7		2522252	1006677	11,7	4,4	0,40	0,00	0,00
NAGZ_ECODH	*nagZ*	Beta-hexosaminidase	8,504	37594,73	5,863	2		65382	99779	32,6	10,3	1,53	0,05	0,08
NANA_ECODH	*nanA*	N-acetylneuraminate lyase	44,108	32593,469	5,61	9		13245153	3587546	9,6	6,8	0,27	0,00	0,00
NANE_ECODH	*nanE*	Putative N-acetylmannosamine-6-phosphate 2-epimeras	22,707	24073,66	4,892	3		1000090	392665	13,8	9,1	0,39	0,00	0,00
NANK_ECODH	*nanK*	N-acetylmannosamine kinase	32,302	29643,865	5,626	7		780866	244975	5,8	13,0	0,31	0,00	0,00
NAPA_ECODH	*napA*	Periplasmic nitrate reductase	17,874	93042,023	8,234	9		1218982	166128	5,8	10,3	0,14	0,00	0,00
B1XAR8_ECODH	*narG*	Nitrate reductase 1, alpha subunit	38,893	140489,391	6,052	33		17465338	737342	9,3	4,1	0,04	0,00	0,00
B1XAR9_ECODH	*narH*	Nitrate reductase 1, beta (Fe-S) subunit	38,672	58066,41	6,362	14		5043912	164006	8,3	5,9	0,03	0,00	0,00
B1XAS1_ECODH	*narI*	Nitrate reductase 1, gamma (Cytochrome b(NR)) subuni	10,222	25497,365	9,774	2		177890	2720	11,4	38,8	0,02	0,00	0,00
B1XAS0_ECODH	*narJ*	Molybdenum-cofactor-assembly chaperone subunit (Del	36,441	26448,865	4,757	5		598401	36211	10,4	35,3	0,06	0,00	0,00
B1XAR7_ECODH	*narK*	Nitrate/nitrite transporter	7,127	49693,434	9,679	2		1878792	40343	12,6	15,8	0,02	0,00	0,00
B1XAR5_ECODH	*narL*	DNA-binding response regulator in two-component regu	12,037	23926,791	5,727	3		273107	359080	11,2	7,8	1,31	0,01	0,02
B1X889_ECODH	*narP*	DNA-binding response regulator in two-component regu	18,14	23574,975	4,816	3		309355	276926	20,6	7,1	0,90	0,38	0,45
B1XA19_ECODH	*ndh*	Respiratory NADH dehydrogenase 2/cupric reductase	42,166	47358,672	8,961	12		5203853	4116482	8,5	4,7	0,79	0,00	0,01
NDK_ECODH	*ndk*	Nucleoside diphosphate kinase	64,336	15463,444	5,54	7		5048926	1972390	12,0	3,6	0,39	0,00	0,00
B1XFW1_ECODH	*nemA*	N-ethylmaleimide reductase, FMN-linked	15,89	39516,414	5,803	4		151593	187710	24,0	10,1	1,24	0,14	0,19
B1X5X3_ECODH	*nfnB*	Dihydropteridine reductase, NAD(P)H-dependent, oxyge	37,327	23905,191	5,802	6		2242933	2921827	7,8	0,7	1,30	0,00	0,00
B1X7Z2_ECODH	*nfsA*	Nitroreductase A, NADPH-dependent, FMN-dependent	31,667	26800,686	6,444	6		1052466	1510382	23,6	8,2	1,44	0,03	0,06
NFUA_ECODH	*nfuA*	Fe/S biogenesis protein NfuA	26,702	20997,705	4,524	4		3199824	4078251	15,4	5,0	1,27	0,03	0,06
B1X7T4_ECODH	*nikA*	Nickel transporter subunit periplasmic-binding componei	13,931	58719,02	5,745	5		397946	45323	13,9	8,0	0,11	0,00	0,00
B1X713_ECODH	*nirB*	Nitrite reductase, large subunit, NAD(P)H-binding	12,869	93121,32	5,79	7		1524584	136469	4,2	8,5	0,09	0,00	0,00
B1X714_ECODH	*nirD*	Nitrite reductase, NAD(P)H-binding, small subunit	13,889	12284,003	5,074	1		317652	8909	7,0	38,4	0,03	0,00	0,00
B1XAE9_ECODH	*nlpB*	Outer membrane protein assembly factor BamC	56,395	36842,43	5,337	12		2707307	3527851	12,1	7,2	1,30	0,01	0,02
B1XCR8_ECODH	*nlpD*	Predicted outer membrane lipoprotein	17,15	40149	9,518	3		409387	601923	13,5	13,7	1,47	0,01	0,02
B1XD62_ECODH	*nlpE*	Lipoprotein involved with copper homeostasis and adhes	70,763	25844,287	5	11		3091263	5776488	7,1	26,2	1,87	0,00	0,01
B1X8C8_ECODH	*nrdA*	Ribonucleoside-diphosphate reductase	31,012	85775,281	5,791	18		6917555	6466229	3,0	2,3	0,93	0,01	0,02
B1X8C9_ECODH	*nrdB*	Ribonucleoside diphosphate reductase 1, beta subunit, f	21,277	43517,273	4,691	7		2120658	2025340	11,4	3,7	0,96	0,48	0,56
B1XEL9_ECODH	*nrdD*	Anaerobic ribonucleoside-triphosphate reductase	12,079	80022,562	6,376	6		693274	88047	10,5	22,2	0,13	0,00	0,00
NRDR_ECODH	*nrdR*	Transcriptional repressor NrdR	16,107	17228,895	7,664	2		224062	233505	25,5	16,1	1,04	0,79	0,83
NUDC_ECODH	*nudC*	NADH pyrophosphatase	23,346	29688,939	5,518	4		181713	191985	4,0	7,1	1,06	0,23	0,29
B1X744_ECODH	*nudE*	ADP-ribose diphosphatase	13,978	21153,166	4,847	1	metabolism	42646	39909	20,6	31,9	0,94	0,76	0,81
NUDK_ECODH	*nudK*	GDP-mannose pyrophosphatase NudK	14,136	21748,664	4,879	1		8677	28376	32,7	39,0	3,27	0,01	0,02
NUOA_ECODH	*nuoA*	NADH-quinone oxidoreductase subunit A	17,687	16457,328	9,874	2		844903	851510	14,5	12,0	1,01	0,94	0,95
NUOB_ECODH	*nuoB*	NADH-quinone oxidoreductase subunit B	30,455	25055,816	5,587	7		2379764	1797372	8,4	4,1	0,76	0,00	0,00
NUOCD_ECODH	*nuoC*	NADH-quinone oxidoreductase subunit C/D	44,833	68694,648	5,98	21		9056337	5861018	5,8	3,1	0,65	0,00	0,00
B1X8Z7_ECODH	*nuoE*	NADH:ubiquinone oxidoreductase, chain E	19,277	18590,051	5,402	2		768682	554421	10,0	1,0	0,72	0,00	0,00
B1X8Z6_ECODH	*nuoF*	NADH:ubiquinone oxidoreductase, chain F	32,36	49292,453	6,441	10		2813394	2166352	14,5	7,1	0,77	0,02	0,04
B1X8Z5_ECODH	*nuoG*	NADH:ubiquinone oxidoreductase, chain G	35,714	100543,555	5,845	22		8981863	5238663	5,8	2,4	0,58	0,00	0,00
NUOH_ECODH	*nuoH*	NADH-quinone oxidoreductase subunit H	9,538	36219,152	6,898	3		825798	506595	17,4	7,0	0,61	0,00	0,01
B1X8Z3_ECODH	*nuoI*	NADH-quinone oxidoreductase subunit I	15	20537,75	5,396	3		478394	304863	11,4	16,3	0,64	0,00	0,01
NUOK_ECODH	*nuoK*	NADH-quinone oxidoreductase subunit K	12	10845,019	8,381	1		283364	140598	19,3	17,8	0,50	0,00	0,01
B1X8Z0_ECODH	*nuoL*	NADH:ubiquinone oxidoreductase, membrane subunit L	4,568	66438,078	8,283	2		654536	394898	22,9	12,0	0,60	0,01	0,02
B1X8Y9_ECODH	*nuoM*	NADH:ubiquinone oxidoreductase, membrane subunit M	3,143	56524,543	8,62	2		306885	284137	20,3	13,1	0,93	0,54	0,60
B1X9R4_ECODH	*nupC*	Nucleoside (Except guanosine) transporter	10,5	43475,754	8,5	3		332252	607219	32,3	4,7	1,83	0,01	0,02
B1XGY1_ECODH	*nusA*	Transcription termination/antitermination L factor	44,242	54870,922	4,527	15		7332866	6559217	6,9	6,7	0,89	0,06	0,09
NUSB_ECODH	*nusB*	N utilization substance protein B homolog	37,41	15689,056	6,601	4		1757353	1163365	3,7	17,6	0,66	0,00	0,01
B1XBY4_ECODH	*nusG*	Transcription termination/antitermination protein NusG	47,514	20531,514	6,339	6		3295984	2560122	4,6	9,2	0,78	0,00	0,01
OBG_ECODH	*obg*	GTPase Obg	25,641	43285,863	4,743	7		1721199	1353028	12,0	7,0	0,79	0,02	0,03

B1X8R1_ECODH	*ompA*	Outer membrane protein A (3aII*Gd)	61,272	37200,762	5,989	14		117862205	144851719	8,9	2,1	1,23	0,00	0,01
B1X8B1_ECODH	*ompC*	Outer membrane porin protein C	80,109	40368,117	4,581	19		41404866	47752496	16,3	20,4	1,15	0,31	0,39
B1X8N3_ECODH	*ompF*	Outer membrane porin 1a (IabF)	30,663	39333,309	4,76	8		3711984	1954363	14,1	8,4	0,53	0,00	0,00
B1X752_ECODH	*ompR*	DNA-binding response regulator in two-component regu	46,862	27353,617	6,038	7		2087056	2175112	10,6	5,1	1,04	0,51	0,58
B1XGF9_ECODH	*ompT*	DLP12 prophage outer membrane protease VII (Outer m	66,562	35562,309	5,755	15		9553423	12615517	8,4	1,9	1,32	0,00	0,00
B1XBK5_ECODH	*ompW*	Outer membrane protein W	10,849	22927,83	6,029	2		1090365	72544	13,9	11,7	0,07	0,00	0,00
B1X7E4_ECODH	*ompX*	Outer membrane protein	46,784	18602,617	6,561	6		22553305	23770159	6,9	4,6	1,05	0,26	0,33
B1XAT3_ECODH	*oppA*	Oligopeptide transporter periplasmic subunit	50,092	60948,004	6,053	17		9501322	21525584	8,2	3,8	2,27	0,00	0,00
B1XAT4_ECODH	*oppB*	Oligopeptide transporter subunit membrane component	8,17	33443,199	9,783	2		95408	2075	17,8	69,9	0,02	0,00	0,00
B1XAT6_ECODH	*oppD*	Oligopeptide transporter subunit ATP-binding componer	19,288	37188,43	5,777	4		286261	16464	5,4	40,4	0,06	0,00	0,00
B1XAT7_ECODH	*oppF*	ATP-binding subunit of oligopeptide ABC transporter	37,725	37181,547	7,647	11		1050577	145569	14,3	10,2	0,14	0,00	0,00
B1XDR6_ECODH	*orn*	Oligoribonuclease	16,575	20815,623	4,963	3		331221	285691	3,8	8,2	0,86	0,02	0,04
B1XE73_ECODH	*osmC*	Osmotically inducible, stress-inducible membrane protei	40,559	15088,142	5,57	4		2086352	2415672	4,3	6,2	1,16	0,01	0,02
B1XFI5_ECODH	*osmY*	Periplasmic protein	17,91	21073,707	6,315	3		171276	4306572	19,0	8,9	25,14	0,00	0,00
B1XBX2_ECODH	*oxyR*	DNA-binding transcriptional dual regulator	16,393	34275,93	5,959	3		373488	513489	18,3	5,1	1,37	0,01	0,03
B1X6S2_ECODH	*pal*	Peptidoglycan-associated outer membrane lipoprotein	32,37	18824,186	6,292	4		1890773	1821939	25,8	25,9	0,96	0,85	0,89
PANB_ECODH	*panB*	3-methyl-2-oxobutanoate hydroxymethyltransferase	46,591	28237,439	5,148	7		1687580	1724708	1,4	7,0	1,02	0,56	0,63
PANC_ECODH	*panC*	Pantothenate synthetase	9,187	31597,672	5,915	2		445392	520084	21,8	3,0	1,17	0,22	0,28
B1XFH0_ECODH	*parC*	DNA topoisomerase 4 subunit A	19,149	83831,266	6,243	8		882540	812413	3,5	6,1	0,92	0,06	0,09
B1XG35_ECODH	*parE*	DNA topoisomerase 4 subunit B	17,302	70243,914	5,436	8		787285	852605	23,5	2,0	1,08	0,49	0,57
PCKA_ECODH	*pckA*	Phosphoenolpyruvate carboxykinase [ATP]	40,37	59643,48	5,459	15		17360214	7023485	5,2	2,9	0,40	0,00	0,00
PIMT_ECODH	*pcm*	Protein-L-isoaspartate O-methyltransferase	33,173	23257,775	6,528	5		237444	282003	5,4	4,6	1,19	0,00	0,01
B1XCB8_ECODH	*pcnB*	Poly(A) polymerase I	22,151	53870,91	9,669	8		807265	766337	7,6	5,7	0,95	0,32	0,39
B1XC88_ECODH	*pdhR*	DNA-binding transcriptional dual regulator	22,441	29425,471	6,036	4		243095	192601	8,4	12,6	0,79	0,02	0,04
PDXA_ECODH	*pdxA*	4-hydroxythreonine-4-phosphate dehydrogenase	12,766	35113,777	5,869	2		106515	103812	17,8	3,4	0,97	0,80	0,84
PDXB_ECODH	*pdxB*	Erythronate-4-phosphate dehydrogenase	36,508	41367,648	6,227	10		1571922	1711303	7,8	1,4	1,09	0,07	0,11
PDXH_ECODH	*pdxH*	Pyridoxine/pyridoxamine 5'-phosphate oxidase	29,817	25545,152	9,182	5		554655	508490	12,0	4,5	0,92	0,23	0,30
B1XB38_ECODH	*pdxJ*	Pyridoxine 5'-phosphate synthase	15,226	26384,287	5,61	3		352999	590280	14,3	6,5	1,67	0,00	0,00
PDXK_ECODH	*pdxK*	Pyridoxine kinase	20,141	30847,4	5,141	4		262623	242234	25,5	10,1	0,92	0,63	0,69
B1XFU8_ECODH	*pdxY*	Pyridoxamine kinase	19,861	31322,197	6,041	4		324944	370169	18,8	5,7	1,14	0,24	0,31
AMPA_ECODH	*pepA*	Probable cytosol aminopeptidase	28,032	54879,809	6,815	11		1643993	2154164	13,9	3,1	1,31	0,01	0,02
PEPB_ECODH	*pepB*	Peptidase B	53,396	46180,168	5,602	17		4347923	4797718	1,6	1,4	1,10	0,00	0,00
B1XDY0_ECODH	*pepD*	Aminoacyl-histidine dipeptidase (Peptidase D)	26,392	52915,406	5,204	10		5169246	5404855	7,8	4,8	1,05	0,36	0,44
B1X8N6_ECODH	*pepN*	Aminopeptidase N	18,736	98918,938	5,142	14		5335769	3760663	13,8	3,0	0,70	0,00	0,01
B1XEJ4_ECODH	*pepP*	Proline aminopeptidase P II	25,17	49815,434	5,245	8		967898	1109902	9,3	9,3	1,15	0,08	0,13
PEPQ_ECODH	*pepQ*	Xaa-Pro dipeptidase	32,731	50176,176	5,602	11		3527535	6154307	4,0	1,1	1,74	0,00	0,00
PEPT_ECODH	*pepT*	Peptidase T	19,608	44923,426	5,36	7		1263556	583784	4,3	7,0	0,46	0,00	0,00
K6PF_ECODH	*pfkA*	6-phosphofructokinase	21,25	34842,039	5,47	6		15577497	12598060	11,4	8,8	0,81	0,02	0,05
B1XGI4_ECODH	*pfkB*	Phosphofructokinase	32,362	32455,992	5,252	7		1113787	1665093	7,8	2,8	1,49	0,00	0,00
B1X842_ECODH	*pflA*	Pyruvate formate lyase activating enzyme 1	11,382	28204,291	6,002	3		153239	172672	37,3	3,7	1,13	0,55	0,62
B1X843_ECODH	*pflB*	Pyruvate formate lyase I	59,605	85357,203	5,69	29		70304609	22488368	9,0	2,4	0,32	0,00	0,00
G6PI_ECODH	*pgi*	Glucose-6-phosphate isomerase	37,705	61529,766	5,853	16		10557213	10492433	3,5	4,0	0,99	0,82	0,86
B1XEK9_ECODH	*pgk*	Phosphoglycerate kinase	59,948	41118,219	5,079	15		46055121	80266613	5,0	4,9	1,74	0,00	0,00
6PGL_ECODH	*pgl*	6-phosphogluconolactonase	34,743	36307,637	5,06	7		3134457	4986564	15,4	5,3	1,59	0,00	0,01
B1X6M1_ECODH	*pgm*	Phosphoglucomutase	40,293	58360,949	5,429	15		3469062	3775460	5,7	5,5	1,09	0,08	0,12
SYFA_ECODH	*pheS*	Phenylalanine--tRNA ligase alpha subunit	36,697	36831,805	5,792	10		4256088	3443680	4,0	3,9	0,81	0,00	0,00
B1XG20_ECODH	*pheT*	Phenylalanine--tRNA ligase beta subunit	44,403	87378,109	5,169	26		12908867	10095980	1,2	4,2	0,78	0,00	0,00
B1XCZ1_ECODH	*phnA*	Conserved protein	25,225	12345,049	4,971	2		224617	439817	27,7	9,9	1,96	0,00	0,01
B1XCZ0_ECODH	*phnB*	Conserved protein	10,884	16171,316	5,037	1		37455	40400	16,4	12,0	1,08	0,48	0,55
B1XA40_ECODH	*phoP*	DNA-binding response regulator in two-component regu	43,049	25535,217	5,098	7		1210074	980643	8,5	5,4	0,81	0,01	0,01
B1X7U8_ECODH	*pitA*	Phosphate transporter, low-affinity	8,016	53389,027	9,533	3		403122	287692	27,1	10,2	0,71	0,06	0,09
B1XAI6_ECODH	*pldA*	Outer membrane phospholipase A	39,1	33163,133	5,154	9		618931	1011379	14,8	1,2	1,63	0,00	0,00
PLSB_ECODH	*plsB*	Glycerol-3-phosphate acyltransferase	36,307	91381,445	8,512	20		2187624	2369098	8,0	1,4	1,08	0,10	0,14
B1XFG9_ECODH	*plsC*	1-acyl-sn-glycerol-3-phosphate acyltransferase	7,755	27453,158	9,655	2		140152	108795	15,8	21,5	0,78	0,11	0,16
B1XEL7_ECODH	*pmbA*	Predicted peptidase required for the maturation and sec	33,556	48369,637	5,401	10		2090594	2194933	7,7	3,0	1,05	0,29	0,36
B1X8X3_ECODH	*pmrD*	Polymyxin resistance protein B	15,909	9870,51	8,641	1		85605	60771	13,1	13,8	0,71	0,01	0,02
PNCB_ECODH	*pncB*	Nicotinate phosphoribosyltransferase	17,5	45897,285	6,208	6		273929	299218	13,2	8,1	1,09	0,31	0,38
PNP_ECODH	*pnp*	Polyribonucleotide nucleotidyltransferase	41,069	77100,977	5,114	20		13143388	11604095	8,3	3,7	0,88	0,03	0,06
B1XF68_ECODH	*pntA*	NAD(P) transhydrogenase subunit alpha	11,961	54623,441	5,645	5		1110482	1139812	14,9	6,5	1,03	0,75	0,80
B1XF67_ECODH	*pntB*	NAD(P) transhydrogenase subunit beta	20,13	48723,027	5,722	6		1114896	1077597	6,3	7,2	0,97	0,50	0,57
B1XAM0_ECODH	*polA*	DNA polymerase I	27,909	103118,125	5,395	18		2287821	2155755	5,4	4,0	0,94	0,13	0,18
B1XA36_ECODH	*potA*	Polyamine transporter subunit ATP-binding component c	28,307	43028,215	5,19	7		1379368	1087861	4,3	8,6	0,79	0,00	0,01
B1XA35_ECODH	*potB*	Polyamine transporter subunit membrane component of	7,273	31061,982	9,437	2		100264	115650	27,4	16,7	1,15	0,37	0,45
B1XA33_ECODH	*potD*	Putrescine-binding periplasmic protein	22,414	38867,082	5,242	6		707094	1021516	24,0	7,0	1,44	0,03	0,05
B1X812_ECODH	*poxB*	Pyruvate dehydrogenase (Pyruvate oxidase), thiamin-de	26,049	62011,387	5,861	10		839473	1042215	7,3	3,5	1,24	0,00	0,00
B1XDX5_ECODH	*ppa*	Inorganic pyrophosphatase	16,477	19703,557	5,027	3		4414666	6231174	33,1	11,1	1,41	0,08	0,12
CAPP_ECODH	*ppc*	Phosphoenolpyruvate carboxylase	36,693	99062,609	5,517	25		3627899	6499350	7,5	2,7	1,79	0,00	0,00
B1X711_ECODH	*ppiA*	Peptidyl-prolyl cis-trans isomerase	27,368	20431,322	8,804	3	degradation/folding	336405	355443	13,7	6,0	1,06	0,50	0,57
B1XGC3_ECODH	*ppiB*	Peptidyl-prolyl cis-trans isomerase	21,341	18153,471	5,514	3		2971103	3917095	9,9	10,7	1,32	0,01	0,02
B1X9Z1_ECODH	*ppiC*	Peptidyl-prolyl cis-trans isomerase C (Rotamase C)	21,505	10232,021	9,23	2		157522	223854	12,0	11,7	1,42	0,01	0,01
B1XFM9_ECODH	*ppiD*	Peptidyl-prolyl cis-trans isomerase (Rotamase D)	34,831	68149,859	4,939	14		4421334	4348803	11,8	5,6	0,98	0,80	0,84
B1XAX6_ECODH	*ppk*	Polyphosphate kinase	20,203	80431,508	8,96	10		439193	466300	9,8	5,0	1,06	0,31	0,38
B1XG09_ECODH	*pps*	Phosphoenolpyruvate synthase	15,909	87435,078	4,934	11		1626461	490342	6,3	4,0	0,30	0,00	0,00
PSRP_ECODH	*ppsR*	Phosphoenolpyruvate synthase regulatory protein	12,635	31210,867	5,987	3		211555	167913	12,3	11,4	0,79	0,04	0,07

B1XAX7_ECODH	*ppx*	Exopolyphosphatase	10,916	58135,969	6,646	4		291790	282046	10,4	7,7	0,97	0,62	0,69
B1XH98_ECODH	*prc*	Carboxy-terminal protease for penicillin-binding protein 3	17,742	76663,094	6,19	9		1139784	1087705	7,5	6,5	0,95	0,38	0,45
RF1_ECODH	*prfA*	Peptide chain release factor 1	19,444	40517,301	5,146	5		355451	404745	16,1	5,4	1,14	0,16	0,22
RF2_ECODH	*prfB*	Peptide chain release factor 2	28,493	41250,727	4,643	7		4759150	3576526	9,1	3,1	0,75	0,00	0,00
RF3_ECODH	*prfC*	Peptide chain release factor 3	18,147	59574,082	5,655	7		1715892	1213934	13,9	5,2	0,71	0,00	0,01
B1X7V3_ECODH	*prlC*	Oligopeptidase A	39,265	77167,234	5,151	20		5002220	5825789	5,5	1,7	1,16	0,00	0,01
B1X9K3_ECODH	*prmB*	50S ribosomal protein L3 glutamine methyltransferase	15,161	35001,73	4,623	4		523053	856497	11,0	4,0	1,64	0,00	0,00
PROA_ECODH	*proA*	Gamma-glutamyl phosphate reductase	32,374	44630,055	5,418	10		1247561	1206368	8,7	6,1	0,97	0,55	0,61
PROB_ECODH	*proB*	Glutamate 5-kinase	24,251	39056,523	6,073	6		850950	729492	4,4	3,4	0,86	0,00	0,00
B1XEX5_ECODH	*proC*	Pyrroline-5-carboxylate reductase	21,933	28144,877	5,638	4		550892	774556	14,1	11,4	1,41	0,01	0,02
B1XCZ4_ECODH	*proP*	Proline/glycine betaine transporter	5,4	54845,816	6,763	2		92353	206674	25,5	8,7	2,24	0,00	0,00
PROQ_ECODH	*proQ*	ProP effector	47,414	25892,518	9,664	9		3290284	2949121	9,9	6,4	0,90	0,11	0,16
SYP_ECODH	*proS*	Proline--tRNA ligase	53,147	63692,602	5,122	21		15180981	12148786	4,2	2,6	0,80	0,00	0,00
B1XCL5_ECODH	*proX*	Glycine betaine transporter subunit periplasmic-binding (	26,97	36022,547	5,903	5		1672522	3948060	16,8	13,9	2,36	0,00	0,00
B1XAP8_ECODH	*prsA*	Ribose-phosphate pyrophosphokinase	42,857	34218,27	5,23	9		8700154	8581462	9,2	3,1	0,99	0,79	0,83
PSD_ECODH	*psd*	Phosphatidylserine decarboxylase proenzyme	32,609	35934,414	5,51	6	metabolism	1465011	1288674	13,8	4,6	0,88	0,14	0,20
B1XEX3_ECODH	*psiF*	Conserved protein	13,208	11686,53	9,784	1		1145	87520	120,7	35,2	76,42	0,00	0,00
B1XCD3_ECODH	*pspA*	Regulatory protein for phage-shock-protein operon	19,82	25492,939	5,385	3		189134	192907	24,2	6,3	1,02	0,88	0,90
B1XCD7_ECODH	*pspE*	Thiosulfate:cyanide sulfurtransferase (Rhodanese)	18,269	11475,313	7,864	1		24266	6495	31,4	33,2	0,27	0,00	0,00
B1XBR2_ECODH	*pssA*	Phosphatidylserine synthase (CDP-diacylglycerol-serine	34,368	52801,793	9,06	11		1916500	1417331	6,3	5,2	0,74	0,00	0,00
B1X9V4_ECODH	*pstB*	Phosphate transporter subunit ATP-binding component (	15,564	29027,289	6,125	2		196127	229966	54,6	11,8	1,17	0,63	0,70
B1X9V7_ECODH	*pstS*	Phosphate-binding protein PstS	6,069	37023,922	8,386	2		105254	152154	23,4	10,3	1,45	0,03	0,06
B1X908_ECODH	*pta*	Phosphate acetyltransferase	41,457	77172,086	5,284	20		15409859	17906418	3,7	2,3	1,16	0,00	0,00
B1XDM7_ECODH	*ptrA*	Protease III	9,148	107708,477	5,782	7		205723	312790	1,1	3,7	1,52	0,00	0,00
B1XA11_ECODH	*ptsG*	Fused glucose-specific PTS enzymes: IIBcomponent IIC	20,545	50676,551	8,765	7		2450790	1755309	5,8	4,3	0,72	0,00	0,00
B1XA86_ECODH	*ptsH*	Phosphohistidinoprotein-hexose phosphotransferase co	15,294	9119,371	5,646	1		221870	328895	41,3	11,4	1,48	0,14	0,19
B1XA87_ECODH	*ptsI*	Phosphoenolpyruvate-protein phosphotransferase	46,957	63561,836	4,776	17		17930060	17360371	5,6	3,9	0,97	0,38	0,45
B1XHH9_ECODH	*ptsN*	Sugar-specific enzyme IIA component of PTS	26,38	17959,568	5,568	3		395882	320426	4,8	12,1	0,81	0,02	0,03
PURA_ECODH	*purA*	Adenylosuccinate synthetase	46,528	47344,949	5,316	14		17032532	10951158	8,9	5,2	0,64	0,00	0,00
B1XA41_ECODH	*purB*	Adenylosuccinate lyase	32,237	51542,805	5,675	11		3086773	2743835	8,2	5,6	0,89	0,05	0,09
PUR7_ECODH	*purC*	Phosphoribosylaminoimidazole-succinocarboxamide syr	46,835	26994,998	5,072	8		2024168	2578485	10,5	1,6	1,27	0,00	0,01
B1XC08_ECODH	*purD*	Phosphoribosylamine--glycine ligase	18,182	45940,359	4,957	6		427584	543143	7,3	3,2	1,27	0,00	0,00
B1XGC1_ECODH	*purE*	N5-carboxyaminoimidazole ribonucleotide mutase	23,669	17780,322	6,038	2		73630	108362	26,3	16,1	1,47	0,04	0,07
B1X923_ECODH	*purF*	Amidophosphoribosyltransferase	14,653	56488,055	5,325	5		316815	371254	15,0	12,6	1,17	0,16	0,22
PUR9_ECODH	*purH*	Bifunctional purine biosynthesis protein PurH	34,405	57329,207	5,526	13		1226762	1376077	0,9	7,2	1,12	0,02	0,04
B1XB32_ECODH	*purL*	Phosphoribosylformylglycinamidine synthase	20,849	141402,703	5,229	15		1230600	1268403	2,5	7,2	1,03	0,45	0,53
B1XAS4_ECODH	*purU*	Formyltetrahydrofolate hydrolase	23,929	31934,717	6,496	4		1910106	1008375	90,4	28,7	0,53	0,26	0,33
B1X9D5_ECODH	*putA*	Multifunctional proline dehydrogenase, delta-1-pyrroline-	24,848	143815,156	5,691	21		2927172	1888837	9,1	3,9	0,65	0,00	0,00
B1XHC2_ECODH	*pykA*	Pyruvate kinase	38,333	51357,145	6,239	13		5792844	3201863	4,1	2,5	0,55	0,00	0,00
B1XFY4_ECODH	*pykF*	Pyruvate kinase	50,851	50729,422	5,77	18		18087688	23538126	7,7	4,4	1,30	0,00	0,00
PYRB_ECODH	*pyrB*	Aspartate carbamoyltransferase	35,37	34427,359	6,116	7	metabolism	582300	1002757	14,3	18,4	1,72	0,00	0,01
PYRC_ECODH	*pyrC*	Dihydroorotase	41,379	38827,383	5,771	8		1954888	2577469	12,1	6,3	1,32	0,01	0,02
PYRD_ECODH	*pyrD*	Dihydroorotate dehydrogenase (quinone)	19,345	36774,52	7,661	5		419778	228677	11,3	7,2	0,54	0,00	0,00
PYRE_ECODH	*pyrE*	Orotate phosphoribosyltransferase	13,615	23566,998	5,333	3		55255	381716	99,5	8,2	6,91	0,00	0,01
PYRF_ECODH	*pyrF*	Orotidine 5'-phosphate decarboxylase	37,551	26350,244	5,813	6		1108103	1116591	7,6	4,5	1,01	0,87	0,89
PYRG_ECODH	*pyrG*	CTP synthase	26,422	60374,148	5,633	14		7928710	7182532	4,3	3,5	0,91	0,01	0,02
B1XD40_ECODH	*pyrH*	Uridylate kinase	19,087	25970,258	6,851	2		65029	135494	28,5	13,7	2,08	0,01	0,01
PYRI_ECODH	*pyrI*	Aspartate carbamoyltransferase regulatory chain	35,294	17120,631	6,895	4	metabolism	359136	634184	12,1	9,1	1,77	0,00	0,00
B1XCT9_ECODH	*qor*	Quinone oxidoreductase, NADPH-dependent	20,183	35172,188	8,351	4		233401	293030	9,0	11,2	1,26	0,02	0,04
QUEA_ECODH	*queA*	S-adenosylmethionine:tRNA ribosyltransferase-isomeras	15,73	39430,844	5,089	3		574489	375052	16,3	23,9	0,65	0,03	0,05
QUEC_ECODH	*queC*	7-cyano-7-deazaguanine synthase	15,152	25514,172	5,682	3		358886	180551	59,8	9,8	0,50	0,05	0,08
QUEF_ECODH	*queF*	NADPH-dependent 7-cyano-7-deazaguanine reductase	17,021	32587,508	5,726	5		234366	328853	10,7	3,1	1,40	0,00	0,00
RBFA_ECODH	*rbfA*	Ribosome-binding factor A	37,594	15154,482	5,932	4		1077891	812335	11,7	12,0	0,75	0,01	0,03
B1X9X6_ECODH	*rbsA*	Fused D-ribose transporter subunits of ABC superfamily	15,369	55041,609	5,837	6		178225	690829	5,1	5,7	3,88	0,00	0,00
B1X9X8_ECODH	*rbsB*	D-ribose transporter subunit periplasmic-binding compo	61,824	30950,48	6,849	11		5483777	45482589	15,4	6,4	8,29	0,00	0,00
B1X9X7_ECODH	*rbsC*	D-ribose transporter subunit membrane component of A	13,707	33451,824	9,811	2		329142	792053	11,6	4,5	2,41	0,00	0,00
RBSD_ECODH	*rbsD*	D-ribose pyranase	23,022	15292,5	5,929	2		306806	1088359	7,9	6,8	3,55	0,00	0,00
B1X9X9_ECODH	*rbsK*	Ribokinase	33,657	32290,518	4,987	6		544738	1587593	10,2	11,8	2,91	0,00	0,00
B1X8B3_ECODH	*rcsB*	DNA-binding response regulator in two-component regu	16,667	23670,617	6,85	2		587711	622105	29,4	13,4	1,06	0,76	0,81
B1X8B2_ECODH	*rcsD*	Phosphotransfer intermediate protein in two-component	6,966	100372,172	5,056	5		182244	220456	12,4	4,2	1,21	0,03	0,05
B1XD66_ECODH	*rcsF*	Predicted outer membrane protein, signal	54,478	14163,468	9,352	4		752063	1159992	26,2	5,8	1,54	0,01	0,03
RDGC_ECODH	*rdgC*	Recombination-associated protein RdgC	34,983	33993,324	5,273	8		760525	894454	5,8	7,0	1,18	0,01	0,03
B1XEH9_ECODH	*recJ*	SsDNA exonuclease, 5'--> 3'-specific	5,546	63388,832	5,388	2		146400	121041	19,5	7,8	0,83	0,12	0,17
RECR_ECODH	*recR*	Recombination protein RecR	24,876	21963	5,133	4		155811	134057	3,1	18,5	0,86	0,20	0,26
B1X9Z2_ECODH	*rep*	DNA helicase and single-stranded DNA-dependent ATP	5,349	77024,195	6,769	2		66499	65755	17,4	15,9	0,99	0,93	0,94
B1X954_ECODH	*rfaF*	ADP-heptose:LPS heptosyltransferase II	14,08	39042,133	7,148	3		180728	179986	9,7	8,8	1,00	0,95	0,96
B1X6X5_ECODH	*rfbA*	Glucose-1-phosphate thymidylyltransferase	38,225	32693,557	5,386	9		2755186	1927240	14,7	3,1	0,70	0,00	0,01
B1X6X7_ECODH	*rfbB*	dTDP-glucose 4,6-dehydratase	29,64	40558,332	5,475	8		2198932	1489046	10,5	2,6	0,68	0,00	0,00
B1X6X4_ECODH	*rfbC*	dTDP-4-deoxyrhamnose-3,5-epimerase	11,351	21270,111	5,476	2		327884	219377	8,0	10,4	0,67	0,00	0,00
B1X6X6_ECODH	*rfbD*	dTDP-4-dehydrorhamnose reductase subunit, NAD(P)-b	11,037	32694,137	5,543	3		475325	419905	26,1	18,3	0,88	0,47	0,55
B1XAG2_ECODH	*rffA*	dTDP-4-keto-6-deoxy-D-glucose aminotransferase	31,383	41901,031	5,759	7		665084	1123813	7,1	5,5	1,69	0,00	0,00
B1XAF8_ECODH	*rffD*	UDP-N-acetyl-D-mannosamine dehydrogenase	10,238	45838,734	5,223	2		244700	705557	52,6	6,7	2,88	0,01	0,02
B1XAF7_ECODH	*rffE*	UDP-N-acetylglucosamine 2-epimerase	14,362	42244,594	5,92	3		253235	462380	9,4	12,0	1,83	0,00	0,00
B1XAF9_ECODH	*rffG*	dTDP-glucose 4,6-dehydratase	21,408	39753,848	5,628	5		593170	592173	10,0	6,1	1,00	0,98	0,98

B1XAG0_ECODH	*rffH*	Glucose-1-phosphate thymidylyltransferase	7,167	32734,406	5,317	2		102257	163516	9,3	6,7	1,60	0,00	0,00
RHLB_ECODH	*rhlB*	ATP-dependent RNA helicase RhlB	29,216	47125,918	7,29	9		1231780	1973855	10,6	6,9	1,60	0,00	0,00
B1X7C7_ECODH	*rhlE*	ATP-dependent RNA helicase RhlE	8,59	49989,316	10,057	3		864493	775398	23,9	6,0	0,90	0,39	0,47
B1X9Z7_ECODH	*rho*	Transcription termination factor Rho	55,131	47004,211	6,751	18		13544094	15561565	3,8	3,1	1,15	0,00	0,00
RIBA_ECODH	*ribA*	GTP cyclohydrolase-2	12,755	21835,979	5,596	3		157336	219299	14,1	16,1	1,39	0,03	0,05
RIBB_ECODH	*ribB*	3,4-dihydroxy-2-butanone 4-phosphate synthase	15,668	23353,467	4,898	3		1134658	1100827	9,2	2,8	0,97	0,57	0,63
B1XFX2_ECODH	*ribC*	Riboflavin synthase, alpha subunit	34,742	23444,896	5,642	6		1073484	1179029	11,1	3,3	1,10	0,15	0,20
B1XF02_ECODH	*ribD*	Riboflavin biosynthesis protein RibD	14,714	40338,312	7,219	4		493399	443716	14,8	1,2	0,90	0,20	0,26
B1XBF0_ECODH	*ribF*	Bifunctional riboflavin kinase and FAD synthetase	22,364	34734,266	9,336	4		304553	260785	9,6	9,5	0,86	0,06	0,10
RISB_ECODH	*ribH*	6,7-dimethyl-8-ribityllumazine synthase	48,077	16156,511	5,155	5		4356575	5276520	14,2	20,9	1,21	0,18	0,24
B1X646_ECODH	*rihA*	Pyrimidine-specific ribonucleoside hydrolase RihA	8,039	33822,922	4,836	2		418068	202587	15,3	3,2	0,48	0,00	0,00
RIHC_ECODH	*rihC*	Non-specific ribonucleoside hydrolase RihC	21,053	32560,77	5,23	3	metabolism	180215	75739	30,1	13,2	0,42	0,00	0,01
RIMM_ECODH	*rimM*	Ribosome maturation factor RimM	43,956	20605,434	4,608	5		1874458	1211938	8,9	9,7	0,65	0,00	0,00
RIMO_ECODH	*rimO*	Ribosomal protein S12 methylthiotransferase RimO	18,367	49581,793	5,305	7		961022	586446	4,5	5,0	0,61	0,00	0,00
RIMP_ECODH	*rimP*	Ribosome maturation factor RimP	12	16650,975	4,597	2		206234	151050	19,1	8,1	0,73	0,03	0,05
B1XDT1_ECODH	*rlmB*	23S rRNA (guanosine-2'-O-)-methyltransferase RlmB	11,111	26556,629	6,174	2		231485	198253	2,4	2,4	0,86	0,00	0,00
RLME_ECODH	*rlmE*	Ribosomal RNA large subunit methyltransferase E	34,45	23335,076	9,435	4		458604	405125	5,0	1,6	0,88	0,00	0,01
RLMF_ECODH	*rlmF*	Ribosomal RNA large subunit methyltransferase F	24,026	34225,922	9,297	4		250260	168364	15,0	14,6	0,67	0,01	0,02
RLMG_ECODH	*rlmG*	Ribosomal RNA large subunit methyltransferase G	12,963	42331,449	6,318	3		162296	143181	4,5	13,5	0,88	0,13	0,18
RLMH_ECODH	*rlmH1*	Ribosomal RNA large subunit methyltransferase H	14,839	17341,111	8,709	2		95805	93785	24,4	2,8	0,98	0,87	0,89
RLMI_ECODH	*rlmI*	Ribosomal RNA large subunit methyltransferase I	14,394	44356,922	7,602	4		618760	515396	7,4	6,6	0,83	0,01	0,02
RLMKL_ECODH	*rlmL*	Ribosomal RNA large subunit methyltransferase K/L	16,382	78854,102	8,964	10		734700	523619	8,7	9,0	0,71	0,00	0,01
RLMM_ECODH	*rlmM*	Ribosomal RNA large subunit methyltransferase M	4,918	41905,199	7,038	2		115309	85206	13,4	17,0	0,74	0,03	0,05
RLMN_ECODH	*rlmN*	Dual-specificity RNA methyltransferase RlmN	12,5	43085,531	6,562	4		743327	469437	12,2	3,9	0,63	0,00	0,00
B1X628_ECODH	*rlpA*	Minor lipoprotein	16,022	37527,996	5,515	2		506182	854365	9,7	2,4	1,69	0,00	0,00
B1X636_ECODH	*rlpB*	LPS-assembly lipoprotein LptE	33,161	21356,531	8,73	5		608219	952685	16,6	7,9	1,57	0,00	0,01
B1XBL8_ECODH	*rluB*	Pseudouridine synthase	21,649	32711,385	10,029	6		894983	574416	6,3	10,9	0,64	0,00	0,00
B1X9J9_ECODH	*rluC*	Pseudouridine synthase	15,047	36026,574	9,85	5		339266	472814	30,2	4,5	1,39	0,07	0,11
B1X608_ECODH	*rna*	Ribonuclease I	11,567	29617,588	8,863	2		123934	270139	25,5	16,0	2,18	0,00	0,01
RNB_ECODH	*rnb*	Exoribonuclease 2	36,801	72490,781	5,402	17		3773601	2953880	4,1	4,1	0,78	0,00	0,00
RNC_ECODH	*rnc*	Ribonuclease 3	19,027	25550,043	6,404	5		654657	823340	10,1	2,2	1,26	0,00	0,01
B1X9J7_ECODH	*rne*	Ribonuclease E	28,087	118196,727	5,476	18		6379687	5157347	9,4	2,1	0,81	0,01	0,01
B1X607_ECODH	*rnk*	Regulator of nucleoside diphosphate kinase	48,529	14927,063	4,483	3		565329	404990	9,7	6,9	0,72	0,00	0,00
RNPA_ECODH	*rnpA*	Ribonuclease P protein component	22,689	13789,198	11,815	2		273953	216041	18,3	5,8	0,79	0,05	0,08
B1XDT0_ECODH	*rnr*	Ribonuclease R	18,696	92109,195	8,777	12		868030	1383422	21,6	6,5	1,59	0,00	0,01
B1XFW3_ECODH	*rnt*	Ribonuclease T	11,163	23522,695	5,187	2		134493	115608	14,7	12,8	0,86	0,20	0,26
RODZ_ECODH	*rodZ*	Cytoskeleton protein RodZ	19,585	36191,668	5,585	4		1359394	1624647	8,1	6,4	1,20	0,02	0,03
B1XD58_ECODH	*rof*	Modulator of Rho-dependent transcription termination	20,238	9479,644	4,628	1		153116	332595	7,7	8,7	2,17	0,00	0,00
B1X733_ECODH	*rpe*	Ribulose-phosphate 3-epimerase	19,111	24554,254	5,127	3		447075	643989	13,2	6,4	1,44	0,00	0,01
RPIA_ECODH	*rpiA*	Ribose-5-phosphate isomerase A	39,269	22860,395	5,204	5		2863507	3870780	16,2	3,5	1,35	0,01	0,02
RL1_ECODH	*rplA*	50S ribosomal protein L1	51,709	24729,635	9,644	9		64228539	52402512	7,6	4,4	0,82	0,00	0,01
RL2_ECODH	*rplB*	50S ribosomal protein L2	49,084	29860,443	10,931	11		43964925	34214653	14,9	10,9	0,78	0,03	0,06
RL3_ECODH	*rplC*	50S ribosomal protein L3	43,541	22243,516	9,905	7		37954226	32824099	15,7	9,9	0,86	0,17	0,23
RL4_ECODH	*rplD*	50S ribosomal protein L4	47,264	22086,529	9,724	8		37411476	31289134	9,3	3,1	0,84	0,01	0,02
RL5_ECODH	*rplE*	50S ribosomal protein L5	55,307	20301,572	9,491	8		39074845	22583190	4,0	8,7	0,58	0,00	0,00
RL6_ECODH	*rplF*	50S ribosomal protein L6	71,186	18903,783	9,71	9		44319846	38307138	12,0	5,4	0,86	0,08	0,12
RL9_ECODH	*rplI*	50S ribosomal protein L9	61,074	15769,064	6,165	9		50988485	43039079	11,0	4,0	0,84	0,02	0,05
RL10_ECODH	*rplJ*	50S ribosomal protein L10	50,909	17711,588	9,038	6		55896250	42283397	8,8	2,7	0,76	0,00	0,00
RL11_ECODH	*rplK*	50S ribosomal protein L11	59,155	14875,379	9,641	6		43922745	41278544	18,2	8,5	0,94	0,57	0,64
RL7_ECODH	*rplL*	50S ribosomal protein L7/L12	43,802	12295,196	4,603	4		25739150	18337430	8,4	3,0	0,71	0,00	0,00
RL13_ECODH	*rplM*	50S ribosomal protein L13	46,479	16018,543	9,905	5		28773198	22381636	9,7	6,5	0,78	0,00	0,01
RL14_ECODH	*rplN*	50S ribosomal protein L14	43,089	13541,023	10,431	4		24679780	21702751	4,6	3,3	0,88	0,00	0,01
RL15_ECODH	*rplO*	50S ribosomal protein L15	40,278	14980,424	11,185	5		39498758	28279982	12,1	1,6	0,72	0,00	0,00
RL16_ECODH	*rplP*	50S ribosomal protein L16	32,353	15281,202	11,225	3		34402625	22854809	11,4	7,5	0,66	0,00	0,00
RL17_ECODH	*rplQ*	50S ribosomal protein L17	25,984	14364,586	11,052	3		22908959	18362017	10,4	9,8	0,80	0,02	0,04
RL19_ECODH	*rplS*	50S ribosomal protein L19	47,826	13133,235	10,619	5		33850823	28416713	9,5	13,2	0,84	0,08	0,12
RL20_ECODH	*rplT*	50S ribosomal protein L20	22,881	13496,957	11,471	3		15058102	11701756	9,7	1,3	0,78	0,00	0,01
RL21_ECODH	*rplU*	50S ribosomal protein L21	51,456	11564,349	9,846	4		19469356	17327951	14,4	10,1	0,89	0,23	0,30
RL22_ECODH	*rplV*	50S ribosomal protein L22	56,364	12226,292	10,233	5		22803080	14902731	14,3	6,1	0,65	0,00	0,01
B1X6G9_ECODH	*rplW*	50S ribosomal protein L23	12	11199,123	9,935	1		7603	3373	33,3	54,8	0,44	0,16	0,22
RL24_ECODH	*rplX*	50S ribosomal protein L24	36,538	11316,215	10,211	3		9594030	6905297	11,3	7,8	0,72	0,00	0,01
RL25_ECODH	*rplY*	50S ribosomal protein L25	56,383	10693,44	9,599	5		13011180	8044622	6,5	8,7	0,62	0,00	0,00
RL27_ECODH	*rpmA*	50S ribosomal protein L27	29,412	9124,472	10,585	2		228775	213101	7,0	7,0	0,93	0,20	0,26
RL28_ECODH	*rpmB*	50S ribosomal protein L28	21,795	9006,487	11,421	2		4181870	2395919	9,7	10,4	0,57	0,00	0,00
RL29_ECODH	*rpmC*	50S ribosomal protein L29	46,032	7273,449	9,984	2		30284618	23453729	5,4	10,3	0,77	0,00	0,01
RL30_ECODH	*rpmD*	50S ribosomal protein L30	33,898	6541,79	10,956	2		6426706	5367628	20,1	15,0	0,84	0,18	0,24
RL31_ECODH	*rpmE*	50S ribosomal protein L31	51,429	7871,062	9,461	3		11712955	10532997	11,1	10,7	0,90	0,22	0,29
RL32_ECODH	*rpmF*	50S ribosomal protein L32	26,316	6446,383	11,032	1		2627072	1430956	22,6	5,6	0,54	0,00	0,01
RL33_ECODH	*rpmG*	50S ribosomal protein L33	38,182	6371,585	10,248	2		4239245	3303687	16,4	10,1	0,78	0,04	0,07
RL34_ECODH	*rpmH*	50S ribosomal protein L34	17,391	5380,389	13,002	1		1896784	1908774	18,1	6,0	1,01	0,95	0,96
B1X6E7_ECODH	*rpoA*	DNA-directed RNA polymerase subunit alpha	51,976	36511,723	4,982	13		20783490	22846386	4,6	6,8	1,10	0,06	0,09
RPOB_ECODH	*rpoB*	DNA-directed RNA polymerase subunit beta	48,137	150632,344	5,148	43		54385226	48618463	2,3	1,2	0,89	0,00	0,00
RPOC_ECODH	*rpoC*	DNA-directed RNA polymerase subunit beta'	43,852	155160,25	6,667	44		45947836	38314903	8,7	1,2	0,83	0,01	0,01
B1XG72_ECODH	*rpoD*	RNA polymerase sigma factor RpoD	19,739	70263,281	4,69	10		1732538	1724103	14,8	4,9	1,00	0,95	0,96

B1XBQ0_ECODH	*rpoE*	RNA polymerase sigma factor	17,277	21695,738	5,382	2		123236	118631	32,2	9,8	0,96	0,83	0,86
B1XHH7_ECODH	*rpoN*	RNA polymerase sigma-54 factor	11,74	53989,785	4,627	4		194446	246374	6,9	17,4	1,27	0,05	0,08
B1XCR7_ECODH	*rpoS*	RNA polymerase sigma factor RpoS	12,424	37971,859	4,888	3		161979	241503	10,1	2,0	1,49	0,00	0,00
RPOZ_ECODH	*rpoZ*	DNA-directed RNA polymerase subunit omega	59,341	10236,573	4,87	4		2205921	1970607	3,6	7,9	0,89	0,04	0,07
B1X851_ECODH	*rpsA*	30S ribosomal protein S1	42,908	61158,074	4,892	17		50209639	39747381	7,0	4,5	0,79	0,00	0,00
RS2_ECODH	*rpsB*	30S ribosomal protein S2	70,539	26743,641	6,613	12		40152851	30835285	4,2	5,6	0,77	0,00	0,00
RS4_ECODH	*rpsD*	30S ribosomal protein S4	46,117	23469,094	10,052	8		45939708	42756695	8,3	4,7	0,93	0,18	0,24
B1X6F5_ECODH	*rpsE*	30S ribosomal protein S5	62,275	17603,379	10,109	7		73024084	59487689	11,5	4,4	0,81	0,01	0,03
RS6_ECODH	*rpsF*	30S ribosomal protein S6	67,176	15187,034	5,258	7		4084834	10520559	58,1	12,0	2,58	0,01	0,02
RS7_ECODH	*rpsG*	30S ribosomal protein S7	50,279	20019,092	10,365	7		62180848	48260725	12,5	7,1	0,78	0,01	0,03
RS8_ECODH	*rpsH*	30S ribosomal protein S8	47,692	14126,548	9,441	5		1494267	977879	5,4	11,7	0,65	0,00	0,00
RS9_ECODH	*rpsI*	30S ribosomal protein S9	46,154	14856,199	10,944	6		26693006	17739108	8,1	2,9	0,66	0,00	0,00
RS10_ECODH	*rpsJ*	30S ribosomal protein S10	48,544	11735,587	9,681	5		20073888	15599172	9,8	6,7	0,78	0,01	0,01
RS11_ECODH	*rpsK*	30S ribosomal protein S11	27,132	13844,926	11,328	3		19873662	19278828	18,0	5,9	0,97	0,75	0,80
B1X6F0_ECODH	*rpsM*	30S ribosomal protein S13	61,864	13099,385	10,783	7		20150831	18280534	7,4	7,3	0,91	0,11	0,16
RS14_ECODH	*rpsN*	30S ribosomal protein S14	19,802	11580,483	11,163	2		369332	495938	34,8	16,3	1,34	0,15	0,20
RS15_ECODH	*rpsO*	30S ribosomal protein S15	15,73	10268,765	10,402	2		1614962	4522089	71,8	15,8	2,80	0,02	0,03
RS16_ECODH	*rpsP*	30S ribosomal protein S16	45,122	9190,556	10,543	3		10426181	9065929	12,6	11,8	0,87	0,15	0,21
RS18_ECODH	*rpsR*	30S ribosomal protein S18	29,333	8986,432	10,595	2		13843561	10026344	12,6	7,4	0,72	0,01	0,01
RS19_ECODH	*rpsS*	30S ribosomal protein S19	55,435	10430,286	10,524	4		3205338	5503892	38,5	15,4	1,72	0,03	0,05
RS20_ECODH	*rpsT*	30S ribosomal protein S20	24,138	9684,388	11,182	2		21840670	17586322	9,8	8,1	0,81	0,01	0,03
RS21_ECODH	*rpsU*	30S ribosomal protein S21	39,437	8499,957	11,15	3		5737306	9139927	45,3	12,4	1,59	0,07	0,11
RRAA_ECODH	*rraA*	Regulator of ribonuclease activity A	39,752	17360,365	4,071	4		609472	722599	11,4	8,5	1,19	0,05	0,08
B1XBP9_ECODH	*rseA*	Anti-sigma factor	12,963	24321,375	5,111	1		94341	180938	46,0	24,7	1,92	0,04	0,07
B1XD45_ECODH	*rseP*	Inner membrane zinc RIP metalloprotease	9,111	49071,324	6,628	3		161723	129577	10,4	5,4	0,80	0,01	0,02
RSGA_ECODH	*rsgA*	Putative ribosome biogenesis GTPase RsgA	12,286	39193,305	5,594	3		426203	454560	7,3	10,6	1,07	0,35	0,42
RSMA_ECODH	*rsmA*	Ribosomal RNA small subunit methyltransferase A	27,106	30420,139	7,822	5		448642	558293	21,8	5,2	1,24	0,10	0,14
RSMB_ECODH	*rsmB*	Ribosomal RNA small subunit methyltransferase B	33,566	48347,512	7,165	8		776497	704694	24,7	9,5	0,91	0,51	0,58
RSMC_ECODH	*rsmC*	Ribosomal RNA small subunit methyltransferase C	10,204	37624,73	5,999	3		289721	226880	6,3	7,4	0,78	0,00	0,01
RSMF_ECODH	*rsmF*	Ribosomal RNA small subunit methyltransferase F	8,559	53227,547	5,441	2		93870	79550	8,6	8,9	0,85	0,03	0,06
RSMG_ECODH	*rsmG*	Ribosomal RNA small subunit methyltransferase G	10,628	23431,119	6,062	1		35576	24294	13,8	9,0	0,68	0,00	0,01
RSMH_ECODH	*rsmH*	Ribosomal RNA small subunit methyltransferase H	18,53	34877,816	6,067	4		542936	462953	6,9	5,9	0,85	0,01	0,02
B1XF73_ECODH	*rstA*	DNA-binding response regulator in two-component regu	18,595	27048,248	5,418	4		153194	161949	12,0	11,0	1,06	0,52	0,59
B1X881_ECODH	*rsuA*	Pseudouridine synthase	12,554	25865,303	5,747	2		277809	266813	13,3	4,2	0,96	0,58	0,64
RSXC_ECODH	*rsxC*	Electron transport complex subunit RsxC	6,351	80171,547	8,884	3		54311	53775	24,8	4,3	0,99	0,94	0,95
B1XBP5_ECODH	*sapA*	Predicted antimicrobial peptide transporter subunit perip	5,667	61564,898	6,847	2		125212	74588	7,9	14,1	0,60	0,00	0,00
B1X6U7_ECODH	*sbcB*	Exodeoxyribonuclease I	5,263	54500,812	5,446	2		97400	79701	14,6	3,9	0,82	0,03	0,06
B1XEW6_ECODH	*sbmA*	Predicted transporter	7,143	46459,391	8,458	2	transport	34265	28373	8,2	4,9	0,83	0,01	0,02
B1X6U5_ECODH	*sbmC*	DNA gyrase inhibitor	10,828	18081,463	4,612	1		53327	64272	33,2	29,5	1,21	0,43	0,51
B1XH81_ECODH	*sdaA*	L-serine deaminase I	19,604	48906,645	5,178	7	metabolism	1562565	782935	9,4	3,3	0,50	0,00	0,00
B1XDK6_ECODH	*sdaB*	L-serine deaminase II	29,011	48752,926	5,509	9		4420011	2127418	9,6	31,1	0,48	0,00	0,01
B1XDK5_ECODH	*sdaC*	Predicted serine transporter	11,888	46906,301	9,209	4		1097326	864226	20,6	10,1	0,79	0,08	0,12
B1X6Q4_ECODH	*sdhA*	Succinate dehydrogenase, flavoprotein subunit	33,503	64421,844	5,849	13		10266339	5418089	5,1	4,6	0,53	0,00	0,00
B1X6Q5_ECODH	*sdhB*	Succinate dehydrogenase, FeS subunit	50,42	26769,863	6,314	10		2265945	1387511	23,1	8,8	0,61	0,01	0,01
SECA_ECODH	*secA*	Protein translocase subunit SecA	42,175	102022,992	5,431	26		7036238	4226330	2,6	1,3	0,60	0,00	0,00
SECB_ECODH	*secB*	Protein-export protein SecB	55,484	17277,361	4,265	5		8539453	10380161	4,5	4,4	1,22	0,00	0,00
B1XEZ6_ECODH	*secD*	Protein translocase subunit SecD	33,496	66632,148	8,621	15		2832914	3073628	10,6	3,9	1,08	0,21	0,27
B1XEZ7_ECODH	*secF*	Protein-export membrane protein SecF	12,074	35382,379	5,567	2		589297	388180	8,7	25,0	0,66	0,03	0,05
B1XGY5_ECODH	*secG*	Preprotein translocase membrane subunit	27,273	11365,157	6,091	2		2904428	2750128	9,5	7,3	0,95	0,39	0,47
B1X6F2_ECODH	*secY*	Protein translocase subunit SecY	16,253	48511,688	9,894	5		1980504	1556689	12,8	8,5	0,79	0,02	0,03
B1X8K6_ECODH	*selB*	Selenocysteinyl-tRNA-specific translation factor	17,427	68867,445	6,107	7		431678	437074	6,1	6,4	1,01	0,79	0,83
B1XGM5_ECODH	*selD*	Selenide, water dikinase	45,245	36687,254	5,299	9		3253475	2956933	9,7	9,7	0,91	0,23	0,29
B1X6M0_ECODH	*seqA*	Negative modulator of initiation of replication	30,387	20315,447	8,815	4		702313	778331	8,7	5,7	1,11	0,09	0,13
B1XEJ8_ECODH	*serA*	D-3-phosphoglycerate dehydrogenase	20	44175,777	5,921	6		289084	697019	13,8	7,6	2,41	0,00	0,00
B1XFJ8_ECODH	*serB*	3-phosphoserine phosphatase	10,559	35042,691	5,492	3		164011	157629	7,5	8,2	0,96	0,50	0,58
SERC_ECODH	*serC*	Phosphoserine aminotransferase	37,293	39783,309	5,369	11		2351280	4002604	6,6	4,8	1,70	0,00	0,00
SYS_ECODH	*serS*	Serine--tRNA ligase	34,651	48414,023	5,34	12		8339481	7086686	6,7	8,2	0,85	0,02	0,04
B1X9L3_ECODH	*sixA*	Phosphohistidine phosphatase	8,696	17207,773	4,454	2		81676	75166	14,8	20,6	0,92	0,54	0,61
B1XD47_ECODH	*skp*	Chaperone protein skp	22,36	17688,234	9,694	3		6459720	7517933	7,4	3,1	1,16	0,01	0,02
SLMA_ECODH	*slmA*	Nucleoid occlusion factor SlmA	16,162	22836,344	8,783	2		146629	117897	6,2	16,9	0,80	0,05	0,08
B1X7W1_ECODH	*slp*	Outer membrane lipoprotein	29,787	20963,914	6,822	3		6614950	8090153	9,1	12,8	1,22	0,04	0,07
B1XFK2_ECODH	*slt*	Lytic murein transglycosylase, soluble	23,256	73353,156	8,847	11	eptidoglycan-modificatio	399524	435270	8,2	6,9	1,09	0,15	0,21
B1XFV3_ECODH	*slyB*	Outer membrane lipoprotein	30,968	15601,666	9,356	3		6702449	5926878	18,5	8,2	0,88	0,24	0,31
B1X6J8_ECODH	*slyD*	Peptidyl-prolyl cis-trans isomerase	11,735	20852,832	4,856	2		2061134	1861063	8,3	9,2	0,90	0,15	0,21
SLYX_ECODH	*slyX*	Protein SlyX	38,889	8214,33	4,889	2		206106	226922	12,8	5,9	1,10	0,23	0,29
B1XBT7_ECODH	*smpA*	Outer membrane protein assembly factor BamE	17,699	12302,034	8,71	1		929886	1147144	12,7	15,0	1,23	0,07	0,11
B1XB75_ECODH	*sodA*	Superoxide dismutase	12,136	23097,098	6,444	2		1260496	1035028	6,1	11,0	0,82	0,02	0,04
B1XFW7_ECODH	*sodB*	Superoxide dismutase	44,56	21265,779	5,581	5		3321777	4802113	33,5	22,3	1,45	0,12	0,17
B1XFV7_ECODH	*sodC*	Superoxide dismutase [Cu-Zn]	10,405	17680,977	5,947	1		#ZAHL!	30880	#DIV/0!	22,1	#NV	#NV	#NV
B1XGU2_ECODH	*sohA*	Predicted regulator	13,514	12358,941	5,109	1		60651	59411	5,2	9,9	0,98	0,72	0,77
B1XBM1_ECODH	*sohB*	Predicted inner membrane peptidase	12,034	39366,441	9,241	3		97908	149160	12,3	14,0	1,52	0,00	0,01
MTOX_ECODH	*solA*	N-methyl-L-tryptophan oxidase	23,925	40902,004	5,142	6		465987	550679	12,1	9,8	1,18	0,08	0,12
B1XFA0_ECODH	*speA*	Biosynthetic arginine decarboxylase	31,307	73898,445	4,832	13		2869198	2876111	7,6	6,7	1,00	0,96	0,97
SPEB_ECODH	*speB*	Agmatinase	25,49	33557,043	5,142	6		1621784	1252301	7,5	6,3	0,77	0,00	0,01

SPED_ECODH	*speD*	S-adenosylmethionine decarboxylase proenzyme	13,636	30384,643	5,19	2		210159	227205	11,9	8,2	1,08	0,32	0,39
SPEE_ECODH	*speE*	Spermidine synthase	23,611	32321,391	5,33	3		590995	714356	8,4	9,3	1,21	0,02	0,04
B1XF49_ECODH	*speG*	Spermidine N1-acetyltransferase	28,495	21887,014	6,201	4		626163	696944	10,4	7,3	1,11	0,14	0,20
B1X984_ECODH	*spoU*	tRNA (Guanosine-2'-O-)-methyltransferase	10,48	25343,074	6,706	2		115848	119965	10,3	9,5	1,04	0,65	0,70
B1XGM7_ECODH	*sppA*	Protease IV (Signal peptide peptidase)	19,256	67219,336	5,724	7		554022	589266	7,9	1,4	1,06	0,18	0,25
B1XGK4_ECODH	*spy*	Envelope stress induced periplasmic protein	38,509	18199,072	9,66	6	stress response	430101	433347	23,8	10,3	1,01	0,96	0,96
B1XCN1_ECODH	*srlB*	Glucitol/sorbitol-specific enzyme IIA component of PTS	51,22	13304,049	4,957	3		2633225	953496	7,8	6,6	0,36	0,00	0,00
B1XCN2_ECODH	*srlD*	Sorbitol-6-phosphate dehydrogenase	58,301	27857,877	5,922	10		2952693	965527	13,1	6,0	0,33	0,00	0,00
B1XCN0_ECODH	*srlE*	Glucitol/sorbitol-specific enzyme IIB component of PTS	17,555	33332,039	6,516	4		1214495	351690	3,0	4,5	0,29	0,00	0,00
B1XCN4_ECODH	*srlR*	DNA-bindng transcriptional repressor	8,949	28236,148	5,217	2		61065	39105	24,0	13,8	0,64	0,02	0,04
B1XBQ3_ECODH	*srmB*	ATP-dependent RNA helicase SrmB	27,928	49914,141	9,273	9		1597581	1489722	12,1	3,4	0,93	0,32	0,39
B1XCU6_ECODH	*ssb*	Single-stranded DNA-binding protein	16,854	18974,996	5,441	3		1492187	1243884	11,0	4,5	0,83	0,02	0,04
B1XDH7_ECODH	*sscR*	6-carboxy-5,6,7,8-tetrahydropterin synthase	15,702	13772,88	6,037	1		36786	51966	13,4	26,6	1,41	0,07	0,11
B1XAZ6_ECODH	*sseA*	3-mercaptopyruvate sulfurtransferase	23,132	30811,84	4,562	6		1486572	1215472	16,4	5,0	0,82	0,05	0,08
B1XHK2_ECODH	*sspA*	Stringent starvation protein A	44,34	24304,908	5,221	7		4153052	3465751	9,4	5,0	0,83	0,02	0,03
B1XHK1_ECODH	*sspB*	ClpXP protease specificity-enhancing factor	21,212	18262,416	4,377	3		399718	336548	10,0	10,3	0,84	0,06	0,09
STHA_ECODH	*sthA*	Soluble pyridine nucleotide transhydrogenase	27,253	51560,395	6,084	6		2461945	899462	8,8	1,8	0,37	0,00	0,00
B1XCK7_ECODH	*stpA*	DNA-binding protein	30,597	15347,512	7,95	3		5231610	2488590	8,6	8,1	0,48	0,00	0,00
B1X6Q6_ECODH	*sucA*	2-oxoglutarate decarboxylase, thiamin-requiring	33,441	105061,719	6,038	23		10894610	7466289	4,4	1,3	0,69	0,00	0,00
B1X6Q7_ECODH	*sucB*	Dihydrolipoyltranssuccinase	28,889	44011,395	5,576	9		13005169	8292133	10,3	6,0	0,64	0,00	0,00
SUCC_ECODH	*sucC*	Succinyl-CoA ligase [ADP-forming] subunit beta	40,722	41392,645	5,367	13		14918991	9487362	8,8	4,7	0,64	0,00	0,00
B1X6Q9_ECODH	*sucD*	Succinyl-CoA ligase [ADP-forming] subunit alpha	46,021	29777,477	6,318	9		5049959	3810921	9,6	6,3	0,75	0,00	0,01
B1XFZ2_ECODH	*sufA*	Fe-S cluster assembly protein	16,393	13300,113	4,848	1		105006	130915	11,9	17,5	1,25	0,07	0,11
B1XFZ1_ECODH	*sufB*	Component of SufBCD complex	8,687	54745,316	5,058	3		48123	78002	24,3	5,1	1,62	0,01	0,01
B1XFZ0_ECODH	*sufC*	Component of SufBCD complex, ATP-binding componen	11,29	27582,367	4,84	3		33116	92116	54,6	17,0	2,78	0,01	0,02
B1XFY9_ECODH	*sufD*	Component of SufBCD complex	5,674	46822,75	6,358	2		52298	58493	16,8	7,1	1,12	0,27	0,34
B1XFG8_ECODH	*sufI*	Cell division protein FtsP	15,319	51858,164	5,935	5		262052	243977	12,8	8,9	0,93	0,40	0,48
B1XB08_ECODH	*suhB*	Inositol monophosphatase	38,577	29172,127	6,448	7		3186870	2731601	9,6	5,1	0,86	0,03	0,05
B1XC54_ECODH	*surA*	Chaperone SurA	36,682	47283,715	6,478	11		3288813	3605134	6,8	2,3	1,10	0,04	0,08
B1XAD5_ECODH	*talA*	Transaldolase	33,544	35658,801	5,889	9		4495993	5695594	22,5	4,3	1,27	0,08	0,12
B1XBD3_ECODH	*talB*	Transaldolase	60,252	35219,246	5,106	15		35799809	40150056	3,6	5,5	1,12	0,01	0,03
TAM_ECODH	*tam*	Trans-aconitate 2-methyltransferase	10,317	29005,959	4,855	2		91907	99943	9,9	5,4	1,09	0,19	0,25
B1XDP0_ECODH	*tas*	Predicted oxidoreductase, NADP(H)-dependent aldo-ket	6,647	38499,539	6,266	2		204733	271062	12,1	2,6	1,32	0,00	0,01
B1XAK1_ECODH	*tatB*	Sec-independent protein translocase protein TatB	14,035	18420,869	5,133	2		125491	188573	17,5	10,6	1,50	0,01	0,01
B1X622_ECODH	*tatE*	Probable Sec-independent protein translocase protein T	16,418	7024,294	9,05	1		93079	78985	15,8	5,3	0,85	0,09	0,14
B1XGT2_ECODH	*tdcB*	Catabolic threonine dehydratase, PLP-dependent	23,1	35232,414	5,749	5		1556274	70119	12,0	14,8	0,05	0,00	0,00
B1XGS9_ECODH	*tdcE*	Pyruvate formate-lyase 4/2-ketobutyrate formate-lyase	17,147	85935,547	5,483	8		6195414	1933747	6,8	12,0	0,31	0,00	0,00
TDH_ECODH	*tdh*	L-threonine 3-dehydrogenase	48,387	37239,043	5,943	10		2782344	2503646	9,8	7,5	0,90	0,14	0,19
B1XAT0_ECODH	*tdk*	Thymidine kinase	28,293	23456,576	5,98	3		433310	403969	8,4	13,9	0,93	0,42	0,49
B1XDE1_ECODH	*tehB*	Predicted S-adenosyl-L-methionine-dependent methyltra	30,457	22530,734	6,836	6		263071	343422	9,2	4,0	1,31	0,00	0,01
B1XFP0_ECODH	*tesB*	Acyl-CoA thioesterase II	9,091	31966,326	6,173	2		135110	133207	13,8	13,5	0,99	0,89	0,91
TGT_ECODH	*tgt*	Queuine tRNA-ribosyltransferase	8,533	42593,66	5,975	3		125627	153117	23,2	5,4	1,22	0,15	0,21
THII_ECODH	*thiI*	tRNA sulfurtransferase	21,162	54973,098	6,162	10		1499820	1033333	10,2	3,1	0,69	0,00	0,00
B1XBC7_ECODH	*thrA*	Fused aspartokinase I and homoserine dehydrogenase	45,854	89120,242	5,465	25	metabolism	3427310	2893635	4,3	3,5	0,84	0,00	0,00
KHSE_ECODH	*thrB*	Homoserine kinase	13,548	33623,645	5,453	2	metabolism	142783	129566	16,2	5,5	0,91	0,32	0,39
B1XBC9_ECODH	*thrC*	Threonine synthase	36,215	47113,836	5,238	10		2051717	1704085	7,7	2,5	0,83	0,00	0,01
SYT_ECODH	*thrS*	Threonine--tRNA ligase	21,651	74014,297	5,802	13		3702019	4399759	16,7	7,7	1,19	0,10	0,14
B1XDN3_ECODH	*thyA*	Thymidylate synthase	21,97	30479,688	5,62	5		718407	698501	12,1	11,1	0,97	0,74	0,80
TIG_ECODH	*tig*	Trigger factor	57,176	48192,668	4,825	20		53856473	38135566	6,2	0,5	0,71	0,00	0,00
B1XF97_ECODH	*tktA*	Transketolase	26,998	72211,742	5,429	13		14282430	19276970	3,6	7,4	1,35	0,00	0,00
B1XAD6_ECODH	*tktB*	Transketolase	19,34	73042,719	5,863	9		1351591	2241759	12,7	7,7	1,66	0,00	0,00
B1XHL6_ECODH	*tldD*	Predicted peptidase	24,532	51364,094	4,93	7		746325	733700	2,8	6,5	0,98	0,65	0,71
KTHY_ECODH	*tmk*	Thymidylate kinase	24,883	23783,143	5,326	4		771246	603141	26,7	8,0	0,78	0,10	0,15
TNAA_ECODH	*tnaA*	Tryptophanase	65,605	52773,465	5,876	21		216942465	34285647	10,4	4,5	0,16	0,00	0,00
B1X6S0_ECODH	*tolA*	Membrane anchored protein in TolA-TolQ-TolR complex	7,838	43156,625	9,097	2		56836	59250	18,5	11,0	1,04	0,74	0,79
TOLB_ECODH	*tolB*	Protein TolB	41,628	45955,539	6,979	12		3905275	5027246	11,8	8,5	1,29	0,01	0,03
B1XG40_ECODH	*tolC*	Transport channel	54,97	53740,723	5,461	16		10467846	12849285	12,1	2,0	1,23	0,02	0,03
B1X6R8_ECODH	*tolQ*	Membrane spanning protein in TolA-TolQ-TolR complex	13,043	25597,717	6,53	2		199136	248213	12,6	14,5	1,25	0,07	0,11
B1XBM3_ECODH	*topA*	DNA topoisomerase 1	20,578	97349,758	8,678	13		2564909	2001220	12,4	3,7	0,78	0,01	0,02
TPIS_ECODH	*tpiA*	Triosephosphate isomerase	48,235	26971,812	5,637	6		5623175	6463983	7,3	4,6	1,15	0,02	0,03
B1XCF2_ECODH	*tpx*	Probable thiol peroxidase	44,643	17835,316	4,75	5		9125338	10491848	3,2	6,2	1,15	0,01	0,02
TREA_ECODH	*treA*	Periplasmic trehalase	5,31	63636,719	5,6	2		38822	58876	12,2	11,7	1,52	0,00	0,01
B1XEM1_ECODH	*treB*	Fused trehalose(Maltose)-specific PTS enzyme: IIB com	21,142	51080,742	9,139	7		6556994	1664574	7,0	10,1	0,25	0,00	0,00
B1XEM0_ECODH	*treC*	Trehalose-6-P hydrolase	21,96	63837,672	5,513	11		8307341	2094555	2,5	2,5	0,25	0,00	0,00
TRMA_ECODH	*trmA*	tRNA/tmRNA (uracil-C(5))-methyltransferase	16,667	41966,949	5,707	3		397850	224632	4,5	7,8	0,56	0,00	0,00
TRMD_ECODH	*trmD*	tRNA (guanine-N(1)-)-methyltransferase	10,588	28422,359	5,502	2		153857	106852	11,9	13,5	0,69	0,01	0,02
B1XFC0_ECODH	*trmI*	tRNA (guanine-N(7)-)-methyltransferase	14,644	27307,322	6,417	3		382752	192506	16,6	21,6	0,50	0,00	0,01
TRPA_ECODH	*trpA*	Tryptophan synthase alpha chain	22,761	28724,162	5,314	4		182293	232183	18,4	11,2	1,27	0,07	0,11
TRPB_ECODH	*trpB*	Tryptophan synthase beta chain	7,305	42983,008	5,709	2		189680	179115	11,8	9,2	0,94	0,46	0,54
B1X731_ECODH	*trpS*	Tryptophan--tRNA ligase	32,934	37437,82	6,267	8		1883796	2164248	17,5	7,7	1,15	0,20	0,26
TRUA_ECODH	*truA*	tRNA pseudouridine synthase A	10,37	30399,619	8,683	2		82231	73612	21,7	14,7	0,90	0,48	0,55
B1XGX8_ECODH	*truB*	tRNA pseudouridine synthase B	29,936	35087,078	5,705	6		483916	344591	15,4	12,3	0,71	0,01	0,03
TRUD_ECODH	*truD*	tRNA pseudouridine synthase D	27,794	39091,488	6,153	6		556589	556635	13,2	5,1	1,00	1,00	1,00
B1X9Z5_ECODH	*trxA*	Thioredoxin	46,789	11806,623	4,674	4		6981308	20151196	15,4	4,6	2,89	0,00	0,00

B1X828_ECODH	*trxB*	Thioredoxin reductase	62,305	34623,035	5,295	12		5068777	5829025	9,6	2,2	1,15	0,03	0,05
TSAC_ECODH	*tsaC*	Threonylcarbamoyl-AMP synthase	28,947	20767,738	4,941	3		244473	287850	10,2	11,9	1,18	0,08	0,12
TSAD_ECODH	*tsaD*	tRNA N6-adenosine threonylcarbamoyltransferase	6,825	36008,406	5,915	2		81214	83048	21,6	18,9	1,02	0,88	0,90
EFTS_ECODH	*tsf*	Elongation factor Ts	49,47	30422,979	5,215	11		32316195	31538777	8,0	6,8	0,98	0,67	0,72
B1XEZ9_ECODH	*tsx*	Nucleoside channel, receptor of phage T6 and colicin K	42,177	33589,023	5,07	8		8925515	10560521	9,0	10,9	1,18	0,06	0,09
TTCA_ECODH	*ttcA*	tRNA 2-thiocytidine biosynthesis protein TtcA	28,939	35560,914	5,953	6		586470	348309	3,1	6,0	0,59	0,00	0,00
B1XBY2_ECODH	*tufB*	Elongation factor Tu				19		534128784	448564332	2,9	4,6	0,84	0,00	0,00
TUSA_ECODH	*tusA*	Sulfurtransferase TusA	23,457	9094,543	5,181	1		97180	76982	42,1	11,4	0,79	0,27	0,34
TUSB_ECODH	*tusB*	Protein TusB	12,632	10718,333	5,115	1		43024	29757	15,4	24,9	0,69	0,05	0,08
B1XBS2_ECODH	*tyrA*	T-protein	7,775	42042,523	5,682	2		28274	31842	5,6	23,7	1,13	0,37	0,45
B1XCU2_ECODH	*tyrB*	Tyrosine aminotransferase, tyrosine-repressible, PLP-de	41,562	43537,809	5,318	12		1204543	1597349	14,6	2,7	1,33	0,01	0,02
B1XCF1_ECODH	*tyrR*	DNA-binding transcriptional dual regulator, tyrosine-bind	12,865	57656,141	5,543	5		226553	141365	7,6	10,6	0,62	0,00	0,00
SYY_ECODH	*tyrS*	Tyrosine--tRNA ligase	26,651	47526,969	5,59	9		1880290	2561644	9,2	8,2	1,36	0,00	0,01
B1XAK5_ECODH	*ubiD*	3-octaprenyl-4-hydroxybenzoate carboxy-lyase	19,718	55603,75	5,31	7		739822	1140379	13,2	2,9	1,54	0,00	0,00
UBIE_ECODH	*ubiE*	Ubiquinone/menaquinone biosynthesis C-methyltransfer	33,068	28073,211	7,771	7		1568345	2132098	14,3	3,6	1,36	0,01	0,01
UBIG_ECODH	*ubiG*	Ubiquinone biosynthesis O-methyltransferase	42,5	26555,451	6,119	6		739010	712863	9,4	7,5	0,96	0,57	0,64
B1XA97_ECODH	*ucpA*	Predicted oxidoredutase, sulfate metabolism protein	20,152	27849,975	5,126	4		903457	299893	3,9	4,5	0,33	0,00	0,00
B1X702_ECODH	*udk*	Uridine kinase	12,676	24353,115	6,393	3		354055	330988	9,3	10,7	0,93	0,38	0,46
B1XAJ5_ECODH	*udp*	Uridine phosphorylase	69,565	27159,082	5,813	10		6045186	15398084	18,0	8,2	2,55	0,00	0,00
B1XF82_ECODH	*uidA*	Beta-D-glucuronidase	21,891	68447	5,244	9	metabolism	316805	199774	10,4	7,3	0,63	0,00	0,00
UPP_ECODH	*upp*	Uracil phosphoribosyltransferase	48,558	22533,264	5,317	7		6777178	3608883	9,1	4,0	0,53	0,00	0,00
B1X930_ECODH	*usg*	Predicted semialdehyde dehydrogenase	20,475	36364,133	4,381	3		364235	362665	12,2	3,9	1,00	0,95	0,96
B1XFR7_ECODH	*ushA*	Bifunctional UDP-sugar hydrolase and 5'-nucleotidase	13,091	60823,973	5,475	5		556841	710002	5,2	4,0	1,28	0,00	0,00
B1XCG2_ECODH	*uspE*	Stress-induced protein	27,848	35706,879	5,157	5		968793	71013	4,1	23,8	0,07	0,00	0,00
B1XCK3_ECODH	*uspF*	Stress-induced protein, ATP-binding protein	18,056	16016,532	5,603	2		713848	363043	11,0	14,3	0,51	0,00	0,00
B1X605_ECODH	*uspG*	Universal stress protein UP12	49,296	15935,185	6,028	5		1541950	1436481	6,6	10,3	0,93	0,30	0,37
B1X8Q3_ECODH	*uup*	Fused predicted transporter subunits of ABC superfamily	9,921	72066,883	5,355	3		347461	188077	16,6	16,8	0,54	0,00	0,01
UVRB_ECODH	*uvrB*	UvrABC system protein B	3,269	76226,055	5,14	2		63712	56988	19,0	16,0	0,89	0,42	0,49
B1XAH9_ECODH	*uvrD*	DNA-dependent ATPase I and helicase II	17,222	81989,883	5,857	10		504218	855968	7,9	2,8	1,70	0,00	0,00
B1X672_ECODH	*uvrY*	DNA-binding response regulator in two-component regu	22,477	23892,654	6,528	4		148162	332652	29,7	6,9	2,25	0,00	0,00
UXAC_ECODH	*uxaC*	Uronate isomerase	25,957	53987,18	5,438	8		886999	324422	5,7	4,8	0,37	0,00	0,00
B1X9L9_ECODH	*vacJ*	Predicted lipoprotein	17,928	28041,602	4,895	3		409319	470947	18,7	4,9	1,15	0,17	0,23
B1XEN7_ECODH	*valS*	Valine--tRNA ligase	34,595	108192,352	5,199	24		9604324	9385872	6,2	5,0	0,98	0,58	0,64
B1X6X0_ECODH	*wbbI*	Conserved protein	26,97	37757,215	5,847	7		2668883	2220606	9,2	8,9	0,83	0,03	0,05
B1X6W8_ECODH	*wbbK*	Lipopolysaccharide biosynthesis protein	23,118	43188,176	9,017	7		802727	563254	4,2	5,6	0,70	0,00	0,00
WECG_ECODH	*wecG*	UDP-N-acetyl-D-mannosaminuronic acid transferase	19,106	27928,182	9,562	3		159654	274655	14,1	5,4	1,72	0,00	0,00
B1XAF6_ECODH	*wzzE*	Enterobacterial Common Antigen (ECA) polysaccharide	32,471	39489,102	6,247	6		550883	1095172	13,4	4,0	1,99	0,00	0,00
EX7L_ECODH	*xseA*	Exodeoxyribonuclease 7 large subunit	12,281	51832,23	9,992	4		127161	152575	11,5	11,2	1,20	0,07	0,11
EX7S_ECODH	*xseB*	Exodeoxyribonuclease 7 small subunit	35	8951,932	4,429	2		591935	546934	12,1	6,8	0,92	0,28	0,35
B1XGL0_ECODH	*xthA*	Exonuclease III	30,597	30969,166	5,799	8		917699	867464	8,4	5,0	0,95	0,29	0,36
YAAA_ECODH	*yaaA*	UPF0246 protein YaaA	18,217	29585,828	6,859	4		408557	479232	18,1	11,1	1,17	0,19	0,25
B1XCA2_ECODH	*yadG*	Predicted transporter subunit: ATP-binding component c	16,883	34647,066	8,438	4		194820	209001	3,1	6,9	1,07	0,11	0,16
YAEH_ECODH	*yaeH*	UPF0325 protein YaeH	39,062	15096,169	6,614	5		964223	1069696	5,9	10,6	1,11	0,15	0,20
YAEP_ECODH	*yaeP*	UPF0253 protein YaeP	56,061	7214,222	4,536	2		303109	624944	11,8	5,2	2,06	0,00	0,00
B1XD72_ECODH	*yafC*	Predicted DNA-binding transcriptional regulator	8,553	33775,82	6,93	2		32368	69647	54,0	18,8	2,15	0,03	0,05
YAIE_ECODH	*yaiE*	UPF0345 protein YaiE	23,404	10234,396	4,469	2		463908	777680	8,6	13,0	1,68	0,00	0,00
B1XEU7_ECODH	*yaiL*	Nucleoprotein/polynucleotide-associated enzyme	17,318	19922,76	7,834	2		145567	106015	16,5	23,2	0,73	0,07	0,11
B1XEZ5_ECODH	*yajC*	SecYEG protein translocase auxillary subunit	20	11887,143	9,572	2		1152952	1096776	19,4	7,1	0,95	0,64	0,70
B1XEZ8_ECODH	*yajD*	Conserved protein	16,522	13363,993	6,14	1		87268	41758	20,0	34,6	0,48	0,02	0,04
B1XFM2_ECODH	*yajG*	Predicted lipoprotein	40,104	20950,027	8,685	4		1141064	961851	11,1	10,7	0,84	0,07	0,11
B1XF12_ECODH	*yajL*	Conserved protein	25	20777,092	5,245	3		344315	356910	11,0	9,9	1,04	0,65	0,71
B1XF07_ECODH	*yajO*	Predicted oxidoreductase, NAD(P)-binding	27,778	36420,168	5,185	6		284441	458690	7,8	3,0	1,61	0,00	0,00
YAJQ_ECODH	*yajQ*	UPF0234 protein YajQ	53,988	18343,861	5,957	7		6440318	6838344	3,9	7,3	1,06	0,21	0,27
YBAB_ECODH	*ybaB*	Nucleoid-associated protein YbaB	21,101	12014,82	5,012	2		824154	684089	17,9	8,1	0,83	0,11	0,16
B1XFR5_ECODH	*ybaL*	Predicted transporter with NAD(P)-binding Rossmann-fc	5,018	59423,531	4,996	2		119484	131041	11,7	8,1	1,10	0,25	0,32
B1XFS2_ECODH	*ybaS*	Glutaminase	16,452	32903,324	4,814	2		171014	276749	22,7	7,2	1,62	0,01	0,02
B1XFP1_ECODH	*ybaY*	Predicted outer membrane lipoprotein	32,632	19431,199	7,866	3		1254415	1560705	12,3	9,1	1,24	0,03	0,05
B1XFS9_ECODH	*ybbN*	Predicted thioredoxin domain-containing protein	63,028	31791,064	4,499	9		2065000	2562101	15,4	7,6	1,24	0,05	0,08
B1XGC6_ECODH	*ybcJ*	Predicted RNA-binding protein	30	7389,537	7,838	2		136434	388008	55,6	12,8	2,84	0,01	0,01
B1X5X4_ECODH	*ybdF*	Conserved protein	13,115	14050,323	8,71	1		18574	18227	9,8	18,8	0,98	0,87	0,89
B1X5X2_ECODH	*ybdG*	Predicted mechanosensitive channel	6,988	46602,648	7,872	2		143168	168285	29,8	2,6	1,18	0,28	0,35
B1X632_ECODH	*ybeB*	Ribosomal silencing factor RsfS	15,238	11582,228	4,519	1		192910	206660	18,8	15,8	1,07	0,60	0,67
B1X626_ECODH	*ybeD*	UPF0250 protein YbeD	39,08	9827,346	5,497	2		1129884	1039476	8,7	15,0	0,92	0,40	0,47
B1X638_ECODH	*ybeL*	Conserved protein	16,875	18797,201	5,107	3		163310	143911	30,4	12,2	0,88	0,51	0,58
B1X655_ECODH	*ybeZ*	Uncharacterized protein	52,601	39038,699	5,711	12		2496865	2824925	6,6	2,1	1,13	0,01	0,03
B1X6L8_ECODH	*ybfE*	LexA regulated protein	16,495	11279,979	10,086	1		85785	55123	11,4	28,0	0,64	0,02	0,04
B1X6S3_ECODH	*ybgF*	Uncharacterized protein	31,559	28231,404	8,581	4		1237424	1371339	13,3	8,4	1,11	0,26	0,33
B1X6P1_ECODH	*ybgI*	Putative GTP cyclohydrolase 1 type 2	27,53	26892,48	5,067	4		1215452	1986247	3,9	5,7	1,63	0,00	0,00
B1X6P2_ECODH	*ybgJ*	Predicted enzyme subunit	12,844	23946,602	5,162	1		61077	94543	20,6	22,9	1,55	0,03	0,05
B1X6P3_ECODH	*ybgK*	Predicted enzyme subunit	17,742	34386,352	8,591	5		137380	252618	5,9	5,0	1,84	0,00	0,00
B1X796_ECODH	*ybhA*	Pyridoxal phosphatase / fructose 1,6-bisphosphatase	30,147	30201,309	5,506	4		327548	421260	12,9	14,6	1,29	0,04	0,07
B1X7A3_ECODH	*ybhB*	Predicted kinase inhibitor	20,886	17085,121	5,27	2		178622	241894	9,1	9,9	1,35	0,00	0,01
B1X7A2_ECODH	*ybhC*	Predicted pectinesterase	33,255	46082,199	5,662	8		2014217	1107899	4,8	6,8	0,55	0,00	0,00
B1X7D0_ECODH	*ybiB*	Predicted transferase/phosphorylase	16,875	35048,539	6,377	4		263535	340577	14,4	3,0	1,29	0,01	0,03

B1X7E9_ECODH	*ybiS*	Conserved protein	44,118	33325,277	5,992	7		1975076	3009192	14,1	11,1	1,52	0,00	0,01
B1X7F0_ECODH	*ybiT*	Fused predicted transporter subunits of ABC superfamily	21,698	59857,996	4,989	8		1746348	1455560	5,2	6,5	0,83	0,00	0,01
B1X7F2_ECODH	*ybiV*	Predicted hydrolase	23,616	30412,828	5,909	4		175960	207049	15,5	12,6	1,18	0,16	0,22
B1X806_ECODH	*ybjP*	Predicted lipoprotein	42,69	18991,309	6,052	5		1293169	1748133	8,0	5,6	1,35	0,00	0,00
B1X818_ECODH	*ybjX*	Conserved protein	24,242	38357,551	9,437	5		739998	551893	7,6	6,0	0,75	0,00	0,00
B1X837_ECODH	*ycaC*	Predicted hydrolase	31,25	23100,314	5,201	4		615297	1005148	15,3	4,9	1,63	0,00	0,00
B1X845_ECODH	*ycaO*	Conserved protein	14,676	65651,578	4,379	6		2285868	1630000	2,6	10,0	0,71	0,00	0,00
B1X8M9_ECODH	*ycbB*	Predicted carboxypeptidase	7,317	67812,492	8,634	3	peptidoglycan-modification	23424	48931	16,1	13,2	2,09	0,00	0,00
B1X8N1_ECODH	*ycbL*	Predicted metal-binding enzyme	39,07	23784,051	4,946	5		940440	1103870	10,4	7,2	1,17	0,05	0,08
B1X8Q1_ECODH	*ycbX*	Predicted 2Fe-2S cluster-containing protein	10,298	40644,41	7,577	2		130136	109313	15,3	21,2	0,84	0,23	0,30
B1X8Q9_ECODH	*ycbZ*	Predicted peptidase	16,041	65818,656	4,689	5		337914	376214	11,7	10,7	1,11	0,24	0,31
B1X8R5_ECODH	*yccF*	Conserved inner membrane protein	14,865	16275,364	9,412	2		44816	64356	16,2	17,2	1,44	0,02	0,04
B1X9C4_ECODH	*yccJ*	Uncharacterized protein	25,333	8524,493	4,701	1		366409	405592	21,0	10,9	1,11	0,46	0,53
B1X8R9_ECODH	*yccU*	Predicted CoA-binding protein with NAD(P)-binding Ros	18,248	14701,123	6,723	2		254998	380420	17,4	10,0	1,49	0,01	0,02
YCDX_ECODH	*ycdX*	Probable phosphatase YcdX	8,163	26890,512	5,534	2		89464	109903	24,6	10,7	1,23	0,19	0,25
B1X9E9_ECODH	*ycdY*	Conserved protein	16,304	20784,387	4,209	2		742367	649966	10,0	8,7	0,88	0,09	0,13
B1X9H6_ECODH	*yceB*	Predicted lipoprotein	25,806	20499,74	6,15	3		379816	434759	7,9	9,4	1,14	0,06	0,10
B1X9K0_ECODH	*yceF*	Maf-like protein YceF	24,227	21690,799	5,887	3		174398	170492	37,8	13,7	0,98	0,92	0,94
YCEH_ECODH	*yceH*	UPF0502 protein YceH	49,767	24177,33	5,02	7		876840	950755	7,3	4,8	1,08	0,11	0,16
B1XA38_ECODH	*ycfD*	Conserved protein	26,005	42578,918	4,694	6		527282	572538	2,3	2,8	1,09	0,00	0,01
B1XA15_ECODH	*ycfM*	Penicillin-binding protein activator LpoB	36,15	22515,645	6,412	5		695778	912649	20,2	6,6	1,31	0,04	0,06
YCFP_ECODH	*ycfP*	UPF0227 protein YcfP	31,667	21226,176	6,132	5		1079210	897349	6,7	7,6	0,83	0,01	0,02
B1XA23_ECODH	*ycfS*	Conserved protein	7,188	34619,887	8,887	2	peptidoglycan-modification	3596	4959	86,4	66,2	1,38	0,83	0,87
B1XA65_ECODH	*ycgK*	Uncharacterized protein	22,556	14905,824	9,498	2		58717	37627	14,4	25,2	0,64	0,02	0,04
YCGL_ECODH	*ycgL*	Protein YcgL	42,593	12414,504	9,162	3		385043	488032	19,1	4,7	1,27	0,07	0,11
B1XA67_ECODH	*ycgM*	Predicted isomerase/hydrolase	10,959	23711,117	5,879	1		165946	204962	24,9	12,1	1,24	0,22	0,29
YCGN_ECODH	*ycgN*	UPF0260 protein YcgN	12,418	17910,371	4,899	1		64202	45661	9,6	7,7	0,71	0,00	0,00
B1XAP4_ECODH	*ychF*	Ribosome-binding ATPase YchF	61,708	39667,32	4,87	15		8302648	7501109	5,5	6,9	0,90	0,06	0,10
B1XAR3_ECODH	*ychN*	Conserved protein	24,786	12692,747	5,018	2		138007	181598	22,5	9,7	1,32	0,06	0,10
B1XBK6_ECODH	*yciE*	Conserved protein	11,31	18961,447	4,939	1		30444	17120	8,7	16,8	0,56	0,00	0,00
B1XBK7_ECODH	*yciF*	Conserved protein	18,072	18597,164	5,474	2		211326	135941	26,4	7,3	0,64	0,02	0,04
B1XBM0_ECODH	*yciK*	Predicted oxoacyl-(Acyl carrier protein) reductase, EmrK	15,079	27932,912	7,666	3		149321	147590	6,0	5,7	0,99	0,79	0,83
B1XBM2_ECODH	*yciN*	Uncharacterized protein	31,325	9385,636	5,474	2		589908	782405	11,7	10,4	1,33	0,01	0,02
B1XBL6_ECODH	*yciO*	Conserved protein	24,272	23211,758	5,969	4		607359	586870	9,3	10,1	0,97	0,64	0,70
B1XBN5_ECODH	*yciT*	Predicted DNA-binding transcriptional regulator	15,261	27602,605	5,986	2		264278	250101	10,0	11,2	0,95	0,48	0,56
YCJF_ECODH	*ycjF*	UPF0283 membrane protein YcjF	8,499	39392,203	8,743	2		84785	93410	20,3	16,0	1,10	0,49	0,56
B1XCE9_ECODH	*ycjX*	Conserved protein with nucleoside triphosphate hydrolas	10,108	52609,484	8,246	3		58917	60966	24,9	9,3	1,03	0,80	0,84
B1XD92_ECODH	*ydbK*	Pyruvate-flavodoxin oxidoreductase	5,111	128824,445	5,523	5		352694	377708	7,3	7,3	1,07	0,24	0,30
B1XDC7_ECODH	*ydcF*	Conserved protein	14,286	29705,711	5,501	3		251940	237774	4,5	6,5	0,94	0,20	0,26
B1XDE2_ECODH	*ydcL*	Predicted lipoprotein	11,261	24426,971	8,401	1		91790	87228	28,0	15,7	0,95	0,78	0,83
B1XE86_ECODH	*yddB*	Predicted porin protein	4,051	89282,883	6,006	2	transport	36372	56363	2,2	16,5	1,55	0,00	0,01
B1XE89_ECODH	*ydeN*	Conserved protein	41,071	62802,266	5,67	12		3070302	285365	2,3	8,3	0,09	0,00	0,00
B1XEC6_ECODH	*ydfG*	L-allo-threonine dehydrogenase, NAD(P)-binding	19,758	27248,932	5,652	4		2030177	2073619	14,0	3,7	1,02	0,78	0,82
B1XEC8_ECODH	*ydfZ*	Conserved protein	34,328	7276,347	7,941	2		223801	9671	24,4	21,5	0,04	0,00	0,00
B1XF79_ECODH	*ydgA*	Conserved protein	28,486	54688,996	5,068	10		3120119	3399467	8,0	3,2	1,09	0,09	0,13
B1XF69_ECODH	*ydgH*	Uncharacterized protein	37,261	33903,293	9,271	10		2119249	2555616	6,0	4,1	1,21	0,00	0,01
B1XF90_ECODH	*ydgT*	Predicted regulator	53,521	8416,578	6,249	3		199028	214905	12,6	11,3	1,08	0,39	0,47
B1XFV8_ECODH	*ydhF*	Predicted oxidoreductase	21,477	33675,648	5,765	4		971607	785049	7,0	16,2	0,81	0,05	0,09
B1XFX4_ECODH	*ydhQ*	Conserved protein	16,986	42876,008	4,423	6		770461	938736	8,7	3,2	1,22	0,01	0,01
B1XFX5_ECODH	*ydhR*	Putative mono-oxygenase	32,673	11287,857	5,085	3		324672	452872	34,6	11,6	1,39	0,09	0,13
B1XFZ3_ECODH	*ydiH*	Uncharacterized protein	13,483	10453,968	6,398	1		13464	16932	36,1	11,2	1,26	0,27	0,34
B1XGM6_ECODH	*ydjA*	Predicted oxidoreductase	28,415	20059,006	6,314	4		789739	903634	9,1	7,7	1,14	0,06	0,10
B1XGJ0_ECODH	*ydjN*	Predicted transporter	5,832	48661,691	9,059	2		115129	118398	4,9	17,2	1,03	0,76	0,81
B1XGN7_ECODH	*yeaC*	Conserved protein	18,889	10338,001	5,232	1		77101	102446	12,7	19,9	1,33	0,06	0,09
B1XGP0_ECODH	*yeaD*	Conserved protein	35,374	32666,096	5,89	7		2312174	4657167	15,7	4,0	2,01	0,00	0,00
B1XGP3_ECODH	*yeaG*	Conserved protein with nucleoside triphosphate hydrolas	11,491	74480,336	5,627	6		351129	393148	14,9	8,9	1,12	0,25	0,32
B1XGP7_ECODH	*yeaK*	Conserved protein	21,557	17850,643	6,509	3		224411	238251	14,1	13,8	1,06	0,58	0,64
B1XGQ2_ECODH	*yeaO*	Conserved protein	10,435	13386,214	6,29	1		30542	67275	35,3	11,2	2,20	0,01	0,02
B1XGR6_ECODH	*yeaY*	Predicted lipoprotein	22,28	20921,123	9,55	3		194671	382042	26,5	8,0	1,96	0,00	0,01
B1XH75_ECODH	*yeaZ*	Predicted peptidase	30,736	25180,812	5,016	4		230089	238393	7,1	6,5	1,04	0,48	0,56
B1XHD2_ECODH	*yebC*	Probable transcriptional regulatory protein YebC	12,195	26422,561	4,714	2		1338810	1090624	15,1	5,9	0,81	0,05	0,08
B1XHB4_ECODH	*yebE*	Conserved protein	38,356	23686,846	5,343	5	unknown	217522	150976	9,9	3,8	0,69	0,00	0,00
B1XHB5_ECODH	*yebF*	Protein YebF	27,049	13464,311	8,806	2		26061	96456	35,8	21,4	3,70	0,00	0,00
B1XHB6_ECODH	*yebG*	Conserved protein regulated by LexA	45,833	10717,01	4,352	2		215975	150789	7,5	9,0	0,70	0,00	0,00
B1XHC1_ECODH	*yebK*	Predicted DNA-binding transcriptional regulator	11,073	31975,572	6,181	2		91936	41145	5,6	9,7	0,45	0,00	0,00
B1XHA0_ECODH	*yebR*	Conserved protein	19,126	20277,213	4,676	3		353129	631803	21,0	2,8	1,79	0,00	0,00
B1XHA4_ECODH	*yebV*	Uncharacterized protein	24,359	8752,796	4,465	1		106328	99777	6,7	5,2	0,94	0,19	0,25
B1X669_ECODH	*yecA*	Conserved metal-binding protein	21,267	25039,277	4,523	3		393274	390548	11,2	11,2	0,99	0,93	0,95
B1X673_ECODH	*yecF*	Uncharacterized protein	21,622	8238,546	5,25	1		110143	53411	15,9	1,3	0,48	0,00	0,00
B1X664_ECODH	*yecJ*	Uncharacterized protein	48,193	9122,583	4,884	3		499018	318715	11,3	20,1	0,64	0,01	0,02
B1XHE3_ECODH	*yecM*	Predicted metal-binding enzyme	13,83	21205,014	5,325	2		46462	50053	9,0	23,5	1,08	0,59	0,66
B1X685_ECODH	*yedD*	Uncharacterized protein	31,387	14983,258	4,867	3		709504	631569	4,8	12,0	0,89	0,12	0,17
B1X6C8_ECODH	*yeeN*	Probable transcriptional regulatory protein YeeN	29,412	25867,105	4,713	5		1094623	1193457	9,6	7,3	1,09	0,20	0,27
B1X6T6_ECODH	*yeeR*	CP4-44 prophage predicted membrane protein	5,49	57236,668	5,773	2		48752	4750	23,6	38,4	0,10	0,00	0,00

B1X6U3_ECODH	*yeeX*	Conserved protein	33,588	15132,504	9,161	2		447143	380443	5,8	6,6	0,85	0,01	0,02
B1X6V2_ECODH	*yeeZ*	Predicted epimerase, with NAD(P)-binding Rossmann-fo	22,993	29679,943	5,278	4		278428	444766	5,9	8,3	1,60	0,00	0,00
B1X7G5_ECODH	*yegD*	Predicted chaperone	11,333	49371,391	5,113	3		190912	145541	14,9	13,5	0,76	0,04	0,06
B1X7H6_ECODH	*yegP*	Uncharacterized protein	22,727	12024,296	9,435	2		134352	165719	20,1	6,8	1,23	0,08	0,12
B1X7H7_ECODH	*yegQ*	Predicted peptidase	8,609	51193,184	5,797	4		364504	321265	9,9	9,2	0,88	0,11	0,16
B1X7M0_ECODH	*yehZ*	Predicted transporter subunit: periplasmic-binding comp	14,426	32609,303	5,816	2		44570	123138	28,6	19,9	2,76	0,00	0,01
SFGH2_ECODH	*yeiG*	S-formylglutathione hydrolase YeiG	10,432	31259,279	5,177	1		30547	36825	16,9	11,2	1,21	0,11	0,15
EFPL_ECODH	*yeiP*	Elongation factor P-like protein	10	21532,631	4,924	2		305831	39532	17,2	22,0	0,13	0,00	0,00
B1X7N5_ECODH	*yeiT*	Predicted oxidoreductase	6,553	44329,465	5,265	2		169205	49337	19,5	15,2	0,29	0,00	0,00
B1X872_ECODH	*yeiU*	Undecaprenyl pyrophosphate phosphatase	9,283	26759,127	9,88	2		332271	16513	2,0	48,5	0,05	0,00	0,00
NDPA_ECODH	*yejK*	Nucleoid-associated protein YejK	24,179	37822,516	4,876	6		1106731	610405	7,2	6,5	0,55	0,00	0,00
YEJL_ECODH	*yejL*	UPF0352 protein YejL	36	8288,458	5,497	1		31537	73839	50,9	30,0	2,34	0,04	0,06
B1X902_ECODH	*yfbQ*	Predicted aminotransferase	17,284	45517,371	5,847	5		339815	463634	10,5	2,6	1,36	0,00	0,00
B1X904_ECODH	*yfbT*	Predicted hydrolase or phosphatase	16,667	23007,521	5,739	2		95159	110230	9,6	12,6	1,16	0,10	0,15
YFBU_ECODH	*yfbU*	UPF0304 protein YfbU	52,439	19536,203	6,065	9		1830132	2616996	8,5	5,8	1,43	0,00	0,00
B1X910_ECODH	*yfcD*	Predicted NUDIX hydrolase	42,778	20375,861	4,698	5		494240	828162	15,4	2,4	1,68	0,00	0,00
B1X911_ECODH	*yfcE*	Predicted phosphatase	28,804	20122,078	5,632	3		147535	357888	23,8	17,3	2,43	0,00	0,00
B1X936_ECODH	*yfcL*	Uncharacterized protein	55,435	10000,043	4,26	3		185904	320542	13,8	11,7	1,72	0,00	0,00
YFCN_ECODH	*yfcN*	UPF0115 protein YfcN	14,754	21013,318	8,405	1		121849	94261	16,8	7,9	0,77	0,03	0,06
B1X9L6_ECODH	*yfcZ*	Conserved protein	27,66	10317,643	4,25	2		3341617	1003084	6,6	10,4	0,30	0,00	0,00
B1X9M3_ECODH	*yfdH*	CPS-53 (KpLE1) prophage bactoprenol glucosyl transfer	22,549	34635,434	7,784	5		688769	723741	3,8	14,2	1,05	0,53	0,60
B1X9M4_ECODH	*yfdI*	CPS-53 (KpLE1) prophage predicted inner membrane p	9,255	51483,586	8,792	3		136034	156311	18,5	10,5	1,15	0,26	0,33
B1X9Q1_ECODH	*yfdZ*	Prediected aminotransferase, PLP-dependent	14,563	46216,215	7,71	3		221355	280445	15,5	5,7	1,27	0,03	0,05
B1X9R8_ECODH	*yfeD*	Predicted DNA-binding transcriptional regulator	12,308	14910,144	7,768	1		44921	16180	5,8	15,7	0,36	0,00	0,00
B1XAA2_ECODH	*yfeX*	Conserved protein	8,696	33052,266	5,336	2		321952	259197	11,3	11,7	0,81	0,04	0,07
B1XAA3_ECODH	*yfeY*	Uncharacterized protein	20,942	20897,609	5,213	2		142943	229299	13,8	7,0	1,60	0,00	0,00
B1XAE2_ECODH	*yffB*	Conserved protein	31,356	13600,587	6,091	3		544839	498052	13,1	7,4	0,91	0,30	0,37
B1XAC1_ECODH	*yffS*	Uncharacterized protein	6,093	31041,461	5,453	2		73701	73979	13,2	8,0	1,00	0,96	0,97
B1XAW9_ECODH	*yfgC*	Predicted peptidase	6,982	53907,922	7,084	2		91511	145784	12,9	14,9	1,59	0,00	0,01
B1XAX0_ECODH	*yfgD*	Predicted oxidoreductase	30,252	13398,582	5,895	2		402329	661725	8,0	9,4	1,64	0,00	0,00
B1XAX9_ECODH	*yfgG*	Uncharacterized protein	23,81	7461,619	11,888	1	unknown	75691	74503	16,5	2,5	0,98	0,87	0,89
B1XAY7_ECODH	*yfgL*	Outer membrane protein assembly factor BamB	30,102	41887,211	4,718	7		2051627	3405116	25,0	3,8	1,66	0,01	0,01
B1XAY8_ECODH	*yfgM*	Conserved protein	46,602	22176,055	5,065	6		1921559	1854602	6,3	7,1	0,97	0,49	0,56
B1XAZ9_ECODH	*yfhJ*	Conserved protein	24,242	7731,568	3,982	1		192103	176676	12,1	12,7	0,92	0,38	0,46
B1XAZ5_ECODH	*yfhM*	Conserved protein	2,057	181585,453	5,255	2		55531	54988	18,5	18,0	0,99	0,94	0,95
B1XB07_ECODH	*yfhQ*	Predicted methyltransferase	26,423	27047,938	5,687	5		796558	757594	7,7	8,7	0,95	0,42	0,50
B1XBR9_ECODH	*yfiA*	Cold shock protein associated with 30S ribosomal subur	53,097	12784,585	6,204	4		3639036	2157328	9,3	3,8	0,59	0,00	0,00
B1XBQ8_ECODH	*yfiF*	Predicted methyltransferase	35,072	37784,375	8,936	6		1458535	1370543	26,8	4,3	0,94	0,64	0,70
B1XBR8_ECODH	*yfiO*	Outer membrane protein assembly factor BamD	50,612	27829,396	6,158	10		3272970	3830716	6,4	3,0	1,17	0,00	0,01
B1XBT9_ECODH	*yfjG*	Conserved protein	10,759	17726,67	8,598	1		144174	74335	21,6	22,1	0,52	0,00	0,01
B1XCM7_ECODH	*ygaD*	Conserved protein	25,455	17581,719	5,061	3		300905	297562	9,9	11,4	0,99	0,89	0,91
B1XBX0_ECODH	*ygaU*	Uncharacterized protein	44,295	16063,117	5,708	6		4129999	5790097	14,0	7,6	1,40	0,01	0,01
B1XDI8_ECODH	*ygcF*	7-carboxy-7-deazaguanine synthase	26,457	25029,645	5,71	5		263130	264075	10,2	5,6	1,00	0,95	0,96
B1XDK4_ECODH	*ygdH*	Conserved protein	28,194	50971,891	6,046	8		845030	478447	6,1	2,7	0,57	0,00	0,00
B1XDL8_ECODH	*ygdI*	Uncharacterized protein	26,667	8174,202	5,599	1		1037095	784210	10,0	9,1	0,76	0,01	0,01
B1XDM0_ECODH	*ygdK*	Predicted Fe-S metabolism protein	13,605	15940,271	6,114	1		95264	97206	12,4	20,5	1,02	0,88	0,90
YGFZ_ECODH	*ygfZ*	tRNA-modifying protein YgfZ	27,607	36094,117	5,175	7		1668542	1872667	6,3	2,4	1,12	0,01	0,03
B1XEK5_ECODH	*yggE*	Conserved protein	31,707	26635,264	6,1	7		993479	1554091	7,1	1,4	1,56	0,00	0,00
B1XF98_ECODH	*yggG*	Predicted peptidase	26,19	26842,318	5,748	5		352972	747536	19,3	8,7	2,12	0,00	0,00
B1XFA6_ECODH	*yggJ*	Ribosomal RNA small subunit methyltransferase E	19,753	26978,062	6,183	4		212690	197529	12,7	10,1	0,93	0,40	0,47
B1XFB9_ECODH	*yggL*	Uncharacterized protein	12,963	12880,56	4,907	1		70260	50794	25,6	19,9	0,72	0,12	0,17
B1XFB8_ECODH	*yggN*	Uncharacterized protein	8,368	26429,213	8,968	2		49363	177181	57,4	5,0	3,59	0,00	0,01
B1XFB1_ECODH	*yggS*	Predicted enzyme	17,949	25787,436	6,092	3		217451	240480	25,2	5,2	1,11	0,52	0,59
B1XFB4_ECODH	*yggV*	dITP/XTP pyrophosphatase	38,579	21038,844	5,214	4		540275	454075	6,6	7,0	0,84	0,01	0,02
FETP_ECODH	*yggX*	Probable Fe(2+)-trafficking protein	52,747	10952,529	5,914	4		1287392	2338989	9,7	11,6	1,82	0,00	0,00
B1XFF5_ECODH	*yghZ*	Aldo-keto reductase	30,925	38832,16	6,723	6		543947	185257	2,5	9,5	0,34	0,00	0,00
B1XG42_ECODH	*ygiB*	Conserved outer membrane protein	34,555	19816,033	8,251	4	metabolism	419436	413934	20,0	7,3	0,99	0,90	0,92
B1XG43_ECODH	*ygiC*	Predicted enzyme	37,306	45026,016	4,688	12	metabolism	1201046	630395	5,9	9,5	0,52	0,00	0,00
B1XG59_ECODH	*ygiF*	Predicted adenylate cyclase	23,095	48388,953	5,731	8		617604	773774	11,1	3,9	1,25	0,01	0,02
B1XG34_ECODH	*ygiN*	Quinol monooxygenase	23,077	11532,391	5,793	3		441010	658844	14,9	8,8	1,49	0,00	0,01
B1XG26_ECODH	*ygiT*	Predicted DNA-binding transcriptional regulator	11,45	14703,1	9,098	1		46100	50938	12,8	21,0	1,10	0,47	0,54
B1XG29_ECODH	*ygiW*	Conserved protein	13,846	14010,758	5,076	2		791540	1214859	10,4	7,6	1,53	0,00	0,00
B1XG78_ECODH	*ygjH*	Conserved protein	10	12315,344	5,012	1		41479	11009	20,4	11,7	0,27	0,00	0,00
B1XGS2_ECODH	*yhaJ*	Predicted DNA-binding transcriptional regulator	6,711	33256,406	6,051	2		53888	59355	15,0	13,8	1,10	0,38	0,46
B1XHH6_ECODH	*yhbG*	Predicted transporter subunit: ATP-binding component c	32,78	26800,648	5,64	5		1181701	1137442	9,1	8,6	0,96	0,57	0,64
YHBJ_ECODH	*yhbJ*	UPF0042 nucleotide-binding protein YhbJ	25	32492,285	6,724	4		243183	294373	9,4	16,0	1,21	0,09	0,13
B1XHH5_ECODH	*yhbN*	Predicted transporter subunit: periplasmic-binding comp	10,27	20126,885	8,962	2		237541	224392	14,2	11,3	0,94	0,55	0,61
B1XGZ0_ECODH	*yhbY*	Predicted RNA-binding protein	21,649	10783,711	9,415	2		489384	712141	51,7	6,8	1,46	0,24	0,31
B1XHK6_ECODH	*yhcB*	Conserved protein	52,273	14960,849	5,648	5		3517374	4650370	9,3	3,3	1,32	0,00	0,00
B1XHM3_ECODH	*yhdH*	Predicted oxidoreductase, Zn-dependent and NAD(P)-bi	31,173	34723,766	5,631	7		1357557	1124511	4,6	2,6	0,83	0,00	0,00
B1X6J5_ECODH	*yheO*	Predicted DNA-binding transcriptional regulator	21,667	26820,564	5,395	3		200797	377626	17,7	21,4	1,88	0,00	0,01
B1X6K2_ECODH	*yheS*	Fused predicted transporter subunits of ABC superfamily	16,797	71843,43	5,5	9		521565	602835	17,3	4,2	1,16	0,15	0,21
B1X754_ECODH	*yhgF*	Predicted transcriptional accessory protein	23,933	85119,867	5,918	15		2283843	2400558	8,0	5,9	1,05	0,34	0,42
B1X7Q6_ECODH	*yhhA*	Conserved protein	34,932	16623,82	10,888	3		130234	964356	24,5	7,7	7,40	0,00	0,00

B1X7S3_ECODH	*yhhF*	Ribosomal RNA small subunit methyltransferase D	12,626	21677,604	5,959	2		94998	89590	12,6	13,4	0,94	0,55	0,62
B1X7R7_ECODH	*yhhK*	Conserved protein	29,921	14505,534	6,585	2		97698	145193	5,8	3,6	1,49	0,00	0,00
B1X7S5_ECODH	*yhhM*	Conserved protein	36,134	13496,499	10,061	3		150822	273319	11,8	8,1	1,81	0,00	0,00
B1X7P8_ECODH	*yhhX*	Predicted oxidoreductase with NAD(P)-binding Rossmar	29,275	38765,008	6,066	7		671157	1017351	9,4	3,4	1,52	0,00	0,00
B1X7V4_ECODH	*yhiR*	Ribosomal RNA large subunit methyltransferase J	13,571	31941,707	8,589	2		180634	129976	12,5	6,7	0,72	0,00	0,01
B1X8E5_ECODH	*yhjJ*	Predicted zinc-dependent peptidase	18,474	55527,371	5,726	6		569682	710719	13,0	5,4	1,25	0,02	0,04
B1X8G7_ECODH	*yiaD*	Predicted outer membrane lipoprotein	52,968	22197,221	9,81	5		2315098	1788054	15,9	10,2	0,77	0,03	0,06
B1X8G9_ECODH	*yiaF*	Conserved protein	27,966	25663,102	6,079	5		1312388	1482038	11,3	4,1	1,13	0,08	0,12
B1X8K7_ECODH	*yibF*	Predicted glutathione S-transferase	7,426	22545,061	5,101	2		35358	50481	3,4	17,0	1,43	0,01	0,01
B1X8L7_ECODH	*yibL*	Conserved protein	18,333	13696,007	9,485	2		160437	173557	9,7	8,2	1,08	0,26	0,33
B1X945_ECODH	*yibN*	Predicted rhodanese-related sulfurtransferase	26,573	15596,255	9,372	2		1667743	1378338	8,4	14,6	0,83	0,06	0,10
B1X8L6_ECODH	*yibT*	Uncharacterized protein	42,029	7995,298	9,395	2		525235	566474	11,7	9,4	1,08	0,37	0,45
B1X977_ECODH	*yicC*	Conserved protein	17,422	33174,926	5,1	5		747717	811903	10,1	4,9	1,09	0,18	0,25
B1X988_ECODH	*yicH*	Conserved protein	11,599	62272,441	5,673	5		310727	324945	17,5	5,6	1,05	0,63	0,69
B1X9S7_ECODH	*yidA*	Predicted hydrolase	12,593	29721,139	5,108	2		318100	385707	12,6	16,6	1,21	0,12	0,17
B1X9S8_ECODH	*yidB*	Conserved protein	34,848	13786,576	4,368	2		148142	131588	2,4	9,0	0,89	0,04	0,07
YIDC_ECODH	*yidC*	Membrane protein insertase YidC	30,657	61525,922	7,7	8		4344267	3568410	10,1	4,7	0,82	0,01	0,03
B1X9U2_ECODH	*yieF*	Chromate reductase, Class I, flavoprotein	50,532	20375,562	5,007	5		527387	739651	6,7	14,7	1,40	0,01	0,01
B1X9Y3_ECODH	*yifE*	Conserved protein	36,607	13133,577	6,102	3		374353	827889	28,2	15,6	2,21	0,00	0,01
B1XAH5_ECODH	*yifL*	Predicted lipoprotein	46,269	7177,217	8,889	2		63781	258905	50,6	8,0	4,06	0,00	0,00
B1XAH8_ECODH	*yigB*	Predicted hydrolase	12,605	27121,842	5,985	2	metabolism	102810	72490	33,7	13,4	0,71	0,12	0,18
B1XAI5_ECODH	*yigI*	Conserved protein	12,258	17162,771	6,423	2		46434	81318	7,2	19,8	1,75	0,00	0,01
B1XAJ1_ECODH	*yigL*	Predicted hydrolase	28,947	29707,729	5,228	4		330567	681504	4,7	5,8	2,06	0,00	0,00
B1XAM1_ECODH	*yihA*	Probable GTP-binding protein EngB	12,121	22083,229	6,769	2		174956	273819	21,4	6,4	1,57	0,01	0,01
B1XAL5_ECODH	*yihD*	Conserved protein	49,438	10272,91	5,179	3		676075	914363	22,1	24,6	1,35	0,13	0,18
B1XAL6_ECODH	*yihE*	Predicted kinase	10,976	38120,316	4,993	3	stress response	160189	83672	51,5	3,8	0,52	0,03	0,05
B1XB77_ECODH	*yiiM*	Conserved protein	14,732	25342,725	5,709	2		143451	221855	18,8	11,3	1,55	0,01	0,02
B1XC02_ECODH	*yjaG*	Conserved protein	22,449	22612,648	4,446	4		415926	320144	8,3	17,1	0,77	0,03	0,06
B1XCT3_ECODH	*yjbJ*	Predicted stress response protein	17,391	8325,252	5,441	1		877523	912164	17,5	16,0	1,04	0,76	0,81
B1XCU5_ECODH	*yjbR*	Conserved protein	26,271	13519,475	6,056	3		160853	348638	9,9	4,4	2,17	0,00	0,00
B1XD15_ECODH	*yjdC*	Predicted transcriptional regulator	27,749	21931,078	4,947	4		166313	436675	17,0	7,6	2,63	0,00	0,00
B1XDR9_ECODH	*yjeE*	ATPase with strong ADP affinity	17,647	16853,078	4,488	2		236724	186346	15,8	5,8	0,79	0,03	0,05
B1XDP8_ECODH	*yjeI*	Conserved protein	30,769	11958,425	5,497	2		1203742	2031676	9,6	6,8	1,69	0,00	0,00
B1XDR3_ECODH	*yjeP*	Predicted mechanosensitive channel	2,439	123967,609	6,551	2		12597	18515	53,8	24,2	1,47	0,21	0,28
YJGA_ECODH	*yjgA*	UPF0307 protein YjgA	15,301	21359,279	5,3	2		173882	183170	6,3	12,4	1,05	0,47	0,55
B1XEM4_ECODH	*yjgF*	Ketoacid-binding protein	64,844	13611,593	5,361	4		5335110	6009570	23,7	4,1	1,13	0,37	0,45
B1XEN1_ECODH	*yjgK*	Conserved protein	22,667	16865,359	5,308	2		190708	151011	11,2	12,3	0,79	0,03	0,06
B1XEP1_ECODH	*yjgQ*	Conserved inner membrane protein	8,056	39618,605	9,48	2		96734	71079	18,9	10,7	0,73	0,04	0,07
B1XEP2_ECODH	*yjgR*	Predicted ATPase	6	54332,293	5,915	2		14306	44842	48,2	31,6	3,13	0,01	0,02
B1XEQ6_ECODH	*yjhC*	KpLE2 phage-like element predicted oxidoreductase	14,247	41384,488	6,102	4	metabolism	502335	371158	8,2	5,8	0,74	0,00	0,00
B1XER9_ECODH	*yjhU*	KpLE2 phage-like element predicted DNA-binding transc	5,488	36111,586	6,326	2		48932	45252	14,2	11,1	0,92	0,43	0,50
B1XFK1_ECODH	*yjjK*	Fused predicted transporter subunits of ABC superfamil	38,018	62442,883	5,431	17		8036433	7598472	5,3	4,4	0,95	0,15	0,21
B1XE48_ECODH	*ykgE*	Predicted oxidoreductase	10,46	26004,295	7,501	2		226379	24608	15,8	15,8	0,11	0,00	0,00
B1XE49_ECODH	*ykgF*	Predicted amino acid dehydrogenase with NAD(P)-bindi	8,211	53051,898	8,749	3		274944	14830	20,3	26,1	0,05	0,00	0,00
B1XE50_ECODH	*ykgG*	Predicted transporter	22,511	25212,707	4,963	3		488072	19171	10,9	23,5	0,04	0,00	0,00
B1X7X9_ECODH	*yliJ*	Predicted glutathione S-transferase	30,288	23713,178	5,05	5		240328	414066	6,8	14,6	1,72	0,00	0,00
B1X8Q6_ECODH	*ymbA*	Conserved protein	28,877	20634,406	5,807	3		261215	234954	8,3	11,4	0,90	0,17	0,23
B1X9F9_ECODH	*ymdB*	O-acetyl-ADP-ribose deacetylase	12,429	18880,385	5,445	1		24623	38412	21,9	14,0	1,56	0,02	0,04
B1XBP6_ECODH	*ymjA*	Uncharacterized protein	30,864	9321,317	4,234	1		53187	57352	16,5	19,6	1,08	0,57	0,64
B1XCF9_ECODH	*ynaI*	Conserved inner membrane protein	19,242	38754,508	9,363	3		172184	286970	17,8	9,7	1,67	0,00	0,01
B1XDG0_ECODH	*yncB*	NADPH-dependent curcumin/dihydrocurcumin reductas	7,536	37609,746	5,526	2		201744	213123	8,2	8,4	1,06	0,39	0,47
B1XDG2_ECODH	*yncD*	Predicted iron outer membrane transporter	4,714	77260,672	5,321	2		42293	35208	48,3	14,5	0,83	0,50	0,57
B1XDG3_ECODH	*yncE*	Conserved protein	29,745	38612,957	9,22	7		696278	781717	7,3	4,5	1,12	0,03	0,06
YNFB_ECODH	*ynfB*	UPF0482 protein YnfB	10,619	12908,668	9,15	1		161289	204630	73,9	16,1	1,27	0,62	0,68
B1XF51_ECODH	*ynfD*	Uncharacterized protein	21,782	10476,697	4,735	1	unknown	45566	72517	23,5	6,6	1,59	0,01	0,02
B1XFY6_ECODH	*ynhG*	Conserved protein	7,784	36082,348	9,422	2		50678	80483	22,0	16,4	1,59	0,02	0,03
B1XGI6_ECODH	*yniA*	Predicted phosphotransferase/kinase	24,476	32458,617	4,979	4		269427	184882	3,4	8,9	0,69	0,00	0,00
B1XGI8_ECODH	*yniC*	Predicted hydrolase	18,018	24330,24	4,797	3		304529	376199	12,6	3,9	1,24	0,02	0,03
B1XH77_ECODH	*yoaB*	Conserved protein	21,93	12493,228	4,964	2		800535	1093216	11,9	2,8	1,37	0,00	0,01
B1XH78_ECODH	*yoaC*	Uncharacterized protein	11,765	13231,266	5,156	1		15203	28706	39,4	16,2	1,89	0,02	0,04
B1XGQ3_ECODH	*yoaF*	Conserved outer membrane protein	16,667	8942,377	7,661	1		39825	33865	25,3	15,8	0,85	0,34	0,42
B1XAA5_ECODH	*ypeA*	Acetyltransferase YpeA	11,348	16311,566	4,835	1		28598	32016	47,9	9,3	1,12	0,73	0,79
B1XAE7_ECODH	*ypfJ*	Conserved protein	14,634	31460,186	5,578	3		141642	319460	27,6	7,8	2,26	0,00	0,00
YQFB_ECODH	*yqfB*	UPF0267 protein YqfB	17,476	11905,412	4,682	1		93092	83976	28,8	9,6	0,90	0,48	0,55
YQGE_ECODH	*yqgE*	UPF0301 protein YqgE	43,85	20685,906	5,343	5		368095	561141	18,7	4,3	1,52	0,01	0,01
RUVX_ECODH	*yqgF*	Putative Holliday junction resolvase	27,536	15186,305	6,741	2		89144	80105	2,4	2,8	0,90	0,00	0,00
B1XFG3_ECODH	*yqhD*	Alcohol dehydrogenase, NAD(P)-dependent	35,917	42097,023	5,72	9		2026968	1805365	12,3	8,2	0,89	0,15	0,21
B1XG47_ECODH	*yqiC*	Conserved protein	16,667	11275,98	5,895	2		203730	475272	38,3	10,5	2,33	0,00	0,01
B1XGA2_ECODH	*yqjD*	Conserved protein	25,743	11051,492	9,046	2		1389828	1554938	16,8	16,0	1,12	0,39	0,47
B1XG74_ECODH	*yqjH*	Predicted siderophore interacting protein	17,717	28871,555	5,532	2	transport	125739	183523	31,9	10,1	1,46	0,06	0,09
B1XG75_ECODH	*yqjI*	Predicted transcriptional regulator	10,145	23401,289	6,266	2		81011	75644	23,3	12,8	0,93	0,62	0,68
B1XGV8_ECODH	*yraL*	Ribosomal RNA small subunit methyltransferase I	15,385	31347,904	5,828	2		123003	82152	9,0	10,7	0,67	0,00	0,00
B1XGV9_ECODH	*yraM*	Penicillin-binding protein activator LpoA	28,171	72825,164	5,265	10		1087232	955612	3,9	4,8	0,88	0,01	0,01
B1XGW2_ECODH	*yraP*	Uncharacterized protein	26,702	20027,949	9,038	3		794735	961990	11,8	3,6	1,21	0,02	0,04

B1XHG5_ECODH	*yrbA*	Predicted DNA-binding transcriptional regulator	29,762	9451,736	5,831	2	351802	537470	15,5	2,5	1,53	0,00	0,00
B1XHG7_ECODH	*yrbC*	Predicted ABC-type organic solvent transporter	37,915	23962,611	9,354	5	256809	605158	32,9	15,4	2,36	0,00	0,01
B1XHG8_ECODH	*yrbD*	Predicted ABC-type organic solvent transporter	44,809	19576,223	4,787	6	1268656	1356533	6,5	5,2	1,07	0,16	0,22
B1XHH0_ECODH	*yrbF*	Predicted toluene transporter subunit: ATP-binding comp	31,599	29096,805	6,157	6	402623	348757	5,4	10,1	0,87	0,05	0,08
B1X6D2_ECODH	*yrdA*	Conserved protein	44,565	20245,023	5,259	6	807223	812377	7,7	12,8	1,01	0,93	0,95
B1XAJ4_ECODH	*ysgA*	Predicted hydrolase	16,605	29425,211	5,569	3	46688	118669	17,6	15,8	2,54	0,00	0,00
B1XDV6_ECODH	*ytfB*	Predicted cell envelope opacity-associated protein	13,679	23505,5	5,063	2	122088	128424	22,0	11,7	1,05	0,70	0,76
B1XDX1_ECODH	*ytfP*	Conserved protein	11,504	12866,424	6,391	1	120010	193887	10,5	9,5	1,62	0,00	0,00
ZAPA_ECODH	*zapA*	Cell division protein ZapA	33,945	12594,17	5,217	3	422337	453252	14,1	10,6	1,07	0,44	0,51
ZAPB_ECODH	*zapB*	Cell division protein ZapB	50,617	9634,807	4,69	3	2708556	3644473	9,8	9,4	1,35	0,00	0,01
ZAPD_ECODH	*zapD*	Cell division protein ZapD	17,004	28291,629	6,31	3	466448	346633	6,4	3,8	0,74	0,00	0,00
B1X7S7_ECODH	*zntA*	Zinc, cobalt and lead efflux system	14,344	76839,875	5,697	7	420791	426615	7,3	4,6	1,01	0,76	0,81
B1XHC5_ECODH	*znuA*	Zinc transporter subunit: periplasmic-binding component	14,194	33777,375	5,608	2	170179	176131	14,4	19,7	1,03	0,79	0,83
B1XCT4_ECODH	*zur*	DNA-binding transcriptional repressor, Zn(II)-binding	11,111	19254,168	5,974	1	23993	43287	39,2	29,4	1,80	0,05	0,09
B1XHC0_ECODH	*zwf*	Glucose-6-phosphate 1-dehydrogenase	39,511	55704,441	5,558	16	3585782	3936860	5,9	3,1	1,10	0,03	0,05
