# Supplementary material for: Molecular and proteome analyses highlight the importance of the Cpx envelope stress system for acid stress and cell wall stability in Escherichia coli
Source: Microbiologyopen. 2016 Apr 2;5(4):582–96. doi: 10.1002/mbo3.353 (PMC4985592; doi:10.1002/mbo3.353)
Supplement: Supplementary file 6 [file MBO3-5-582-s006.pdf]

Table S3C: Proteome profile of *E. coli* and an isogenic *cpxRA* mutant grown under wild-type (WT) and Cpx-inducing (ON, by *nlpE*-overexpression) conditions.

Table S3 is subdivided in the following three tables: Table S3A describes the proteome profiling of WT_{ON} compared to WT, S3B the proteome profiling of *cpxRA* compared to WT, and S3C the proteome profiling of $cpxRA_{ON}$ compared to WT. For all four conditions (WT, WT_{ON}, *cpxRA*, $cpxRA_{ON}$) geometric means of median-normalized protein intensities (normalization performed with Genedata Analyst 8.2 (Genedata, Basel, Switzerland) from four independent biological replicates are displayed for all detected proteins, which were identified with at least two peptides, or when the sequence coverage exceeded 10%, only one peptide was identified, respectively. Further, ratios relative to the WT, p-values from a student's t-test, and multiple testing-corrected q-values according to Benjamini-Hochberg (BH) were determined using Genedata Analyst. Proteins whose intensities resulted in q-values <0.05 (highlighted green) and exceeded an absolute fold change of 2 were regarded as significantly different in the compared conditions. Ratios >2 were highlighted in red, those <0.5 were highlighted in blue. Also the coefficient of variance (CV) between the four replicates for each condition was determined.

Uniprot identifier	gene name	description	protein coverage [%]	MW [Da]	pI	number of peptides	category according to Figure 2	geometric mean median normalized intensity WT	geometric mean median normalized intensity ΔcpxRA ON	CV [%] normalized intensity WT	[CV%] median normalized intensity ΔcpxRA ON	ratio ΔcpxRA ON /WT	ΔcpxRA/WT ON p-value	ΔcpxRA ON/WT q-value (BH)
ACCA_ECODH	*accA*	Acetyl-coenzyme A carboxylase carboxyl transferase su	40,752	35241,539	5,762	9		3006460	2788495	7,3	7,1	0,93	0,19	0,25
B1XHM4_ECODH	*accB*	Acetyl CoA carboxylase, BCCP subunit	23,718	16687,211	4,658	3		3198997	3417842	14,9	18,5	1,07	0,58	0,65
B1XHM5_ECODH	*accC*	Acetyl-CoA carboxylase, biotin carboxylase subunit	37,194	49320,738	6,655	14		7467437	6319846	7,1	6,8	0,85	0,01	0,03
ACCD_ECODH	*accD*	Acetyl-coenzyme A carboxylase carboxyl transferase su	20,066	33321,891	7,578	5		2347406	1822499	12,1	9,1	0,78	0,02	0,03
B1XC14_ECODH	*aceA*	Isocitrate lyase	62,903	47521,566	5,161	17	metabolism	29467616	4381955	8,4	4,2	0,15	0,00	0,00
B1XC13_ECODH	*aceB*	Malate synthase	37,899	60273,574	5,386	14		5541986	1061782	9,1	14,1	0,19	0,00	0,00
B1XC89_ECODH	*aceE*	Pyruvate dehydrogenase E1 component	50,169	99668,484	5,457	33		83823832	65928743	4,4	3,9	0,79	0,00	0,00
B1XC90_ECODH	*aceF*	Pyruvate dehydrogenase, dihydrolipoyltransacetylase cc	55,873	66096,07	5,09	22		43025454	36100760	6,2	7,5	0,84	0,01	0,02
B1X907_ECODH	*ackA*	Acetate kinase	53,25	43290,453	5,846	12		16194575	15444676	8,8	8,4	0,95	0,48	0,55
B1XBM7_ECODH	*acnA*	Aconitate hydratase 1	32,66	97647,195	5,593	18		3515428	1424154	2,5	9,2	0,41	0,00	0,00
B1XC93_ECODH	*acnB*	Aconitate hydratase 2	46,59	93498,109	5,239	28		21519467	15688478	5,9	3,4	0,73	0,00	0,00
ACP_ECODH	*acpP*	Acyl carrier protein	32,051	8639,52	3,976	2		14209819	19976443	17,6	17,2	1,41	0,03	0,05
B1XFQ0_ECODH	*acrA*	Multidrug efflux system	35,264	42196,707	7,69	9		2290160	2634814	7,8	7,0	1,15	0,04	0,06
B1XFP9_ECODH	*acrB*	Multidrug efflux system protein	24,023	113573,65	5,387	17		3259625	3375431	13,6	2,1	1,04	0,62	0,68
B1XCV5_ECODH	*acs*	Acetyl-coenzyme A synthetase	27,454	72093,586	5,501	12		1177069	651951	3,4	5,4	0,55	0,00	0,00
ADD_ECODH	*add*	Adenosine deaminase	46,246	36397,461	5,363	10		2001165	4195298	4,3	3,3	2,10	0,00	0,00
ADEC_ECODH	*ade*	Adenine deaminase	7,993	63739,359	5,234	3		218890	162929	12,4	8,5	0,74	0,01	0,01
B1XAT1_ECODH	*adhE*	Fused acetaldehyde-CoA dehydrogenase and iron-depe	45,342	96127,242	6,324	29		24277271	29204912	4,9	3,8	1,20	0,00	0,00
KAD_ECODH	*adk*	Adenylate kinase	39,252	23586,016	5,551	7		8848699	8034689	11,3	5,9	0,91	0,17	0,22
B1X9C3_ECODH	*agp*	Glucose-1-phosphatase/inositol phosphatase	23,729	45682,91	5,484	6		1662520	592413	9,6	3,5	0,36	0,00	0,00
B1X603_ECODH	*ahpC*	Alkyl hydroperoxide reductase, C22 subunit	52,941	20761,443	5,027	7		11698272	31710416	34,3	12,3	2,71	0,00	0,00
B1X604_ECODH	*ahpF*	Alkyl hydroperoxide reductase, F52a subunit, FAD/NAD	30,518	56177,109	5,469	12		3126874	3829888	3,9	7,8	1,22	0,00	0,01
SYA_ECODH	*alaS*	Alanine--tRNA ligase	43,95	96032,398	5,533	29		11421869	16081962	6,4	4,3	1,41	0,00	0,00
B1XDC8_ECODH	*aldA*	Aldehyde dehydrogenase A, NAD-linked	48,434	52272,789	5,067	16		9433873	2242640	6,4	8,0	0,24	0,00	0,00
B1X8K4_ECODH	*aldB*	Aldehyde dehydrogenase B	15,039	56306,352	5,439	5		468445	108556	8,4	15,3	0,23	0,00	0,00
B1XGA4_ECODH	*allR*	DNA-binding transcriptional repressor	15,498	29269,666	5,773	3		154760	158234	23,7	7,0	1,02	0,87	0,89
B1XAA6_ECODH	*amiA*	N-acetylmuramoyl-l-alanine amidase I	11,419	31412,129	10,098	2	peptidoglycan-modification	68826	56975	9,2	6,3	0,83	0,01	0,03
B1X6C7_ECODH	*amn*	AMP nucleosidase	9,711	53994,914	5,897	3		217383	308500	18,9	10,7	1,42	0,02	0,03
ANMK_ECODH	*anmK*	Anhydro-N-acetylmuramic acid kinase	8,401	39496,277	5,699	2		102438	97220	7,5	0,3	0,95	0,21	0,27
B1XFB7_ECODH	*ansB*	Periplasmic L-asparaginase II	32,759	36850,691	5,956	8		4701317	336532	7,5	7,8	0,07	0,00	0,00
APT_ECODH	*apt*	Adenine phosphoribosyltransferase	43,169	19858,885	5,258	5		2344898	1817757	4,9	3,1	0,78	0,00	0,00
B1XFL1_ECODH	*arcA*	DNA-binding response regulator in two-component regu	30,672	27292,016	5,212	5		1831233	1872511	14,6	8,4	1,02	0,80	0,84
B1XHI5_ECODH	*arcB*	Hybrid sensory histidine kinase in two-component regul	3,985	87982,906	4,986	2		89952	88338	9,4	8,4	0,98	0,78	0,82
ARGE_ECODH	*argE*	Acetylornithine deacetylase	15,927	42347,344	5,538	4		192832	427305	2,2	10,7	2,22	0,00	0,00
ASSY_ECODH	*argG*	Argininosuccinate synthase	15,884	49898,398	5,23	4		231952	337280	10,0	8,0	1,45	0,00	0,00
ARLY_ECODH	*argH*	Argininosuccinate lyase	8,753	50318,223	5,108	2		68060	141698	14,0	4,3	2,08	0,00	0,00
ARGR_ECODH	*argR*	Arginine repressor	13,462	16994,521	4,928	1		179581	170162	23,2	10,7	0,95	0,67	0,72
SYR_ECODH	*argS*	Arginine--tRNA ligase	34,662	64682,961	5,321	16		4001860	3304129	6,0	6,3	0,83	0,00	0,01
AROA_ECODH	*aroA*	3-phosphoshikimate 1-carboxyvinyltransferase	24,122	46095,781	5,366	8		439700	799671	6,3	6,2	1,82	0,00	0,00
AROB_ECODH	*aroB*	3-dehydroquinate synthase	25,967	38880,988	5,721	6		560425	541175	6,7	14,5	0,97	0,69	0,74
AROC_ECODH	*aroC*	Chorismate synthase	5,54	39137,445	5,81	2		62898	61814	17,7	24,3	0,98	0,92	0,93
AROD_ECODH	*aroD*	3-dehydroquinate dehydratase	14,683	27466,654	5,19	3		77831	144038	12,8	9,4	1,85	0,00	0,00
AROE_ECODH	*aroE*	Shikimate dehydrogenase	18,382	29413,639	5,349	3		97079	133720	18,6	2,2	1,38	0,01	0,02
B1XBS3_ECODH	*aroF*	Phospho-2-dehydro-3-deoxyheptonate aldolase	11,798	38803,961	5,423	2		95023	129334	12,2	16,7	1,36	0,02	0,04
B1X785_ECODH	*aroG*	Phospho-2-dehydro-3-deoxyheptonate aldolase	30	38009,523	6,138	7		1208585	2409460	4,0	4,2	1,99	0,00	0,00
B1XG11_ECODH	*aroH*	Phospho-2-dehydro-3-deoxyheptonate aldolase	9,77	38735,055	6,423	3		104772	126376	21,5	17,4	1,21	0,23	0,29
AROK_ECODH	*aroK*	Shikimate kinase 1	21,965	19538,047	5,263	3		1327601	1294491	18,5	3,8	0,98	0,79	0,83
B1X804_ECODH	*artI*	Arginine transporter subunit periplasmic-binding compo	32,922	26929,557	5,792	5		613296	736831	13,4	30,0	1,20	0,38	0,45
B1X778_ECODH	*asd*	Aspartate-semialdehyde dehydrogenase	35,15	40017,883	5,369	8		3218349	4914278	4,2	4,2	1,53	0,00	0,00
ASNA_ECODH	*asnA*	Aspartate--ammonia ligase	19,091	36650,555	5,453	5		421725	349626	12,1	7,3	0,83	0,04	0,06
B1X6K7_ECODH	*asnB*	Asparagine synthetase	18,953	62659,012	5,55	6		572270	818896	10,1	2,2	1,43	0,00	0,00
B1X8N4_ECODH	*asnS*	Asparagine--tRNA ligase	43,991	52570,441	5,172	17		11837356	11583060	4,2	3,7	0,98	0,47	0,54
B1XD19_ECODH	*aspA*	Aspartate ammonia-lyase	37,657	52356,129	5,191	13		17083751	2177888	6,4	9,2	0,13	0,00	0,00
B1X8N2_ECODH	*aspC*	Aspartate aminotransferase, PLP-dependent	55,051	43573,359	5,544	17		6576795	9497682	4,6	4,4	1,44	0,00	0,00
SYD_ECODH	*aspS*	Aspartate--tRNA ligase	44,915	65913,445	5,472	19		10042086	6015788	9,3	3,0	0,60	0,00	0,00
ASTC_ECODH	*astC*	Succinylornithine transaminase	11,084	43665,375	5,913	2		188320	27024	10,8	44,5	0,14	0,00	0,00
ATPA_ECODH	*atpA*	ATP synthase subunit alpha	50,097	55222,078	5,796	17		27712376	27516430	4,4	1,5	0,99	0,77	0,82
ATPB_ECODH	*atpD*	ATP synthase subunit beta	73,913	50325,422	4,902	21		50264832	44365306	5,7	1,0	0,88	0,00	0,01
ATPF_ECODH	*atpF*	ATP synthase subunit b	46,795	17263,957	5,991	6		2658358	3251644	11,6	8,9	1,22	0,03	0,06
ATPG_ECODH	*atpG*	ATP synthase gamma chain	41,115	31577,42	8,839	9		4866229	5194537	4,6	7,3	1,07	0,19	0,24

ATPD_ECODH	*atpH*	ATP synthase subunit delta	52,542	19332,225	4,945	7		3502126	2572059	9,8	7,3	0,73	0,00	0,01
B1X8I8_ECODH	*avtA*	Valine-pyruvate aminotransferase	15,588	46711,227	5,65	6		231258	329336	12,4	10,7	1,42	0,01	0,01
B1XDC5_ECODH	*azoR*	FMN-dependent NADH-azoreductase	24,378	21657,717	5,063	3		400931	440042	10,7	8,7	1,10	0,23	0,29
B1X7H5_ECODH	*baeR*	DNA-binding response regulator in two-component regu	47,083	27655,934	5,522	7		282842	363524	8,2	7,9	1,29	0,00	0,01
BAMA_ECODH	*bamA*	Outer membrane protein assembly factor BamA	42,469	90552,766	4,93	26		8280189	10569760	5,1	5,9	1,28	0,00	0,00
B1XAF2_ECODH	*bcp*	Thiol peroxidase, thioredoxin-dependent	64,744	17633,936	5,032	7		4321502	7313026	4,5	5,6	1,69	0,00	0,00
BETB_ECODH	*betB*	NAD/NADP-dependent betaine aldehyde dehydrogenas	11,633	52911,133	5,19	2		197948	34122	18,6	17,0	0,17	0,00	0,00
B1X6I7_ECODH	*bfr*	Bacterioferritin	60,759	18495,027	4,69	7		2682373	3519666	4,9	8,9	1,31	0,00	0,01
B1XEI8_ECODH	*bglA*	6-phospho-beta-glucosidase A	34,447	55360,766	5,577	14		2614121	2862938	3,6	6,4	1,10	0,05	0,07
B1X7M1_ECODH	*bglX*	Beta-D-glucoside glucohydrolase, periplasmic	22,876	83460,047	5,854	12		779213	877513	8,8	2,7	1,13	0,04	0,06
B1XAM7_ECODH	*bipA*	GTP-binding protein	26,359	67355,453	5,16	12		6358882	7194414	7,6	5,2	1,13	0,04	0,06
B1XDQ4_ECODH	*blc*	Outer membrane lipoprotein (Lipocalin)	10,169	19851,607	8,884	2		25506	71959	9,9	15,3	2,82	0,00	0,00
B1XFM3_ECODH	*bolA*	Regulator of penicillin binding proteins and beta lactama	15,238	11993,675	6,187	1		260716	271161	21,1	16,4	1,04	0,80	0,84
B1XBX7_ECODH	*btuB*	Vitamin B12 transporter BtuB	19,055	68407,148	5,226	8		935209	926063	6,3	7,7	0,99	0,85	0,88
B1XG17_ECODH	*btuE*	Glutathione peroxidase	47,541	20469,557	4,812	6		739373	1327130	11,7	6,6	1,79	0,00	0,00
B1XD12_ECODH	*cadA*	Lysine decarboxylase 1	20,28	81260,125	5,914	10	acid stress response	723120	2709486	21,6	6,2	3,75	0,00	0,00
B1XCA1_ECODH	*can*	Carbonic anhydrase	21,364	25096,768	6,164	4		1849930	2413110	12,7	10,5	1,30	0,02	0,03
B1XBF8_ECODH	*carB*	Carbamoyl-phosphate synthase large chain	14,632	117841,72	5,221	11		1163861	651423	44,1	17,2	0,56	0,04	0,06
CBPA_ECODH	*cbpA*	Curved DNA-binding protein	11,765	34455,008	6,327	2		568691	618053	6,8	17,1	1,09	0,38	0,45
CDD_ECODH	*cdd*	Cytidine deaminase	55,782	31539,869	5,419	9		3010913	2070359	5,8	4,9	0,69	0,00	0,00
B1XFX1_ECODH	*cfa*	Cyclopropane fatty acyl phospholipid synthase (Unsatur	9,686	43909,004	5,694	3		164741	615380	19,3	18,7	3,74	0,00	0,00
B1X7P3_ECODH	*cirA*	Ferric iron-catecholate outer membrane transporter	13,876	73895,727	5,106	5	transport	69349	77233	23,5	21,6	1,11	0,52	0,58
B1X6W4_ECODH	*cld*	Regulator of length of O-antigen component of lipopolys	31,595	36454,746	5,427	6		1197243	1159442	13,3	7,3	0,97	0,69	0,74
B1X823_ECODH	*clpA*	ATPase and specificity subunit of ClpA-ClpP ATP-depe	22,296	84206,93	5,911	13		1740778	2505109	6,2	7,3	1,44	0,00	0,00
B1XBR5_ECODH	*clpB*	Protein disaggregation chaperone	47,841	95585,023	5,367	30		18857061	32949521	3,1	9,3	1,75	0,00	0,00
B1XFM5_ECODH	*clpP*	ATP-dependent Clp protease proteolytic subunit	25,121	23186,648	5,523	4		3108536	3795506	15,9	10,2	1,22	0,08	0,11
CLPS_ECODH	*clpS*	ATP-dependent Clp protease adapter protein ClpS	32,075	12179,059	4,936	2		36012	55264	12,5	24,6	1,53	0,02	0,04
CLPX_ECODH	*clpX*	ATP-dependent Clp protease ATP-binding subunit ClpX	47,642	46356,008	5,236	12		4844448	6216491	3,0	2,7	1,28	0,00	0,00
KCY_ECODH	*cmk*	Cytidylate kinase	26,432	24746,342	5,559	4		421511	388266	10,6	6,4	0,92	0,22	0,29
CMOA_ECODH	*cmoA*	tRNA (cmo5U34)-methyltransferase	37,652	27776,645	5,357	6		741466	602531	4,3	5,3	0,81	0,00	0,00
COAA_ECODH	*coaA*	Pantothenate kinase	12,025	36359,777	6,325	2		142844	125205	9,4	11,3	0,88	0,13	0,17
B1XA30_ECODH	*cobB*	NAD-dependent protein deacylase	17,204	31464,039	7,788	3		221146	355971	12,9	20,3	1,61	0,01	0,01
B1XAI1_ECODH	*corA*	Magnesium/nickel/cobalt transporter	39,873	36589,711	4,636	8		995570	1251641	6,6	8,4	1,26	0,01	0,01
B1X653_ECODH	*corC*	Predicted ion transport	6,507	33298,488	4,55	2		178215	182063	14,4	23,1	1,02	0,89	0,91
B1XG37_ECODH	*cpdA*	3',5'-cyclic adenosine monophosphate phosphodiestera	13,818	30937,096	5,543	3		145325	92458	17,8	12,8	0,64	0,01	0,01
B1XDW3_ECODH	*cpdB*	2':3'-cyclic-nucleotide 2'-phosphodiesterase	27,202	70832,195	5,45	9		1337862	805151	9,6	6,4	0,60	0,00	0,00
B1XB80_ECODH	*cpxP*	Periplasmic protein combats stress	27,711	18965,246	6,379	3	stress response	65948	23766	8,5	44,8	0,36	0,00	0,01
B1XB79_ECODH	*cpxR*	DNA-binding response regulator in two-component regu	25,431	26312,113	5,393	5	stress response	383597	78287	30,9	19,2	0,20	0,00	0,00
B1XFK7_ECODH	*creA*	Protein CreA	29,936	17107,891	9,037	3		193212	225168	9,2	18,1	1,17	0,18	0,24
CRL_ECODH	*crl*	Sigma factor-binding protein Crl	22,556	15654,994	6,316	3		567209	703645	9,1	4,0	1,24	0,00	0,01
B1X705_ECODH	*crp*	DNA-binding transcriptional dual regulator	42,381	23640,426	8,376	8		5370359	5977108	9,4	6,0	1,11	0,10	0,14
B1XA88_ECODH	*crr*	Glucose-specific enzyme IIA component of PTS	58,58	18251,068	4,734	6		16009474	20063238	8,1	3,5	1,25	0,00	0,01
B1X8H1_ECODH	*cspA*	Major cold shock protein	31,429	7403,278	5,578	2		1432668	1327205	12,5	6,4	0,93	0,31	0,38
B1XH90_ECODH	*cspC*	Stress protein, member of the CspA-family	63,768	7402,368	6,539	3		4674281	3293325	7,9	4,6	0,70	0,00	0,00
B1X821_ECODH	*cspD*	Cold shock protein-like protein	55,405	7968,975	5,808	2		1596597	911969	23,8	6,9	0,57	0,00	0,01
B1X620_ECODH	*cspE*	DNA-binding transcriptional repressor	53,623	7463,451	8,087	3		11926045	14385323	8,1	9,4	1,21	0,02	0,04
B1X5Z5_ECODH	*cstA*	Carbon starvation protein	6,134	75105,234	8,01	3		212933	26984	14,1	29,4	0,13	0,00	0,00
B1XC98_ECODH	*cueO*	Multicopper oxidase (Laccase)	12,791	56556,152	6,28	3		83284	163010	19,8	17,8	1,96	0,00	0,01
CUTA_ECODH	*cutA*	Divalent-cation tolerance protein CutA	22,321	12331,083	4,852	1		35065	108113	26,3	9,9	3,08	0,00	0,00
CYAY_ECODH	*cyaY*	Protein CyaY	41,509	12231,413	4,241	3		40103	106426	33,7	31,6	2,65	0,01	0,01
B1XDV8_ECODH	*cycA*	D-alanine/D-serine/glycine transporter	5,745	51659,656	8,918	2		75310	72457	9,4	14,0	0,96	0,66	0,72
B1X6R3_ECODH	*cydA*	Cytochrome d terminal oxidase, subunit I	17,816	58205,082	6,353	8	acid stress response	7613775	11641826	4,8	2,2	1,53	0,00	0,00
B1X6R4_ECODH	*cydB*	Cytochrome d terminal oxidase, subunit II	5,805	42453,117	6,901	2	acid stress response	1673172	3415309	6,0	2,8	2,04	0,00	0,00
B1X826_ECODH	*cydC*	Fused cysteine transporter subunits of ABC superfamily	11,344	62920,168	8,754	4		399104	427129	8,6	7,5	1,07	0,28	0,35
B1X827_ECODH	*cydD*	Fused cysteine transporter subunits of ABC superfamily	12,925	65056,191	5,997	4		295663	256605	6,1	19,7	0,87	0,22	0,28
B1XFM0_ECODH	*cyoA*	Cytochrome o ubiquinol oxidase subunit II	33,333	34911,297	6,758	6		7281483	3445243	14,6	10,4	0,47	0,00	0,00
B1XFL9_ECODH	*cyoB*	Cytochrome o ubiquinol oxidase subunit I	7,994	74367,906	6,698	5		1797954	1468448	15,8	7,6	0,82	0,06	0,09
B1XBM4_ECODH	*cysB*	DNA-binding transcriptional dual regulator, O-acetyl-L-s	14,815	36150,469	6,873	4		182232	237793	10,6	7,7	1,30	0,01	0,01
B1X941_ECODH	*cysE*	Serine acetyltransferase	20,513	29316,639	6,05	3		265597	348170	19,5	7,2	1,31	0,05	0,08
B1XA85_ECODH	*cysK*	Cysteine synthase	45,82	34489,652	5,829	11		5522376	12427360	4,9	3,0	2,25	0,00	0,00
SYC_ECODH	*cysS*	Cysteine--tRNA ligase	35,792	52202,086	5,326	13		1917801	2482980	19,2	4,7	1,29	0,04	0,07
B1XBA0_ECODH	*cytR*	DNA-binding transcriptional dual regulator	11,437	37819,781	6,015	2		64038	47139	12,4	15,2	0,74	0,02	0,04
B1X627_ECODH	*dacA*	D-alanyl-D-alanine carboxypeptidase (Penicillin-binding	28,288	44443,957	8,31	9		1799978	1732522	10,4	3,0	0,96	0,50	0,57
B1X7Y0_ECODH	*dacC*	D-alanyl-D-alanine carboxypeptidase (Penicillin-binding	43,25	43608,906	7,738	12	peptidoglycan-modification	1663586	2188388	20,2	10,6	1,32	0,05	0,07
DADA_ECODH	*dadA*	D-amino acid dehydrogenase small subunit	40,278	47607,293	6,174	12		2723001	1703050	5,7	6,0	0,63	0,00	0,00
B1X735_ECODH	*damX*	Uncharacterized protein	10,748	46161,91	5,687	3		694871	1046325	13,9	9,1	1,51	0,00	0,01
B1XAF0_ECODH	*dapA*	4-hydroxy-tetrahydrodipicolinate synthase	34,247	31269,971	5,981	7		2699024	3085250	3,9	8,7	1,14	0,03	0,06
DAPB_ECODH	*dapB*	4-hydroxy-tetrahydrodipicolinate reductase	11,355	28756,613	5,446	3		356577	475479	9,4	13,8	1,33	0,01	0,03
DAPD_ECODH	*dapD*	2,3,4,5-tetrahydropyridine-2,6-dicarboxylate N-succinyltı	34,672	29892,098	5,563	8		11244404	12132374	7,3	1,8	1,08	0,09	0,13
DAPE_ECODH	*dapE*	Succinyl-diaminopimelate desuccinylase	21,867	41269,012	5,307	6		364110	338002	9,5	4,5	0,93	0,21	0,27
DAPF_ECODH	*dapF*	Diaminopimelate epimerase	8,029	30208,562	5,856	2		258757	209729	31,7	9,3	0,81	0,30	0,36
B1XEC5_ECODH	*dcp*	Dipeptidyl carboxypeptidase II	26,872	77515,75	5,488	12		1449804	2007759	6,4	5,2	1,38	0,00	0,00
B1X7T0_ECODH	*dcrB*	Periplasmic protein	23,784	19787,492	5,085	3		2705675	2877370	11,0	5,7	1,06	0,36	0,43
B1XD18_ECODH	*dcuA*	Anaerobic C4-dicarboxylate transporter	3,926	45750,707	7,629	2		413352	217459	3,2	16,9	0,53	0,00	0,00

B1XEX0_ECODH	*ddlA*	D-alanine--D-alanine ligase	35,714	39315,809	5,02	9		2049956	2007033	4,3	5,5	0,98	0,57	0,63
B1XC69_ECODH	*ddlB*	D-alanine--D-alanine ligase	17,974	32839,707	4,772	4		780643	707444	11,7	5,2	0,91	0,18	0,24
B1XGX4_ECODH	*deaD*	ATP-dependent RNA helicase DeaD	29,253	70546,156	8,746	14		4234079	2662792	7,2	12,2	0,63	0,00	0,00
DEF_ECODH	*def*	Peptide deformylase	15,976	19328,422	5,229	2		416736	574304	18,5	11,2	1,38	0,04	0,06
B1XD31_ECODH	*degP*	Serine endoprotease (Protease Do), membrane-associ	41,772	49354,23	8,648	13	degradation/folding	5493300	6243062	7,4	7,1	1,14	0,04	0,07
B1XHK7_ECODH	*degQ*	Serine endoprotease, periplasmic	18,022	47205,055	5,763	4		371799	465852	8,2	6,9	1,25	0,01	0,01
TYPH_ECODH	*deoA*	Thymidine phosphorylase	62,727	47207,121	5,214	19		3638898	41647827	6,8	1,6	11,45	0,00	0,00
DEOB_ECODH	*deoB*	Phosphopentomutase	60,934	44369,961	5,106	17		8957027	50186877	4,2	4,9	5,60	0,00	0,00
DEOC_ECODH	*deoC*	Deoxyribose-phosphate aldolase	61,004	27733,801	5,496	12		6588487	75783823	3,5	6,1	11,50	0,00	0,00
DEOD_ECODH	*deoD*	Purine nucleoside phosphorylase DeoD-type	64,435	25949,91	5,418	11		10616246	45563557	5,8	9,6	4,29	0,00	0,00
DER_ECODH	*der*	GTPase Der	21,837	55035,875	5,596	8		1452154	1231244	4,4	2,7	0,85	0,00	0,00
B1X973_ECODH	*dfp*	Fused 4'-phosphopantothenoylcysteine decarboxylase p	22,414	43438,141	7,056	5		321417	285786	20,0	6,5	0,89	0,34	0,41
B1XAP1_ECODH	*dhaK*	Dihydroxyacetone kinase, N-terminal domain	16,854	38215,207	4,815	3		1678691	913071	7,0	12,3	0,54	0,00	0,00
B1XAP0_ECODH	*dhaL*	Dihydroxyacetone kinase, C-terminal domain	58,571	22631,746	5,309	7		1185980	845900	15,4	7,5	0,71	0,01	0,02
B1XAN9_ECODH	*dhaM*	Fused predicted dihydroxyacetone-specific PTS enzyme	31,78	51448,621	4,612	9		1628995	1675337	7,3	4,0	1,03	0,52	0,59
B1XC56_ECODH	*djlA*	DnaJ-like protein DjlA	8,118	30579,363	9,744	2		117821	94463	2,5	5,8	0,80	0,00	0,00
B1XFG4_ECODH	*dkgA*	2,5-diketo-D-gluconate reductase A	22,182	31109,637	5,999	5		739654	1066753	15,5	3,5	1,44	0,00	0,01
B1XD71_ECODH	*dkgB*	2,5-diketo-D-gluconate reductase B	8,989	29436,791	5,492	2		84454	104486	30,9	8,5	1,24	0,29	0,35
B1XCC0_ECODH	*dksA*	RNA polymerase-binding transcription factor DksA	23,841	17527,752	5,063	3		4180235	3803821	13,9	8,1	0,91	0,29	0,36
B1X7M2_ECODH	*dld*	D-lactate dehydrogenase	24,343	64612,332	6,19	11		755846	1029547	3,5	5,0	1,36	0,00	0,00
B1X834_ECODH	*dmsA*	Dimethyl sulfoxide reductase, anaerobic, subunit A	9,459	90398,617	6,42	6		579529	113195	8,9	13,4	0,20	0,00	0,00
B1XCU0_ECODH	*dnaB*	Replicative DNA helicase	10,828	52390,082	4,946	3		310124	234764	5,9	9,4	0,76	0,00	0,01
B1XD53_ECODH	*dnaE*	DNA polymerase III alpha subunit	1,897	129904,57	5,165	2		33528	20362	11,3	14,1	0,61	0,00	0,00
B1XG71_ECODH	*dnaG*	DNA primase	6,885	65564,625	5,685	2		26015	42268	53,7	20,6	1,62	0,18	0,24
DNAJ_ECODH	*dnaJ*	Chaperone protein DnaJ	23,404	41100,332	7,977	7		1431518	5654698	12,8	12,5	3,95	0,00	0,00
B1XBD9_ECODH	*dnaK*	Chaperone protein DnaK	54,859	69114,961	4,831	23		51520712	97001347	3,7	7,0	1,88	0,00	0,00
B1X9T0_ECODH	*dnaN*	DNA polymerase III subunit beta	17,213	40586,602	5,25	4		749729	760336	6,2	10,5	1,01	0,82	0,86
DNAT_ECODH	*dnaT*	Primosomal protein 1	12,849	19455,121	5,145	1		39686	26356	18,1	9,0	0,66	0,01	0,02
B1X8G0_ECODH	*dppA*	Dipeptide transporter periplasmic-binding component of	14,766	60293,672	6,211	4		638159	79330	19,9	18,7	0,12	0,00	0,00
DPS_ECODH	*dps*	DNA protection during starvation protein	66,467	18695,307	5,723	9		16441117	8036065	7,6	9,6	0,49	0,00	0,00
B1XAL7_ECODH	*dsbA*	Thiol:disulfide interchange protein	24,519	23104,537	5,946	3	degradation/folding	611814	757882	11,1	5,4	1,24	0,01	0,02
B1XEI0_ECODH	*dsbC*	Protein disulfide isomerase II	17,797	25621,656	6,296	4		140652	214528	17,6	30,6	1,53	0,05	0,07
B1X602_ECODH	*dsbG*	Periplasmic disulfide isomerase/thiol-disulphide oxidase	12,5	27494,99	8,318	2		138660	130363	4,9	10,9	0,94	0,35	0,42
SDHD_ECODH	*dsdA*	D-serine dehydratase	42,986	47900,57	5,584	11		5072805	2684927	7,6	3,2	0,53	0,00	0,00
B1XCT7_ECODH	*dusA*	tRNA-dihydrouridine synthase	22,899	38467,898	6,116	4		336998	432658	12,0	4,6	1,28	0,01	0,02
DUT_ECODH	*dut*	Deoxyuridine 5'-triphosphate nucleotidohydrolase	32,45	16155,47	5,031	2		965588	1583716	18,1	19,4	1,64	0,01	0,02
B1XD42_ECODH	*dxr*	1-deoxy-D-xylulose 5-phosphate reductoisomerase	5,779	43387,977	5,675	2		45176	59374	21,1	17,5	1,31	0,10	0,14
DXS_ECODH	*dxs*	1-deoxy-D-xylulose-5-phosphate synthase	16,452	67616,938	6,164	8		846079	848148	8,2	7,4	1,00	0,97	0,97
NQOR_ECODH	*CDH10B_107*	NAD(P)H dehydrogenase (quinone)	51,01	20845,555	5,594	7		4789765	6506752	7,0	6,1	1,36	0,00	0,00
CINAL_ECODH	*CDH10B_240*	CinA-like protein	20,5	44225,645	5,463	5		255474	512315	14,6	9,2	2,01	0,00	0,00
B1XDQ2_ECODH	*ecnB*	Entericidin B membrane lipoprotein	39,583	4809,528	7,934	1		1924480	1472183	12,3	11,9	0,76	0,02	0,04
ECOT_ECODH	*eco*	Ecotin	43,827	18192,049	6,602	5		1144393	1415184	22,1	3,2	1,24	0,10	0,15
B1XHB8_ECODH	*eda*	Multifunctional 2-keto-3-deoxygluconate 6-phosphate al	34,272	22284,027	5,569	5		3666416	4886971	12,4	11,1	1,33	0,02	0,03
EFP_ECODH	*efp*	Elongation factor P	17,021	20591,314	4,897	2		2652391	2261574	15,4	7,0	0,85	0,09	0,14
B1X8Y0_ECODH	*elaB*	Conserved protein	27,723	11305,689	5,35	2		727051	2448930	60,3	6,7	3,37	0,00	0,01
B1XHI4_ECODH	*elbB*	Isoprenoid biosynthesis protein with amidotransferase-li	23,041	22981,551	4,675	3		1498034	1576340	6,9	4,7	1,05	0,26	0,32
B1XCL8_ECODH	*emrA*	Multidrug efflux system	8,205	42736,047	9,333	2		225074	166669	13,0	11,5	0,74	0,01	0,03
EMTA_ECODH	*emtA*	Endo-type membrane-bound lytic murein transglycosyla	35,961	22226,547	9,161	4		313275	371268	6,0	10,8	1,19	0,03	0,06
ENO_ECODH	*eno*	Enolase	52,546	45654,945	5,324	14		57757965	53464834	4,5	2,4	0,93	0,02	0,04
B1X5Z2_ECODH	*entB*	Isochorismatase	8,772	32554,338	5,046	2	metabolism	121152	90320	13,2	11,9	0,75	0,02	0,03
B1X5Z1_ECODH	*entE*	Enterobactin synthetase component E	11,754	59112,152	5,419	3	metabolism	40207	41580	14,3	12,8	1,03	0,74	0,78
E4PD_ECODH	*epd*	D-erythrose-4-phosphate dehydrogenase	14,749	37299,391	6,264	5		230172	455575	4,0	4,9	1,98	0,00	0,00
ERA_ECODH	*era*	GTPase Era	19,934	33810,066	6,73	5		565770	496957	2,8	7,9	0,88	0,02	0,04
ERPA_ECODH	*erpA*	Iron-sulfur cluster insertion protein ErpA	18,421	12100,484	4,109	2		805230	1583900	17,1	11,6	1,97	0,00	0,00
B1XAB2_ECODH	*eutB*	Ethanolamine ammonia-lyase, large subunit, heavy cha	8,168	49403,047	4,789	3		114446	131464	27,6	10,0	1,15	0,37	0,44
B1XFF9_ECODH	*exbB*	Membrane spanning protein in TonB-ExbB-ExbD compl	16,393	26287,387	7,888	3		699985	423323	3,1	13,4	0,60	0,00	0,00
B1XFF8_ECODH	*exbD*	Membrane spanning protein in TonB-ExbB-ExbD compl	13,475	15526,983	4,698	2		50998	54193	23,0	20,9	1,06	0,70	0,75
B1XG98_ECODH	*exuR*	DNA-binding transcriptional repressor	13,953	29835,695	5,395	3		513301	394829	7,3	8,1	0,77	0,00	0,01
FABA_ECODH	*fabA*	3-hydroxydecanoyl-[acyl-carrier-protein] dehydratase	19,186	18969,049	6,126	3		4282581	5654068	9,6	4,9	1,32	0,00	0,01
B1X934_ECODH	*fabB*	3-oxoacyl-[acyl-carrier-protein] synthase I	39,655	42613,32	5,347	10		6604220	19135530	9,1	7,1	2,90	0,00	0,00
B1XA02_ECODH	*fabD*	Malonyl CoA-acyl carrier protein transacylase	45,307	32417,203	4,947	7		10883343	9622731	4,9	7,1	0,88	0,03	0,05
B1XA05_ECODH	*fabF*	3-oxoacyl-[acyl-carrier-protein] synthase 2	33,414	43045,766	5,714	8		3699537	4692370	12,6	5,2	1,27	0,01	0,03
B1XA03_ECODH	*fabG*	3-oxoacyl-[acyl-carrier-protein] reductase	54,508	25560,295	6,763	9		7000366	5235982	11,8	10,8	0,75	0,01	0,03
B1XA01_ECODH	*fabH*	3-oxoacyl-[acyl-carrier-protein] synthase 3	38,801	33515,121	5,078	8		4216170	3812326	4,6	5,2	0,90	0,03	0,05
B1XBN9_ECODH	*fabI*	Enoyl-[acyl-carrier-protein] reductase [NADH]	50,382	27863,939	5,577	9		18240176	17832271	5,5	2,3	0,98	0,49	0,56
FABZ_ECODH	*fabZ*	3-hydroxyacyl-[acyl-carrier-protein] dehydratase FabZ	28,477	17032,953	6,84	4		2510322	2157956	7,1	6,7	0,86	0,02	0,04
FADB_ECODH	*fadB*	Fatty acid oxidation complex subunit alpha	7,682	79593,914	5,838	4	metabolism	116228	44613	10,3	18,3	0,38	0,00	0,00
B1XD83_ECODH	*fadE*	Acyl coenzyme A dehydrogenase	3,931	89224,445	8,221	2		60777	8472	12,8	16,0	0,14	0,00	0,00
B1X9L7_ECODH	*fadL*	Long-chain fatty acid outer membrane transporter	25,223	48771,914	5,086	7		3080747	1400329	7,3	2,2	0,45	0,00	0,00
FADR_ECODH	*fadR*	Fatty acid metabolism regulator protein	30,544	26968,609	6,507	5		315983	216014	16,4	15,6	0,68	0,02	0,04
B1XEK8_ECODH	*fbaA*	Fructose-bisphosphate aldolase, class II	32,869	39147,262	5,524	7		24756889	58316846	7,0	4,0	2,36	0,00	0,00
B1X7I8_ECODH	*fbaB*	Fructose-bisphosphate aldolase class I	43,714	38109,191	6,248	11		2042080	4187770	7,7	6,8	2,05	0,00	0,00
F16PA_ECODH	*fbp*	Fructose-1,6-bisphosphatase class 1	39,157	36833,934	5,665	9		2202928	1857927	4,8	6,1	0,84	0,01	0,01
FDHE_ECODH	*fdhE*	Protein FdhE	8,091	34746,711	5,033	2		269150	253123	9,6	12,4	0,94	0,47	0,54

B1XE65_ECODH	*fdnG*	Formate dehydrogenase-N, alpha subunit, nitrate-induc	33,3	112963,34	6,543	22		5395074	831749	7,1	8,1	0,15	0,00	0,00
B1XE66_ECODH	*fdnH*	Formate dehydrogenase-N, Fe-S (Beta) subunit, nitrate	14,286	32238,719	5,575	4		501819	139876	16,1	32,7	0,28	0,00	0,00
B1XB62_ECODH	*fdoG*	Formate dehydrogenase-O, large subunit	42,52	112536,41	6,883	25		8587833	6016914	7,5	2,3	0,70	0,00	0,00
B1XB61_ECODH	*fdoH*	Formate dehydrogenase-O, Fe-S subunit	20,667	33100,383	5,166	5		403116	845565	39,9	13,3	2,10	0,01	0,02
B1XB60_ECODH	*fdoI*	Formate dehydrogenase-O, cytochrome b556 subunit	9,005	24606,365	10,21	2		213173	291324	21,3	11,9	1,37	0,05	0,08
B1XB00_ECODH	*fdx*	[2Fe-2S] ferredoxin	55,856	12330,815	4,491	4		1298059	1748352	18,3	7,6	1,35	0,03	0,05
B1XER5_ECODH	*fecA*	KpLE2 phage-like element ferric citrate outer membrane	18,088	85321,609	5,59	10		1755417	1371619	12,0	11,4	0,78	0,02	0,04
B1XER4_ECODH	*fecB*	KpLE2 phage-like element iron-dicitrate transporter sub	9,333	33146,348	8,832	2		174724	82504	11,5	23,0	0,47	0,00	0,00
B1X755_ECODH	*feoA*	Ferrous iron transporter, protein A	21,333	8370,826	9,425	1		39783	40472	25,3	16,9	1,02	0,91	0,93
B1X756_ECODH	*feoB*	Fused ferrous iron transporter, protein B: GTP-binding p	5,304	84473,656	5,81	3		192191	168749	6,5	13,6	0,88	0,13	0,17
B1X5Y0_ECODH	*fepA*	Iron-enterobactin outer membrane transporter	13,137	82106,898	5,386	6	transport	135800	145225	12,6	21,3	1,07	0,62	0,68
B1XBT2_ECODH	*ffh*	Signal recognition particle protein	43,929	49787,305	9,517	14		3437472	3288578	3,9	4,1	0,96	0,17	0,23
B1XCC5_ECODH	*fhuA*	Ferrichrome outer membrane transporter	34,672	82114,953	5,465	15		1829618	2442525	2,4	6,1	1,33	0,00	0,00
FIS_ECODH	*fis*	DNA-binding protein Fis	32,653	11239,932	9,344	2		5978028	3638393	11,4	15,0	0,61	0,00	0,01
B1XDV7_ECODH	*fklB*	Peptidyl-prolyl cis-trans isomerase	43,204	22216,193	4,849	6		6020863	8308614	7,9	4,6	1,38	0,00	0,00
B1X6J6_ECODH	*fkpA*	Peptidyl-prolyl cis-trans isomerase	27,407	28881,926	8,386	6		3459592	3960199	17,7	10,7	1,14	0,23	0,29
B1XBF3_ECODH	*fkpB*	Peptidyl-prolyl cis-trans isomerase	47,651	16081,006	4,3	3		601267	509398	12,3	13,6	0,85	0,12	0,16
B1X6L7_ECODH	*fldA*	Flavodoxin	38,636	19736,941	4,213	4		783980	1989115	48,0	10,0	2,54	0,01	0,02
B1X678_ECODH	*fliY*	Cystine transporter subunit periplasmic-binding compon	12,406	29039,189	6,216	3		32216	92633	57,4	8,1	2,88	0,01	0,01
B1X6T5_ECODH	*flu*	CP4-44 prophage antigen 43 (Ag43) phase-variable bio	35,515	106825,45	5,777	21		26222767	2097377	11,9	15,1	0,08	0,00	0,00
FMT_ECODH	*fmt*	Methionyl-tRNA formyltransferase	46,032	34168,465	5,559	9		1820144	1692248	11,5	3,5	0,93	0,26	0,32
B1XCG3_ECODH	*fnr*	DNA-binding transcriptional dual regulator, global regula	17,6	27967,207	8,25	3		982941	106045	21,5	12,1	0,11	0,00	0,00
B1XC49_ECODH	*folA*	Dihydrofolate reductase	33,333	17999,377	4,839	3		198435	195033	13,1	10,4	0,98	0,84	0,87
B1XG63_ECODH	*folB*	Bifunctional dihydroneopterin aldolase and dihydroneop	13,934	13619,531	4,678	2		64434	59713	19,2	17,6	0,93	0,57	0,63
B1X926_ECODH	*folC*	Bifunctional protein FolC	19,194	45405,699	5,502	6		398435	374840	6,8	9,5	0,94	0,33	0,40
FOLD_ECODH	*folD*	Bifunctional protein FolD	13,889	31043,748	5,742	3		271822	341031	19,0	10,8	1,25	0,09	0,13
GCH1_ECODH	*folE*	GTP cyclohydrolase 1	34,234	24830,621	6,796	6		728044	895433	15,4	1,8	1,23	0,05	0,08
B1XGY7_ECODH	*folP*	7,8-dihydropteroate synthase	14,539	30615,111	5,677	3		195235	222808	11,4	8,9	1,14	0,13	0,17
B1X914_ECODH	*folX*	D-erythro-7,8-dihydroneopterin triphosphate 2'-epimera:	31,667	14082,037	6,509	4		947050	1242005	12,0	8,9	1,31	0,01	0,02
B1XB90_ECODH	*fpr*	Ferredoxin-NADP reductase	17,742	27750,938	6,174	2		55055	133122	14,8	17,2	2,42	0,00	0,00
B1XDQ8_ECODH	*frdA*	Fumarate reductase (Anaerobic) catalytic and NAD/flav	19,934	65971,602	5,863	8		3146135	331116	9,0	12,7	0,11	0,00	0,00
B1XEU9_ECODH	*frmA*	Alcohol dehydrogenase class III/glutathione-dependent	12,737	39358,992	5,849	4		607613	1014901	18,5	13,3	1,67	0,01	0,01
B1XEV0_ECODH	*frmR*	Regulator protein that represses frmRAB operon	26,374	10317,724	5,843	2		122134	124917	15,5	6,5	1,02	0,80	0,84
RRF_ECODH	*frr*	Ribosome-recycling factor	36,216	20638,572	6,436	5		6519852	8740024	14,3	5,4	1,34	0,01	0,02
FRSA_ECODH	*frsA*	Esterase FrsA	17,874	47008,77	6,465	5		237580	376685	27,1	7,3	1,59	0,03	0,05
B1X865_ECODH	*fruA*	Fused fructose-specific PTS enzymes: IIBcomponent II(	8,526	57519,113	8,911	4		533297	42911	17,1	24,2	0,08	0,00	0,00
B1X866_ECODH	*fruK*	Phosphofructokinase	22,115	33755,688	5,372	5		1080046	126654	11,8	5,8	0,12	0,00	0,00
B1X666_ECODH	*ftn*	Ferritin iron storage protein (Cytoplasmic)	27,879	19423,816	4,768	3		2302928	1763465	8,1	11,4	0,77	0,01	0,02
B1XC71_ECODH	*ftsA*	Cell division protein ftsA	31,905	45329,969	5,845	6		770387	980066	23,6	9,2	1,27	0,12	0,16
FTSB_ECODH	*ftsB*	Cell division protein FtsB	13,592	11622,075	6,568	1		15271	84566	102,8	18,1	5,54	0,01	0,01
B1XGY8_ECODH	*ftsH*	ATP-dependent zinc metalloprotease FtsH	37,422	70708,086	5,907	17		7239062	7602321	7,5	3,1	1,05	0,27	0,34
B1XC61_ECODH	*ftsI*	Transpeptidase involved in septal peptidoglycan synthe:	14,116	63877,434	9,644	5		219213	236865	11,8	7,4	1,08	0,32	0,39
B1X830_ECODH	*ftsK*	DNA-binding membrane protein required for chromosor	5,192	146662,72	4,916	3		173804	164433	4,5	18,6	0,95	0,58	0,65
B1XB99_ECODH	*ftsN*	Essential cell division protein	12,853	35793,23	10,17	3		320448	327635	11,5	3,2	1,02	0,71	0,76
B1X7S0_ECODH	*ftsX*	Cell division protein FtsX	9,659	38543,652	9,078	2		85311	130191	23,1	8,0	1,53	0,02	0,03
B1X7S2_ECODH	*ftsY*	Signal recognition particle receptor FtsY	34,004	54513,348	4,471	10		1381534	1555879	13,4	5,8	1,13	0,17	0,22
B1XC72_ECODH	*ftsZ*	Cell division protein FtsZ	53,525	40323,914	4,649	14		8848277	11367649	5,8	4,5	1,28	0,00	0,00
FUCI_ECODH	*fucI*	L-fucose isomerase	5,584	64976,672	5,586	3		287943	37818	17,4	19,8	0,13	0,00	0,00
B1XDK8_ECODH	*fucO*	L-1,2-propanediol oxidoreductase	7,833	40644,562	5,095	2		149165	30833	12,4	28,4	0,21	0,00	0,00
FUCM_ECODH	*fucU*	L-fucose mutarotase	26,429	15473,123	5,57	4		247656	313538	8,3	9,1	1,27	0,01	0,02
B1XF77_ECODH	*fumA*	Fumarate hydratase (Fumarase A), aerobic Class I	20,438	60298,578	6,114	8		5808238	1345903	9,2	10,3	0,23	0,00	0,00
B1XD03_ECODH	*fumB*	Anaerobic class I fumarate hydratase (Fumarase B)	24,453	60105,324	5,88	8		3232194	778531	5,0	8,6	0,24	0,00	0,00
EFG_ECODH	*fusA*	Elongation factor G	65,199	77581,305	5,236	30		131344424	136692171	11,2	2,4	1,04	0,51	0,58
B1XD20_ECODH	*fxsA*	Inner membrane protein	18,987	17691,945	8,062	2		99455	171916	6,5	7,7	1,73	0,00	0,00
B1XBW7_ECODH	*gabT*	4-aminobutyrate aminotransferase, PLP-dependent	25,822	45774,629	5,778	6		807241	325161	10,9	4,0	0,40	0,00	0,00
B1X7X2_ECODH	*gadA*	Glutamate decarboxylase A, PLP-dependent	48,283	52685,156	5,221	15	acid stress response	27203143	28967497	14,5	1,1	1,06	0,44	0,51
B1XE83_ECODH	*gadC*	Predicted glutamate:gamma-aminobutyric acid antiporte	5,284	55076,672	8,756	3	acid stress response	394920	679046	6,8	3,8	1,72	0,00	0,00
B1X789_ECODH	*galE*	UDP-glucose-4-epimerase	27,811	37325,207	5,887	6		1306312	795202	13,7	2,7	0,61	0,00	0,00
B1X6X8_ECODH	*galF*	Predicted subunit with GalU	50,842	32829,227	5,733	10		2804253	3031023	5,8	6,9	1,08	0,14	0,19
B1X787_ECODH	*galM*	Aldose 1-epimerase	55,78	38190,457	4,845	12		2798592	2573405	5,1	5,4	0,92	0,06	0,09
B1XAS8_ECODH	*galU*	Glucose-1-phosphate uridylyltransferase	40,066	32942,258	5,108	8		5212777	4889044	4,0	2,8	0,94	0,04	0,06
B1XGN9_ECODH	*gapA*	Glyceraldehyde-3-phosphate dehydrogenase A	69,184	35532,488	6,609	16		107287691	187095033	4,5	7,5	1,74	0,00	0,00
B1XGT8_ECODH	*garR*	Tartronate semialdehyde reductase	20,608	30659,734	5,585	3		244436	51591	16,7	8,3	0,21	0,00	0,00
B1X7I6_ECODH	*gatA*	Galactitol-specific enzyme IIA component of PTS	44,667	16907,457	5,148	5		7059180	3050217	6,3	7,4	0,43	0,00	0,00
B1X7I5_ECODH	*gatB*	Galactitol-specific enzyme IIB component of PTS	63,83	10222,037	5,84	3		11562664	6771784	25,8	12,5	0,59	0,02	0,03
B1X7I4_ECODH	*gatC*	Galactitol-specific enzyme IIC component of PTS	10,421	48364,996	9,319	5		3474304	2184196	8,0	5,8	0,63	0,00	0,00
B1X7I3_ECODH	*gatD*	Galactitol-1-phosphate dehydrogenase, Zn-dependent a	38,15	37390,137	5,939	10		5428339	2915636	4,8	8,6	0,54	0,00	0,00
GATY_ECODH	*gatY*	D-tagatose-1,6-bisphosphate aldolase subunit GatY	33,803	30811,932	5,874	6		9672090	5473013	6,8	5,9	0,57	0,00	0,00
GCSP_ECODH	*gcvP*	Glycine dehydrogenase (decarboxylating)	27,9	104376,35	5,623	16		6939598	9754193	6,1	4,0	1,41	0,00	0,00
B1XGM2_ECODH	*gdhA*	Glutamate dehydrogenase	15,213	48581,375	5,982	5		118377	212805	4,7	11,9	1,80	0,00	0,00
GHRA_ECODH	*ghrA*	Glyoxylate/hydroxypyruvate reductase A	23,397	35343,422	6,317	4		237582	603827	14,6	5,1	2,54	0,00	0,00
GHRB_ECODH	*ghrB*	Glyoxylate/hydroxypyruvate reductase B	26,235	35395,504	5,5	7		860293	1697014	9,3	3,6	1,97	0,00	0,00
B1XFD1_ECODH	*glcB*	Malate synthase G	14,938	80488,516	5,792	9		560147	421251	4,7	13,8	0,75	0,01	0,02
B1XFD2_ECODH	*glcG*	Conserved protein	11,194	13736,714	5,77	1		82445	57029	13,1	16,6	0,69	0,02	0,03

B1XBB1_ECODH	*gldA*	Glycerol dehydrogenase, NAD	24,796	38712,199	4,813	7		704695	176436	4,5	18,5	0,25	0,00	0,00
B1X6X2_ECODH	*glf*	UDP-galactopyranose mutase, FAD/NAD(P)-binding	39,237	42965,883	6,615	12		3475905	2612063	6,0	4,8	0,75	0,00	0,00
GLGA_ECODH	*glgA*	Glycogen synthase	12,998	52822,426	6,145	5		290004	423729	7,2	14,4	1,46	0,00	0,01
B1X777_ECODH	*glgB*	1,4-alpha-glucan branching enzyme GlgB	4,258	84336,898	5,912	2		69647	133980	17,3	17,4	1,92	0,00	0,00
GLGC_ECODH	*glgC*	Glucose-1-phosphate adenylyltransferase	19,722	48697,645	5,774	5		501598	670622	17,8	10,3	1,34	0,04	0,06
B1X773_ECODH	*glgP*	Phosphorylase	6,994	93172,555	5,572	4		145342	206868	57,6	9,0	1,42	0,21	0,27
GLK_ECODH	*glk*	Glucokinase	17,134	34723,086	6,061	5		1239235	979741	9,1	5,7	0,79	0,01	0,01
GLMM_ECODH	*glmM*	Phosphoglucosamine mutase	40,674	47543,574	5,711	13		3315975	3014650	3,9	4,2	0,91	0,02	0,03
GLMU_ECODH	*glmU*	Bifunctional protein GlmU	21,93	49190,086	6,087	8		708249	813938	8,0	5,0	1,15	0,03	0,05
B1XAM6_ECODH	*glnA*	Glutamine synthetase	47,761	51903,762	5,264	13		5450693	2812311	7,0	7,0	0,52	0,00	0,00
B1XB28_ECODH	*glnB*	Regulatory protein P-II for glutamine synthetase	33,929	12425,448	5,17	3		557122	622351	6,2	11,7	1,12	0,14	0,19
GLNE_ECODH	*glnE*	Glutamate-ammonia-ligase adenylyltransferase	3,383	108418,41	5,078	2		49040	70945	9,9	20,9	1,45	0,02	0,03
B1X7E1_ECODH	*glnH*	Glutamine transporter subunit periplasmic binding comp	45,968	27190,117	8,439	8		939650	553791	7,7	16,9	0,59	0,00	0,00
SYQ_ECODH	*glnS*	Glutamine--tRNA ligase	33,755	63477,891	5,886	15		7526797	6827294	6,6	4,8	0,91	0,06	0,08
GLO2_ECODH	*gloB*	Hydroxyacylglutathione hydrolase	27,49	28434,379	5,434	4		276486	275274	11,0	12,7	1,00	0,96	0,97
B1X8D5_ECODH	*glpA*	sn-glycerol-3-phosphate dehydrogenase (Anaerobic), la	35,978	58958,254	6,196	15		5782436	728434	7,2	3,8	0,13	0,00	0,00
GLPB_ECODH	*glpB*	Anaerobic glycerol-3-phosphate dehydrogenase subuni	27,208	45357,238	5,749	7		2232671	201906	10,4	5,7	0,09	0,00	0,00
B1X8D7_ECODH	*glpC*	sn-glycerol-3-phosphate dehydrogenase (Anaerobic), sı	26,768	44108,039	8,778	9		2226882	281104	6,2	6,2	0,13	0,00	0,00
B1X771_ECODH	*glpD*	sn-glycerol-3-phosphate dehydrogenase, aerobic, FAD/	59,481	56750,543	6,964	21		24075162	21795352	5,6	4,8	0,91	0,04	0,06
GLPK_ECODH	*glpK*	Glycerol kinase	61,155	56230,766	5,365	23		77451726	32584987	3,6	4,0	0,42	0,00	0,00
B1X8D3_ECODH	*glpQ*	Periplasmic glycerophosphodiester phosphodiesterase	52,514	40843,352	5,377	12		10322805	1932123	5,7	2,1	0,19	0,00	0,00
B1X768_ECODH	*glpR*	DNA-binding transcriptional repressor	8,73	28047,838	5,821	2		143237	153745	5,9	11,6	1,07	0,32	0,39
B1X8D4_ECODH	*glpT*	sn-glycerol-3-phosphate transporter	7,743	50310,387	8,684	3		2169930	266658	18,2	7,6	0,12	0,00	0,00
B1XB91_ECODH	*glpX*	Fructose-1,6-bisphosphatase	16,369	35852,309	5,325	5		436638	346701	6,9	8,9	0,79	0,01	0,01
B1X6Q1_ECODH	*gltA*	Citrate synthase	45,199	48014,992	6,213	14	metabolism	8134109	4776414	4,6	5,0	0,59	0,00	0,00
B1XHI7_ECODH	*gltB*	Glutamate synthase, large subunit	21,49	166709,88	6,265	23		745388	1425876	8,7	5,7	1,91	0,00	0,00
B1XHI8_ECODH	*gltD*	Glutamate synthase, 4Fe-4S protein, small subunit	13,559	52015,273	5,537	4		240841	436213	12,0	10,6	1,81	0,00	0,00
B1X650_ECODH	*gltI*	Glutamate and aspartate transporter subunit, periplasm	10,265	33420,164	8,61	2		78548	57254	19,2	31,6	0,73	0,17	0,22
SYE_ECODH	*gltX*	Glutamate--tRNA ligase	32,484	53815,727	5,593	12		5652760	5018214	4,1	2,1	0,89	0,00	0,01
GLYA_ECODH	*glyA*	Serine hydroxymethyltransferase	39,089	45316,594	6,028	11		8110992	7715199	6,3	6,5	0,95	0,32	0,38
SYGB_ECODH	*glyS*	Glycine--tRNA ligase beta subunit	49,927	76812,945	5,291	25		9774754	10191848	5,6	3,7	1,04	0,27	0,34
GMHA_ECODH	*gmhA*	Phosphoheptose isomerase	36,458	20814,748	5,966	6		1810557	1675282	4,7	5,1	0,93	0,06	0,10
B1X6W6_ECODH	*gnd*	6-phosphogluconate dehydrogenase, decarboxylating	62,821	51481,309	5,055	20		31560622	48274628	6,1	4,6	1,53	0,00	0,00
B1X8U6_ECODH	*gnsA*	Predicted regulator of phosphatidylethanolamine synthe	40,351	6575,613	5,392	2		1589437	2748338	10,7	12,0	1,73	0,00	0,00
B1XF19_ECODH	*gnsB*	Uncharacterized protein	12,281	6547,669	8,963	1		86146	469044	54,0	7,7	5,44	0,00	0,00
B1X7V5_ECODH	*gor*	Glutathione oxidoreductase	18	48772,516	5,639	5		1098395	1157680	11,7	3,0	1,05	0,42	0,49
B1X732_ECODH	*gph*	Phosphoglycolate phosphatase	22,222	27389,166	4,578	3		419519	506432	12,0	5,3	1,21	0,03	0,05
GPMA_ECODH	*gpmA*	2,3-bisphosphoglycerate-dependent phosphoglycerate r	32,8	28556,404	5,851	7		14240771	13731147	7,5	5,1	0,96	0,44	0,51
B1X946_ECODH	*gpmI*	2,3-bisphosphoglycerate-independent phosphoglycerate	36,381	56193,887	5,141	11		7942175	7343311	5,9	6,6	0,92	0,13	0,17
GPPA_ECODH	*gppA*	Guanosine-5'-triphosphate,3'-diphosphate pyrophospha	6,275	54871,141	5,946	3		60890	203973	18,4	104,3	3,35	0,03	0,05
GPDA_ECODH	*gpsA*	Glycerol-3-phosphate dehydrogenase [NAD(P)+]	41,298	36361,602	6,085	7		528453	716765	18,7	1,6	1,36	0,02	0,04
XGPT_ECODH	*gpt*	Xanthine phosphoribosyltransferase	42,763	16970,588	5,52	4		458157	512507	2,9	13,3	1,12	0,14	0,18
GRCA_ECODH	*grcA*	Autonomous glycyl radical cofactor	74,016	14284,189	5,087	9		16580981	905598	4,4	45,8	0,05	0,00	0,00
B1XGZ1_ECODH	*greA*	Transcription elongation factor GreA	55,063	17640,957	4,712	7		2075949	1652150	9,0	10,6	0,80	0,02	0,03
CH60_ECODH	*groL*	60 kDa chaperonin	69,708	57328,848	4,845	24		71878789	140652343	7,9	6,8	1,96	0,00	0,00
CH10_ECODH	*groS*	10 kDa chaperonin	51,546	10386,952	5,147	4		20955487	31062283	22,3	10,1	1,48	0,02	0,03
GRPE_ECODH	*grpE*	Protein GrpE	29,949	21797,83	4,681	5		11356294	10361325	8,2	5,0	0,91	0,11	0,15
B1X9H7_ECODH	*grxB*	Glutaredoxin 2	47,442	24349,266	8,717	8		1187584	1859199	6,7	7,0	1,57	0,00	0,00
B1X944_ECODH	*grxC*	Glutaredoxin 3	68,675	9137,494	6,707	4		1657836	3718201	16,1	3,2	2,24	0,00	0,00
B1XFW5_ECODH	*grxD*	Glutaredoxin	46,087	12878,761	4,75	4		5083433	5952000	5,0	6,4	1,17	0,01	0,02
GSH1_ECODH	*gshA*	Glutamate--cysteine ligase	16,409	58269,293	5,273	6		406350	793432	15,9	5,2	1,95	0,00	0,00
B1XFA7_ECODH	*gshB*	Glutathione synthetase	31,646	35560,898	5,109	7		1235538	1680286	7,2	3,0	1,36	0,00	0,00
B1XFR4_ECODH	*gsk*	Inosine/guanosine kinase	14,516	48448,781	5,502	4		200868	164824	15,5	8,7	0,82	0,08	0,12
B1XFE4_ECODH	*gss*	Fused glutathionylspermidine amidase glutathionylsperr	8,562	70531,961	5,13	4		256805	577015	12,9	7,5	2,25	0,00	0,00
B1XFU7_ECODH	*gst*	Glutathionine S-transferase	34,826	22868,369	5,849	5		611195	912556	9,3	7,0	1,49	0,00	0,00
GUAA_ECODH	*guaA*	GMP synthase [glutamine-hydrolyzing]	43,429	58679,223	5,239	16		9029129	12247072	2,1	4,9	1,36	0,00	0,00
B1XAY3_ECODH	*guaB*	Inosine-5'-monophosphate dehydrogenase	46,107	52022,453	6,016	12		7710779	12200471	6,5	5,9	1,58	0,00	0,00
GUAC_ECODH	*guaC*	GMP reductase	24,784	37383,668	6,103	4		819052	960885	33,6	6,9	1,17	0,44	0,51
B1XCN5_ECODH	*gutQ*	Arabinose 5-phosphate isomerase	20,249	34031,43	5,919	4		206208	196782	8,1	17,4	0,95	0,64	0,70
B1X8C5_ECODH	*gyrA*	DNA gyrase subunit A	27,314	96963,516	5,086	20		7962950	7052134	3,5	3,5	0,89	0,00	0,01
HCHA_ECODH	*hchA*	Molecular chaperone Hsp31 and glyoxalase 3	28,975	31190,463	5,628	6		3534679	4908580	8,2	6,9	1,39	0,00	0,00
B1X7W5_ECODH	*hdeA*	Acid stress chaperone HdeA	54,545	11857,629	5,056	2	acid stress response	534489	1549177	33,2	29,8	2,90	0,01	0,01
B1X7W4_ECODH	*hdeB*	Acid stress chaperone HdeB	43,519	12042,821	5,727	3	acid stress response	554124	1904710	14,6	23,3	3,44	0,00	0,00
B1X7W6_ECODH	*hdeD*	Acid-resistance membrane protein	6,316	20903,369	9,553	2	acid stress response	35149	159347	41,3	17,0	4,53	0,00	0,00
B1XF84_ECODH	*hdhA*	7-alpha-hydroxysteroid dehydrogenase, NAD-depender	34,902	26778,59	5,216	6		905859	2443005	5,5	4,5	2,70	0,00	0,00
B1XEW0_ECODH	*hemB*	Delta-aminolevulinic acid dehydratase	22,531	35624,793	5,25	5		1491537	1688486	22,0	6,0	1,13	0,33	0,39
HEM3_ECODH	*hemC*	Porphobilinogen deaminase	54,313	33851,836	5,312	10		625231	688313	13,1	10,5	1,10	0,30	0,36
DCUP_ECODH	*hemE*	Uroporphyrinogen decarboxylase	24,011	39248,117	5,882	6		1004480	1191732	10,7	10,8	1,19	0,07	0,10
B1XAL2_ECODH	*hemG*	Protoporphyrin oxidase, flavoprotein	24,862	21226,301	9,678	4		307344	326933	21,3	12,1	1,06	0,62	0,68
GSA_ECODH	*hemL*	Glutamate-1-semialdehyde 2,1-aminomutase	37,793	45366,098	4,733	11		6434240	7323966	8,8	4,6	1,14	0,04	0,06
B1XAM3_ECODH	*hemN*	Coproporphyrinogen-III oxidase	9,409	52729,043	5,7	3		144003	215456	4,0	16,5	1,50	0,00	0,01
B1XAH0_ECODH	*hemY*	Predicted protoheme IX synthesis protein	31,156	45245,023	8,525	8		2341316	1129271	15,5	15,5	0,48	0,00	0,00
B1XDS6_ECODH	*hflC*	Modulator of FtsH protease HflC	35,629	37649,883	6,299	8	degradation/folding	1405943	1370556	9,9	1,2	0,97	0,62	0,68
HFLD_ECODH	*hflD*	High frequency lysogenization protein HflD	15,493	22947,547	9,338	2		202583	152542	9,7	12,2	0,75	0,01	0,02

B1XDS5_ECODH	*hflK*	Modulator for HflB protease specific for phage lambda c	39,618	45544,883	6,189	12	degradation/folding	4133618	5560066	11,5	7,9	1,35	0,01	0,01
B1XDS4_ECODH	*hflX*	GTPase HflX	38,028	48327,102	5,684	11	metabolism	750564	1117731	8,4	6,7	1,49	0,00	0,00
HFQ_ECODH	*hfq*	RNA-binding protein Hfq	21,569	11166,386	6,97	2		997738	1309777	17,6	13,9	1,31	0,05	0,08
B1XFP7_ECODH	*hha*	Haemolysin expression modulating protein	11,111	8678,061	8,787	1		14814	29926	68,8	19,3	2,02	0,08	0,12
B1XA13_ECODH	*hinT*	Purine nucleoside phosphoramidase	25,21	13241,277	5,731	2		842422	1732733	22,4	4,9	2,06	0,00	0,00
B1X6V9_ECODH	*hisB*	Histidine biosynthesis bifunctional protein HisB	16,338	40277,965	5,763	5		281196	438203	21,5	7,5	1,56	0,01	0,02
HIS6_ECODH	*hisF*	Imidazole glycerol phosphate synthase subunit HisF	14,729	28454,453	5,034	3		103802	154695	9,9	8,8	1,49	0,00	0,00
HIS1_ECODH	*hisG*	ATP phosphoribosyltransferase	27,425	33366,715	5,471	4		246225	291596	20,3	10,2	1,18	0,22	0,28
B1X6W0_ECODH	*hisH*	Imidazole glycerol phosphate synthase subunit HisH	16,327	21652,857	5,335	2		74595	91558	16,2	16,4	1,23	0,14	0,19
B1X6W3_ECODH	*hisI*	Histidine biosynthesis bifunctional protein HisIE	17,241	22755,818	5,245	1		42160	92781	48,8	6,5	2,20	0,05	0,08
B1X920_ECODH	*hisJ*	Histidine/lysine/arginine/ornithine transporter subunit pe	26,154	28483,377	5,465	4	transport	332016	392773	10,4	14,2	1,18	0,11	0,15
SYH_ECODH	*hisS*	Histidine--tRNA ligase	44,811	47029,441	5,647	15		5096946	5264687	6,4	2,8	1,03	0,39	0,46
HLDD_ECODH	*hldD*	ADP-L-glycero-D-manno-heptose-6-epimerase	47,742	34893,168	4,8	13		8060830	7760829	9,9	2,1	0,96	0,47	0,54
HLDE_ECODH	*hldE*	Bifunctional protein HldE	37,317	51050,617	5,294	12		2975409	2946522	9,0	2,1	0,99	0,84	0,87
B1XB27_ECODH	*hmp*	Flavohemoprotein	15,152	43867,656	5,484	6		378381	338360	5,0	3,5	0,89	0,01	0,02
B1XAS9_ECODH	*hns*	DNA-binding protein	54,015	15539,633	5,436	6		24184214	23762170	5,6	5,1	0,98	0,65	0,71
B1XHH8_ECODH	*hpf*	Ribosomal hibernation promoting factor	17,895	10750,249	6,5	1		39106	66423	7,7	15,5	1,70	0,00	0,00
B1XCA0_ECODH	*hpt*	Hypoxanthine phosphoribosyltransferase	44,944	20115,238	5,091	7		1190083	1010625	12,6	5,7	0,85	0,06	0,08
B1XDC6_ECODH	*hrpA*	ATP-dependent helicase	5,923	149027,92	7,887	6		394218	322709	8,5	2,2	0,82	0,00	0,01
HSCA_ECODH	*hscA*	Chaperone protein HscA	20,942	65652,438	4,983	8		1273386	1254324	6,5	4,2	0,99	0,71	0,76
HSCB_ECODH	*hscB*	Co-chaperone protein HscB	13,45	20137,709	5,048	2		394291	386310	11,3	13,3	0,98	0,82	0,86
B1XD94_ECODH	*hslJ*	Heat-inducible protein	27,143	15165,621	6,721	2		148594	152145	30,5	11,7	1,02	0,91	0,92
HSLO_ECODH	*hslO*	33 kDa chaperonin	29,11	32534,477	4,354	5		1146412	2207330	12,6	10,7	1,93	0,00	0,00
HSLU_ECODH	*hslU*	ATP-dependent protease ATPase subunit HslU	30,926	49593,805	5,239	10		5985088	10534082	3,8	9,5	1,76	0,00	0,00
B1XFR0_ECODH	*htpG*	Chaperone protein HtpG	58,333	71422,531	5,086	28		27381473	40543293	2,1	6,8	1,48	0,00	0,00
HTPX_ECODH	*htpX*	Protease HtpX	16,041	31923,312	6,605	3	degradation/folding	809019	949578	8,4	15,2	1,17	0,12	0,17
B1XG60_ECODH	*htrG*	Predicted signal transduction protein (SH3 domain)	14,078	23076,389	9,096	3		481823	674262	5,9	6,9	1,40	0,00	0,00
B1XC03_ECODH	*hupA*	HU, DNA-binding transcriptional regulator, alpha subuni	57,778	9534,985	9,572	4		30576421	30200431	7,7	5,7	0,99	0,80	0,84
B1XFM8_ECODH	*hupB*	HU, DNA-binding transcriptional regulator, beta subunit	15,556	9225,579	9,694	1		985714	353620	33,1	21,5	0,36	0,00	0,01
B1XFF2_ECODH	*hybA*	Hydrogenase 2 4Fe-4S ferredoxin-type component	9,756	36002,996	7,104	2		74901	17428	29,2	110,2	0,23	0,02	0,04
B1XFF0_ECODH	*hybC*	Hydrogenase 2, large subunit	18,519	62491,16	5,843	6		921428	188706	13,1	4,6	0,20	0,00	0,00
IBPA_ECODH	*ibpA*	Small heat shock protein IbpA	37,226	15773,711	5,574	5		923405	2243864	15,3	13,6	2,43	0,00	0,00
IBPB_ECODH	*ibpB*	Small heat shock protein IbpB	37,324	16093,197	5,189	4		364491	907316	19,2	12,3	2,49	0,00	0,00
B1XA46_ECODH	*icd*	Isocitrate dehydrogenase [NADP]	38,702	45784,762	5,163	12		18780688	16616210	10,7	5,5	0,88	0,09	0,13
ICIA_ECODH	*iciA*	Chromosome initiation inhibitor	8,081	33471,688	6,402	2		99231	153419	17,4	7,7	1,55	0,00	0,01
IHFA_ECODH	*ihfA*	Integration host factor subunit alpha	41,414	11353,932	9,342	4		2230837	3987252	6,7	11,2	1,79	0,00	0,00
IHFB_ECODH	*ihfB*	Integration host factor subunit beta	26,596	10651,144	9,344	2		786442	837585	13,6	10,8	1,07	0,49	0,56
SYI_ECODH	*ileS*	Isoleucine--tRNA ligase	44,456	104296,84	5,657	29		12400238	13095076	5,2	3,0	1,06	0,12	0,16
B1X9A4_ECODH	*ilvB*	Acetolactate synthase	16,192	60440,57	5,302	4	metabolism	107900	200215	22,6	8,2	1,86	0,00	0,01
ILVC_ECODH	*ilvC*	Ketol-acid reductoisomerase	10,998	54069,02	5,204	5	metabolism	96932	253038	6,7	81,3	2,61	0,03	0,05
B1X9Y7_ECODH	*ilvE*	Branched-chain amino-acid aminotransferase	14,887	34093,652	5,54	3		191533	432655	11,5	9,9	2,26	0,00	0,00
B1X9A3_ECODH	*ilvN*	Acetolactate synthase I, small subunit	10,417	11105,758	5,69	1	metabolism	14387	49071	42,5	19,8	3,41	0,00	0,01
B1XC55_ECODH	*imp*	LPS-assembly protein LptD	37,372	89671,148	4,936	20		4090624	2932366	5,2	4,9	0,72	0,00	0,00
B1X824_ECODH	*infA*	Translation initiation factor IF-1	27,778	8249,575	9,22	1		162569	135840	30,7	10,6	0,84	0,39	0,46
IF2_ECODH	*infB*	Translation initiation factor IF-2	31,573	97349,898	5,802	20		13352697	11617892	7,5	1,8	0,87	0,01	0,02
IRAP_ECODH	*iraP*	Anti-adapter protein IraP	31,395	9937,478	4,818	1		33265	22020	14,4	18,1	0,66	0,01	0,02
ISCR_ECODH	*iscR*	HTH-type transcriptional regulator IscR	29,012	17336,561	6,824	2		296800	232774	10,1	16,1	0,78	0,05	0,08
ISCS_ECODH	*iscS*	Cysteine desulfurase IscS	46,782	45089,5	5,943	15		9256794	10474951	9,2	6,8	1,13	0,07	0,11
B1XB04_ECODH	*iscU*	Scaffold protein	60,938	13848,59	4,815	5		2880071	4642663	4,7	10,0	1,61	0,00	0,00
B1XF09_ECODH	*ispA*	Geranyltranstransferase	24,08	32159,633	5,273	4		530637	434782	9,9	8,2	0,82	0,02	0,04
ISPF_ECODH	*ispF*	2-C-methyl-D-erythritol 2,4-cyclodiphosphate synthase	19,497	16897,537	6,044	2		240887	424307	32,6	7,7	1,76	0,03	0,05
ISPG_ECODH	*ispG*	4-hydroxy-3-methylbut-2-en-1-yl diphosphate synthase	42,742	40683,613	5,871	14		2177761	2598941	6,4	3,9	1,19	0,00	0,01
ISPH_ECODH	*ispH*	4-hydroxy-3-methylbut-2-enyl diphosphate reductase	13,608	34774,559	5,196	3		399029	343217	11,4	2,8	0,86	0,04	0,07
B1XD82_ECODH	*ivy*	Inhibitor of vertebrate C-lysozyme	35,032	16872,289	6,267	5		252085	722405	13,2	13,0	2,87	0,00	0,00
B1XGJ3_ECODH	*katE*	Catalase	17,928	84162,617	5,541	9		1264073	1528355	13,5	8,2	1,21	0,05	0,08
KATG_ECODH	*katG*	Catalase-peroxidase	37,328	80023,812	5,139	20		8369889	6758873	5,7	7,0	0,81	0,00	0,01
B1X951_ECODH	*kbl*	Glycine C-acetyltransferase	33,668	43117,039	5,64	10		5273266	5876293	9,3	6,6	1,11	0,10	0,15
B1X8E4_ECODH	*kdgK*	Ketodeoxygluconokinase	16,505	33962,359	4,923	3		570285	423415	10,0	10,4	0,74	0,01	0,01
B1XH95_ECODH	*kdgR*	Predicted DNA-binding transcriptional regulator	38,403	30029,348	5,435	8		981038	885297	7,7	2,6	0,90	0,04	0,07
KDSA_ECODH	*kdsA*	2-dehydro-3-deoxyphosphooctonate aldolase	48,944	30832,693	6,32	11		6363419	6847448	11,0	6,1	1,08	0,30	0,36
KDSB_ECODH	*kdsB*	3-deoxy-manno-octulosonate cytidylyltransferase	35,081	27614,439	5,147	6		920407	1129973	9,7	0,4	1,23	0,00	0,01
B1XHH3_ECODH	*kdsC*	3-deoxy-D-manno-octulosonate 8-phosphate phosphata	29,787	19997,113	4,942	3		366882	310949	8,2	14,9	0,85	0,11	0,16
B1XHH2_ECODH	*kdsD*	Arabinose 5-phosphate isomerase	5,793	35196,086	6,219	2		100038	142095	13,3	6,6	1,42	0,00	0,01
B1X967_ECODH	*kdtA*	3-deoxy-D-manno-octulosonic-acid transferase (KDO tr	6,118	47291,082	9,797	2		87220	78538	13,2	12,4	0,90	0,30	0,36
B1XFQ2_ECODH	*kefA*	Fused conserved protein mechanosensitive channel pro	7,679	127215,28	8,045	6		235764	414530	14,2	6,1	1,76	0,00	0,00
B1XBJ2_ECODH	*lacI*	DNA-binding transcriptional repressor	45,278	38590,164	6,39	11		1719418	80935046	10,6	5,2	47,07	0,00	0,00
LAMB_ECODH	*lamB*	Maltoporin	37,892	49912,227	4,807	10		3407953	703519	3,7	8,0	0,21	0,00	0,00
B1XD95_ECODH	*ldhA*	Fermentative D-lactate dehydrogenase, NAD-depender	21,581	36534,793	5,285	5		893013	1315402	3,7	6,7	1,47	0,00	0,00
LEPA_ECODH	*lepA*	Elongation factor 4	33,556	66570,273	5,401	15		3585084	3325255	8,2	5,5	0,93	0,18	0,23
B1X637_ECODH	*leuS*	Leucine--tRNA ligase	34,767	97233,758	5,156	21		13253120	7531543	3,4	3,6	0,57	0,00	0,00
LEXA_ECODH	*lexA*	LexA repressor	11,386	22357,719	6,229	2		127443	119940	15,1	18,3	0,94	0,62	0,68
LGT_ECODH	*lgt*	Prolipoprotein diacylglyceryl transferase	20,962	33107,949	9,515	3		220032	196674	5,0	18,6	0,89	0,29	0,35
DNLJ_ECODH	*ligA*	DNA ligase	14,158	73606,07	5,385	9		777280	878123	9,2	7,3	1,13	0,08	0,12
B1X623_ECODH	*lipA*	Lipoyl synthase	19,315	36071,715	8,084	5		1921475	1113144	9,9	5,3	0,58	0,00	0,00

LLDD_ECODH	*lldD*	L-lactate dehydrogenase [cytochrome]	34,091	42728,191	6,332	9		3025801	544494	14,2	15,1	0,18	0,00	0,00
LOLA_ECODH	*lolA*	Outer-membrane lipoprotein carrier protein	43,35	22497,055	6,278	6		547191	1257917	7,4	13,9	2,30	0,00	0,00
LOLB_ECODH	*lolB*	Outer-membrane lipoprotein LolB	46,377	23550,77	8,886	7		806114	832839	10,2	5,6	1,03	0,59	0,66
B1XA26_ECODH	*lolC*	Outer membrane-specific lipoprotein transporter subuni	11,028	43263,762	6,043	3		157281	196643	14,3	11,2	1,25	0,05	0,08
B1XFM7_ECODH	*lon*	Lon protease	41,071	87438,125	6,012	22		6651655	11497650	9,8	7,5	1,73	0,00	0,00
B1XC91_ECODH	*lpd*	Dihydrolipoyl dehydrogenase	43,038	50688,492	5,789	15		41789445	32388083	11,6	2,4	0,78	0,01	0,01
LPLA_ECODH	*lplA*	Lipoate-protein ligase A	11,834	37925,883	5,708	2		182005	193745	16,8	15,1	1,06	0,60	0,67
B1XFY5_ECODH	*lpp*	Murein lipoprotein	33,333	8323,479	9,3	2		36947808	58155118	21,7	3,5	1,57	0,00	0,01
LPXA_ECODH	*lpxA*	Acyl-[acyl-carrier-protein]--UDP-N-acetylglucosamine O	17,557	28079,996	6,63	3		459346	423020	14,5	12,7	0,92	0,44	0,51
B1XD48_ECODH	*lpxD*	UDP-3-O-(3-hydroxymyristoyl)glucosamine N-acyltransf	22,287	36038,484	6,073	4		808670	932823	9,4	6,1	1,15	0,04	0,07
B1X9G7_ECODH	*lpxL*	Lauryl-acyl carrier protein (ACP)-dependent acyltransfe	11,438	35406,824	9,805	2		73419	137634	19,8	19,8	1,87	0,00	0,01
B1X901_ECODH	*lrhA*	DNA-binding transcriptional repressor of flagellar, motili	10,897	34593,828	5,238	2	metabolism	122327	42692	13,3	4,9	0,35	0,00	0,00
B1X829_ECODH	*lrp*	DNA-binding transcriptional dual regulator, leucine-bindi	26,22	18886,783	8,904	4		923182	1280675	4,7	9,8	1,39	0,00	0,00
B1X811_ECODH	*ltaE*	L-allo-threonine aldolase, PLP-dependent	16,216	36494,707	5,807	5		334103	583631	11,3	10,5	1,75	0,00	0,00
LUXS_ECODH	*luxS*	S-ribosylhomocysteine lyase	50,877	19416,189	5,181	6		3495988	9003196	9,6	8,0	2,58	0,00	0,00
B1XC23_ECODH	*lysC*	Aspartokinase	7,795	48531,73	5,025	2		42636	88335	17,9	14,9	2,07	0,00	0,00
B1XEH7_ECODH	*lysS*	Lysine--tRNA ligase	41,98	57603,422	5,106	14		5897358	5207268	8,1	5,9	0,88	0,05	0,07
B1XD10_ECODH	*lysU*	Lysine--tRNA ligase	47,327	57826,57	5,104	19		12151150	16143726	6,9	8,1	1,33	0,00	0,01
MAO1_ECODH	*maeA*	NAD-dependent malic enzyme	43,009	63197,328	5,188	16		3789475	3595734	5,4	2,2	0,95	0,12	0,16
B1XAD4_ECODH	*maeB*	Fused malic enzyme predicted oxidoreductase predicte	38,208	82417,383	5,338	20		7557969	2710818	5,0	3,7	0,36	0,00	0,00
B1XEY3_ECODH	*mak*	Manno(Fructo)kinase	11,258	32499,744	5,48	2		139695	164906	16,5	5,5	1,18	0,11	0,16
B1XC33_ECODH	*malE*	Maltose transporter subunit periplasmic-binding compor	37,626	43387,641	5,534	10		5070923	614652	9,9	12,0	0,12	0,00	0,00
B1XC32_ECODH	*malF*	Maltose transporter subunit membrane component of A	18,482	57013,402	8,477	5		397771	52461	13,7	20,0	0,13	0,00	0,00
B1XC34_ECODH	*malK*	Fused maltose transport subunit, ATP-binding compone	15,094	40990,453	6,228	4		1041012	142583	6,1	7,5	0,14	0,00	0,00
B1XC36_ECODH	*malM*	Maltose regulon periplasmic protein	14,706	31943,5	7,72	3		1619708	229838	9,3	12,3	0,14	0,00	0,00
B1X763_ECODH	*malP*	Phosphorylase	33,124	90522,398	6,938	18		4169601	1646883	2,8	2,5	0,39	0,00	0,00
B1X762_ECODH	*malQ*	4-alpha-glucanotransferase (Amylomaltase)	10,519	78503,492	6,138	5		327543	147278	4,4	21,0	0,45	0,00	0,00
MALT_ECODH	*malT*	HTH-type transcriptional regulator MalT	5,66	103118,45	6,028	3		296051	180673	10,0	8,0	0,61	0,00	0,00
B1XF78_ECODH	*manA*	Mannose-6-phosphate isomerase	29,923	42849,949	5,29	8		2234063	2062472	6,5	4,9	0,92	0,10	0,15
B1XH84_ECODH	*manX*	Fused mannose-specific PTS enzymes: IIA component	51,084	35047,551	5,742	10		5952514	3647182	4,1	3,6	0,61	0,00	0,00
B1XH86_ECODH	*manZ*	Mannose-specific enzyme IID component of PTS	21,329	31302,873	9,171	5		1835094	1120806	14,6	7,7	0,61	0,00	0,00
B1XD37_ECODH	*map*	Methionine aminopeptidase	29,545	29330,799	5,641	4		1744471	1697972	22,9	3,5	0,97	0,84	0,87
B1XG33_ECODH	*mdaB*	NADPH quinone reductase	12,953	21890,906	5,845	2		44320	88854	10,3	20,6	2,00	0,00	0,00
MDH_ECODH	*mdh*	Malate dehydrogenase	66,987	32337,299	5,607	13		27639542	18962315	6,5	5,7	0,69	0,00	0,00
OPGD_ECODH	*mdoD*	Glucans biosynthesis protein D	15,064	62757,945	5,893	5	metabolism	160453	106530	21,5	7,3	0,66	0,01	0,02
OPGG_ECODH	*mdoG*	Glucans biosynthesis protein G	30,724	57912,484	6,7	13		3613184	4022214	7,8	4,9	1,11	0,06	0,09
B1X9G3_ECODH	*mdoH*	Glucans biosynthesis glucosyltransferase H	10,39	96953,43	8,871	6		554545	194685	10,3	7,9	0,35	0,00	0,00
MDTA_ECODH	*mdtA*	Multidrug resistance protein MdtA	6,506	44464,289	8,903	2		23266	44623	36,5	12,7	1,92	0,03	0,05
B1X7W8_ECODH	*mdtE*	Multidrug resistance efflux transporter	42,857	41190,57	5,73	9		393338	1068979	10,2	2,7	2,72	0,00	0,00
B1X7W9_ECODH	*mdtF*	Multidrug transporter, RpoS-dependent	8,004	111517,17	5,326	5		70557	187114	27,9	14,4	2,65	0,00	0,00
MDTK_ECODH	*mdtK*	Multidrug resistance protein MdtK	6,565	49447,047	9,63	2		80689	67903	17,1	7,0	0,84	0,10	0,14
B1X8X6_ECODH	*menB*	Dihydroxynaphthoic acid synthetase	29,825	31633,08	5,994	7		1654212	1384446	5,6	3,3	0,84	0,00	0,00
MENC_ECODH	*menC*	o-succinylbenzoate synthase	10,625	35476,613	4,836	2		141462	111784	11,2	13,2	0,79	0,04	0,06
METE_ECODH	*metE*	5-methyltetrahydropteroyltriglutamate--homocysteine me	14,475	84673,539	5,609	8		105289	523297	39,2	9,0	4,97	0,00	0,00
SYM_ECODH	*metG*	Methionine--tRNA ligase	41,802	76254,734	5,565	21		7789794	7032804	5,5	2,0	0,90	0,01	0,03
B1XC18_ECODH	*metH*	Homocysteine-N5-methyltetrahydrofolate transmethylas	16,87	135997,05	4,97	14		529604	793926	7,5	4,3	1,50	0,00	0,00
METK_ECODH	*metK*	S-adenosylmethionine synthase	50,26	41951,625	5,096	13		5592313	5553377	16,1	6,6	0,99	0,94	0,95
B1XBA6_ECODH	*metL*	Fused aspartokinase II homoserine dehydrogenase II	7,901	88887,695	5,343	4		97858	110700	11,2	8,1	1,13	0,12	0,17
B1XD69_ECODH	*metN*	ATP-binding component of DL-methionine uptake trans	32,362	37788,371	5,947	6		336229	617318	1,7	3,5	1,84	0,00	0,00
B1XD67_ECODH	*metQ*	Lipoprotein	53,506	29431,65	5,127	8		1626926	2757153	18,0	4,9	1,69	0,00	0,00
B1XA24_ECODH	*mfd*	Transcription-repair-coupling factor	13,415	129982,73	5,787	13		2707625	3393196	3,3	8,3	1,25	0,00	0,01
B1X7N8_ECODH	*mglB*	Methyl-galactoside transporter subunit periplasmic-bind	34,337	35712,574	5,68	8		6766580	366641	14,2	9,6	0,05	0,00	0,00
MGSA_ECODH	*mgsA*	Methylglyoxal synthase	19,737	16918,576	6,12	2		589560	361771	18,4	25,8	0,61	0,02	0,03
B1XEM3_ECODH	*mgtA*	Magnesium transporter	15,924	99466,484	5,635	11		888880	2771532	8,1	3,9	3,12	0,00	0,00
MIAA_ECODH	*miaA*	tRNA dimethylallyltransferase	18,354	35065,152	5,682	4	metabolism	183851	180431	12,9	83,7	0,98	0,96	0,97
MIAB_ECODH	*miaB*	tRNA-2-methylthio-N(6)-dimethylallyladenosine synthas	36,92	53662,957	5,197	14		4113812	3844982	5,5	1,1	0,93	0,06	0,09
B1XA62_ECODH	*minD*	Site-determining protein	44,444	29614,033	5,246	9		5580154	6699667	4,1	2,2	1,20	0,00	0,00
MINE_ECODH	*minE*	Cell division topological specificity factor	54,545	10234,906	5,147	3		934685	1025824	7,6	7,4	1,10	0,14	0,19
B1XGP2_ECODH	*mipA*	Scaffolding protein for murein synthesizing machinery	31,048	27830,986	5,501	5		3670085	1864930	12,5	8,8	0,51	0,00	0,00
B1XDM2_ECODH	*mltA*	Membrane-bound lytic murein transglycosylase A	15,89	40410,609	9,041	3		482508	507612	9,7	22,7	1,05	0,71	0,76
B1XCM8_ECODH	*mltB*	Membrane-bound lytic murein transglycosylase B	21,33	40255,746	9,068	4		317407	283848	6,9	11,8	0,89	0,16	0,21
MNMC_ECODH	*mnmC*	tRNA 5-methylaminomethyl-2-thiouridine biosynthesis b	6,737	74434,164	5,43	2		46024	66002	13,5	9,2	1,43	0,00	0,01
MNME_ECODH	*mnmE*	tRNA modification GTPase MnmE	9,692	49230,871	4,892	3		121346	166811	10,3	11,8	1,37	0,01	0,02
MNMG_ECODH	*mnmG*	tRNA uridine 5-carboxymethylaminomethyl modification	19,714	69521,258	6,195	8		774735	496360	6,7	6,5	0,64	0,00	0,00
B1X7B2_ECODH	*moaB*	Molybdopterin biosynthesis protein B	29,412	18665,094	5,727	4		1551415	1377729	12,8	6,8	0,89	0,17	0,23
MOAC_ECODH	*moaC*	Cyclic pyranopterin monophosphate synthase accessor	24,224	17467,236	6,593	3		453317	451405	11,6	10,8	1,00	0,96	0,97
B1X7B4_ECODH	*moaD*	Molybdopterin synthase, small subunit	37,037	8757,937	4,381	2		236372	231565	11,4	5,8	0,98	0,76	0,81
B1X7B5_ECODH	*moaE*	Molybdopterin synthase, large subunit	23,333	16981,055	5,256	2		479309	366025	9,1	20,8	0,76	0,07	0,11
B1X791_ECODH	*modE*	DNA-binding transcriptional dual regulator	27,099	28281,225	5,22	3		215666	160768	18,4	22,7	0,75	0,11	0,15
B1X7F7_ECODH	*moeA*	Molybdopterin biosynthesis protein	40,876	44067,273	5,026	8		1796226	1957697	4,7	4,5	1,09	0,04	0,06
B1XBD4_ECODH	*mog*	Predicted molybdochelatase	41,026	21222,354	4,971	6		740304	472877	10,6	14,1	0,64	0,00	0,01
B1XEL5_ECODH	*mpl*	UDP-N-acetylmuramate:L-alanyl-gamma-D-glutamyl-me	19,037	49874,117	5,531	6		785866	746892	8,7	8,2	0,95	0,42	0,49
B1XCF8_ECODH	*mppA*	Murein tripeptide (L-ala-gamma-D-glutamyl-meso-DAP)	5,959	59900,207	8,445	2		224235	114372	8,0	8,5	0,51	0,00	0,00
B1X743_ECODH	*mrcA*	Fused penicillin-binding protein 1a: murein transglycosy	9,882	93636,172	6,155	6		313149	330633	7,4	6,3	1,06	0,31	0,38

B1XCC4_ECODH	*mrcB*	Bifunctional glycosyl transferase and transpeptidase	8,886	94292,555	9,1	5		313003	222825	10,7	7,2	0,71	0,00	0,01
B1XHM1_ECODH	*mreB*	Cell wall structural complex MreBCD, actin-like compon	40,634	36952,402	5,192	11		6796024	7862187	11,1	3,3	1,16	0,05	0,07
B1X7K3_ECODH	*mrp*	Antiporter inner membrane protein	18,97	39938,078	5,854	4		683796	718013	7,1	14,3	1,05	0,56	0,63
B1X854_ECODH	*msbA*	Fused lipid transporter subunits of ABC superfamily: me	15,979	64460,707	8,62	7		814459	773484	13,0	8,8	0,95	0,52	0,59
B1XEK7_ECODH	*mscS*	Mechanosensitive channel	22,028	30896,021	7,896	5		1138295	3494434	14,9	1,3	3,07	0,00	0,00
MSRB_ECODH	*msrB*	Peptide methionine sulfoxide reductase MsrB	37,226	15451,175	5,581	3		690558	1034136	6,1	9,3	1,50	0,00	0,00
B1X9G5_ECODH	*msyB*	Uncharacterized protein	50	14259,198	3,587	3		156594	410757	27,2	10,7	2,62	0,00	0,00
B1X8L3_ECODH	*mtlA*	Fused mannitol-specific PTS enzymes: IIA components	11,774	67972,258	6,047	4		238941	469707	19,1	5,2	1,97	0,00	0,00
MTLD_ECODH	*mtlD*	Mannitol-1-phosphate 5-dehydrogenase	25,131	41139,016	5,357	6		496198	690396	13,9	10,6	1,39	0,01	0,02
MTNN_ECODH	*mtnN*	5'-methylthioadenosine/S-adenosylhomocysteine nuclec	15,086	24353,967	5,092	2		416729	1059837	31,3	11,2	2,54	0,00	0,00
MUG_ECODH	*mug*	G/U mismatch-specific DNA glycosylase	35,119	18673,393	9,169	4		132001	374641	6,0	7,8	2,84	0,00	0,00
MUKB_ECODH	*mukB*	Chromosome partition protein MukB	15,007	170230,17	5,237	18		2379563	7235578	7,4	14,8	3,04	0,00	0,00
B1X8M7_ECODH	*mukE*	Chromosome partition protein MukE	32	25984,404	4,974	5		611704	506289	8,1	12,2	0,83	0,04	0,06
MUKF_ECODH	*mukF*	Chromosome partition protein MukF	9,773	50578,91	4,752	3		184186	154253	19,5	20,1	0,84	0,28	0,34
B1XBX9_ECODH	*murB*	UDP-N-acetylenolpyruvoylglucosamine reductase	11,404	37851,031	5,779	3		193427	214867	17,9	8,2	1,11	0,30	0,37
MURC_ECODH	*murC*	UDP-N-acetylmuramate--L-alanine ligase	12,22	53625,961	5,529	3		225879	286308	18,1	4,9	1,27	0,05	0,08
B1XC65_ECODH	*murD*	UDP-N-acetylmuramoylalanine--D-glutamate ligase	8,447	46973,621	5,153	3		153651	206814	7,6	13,8	1,35	0,01	0,02
B1XC62_ECODH	*murE*	UDP-N-acetylmuramoyl-L-alanyl-D-glutamate--2,6-diam	16,162	53343,598	5,425	6		541420	687069	13,9	2,4	1,27	0,02	0,03
B1XC63_ECODH	*murF*	UDP-N-acetylmuramoyl-tripeptide--D-alanyl-D-alanine li	4,867	47447,148	5,253	2		122241	143347	14,1	8,5	1,17	0,11	0,15
MUTS_ECODH	*mutS*	DNA mismatch repair protein MutS	15,592	95246,898	5,394	9		645472	582285	7,3	7,1	0,90	0,09	0,13
B1XC76_ECODH	*mutT*	Nucleoside triphosphate pyrophosphohydrolase, marke	10,078	14927,073	5,012	1		29640	57199	19,4	4,8	1,93	0,00	0,00
NADE_ECODH	*nadE*	NH(3)-dependent NAD(+) synthetase	44,727	30636,834	5,413	11		2225754	4055278	5,8	8,0	1,82	0,00	0,00
B1XFK0_ECODH	*nadR*	NadR transcriptional repressor / ribosylnicotinamide kin	15,61	47345,832	5,423	4		244108	251557	17,0	15,1	1,03	0,80	0,84
B1X6L0_ECODH	*nagA*	N-acetylglucosamine-6-phosphate deacetylase	8,115	40949,098	5,645	2		308686	234536	13,4	6,9	0,76	0,01	0,03
NAGB_ECODH	*nagB*	Glucosamine-6-phosphate deaminase	8,647	29774,203	6,411	2		577102	253099	11,8	18,6	0,44	0,00	0,00
B1X6K8_ECODH	*nagD*	UMP phosphatase	35,6	27189,133	5,067	5		801032	1036175	5,6	5,8	1,29	0,00	0,00
B1X6L2_ECODH	*nagE*	Fused N-acetyl glucosamine specific PTS enzyme: IIC,	17,13	68346,891	5,784	7		2522252	781251	11,7	12,1	0,31	0,00	0,00
NAGZ_ECODH	*nagZ*	Beta-hexosaminidase	8,504	37594,73	5,863	2		65382	126681	32,6	8,6	1,94	0,01	0,02
NANA_ECODH	*nanA*	N-acetylneuraminate lyase	44,108	32593,469	5,61	9		13245153	1589781	9,6	15,1	0,12	0,00	0,00
NANE_ECODH	*nanE*	Putative N-acetylmannosamine-6-phosphate 2-epimera	22,707	24073,66	4,892	3		1000090	211334	13,8	9,8	0,21	0,00	0,00
NANK_ECODH	*nanK*	N-acetylmannosamine kinase	32,302	29643,865	5,626	7		780866	210877	5,8	14,8	0,27	0,00	0,00
NAPA_ECODH	*napA*	Periplasmic nitrate reductase	17,874	93042,023	8,234	9		1218982	199084	5,8	12,7	0,16	0,00	0,00
B1XAR8_ECODH	*narG*	Nitrate reductase 1, alpha subunit	38,893	140489,39	6,052	33		17465338	832102	9,3	12,1	0,05	0,00	0,00
B1XAR9_ECODH	*narH*	Nitrate reductase 1, beta (Fe-S) subunit	38,672	58066,41	6,362	14		5043912	203066	8,3	17,0	0,04	0,00	0,00
B1XAS1_ECODH	*narI*	Nitrate reductase 1, gamma (Cytochrome b(NR)) subur	10,222	25497,365	9,774	2		177890	5285	11,4	58,0	0,03	0,00	0,00
B1XAS0_ECODH	*narJ*	Molybdenum-cofactor-assembly chaperone subunit (De	36,441	26448,865	4,757	5		598401	60005	10,4	40,9	0,10	0,00	0,00
B1XAR7_ECODH	*narK*	Nitrate/nitrite transporter	7,127	49693,434	9,679	2		1878792	131666	12,6	164,5	0,07	0,01	0,02
B1XAR5_ECODH	*narL*	DNA-binding response regulator in two-component regu	12,037	23926,791	5,727	3		273107	609396	11,2	17,7	2,23	0,00	0,00
B1X889_ECODH	*narP*	DNA-binding response regulator in two-component regu	18,14	23574,975	4,816	3		309355	281894	20,6	15,1	0,91	0,52	0,58
B1XA19_ECODH	*ndh*	Respiratory NADH dehydrogenase 2/cupric reductase	42,166	47358,672	8,961	12		5203853	4176752	8,5	5,0	0,80	0,00	0,01
NDK_ECODH	*ndk*	Nucleoside diphosphate kinase	64,336	15463,444	5,54	7		5048926	1547299	12,0	8,9	0,31	0,00	0,00
B1XFW1_ECODH	*nemA*	N-ethylmaleimide reductase, FMN-linked	15,89	39516,414	5,803	4		151593	271314	24,0	17,7	1,79	0,01	0,02
B1X5X3_ECODH	*nfnB*	Dihydropteridine reductase, NAD(P)H-dependent, oxyge	37,327	23905,191	5,802	6		2242933	3944031	7,8	11,0	1,76	0,00	0,00
B1X7Z2_ECODH	*nfsA*	Nitroreductase A, NADPH-dependent, FMN-dependent	31,667	26800,686	6,444	6		1052466	1746893	23,6	3,1	1,66	0,01	0,01
NFUA_ECODH	*nfuA*	Fe/S biogenesis protein NfuA	26,702	20997,705	4,524	4		3199824	6631058	15,4	8,9	2,07	0,00	0,00
B1X7T4_ECODH	*nikA*	Nickel transporter subunit periplasmic-binding compone	13,931	58719,02	5,745	5		397946	44150	13,9	22,8	0,11	0,00	0,00
B1X713_ECODH	*nirB*	Nitrite reductase, large subunit, NAD(P)H-binding	12,869	93121,32	5,79	7		1524584	156914	4,2	21,9	0,10	0,00	0,00
B1X714_ECODH	*nirD*	Nitrite reductase, NAD(P)H-binding, small subunit	13,889	12284,003	5,074	1		317652	5710	7,0	20,0	0,02	0,00	0,00
B1XAE9_ECODH	*nlpB*	Outer membrane protein assembly factor BamC	56,395	36842,43	5,337	12		2707307	3694871	12,1	6,1	1,36	0,00	0,01
B1XCR8_ECODH	*nlpD*	Predicted outer membrane lipoprotein	17,15	40149	9,518	3		409387	732479	13,5	11,2	1,79	0,00	0,00
B1XD62_ECODH	*nlpE*	Lipoprotein involved with copper homeostasis and adhe	70,763	25844,287	5	11		3091263	651626090	7,1	4,6	210,80	0,00	0,00
B1X8C8_ECODH	*nrdA*	Ribonucleoside-diphosphate reductase	31,012	85775,281	5,791	18		6917555	6137767	3,0	4,2	0,89	0,00	0,01
B1X8C9_ECODH	*nrdB*	Ribonucleoside diphosphate reductase 1, beta subunit,	21,277	43517,273	4,691	7		2120658	2111088	11,4	5,0	1,00	0,95	0,96
B1XEL9_ECODH	*nrdD*	Anaerobic ribonucleoside-triphosphate reductase	12,079	80022,562	6,376	6		693274	88158	10,5	15,1	0,13	0,00	0,00
NRDR_ECODH	*nrdR*	Transcriptional repressor NrdR	16,107	17228,895	7,664	2		224062	231932	25,5	7,9	1,04	0,80	0,84
NUDC_ECODH	*nudC*	NADH pyrophosphatase	23,346	29688,939	5,518	4		181713	209502	4,0	4,7	1,15	0,00	0,01
B1X744_ECODH	*nudE*	ADP-ribose diphosphatase	13,978	21153,166	4,847	1	metabolism	42646	47413	20,6	15,2	1,11	0,47	0,54
NUDK_ECODH	*nudK*	GDP-mannose pyrophosphatase NudK	14,136	21748,664	4,879	1		8677	36837	32,7	17,9	4,25	0,00	0,00
NUOA_ECODH	*nuoA*	NADH-quinone oxidoreductase subunit A	17,687	16457,328	9,874	2		844903	976199	14,5	7,3	1,16	0,12	0,16
NUOB_ECODH	*nuoB*	NADH-quinone oxidoreductase subunit B	30,455	25055,816	5,587	7		2379764	1646128	8,4	8,2	0,69	0,00	0,00
NUOCD_ECODH	*nuoC*	NADH-quinone oxidoreductase subunit C/D	44,833	68694,648	5,98	21		9056337	5053498	5,8	4,6	0,56	0,00	0,00
B1X8Z7_ECODH	*nuoE*	NADH:ubiquinone oxidoreductase, chain E	19,277	18590,051	5,402	2		768682	483674	10,0	14,8	0,63	0,00	0,00
B1X8Z6_ECODH	*nuoF*	NADH:ubiquinone oxidoreductase, chain F	32,36	49292,453	6,441	10		2813394	1860774	14,5	7,1	0,66	0,00	0,01
B1X8Z5_ECODH	*nuoG*	NADH:ubiquinone oxidoreductase, chain G	35,714	100543,56	5,845	22		8981863	4555283	5,8	4,4	0,51	0,00	0,00
NUOH_ECODH	*nuoH*	NADH-quinone oxidoreductase subunit H	9,538	36219,152	6,898	3		825798	449545	17,4	5,3	0,54	0,00	0,00
B1X8Z3_ECODH	*nuoI*	NADH-quinone oxidoreductase subunit I	15	20537,75	5,396	3		478394	385912	11,4	13,6	0,81	0,06	0,09
NUOK_ECODH	*nuoK*	NADH-quinone oxidoreductase subunit K	12	10845,019	8,381	1		283364	115926	19,3	7,2	0,41	0,00	0,00
B1X8Z0_ECODH	*nuoL*	NADH:ubiquinone oxidoreductase, membrane subunit L	4,568	66438,078	8,283	2		654536	307117	22,9	7,4	0,47	0,00	0,00
B1X8Y9_ECODH	*nuoM*	NADH:ubiquinone oxidoreductase, membrane subunit M	3,143	56524,543	8,62	2		306885	254013	20,3	6,6	0,83	0,11	0,15
B1X9R4_ECODH	*nupC*	Nucleoside (Except guanosine) transporter	10,5	43475,754	8,5	3		332252	424087	32,3	10,4	1,28	0,17	0,22
B1XGY1_ECODH	*nusA*	Transcription termination/antitermination L factor	44,242	54870,922	4,527	15		7332866	7781016	6,9	1,9	1,06	0,14	0,19
NUSB_ECODH	*nusB*	N utilization substance protein B homolog	37,41	15689,056	6,601	4		1757353	1222035	3,7	3,4	0,70	0,00	0,00
B1XBY4_ECODH	*nusG*	Transcription termination/antitermination protein NusG	47,514	20531,514	6,339	6		3295984	2869576	4,6	6,5	0,87	0,01	0,03
OBG_ECODH	*obg*	GTPase Obg	25,641	43285,863	4,743	7		1721199	1139941	12,0	6,2	0,66	0,00	0,00

B1X8R1_ECODH	*ompA*	Outer membrane protein A (3aII*Gd)	61,272	37200,762	5,989	14		117862205	169928876	8,9	2,0	1,44	0,00	0,00
B1X8B1_ECODH	*ompC*	Outer membrane porin protein C	80,109	40368,117	4,581	19		41404866	55973304	16,3	13,0	1,35	0,03	0,05
B1X8N3_ECODH	*ompF*	Outer membrane porin 1a (IabF)	30,663	39333,309	4,76	8		3711984	3378989	14,1	9,1	0,91	0,28	0,35
B1X752_ECODH	*ompR*	DNA-binding response regulator in two-component regu	46,862	27353,617	6,038	7		2087056	2333010	10,6	6,5	1,12	0,12	0,17
B1XGF9_ECODH	*ompT*	DLP12 prophage outer membrane protease VII (Outer r	66,562	35562,309	5,755	15		9553423	19007479	8,4	3,9	1,99	0,00	0,00
B1XBK5_ECODH	*ompW*	Outer membrane protein W	10,849	22927,83	6,029	2		1090365	204377	13,9	25,6	0,19	0,00	0,00
B1X7E4_ECODH	*ompX*	Outer membrane protein	46,784	18602,617	6,561	6		22553305	29816976	6,9	4,3	1,32	0,00	0,00
B1XAT3_ECODH	*oppA*	Oligopeptide transporter periplasmic subunit	50,092	60948,004	6,053	17		9501322	14915890	8,2	9,7	1,57	0,00	0,00
B1XAT4_ECODH	*oppB*	Oligopeptide transporter subunit membrane component	8,17	33443,199	9,783	2		95408	1929	17,8	75,1	0,02	0,00	0,00
B1XAT6_ECODH	*oppD*	Oligopeptide transporter subunit ATP-binding componer	19,288	37188,43	5,777	4		286261	26372	5,4	23,9	0,09	0,00	0,00
B1XAT7_ECODH	*oppF*	ATP-binding subunit of oligopeptide ABC transporter	37,725	37181,547	7,647	11		1050577	157701	14,3	17,0	0,15	0,00	0,00
B1XDR6_ECODH	*orn*	Oligoribonuclease	16,575	20815,623	4,963	3		331221	290030	3,8	8,2	0,88	0,03	0,05
B1XE73_ECODH	*osmC*	Osmotically inducible, stress-inducible membrane prote	40,559	15088,142	5,57	4		2086352	2410040	4,3	4,7	1,16	0,00	0,01
B1XFI5_ECODH	*osmY*	Periplasmic protein	17,91	21073,707	6,315	3		171276	993216	19,0	10,6	5,80	0,00	0,00
B1XBX2_ECODH	*oxyR*	DNA-binding transcriptional dual regulator	16,393	34275,93	5,959	3		373488	610174	18,3	12,4	1,63	0,00	0,01
B1X6S2_ECODH	*pal*	Peptidoglycan-associated outer membrane lipoprotein	32,37	18824,186	6,292	4		1890773	3335965	25,8	17,3	1,76	0,02	0,03
PANB_ECODH	*panB*	3-methyl-2-oxobutanoate hydroxymethyltransferase	46,591	28237,439	5,148	7		1687580	1952585	1,4	12,4	1,16	0,05	0,08
PANC_ECODH	*panC*	Pantothenate synthetase	9,187	31597,672	5,915	2		445392	446335	21,8	19,3	1,00	0,99	0,99
B1XFH0_ECODH	*parC*	DNA topoisomerase 4 subunit A	19,149	83831,266	6,243	8		882540	813377	3,5	9,6	0,92	0,15	0,20
B1XG35_ECODH	*parE*	DNA topoisomerase 4 subunit B	17,302	70243,914	5,436	8		787285	910158	23,5	3,3	1,16	0,24	0,30
PCKA_ECODH	*pckA*	Phosphoenolpyruvate carboxykinase [ATP]	40,37	59643,48	5,459	15		17360214	6017663	5,2	1,8	0,35	0,00	0,00
PIMT_ECODH	*pcm*	Protein-L-isoaspartate O-methyltransferase	33,173	23257,775	6,528	5		237444	361950	5,4	6,9	1,52	0,00	0,00
B1XCB8_ECODH	*pcnB*	Poly(A) polymerase I	22,151	53870,91	9,669	8		807265	788018	7,6	5,7	0,98	0,63	0,69
B1XC88_ECODH	*pdhR*	DNA-binding transcriptional dual regulator	22,441	29425,471	6,036	4		243095	203201	8,4	7,0	0,84	0,02	0,03
PDXA_ECODH	*pdxA*	4-hydroxythreonine-4-phosphate dehydrogenase	12,766	35113,777	5,869	2		106515	138120	17,8	17,2	1,30	0,08	0,12
PDXB_ECODH	*pdxB*	Erythronate-4-phosphate dehydrogenase	36,508	41367,648	6,227	10		1571922	1649779	7,8	3,3	1,05	0,29	0,36
PDXH_ECODH	*pdxH*	Pyridoxine/pyridoxamine 5'-phosphate oxidase	29,817	25545,152	9,182	5		554655	488064	12,0	8,3	0,88	0,14	0,19
B1XB38_ECODH	*pdxJ*	Pyridoxine 5'-phosphate synthase	15,226	26384,287	5,61	3		352999	743136	14,3	7,8	2,11	0,00	0,00
PDXK_ECODH	*pdxK*	Pyridoxine kinase	20,141	30847,4	5,141	4		262623	267733	25,5	1,9	1,02	0,90	0,92
B1XFU8_ECODH	*pdxY*	Pyridoxamine kinase	19,861	31322,197	6,041	4		324944	445751	18,8	6,1	1,37	0,02	0,04
AMPA_ECODH	*pepA*	Probable cytosol aminopeptidase	28,032	54879,809	6,815	11		1643993	2552682	13,9	6,2	1,55	0,00	0,00
PEPB_ECODH	*pepB*	Peptidase B	53,396	46180,168	5,602	17		4347923	5331113	1,6	5,5	1,23	0,00	0,00
B1XDY0_ECODH	*pepD*	Aminoacyl-histidine dipeptidase (Peptidase D)	26,392	52915,406	5,204	10		5169246	6028542	7,8	4,7	1,17	0,01	0,03
B1X8N6_ECODH	*pepN*	Aminopeptidase N	18,736	98918,938	5,142	14		5335769	4843245	13,8	8,2	0,91	0,26	0,33
B1XEJ4_ECODH	*pepP*	Proline aminopeptidase P II	25,17	49815,434	5,245	8		967898	1069083	9,3	23,8	1,10	0,44	0,51
PEPQ_ECODH	*pepQ*	Xaa-Pro dipeptidase	32,731	50176,176	5,602	11		3527535	3588602	4,0	5,9	1,02	0,65	0,71
PEPT_ECODH	*pepT*	Peptidase T	19,608	44923,426	5,36	7		1263556	543648	4,3	15,7	0,43	0,00	0,00
K6PF_ECODH	*pfkA*	6-phosphofructokinase	21,25	34842,039	5,47	6		15577497	14573116	11,4	3,8	0,94	0,31	0,37
B1XGI4_ECODH	*pfkB*	Phosphofructokinase	32,362	32455,992	5,252	7		1113787	2006033	7,8	4,6	1,80	0,00	0,00
B1X842_ECODH	*pflA*	Pyruvate formate lyase activating enzyme 1	11,382	28204,291	6,002	3		153239	222096	37,3	12,2	1,45	0,11	0,16
B1X843_ECODH	*pflB*	Pyruvate formate lyase I	59,605	85357,203	5,69	29		70304609	24749122	9,0	2,7	0,35	0,00	0,00
G6PI_ECODH	*pgi*	Glucose-6-phosphate isomerase	37,705	61529,766	5,853	16		10557213	9816012	3,5	3,9	0,93	0,03	0,05
B1XEK9_ECODH	*pgk*	Phosphoglycerate kinase	59,948	41118,219	5,079	15		46055121	81556797	5,0	7,4	1,77	0,00	0,00
6PGL_ECODH	*pgl*	6-phosphogluconolactonase	34,743	36307,637	5,06	7		3134457	5425306	15,4	9,6	1,73	0,00	0,00
B1X6M1_ECODH	*pgm*	Phosphoglucomutase	40,293	58360,949	5,429	15		3469062	3728037	5,7	4,0	1,07	0,08	0,12
SYFA_ECODH	*pheS*	Phenylalanine--tRNA ligase alpha subunit	36,697	36831,805	5,792	10		4256088	3048053	4,0	4,1	0,72	0,00	0,00
B1XG20_ECODH	*pheT*	Phenylalanine--tRNA ligase beta subunit	44,403	87378,109	5,169	26		12908867	8777993	1,2	2,3	0,68	0,00	0,00
B1XCZ1_ECODH	*phnA*	Conserved protein	25,225	12345,049	4,971	2		224617	585839	27,7	14,6	2,61	0,00	0,00
B1XCZ0_ECODH	*phnB*	Conserved protein	10,884	16171,316	5,037	1		37455	34221	16,4	13,0	0,91	0,42	0,49
B1XA40_ECODH	*phoP*	DNA-binding response regulator in two-component regu	43,049	25535,217	5,098	7		1210074	1202943	8,5	5,0	0,99	0,91	0,93
B1X7U8_ECODH	*pitA*	Phosphate transporter, low-affinity	8,016	53389,027	9,533	3		403122	262854	27,1	11,5	0,65	0,03	0,05
B1XAI6_ECODH	*pldA*	Outer membrane phospholipase A	39,1	33163,133	5,154	9		618931	636469	14,8	2,1	1,03	0,72	0,77
PLSB_ECODH	*plsB*	Glycerol-3-phosphate acyltransferase	36,307	91381,445	8,512	20		2187624	2257627	8,0	4,5	1,03	0,52	0,58
B1XFG9_ECODH	*plsC*	1-acyl-sn-glycerol-3-phosphate acyltransferase	7,755	27453,158	9,655	2		140152	113974	15,8	18,0	0,81	0,12	0,16
B1XEL7_ECODH	*pmbA*	Predicted peptidase required for the maturation and sec	33,556	48369,637	5,401	10		2090594	2262588	7,7	6,2	1,08	0,16	0,21
B1X8X3_ECODH	*pmrD*	Polymyxin resistance protein B	15,909	9870,51	8,641	1		85605	80732	13,1	14,3	0,94	0,57	0,63
PNCB_ECODH	*pncB*	Nicotinate phosphoribosyltransferase	17,5	45897,285	6,208	6		273929	293072	13,2	7,1	1,07	0,42	0,49
PNP_ECODH	*pnp*	Polyribonucleotide nucleotidyltransferase	41,069	77100,977	5,114	20		13143388	13078620	8,3	4,2	1,00	0,92	0,93
B1XF68_ECODH	*pntA*	NAD(P) transhydrogenase subunit alpha	11,961	54623,441	5,645	5		1110482	1235256	14,9	8,9	1,11	0,25	0,31
B1XF67_ECODH	*pntB*	NAD(P) transhydrogenase subunit beta	20,13	48723,027	5,722	6		1114896	1307397	6,3	2,2	1,17	0,00	0,01
B1XAM0_ECODH	*polA*	DNA polymerase I	27,909	103118,13	5,395	18		2287821	1949886	5,4	4,9	0,85	0,00	0,01
B1XA36_ECODH	*potA*	Polyamine transporter subunit ATP-binding component	28,307	43028,215	5,19	7		1379368	1223180	4,3	6,5	0,89	0,02	0,04
B1XA35_ECODH	*potB*	Polyamine transporter subunit membrane component of	7,273	31061,982	9,437	2		100264	122328	27,4	10,0	1,22	0,19	0,25
B1XA33_ECODH	*potD*	Putrescine-binding periplasmic protein	22,414	38867,082	5,242	6		707094	841515	24,0	7,9	1,19	0,22	0,28
B1X812_ECODH	*poxB*	Pyruvate dehydrogenase (Pyruvate oxidase), thiamin-de	26,049	62011,387	5,861	10		839473	1209031	7,3	7,2	1,44	0,00	0,00
B1XDX5_ECODH	*ppa*	Inorganic pyrophosphatase	16,477	19703,557	5,027	3		4414666	6708263	33,1	8,0	1,52	0,04	0,06
CAPP_ECODH	*ppc*	Phosphoenolpyruvate carboxylase	36,693	99062,609	5,517	25		3627899	7597526	7,5	3,9	2,09	0,00	0,00
B1X711_ECODH	*ppiA*	Peptidyl-prolyl cis-trans isomerase	27,368	20431,322	8,804	3	degradation/folding	336405	195818	13,7	15,0	0,58	0,00	0,01
B1XGC3_ECODH	*ppiB*	Peptidyl-prolyl cis-trans isomerase	21,341	18153,471	5,514	3		2971103	4353321	9,9	5,4	1,47	0,00	0,00
B1X9Z1_ECODH	*ppiC*	Peptidyl-prolyl cis-trans isomerase C (Rotamase C)	21,505	10232,021	9,23	2		157522	139467	12,0	35,6	0,89	0,52	0,58
B1XFM9_ECODH	*ppiD*	Peptidyl-prolyl cis-trans isomerase (Rotamase D)	34,831	68149,859	4,939	14		4421334	4377317	11,8	7,2	0,99	0,89	0,91
B1XAX6_ECODH	*ppk*	Polyphosphate kinase	20,203	80431,508	8,96	10		439193	542209	9,8	1,4	1,23	0,00	0,01
B1XG09_ECODH	*pps*	Phosphoenolpyruvate synthase	15,909	87435,078	4,934	11		1626461	460387	6,3	7,8	0,28	0,00	0,00
PSRP_ECODH	*ppsR*	Phosphoenolpyruvate synthase regulatory protein	12,635	31210,867	5,987	3		211555	182920	12,3	6,4	0,86	0,09	0,14

B1XAX7_ECODH	*ppx*	Exopolyphosphatase	10,916	58135,969	6,646	4		291790	329897	10,4	9,9	1,13	0,15	0,20
B1XH98_ECODH	*prc*	Carboxy-terminal protease for penicillin-binding protein	17,742	76663,094	6,19	9		1139784	1050450	7,5	2,8	0,92	0,09	0,13
RF1_ECODH	*prfA*	Peptide chain release factor 1	19,444	40517,301	5,146	5		355451	524677	16,1	26,5	1,48	0,03	0,06
RF2_ECODH	*prfB*	Peptide chain release factor 2	28,493	41250,727	4,643	7		4759150	3637099	9,1	5,3	0,76	0,00	0,01
RF3_ECODH	*prfC*	Peptide chain release factor 3	18,147	59574,082	5,655	7		1715892	1199724	13,9	4,7	0,70	0,00	0,01
B1X7V3_ECODH	*prlC*	Oligopeptidase A	39,265	77167,234	5,151	20		5002220	6696218	5,5	2,4	1,34	0,00	0,00
B1X9K3_ECODH	*prmB*	50S ribosomal protein L3 glutamine methyltransferase	15,161	35001,73	4,623	4		523053	835199	11,0	7,3	1,60	0,00	0,00
PROA_ECODH	*proA*	Gamma-glutamyl phosphate reductase	32,374	44630,055	5,418	10		1247561	1465827	8,7	8,9	1,17	0,04	0,06
PROB_ECODH	*proB*	Glutamate 5-kinase	24,251	39056,523	6,073	6		850950	812180	4,4	6,6	0,95	0,28	0,35
B1XEX5_ECODH	*proC*	Pyrroline-5-carboxylate reductase	21,933	28144,877	5,638	4		550892	788285	14,1	6,2	1,43	0,00	0,01
B1XCZ4_ECODH	*proP*	Proline/glycine betaine transporter	5,4	54845,816	6,763	2		92353	392872	25,5	8,6	4,25	0,00	0,00
PROQ_ECODH	*proQ*	ProP effector	47,414	25892,518	9,664	9		3290284	3268211	9,9	5,1	0,99	0,91	0,92
SYP_ECODH	*proS*	Proline--tRNA ligase	53,147	63692,602	5,122	21		15180981	13298420	4,2	2,0	0,88	0,00	0,00
B1XCL5_ECODH	*proX*	Glycine betaine transporter subunit periplasmic-binding	26,97	36022,547	5,903	5		1672522	2143275	16,8	26,8	1,28	0,21	0,26
B1XAP8_ECODH	*prsA*	Ribose-phosphate pyrophosphokinase	42,857	34218,27	5,23	9		8700154	6210642	9,2	3,4	0,71	0,00	0,00
PSD_ECODH	*psd*	Phosphatidylserine decarboxylase proenzyme	32,609	35934,414	5,51	6	metabolism	1465011	1094095	13,8	9,1	0,75	0,01	0,03
B1XEX3_ECODH	*psiF*	Conserved protein	13,208	11686,53	9,784	1		1145	95470	120,7	29,9	83,36	0,00	0,00
B1XCD3_ECODH	*pspA*	Regulatory protein for phage-shock-protein operon	19,82	25492,939	5,385	3		189134	477527	24,2	29,5	2,52	0,00	0,01
B1XCD7_ECODH	*pspE*	Thiosulfate:cyanide sulfurtransferase (Rhodanese)	18,269	11475,313	7,864	1		24266	919	31,4	89,3	0,04	0,02	0,04
B1XBR2_ECODH	*pssA*	Phosphatidylserine synthase (CDP-diacylglycerol-serine	34,368	52801,793	9,06	11		1916500	1561799	6,3	2,8	0,81	0,00	0,00
B1X9V4_ECODH	*pstB*	Phosphate transporter subunit ATP-binding component	15,564	29027,289	6,125	2		196127	316367	54,6	93,2	1,61	0,37	0,44
B1X9V7_ECODH	*pstS*	Phosphate-binding protein PstS	6,069	37023,922	8,386	2		105254	105053	23,4	16,1	1,00	0,99	0,99
B1X908_ECODH	*pta*	Phosphate acetyltransferase	41,457	77172,086	5,284	20		15409859	18146821	3,7	4,3	1,18	0,00	0,00
B1XDM7_ECODH	*ptrA*	Protease III	9,148	107708,48	5,782	7		205723	333913	1,1	6,3	1,62	0,00	0,00
B1XA11_ECODH	*ptsG*	Fused glucose-specific PTS enzymes: IIBcomponent II(	20,545	50676,551	8,765	7		2450790	1799822	5,8	7,5	0,73	0,00	0,00
B1XA86_ECODH	*ptsH*	Phosphohistidinoprotein-hexose phosphotransferase cc	15,294	9119,371	5,646	1		221870	242979	41,3	20,1	1,10	0,72	0,77
B1XA87_ECODH	*ptsI*	Phosphoenolpyruvate-protein phosphotransferase	46,957	63561,836	4,776	17		17930060	20021846	5,6	3,4	1,12	0,01	0,03
B1XHH9_ECODH	*ptsN*	Sugar-specific enzyme IIA component of PTS	26,38	17959,568	5,568	3		395882	383974	4,8	4,4	0,97	0,39	0,46
PURA_ECODH	*purA*	Adenylosuccinate synthetase	46,528	47344,949	5,316	14		17032532	9628802	8,9	4,0	0,57	0,00	0,00
B1XA41_ECODH	*purB*	Adenylosuccinate lyase	32,237	51542,805	5,675	11		3086773	2718281	8,2	3,7	0,88	0,03	0,05
PUR7_ECODH	*purC*	Phosphoribosylaminoimidazole-succinocarboxamide sy	46,835	26994,998	5,072	8		2024168	2180981	10,5	6,7	1,08	0,28	0,35
B1XC08_ECODH	*purD*	Phosphoribosylamine--glycine ligase	18,182	45940,359	4,957	6		427584	480511	7,3	12,0	1,12	0,14	0,19
B1XGC1_ECODH	*purE*	N5-carboxyaminoimidazole ribonucleotide mutase	23,669	17780,322	6,038	2		73630	92697	26,3	5,1	1,26	0,13	0,18
B1X923_ECODH	*purF*	Amidophosphoribosyltransferase	14,653	56488,055	5,325	5		316815	242761	15,0	5,7	0,77	0,02	0,03
PUR9_ECODH	*purH*	Bifunctional purine biosynthesis protein PurH	34,405	57329,207	5,526	13		1226762	1138500	0,9	2,6	0,93	0,00	0,01
B1XB32_ECODH	*purL*	Phosphoribosylformylglycinamidine synthase	20,849	141402,7	5,229	15		1230600	902119	2,5	5,2	0,73	0,00	0,00
B1XAS4_ECODH	*purU*	Formyltetrahydrofolate hydrolase	23,929	31934,717	6,496	4		1910106	1091088	90,4	3,5	0,57	0,30	0,37
B1X9D5_ECODH	*putA*	Multifunctional proline dehydrogenase, delta-1-pyrroline	24,848	143815,16	5,691	21		2927172	1155187	9,1	7,0	0,39	0,00	0,00
B1XHC2_ECODH	*pykA*	Pyruvate kinase	38,333	51357,145	6,239	13		5792844	3230702	4,1	3,0	0,56	0,00	0,00
B1XFY4_ECODH	*pykF*	Pyruvate kinase	50,851	50729,422	5,77	18		18087688	22101650	7,7	10,4	1,22	0,02	0,04
PYRB_ECODH	*pyrB*	Aspartate carbamoyltransferase	35,37	34427,359	6,116	7	metabolism	582300	534916	14,3	8,0	0,92	0,37	0,44
PYRC_ECODH	*pyrC*	Dihydroorotase	41,379	38827,383	5,771	8		1954888	1731526	12,1	5,2	0,89	0,11	0,16
PYRD_ECODH	*pyrD*	Dihydroorotate dehydrogenase (quinone)	19,345	36774,52	7,661	5		419778	201240	11,3	22,7	0,48	0,00	0,00
PYRE_ECODH	*pyrE*	Orotate phosphoribosyltransferase	13,615	23566,998	5,333	3		55255	506611	99,5	10,6	9,17	0,00	0,01
PYRF_ECODH	*pyrF*	Orotidine 5'-phosphate decarboxylase	37,551	26350,244	5,813	6		1108103	887368	7,6	6,6	0,80	0,00	0,01
PYRG_ECODH	*pyrG*	CTP synthase	26,422	60374,148	5,633	14		7928710	6712911	4,3	5,6	0,85	0,00	0,01
B1XD40_ECODH	*pyrH*	Uridylate kinase	19,087	25970,258	6,851	2		65029	150380	28,5	11,4	2,31	0,00	0,01
PYRI_ECODH	*pyrI*	Aspartate carbamoyltransferase regulatory chain	35,294	17120,631	6,895	4	metabolism	359136	349677	12,1	13,1	0,97	0,77	0,82
B1XCT9_ECODH	*qor*	Quinone oxidoreductase, NADPH-dependent	20,183	35172,188	8,351	4		233401	326559	9,0	8,2	1,40	0,00	0,00
QUEA_ECODH	*queA*	S-adenosylmethionine:tRNA ribosyltransferase-isomera	15,73	39430,844	5,089	3		574489	312421	16,3	16,2	0,54	0,00	0,00
QUEC_ECODH	*queC*	7-cyano-7-deazaguanine synthase	15,152	25514,172	5,682	3		358886	225389	59,8	13,5	0,63	0,14	0,19
QUEF_ECODH	*queF*	NADPH-dependent 7-cyano-7-deazaguanine reductase	17,021	32587,508	5,726	5		234366	337780	10,7	16,0	1,44	0,01	0,02
RBFA_ECODH	*rbfA*	Ribosome-binding factor A	37,594	15154,482	5,932	4		1077891	938042	11,7	5,2	0,87	0,07	0,11
B1X9X6_ECODH	*rbsA*	Fused D-ribose transporter subunits of ABC superfamily	15,369	55041,609	5,837	6		178225	515369	5,1	2,3	2,89	0,00	0,00
B1X9X8_ECODH	*rbsB*	D-ribose transporter subunit periplasmic-binding compo	61,824	30950,48	6,849	11		5483777	11712435	15,4	14,6	2,14	0,00	0,00
B1X9X7_ECODH	*rbsC*	D-ribose transporter subunit membrane component of /	13,707	33451,824	9,811	2		329142	487229	11,6	14,8	1,48	0,01	0,01
RBSD_ECODH	*rbsD*	D-ribose pyranase	23,022	15292,5	5,929	2		306806	643895	7,9	16,0	2,10	0,00	0,00
B1X9X9_ECODH	*rbsK*	Ribokinase	33,657	32290,518	4,987	6		544738	943324	10,2	7,1	1,73	0,00	0,00
B1X8B3_ECODH	*rcsB*	DNA-binding response regulator in two-component regu	16,667	23670,617	6,85	2		587711	584601	29,4	14,9	0,99	0,98	0,98
B1X8B2_ECODH	*rcsD*	Phosphotransfer intermediate protein in two-component	6,966	100372,17	5,056	5		182244	221911	12,4	12,0	1,22	0,06	0,09
B1XD66_ECODH	*rcsF*	Predicted outer membrane protein, signal	54,478	14163,468	9,352	4		752063	1213469	26,2	9,2	1,61	0,01	0,02
RDGC_ECODH	*rdgC*	Recombination-associated protein RdgC	34,983	33993,324	5,273	8		760525	1030281	5,8	11,9	1,35	0,00	0,01
B1XEH9_ECODH	*recJ*	SsDNA exonuclease, 5'--> 3'-specific	5,546	63388,832	5,388	2		146400	101516	19,5	18,0	0,69	0,03	0,05
RECR_ECODH	*recR*	Recombination protein RecR	24,876	21963	5,133	4		155811	159423	3,1	6,9	1,02	0,56	0,62
B1X9Z2_ECODH	*rep*	DNA helicase and single-stranded DNA-dependent ATF	5,349	77024,195	6,769	2		66499	33215	17,4	16,3	0,50	0,00	0,00
B1X954_ECODH	*rfaF*	ADP-heptose:LPS heptosyltransferase II	14,08	39042,133	7,148	3		180728	163246	9,7	11,8	0,90	0,24	0,30
B1X6X5_ECODH	*rfbA*	Glucose-1-phosphate thymidylyltransferase	38,225	32693,557	5,386	9		2755186	1974437	14,7	5,7	0,72	0,01	0,01
B1X6X7_ECODH	*rfbB*	dTDP-glucose 4,6-dehydratase	29,64	40558,332	5,475	8		2198932	1611315	10,5	6,7	0,73	0,00	0,01
B1X6X4_ECODH	*rfbC*	dTDP-4-deoxyrhamnose-3,5-epimerase	11,351	21270,111	5,476	2		327884	174028	8,0	18,5	0,53	0,00	0,00
B1X6X6_ECODH	*rfbD*	dTDP-4-dehydrorhamnose reductase subunit, NAD(P)-l	11,037	32694,137	5,543	3		475325	379120	26,1	12,5	0,80	0,19	0,24
B1XAG2_ECODH	*rffA*	dTDP-4-keto-6-deoxy-D-glucose aminotransferase	31,383	41901,031	5,759	7		665084	648937	7,1	8,1	0,98	0,67	0,73
B1XAF8_ECODH	*rffD*	UDP-N-acetyl-D-mannosamine dehydrogenase	10,238	45838,734	5,223	2		244700	441561	52,6	10,1	1,80	0,08	0,11
B1XAF7_ECODH	*rffE*	UDP-N-acetylglucosamine 2-epimerase	14,362	42244,594	5,92	3		253235	318648	9,4	9,4	1,26	0,01	0,03
B1XAF9_ECODH	*rffG*	dTDP-glucose 4,6-dehydratase	21,408	39753,848	5,628	5		593170	530774	10,0	3,5	0,89	0,09	0,13

B1XAG0_ECODH	*rffH*	Glucose-1-phosphate thymidylyltransferase	7,167	32734,406	5,317	2		102257	121830	9,3	12,9	1,19	0,08	0,12
RHLB_ECODH	*rhlB*	ATP-dependent RNA helicase RhlB	29,216	47125,918	7,29	9		1231780	1262943	10,6	8,9	1,03	0,73	0,78
B1X7C7_ECODH	*rhlE*	ATP-dependent RNA helicase RhlE	8,59	49989,316	10,057	3		864493	740545	23,9	11,7	0,86	0,27	0,34
B1X9Z7_ECODH	*rho*	Transcription termination factor Rho	55,131	47004,211	6,751	18		13544094	12136656	3,8	3,0	0,90	0,00	0,01
RIBA_ECODH	*ribA*	GTP cyclohydrolase-2	12,755	21835,979	5,596	3		157336	216160	14,1	20,8	1,37	0,06	0,08
RIBB_ECODH	*ribB*	3,4-dihydroxy-2-butanone 4-phosphate synthase	15,668	23353,467	4,898	3		1134658	923421	9,2	11,8	0,81	0,04	0,06
B1XFX2_ECODH	*ribC*	Riboflavin synthase, alpha subunit	34,742	23444,896	5,642	6		1073484	1171344	11,1	7,2	1,09	0,22	0,28
B1XF02_ECODH	*ribD*	Riboflavin biosynthesis protein RibD	14,714	40338,312	7,219	4		493399	515831	14,8	4,7	1,05	0,58	0,65
B1XBF0_ECODH	*ribF*	Bifunctional riboflavin kinase and FAD synthetase	22,364	34734,266	9,336	4		304553	209352	9,6	8,3	0,69	0,00	0,00
RISB_ECODH	*ribH*	6,7-dimethyl-8-ribityllumazine synthase	48,077	16156,511	5,155	5		4356575	5458756	14,2	18,3	1,25	0,09	0,13
B1X646_ECODH	*rihA*	Pyrimidine-specific ribonucleoside hydrolase RihA	8,039	33822,922	4,836	2		418068	137819	15,3	2,9	0,33	0,00	0,00
RIHC_ECODH	*rihC*	Non-specific ribonucleoside hydrolase RihC	21,053	32560,77	5,23	3	metabolism	180215	90422	30,1	25,3	0,50	0,01	0,03
RIMM_ECODH	*rimM*	Ribosome maturation factor RimM	43,956	20605,434	4,608	5		1874458	1275963	8,9	4,2	0,68	0,00	0,00
RIMO_ECODH	*rimO*	Ribosomal protein S12 methylthiotransferase RimO	18,367	49581,793	5,305	7		961022	789703	4,5	20,4	0,82	0,09	0,13
RIMP_ECODH	*rimP*	Ribosome maturation factor RimP	12	16650,975	4,597	2		206234	258323	19,1	16,1	1,25	0,13	0,18
B1XDT1_ECODH	*rlmB*	23S rRNA (guanosine-2'-O-)-methyltransferase RlmB	11,111	26556,629	6,174	2		231485	242522	2,4	1,8	1,05	0,02	0,04
RLME_ECODH	*rlmE*	Ribosomal RNA large subunit methyltransferase E	34,45	23335,076	9,435	4		458604	674697	5,0	4,5	1,47	0,00	0,00
RLMF_ECODH	*rlmF*	Ribosomal RNA large subunit methyltransferase F	24,026	34225,922	9,297	4		250260	153679	15,0	3,1	0,61	0,00	0,00
RLMG_ECODH	*rlmG*	Ribosomal RNA large subunit methyltransferase G	12,963	42331,449	6,318	3		162296	160819	4,5	4,2	0,99	0,78	0,82
RLMH_ECODH	*rlmH1*	Ribosomal RNA large subunit methyltransferase H	14,839	17341,111	8,709	2		95805	92515	24,4	9,1	0,97	0,80	0,84
RLMI_ECODH	*rlmI*	Ribosomal RNA large subunit methyltransferase I	14,394	44356,922	7,602	4		618760	614695	7,4	6,9	0,99	0,90	0,92
RLMKL_ECODH	*rlmL*	Ribosomal RNA large subunit methyltransferase K/L	16,382	78854,102	8,964	10		734700	637646	8,7	9,8	0,87	0,08	0,12
RLMM_ECODH	*rlmM*	Ribosomal RNA large subunit methyltransferase M	4,918	41905,199	7,038	2		115309	76030	13,4	27,8	0,66	0,05	0,07
RLMN_ECODH	*rlmN*	Dual-specificity RNA methyltransferase RlmN	12,5	43085,531	6,562	4		743327	371644	12,2	6,6	0,50	0,00	0,00
B1X628_ECODH	*rlpA*	Minor lipoprotein	16,022	37527,996	5,515	2		506182	807577	9,7	11,2	1,60	0,00	0,00
B1X636_ECODH	*rlpB*	LPS-assembly lipoprotein LptE	33,161	21356,531	8,73	5		608219	718956	16,6	7,5	1,18	0,11	0,15
B1XBL8_ECODH	*rluB*	Pseudouridine synthase	21,649	32711,385	10,029	6		894983	569243	6,3	5,3	0,64	0,00	0,00
B1X9J9_ECODH	*rluC*	Pseudouridine synthase	15,047	36026,574	9,85	5		339266	575190	30,2	11,0	1,70	0,02	0,03
B1X608_ECODH	*rna*	Ribonuclease I	11,567	29617,588	8,863	2		123934	142868	25,5	18,2	1,15	0,42	0,49
RNB_ECODH	*rnb*	Exoribonuclease 2	36,801	72490,781	5,402	17		3773601	2103976	4,1	23,7	0,56	0,00	0,01
RNC_ECODH	*rnc*	Ribonuclease 3	19,027	25550,043	6,404	5		654657	823170	10,1	4,8	1,26	0,01	0,01
B1X9J7_ECODH	*rne*	Ribonuclease E	28,087	118196,73	5,476	18		6379687	5999395	9,4	9,0	0,94	0,39	0,46
B1X607_ECODH	*rnk*	Regulator of nucleoside diphosphate kinase	48,529	14927,063	4,483	3		565329	359835	9,7	36,3	0,64	0,04	0,06
RNPA_ECODH	*rnpA*	Ribonuclease P protein component	22,689	13789,198	11,815	2		273953	250486	18,3	13,9	0,91	0,46	0,53
B1XDT0_ECODH	*rnr*	Ribonuclease R	18,696	92109,195	8,777	12		868030	1746731	21,6	7,7	2,01	0,00	0,00
B1XFW3_ECODH	*rnt*	Ribonuclease T	11,163	23522,695	5,187	2		134493	89925	14,7	10,8	0,67	0,01	0,01
RODZ_ECODH	*rodZ*	Cytoskeleton protein RodZ	19,585	36191,668	5,585	4		1359394	1782141	8,1	7,2	1,31	0,00	0,01
B1XD58_ECODH	*rof*	Modulator of Rho-dependent transcription termination	20,238	9479,644	4,628	1		153116	425533	7,7	11,0	2,78	0,00	0,00
B1X733_ECODH	*rpe*	Ribulose-phosphate 3-epimerase	19,111	24554,254	5,127	3		447075	704293	13,2	7,7	1,58	0,00	0,00
RPIA_ECODH	*rpiA*	Ribose-5-phosphate isomerase A	39,269	22860,395	5,204	5		2863507	4120283	16,2	8,3	1,44	0,01	0,01
RL1_ECODH	*rplA*	50S ribosomal protein L1	51,709	24729,635	9,644	9		64228539	57273684	7,6	3,5	0,89	0,03	0,05
RL2_ECODH	*rplB*	50S ribosomal protein L2	49,084	29860,443	10,931	11		43964925	34533534	14,9	4,1	0,79	0,02	0,03
RL3_ECODH	*rplC*	50S ribosomal protein L3	43,541	22243,516	9,905	7		37954226	32091782	15,7	4,8	0,85	0,09	0,13
RL4_ECODH	*rplD*	50S ribosomal protein L4	47,264	22086,529	9,724	8		37411476	34300756	9,3	5,5	0,92	0,16	0,21
RL5_ECODH	*rplE*	50S ribosomal protein L5	55,307	20301,572	9,491	8		39074845	25845042	4,0	9,6	0,66	0,00	0,00
RL6_ECODH	*rplF*	50S ribosomal protein L6	71,186	18903,783	9,71	9		44319846	38337180	12,0	4,1	0,87	0,07	0,11
RL9_ECODH	*rplI*	50S ribosomal protein L9	61,074	15769,064	6,165	9		50988485	42483841	11,0	2,9	0,83	0,02	0,03
RL10_ECODH	*rplJ*	50S ribosomal protein L10	50,909	17711,588	9,038	6		55896250	47712030	8,8	5,1	0,85	0,02	0,04
RL11_ECODH	*rplK*	50S ribosomal protein L11	59,155	14875,379	9,641	6		43922745	43345066	18,2	6,6	0,99	0,90	0,92
RL7_ECODH	*rplL*	50S ribosomal protein L7/L12	43,802	12295,196	4,603	4		25739150	22515021	8,4	6,6	0,87	0,05	0,07
RL13_ECODH	*rplM*	50S ribosomal protein L13	46,479	16018,543	9,905	5		28773198	25681567	9,7	6,6	0,89	0,10	0,14
RL14_ECODH	*rplN*	50S ribosomal protein L14	43,089	13541,023	10,431	4		24679780	25317444	4,6	5,8	1,03	0,52	0,58
RL15_ECODH	*rplO*	50S ribosomal protein L15	40,278	14980,424	11,185	5		39498758	32713160	12,1	8,6	0,83	0,04	0,07
RL16_ECODH	*rplP*	50S ribosomal protein L16	32,353	15281,202	11,225	3		34402625	25229253	11,4	4,5	0,73	0,00	0,01
RL17_ECODH	*rplQ*	50S ribosomal protein L17	25,984	14364,586	11,052	3		22908959	21399551	10,4	3,4	0,93	0,25	0,31
RL19_ECODH	*rplS*	50S ribosomal protein L19	47,826	13133,235	10,619	5		33850823	30452204	9,5	9,5	0,90	0,17	0,22
RL20_ECODH	*rplT*	50S ribosomal protein L20	22,881	13496,957	11,471	3		15058102	13185239	9,7	6,1	0,88	0,05	0,08
RL21_ECODH	*rplU*	50S ribosomal protein L21	51,456	11564,349	9,846	4		19469356	18887226	14,4	1,6	0,97	0,68	0,73
RL22_ECODH	*rplV*	50S ribosomal protein L22	56,364	12226,292	10,233	5		22803080	15569930	14,3	8,6	0,68	0,00	0,01
B1X6G9_ECODH	*rplW*	50S ribosomal protein L23	12	11199,123	9,935	1		7603	972	33,3	154,5	0,13	0,08	0,12
RL24_ECODH	*rplX*	50S ribosomal protein L24	36,538	11316,215	10,211	3		9594030	7707162	11,3	3,0	0,80	0,01	0,02
RL25_ECODH	*rplY*	50S ribosomal protein L25	56,383	10693,44	9,599	5		13011180	10172995	6,5	9,4	0,78	0,00	0,01
RL27_ECODH	*rpmA*	50S ribosomal protein L27	29,412	9124,472	10,585	2		228775	202090	7,0	30,0	0,88	0,52	0,59
RL28_ECODH	*rpmB*	50S ribosomal protein L28	21,795	9006,487	11,421	2		4181870	3389717	9,7	10,9	0,81	0,03	0,05
RL29_ECODH	*rpmC*	50S ribosomal protein L29	46,032	7273,449	9,984	2		30284618	21691556	5,4	7,0	0,72	0,00	0,00
RL30_ECODH	*rpmD*	50S ribosomal protein L30	33,898	6541,79	10,956	2		6426706	6063349	20,1	19,6	0,94	0,69	0,74
RL31_ECODH	*rpmE*	50S ribosomal protein L31	51,429	7871,062	9,461	3		11712955	13373750	11,1	14,3	1,14	0,20	0,26
RL32_ECODH	*rpmF*	50S ribosomal protein L32	26,316	6446,383	11,032	1		2627072	1708481	22,6	7,0	0,65	0,01	0,02
RL33_ECODH	*rpmG*	50S ribosomal protein L33	38,182	6371,585	10,248	2		4239245	4317356	16,4	14,1	1,02	0,87	0,90
RL34_ECODH	*rpmH*	50S ribosomal protein L34	17,391	5380,389	13,002	1		1896784	2028711	18,1	17,5	1,07	0,62	0,68
B1X6E7_ECODH	*rpoA*	DNA-directed RNA polymerase subunit alpha	51,976	36511,723	4,982	13		20783490	23791898	4,6	3,9	1,14	0,00	0,01
RPOB_ECODH	*rpoB*	DNA-directed RNA polymerase subunit beta	48,137	150632,34	5,148	43		54385226	49474789	2,3	2,6	0,91	0,00	0,00
RPOC_ECODH	*rpoC*	DNA-directed RNA polymerase subunit beta'	43,852	155160,25	6,667	44		45947836	40038327	8,7	1,9	0,87	0,02	0,04
B1XG72_ECODH	*rpoD*	RNA polymerase sigma factor RpoD	19,739	70263,281	4,69	10		1732538	2386436	14,8	9,2	1,38	0,01	0,02

B1XBQ0_ECODH	*rpoE*	RNA polymerase sigma factor	17,277	21695,738	5,382	2		123236	95639	32,2	13,0	0,78	0,19	0,25
B1XHH7_ECODH	*rpoN*	RNA polymerase sigma-54 factor	11,74	53989,785	4,627	4		194446	264139	6,9	16,4	1,36	0,01	0,03
B1XCR7_ECODH	*rpoS*	RNA polymerase sigma factor RpoS	12,424	37971,859	4,888	3		161979	334568	10,1	19,1	2,07	0,00	0,00
RPOZ_ECODH	*rpoZ*	DNA-directed RNA polymerase subunit omega	59,341	10236,573	4,87	4		2205921	2611819	3,6	4,7	1,18	0,00	0,00
B1X851_ECODH	*rpsA*	30S ribosomal protein S1	42,908	61158,074	4,892	17		50209639	45609465	7,0	3,3	0,91	0,05	0,07
RS2_ECODH	*rpsB*	30S ribosomal protein S2	70,539	26743,641	6,613	12		40152851	37005499	4,2	5,5	0,92	0,05	0,08
RS4_ECODH	*rpsD*	30S ribosomal protein S4	46,117	23469,094	10,052	8		45939708	45521232	8,3	5,8	0,99	0,86	0,89
B1X6F5_ECODH	*rpsE*	30S ribosomal protein S5	62,275	17603,379	10,109	7		73024084	61185305	11,5	6,4	0,84	0,03	0,06
RS6_ECODH	*rpsF*	30S ribosomal protein S6	67,176	15187,034	5,258	7		4084834	14471426	58,1	14,6	3,54	0,00	0,01
RS7_ECODH	*rpsG*	30S ribosomal protein S7	50,279	20019,092	10,365	7		62180848	48099447	12,5	6,0	0,77	0,01	0,02
RS8_ECODH	*rpsH*	30S ribosomal protein S8	47,692	14126,548	9,441	5		1494267	1096628	5,4	7,8	0,73	0,00	0,00
RS9_ECODH	*rpsI*	30S ribosomal protein S9	46,154	14856,199	10,944	6		26693006	20217356	8,1	5,4	0,76	0,00	0,00
RS10_ECODH	*rpsJ*	30S ribosomal protein S10	48,544	11735,587	9,681	5		20073888	16432276	9,8	5,4	0,82	0,01	0,03
RS11_ECODH	*rpsK*	30S ribosomal protein S11	27,132	13844,926	11,328	3		19873662	19729049	18,0	11,7	0,99	0,95	0,96
B1X6F0_ECODH	*rpsM*	30S ribosomal protein S13	61,864	13099,385	10,783	7		20150831	20006586	7,4	5,8	0,99	0,88	0,91
RS14_ECODH	*rpsN*	30S ribosomal protein S14	19,802	11580,483	11,163	2		369332	612714	34,8	19,2	1,66	0,04	0,06
RS15_ECODH	*rpsO*	30S ribosomal protein S15	15,73	10268,765	10,402	2		1614962	6949400	71,8	14,5	4,30	0,00	0,01
RS16_ECODH	*rpsP*	30S ribosomal protein S16	45,122	9190,556	10,543	3		10426181	8430336	12,6	9,0	0,81	0,03	0,05
RS18_ECODH	*rpsR*	30S ribosomal protein S18	29,333	8986,432	10,595	2		13843561	11126314	12,6	2,0	0,80	0,02	0,03
RS19_ECODH	*rpsS*	30S ribosomal protein S19	55,435	10430,286	10,524	4		3205338	7059455	38,5	22,9	2,20	0,01	0,02
RS20_ECODH	*rpsT*	30S ribosomal protein S20	24,138	9684,388	11,182	2		21840670	17884684	9,8	11,3	0,82	0,03	0,06
RS21_ECODH	*rpsU*	30S ribosomal protein S21	39,437	8499,957	11,15	3		5737306	12852502	45,3	6,7	2,24	0,01	0,02
RRAA_ECODH	*rraA*	Regulator of ribonuclease activity A	39,752	17360,365	4,071	4		609472	881297	11,4	7,2	1,45	0,00	0,00
B1XBP9_ECODH	*rseA*	Anti-sigma factor	12,963	24321,375	5,111	1		94341	51825	46,0	19,2	0,55	0,05	0,07
B1XD45_ECODH	*rseP*	Inner membrane zinc RIP metalloprotease	9,111	49071,324	6,628	3		161723	153016	10,4	18,8	0,95	0,62	0,68
RSGA_ECODH	*rsgA*	Putative ribosome biogenesis GTPase RsgA	12,286	39193,305	5,594	3		426203	468742	7,3	8,3	1,10	0,13	0,18
RSMA_ECODH	*rsmA*	Ribosomal RNA small subunit methyltransferase A	27,106	30420,139	7,822	5		448642	784041	21,8	7,3	1,75	0,00	0,01
RSMB_ECODH	*rsmB*	Ribosomal RNA small subunit methyltransferase B	33,566	48347,512	7,165	8		776497	778515	24,7	7,7	1,00	0,99	0,99
RSMC_ECODH	*rsmC*	Ribosomal RNA small subunit methyltransferase C	10,204	37624,73	5,999	3		289721	278475	6,3	11,6	0,96	0,56	0,63
RSMF_ECODH	*rsmF*	Ribosomal RNA small subunit methyltransferase F	8,559	53227,547	5,441	2		93870	76114	8,6	16,5	0,81	0,07	0,10
RSMG_ECODH	*rsmG*	Ribosomal RNA small subunit methyltransferase G	10,628	23431,119	6,062	1		35576	26768	13,8	24,1	0,75	0,10	0,15
RSMH_ECODH	*rsmH*	Ribosomal RNA small subunit methyltransferase H	18,53	34877,816	6,067	4		542936	542915	6,9	4,9	1,00	1,00	1,00
B1XF73_ECODH	*rstA*	DNA-binding response regulator in two-component regu	18,595	27048,248	5,418	4		153194	229290	12,0	9,9	1,50	0,00	0,01
B1X881_ECODH	*rsuA*	Pseudouridine synthase	12,554	25865,303	5,747	2		277809	300751	13,3	6,2	1,08	0,32	0,38
RSXC_ECODH	*rsxC*	Electron transport complex subunit RsxC	6,351	80171,547	8,884	3		54311	59923	24,8	8,0	1,10	0,45	0,52
B1XBP5_ECODH	*sapA*	Predicted antimicrobial peptide transporter subunit perip	5,667	61564,898	6,847	2		125212	88518	7,9	26,4	0,71	0,04	0,07
B1X6U7_ECODH	*sbcB*	Exodeoxyribonuclease I	5,263	54500,812	5,446	2		97400	42519	14,6	29,7	0,44	0,00	0,01
B1XEW6_ECODH	*sbmA*	Predicted transporter	7,143	46459,391	8,458	2	transport	34265	28056	8,2	12,3	0,82	0,04	0,07
B1X6U5_ECODH	*sbmC*	DNA gyrase inhibitor	10,828	18081,463	4,612	1		53327	55634	33,2	6,3	1,04	0,80	0,84
B1XH81_ECODH	*sdaA*	L-serine deaminase I	19,604	48906,645	5,178	7	metabolism	1562565	848217	9,4	10,6	0,54	0,00	0,00
B1XDK6_ECODH	*sdaB*	L-serine deaminase II	29,011	48752,926	5,509	9		4420011	2078878	9,6	17,8	0,47	0,00	0,00
B1XDK5_ECODH	*sdaC*	Predicted serine transporter	11,888	46906,301	9,209	4		1097326	628900	20,6	9,5	0,57	0,00	0,01
B1X6Q4_ECODH	*sdhA*	Succinate dehydrogenase, flavoprotein subunit	33,503	64421,844	5,849	13		10266339	3597073	5,1	4,9	0,35	0,00	0,00
B1X6Q5_ECODH	*sdhB*	Succinate dehydrogenase, FeS subunit	50,42	26769,863	6,314	10		2265945	1146713	23,1	5,3	0,51	0,00	0,00
SECA_ECODH	*secA*	Protein translocase subunit SecA	42,175	102022,99	5,431	26		7036238	15023361	2,6	6,9	2,14	0,00	0,00
SECB_ECODH	*secB*	Protein-export protein SecB	55,484	17277,361	4,265	5		8539453	9582938	4,5	5,8	1,12	0,02	0,04
B1XEZ6_ECODH	*secD*	Protein translocase subunit SecD	33,496	66632,148	8,621	15		2832914	3604471	10,6	5,5	1,27	0,01	0,02
B1XEZ7_ECODH	*secF*	Protein-export membrane protein SecF	12,074	35382,379	5,567	2		589297	502160	8,7	8,6	0,85	0,04	0,06
B1XGY5_ECODH	*secG*	Preprotein translocase membrane subunit	27,273	11365,157	6,091	2		2904428	2686314	9,5	12,3	0,92	0,33	0,40
B1X6F2_ECODH	*secY*	Protein translocase subunit SecY	16,253	48511,688	9,894	5		1980504	1503796	12,8	9,0	0,76	0,01	0,02
B1X8K6_ECODH	*selB*	Selenocysteinyl-tRNA-specific translation factor	17,427	68867,445	6,107	7		431678	524056	6,1	2,5	1,21	0,00	0,00
B1XGM5_ECODH	*selD*	Selenide, water dikinase	45,245	36687,254	5,299	9		3253475	2636916	9,7	2,9	0,81	0,01	0,01
B1X6M0_ECODH	*seqA*	Negative modulator of initiation of replication	30,387	20315,447	8,815	4		702313	811865	8,7	5,8	1,16	0,03	0,05
B1XEJ8_ECODH	*serA*	D-3-phosphoglycerate dehydrogenase	20	44175,777	5,921	6		289084	558145	13,8	10,3	1,93	0,00	0,00
B1XFJ8_ECODH	*serB*	3-phosphoserine phosphatase	10,559	35042,691	5,492	3		164011	170457	7,5	4,2	1,04	0,41	0,48
SERC_ECODH	*serC*	Phosphoserine aminotransferase	37,293	39783,309	5,369	11		2351280	4876124	6,6	4,1	2,07	0,00	0,00
SYS_ECODH	*serS*	Serine--tRNA ligase	34,651	48414,023	5,34	12		8339481	6726979	6,7	4,4	0,81	0,00	0,00
B1X9L3_ECODH	*sixA*	Phosphohistidine phosphatase	8,696	17207,773	4,454	2		81676	104285	14,8	19,7	1,28	0,11	0,15
B1XD47_ECODH	*skp*	Chaperone protein skp	22,36	17688,234	9,694	3		6459720	7649887	7,4	8,3	1,18	0,03	0,04
SLMA_ECODH	*slmA*	Nucleoid occlusion factor SlmA	16,162	22836,344	8,783	2		146629	117099	6,2	11,1	0,80	0,01	0,02
B1X7W1_ECODH	*slp*	Outer membrane lipoprotein	29,787	20963,914	6,822	3		6614950	6884565	9,1	2,9	1,04	0,43	0,50
B1XFK2_ECODH	*slt*	Lytic murein transglycosylase, soluble	23,256	73353,156	8,847	11	peptidoglycan-modification	399524	597968	8,2	35,6	1,50	0,05	0,07
B1XFV3_ECODH	*slyB*	Outer membrane lipoprotein	30,968	15601,666	9,356	3		6702449	6522584	18,5	9,0	0,97	0,79	0,83
B1X6J8_ECODH	*slyD*	Peptidyl-prolyl cis-trans isomerase	11,735	20852,832	4,856	2		2061134	2024403	8,3	8,4	0,98	0,77	0,82
SLYX_ECODH	*slyX*	Protein SlyX	38,889	8214,33	4,889	2		206106	234343	12,8	8,5	1,14	0,15	0,20
B1XBT7_ECODH	*smpA*	Outer membrane protein assembly factor BamE	17,699	12302,034	8,71	1		929886	1384853	12,7	9,9	1,49	0,00	0,01
B1XB75_ECODH	*sodA*	Superoxide dismutase	12,136	23097,098	6,444	2		1260496	955064	6,1	20,2	0,76	0,04	0,07
B1XFW7_ECODH	*sodB*	Superoxide dismutase	44,56	21265,779	5,581	5		3321777	5548114	33,5	10,4	1,67	0,03	0,04
B1XFV7_ECODH	*sodC*	Superoxide dismutase [Cu-Zn]	10,405	17680,977	5,947	1		#ZAHL!	3128	#DIV/0!	84,0	#NV	#NV	#NV
B1XGU2_ECODH	*sohA*	Predicted regulator	13,514	12358,941	5,109	1		60651	89216	5,2	10,7	1,47	0,00	0,00
B1XBM1_ECODH	*sohB*	Predicted inner membrane peptidase	12,034	39366,441	9,241	3		97908	172250	12,3	13,5	1,76	0,00	0,00
MTOX_ECODH	*solA*	N-methyl-L-tryptophan oxidase	23,925	40902,004	5,142	6		465987	570384	12,1	8,1	1,22	0,04	0,06
B1XFA0_ECODH	*speA*	Biosynthetic arginine decarboxylase	31,307	73898,445	4,832	13		2869198	2339543	7,6	7,3	0,82	0,01	0,02
SPEB_ECODH	*speB*	Agmatinase	25,49	33557,043	5,142	6		1621784	1022707	7,5	3,0	0,63	0,00	0,00

SPED_ECODH	*speD*	S-adenosylmethionine decarboxylase proenzyme	13,636	30384,643	5,19	2		210159	243112	11,9	12,6	1,16	0,15	0,20
SPEE_ECODH	*speE*	Spermidine synthase	23,611	32321,391	5,33	3		590995	724172	8,4	5,1	1,23	0,01	0,01
B1XF49_ECODH	*speG*	Spermidine N1-acetyltransferase	28,495	21887,014	6,201	4		626163	746613	10,4	10,2	1,19	0,05	0,08
B1X984_ECODH	*spoU*	tRNA (Guanosine-2'-O-)-methyltransferase	10,48	25343,074	6,706	2		115848	162260	10,3	8,7	1,40	0,00	0,01
B1XGM7_ECODH	*sppA*	Protease IV (Signal peptide peptidase)	19,256	67219,336	5,724	7		554022	498617	7,9	7,2	0,90	0,10	0,14
B1XGK4_ECODH	*spy*	Envelope stress induced periplasmic protein	38,509	18199,072	9,66	6	stress response	430101	354778	23,8	7,9	0,82	0,18	0,23
B1XCN1_ECODH	*srlB*	Glucitol/sorbitol-specific enzyme IIA component of PTS	51,22	13304,049	4,957	3		2633225	1419384	7,8	16,7	0,54	0,00	0,00
B1XCN2_ECODH	*srlD*	Sorbitol-6-phosphate dehydrogenase	58,301	27857,877	5,922	10		2952693	1633825	13,1	3,5	0,55	0,00	0,00
B1XCN0_ECODH	*srlE*	Glucitol/sorbitol-specific enzyme IIB component of PTS	17,555	33332,039	6,516	4		1214495	534716	3,0	4,4	0,44	0,00	0,00
B1XCN4_ECODH	*srlR*	DNA-bindng transcriptional repressor	8,949	28236,148	5,217	2		61065	37814	24,0	25,6	0,62	0,03	0,05
B1XBQ3_ECODH	*srmB*	ATP-dependent RNA helicase SrmB	27,928	49914,141	9,273	9		1597581	1436004	12,1	7,8	0,90	0,19	0,25
B1XCU6_ECODH	*ssb*	Single-stranded DNA-binding protein	16,854	18974,996	5,441	3		1492187	1435795	11,0	10,7	0,96	0,63	0,69
B1XDH7_ECODH	*sscR*	6-carboxy-5,6,7,8-tetrahydropterin synthase	15,702	13772,88	6,037	1		36786	27001	13,4	34,3	0,73	0,22	0,28
B1XAZ6_ECODH	*sseA*	3-mercaptopyruvate sulfurtransferase	23,132	30811,84	4,562	6		1486572	971190	16,4	3,7	0,65	0,00	0,01
B1XHK2_ECODH	*sspA*	Stringent starvation protein A	44,34	24304,908	5,221	7		4153052	3966583	9,4	11,1	0,96	0,56	0,62
B1XHK1_ECODH	*sspB*	ClpXP protease specificity-enhancing factor	21,212	18262,416	4,377	3		399718	267494	10,0	5,8	0,67	0,00	0,00
STHA_ECODH	*sthA*	Soluble pyridine nucleotide transhydrogenase	27,253	51560,395	6,084	6		2461945	708949	8,8	7,0	0,29	0,00	0,00
B1XCK7_ECODH	*stpA*	DNA-binding protein	30,597	15347,512	7,95	3		5231610	2526947	8,6	7,2	0,48	0,00	0,00
B1X6Q6_ECODH	*sucA*	2-oxoglutarate decarboxylase, thiamin-requiring	33,441	105061,72	6,038	23		10894610	6182535	4,4	3,3	0,57	0,00	0,00
B1X6Q7_ECODH	*sucB*	Dihydrolipoyltranssuccinase	28,889	44011,395	5,576	9		13005169	7183736	10,3	8,0	0,55	0,00	0,00
SUCC_ECODH	*sucC*	Succinyl-CoA ligase [ADP-forming] subunit beta	40,722	41392,645	5,367	13		14918991	7464789	8,8	5,2	0,50	0,00	0,00
B1X6Q9_ECODH	*sucD*	Succinyl-CoA ligase [ADP-forming] subunit alpha	46,021	29777,477	6,318	9		5049959	3320081	9,6	12,4	0,66	0,00	0,00
B1XFZ2_ECODH	*sufA*	Fe-S cluster assembly protein	16,393	13300,113	4,848	1		105006	110184	11,9	8,7	1,05	0,53	0,59
B1XFZ1_ECODH	*sufB*	Component of SufBCD complex	8,687	54745,316	5,058	3		48123	88289	24,3	8,8	1,83	0,00	0,01
B1XFZ0_ECODH	*sufC*	Component of SufBCD complex, ATP-binding compone	11,29	27582,367	4,84	3		33116	98936	54,6	7,7	2,99	0,01	0,01
B1XFY9_ECODH	*sufD*	Component of SufBCD complex	5,674	46822,75	6,358	2		52298	54674	16,8	5,2	1,05	0,63	0,69
B1XFG8_ECODH	*sufI*	Cell division protein FtsP	15,319	51858,164	5,935	5		262052	253402	12,8	17,4	0,97	0,77	0,82
B1XB08_ECODH	*suhB*	Inositol monophosphatase	38,577	29172,127	6,448	7		3186870	2449053	9,6	3,2	0,77	0,00	0,00
B1XC54_ECODH	*surA*	Chaperone SurA	36,682	47283,715	6,478	11		3288813	4048968	6,8	3,4	1,23	0,00	0,00
B1XAD5_ECODH	*talA*	Transaldolase	33,544	35658,801	5,889	9		4495993	4922468	22,5	8,0	1,09	0,48	0,54
B1XBD3_ECODH	*talB*	Transaldolase	60,252	35219,246	5,106	15		35799809	42913459	3,6	2,4	1,20	0,00	0,00
TAM_ECODH	*tam*	Trans-aconitate 2-methyltransferase	10,317	29005,959	4,855	2		91907	71124	9,9	11,0	0,77	0,01	0,03
B1XDP0_ECODH	*tas*	Predicted oxidoreductase, NADP(H)-dependent aldo-ke	6,647	38499,539	6,266	2		204733	308966	12,1	8,4	1,51	0,00	0,00
B1XAK1_ECODH	*tatB*	Sec-independent protein translocase protein TatB	14,035	18420,869	5,133	2		125491	111953	17,5	13,1	0,89	0,32	0,39
B1X622_ECODH	*tatE*	Probable Sec-independent protein translocase protein T	16,418	7024,294	9,05	1		93079	109894	15,8	11,8	1,18	0,14	0,18
B1XGT2_ECODH	*tdcB*	Catabolic threonine dehydratase, PLP-dependent	23,1	35232,414	5,749	5		1556274	74126	12,0	13,9	0,05	0,00	0,00
B1XGS9_ECODH	*tdcE*	Pyruvate formate-lyase 4/2-ketobutyrate formate-lyase	17,147	85935,547	5,483	8		6195414	2180776	6,8	5,4	0,35	0,00	0,00
TDH_ECODH	*tdh*	L-threonine 3-dehydrogenase	48,387	37239,043	5,943	10		2782344	2747157	9,8	4,1	0,99	0,82	0,85
B1XAT0_ECODH	*tdk*	Thymidine kinase	28,293	23456,576	5,98	3		433310	488248	8,4	9,6	1,13	0,11	0,15
B1XDE1_ECODH	*tehB*	Predicted S-adenosyl-L-methionine-dependent methyltr	30,457	22530,734	6,836	6		263071	443976	9,2	7,2	1,69	0,00	0,00
B1XFP0_ECODH	*tesB*	Acyl-CoA thioesterase II	9,091	31966,326	6,173	2		135110	135767	13,8	5,6	1,00	0,95	0,96
TGT_ECODH	*tgt*	Queuine tRNA-ribosyltransferase	8,533	42593,66	5,975	3		125627	156070	23,2	17,9	1,24	0,19	0,24
THII_ECODH	*thiI*	tRNA sulfurtransferase	21,162	54973,098	6,162	10		1499820	1176554	10,2	2,6	0,78	0,00	0,01
B1XBC7_ECODH	*thrA*	Fused aspartokinase I and homoserine dehydrogenase	45,854	89120,242	5,465	25	metabolism	3427310	1907606	4,3	6,8	0,56	0,00	0,00
KHSE_ECODH	*thrB*	Homoserine kinase	13,548	33623,645	5,453	2	metabolism	142783	87385	16,2	11,9	0,61	0,00	0,01
B1XBC9_ECODH	*thrC*	Threonine synthase	36,215	47113,836	5,238	10		2051717	1120405	7,7	5,4	0,55	0,00	0,00
SYT_ECODH	*thrS*	Threonine--tRNA ligase	21,651	74014,297	5,802	13		3702019	4953419	16,7	2,2	1,34	0,01	0,02
B1XDN3_ECODH	*thyA*	Thymidylate synthase	21,97	30479,688	5,62	5		718407	697178	12,1	5,4	0,97	0,66	0,72
TIG_ECODH	*tig*	Trigger factor	57,176	48192,668	4,825	20		53856473	43105130	6,2	2,2	0,80	0,00	0,00
B1XF97_ECODH	*tktA*	Transketolase	26,998	72211,742	5,429	13		14282430	16018294	3,6	5,6	1,12	0,01	0,02
B1XAD6_ECODH	*tktB*	Transketolase	19,34	73042,719	5,863	9		1351591	2250150	12,7	8,4	1,66	0,00	0,00
B1XHL6_ECODH	*tldD*	Predicted peptidase	24,532	51364,094	4,93	7		746325	724579	2,8	11,5	0,97	0,65	0,71
KTHY_ECODH	*tmk*	Thymidylate kinase	24,883	23783,143	5,326	4		771246	706411	26,7	11,6	0,92	0,54	0,60
TNAA_ECODH	*tnaA*	Tryptophanase	65,605	52773,465	5,876	21		216942465	3147705	10,4	7,3	0,01	0,00	0,00
B1X6S0_ECODH	*tolA*	Membrane anchored protein in TolA-TolQ-TolR comple:	7,838	43156,625	9,097	2		56836	68296	18,5	15,1	1,20	0,19	0,25
TOLB_ECODH	*tolB*	Protein TolB	41,628	45955,539	6,979	12		3905275	5319327	11,8	4,9	1,36	0,00	0,01
B1XG40_ECODH	*tolC*	Transport channel	54,97	53740,723	5,461	16		10467846	11523408	12,1	2,6	1,10	0,17	0,23
B1X6R8_ECODH	*tolQ*	Membrane spanning protein in TolA-TolQ-TolR comple›	13,043	25597,717	6,53	2		199136	340823	12,6	3,9	1,71	0,00	0,00
B1XBM3_ECODH	*topA*	DNA topoisomerase 1	20,578	97349,758	8,678	13		2564909	2484503	12,4	8,3	0,97	0,69	0,75
TPIS_ECODH	*tpiA*	Triosephosphate isomerase	48,235	26971,812	5,637	6		5623175	6468824	7,3	7,1	1,15	0,03	0,06
B1XCF2_ECODH	*tpx*	Probable thiol peroxidase	44,643	17835,316	4,75	5		9125338	12722291	3,2	9,4	1,39	0,00	0,00
TREA_ECODH	*treA*	Periplasmic trehalase	5,31	63636,719	5,6	2		38822	83775	12,2	7,4	2,16	0,00	0,00
B1XEM1_ECODH	*treB*	Fused trehalose(Maltose)-specific PTS enzyme: IIB cor	21,142	51080,742	9,139	7		6556994	1239704	7,0	9,4	0,19	0,00	0,00
B1XEM0_ECODH	*treC*	Trehalose-6-P hydrolase	21,96	63837,672	5,513	11		8307341	1743538	2,5	5,4	0,21	0,00	0,00
TRMA_ECODH	*trmA*	tRNA/tmRNA (uracil-C(5))-methyltransferase	16,667	41966,949	5,707	3		397850	187174	4,5	11,7	0,47	0,00	0,00
TRMD_ECODH	*trmD*	tRNA (guanine-N(1)-)-methyltransferase	10,588	28422,359	5,502	2		153857	116918	11,9	34,6	0,76	0,22	0,28
B1XFC0_ECODH	*trmI*	tRNA (guanine-N(7)-)-methyltransferase	14,644	27307,322	6,417	3		382752	300295	16,6	9,9	0,78	0,05	0,08
TRPA_ECODH	*trpA*	Tryptophan synthase alpha chain	22,761	28724,162	5,314	4		182293	255671	18,4	9,2	1,40	0,02	0,03
TRPB_ECODH	*trpB*	Tryptophan synthase beta chain	7,305	42983,008	5,709	2		189680	202225	11,8	17,0	1,07	0,54	0,60
B1X731_ECODH	*trpS*	Tryptophan--tRNA ligase	32,934	37437,82	6,267	8		1883796	2343743	17,5	4,2	1,24	0,05	0,08
TRUA_ECODH	*truA*	tRNA pseudouridine synthase A	10,37	30399,619	8,683	2		82231	65917	21,7	24,5	0,80	0,24	0,30
B1XGX8_ECODH	*truB*	tRNA pseudouridine synthase B	29,936	35087,078	5,705	6		483916	413389	15,4	3,9	0,85	0,08	0,12
TRUD_ECODH	*truD*	tRNA pseudouridine synthase D	27,794	39091,488	6,153	6		556589	625438	13,2	7,3	1,12	0,19	0,25
B1X9Z5_ECODH	*trxA*	Thioredoxin	46,789	11806,623	4,674	4		6981308	12329174	15,4	6,6	1,77	0,00	0,00

B1X828_ECODH	*trxB*	Thioredoxin reductase	62,305	34623,035	5,295	12		5068777	6356114	9,6	4,1	1,25	0,00	0,01
TSAC_ECODH	*tsaC*	Threonylcarbamoyl-AMP synthase	28,947	20767,738	4,941	3		244473	318822	10,2	17,5	1,30	0,04	0,06
TSAD_ECODH	*tsaD*	tRNA N6-adenosine threonylcarbamoyltransferase	6,825	36008,406	5,915	2		81214	109188	21,6	16,5	1,34	0,08	0,12
EFTS_ECODH	*tsf*	Elongation factor Ts	49,47	30422,979	5,215	11		32316195	34652515	8,0	4,1	1,07	0,18	0,23
B1XEZ9_ECODH	*tsx*	Nucleoside channel, receptor of phage T6 and colicin K	42,177	33589,023	5,07	8		8925515	8255553	9,0	8,8	0,92	0,26	0,32
TTCA_ECODH	*ttcA*	tRNA 2-thiocytidine biosynthesis protein TtcA	28,939	35560,914	5,953	6		586470	375413	3,1	12,1	0,64	0,00	0,00
B1XBY2_ECODH	*tufB*	Elongation factor Tu				19		534128784	505218027	2,9	6,5	0,95	0,17	0,22
TUSA_ECODH	*tusA*	Sulfurtransferase TusA	23,457	9094,543	5,181	1		97180	82895	42,1	18,0	0,85	0,47	0,54
TUSB_ECODH	*tusB*	Protein TusB	12,632	10718,333	5,115	1		43024	22886	15,4	18,3	0,53	0,00	0,01
B1XBS2_ECODH	*tyrA*	T-protein	7,775	42042,523	5,682	2		28274	34931	5,6	21,8	1,24	0,10	0,14
B1XCU2_ECODH	*tyrB*	Tyrosine aminotransferase, tyrosine-repressible, PLP-d	41,562	43537,809	5,318	12		1204543	1908283	14,6	7,4	1,58	0,00	0,00
B1XCF1_ECODH	*tyrR*	DNA-binding transcriptional dual regulator, tyrosine-binc	12,865	57656,141	5,543	5		226553	177907	7,6	10,8	0,79	0,01	0,02
SYY_ECODH	*tyrS*	Tyrosine--tRNA ligase	26,651	47526,969	5,59	9		1880290	2539905	9,2	7,6	1,35	0,00	0,01
B1XAK5_ECODH	*ubiD*	3-octaprenyl-4-hydroxybenzoate carboxy-lyase	19,718	55603,75	5,31	7		739822	601791	13,2	9,0	0,81	0,04	0,07
UBIE_ECODH	*ubiE*	Ubiquinone/menaquinone biosynthesis C-methyltransfe	33,068	28073,211	7,771	7		1568345	1451974	14,3	7,7	0,93	0,37	0,44
UBIG_ECODH	*ubiG*	Ubiquinone biosynthesis O-methyltransferase	42,5	26555,451	6,119	6		739010	814647	9,4	8,4	1,10	0,17	0,23
B1XA97_ECODH	*ucpA*	Predicted oxidoredutase, sulfate metabolism protein	20,152	27849,975	5,126	4		903457	213461	3,9	5,9	0,24	0,00	0,00
B1X702_ECODH	*udk*	Uridine kinase	12,676	24353,115	6,393	3		354055	346609	9,3	11,4	0,98	0,78	0,82
B1XAJ5_ECODH	*udp*	Uridine phosphorylase	69,565	27159,082	5,813	10		6045186	17750574	18,0	5,7	2,94	0,00	0,00
B1XF82_ECODH	*uidA*	Beta-D-glucuronidase	21,891	68447	5,244	9	metabolism	316805	1090936	10,4	8,5	3,44	0,00	0,00
UPP_ECODH	*upp*	Uracil phosphoribosyltransferase	48,558	22533,264	5,317	7		6777178	3362007	9,1	6,4	0,50	0,00	0,00
B1X930_ECODH	*usg*	Predicted semialdehyde dehydrogenase	20,475	36364,133	4,381	3		364235	321161	12,2	13,8	0,88	0,20	0,26
B1XFR7_ECODH	*ushA*	Bifunctional UDP-sugar hydrolase and 5'-nucleotidase	13,091	60823,973	5,475	5		556841	675946	5,2	4,7	1,21	0,00	0,00
B1XCG2_ECODH	*uspE*	Stress-induced protein	27,848	35706,879	5,157	5		968793	62505	4,1	24,4	0,06	0,00	0,00
B1XCK3_ECODH	*uspF*	Stress-induced protein, ATP-binding protein	18,056	16016,532	5,603	2		713848	219211	11,0	11,4	0,31	0,00	0,00
B1X605_ECODH	*uspG*	Universal stress protein UP12	49,296	15935,185	6,028	5		1541950	1352569	6,6	14,8	0,88	0,15	0,20
B1X8Q3_ECODH	*uup*	Fused predicted transporter subunits of ABC superfamil	9,921	72066,883	5,355	3		347461	214349	16,6	11,8	0,62	0,00	0,01
UVRB_ECODH	*uvrB*	UvrABC system protein B	3,269	76226,055	5,14	2		63712	77990	19,0	7,5	1,22	0,11	0,15
B1XAH9_ECODH	*uvrD*	DNA-dependent ATPase I and helicase II	17,222	81989,883	5,857	10		504218	540863	7,9	7,6	1,07	0,25	0,31
B1X672_ECODH	*uvrY*	DNA-binding response regulator in two-component regu	22,477	23892,654	6,528	4		148162	455726	29,7	12,0	3,08	0,00	0,00
UXAC_ECODH	*uxaC*	Uronate isomerase	25,957	53987,18	5,438	8		886999	315597	5,7	15,2	0,36	0,00	0,00
B1X9L9_ECODH	*vacJ*	Predicted lipoprotein	17,928	28041,602	4,895	3		409319	405491	18,7	3,4	0,99	0,92	0,93
B1XEN7_ECODH	*valS*	Valine--tRNA ligase	34,595	108192,35	5,199	24		9604324	9674977	6,2	2,8	1,01	0,83	0,87
B1X6X0_ECODH	*wbbI*	Conserved protein	26,97	37757,215	5,847	7		2668883	2317496	9,2	7,7	0,87	0,06	0,09
B1X6W8_ECODH	*wbbK*	Lipopolysaccharide biosynthesis protein	23,118	43188,176	9,017	7		802727	554109	4,2	5,6	0,69	0,00	0,00
WECG_ECODH	*wecG*	UDP-N-acetyl-D-mannosaminuronic acid transferase	19,106	27928,182	9,562	3		159654	178207	14,1	7,6	1,12	0,21	0,27
B1XAF6_ECODH	*wzzE*	Enterobacterial Common Antigen (ECA) polysaccharide	32,471	39489,102	6,247	6		550883	671426	13,4	6,5	1,22	0,04	0,06
EX7L_ECODH	*xseA*	Exodeoxyribonuclease 7 large subunit	12,281	51832,23	9,992	4		127161	289448	11,5	9,1	2,28	0,00	0,00
EX7S_ECODH	*xseB*	Exodeoxyribonuclease 7 small subunit	35	8951,932	4,429	2		591935	593378	12,1	8,2	1,00	0,97	0,98
B1XGL0_ECODH	*xthA*	Exonuclease III	30,597	30969,166	5,799	8		917699	915498	8,4	11,2	1,00	0,97	0,98
YAAA_ECODH	*yaaA*	UPF0246 protein YaaA	18,217	29585,828	6,859	4		408557	452247	18,1	11,8	1,11	0,39	0,46
B1XCA2_ECODH	*yadG*	Predicted transporter subunit: ATP-binding component (	16,883	34647,066	8,438	4		194820	251664	3,1	9,7	1,29	0,00	0,01
YAEH_ECODH	*yaeH*	UPF0325 protein YaeH	39,062	15096,169	6,614	5		964223	1228484	5,9	8,9	1,27	0,00	0,01
YAEP_ECODH	*yaeP*	UPF0253 protein YaeP	56,061	7214,222	4,536	2		303109	561145	11,8	7,6	1,85	0,00	0,00
B1XD72_ECODH	*yafC*	Predicted DNA-binding transcriptional regulator	8,553	33775,82	6,93	2		32368	78027	54,0	4,9	2,41	0,01	0,02
YAIE_ECODH	*yaiE*	UPF0345 protein YaiE	23,404	10234,396	4,469	2		463908	975305	8,6	14,0	2,10	0,00	0,00
B1XEU7_ECODH	*yaiL*	Nucleoprotein/polynucleotide-associated enzyme	17,318	19922,76	7,834	2		145567	146836	16,5	3,8	1,01	0,93	0,94
B1XEZ5_ECODH	*yajC*	SecYEG protein translocase auxillary subunit	20	11887,143	9,572	2		1152952	1207343	19,4	14,3	1,05	0,72	0,77
B1XEZ8_ECODH	*yajD*	Conserved protein	16,522	13363,993	6,14	1		87268	63112	20,0	35,1	0,72	0,17	0,23
B1XFM2_ECODH	*yajG*	Predicted lipoprotein	40,104	20950,027	8,685	4		1141064	1162970	11,1	8,6	1,02	0,79	0,83
B1XF12_ECODH	*yajL*	Conserved protein	25	20777,092	5,245	3		344315	328893	11,0	13,9	0,96	0,62	0,68
B1XF07_ECODH	*yajO*	Predicted oxidoreductase, NAD(P)-binding	27,778	36420,168	5,185	6		284441	417449	7,8	8,1	1,47	0,00	0,00
YAJQ_ECODH	*yajQ*	UPF0234 protein YajQ	53,988	18343,861	5,957	7		6440318	6658973	3,9	3,8	1,03	0,27	0,33
YBAB_ECODH	*ybaB*	Nucleoid-associated protein YbaB	21,101	12014,82	5,012	2		824154	773236	17,9	5,3	0,94	0,52	0,59
B1XFR5_ECODH	*ybaL*	Predicted transporter with NAD(P)-binding Rossmann-fo	5,018	59423,531	4,996	2		119484	99987	11,7	12,7	0,84	0,10	0,14
B1XFS2_ECODH	*ybaS*	Glutaminase	16,452	32903,324	4,814	2		171014	279396	22,7	16,6	1,63	0,02	0,03
B1XFP1_ECODH	*ybaY*	Predicted outer membrane lipoprotein	32,632	19431,199	7,866	3		1254415	969929	12,3	3,9	0,77	0,01	0,01
B1XFS9_ECODH	*ybbN*	Predicted thioredoxin domain-containing protein	63,028	31791,064	4,499	9		2065000	3771988	15,4	7,2	1,83	0,00	0,00
B1XGC6_ECODH	*ybcJ*	Predicted RNA-binding protein	30	7389,537	7,838	2		136434	572675	55,6	11,8	4,20	0,00	0,00
B1X5X4_ECODH	*ybdF*	Conserved protein	13,115	14050,323	8,71	1		18574	13046	9,8	29,3	0,70	0,10	0,15
B1X5X2_ECODH	*ybdG*	Predicted mechanosensitive channel	6,988	46602,648	7,872	2		143168	212971	29,8	17,6	1,49	0,05	0,08
B1X632_ECODH	*ybeB*	Ribosomal silencing factor RsfS	15,238	11582,228	4,519	1		192910	368579	18,8	7,2	1,91	0,00	0,00
B1X626_ECODH	*ybeD*	UPF0250 protein YbeD	39,08	9827,346	5,497	2		1129884	1428104	8,7	14,1	1,26	0,03	0,05
B1X638_ECODH	*ybeL*	Conserved protein	16,875	18797,201	5,107	3		163310	162718	30,4	15,7	1,00	0,99	0,99
B1X655_ECODH	*ybeZ*	Uncharacterized protein	52,601	39038,699	5,711	12		2496865	4375002	6,6	8,9	1,75	0,00	0,00
B1X6L8_ECODH	*ybfE*	LexA regulated protein	16,495	11279,979	10,086	1		85785	69891	11,4	7,1	0,81	0,02	0,04
B1X6S3_ECODH	*ybgF*	Uncharacterized protein	31,559	28231,404	8,581	4		1237424	1395428	13,3	9,8	1,13	0,21	0,27
B1X6P1_ECODH	*ybgI*	Putative GTP cyclohydrolase 1 type 2	27,53	26892,48	5,067	4		1215452	2460000	3,9	7,7	2,02	0,00	0,00
B1X6P2_ECODH	*ybgJ*	Predicted enzyme subunit	12,844	23946,602	5,162	1		61077	82004	20,6	20,7	1,34	0,09	0,13
B1X6P3_ECODH	*ybgK*	Predicted enzyme subunit	17,742	34386,352	8,591	5		137380	342051	5,9	3,9	2,49	0,00	0,00
B1X796_ECODH	*ybhA*	Pyridoxal phosphatase / fructose 1,6-bisphosphatase	30,147	30201,309	5,506	4		327548	413535	12,9	6,9	1,26	0,02	0,03
B1X7A3_ECODH	*ybhB*	Predicted kinase inhibitor	20,886	17085,121	5,27	2		178622	221768	9,1	19,6	1,24	0,09	0,13
B1X7A2_ECODH	*ybhC*	Predicted pectinesterase	33,255	46082,199	5,662	8		2014217	1303140	4,8	1,9	0,65	0,00	0,00
B1X7D0_ECODH	*ybiB*	Predicted transferase/phosphorylase	16,875	35048,539	6,377	4		263535	340432	14,4	11,5	1,29	0,03	0,06

B1X7E9_ECODH	*ybiS*	Conserved protein	44,118	33325,277	5,992	7		1975076	1806244	14,1	19,5	0,91	0,50	0,57
B1X7F0_ECODH	*ybiT*	Fused predicted transporter subunits of ABC superfamil	21,698	59857,996	4,989	8		1746348	1480389	5,2	4,5	0,85	0,00	0,01
B1X7F2_ECODH	*ybiV*	Predicted hydrolase	23,616	30412,828	5,909	4		175960	278450	15,5	9,6	1,58	0,00	0,01
B1X806_ECODH	*ybjP*	Predicted lipoprotein	42,69	18991,309	6,052	5		1293169	2013856	8,0	9,1	1,56	0,00	0,00
B1X818_ECODH	*ybjX*	Conserved protein	24,242	38357,551	9,437	5		739998	877404	7,6	8,4	1,19	0,02	0,04
B1X837_ECODH	*ycaC*	Predicted hydrolase	31,25	23100,314	5,201	4		615297	656446	15,3	5,3	1,07	0,45	0,52
B1X845_ECODH	*ycaO*	Conserved protein	14,676	65651,578	4,379	6		2285868	1448847	2,6	6,8	0,63	0,00	0,00
B1X8M9_ECODH	*ycbB*	Predicted carboxypeptidase	7,317	67812,492	8,634	3	peptidoglycan-modification	23424	49941	16,1	22,7	2,13	0,00	0,01
B1X8N1_ECODH	*ycbL*	Predicted metal-binding enzyme	39,07	23784,051	4,946	5		940440	1266897	10,4	10,5	1,35	0,01	0,01
B1X8Q1_ECODH	*ycbX*	Predicted 2Fe-2S cluster-containing protein	10,298	40644,41	7,577	2		130136	130418	15,3	20,0	1,00	0,99	0,99
B1X8Q9_ECODH	*ycbZ*	Predicted peptidase	16,041	65818,656	4,689	5		337914	308628	11,7	11,6	0,91	0,33	0,40
B1X8R5_ECODH	*yccF*	Conserved inner membrane protein	14,865	16275,364	9,412	2		44816	110425	16,2	13,6	2,46	0,00	0,00
B1X9C4_ECODH	*yccJ*	Uncharacterized protein	25,333	8524,493	4,701	1		366409	394647	21,0	16,0	1,08	0,63	0,69
B1X8R9_ECODH	*yccU*	Predicted CoA-binding protein with NAD(P)-binding Ros	18,248	14701,123	6,723	2		254998	340462	17,4	15,5	1,34	0,05	0,08
YCDX_ECODH	*ycdX*	Probable phosphatase YcdX	8,163	26890,512	5,534	2		89464	164285	24,6	8,3	1,84	0,00	0,01
B1X9E9_ECODH	*ycdY*	Conserved protein	16,304	20784,387	4,209	2		742367	733871	10,0	11,7	0,99	0,89	0,91
B1X9H6_ECODH	*yceB*	Predicted lipoprotein	25,806	20499,74	6,15	3		379816	465976	7,9	11,1	1,23	0,02	0,04
B1X9K0_ECODH	*yceF*	Maf-like protein YceF	24,227	21690,799	5,887	3		174398	226803	37,8	7,2	1,30	0,25	0,32
YCEH_ECODH	*yceH*	UPF0502 protein YceH	49,767	24177,33	5,02	7		876840	1101211	7,3	2,6	1,26	0,00	0,00
B1XA38_ECODH	*ycfD*	Conserved protein	26,005	42578,918	4,694	6		527282	558479	2,3	12,9	1,06	0,40	0,47
B1XA15_ECODH	*ycfM*	Penicillin-binding protein activator LpoB	36,15	22515,645	6,412	5		695778	1190270	20,2	10,6	1,71	0,00	0,01
YCFP_ECODH	*ycfP*	UPF0227 protein YcfP	31,667	21226,176	6,132	5		1079210	1153831	6,7	8,5	1,07	0,27	0,34
B1XA23_ECODH	*ycfS*	Conserved protein	7,188	34619,887	8,887	2	peptidoglycan-modification	3596	448	86,4	156,9	0,12	0,28	0,35
B1XA65_ECODH	*ycgK*	Uncharacterized protein	22,556	14905,824	9,498	2		58717	55540	14,4	21,6	0,95	0,71	0,76
YCGL_ECODH	*ycgL*	Protein YcgL	42,593	12414,504	9,162	3		385043	495568	19,1	13,1	1,29	0,09	0,13
B1XA67_ECODH	*ycgM*	Predicted isomerase/hydrolase	10,959	23711,117	5,879	1		165946	190576	24,9	17,6	1,15	0,46	0,52
YCGN_ECODH	*ycgN*	UPF0260 protein YcgN	12,418	17910,371	4,899	1		64202	78087	9,6	43,7	1,22	0,41	0,48
B1XAP4_ECODH	*ychF*	Ribosome-binding ATPase YchF	61,708	39667,32	4,87	15		8302648	7620389	5,5	4,6	0,92	0,05	0,08
B1XAR3_ECODH	*ychN*	Conserved protein	24,786	12692,747	5,018	2		138007	233410	22,5	14,7	1,69	0,01	0,01
B1XBK6_ECODH	*yciE*	Conserved protein	11,31	18961,447	4,939	1		30444	10544	8,7	20,5	0,35	0,00	0,00
B1XBK7_ECODH	*yciF*	Conserved protein	18,072	18597,164	5,474	2		211326	64541	26,4	14,3	0,31	0,00	0,00
B1XBM0_ECODH	*yciK*	Predicted oxoacyl-(Acyl carrier protein) reductase, Emrk	15,079	27932,912	7,666	3		149321	206555	6,0	11,7	1,38	0,00	0,01
B1XBM2_ECODH	*yciN*	Uncharacterized protein	31,325	9385,636	5,474	2		589908	870207	11,7	16,2	1,48	0,01	0,02
B1XBL6_ECODH	*yciO*	Conserved protein	24,272	23211,758	5,969	4		607359	596449	9,3	8,1	0,98	0,78	0,82
B1XBN5_ECODH	*yciT*	Predicted DNA-binding transcriptional regulator	15,261	27602,605	5,986	2		264278	239837	10,0	10,2	0,91	0,23	0,29
YCJF_ECODH	*ycjF*	UPF0283 membrane protein YcjF	8,499	39392,203	8,743	2		84785	253826	20,3	17,8	2,99	0,00	0,00
B1XCE9_ECODH	*ycjX*	Conserved protein with nucleoside triphosphate hydrola	10,108	52609,484	8,246	3		58917	155398	24,9	7,8	2,64	0,00	0,00
B1XD92_ECODH	*ydbK*	Pyruvate-flavodoxin oxidoreductase	5,111	128824,45	5,523	5		352694	369902	7,3	11,2	1,05	0,50	0,57
B1XDC7_ECODH	*ydcF*	Conserved protein	14,286	29705,711	5,501	3		251940	296698	4,5	3,7	1,18	0,00	0,00
B1XDE2_ECODH	*ydcL*	Predicted lipoprotein	11,261	24426,971	8,401	1		91790	87696	28,0	21,9	0,96	0,82	0,85
B1XE86_ECODH	*yddB*	Predicted porin protein	4,051	89282,883	6,006	2	transport	36372	67152	2,2	10,1	1,85	0,00	0,00
B1XE89_ECODH	*ydeN*	Conserved protein	41,071	62802,266	5,67	12		3070302	213889	2,3	7,1	0,07	0,00	0,00
B1XEC6_ECODH	*ydfG*	L-allo-threonine dehydrogenase, NAD(P)-binding	19,758	27248,932	5,652	4		2030177	2490044	14,0	4,7	1,23	0,03	0,05
B1XEC8_ECODH	*ydfZ*	Conserved protein	34,328	7276,347	7,941	2		223801	9672	24,4	46,5	0,04	0,00	0,00
B1XF79_ECODH	*ydgA*	Conserved protein	28,486	54688,996	5,068	10		3120119	3657565	8,0	4,5	1,17	0,01	0,02
B1XF69_ECODH	*ydgH*	Uncharacterized protein	37,261	33903,293	9,271	10		2119249	1808953	6,0	12,9	0,85	0,07	0,11
B1XF90_ECODH	*ydgT*	Predicted regulator	53,521	8416,578	6,249	3		199028	238580	12,6	12,5	1,20	0,08	0,12
B1XFV8_ECODH	*ydhF*	Predicted oxidoreductase	21,477	33675,648	5,765	4		971607	693886	7,0	8,7	0,71	0,00	0,00
B1XFX4_ECODH	*ydhQ*	Conserved protein	16,986	42876,008	4,423	6		770461	3369677	8,7	24,3	4,37	0,00	0,00
B1XFX5_ECODH	*ydhR*	Putative mono-oxygenase	32,673	11287,857	5,085	3		324672	593427	34,6	10,0	1,83	0,01	0,02
B1XFZ3_ECODH	*ydiH*	Uncharacterized protein	13,483	10453,968	6,398	1		13464	44468	36,1	9,4	3,30	0,00	0,00
B1XGM6_ECODH	*ydjA*	Predicted oxidoreductase	28,415	20059,006	6,934	4		789739	1066797	9,1	5,9	1,35	0,00	0,00
B1XGJ0_ECODH	*ydjN*	Predicted transporter	5,832	48661,691	9,059	2		115129	208298	4,9	10,6	1,81	0,00	0,00
B1XGN7_ECODH	*yeaC*	Conserved protein	18,889	10338,001	5,232	1		77101	53124	12,7	17,1	0,69	0,01	0,02
B1XGP0_ECODH	*yeaD*	Conserved protein	35,374	32666,096	5,89	7		2312174	5672208	15,7	2,6	2,45	0,00	0,00
B1XGP3_ECODH	*yeaG*	Conserved protein with nucleoside triphosphate hydrola	11,491	74480,336	5,627	6		351129	312302	14,9	11,6	0,89	0,27	0,33
B1XGP7_ECODH	*yeaK*	Conserved protein	21,557	17850,643	6,509	3		224411	349503	14,1	16,6	1,56	0,01	0,01
B1XGQ2_ECODH	*yeaO*	Conserved protein	10,435	13386,214	6,29	1		30542	107476	35,3	11,4	3,52	0,00	0,00
B1XGR6_ECODH	*yeaY*	Predicted lipoprotein	22,28	20921,123	9,55	3		194671	387630	26,5	11,0	1,99	0,00	0,01
B1XH75_ECODH	*yeaZ*	Predicted peptidase	30,736	25180,812	5,016	4		230089	212950	7,1	6,5	0,93	0,15	0,20
B1XHD2_ECODH	*yebC*	Probable transcriptional regulatory protein YebC	12,195	26422,561	4,714	2		1338810	1107420	15,1	6,7	0,83	0,06	0,10
B1XHB4_ECODH	*yebE*	Conserved protein	38,356	23686,846	5,343	5	unknown	217522	167359	9,9	16,6	0,77	0,05	0,07
B1XHB5_ECODH	*yebF*	Protein YebF	27,049	13464,311	8,806	2		26061	47869	35,8	12,7	1,84	0,02	0,03
B1XHB6_ECODH	*yebG*	Conserved protein regulated by LexA	45,833	10717,01	4,352	2		215975	151646	7,5	7,3	0,70	0,00	0,00
B1XHC1_ECODH	*yebK*	Predicted DNA-binding transcriptional regulator	11,073	31975,572	6,181	2		91936	29999	5,6	26,4	0,33	0,00	0,00
B1XHA0_ECODH	*yebR*	Conserved protein	19,126	20277,213	4,676	3		353129	752011	21,0	10,8	2,13	0,00	0,00
B1XHA4_ECODH	*yebV*	Uncharacterized protein	24,359	8752,796	4,465	1		106328	109137	6,7	4,7	1,03	0,55	0,62
B1X669_ECODH	*yecA*	Conserved metal-binding protein	21,267	25039,277	4,523	3		393274	359441	11,2	5,5	0,91	0,21	0,27
B1X673_ECODH	*yecF*	Uncharacterized protein	21,622	8238,546	5,25	1		110143	64633	15,9	16,1	0,59	0,00	0,01
B1X664_ECODH	*yecJ*	Uncharacterized protein	48,193	9122,583	4,884	3		499018	277379	11,3	5,2	0,56	0,00	0,00
B1XHE3_ECODH	*yecM*	Predicted metal-binding enzyme	13,83	21205,014	5,325	2		46462	75168	9,0	16,5	1,62	0,00	0,01
B1X685_ECODH	*yedD*	Uncharacterized protein	31,387	14983,258	4,867	3		709504	657314	4,8	12,5	0,93	0,31	0,38
B1X6C8_ECODH	*yeeN*	Probable transcriptional regulatory protein YeeN	29,412	25867,105	4,713	5		1094623	1475565	9,6	10,4	1,35	0,01	0,01
B1X6T6_ECODH	*yeeR*	CP4-44 prophage predicted membrane protein	5,49	57236,668	5,773	2		48752	6677	23,6	51,7	0,14	0,00	0,00

B1X6U3_ECODH	*yeeX*	Conserved protein	33,588	15132,504	9,161	2		447143	411533	5,8	9,2	0,92	0,18	0,23
B1X6V2_ECODH	*yeeZ*	Predicted epimerase, with NAD(P)-binding Rossmann-f	22,993	29679,943	5,278	4		278428	471036	5,9	25,7	1,69	0,01	0,01
B1X7G5_ECODH	*yegD*	Predicted chaperone	11,333	49371,391	5,113	3		190912	85999	14,9	13,9	0,45	0,00	0,00
B1X7H6_ECODH	*yegP*	Uncharacterized protein	22,727	12024,296	9,435	2		134352	117074	20,1	18,0	0,87	0,32	0,38
B1X7H7_ECODH	*yegQ*	Predicted peptidase	8,609	51193,184	5,797	4		364504	341350	9,9	6,6	0,94	0,29	0,36
B1X7M0_ECODH	*yehZ*	Predicted transporter subunit: periplasmic-binding comp	14,426	32609,303	5,816	2		44570	71138	28,6	17,1	1,60	0,04	0,07
SFGH2_ECODH	*yeiG*	S-formylglutathione hydrolase YeiG	10,432	31259,279	5,177	1		30547	38443	16,9	37,6	1,26	0,33	0,40
EFPL_ECODH	*yeiP*	Elongation factor P-like protein	10	21532,631	4,924	2		305831	49041	17,2	37,4	0,16	0,00	0,00
B1X7N5_ECODH	*yeiT*	Predicted oxidoreductase	6,553	44329,465	5,265	2		169205	31475	19,5	35,1	0,19	0,00	0,00
B1X872_ECODH	*yeiU*	Undecaprenyl pyrophosphate phosphatase	9,283	26759,127	9,88	2		332271	24697	2,0	60,9	0,07	0,00	0,00
NDPA_ECODH	*yejK*	Nucleoid-associated protein YejK	24,179	37822,516	4,876	6		1106731	620177	7,2	6,1	0,56	0,00	0,00
YEJL_ECODH	*yejL*	UPF0352 protein YejL	36	8288,458	5,497	1		31537	85953	50,9	38,1	2,73	0,03	0,04
B1X902_ECODH	*yfbQ*	Predicted aminotransferase	17,284	45517,371	5,847	5		339815	557390	10,5	12,2	1,64	0,00	0,00
B1X904_ECODH	*yfbT*	Predicted hydrolase or phosphatase	16,667	23007,521	5,739	2		95159	81221	9,6	7,4	0,85	0,04	0,07
YFBU_ECODH	*yfbU*	UPF0304 protein YfbU	52,439	19536,203	6,065	9		1830132	3001542	8,5	7,2	1,64	0,00	0,00
B1X910_ECODH	*yfcD*	Predicted NUDIX hydrolase	42,778	20375,861	4,698	5		494240	793906	15,4	8,3	1,61	0,00	0,00
B1X911_ECODH	*yfcE*	Predicted phosphatase	28,804	20122,078	5,632	3		147535	336767	23,8	15,4	2,28	0,00	0,00
B1X936_ECODH	*yfcL*	Uncharacterized protein	55,435	10000,043	4,26	3		185904	284574	13,8	18,0	1,53	0,01	0,02
YFCN_ECODH	*yfcN*	UPF0115 protein YfcN	14,754	21013,318	8,405	1		121849	83903	16,8	20,1	0,69	0,03	0,05
B1X9L6_ECODH	*yfcZ*	Conserved protein	27,66	10317,643	4,25	2		3341617	1011095	6,6	9,6	0,30	0,00	0,00
B1X9M3_ECODH	*yfdH*	CPS-53 (KpLE1) prophage bactoprenol glucosyl transfe	22,549	34635,434	7,784	5		688769	626800	3,8	17,4	0,91	0,33	0,40
B1X9M4_ECODH	*yfdI*	CPS-53 (KpLE1) prophage predicted inner membrane p	9,255	51483,586	8,792	3		136034	143865	18,5	13,9	1,06	0,66	0,72
B1X9Q1_ECODH	*yfdZ*	Prediected aminotransferase, PLP-dependent	14,563	46216,215	7,71	3		221355	231720	15,5	15,7	1,05	0,69	0,74
B1X9R8_ECODH	*yfeD*	Predicted DNA-binding transcriptional regulator	12,308	14910,144	7,768	1		44921	14193	5,8	27,3	0,32	0,00	0,00
B1XAA2_ECODH	*yfeX*	Conserved protein	8,696	33052,266	5,336	2		321952	196136	11,3	16,4	0,61	0,00	0,01
B1XAA3_ECODH	*yfeY*	Uncharacterized protein	20,942	20897,609	5,213	2		142943	265359	13,8	8,0	1,86	0,00	0,00
B1XAE2_ECODH	*yffB*	Conserved protein	31,356	13600,587	6,091	3		544839	587654	13,1	10,2	1,08	0,41	0,48
B1XAC1_ECODH	*yffS*	Uncharacterized protein	6,093	31041,461	5,453	2		73701	105832	13,2	24,0	1,44	0,04	0,06
B1XAW9_ECODH	*yfgC*	Predicted peptidase	6,982	53907,922	7,084	2		91511	89456	12,9	29,4	0,98	0,90	0,92
B1XAX0_ECODH	*yfgD*	Predicted oxidoreductase	30,252	13398,582	5,895	2		402329	535482	8,0	9,4	1,33	0,00	0,01
B1XAX9_ECODH	*yfgG*	Uncharacterized protein	23,81	7461,619	11,888	1	unknown	75691	75824	16,5	4,7	1,00	0,99	0,99
B1XAY7_ECODH	*yfgL*	Outer membrane protein assembly factor BamB	30,102	41887,211	4,718	7		2051627	3732219	25,0	3,8	1,82	0,00	0,01
B1XAY8_ECODH	*yfgM*	Conserved protein	46,602	22176,055	5,065	6		1921559	2112866	6,3	7,1	1,10	0,09	0,13
B1XAZ9_ECODH	*yfhJ*	Conserved protein	24,242	7731,568	3,982	1		192103	227952	12,1	10,1	1,19	0,08	0,11
B1XAZ5_ECODH	*yfhM*	Conserved protein	2,057	181585,45	5,255	2		55531	61530	18,5	25,6	1,11	0,58	0,64
B1XB07_ECODH	*yfhQ*	Predicted methyltransferase	26,423	27047,938	5,687	5		796558	868057	7,7	3,6	1,09	0,09	0,13
B1XBR9_ECODH	*yfiA*	Cold shock protein associated with 30S ribosomal subu	53,097	12784,585	6,204	4		3639036	1350823	9,3	15,4	0,37	0,00	0,00
B1XBQ8_ECODH	*yfiF*	Predicted methyltransferase	35,072	37784,375	8,936	6		1458535	1154104	26,8	9,2	0,79	0,13	0,18
B1XBR8_ECODH	*yfiO*	Outer membrane protein assembly factor BamD	50,612	27829,396	6,158	10		3272970	4133851	6,4	5,6	1,26	0,00	0,00
B1XBT9_ECODH	*yfjG*	Conserved protein	10,759	17726,67	8,598	1		144174	40939	21,6	17,3	0,28	0,00	0,00
B1XCM7_ECODH	*ygaD*	Conserved protein	25,455	17581,719	5,061	3		300905	290957	9,9	4,8	0,97	0,56	0,63
B1XBX0_ECODH	*ygaU*	Uncharacterized protein	44,295	16063,117	5,708	6		4129999	4233206	14,0	9,5	1,02	0,78	0,82
B1XDI8_ECODH	*ygcF*	7-carboxy-7-deazaguanine synthase	26,457	25029,645	5,71	5		263130	307578	10,2	11,8	1,17	0,09	0,13
B1XDK4_ECODH	*ygdH*	Conserved protein	28,194	50971,891	6,046	8		845030	401739	6,1	3,6	0,48	0,00	0,00
B1XDL8_ECODH	*ygdI*	Uncharacterized protein	26,667	8174,202	5,599	1		1037095	897827	10,0	19,8	0,87	0,24	0,30
B1XDM0_ECODH	*ygdK*	Predicted Fe-S metabolism protein	13,605	15940,271	6,114	1		95264	74248	12,4	20,5	0,78	0,10	0,14
YGFZ_ECODH	*ygfZ*	tRNA-modifying protein YgfZ	27,607	36094,117	5,175	7		1668542	2013910	6,3	10,2	1,21	0,02	0,03
B1XEK5_ECODH	*yggE*	Conserved protein	31,707	26635,264	6,1	7		993479	2018908	7,1	1,8	2,03	0,00	0,00
B1XF98_ECODH	*yggG*	Predicted peptidase	26,19	26842,318	5,748	5		352972	844632	19,3	12,8	2,39	0,00	0,00
B1XFA6_ECODH	*yggJ*	Ribosomal RNA small subunit methyltransferase E	19,753	26978,062	6,183	4		212690	255552	12,7	5,6	1,20	0,04	0,07
B1XFB9_ECODH	*yggL*	Uncharacterized protein	12,963	12880,56	4,907	1		70260	70953	25,6	8,0	1,01	0,95	0,96
B1XFB8_ECODH	*yggN*	Uncharacterized protein	8,368	26429,213	8,968	2		49363	165301	57,4	5,4	3,35	0,01	0,01
B1XFB1_ECODH	*yggS*	Predicted enzyme	17,949	25787,436	6,092	3		217451	261532	25,2	9,3	1,20	0,27	0,34
B1XFB4_ECODH	*yggV*	dITP/XTP pyrophosphatase	38,579	21038,844	5,214	4		540275	411465	6,6	5,4	0,76	0,00	0,00
FETP_ECODH	*yggX*	Probable Fe(2+)-trafficking protein	52,747	10952,529	5,914	4		1287392	2708372	9,7	4,9	2,10	0,00	0,00
B1XFF5_ECODH	*yghZ*	Aldo-keto reductase	30,925	38832,16	6,723	6		543947	159878	2,5	6,2	0,29	0,00	0,00
B1XG42_ECODH	*ygiB*	Conserved outer membrane protein	34,555	19816,033	8,251	4	metabolism	419436	381910	20,0	8,3	0,91	0,39	0,46
B1XG43_ECODH	*ygiC*	Predicted enzyme	37,306	45026,016	4,688	12	metabolism	1201046	852169	5,9	91,3	0,71	0,39	0,46
B1XG59_ECODH	*ygiF*	Predicted adenylate cyclase	23,095	48388,953	5,731	8		617604	785908	11,1	8,9	1,27	0,01	0,03
B1XG34_ECODH	*ygiN*	Quinol monooxygenase	23,077	11532,391	5,793	3		441010	660088	14,9	7,9	1,50	0,00	0,01
B1XG26_ECODH	*ygiT*	Predicted DNA-binding transcriptional regulator	11,45	14703,1	9,098	1		46100	55952	12,8	3,7	1,21	0,02	0,04
B1XG29_ECODH	*ygiW*	Conserved protein	13,846	14010,758	5,076	2		791540	622986	10,4	15,5	0,79	0,05	0,07
B1XG78_ECODH	*ygjH*	Conserved protein	10	12315,344	5,012	1		41479	8940	20,4	15,7	0,22	0,00	0,00
B1XGS2_ECODH	*yhaJ*	Predicted DNA-binding transcriptional regulator	6,711	33256,406	6,051	2		53888	99233	15,0	12,1	1,84	0,00	0,00
B1XHH6_ECODH	*yhbG*	Predicted transporter subunit: ATP-binding component	32,78	26800,648	5,64	5		1181701	1245698	9,1	6,7	1,05	0,39	0,46
YHBJ_ECODH	*yhbJ*	UPF0042 nucleotide-binding protein YhbJ	25	32492,285	6,724	4		243183	323599	9,4	10,2	1,33	0,01	0,01
B1XHH5_ECODH	*yhbN*	Predicted transporter subunit: periplasmic-binding comp	10,27	20126,885	8,962	2		237541	208062	14,2	5,6	0,88	0,12	0,16
B1XGZ0_ECODH	*yhbY*	Predicted RNA-binding protein	21,649	10783,711	9,415	2		489384	799925	51,7	17,1	1,63	0,15	0,20
B1XHK6_ECODH	*yhcB*	Conserved protein	52,273	14960,849	5,648	5		3517374	4539249	9,3	4,4	1,29	0,00	0,01
B1XHM3_ECODH	*yhdH*	Predicted oxidoreductase, Zn-dependent and NAD(P)-b	31,173	34723,766	5,631	7		1357557	1064720	4,6	3,3	0,78	0,00	0,00
B1X6J5_ECODH	*yheO*	Predicted DNA-binding transcriptional regulator	21,667	26820,564	5,395	3		200797	378676	17,7	8,2	1,89	0,00	0,00
B1X6K2_ECODH	*yheS*	Fused predicted transporter subunits of ABC superfamil	16,797	71843,43	5,5	9		521565	728834	17,3	6,5	1,40	0,01	0,02
B1X754_ECODH	*yhgF*	Predicted transcriptional accessory protein	23,933	85119,867	5,918	15		2283843	2637820	8,0	10,2	1,15	0,07	0,10
B1X7Q6_ECODH	*yhhA*	Conserved protein	34,932	16623,82	10,888	3		130234	367404	24,5	9,9	2,82	0,00	0,00

B1X7S3_ECODH	*yhhF*	Ribosomal RNA small subunit methyltransferase D	12,626	21677,604	5,959	2		94998	116229	12,6	11,7	1,22	0,05	0,08
B1X7R7_ECODH	*yhhK*	Conserved protein	29,921	14505,534	6,585	2		97698	168846	5,8	9,9	1,73	0,00	0,00
B1X7S5_ECODH	*yhhM*	Conserved protein	36,134	13496,499	10,061	3		150822	333766	11,8	5,6	2,21	0,00	0,00
B1X7P8_ECODH	*yhhX*	Predicted oxidoreductase with NAD(P)-binding Rossma	29,275	38765,008	6,066	7		671157	1097723	9,4	7,5	1,64	0,00	0,00
B1X7V4_ECODH	*yhiR*	Ribosomal RNA large subunit methyltransferase J	13,571	31941,707	8,589	2		180634	118540	12,5	12,1	0,66	0,00	0,01
B1X8E5_ECODH	*yhjJ*	Predicted zinc-dependent peptidase	18,474	55527,371	5,726	6		569682	560979	13,0	10,2	0,98	0,86	0,89
B1X8G7_ECODH	*yiaD*	Predicted outer membrane lipoprotein	52,968	22197,221	9,81	5		2315098	1771207	15,9	4,4	0,77	0,02	0,03
B1X8G9_ECODH	*yiaF*	Conserved protein	27,966	25663,102	6,079	5		1312388	1360167	11,3	12,6	1,04	0,68	0,73
B1X8K7_ECODH	*yibF*	Predicted glutathione S-transferase	7,426	22545,061	5,101	2		35358	78196	3,4	3,3	2,21	0,00	0,00
B1X8L7_ECODH	*yibL*	Conserved protein	18,333	13696,007	9,485	2		160437	209387	9,7	7,0	1,31	0,00	0,01
B1X945_ECODH	*yibN*	Predicted rhodanese-related sulfurtransferase	26,573	15596,255	9,372	2		1667743	1755097	8,4	9,3	1,05	0,45	0,52
B1X8L6_ECODH	*yibT*	Uncharacterized protein	42,029	7995,298	9,395	2		525235	598573	11,7	6,7	1,14	0,11	0,16
B1X977_ECODH	*yicC*	Conserved protein	17,422	33174,926	5,1	5		747717	959204	10,1	8,9	1,28	0,01	0,02
B1X988_ECODH	*yicH*	Conserved protein	11,599	62272,441	5,673	5		310727	354872	17,5	12,7	1,14	0,25	0,32
B1X9S7_ECODH	*yidA*	Predicted hydrolase	12,593	29721,139	5,108	2		318100	322996	12,6	16,2	1,02	0,88	0,91
B1X9S8_ECODH	*yidB*	Conserved protein	34,848	13786,576	4,368	2		148142	141655	2,4	13,0	0,96	0,54	0,61
YIDC_ECODH	*yidC*	Membrane protein insertase YidC	30,657	61525,922	7,7	8		4344267	3442190	10,1	6,0	0,79	0,01	0,02
B1X9U2_ECODH	*yieF*	Chromate reductase, Class I, flavoprotein	50,532	20375,562	5,007	5		527387	830538	6,7	7,9	1,57	0,00	0,00
B1X9Y3_ECODH	*yifE*	Conserved protein	36,607	13133,577	6,102	3		374353	558569	28,2	13,7	1,49	0,04	0,06
B1XAH5_ECODH	*yifL*	Predicted lipoprotein	46,269	7177,217	8,889	2		63781	177523	50,6	9,5	2,78	0,01	0,01
B1XAH8_ECODH	*yigB*	Predicted hydrolase	12,605	27121,842	5,985	2	metabolism	102810	50298	33,7	9,1	0,49	0,01	0,02
B1XAI5_ECODH	*yigI*	Conserved protein	12,258	17162,771	6,423	2		46434	36534	7,2	9,0	0,79	0,01	0,01
B1XAJ1_ECODH	*yigL*	Predicted hydrolase	28,947	29707,729	5,228	4		330567	341612	4,7	18,0	1,03	0,75	0,79
B1XAM1_ECODH	*yihA*	Probable GTP-binding protein EngB	12,121	22083,229	6,769	2		174956	290959	21,4	8,8	1,66	0,00	0,01
B1XAL5_ECODH	*yihD*	Conserved protein	49,438	10272,91	5,179	3		676075	830824	22,1	6,3	1,23	0,14	0,19
B1XAL6_ECODH	*yihE*	Predicted kinase	10,976	38120,316	4,993	3	stress response	160189	90853	51,5	18,3	0,57	0,05	0,08
B1XB77_ECODH	*yiiM*	Conserved protein	14,732	25342,725	5,709	2		143451	225797	18,8	8,1	1,57	0,00	0,01
B1XC02_ECODH	*yjaG*	Conserved protein	22,449	22612,648	4,446	4		415926	347114	8,3	8,0	0,83	0,02	0,04
B1XCT3_ECODH	*yjbJ*	Predicted stress response protein	17,391	8325,252	5,441	1		877523	925408	17,5	11,5	1,05	0,64	0,69
B1XCU5_ECODH	*yjbR*	Conserved protein	26,271	13519,475	6,056	3		160853	419964	9,9	13,6	2,61	0,00	0,00
B1XD15_ECODH	*yjdC*	Predicted transcriptional regulator	27,749	21931,078	4,947	4		166313	495566	17,0	13,2	2,98	0,00	0,00
B1XDR9_ECODH	*yjeE*	ATPase with strong ADP affinity	17,647	16853,078	4,488	2		236724	228265	15,8	8,4	0,96	0,70	0,75
B1XDP8_ECODH	*yjeI*	Conserved protein	30,769	11958,425	5,497	2		1203742	2200289	9,6	8,0	1,83	0,00	0,00
B1XDR3_ECODH	*yjeP*	Predicted mechanosensitive channel	2,439	123967,61	6,551	2		12597	35094	53,8	22,9	2,79	0,01	0,02
YJGA_ECODH	*yjgA*	UPF0307 protein YjgA	15,301	21359,279	5,3	2		173882	175478	6,3	6,1	1,01	0,84	0,87
B1XEM4_ECODH	*yjgF*	Ketoacid-binding protein	64,844	13611,593	5,361	4		5335110	5650572	23,7	6,9	1,06	0,66	0,72
B1XEN1_ECODH	*yjgK*	Conserved protein	22,667	16865,359	5,308	2		190708	185137	11,2	13,3	0,97	0,75	0,80
B1XEP1_ECODH	*yjgQ*	Conserved inner membrane protein	8,056	39618,605	9,48	2		96734	91580	18,9	13,6	0,95	0,68	0,73
B1XEP2_ECODH	*yjgR*	Predicted ATPase	6	54332,293	5,915	2		14306	52238	48,2	12,6	3,65	0,00	0,01
B1XEQ6_ECODH	*yjhC*	KpLE2 phage-like element predicted oxidoreductase	14,247	41384,488	6,102	4	metabolism	502335	333825	8,2	12,5	0,66	0,00	0,00
B1XER9_ECODH	*yjhU*	KpLE2 phage-like element predicted DNA-binding trans	5,488	36111,586	6,326	2		48932	56775	14,2	37,5	1,16	0,45	0,52
B1XFK1_ECODH	*yjjK*	Fused predicted transporter subunits of ABC superfamil	38,018	62442,883	5,431	17		8036433	7581272	5,3	3,5	0,94	0,11	0,16
B1XE48_ECODH	*ykgE*	Predicted oxidoreductase	10,46	26004,295	7,501	2		226379	27737	15,8	23,8	0,12	0,00	0,00
B1XE49_ECODH	*ykgF*	Predicted amino acid dehydrogenase with NAD(P)-bindi	8,211	53051,898	8,749	3		274944	22111	20,3	10,0	0,08	0,00	0,00
B1XE50_ECODH	*ykgG*	Predicted transporter	22,511	25212,707	4,963	3		488072	27821	10,9	46,7	0,06	0,00	0,00
B1X7X9_ECODH	*yliJ*	Predicted glutathione S-transferase	30,288	23713,178	5,05	5		240328	356581	6,8	10,6	1,48	0,00	0,00
B1X8Q6_ECODH	*ymbA*	Conserved protein	28,877	20634,406	5,807	3		261215	284288	8,3	13,8	1,09	0,33	0,40
B1X9F9_ECODH	*ymdB*	O-acetyl-ADP-ribose deacetylase	12,429	18880,385	5,445	1		24623	38456	21,9	12,2	1,56	0,02	0,03
B1XBP6_ECODH	*ymjA*	Uncharacterized protein	30,864	9321,317	4,234	1		53187	57048	16,5	10,3	1,07	0,51	0,58
B1XCF9_ECODH	*ynaI*	Conserved inner membrane protein	19,242	38754,508	9,363	3		172184	284282	17,8	16,3	1,65	0,01	0,02
B1XDG0_ECODH	*yncB*	NADPH-dependent curcumin/dihydrocurcumin reductas	7,536	37609,746	5,526	2		201744	147352	8,2	7,1	0,73	0,00	0,00
B1XDG2_ECODH	*yncD*	Predicted iron outer membrane transporter	4,714	77260,672	5,321	2		42293	40484	48,3	7,0	0,96	0,86	0,89
B1XDG3_ECODH	*yncE*	Conserved protein	29,745	38612,957	9,22	7		696278	673626	7,3	5,7	0,97	0,49	0,56
YNFB_ECODH	*ynfB*	UPF0482 protein YnfB	10,619	12908,668	9,15	1		161289	279061	73,9	4,5	1,73	0,27	0,34
B1XF51_ECODH	*ynfD*	Uncharacterized protein	21,782	10476,697	4,735	1	unknown	45566	35725	23,5	17,4	0,78	0,16	0,21
B1XFY6_ECODH	*ynhG*	Conserved protein	7,784	36082,348	9,422	2		50678	91070	22,0	161,0	1,80	0,45	0,52
B1XGI6_ECODH	*yniA*	Predicted phosphotransferase/kinase	24,476	32458,617	4,979	4		269427	138530	3,4	15,2	0,51	0,00	0,00
B1XGI8_ECODH	*yniC*	Predicted hydrolase	18,018	24330,24	4,797	3		304529	361349	12,6	5,2	1,19	0,04	0,06
B1XH77_ECODH	*yoaB*	Conserved protein	21,93	12493,228	4,964	2		800535	1094878	11,9	4,4	1,37	0,00	0,01
B1XH78_ECODH	*yoaC*	Uncharacterized protein	11,765	13231,266	5,156	1		15203	19323	39,4	24,3	1,27	0,32	0,38
B1XGQ3_ECODH	*yoaF*	Conserved outer membrane protein	16,667	8942,377	7,661	1		39825	44488	25,3	16,1	1,12	0,51	0,58
B1XAA5_ECODH	*ypeA*	Acetyltransferase YpeA	11,348	16311,566	4,835	1		28598	37312	47,9	10,9	1,30	0,43	0,50
B1XAE7_ECODH	*ypfJ*	Conserved protein	14,634	31460,186	5,578	3		141642	307368	27,6	12,5	2,17	0,00	0,01
YQFB_ECODH	*yqfB*	UPF0267 protein YqfB	17,476	11905,412	4,682	1		93092	89589	28,8	14,8	0,96	0,81	0,84
YQGE_ECODH	*yqgE*	UPF0301 protein YqgE	43,85	20685,906	5,343	5		368095	672385	18,7	7,2	1,83	0,00	0,00
RUVX_ECODH	*yqgF*	Putative Holliday junction resolvase	27,536	15186,305	6,741	2		89144	87993	2,4	17,5	0,99	0,88	0,91
B1XFG3_ECODH	*yqhD*	Alcohol dehydrogenase, NAD(P)-dependent	35,917	42097,023	5,72	9		2026968	1705464	12,3	9,7	0,84	0,07	0,10
B1XG47_ECODH	*yqiC*	Conserved protein	16,667	11275,98	5,895	2		203730	505762	38,3	10,0	2,48	0,00	0,01
B1XGA2_ECODH	*yqjD*	Conserved protein	25,743	11051,492	9,046	2		1389828	917591	16,8	20,6	0,66	0,03	0,05
B1XG74_ECODH	*yqjH*	Predicted siderophore interacting protein	17,717	28871,555	5,532	2	transport	125739	152154	31,9	21,7	1,21	0,35	0,42
B1XG75_ECODH	*yqjI*	Predicted transcriptional regulator	10,145	23401,289	6,266	2		81011	71584	23,3	11,5	0,88	0,38	0,45
B1XGV8_ECODH	*yraL*	Ribosomal RNA small subunit methyltransferase I	15,385	31347,904	5,828	2		123003	75205	9,0	30,0	0,61	0,03	0,04
B1XGV9_ECODH	*yraM*	Penicillin-binding protein activator LpoA	28,171	72825,164	5,265	10		1087232	971310	3,9	3,5	0,89	0,01	0,01
B1XGW2_ECODH	*yraP*	Uncharacterized protein	26,702	20027,949	9,038	3		794735	964385	11,8	7,4	1,21	0,03	0,05

B1XHG5_ECODH	*yrbA*	Predicted DNA-binding transcriptional regulator	29,762	9451,736	5,831	2	351802	497982	15,5	10,7	1,42	0,01	0,02
B1XHG7_ECODH	*yrbC*	Predicted ABC-type organic solvent transporter	37,915	23962,611	9,354	5	256809	568235	32,9	18,8	2,21	0,01	0,01
B1XHG8_ECODH	*yrbD*	Predicted ABC-type organic solvent transporter	44,809	19576,223	4,787	6	1268656	1489178	6,5	6,3	1,17	0,01	0,02
B1XHH0_ECODH	*yrbF*	Predicted toluene transporter subunit: ATP-binding com	31,599	29096,805	6,157	6	402623	473575	5,4	7,9	1,18	0,02	0,03
B1X6D2_ECODH	*yrdA*	Conserved protein	44,565	20245,023	5,259	6	807223	872818	7,7	2,1	1,08	0,09	0,13
B1XAJ4_ECODH	*ysgA*	Predicted hydrolase	16,605	29425,211	5,569	3	46688	52589	17,6	15,7	1,13	0,36	0,43
B1XDV6_ECODH	*ytfB*	Predicted cell envelope opacity-associated protein	13,679	23505,5	5,063	2	122088	150139	22,0	16,9	1,23	0,20	0,26
B1XDX1_ECODH	*ytfP*	Conserved protein	11,504	12866,424	6,391	1	120010	242781	10,5	12,1	2,02	0,00	0,00
ZAPA_ECODH	*zapA*	Cell division protein ZapA	33,945	12594,17	5,217	3	422337	626524	14,1	9,4	1,48	0,00	0,01
ZAPB_ECODH	*zapB*	Cell division protein ZapB	50,617	9634,807	4,69	3	2708556	3988150	9,8	11,1	1,47	0,00	0,01
ZAPD_ECODH	*zapD*	Cell division protein ZapD	17,004	28291,629	6,31	3	466448	401658	6,4	4,2	0,86	0,01	0,02
B1X7S7_ECODH	*zntA*	Zinc, cobalt and lead efflux system	14,344	76839,875	5,697	7	420791	431222	7,3	12,1	1,02	0,74	0,79
B1XHC5_ECODH	*znuA*	Zinc transporter subunit: periplasmic-binding componen	14,194	33777,375	5,608	2	170179	126866	14,4	42,3	0,75	0,21	0,26
B1XCT4_ECODH	*zur*	DNA-binding transcriptional repressor, Zn(II)-binding	11,111	19254,168	5,974	1	23993	44486	39,2	25,5	1,85	0,04	0,07
B1XHC0_ECODH	*zwf*	Glucose-6-phosphate 1-dehydrogenase	39,511	55704,441	5,558	16	3585782	4885820	5,9	2,1	1,36	0,00	0,00
