# Supplementary material for: Molecular and proteome analyses highlight the importance of the Cpx envelope stress system for acid stress and cell wall stability in Escherichia coli
Source: Microbiologyopen. 2016 Apr 2;5(4):582–96. doi: 10.1002/mbo3.353 (PMC4985592; doi:10.1002/mbo3.353)
Supplement: Supplementary file 7 [file MBO3-5-582-s007.pdf]

Table S4A: Comparison of transcriptome and proteome data of Cpx-TCS target proteins.

All proteins being Cpx-dependently/-independently induced (S4A) or inhibited (S4B) are listed in comparison with the WT_{ON}/WT-ratios of transcriptome data from Raivio *et al.* , 2013. Furthermore, information on known CpxR~P binding motifs is provided. For each protein, the following ratios were calculated using the protein intensities measured in this study: WT_{ON}/WT (compares protein abundance between induced and non-induced WT); *cpxRA* /WT (compares protein abundance between non-induced *cpxRA* -strain and non-induced WT); *cpxRA* $_{ON}$/WT (compares protein abundance between induced *cpxRA* -strain and non-induced WT). To emphasize the higher amount (Cpx-specific induction) of proteins in induced WT-cells compared to induced *cpxRA* -cells we additionally calculated the [WT_{ON}/WT / *cpxRA* $_{ON}$/WT]-ratio (S4A). To highlight the higher amount (Cpx-specific inhibition) of proteins in induced *cpxRA* -cells compared to induced WT-cells we additionally calculated the [*cpxRA* $_{ON}$/WT / WT_{ON}/WT]-ratio (S4B). For each ratio minimum 2-fold difference is defined as significant. Calculating the difference between induced and non-induced *cpxRA* -cells, we checked whether *nlpE* -overexpression has an additional effect on the relative amounts of proteins. Minimum 2-fold difference (+) or (-) was defined as an additional NlpE-effect (S4A,B).

gene symbol	gene bank	Raivio mRNA NlpE/WT (MC4100 in LB)	CpxR~P motif	WT_{ON} p-value	WT_{ON} q-value (BH)	WT_{ON}/WT-ratio	*cpxAR* p-value	*cpxAR* q-value (BH)	*cpxAR* /WT-ratio	*cpxAR* $_{ON}$ p-value	*cpxAR* $_{ON}$ q-value (BH)	*cpxAR* $_{ON}$ /WT-ratio	x-fold more in induced WT compared to induced *cpxRA* -strain [WT_{ON}/WT] /[$cpxAR_{ON}$ /WT]-ratio	additional NlpE-effect (minimum 2-fold difference between induced and non-induced deletion mutant): positive (+) or negative (-)	global function
**Cpx-dependent induction**															
ycfS	b1113	4,152	yes (E; Yamamoto et	0,03	0,06	**28,70**	0,83	0,87	1,38	0,28	0,35	**0,12**	**230,47**	yes (-)	PG
cpxP	b4484	5,354	yes (E; Danese et al,	0,00	0,00	**14,01**	0,00	0,01	0,18	0,00	0,01	**0,36**	**38,88**	yes (+)	stress response
yebE	b1846	11,563	yes (E; Raivio, 2013)	0,00	0,00	**19,77**	0,00	0,00	0,69	0,05	0,07	**0,77**	**25,70**	no	unknown
cpxR	b3912	2,600	yes (E; De Wulf et al.	0,00	0,00	**3,02**	0,00	0,00	0,20	0,00	0,00	**0,20**	**14,80**	no	stress response
aceA	b4015	n.d.	no	0,00	0,00	**2,00**	0,00	0,00	0,18	0,00	0,00	**0,15**	**13,46**	no	metabolism
sbmA	b0377	4,183	yes (P; Raivio et al., 2	0,00	0,00	**8,30**	0,01	0,02	0,83	0,04	0,07	**0,82**	**10,14**	no	transport
degP	b0161	6,843	yes (E; Pogliano et al	0,00	0,00	**11,13**	0,03	0,06	0,88	0,04	0,07	**1,14**	**9,80**	no	degradation
htpX	b1829	2,324	yes (P; De Wulf et al.	0,00	0,00	**10,78**	0,79	0,83	0,95	0,12	0,17	**1,17**	**9,18**	no	degradation
spy	b1743	9,022	yes (E; Raivio et al., 2	0,00	0,00	**5,91**	0,96	0,96	1,01	0,18	0,23	**0,82**	**7,16**	no	stress response
lrhA	b2289	n.d.	no	0,00	0,00	**2,41**	0,02	0,03	0,76	0,00	0,00	**0,35**	**6,91**	yes (-)	chemotaxis
gltA	b0720	n.d.	no	0,00	0,00	**3,55**	0,00	0,00	0,60	0,00	0,00	**0,59**	**6,04**	no	metabolism
uidA	b1617	0,529	no	0,00	0,00	**20,12**	0,00	0,00	0,63	0,00	0,00	**3,44**	**5,84**	yes (+)	metabolism
fepA	b0584	n.d.	no	0,00	0,00	**6,21**	0,39	0,46	1,08	0,62	0,68	**1,07**	**5,81**	no	transport
yihE	b3859	2,187	yes (E; Pogliano et al	0,00	0,01	**3,23**	0,03	0,05	0,52	0,05	0,08	**0,57**	**5,70**	no	stress response
amiA	b2435	2,912	yes (E; Weatherspoo	0,00	0,00	**4,51**	0,05	0,08	0,82	0,01	0,03	**0,83**	**5,45**	no	PG
cirA	b2155	n.d.	no	0,00	0,00	**6,02**	0,20	0,26	1,22	0,52	0,58	**1,11**	**5,41**	no	transport
fadB	b3846	n.d.	no	0,00	0,00	**2,07**	0,00	0,00	1,78	0,00	0,00	**0,38**	**5,38**	yes (-)	metabolism
ppiA	b3363	1,832	yes (E; Pogliano et al	0,00	0,00	**3,03**	0,50	0,57	1,06	0,00	0,01	**0,58**	**5,21**	yes (-)	folding
ygiC	b3038	2,474	no	0,00	0,00	**3,55**	0,00	0,00	0,52	0,39	0,46	**0,71**	**5,00**	no	metabolism
ilvB	b3671	n.d.	no	0,00	0,00	**8,71**	0,05	0,08	0,75	0,00	0,01	**1,86**	**4,69**	yes (+)	metabolism
thrB	b0003	n.d.	no	0,00	0,00	**2,81**	0,32	0,39	0,91	0,00	0,01	**0,61**	**4,60**	no	metabolism
ygiB	b3037	2,311	no	0,00	0,00	**4,14**	0,90	0,92	0,99	0,39	0,46	**0,91**	**4,55**	no	metabolism
yigB	b3812	n.d.	no	0,01	0,02	**2,20**	0,12	0,18	0,71	0,01	0,02	**0,49**	**4,50**	no	metabolism
sdaA	b1814	1,786	no	0,00	0,00	**2,35**	0,00	0,00	0,50	0,00	0,00	**0,54**	**4,32**	no	metabolism
thrA	b0002	n.d.	no	0,00	0,00	**2,37**	0,00	0,00	0,84	0,00	0,00	**0,56**	**4,26**	no	metabolism
rihC	b0030	n.d.	no	0,02	0,05	**2,12**	0,00	0,01	0,42	0,01	0,03	**0,50**	**4,23**	no	metabolism
dsbA	b3860	2,136	yes (E; Raivio et al., 2	0,00	0,00	**5,07**	0,00	0,00	1,58	0,01	0,02	**1,24**	**4,09**	no	folding
miaA	b4171	2,053	no	0,00	0,00	**3,94**	0,01	0,02	0,72	0,96	0,97	**0,98**	**4,02**	no	metabolism
ynfD	b1586	2,373	no	0,00	0,00	**3,01**	0,01	0,02	1,59	0,16	0,21	**0,78**	**3,85**	yes (-)	unknown
yjhC	b4280	n.d.	no	0,00	0,00	**2,25**	0,00	0,00	0,74	0,00	0,00	**0,66**	**3,38**	no	metabolism
ilvC	b3774	n.d.	no	0,00	0,00	**8,45**	0,00	0,00	1,32	0,03	0,05	**2,61**	**3,24**	yes (+)	metabolism
mdoD	b1424	n.d.	no	0,00	0,01	**2,14**	0,42	0,49	0,90	0,01	0,02	**0,66**	**3,23**	no	metabolism
pyrB	b4245	n.d.	no	0,00	0,00	**2,96**	0,00	0,01	1,72	0,37	0,44	**0,92**	**3,22**	no	metabolism
entB	b0595	n.d.	no	0,00	0,01	**2,25**	0,03	0,05	0,73	0,02	0,03	**0,75**	**3,01**	no	metabolism
psd	b4160	1,614	yes (P; De Wulf et al.	0,00	0,00	**2,13**	0,14	0,20	0,88	0,01	0,03	**0,75**	**2,85**	no	metabolism
slt	b4392	2,519	yes (P; Raivio et al., 2	0,00	0,00	**4,21**	0,15	0,21	1,09	0,05	0,07	**1,50**	**2,82**	no	PG
entE	b0594	n.d.	no	0,00	0,00	**2,91**	0,75	0,80	0,97	0,74	0,78	**1,03**	**2,81**	no	metabolism
yfgG	b2504	2,595	yes (P; De Wulf et al.	0,00	0,00	**2,74**	0,87	0,89	0,98	0,99	0,99	**1,00**	**2,74**	no	unknown
ilvN	b3670	n.d.	no	0,00	0,00	**9,00**	0,49	0,56	1,20	0,00	0,01	**3,41**	**2,64**	yes (+)	metabolism
dacC	b0839	4,215	no	0,00	0,00	**3,13**	0,28	0,35	0,88	0,05	0,07	**1,32**	**2,38**	no	PG
yddB	b1495	n.d.	no	0,00	0,00	**4,34**	0,00	0,01	1,55	0,00	0,00	**1,85**	**2,35**	no	transport
hflX	b4173	1,726	no	0,00	0,00	**3,32**	0,27	0,34	1,11	0,00	0,00	**1,49**	**2,23**	no	metabolism
ycbB	b0925	2,907	no	0,00	0,00	**4,69**	0,00	0,00	2,09	0,00	0,01	**2,13**	**2,20**	no	PG
hflC	b4175	1,729	no	0,00	0,00	**2,12**	0,01	0,03	0,84	0,62	0,68	**0,97**	**2,18**	no	stress response
hflK	b4174	1,689	no	0,00	0,00	**2,89**	0,81	0,85	1,01	0,01	0,01	**1,35**	**2,15**	no	stress response
nudE	b3397	n.d.	no	0,00	0,00	**2,37**	0,76	0,81	0,94	0,47	0,54	**1,11**	**2,13**	no	metabolism
pyrI	b4246	n.d.	no	0,00	0,00	**2,07**	0,00	0,00	1,77	0,77	0,82	**0,97**	**2,13**	no	metabolism
yqjH	b3047	n.d.	no	0,01	0,02	**2,48**	0,06	0,09	1,46	0,35	0,42	**1,21**	**2,05**	no	transport
hisJ	b2309	n.d.	no; H-NS regulated	0,00	0,00	**2,37**	0,00	0,00	2,62	0,11	0,15	**1,18**	**2,00**	yes (-)	transport
**Cpx-independent induction**															
rlmE	b3179	n.d.	no	0,00	0,00	**2,78**	0,00	0,01	0,88	0,00	0,00	**1,47**	**1,89**	no	metabolism
yhaJ	b3105	1,863	no	0,00	0,00	**3,45**	0,38	0,46	1,10	0,00	0,00	**1,84**	**1,87**	no	unknown

hisI	b2018	n.d.	no	0,01	0,02	**4,12**	0,11	0,16	1,92	0,05	0,08	**2,20**	**1,87**	no	metabolism
hha	b0460	n.d.	yes	0,01	0,03	**3,76**	0,76	0,81	1,12	0,08	0,12	**2,02**	**1,86**	no	metabolism
hisG	b2019	1,989	no	0,00	0,01	**2,13**	0,68	0,73	0,95	0,22	0,28	**1,18**	**1,80**	no	metabolism
dxr	b0173	n.d.	no	0,00	0,01	**2,28**	0,81	0,85	0,97	0,10	0,14	**1,31**	**1,74**	no	metabolism
metH	b4019	n.d.	no	0,00	0,00	**2,45**	0,00	0,00	1,37	0,00	0,00	**1,50**	**1,63**	no	metabolism
aroF	b2601	n.d.	no	0,00	0,00	**2,19**	0,86	0,89	0,99	0,02	0,04	**1,36**	**1,61**	no	metabolism
asnB	b0674	n.d.	no	0,00	0,00	**2,29**	0,00	0,00	1,41	0,00	0,00	**1,43**	**1,60**	no	metabolism
pal	b0741	n.d.	no	0,00	0,01	**2,62**	0,85	0,89	0,96	0,02	0,03	**1,76**	**1,48**	no	PG
dnaG	b3066	n.d.	no	0,04	0,08	**2,34**	0,16	0,21	1,64	0,18	0,24	**1,62**	**1,44**	no	metabolism
pstB	b3725	n.d.	no	0,06	0,11	**2,31**	0,63	0,70	1,17	0,37	0,44	**1,61**	**1,43**	no	metabolism
lysC	b4024	n.d.	no	0,00	0,00	**2,94**	0,12	0,17	1,23	0,00	0,00	**2,07**	**1,42**	no	metabolism
asd	b3433	n.d.	no	0,00	0,00	**2,12**	0,00	0,00	1,38	0,00	0,00	**1,53**	**1,39**	no	metabolism
yjeP	b4159	n.d.	yes (P; De Wulf et al.	0,01	0,02	**3,77**	0,21	0,28	1,47	0,01	0,02	**2,79**	**1,35**	no	transport
metN	b0199	n.d.	no	0,00	0,00	**2,48**	0,04	0,07	1,19	0,00	0,00	**1,84**	**1,35**	no	metabolism
gltB	b3212	n.d.	no	0,00	0,00	**2,58**	0,00	0,00	2,33	0,00	0,00	**1,91**	**1,35**	no	metabolism
metQ	b0197	n.d.	no	0,00	0,00	**2,27**	0,03	0,06	1,30	0,00	0,00	**1,69**	**1,34**	no	transport
gltD	b3213	n.d.	no	0,00	0,00	**2,43**	0,00	0,00	2,23	0,00	0,00	**1,81**	**1,34**	no	metabolism
secA	b0098	n.d.	yes (P; De Wulf et al.	0,00	0,00	**2,80**	0,00	0,00	0,60	0,00	0,00	**2,14**	**1,31**	yes (+)	transport
ybbN	b0492	n.d.	no	0,00	0,00	**2,37**	0,05	0,08	1,24	0,00	0,00	**1,83**	**1,30**	no	metabolism
gdhA	b1761	n.d.	no	0,00	0,00	**2,33**	0,00	0,00	1,36	0,00	0,00	**1,80**	**1,30**	no	metabolism
sufB	b1683	n.d.	no	0,00	0,01	**2,35**	0,01	0,01	1,62	0,00	0,01	**1,83**	**1,28**	no	metabolism
ybeZ	b0660	n.d.	no	0,00	0,00	**2,18**	0,01	0,03	1,13	0,00	0,00	**1,75**	**1,24**	no	unknown
hslO	b3401	n.d.	no	0,00	0,00	**2,36**	0,07	0,11	1,17	0,00	0,00	**1,93**	**1,23**	no	stress response
yfbQ	b2290	n.d.	no	0,00	0,00	**2,01**	0,00	0,00	1,36	0,00	0,00	**1,64**	**1,23**	no	metabolism
hslU	b3931	n.d.	no	0,00	0,00	**2,13**	0,06	0,09	1,06	0,00	0,00	**1,76**	**1,21**	no	stress response
nlpE	b0192	n.d.	no	0,00	0,00	**250,41**	0,00	0,01	1,87	0,00	0,00	**210,80**	**1,19**	yes (+)	metabolism
ilvE	b3770	n.d.	no	0,00	0,00	**2,63**	0,00	0,00	2,39	0,00	0,00	**2,26**	**1,16**	no	metabolism
dnaK	b0014	n.d.	yes (P; De Wulf et al.	0,00	0,00	**2,16**	0,56	0,63	0,99	0,00	0,00	**1,88**	**1,15**	no	stress response
groL	b4143	n.d.	no	0,00	0,00	**2,24**	0,03	0,06	1,12	0,00	0,00	**1,96**	**1,15**	no	stress response
pyrH	b0171	n.d.	no	0,02	0,04	**2,59**	0,01	0,01	2,08	0,00	0,01	**2,31**	**1,12**	no	metabolism
lpxL	b1054	n.d.	no	0,00	0,01	**2,10**	0,84	0,87	0,97	0,00	0,01	**1,87**	**1,12**	no	metabolism
lacI	b0345	n.d.	no	0,00	0,00	**52,00**	0,16	0,22	1,12	0,00	0,00	**47,07**	**1,10**	yes (+)	metabolism
ibpA	b3687	1,594	no	0,00	0,00	**2,68**	0,56	0,62	0,95	0,00	0,00	**2,43**	**1,10**	yes (+)	stress response
pspA	b1304	1,901	no	0,01	0,02	**2,78**	0,88	0,90	1,02	0,00	0,01	**2,52**	**1,10**	yes (+)	stress response
yrbC	b3192	n.d.	no	0,00	0,01	**2,31**	0,00	0,01	2,36	0,01	0,01	**2,21**	**1,04**	no	transport
add	b1623	n.d.	no	0,00	0,00	**2,16**	0,00	0,00	1,44	0,00	0,00	**2,10**	**1,03**	no	metabolism
metE	b3829	n.d.	no	0,00	0,00	**5,12**	0,00	0,00	5,70	0,00	0,00	**4,97**	**1,03**	no	metabolism
ibpB	b3686	n.d.	no	0,00	0,00	**2,51**	0,15	0,21	0,86	0,00	0,00	**2,49**	**1,01**	yes (+)	stress response
rpsS	b3316	n.d.	no	0,01	0,02	**2,20**	0,03	0,05	1,72	0,01	0,02	**2,20**	**1,00**	no	metabolism
ycjX	b1321	n.d.	no	0,00	0,00	**2,62**	0,80	0,84	1,03	0,00	0,00	**2,64**	**1,00**	yes (+)	metabolism
ahpC	b0605	n.d.	no	0,01	0,02	**2,68**	0,02	0,03	1,80	0,00	0,00	**2,71**	**0,99**	no	metabolism
sufC	b1682	n.d.	no	0,01	0,02	**2,95**	0,01	0,02	2,78	0,01	0,01	**2,99**	**0,99**	no	metabolism
nudK	b2467	n.d.	no	0,01	0,03	**4,11**	0,01	0,02	3,27	0,00	0,00	**4,25**	**0,97**	no	metabolism
ycjF	b1322	n.d.	no	0,00	0,00	**2,79**	0,49	0,56	1,10	0,00	0,00	**2,99**	**0,93**	yes (+)	stress response
ghrA	b1033	n.d.	no	0,00	0,00	**2,35**	0,00	0,00	1,73	0,00	0,00	**2,54**	**0,92**	no	metabolism
cysK	b2414	n.d.	no	0,00	0,01	**2,05**	0,00	0,00	1,27	0,00	0,00	**2,25**	**0,91**	no	metabolism
udp	b3831	n.d.	no	0,00	0,00	**2,65**	0,00	0,00	2,55	0,00	0,00	**2,94**	**0,90**	no	metabolism
yggN	b2958	n.d.	no	0,02	0,04	**2,75**	0,00	0,01	3,59	0,01	0,01	**3,35**	**0,82**	no	unknown
yifL	b4558	n.d.	no	0,03	0,06	**2,26**	0,00	0,00	4,06	0,01	0,01	**2,78**	**0,81**	no	unknown
fliY	b1920	n.d.	no	0,02	0,05	**2,18**	0,00	0,01	3,08	0,01	0,01	**2,88**	**0,76**	no	transport
rpsF	b4200	n.d.	no	0,03	0,06	**2,67**	0,01	0,02	2,58	0,00	0,01	**3,54**	**0,75**	no	metabolism
ydhQ	b1664	n.d.	no	0,00	0,01	**2,83**	0,01	0,01	1,22	0,00	0,00	**4,37**	**0,65**	yes (+)	unknown
rpsO	b3165	n.d.	no	0,04	0,07	**2,63**	0,02	0,03	2,80	0,00	0,01	**4,30**	**0,61**	no	metabolism
dnaJ	b0015	n.d.	no	0,00	0,00	**2,01**	0,00	0,00	2,09	0,00	0,00	**3,95**	**0,51**	no	stress response
gnsB	b1550	n.d.	no	0,04	0,08	**2,13**	0,00	0,01	3,63	0,00	0,00	**5,44**	**0,39**	no	metabolism
ftsB	b2748	n.d.	no	0,35	0,45	**2,05**	0,01	0,02	4,45	0,01	0,01	**5,54**	**0,37**	no	cell division
psiF	b0384	n.d.	no	0,44	0,53	**2,42**	0,00	0,00	76,42	0,00	0,00	**83,36**	**0,03**	no	stress response
