# Supplementary material for: Molecular and proteome analyses highlight the importance of the Cpx envelope stress system for acid stress and cell wall stability in Escherichia coli
Source: Microbiologyopen. 2016 Apr 2;5(4):582–96. doi: 10.1002/mbo3.353 (PMC4985592; doi:10.1002/mbo3.353)
Supplement: Supplementary file 8 [file MBO3-5-582-s008.pdf]

Table S4B: Comparison of transcriptome and proteome data of Cpx-TCS target proteins.

All proteins being Cpx-dependently/-independently induced (S4A) or inhibited (S4B) are listed in comparison with the WT_{ON}/WT-ratios of transcriptome data from Raivio *et al.* , 2013. Furthermore, information on known CpxR~P binding motifs is provided. For each protein, the following ratios were calculated using the protein intensities measured in this study: WT_{ON}/WT (compares protein abundance between induced and non-induced WT); *cpxRA* /WT (compares protein abundance between non-induced *cpxRA* -strain and non-induced WT); *cpxRA* $_{ON}$/WT (compares protein abundance between induced *cpxRA* -strain and non-induced WT). To emphasize the higher amount (Cpx-specific induction) of proteins in induced WT-cells compared to induced *cpxRA* -cells we additionally calculated the [WT_{ON}/WT / *cpxRA* $_{ON}$/WT]-ratio (S4A). To highlight the higher amount (Cpx-specific inhibition) of proteins in induced *cpxRA* -cells compared to induced WT-cells we additionally calculated the [*cpxRA* $_{ON}$/WT / WT_{ON}/WT]-ratio (S4B). For each ratio minimum 2-fold difference is defined as significant. Calculating the difference between induced and non-induced *cpxRA* -cells, we checked whether *nlpE* -overexpression has an additional effect on the relative amounts of proteins. Minimum 2-fold difference (+) or (-) was defined as an additional NlpE-effect (S4A,B).

gene symbol	gene bank	Raivio mRNA NlpE/WT (MC4100 in LB)	CpxR~P motif	WT_{ON} p-value	WT_{ON} q-value (BH)	WT_{ON}/WT-ratio	*cpxAR* p-value	*cpxAR* q-value (BH)	*cpxAR* /WT-ratio	*cpxAR* $_{ON}$ p-value	*cpxAR* $_{ON}$ q-value (BH)	*cpxAR* $_{ON}$ /WT-ratio	x-fold more in induced cpxRA-strain compared to induced WT [$cpxAR_{ON}$/WT]/ WT_{ON}/WT]-ratio	additional NlpE-effect (minimum 2-fold difference between induced and non-induced deletion mutant): positive (+) or negative (-)	global function
**Cpx-dependent inhibition**															
gadC	b1492	n.d.	no; H-NS regulated	0,00	0,00	**0,08**	0,00	0,00	1,58	0,00	0,00	**1,72**	**20,94**	no	stress response
gadA	b3517	n.d.	no; H-NS regulated	0,00	0,00	**0,06**	0,02	0,04	1,29	0,44	0,51	**1,06**	**16,93**	no	stress response
hdeA	b3510	n.d.	no; H-NS regulated	0,00	0,01	**0,25**	0,00	0,00	6,58	0,01	0,01	**2,90**	**11,73**	yes (-)	stress response
hdeB	b3509	n.d.	no; H-NS regulated	0,00	0,00	**0,36**	0,00	0,00	10,16	0,00	0,00	**3,44**	**9,58**	yes (-)	stress response
hdeD	b3511	n.d.	no; H-NS regulated	0,01	0,03	**0,48**	0,00	0,00	3,43	0,00	0,00	**4,53**	**9,42**	no	stress response
cadA	b4131	n.d.	no; H-NS regulated	0,00	0,00	**0,48**	0,05	0,08	1,33	0,00	0,00	**3,75**	**7,76**	yes (+)	stress response
yjbJ	b4045	n.d.	no	0,00	0,00	**0,17**	0,76	0,81	1,04	0,64	0,69	**1,05**	**6,23**	no	stress response
cydB	b0734	n.d.	no; H-NS regulated	0,00	0,01	**0,37**	0,00	0,00	1,66	0,00	0,00	**2,04**	**5,58**	no	metabolism
cydA	b0733	n.d.	no; H-NS regulated	0,00	0,00	**0,35**	0,00	0,00	1,44	0,00	0,00	**1,53**	**4,32**	no	metabolism
hpf	b3203	n.d.	no	0,00	0,00	**0,41**	0,80	0,84	1,08	0,00	0,00	**1,70**	**4,18**	no	metabolism
osmC	b1482	n.d.	no; H-NS regulated	0,00	0,00	**0,28**	0,01	0,02	1,16	0,00	0,01	**1,16**	**4,07**	no	stress response
skp	b0178	n.d.	yes	0,00	0,00	**0,30**	0,01	0,02	1,16	0,03	0,04	**1,18**	**3,95**	no	stress response
yecM	b1875	n.d.	no	0,00	0,01	**0,43**	0,59	0,66	1,08	0,00	0,01	**1,62**	**3,80**	no	metabolism
yfcD	b2299	n.d.	no	0,01	0,02	**0,44**	0,00	0,00	1,68	0,00	0,00	**1,61**	**3,67**	no	unknown
ycaC	b0897	n.d.	no	0,00	0,00	**0,29**	0,00	0,00	1,63	0,45	0,52	**1,07**	**3,64**	no	metabolism
ybiB	b0800	n.d.	no	0,00	0,00	**0,36**	0,01	0,03	1,29	0,03	0,06	**1,29**	**3,57**	no	unknown
minD	b1175	n.d.	no	0,00	0,00	**0,36**	0,86	0,89	1,01	0,00	0,00	**1,20**	**3,32**	no	cell division
slp	b3506	n.d.	no; H-NS regulated	0,00	0,00	**0,32**	0,04	0,07	1,22	0,43	0,50	**1,04**	**3,23**	no	stress response
minE	b1174	0,563	no	0,00	0,00	**0,36**	0,31	0,39	0,93	0,14	0,19	**1,10**	**3,01**	no	cell division
ygiW	b3024	n.d.	no	0,00	0,00	**0,27**	0,00	0,00	1,53	0,05	0,07	**0,79**	**2,87**	no	stress response
yccU	b0965	n.d.	no	0,00	0,00	**0,47**	0,01	0,02	1,49	0,05	0,08	**1,34**	**2,84**	no	metabolism
dps	b0812	n.d.	no; H-NS regulated	0,00	0,00	**0,17**	0,00	0,00	0,58	0,00	0,00	**0,49**	**2,83**	no	metabolism
hchA	b1967	n.d.	no; H-NS regulated	0,00	0,00	**0,50**	0,00	0,00	1,30	0,00	0,00	**1,39**	**2,78**	no	metabolism
emrA	b2685	0,548	no	0,00	0,00	**0,27**	0,03	0,05	0,77	0,01	0,03	**0,74**	**2,72**	no	transport
yggL	b2959	n.d.	no	0,01	0,03	**0,39**	0,12	0,17	0,72	0,95	0,96	**1,01**	**2,62**	no	unknown
pdxK	b2418	0,505	no	0,00	0,01	**0,42**	0,63	0,69	0,92	0,90	0,92	**1,02**	**2,46**	no	metabolism
ydgT	b1625	n.d.	no	0,00	0,01	**0,50**	0,39	0,47	1,08	0,08	0,12	**1,20**	**2,41**	no	metabolism
ftn	b1905	n.d.	no; H-NS regulated	0,00	0,00	**0,33**	0,00	0,00	0,66	0,01	0,02	**0,77**	**2,33**	no	transport
**Cpx-independent inhibition**															
moaB	b0782	n.d.	no	0,00	0,00	**0,45**	0,00	0,00	0,58	0,17	0,23	**0,89**	**1,96**	no	metabolism
ppiC	b3775	n.d.	no	0,00	0,00	**0,46**	0,01	0,01	1,42	0,52	0,58	**0,89**	**1,91**	no	metabolism
ppsR	b1703	n.d.	no	0,01	0,02	**0,46**	0,04	0,07	0,79	0,09	0,14	**0,86**	**1,89**	no	metabolism
phnB	b4107	n.d.	no	0,00	0,01	**0,49**	0,48	0,55	1,08	0,42	0,49	**0,91**	**1,88**	no	metabolism
ompF	b0929	0,196	yes	0,00	0,00	**0,49**	0,00	0,00	0,53	0,28	0,35	**0,91**	**1,85**	no	transport
yegP	b2080	n.d.	no	0,00	0,00	**0,49**	0,08	0,12	1,23	0,32	0,38	**0,87**	**1,78**	no	unknown
ydgH	b1604	n.d.	no	0,00	0,00	**0,49**	0,00	0,01	1,21	0,07	0,11	**0,85**	**1,73**	no	unknown
ybaY	b0453	n.d.	no	0,00	0,00	**0,45**	0,03	0,05	1,24	0,01	0,01	**0,77**	**1,72**	no	stress response
cspC	b1823	n.d.	no	0,00	0,00	**0,43**	0,02	0,03	0,77	0,00	0,00	**0,70**	**1,66**	no	stress response
ecnB	b4411	0,502	no	0,00	0,00	**0,47**	0,40	0,47	1,08	0,02	0,04	**0,76**	**1,64**	no	stress response
slmA	b3641	n.d.	no	0,05	0,09	**0,50**	0,05	0,08	0,80	0,01	0,02	**0,80**	**1,60**	no	cell division
yeeR	b2001	n.d.	no	0,01	0,02	**0,09**	0,00	0,00	0,10	0,00	0,00	**0,14**	**1,56**	no	unknown
queC	b0444	n.d.	no	0,02	0,05	**0,41**	0,05	0,08	0,50	0,14	0,19	**0,63**	**1,54**	no	metabolism
srlB	b2704	n.d.	no; H-NS regulated	0,00	0,00	**0,36**	0,00	0,00	0,36	0,00	0,00	**0,54**	**1,52**	no	metabolism
iraP	b0382	n.d.	no	0,00	0,01	**0,45**	0,00	0,00	0,58	0,01	0,02	**0,66**	**1,47**	no	stress response
mppA	b1329	n.d.	no	0,00	0,01	**0,35**	0,00	0,00	0,55	0,00	0,00	**0,51**	**1,44**	no	transport
dcuA	b4138	n.d.	no	0,00	0,00	**0,37**	0,00	0,00	0,42	0,00	0,00	**0,53**	**1,43**	no	transport
yecF	b1915	n.d.	no	0,00	0,01	**0,43**	0,00	0,00	0,48	0,00	0,01	**0,59**	**1,38**	no	unknown
nuoK	b2279	n.d.	no	0,00	0,00	**0,32**	0,00	0,01	0,50	0,00	0,00	**0,41**	**1,29**	no	metabolism
gatB	b2093	n.d.	no	0,00	0,01	**0,46**	0,09	0,13	0,71	0,02	0,03	**0,59**	**1,29**	no	metabolism
yfjG	b2619	n.d.	no	0,00	0,00	**0,22**	0,00	0,01	0,52	0,00	0,00	**0,28**	**1,27**	no	metabolism
galE	b0759	0,263	no; H-NS regulated	0,00	0,00	**0,48**	0,02	0,04	0,78	0,00	0,00	**0,61**	**1,27**	no	metabolism

yeiT	b2146	n.d.	no	0,00	0,00	**0,17**	0,00	0,00	0,29	0,00	0,00	**0,19**	**1,13**	no	metabolism
stpA	b2669	n.d.	no; H-NS regulated	0,00	0,00	**0,44**	0,00	0,00	0,48	0,00	0,00	**0,48**	**1,10**	no	metabolism
hybA	b2996	n.d.	no	0,00	0,00	**0,21**	0,00	0,00	0,12	0,02	0,04	**0,23**	**1,10**	no	metabolism
hupB	b0440	n.d.	no	0,00	0,01	**0,33**	0,00	0,00	0,34	0,00	0,01	**0,36**	**1,09**	no	transcription
narJ	b1226	n.d.	no	0,00	0,00	**0,09**	0,00	0,00	0,06	0,00	0,00	**0,10**	**1,09**	no	folding
yciE	b1257	n.d.	no; H-NS regulated	0,01	0,03	**0,33**	0,00	0,00	0,56	0,00	0,00	**0,35**	**1,04**	no	stress response
tdcB	b3117	n.d.	no	0,00	0,00	**0,05**	0,00	0,00	0,05	0,00	0,00	**0,05**	**1,03**	no	degradation
gatA	b2094	n.d.	no	0,00	0,00	**0,43**	0,00	0,00	0,54	0,00	0,00	**0,43**	**1,01**	no	metabolism
srlE	b2703	n.d.	no; H-NS regulated	0,00	0,00	**0,47**	0,00	0,00	0,29	0,00	0,00	**0,44**	**0,94**	no	metabolism
yciF	b1258	n.d.	no; H-NS regulated	0,00	0,00	**0,32**	0,02	0,04	0,64	0,00	0,00	**0,31**	**0,94**	yes (-)	stress response
dmsA	b0894	n.d.	no	0,00	0,00	**0,21**	0,00	0,00	0,13	0,00	0,00	**0,20**	**0,93**	no	metabolism
pepT	b1127	n.d.	no	0,00	0,00	**0,46**	0,00	0,00	0,46	0,00	0,00	**0,43**	**0,93**	no	metabolism
glpQ	b2239	n.d.	no	0,00	0,00	**0,20**	0,00	0,00	0,32	0,00	0,00	**0,19**	**0,92**	no	metabolism
napA	b2206	n.d.	no	0,00	0,00	**0,18**	0,00	0,00	0,14	0,00	0,00	**0,16**	**0,91**	no	metabolism
hybC	b2994	n.d.	no	0,00	0,00	**0,23**	0,00	0,00	0,19	0,00	0,00	**0,20**	**0,89**	no	metabolism
yfcZ	b2343	n.d.	no	0,00	0,00	**0,35**	0,00	0,00	0,30	0,00	0,00	**0,30**	**0,87**	no	unknown
fdnH	b1475	n.d.	no	0,00	0,01	**0,33**	0,00	0,00	0,18	0,00	0,00	**0,28**	**0,85**	no	metabolism
yfeD	b2399	0,516	no	0,00	0,01	**0,37**	0,00	0,00	0,36	0,00	0,00	**0,32**	**0,85**	no	transcription
tdcE	b3114	n.d.	no	0,00	0,00	**0,41**	0,00	0,00	0,31	0,00	0,00	**0,35**	**0,85**	no	metabolism
flu	b2000	n.d.	no	0,00	0,00	**0,12**	0,00	0,00	0,09	0,00	0,00	**0,08**	**0,69**	no	transport
glpC	b2243	n.d.	no	0,00	0,00	**0,19**	0,00	0,00	0,17	0,00	0,00	**0,13**	**0,67**	no	metabolism
fumB	b4122	n.d.	no	0,00	0,00	**0,38**	0,00	0,00	0,33	0,00	0,00	**0,24**	**0,63**	no	metabolism
narI	b1227	n.d.	no	0,00	0,00	**0,05**	0,00	0,00	0,02	0,00	0,00	**0,03**	**0,61**	no	metabolism
gldA	b3945	n.d.	no	0,00	0,00	**0,41**	0,00	0,00	0,21	0,00	0,00	**0,25**	**0,61**	no	metabolism
nirB	b3365	n.d.	no	0,00	0,00	**0,17**	0,00	0,00	0,09	0,00	0,00	**0,10**	**0,61**	no	metabolism
ykgE	b0306	n.d.	no	0,00	0,00	**0,21**	0,00	0,00	0,11	0,00	0,00	**0,12**	**0,60**	no	unknown
nikA	b3476	n.d.	no	0,00	0,00	**0,19**	0,00	0,00	0,11	0,00	0,00	**0,11**	**0,59**	no	transport
ykgF	b0307	n.d.	no	0,00	0,00	**0,15**	0,00	0,00	0,05	0,00	0,00	**0,08**	**0,55**	no	unknown
frdA	b4154	n.d.	no	0,00	0,00	**0,20**	0,00	0,00	0,14	0,00	0,00	**0,11**	**0,52**	no	metabolism
garR	b3125	n.d.	no	0,00	0,00	**0,40**	0,00	0,00	0,32	0,00	0,00	**0,21**	**0,52**	no	metabolism
fumA	b1612	n.d.	no	0,00	0,00	**0,45**	0,00	0,00	0,41	0,00	0,00	**0,23**	**0,51**	no	metabolism
fdnG	b1474	n.d.	no	0,00	0,00	**0,31**	0,00	0,00	0,14	0,00	0,00	**0,15**	**0,50**	no	metabolism
glpA	b2241	n.d.	no	0,00	0,00	**0,26**	0,00	0,00	0,18	0,00	0,00	**0,13**	**0,49**	no	metabolism
narK	b1223	n.d.	no	0,00	0,00	**0,15**	0,00	0,00	0,02	0,01	0,02	**0,07**	**0,48**	yes (+)	transport
glpB	b2242	n.d.	no	0,00	0,00	**0,19**	0,00	0,00	0,12	0,00	0,00	**0,09**	**0,48**	no	metabolism
fucI	b2802	n.d.	no	0,00	0,00	**0,29**	0,00	0,00	0,17	0,00	0,00	**0,13**	**0,46**	no	metabolism
glpT	b2240	0,601	no	0,00	0,00	**0,29**	0,00	0,00	0,29	0,00	0,00	**0,12**	**0,43**	yes (-)	transport
ydeN	b1498	n.d.	no	0,00	0,00	**0,17**	0,00	0,00	0,09	0,00	0,00	**0,07**	**0,41**	no	unknown
nrdD	b4238	n.d.	no; H-NS regulated	0,00	0,00	**0,31**	0,00	0,00	0,13	0,00	0,00	**0,13**	**0,40**	no	metabolism
aspA	b4139	n.d.	no	0,00	0,00	**0,32**	0,00	0,00	0,18	0,00	0,00	**0,13**	**0,40**	no	metabolism
malM	b4037	n.d.	no	0,00	0,00	**0,38**	0,00	0,00	0,36	0,00	0,00	**0,14**	**0,38**	yes (-)	metabolism
ydfZ	b1541	n.d.	no	0,00	0,01	**0,12**	0,00	0,00	0,04	0,00	0,00	**0,04**	**0,37**	no	unknown
ansB	b2957	n.d.	no	0,00	0,00	**0,19**	0,00	0,00	0,05	0,00	0,00	**0,07**	**0,37**	no	metabolism
narG	b1224	n.d.	no	0,00	0,00	**0,14**	0,00	0,00	0,04	0,00	0,00	**0,05**	**0,35**	no	metabolism
narH	b1225	n.d.	no	0,00	0,00	**0,13**	0,00	0,00	0,03	0,00	0,00	**0,04**	**0,31**	no	metabolism
malE	b4034	n.d.	no	0,00	0,01	**0,41**	0,00	0,00	0,37	0,00	0,00	**0,12**	**0,29**	yes (-)	transport
malF	b4033	n.d.	no	0,00	0,00	**0,45**	0,00	0,00	0,25	0,00	0,00	**0,13**	**0,29**	yes (-)	transport
mglB	b2150	0,357	no	0,00	0,00	**0,19**	0,00	0,00	0,25	0,00	0,00	**0,05**	**0,28**	yes (-)	transport
ykgG	b0308	n.d.	no	0,00	0,00	**0,20**	0,00	0,00	0,04	0,00	0,00	**0,06**	**0,28**	no	unknown
nanA	b3225	0,487	no	0,00	0,00	**0,48**	0,00	0,00	0,27	0,00	0,00	**0,12**	**0,25**	yes (-)	metabolism
grcA	b2579	n.d.	no	0,00	0,00	**0,27**	0,00	0,00	0,04	0,00	0,00	**0,05**	**0,20**	no	metabolism
nirD	b3366	n.d.	no; H-NS regulated	0,00	0,00	**0,11**	0,00	0,00	0,03	0,00	0,00	**0,02**	**0,16**	no	metabolism
pspE	b1308	n.d.	no	0,01	0,02	**0,35**	0,00	0,00	0,27	0,02	0,04	**0,04**	**0,11**	yes (-)	metabolism
tnaA	b3708	n.d.	no	0,00	0,00	**0,14**	0,00	0,00	0,16	0,00	0,00	**0,01**	**0,11**	yes (-)	metabolism
